# Supplementary material for: Stereoselective synthesis of medium lactams enabled by metal-free hydroalkoxylation/stereospecific [1,3]-rearrangement
Source: Nat Commun. 2019 Jul 19;10:3234. doi: 10.1038/s41467-019-11245-2 (PMC6642132; doi:10.1038/s41467-019-11245-2)
Supplement: Supplementary file 2 — Supplementary Information [file 41467_2019_11245_MOESM2_ESM.pdf]

Diastereo- and enantioselective synthesis of medium lactams enabled by metal-free hydroalkoxylation/stereospecific [1,3]-rearrangement

Zhou et al.

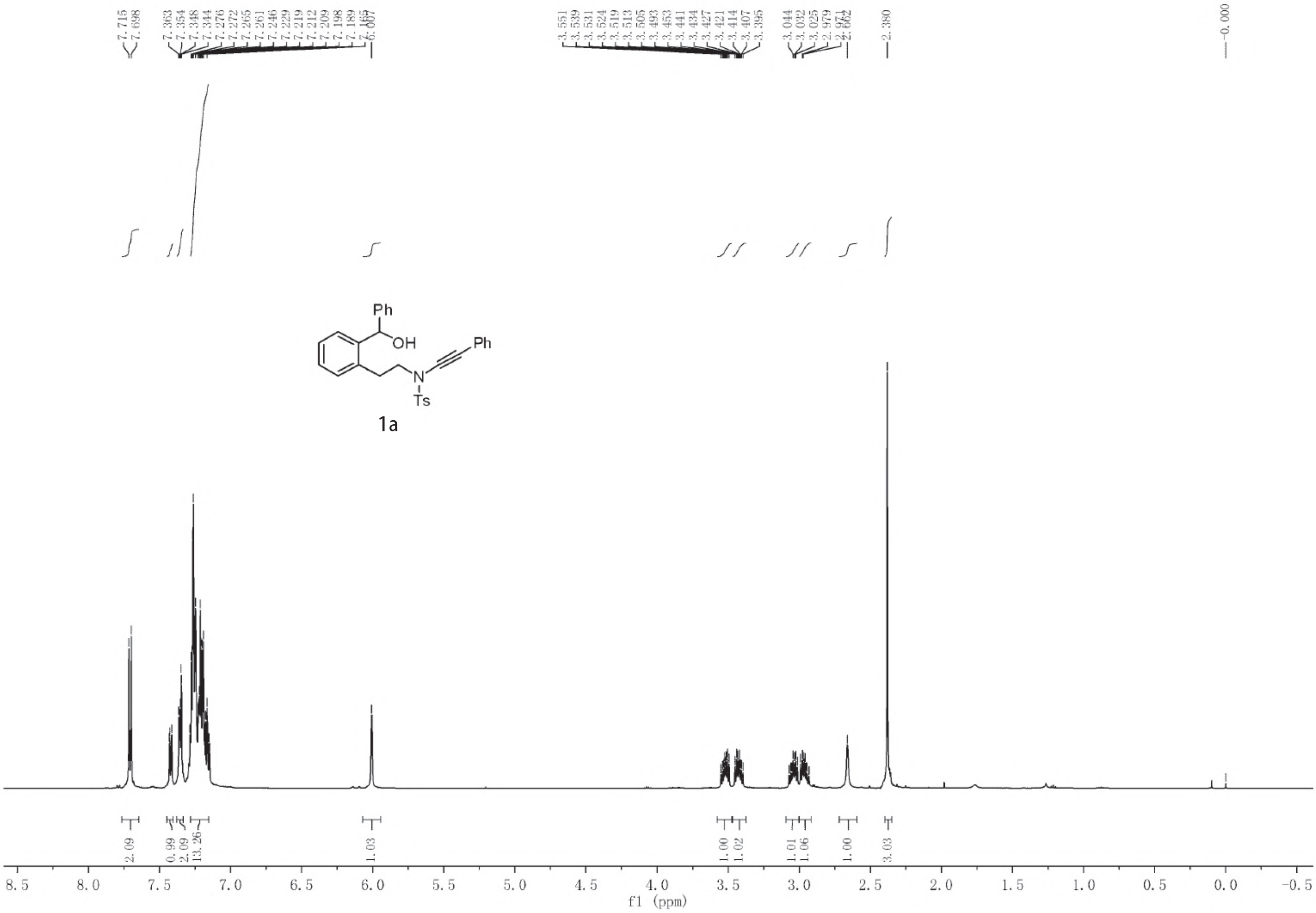

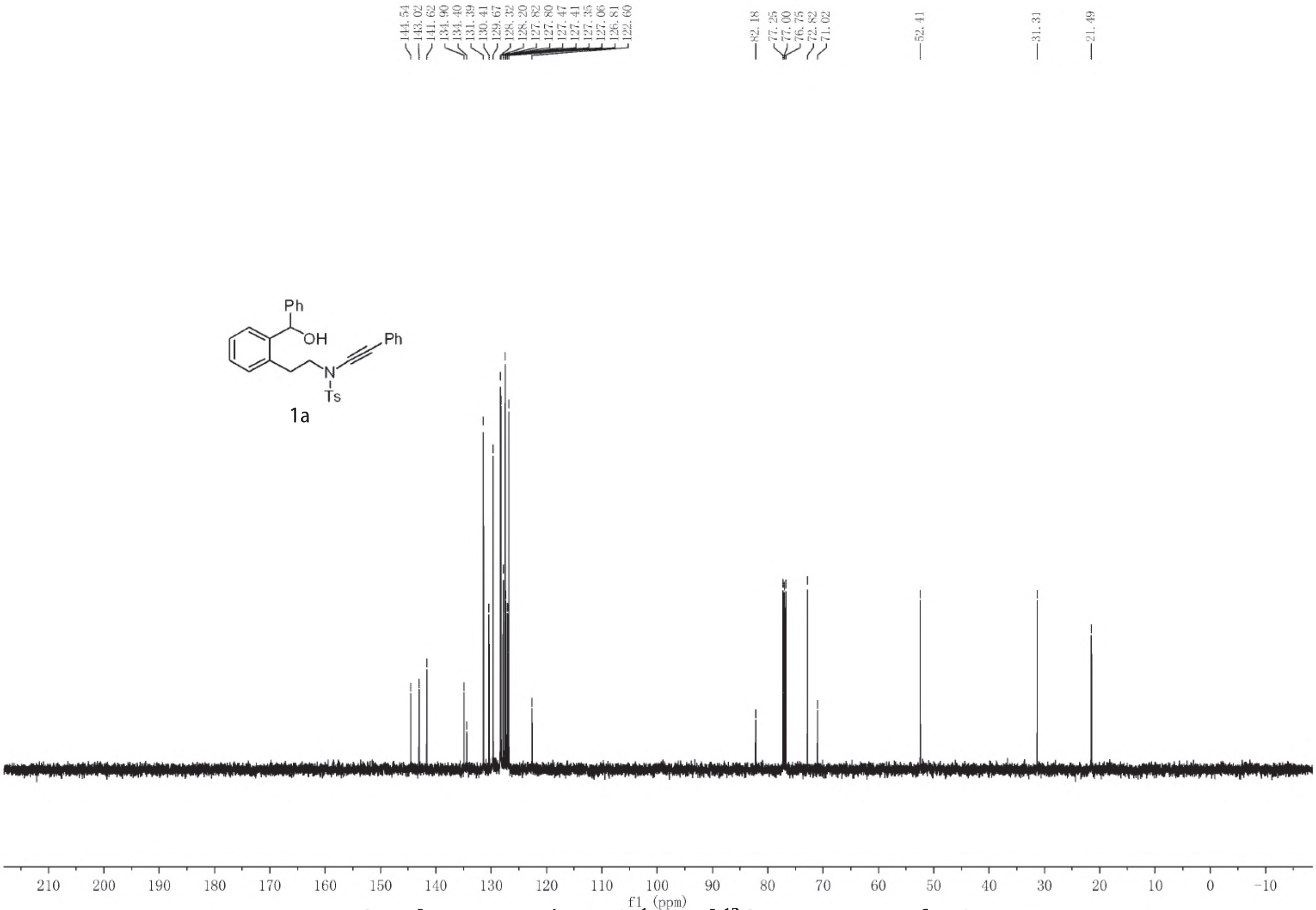

Supplementary Figure 1. ^{1}H and ^{13}C NMR spectra for 1a

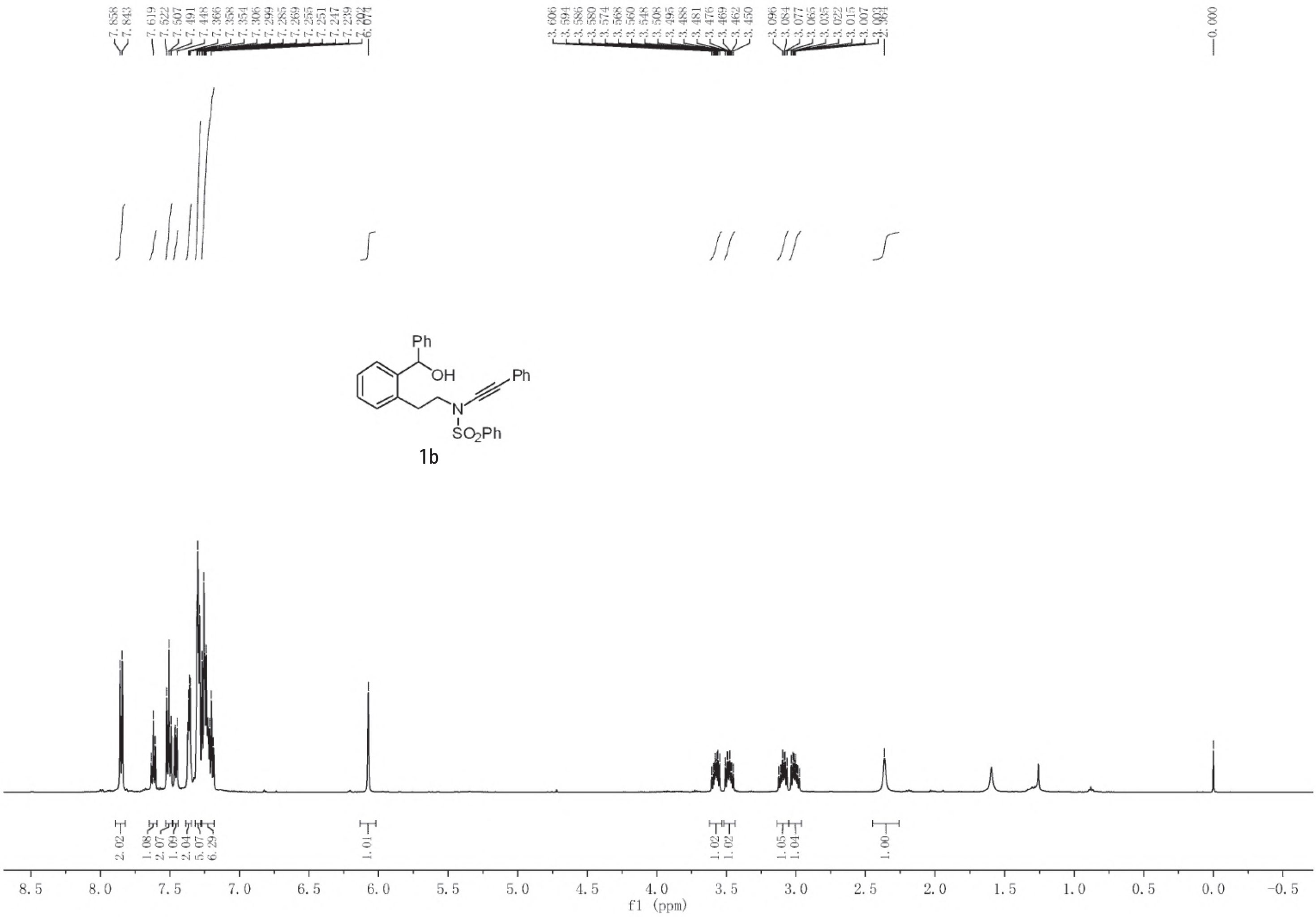

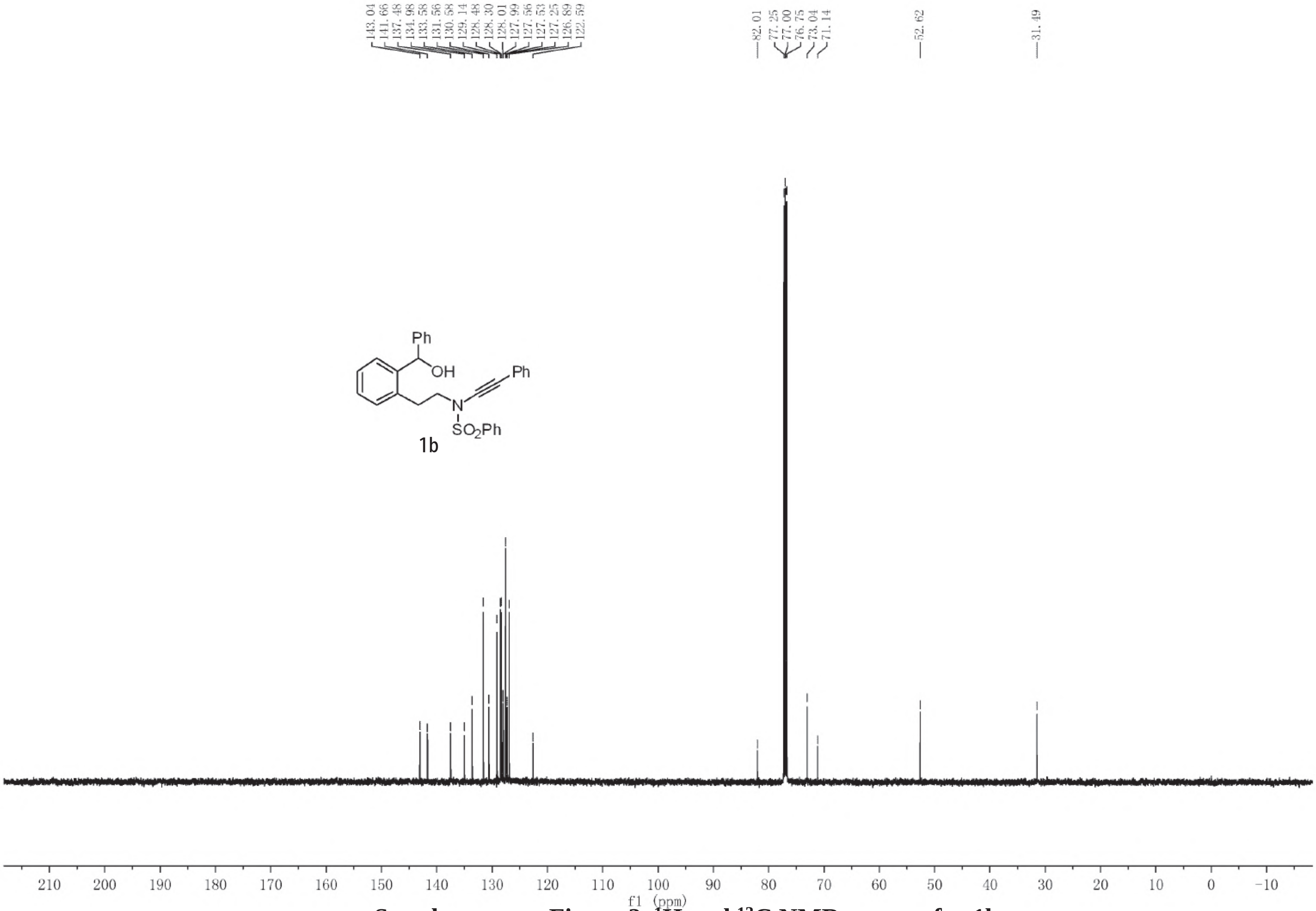

Supplementary Figure 2. ^{1}H and ^{13}C NMR spectra for 1b

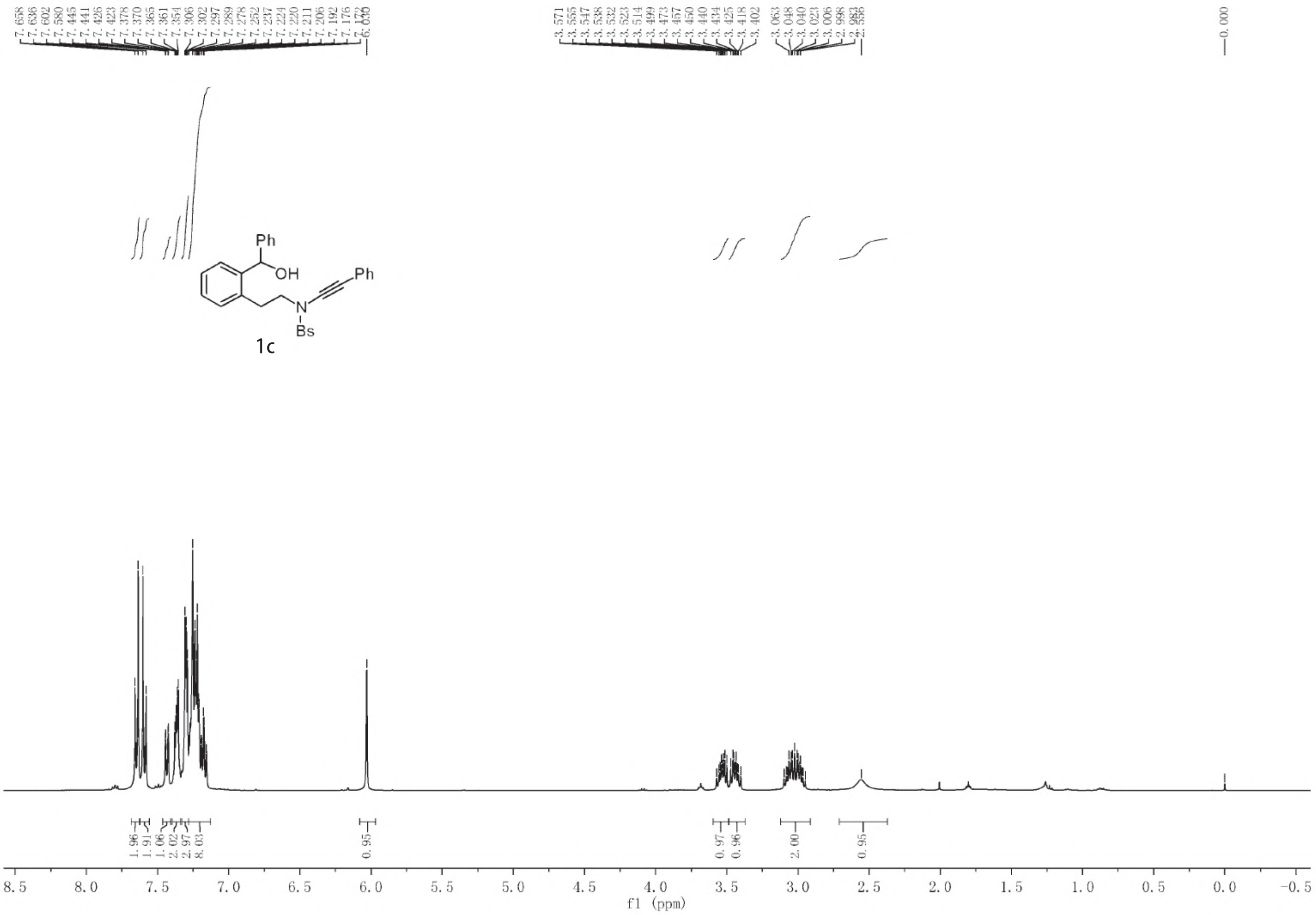

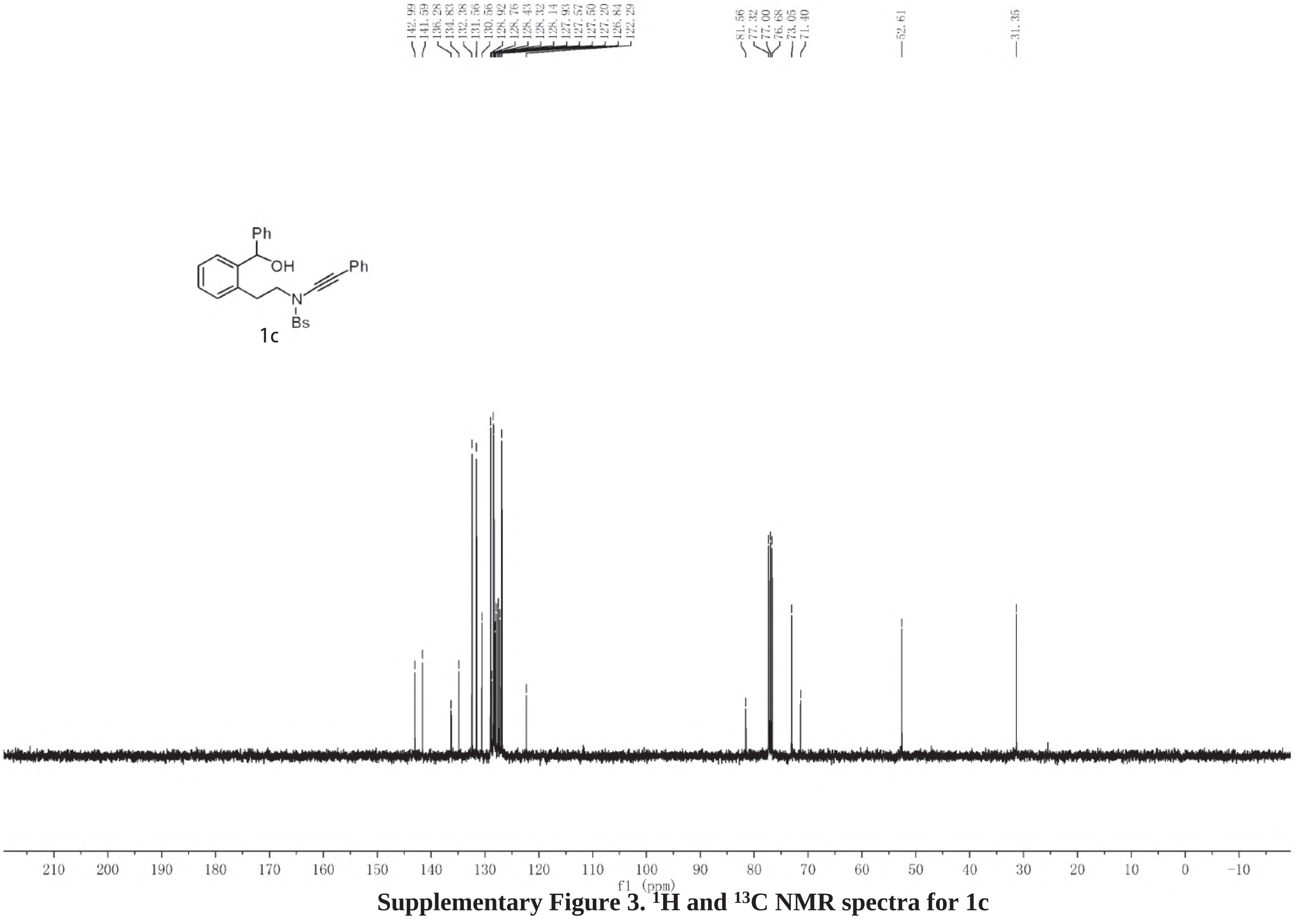

Supplementary Figure 3. ^{1}H and ^{13}C NMR spectra for 1c

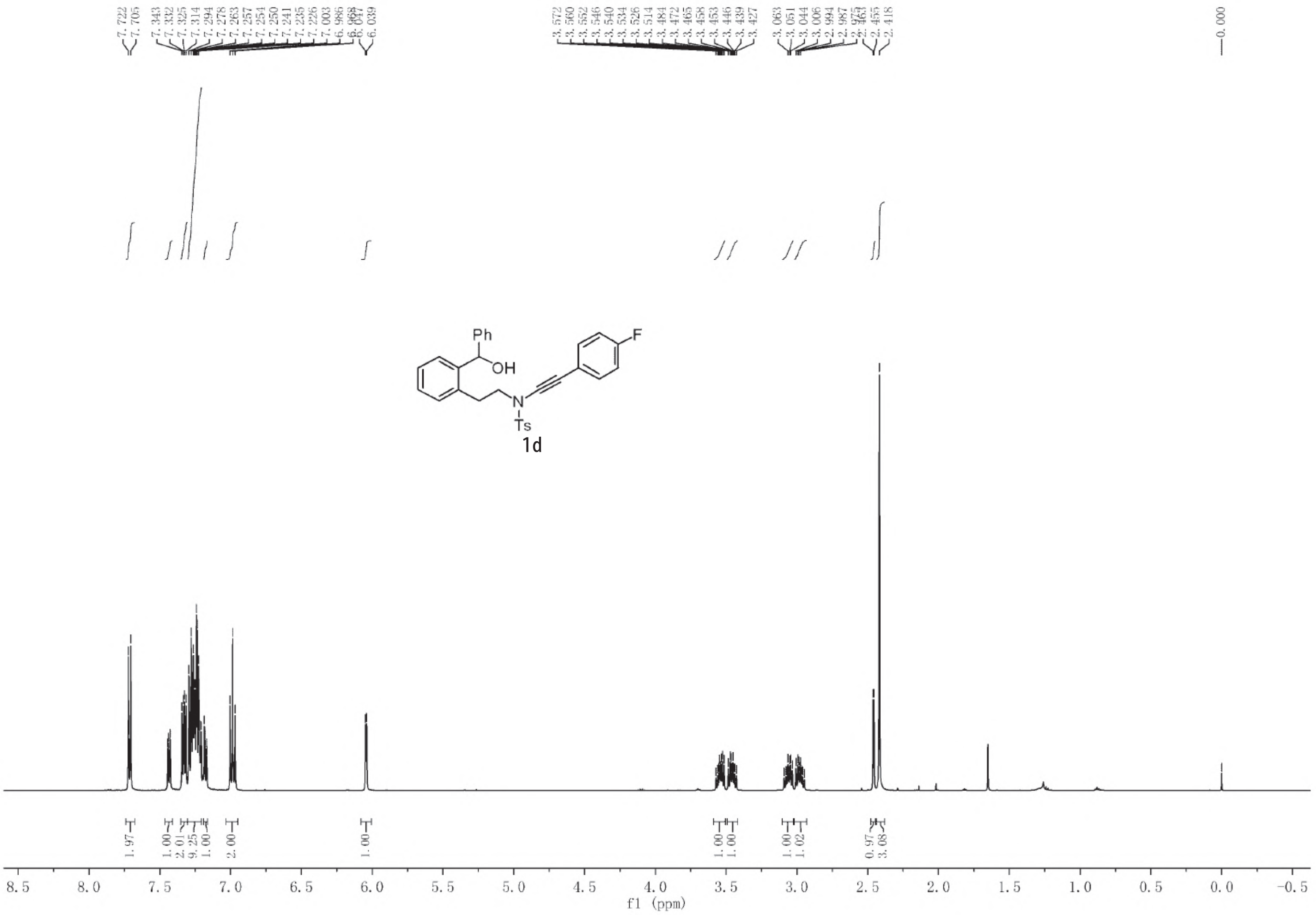

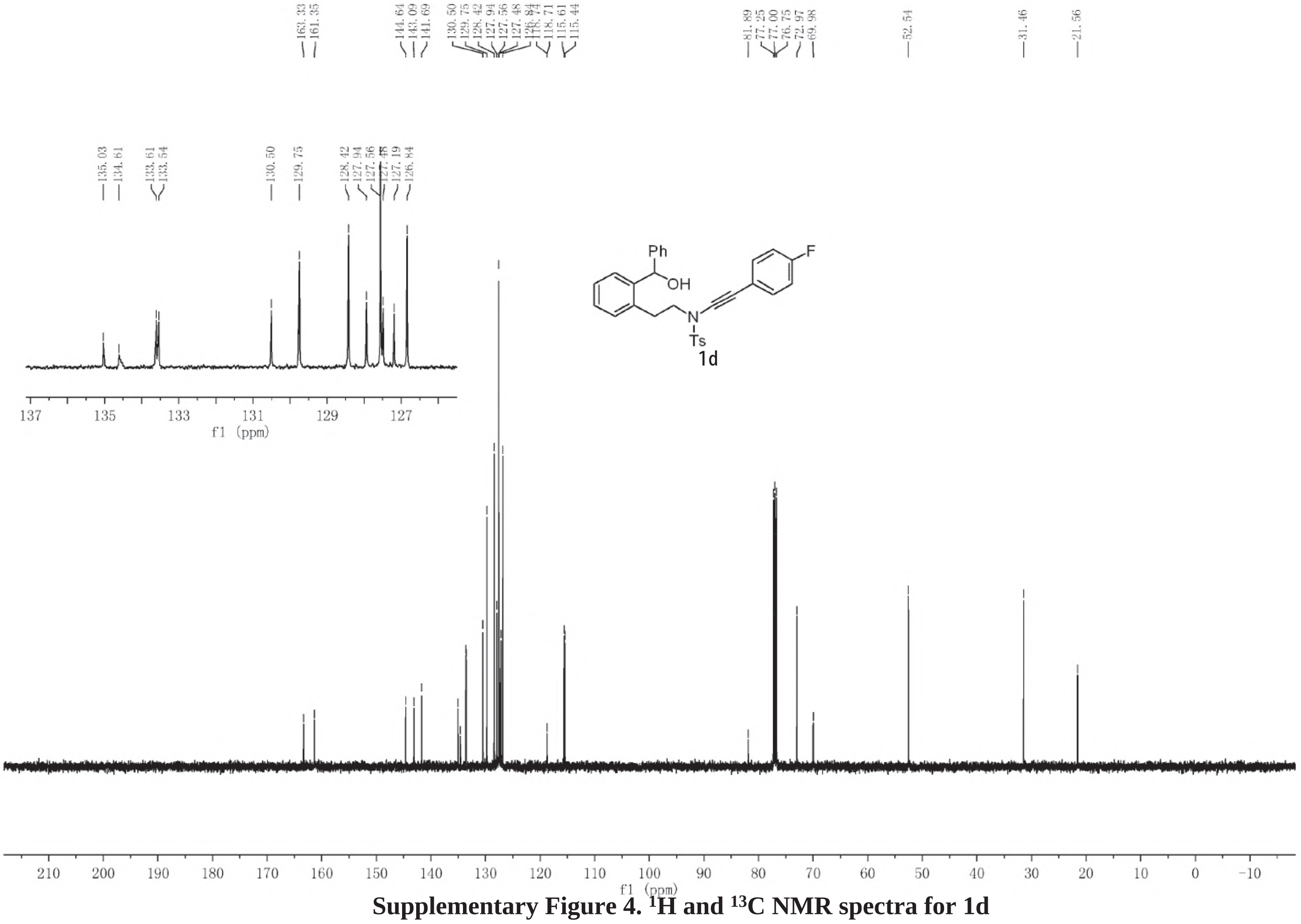

Supplementary Figure 4. ^{1}H and ^{13}C NMR spectra for 1d

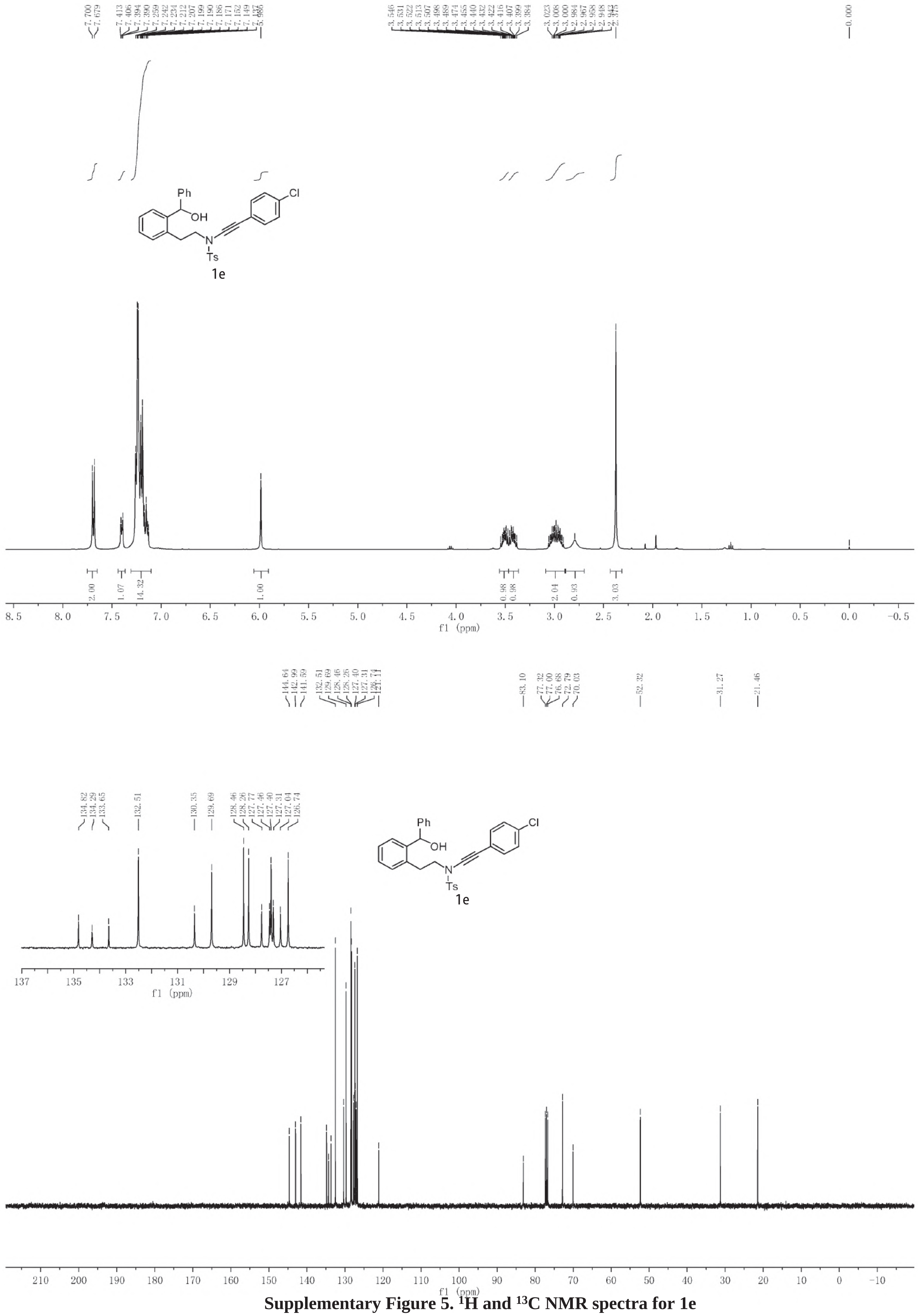

Supplementary Figure 5. ^{1}H and ^{13}C NMR spectra for 1e

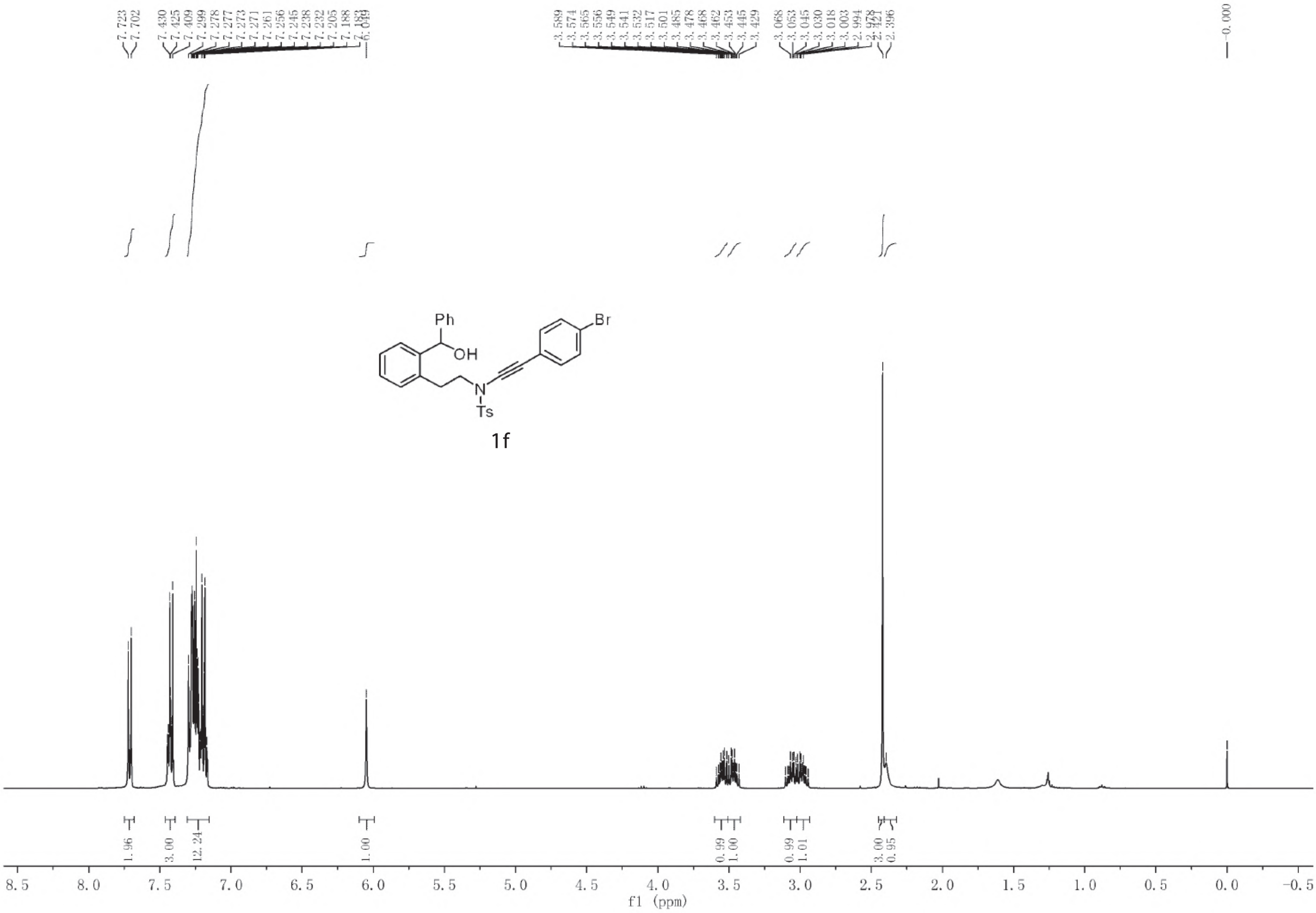

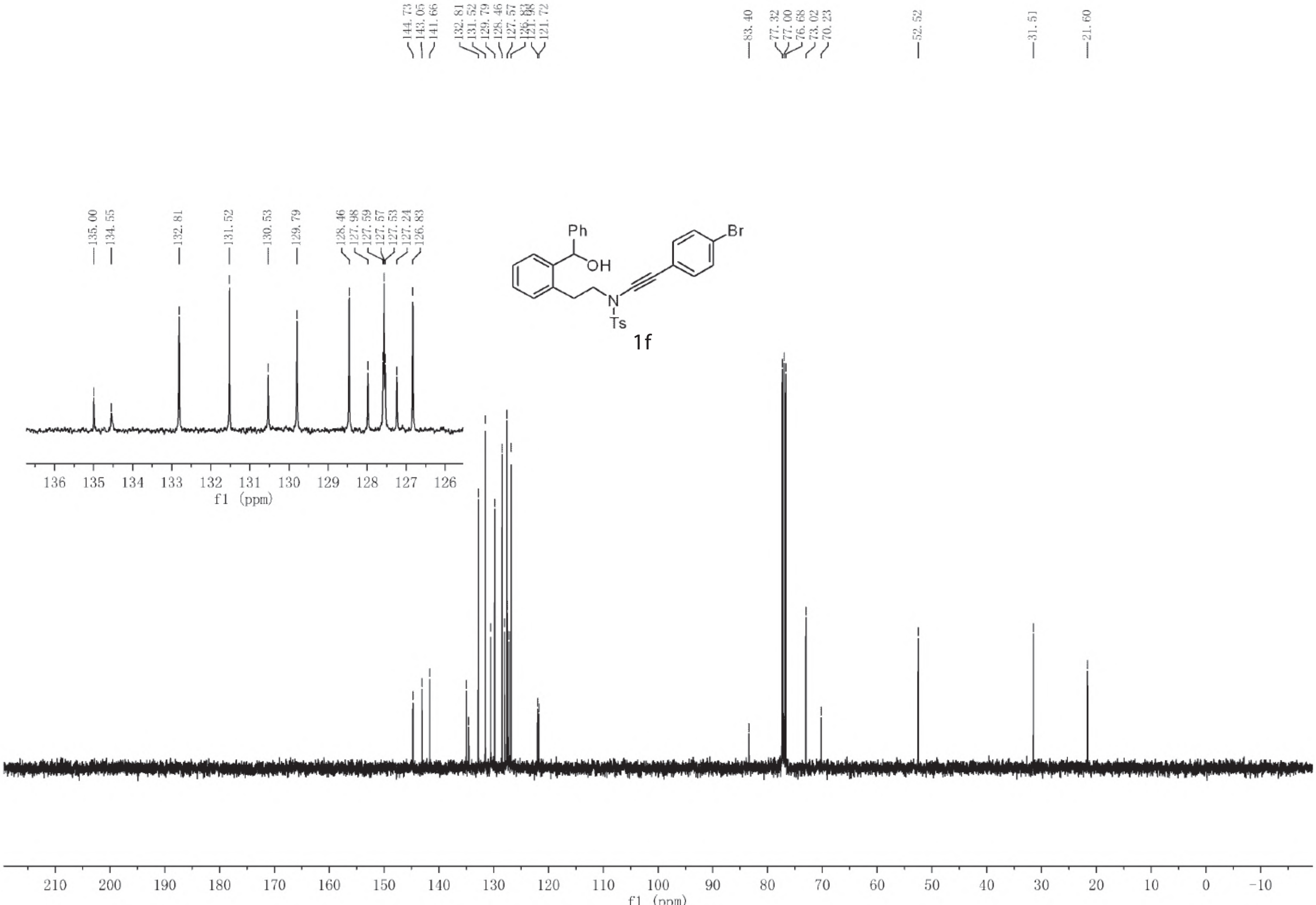

Supplementary Figure 6. ^{1}H and ^{13}C NMR spectra for 1f

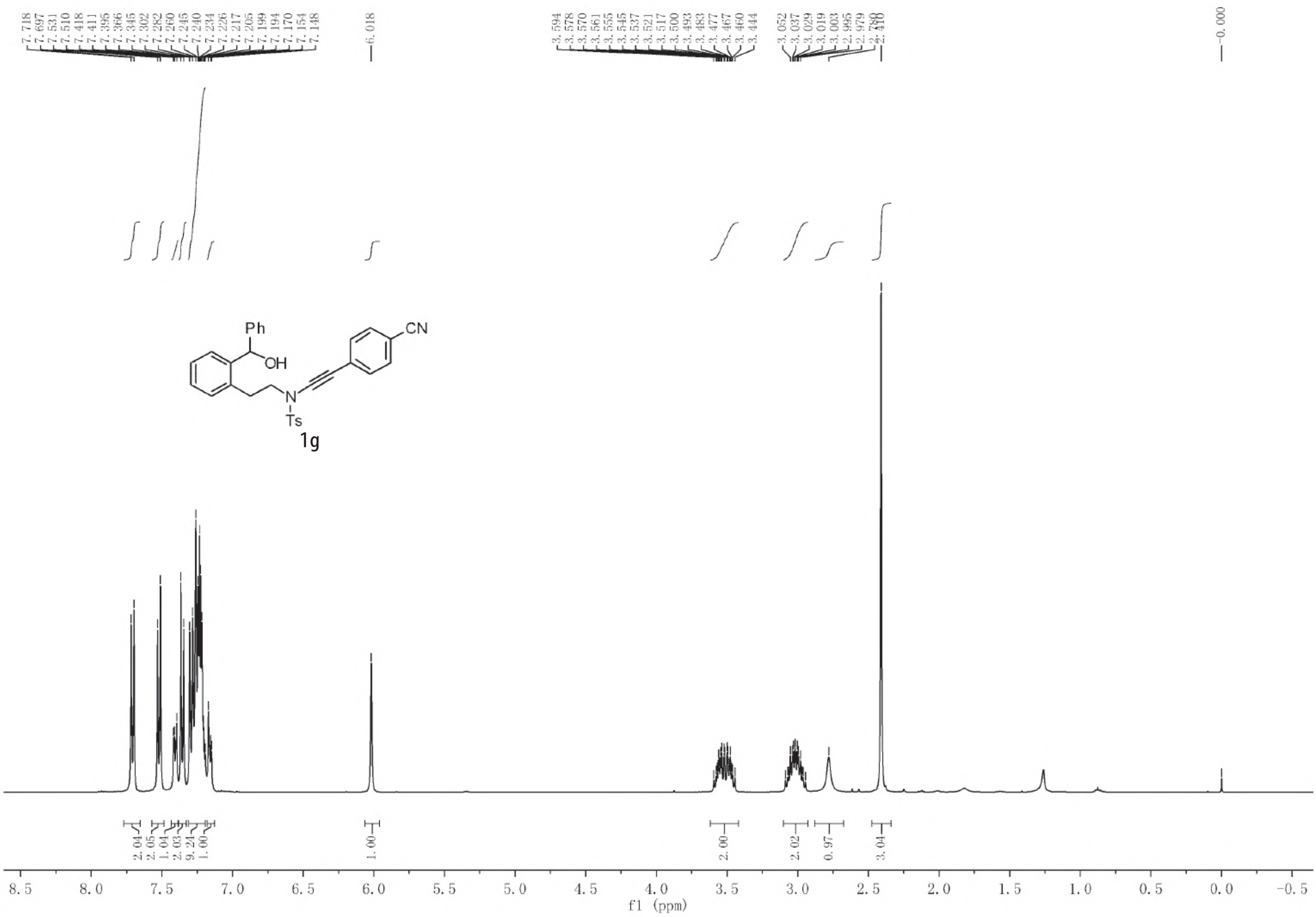

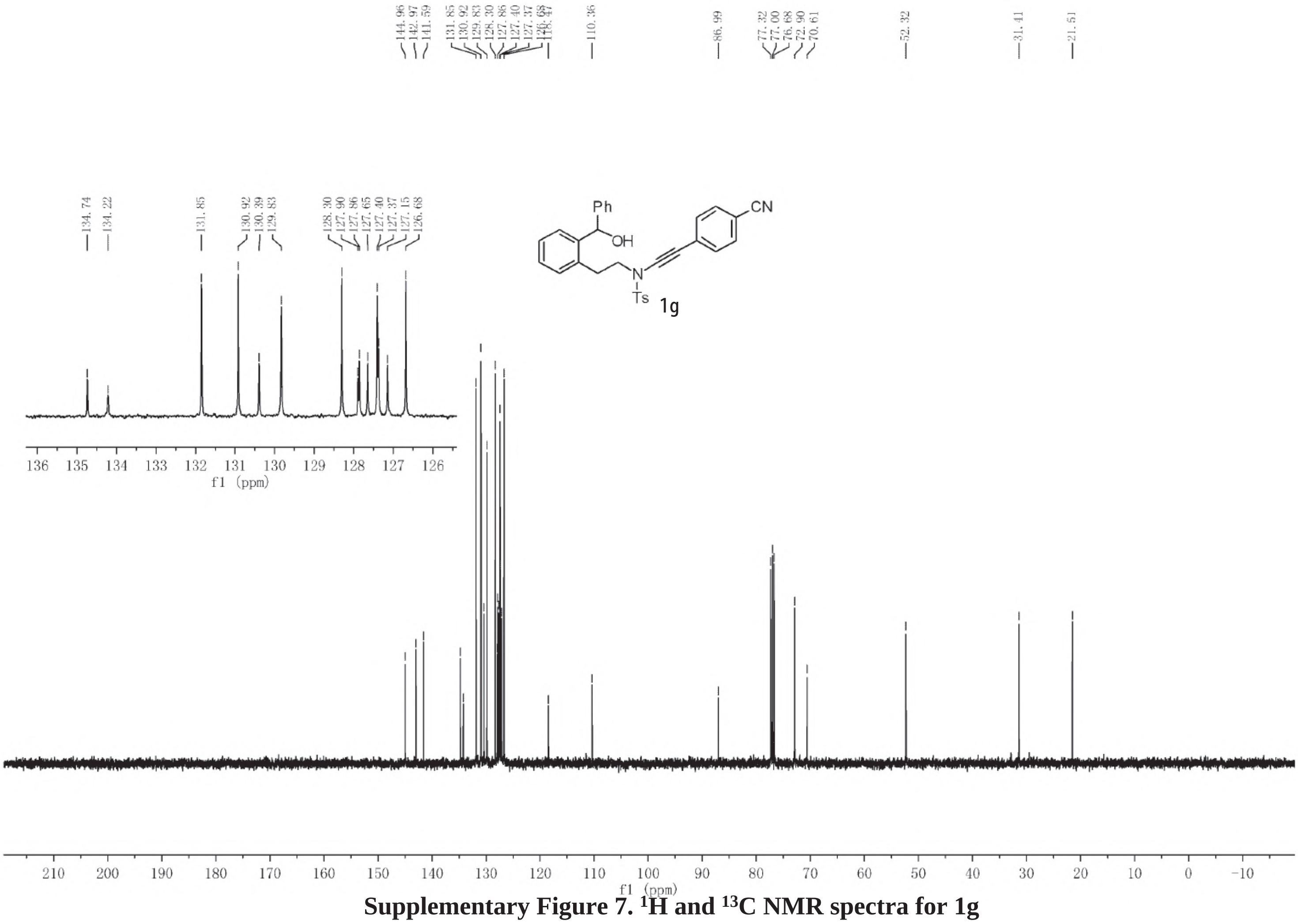

Supplementary Figure 7. ^{1}H and ^{13}C NMR spectra for 1g

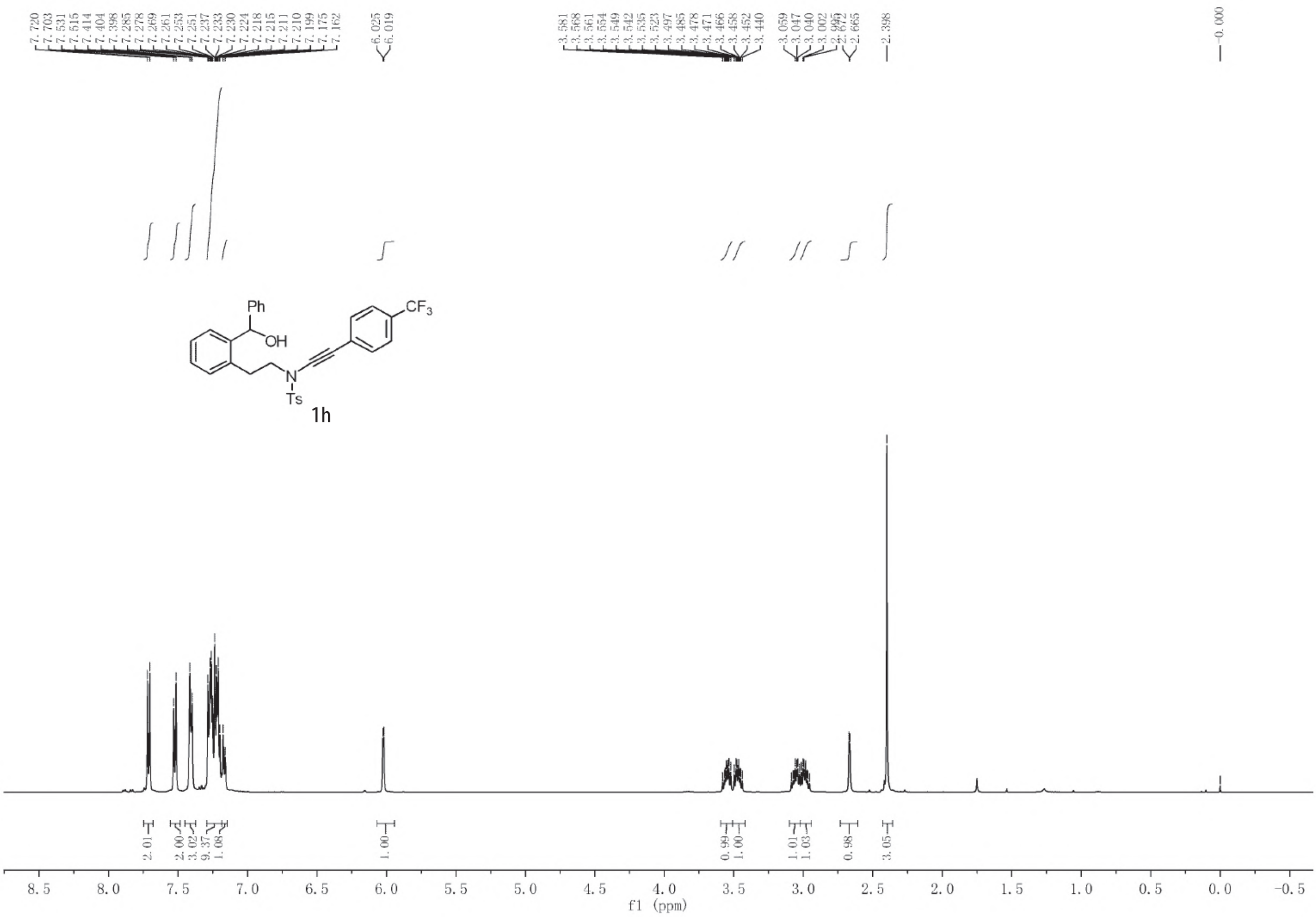

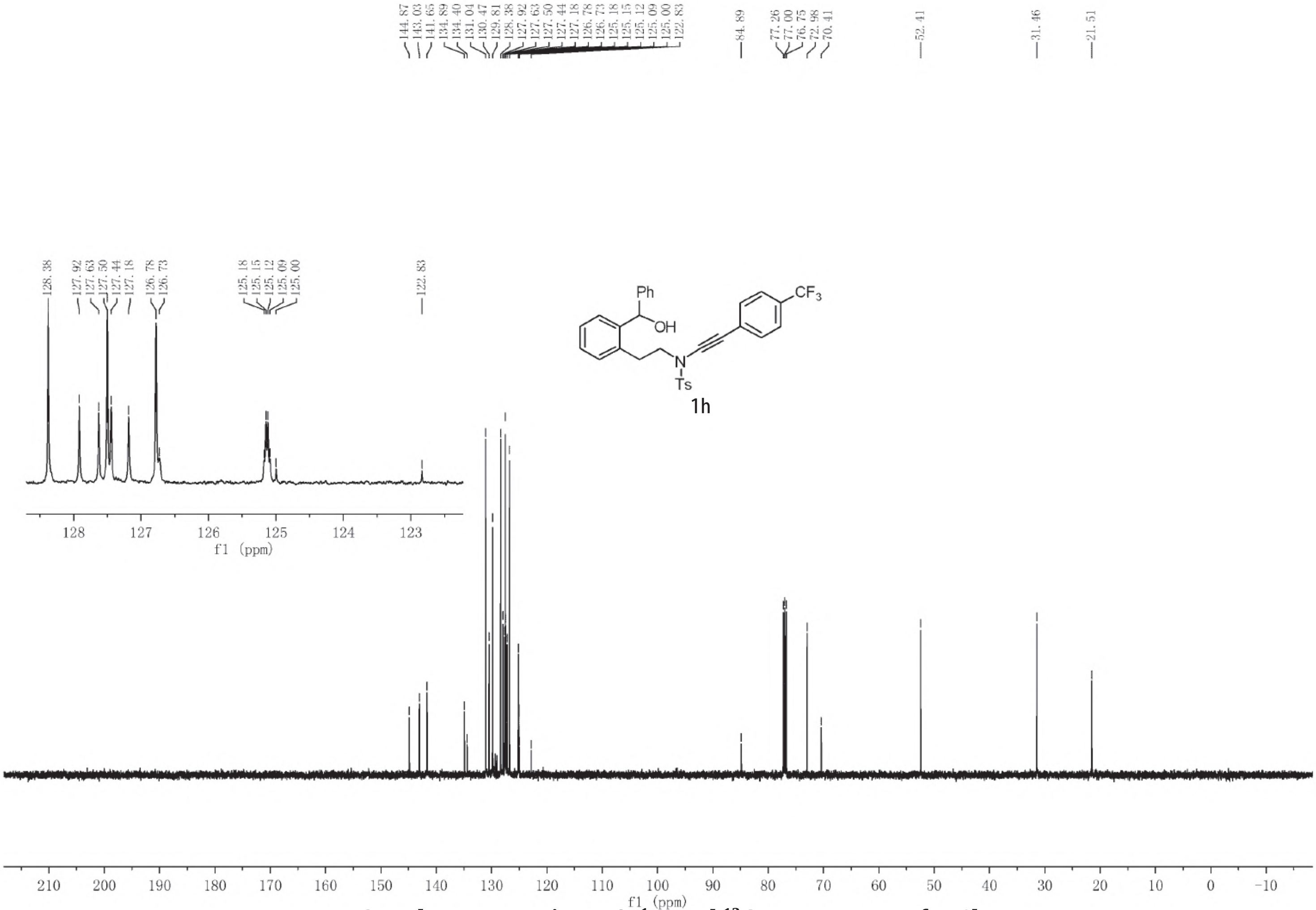

Supplementary Figure 8. ^{1}H and ^{13}C NMR spectra for 1h

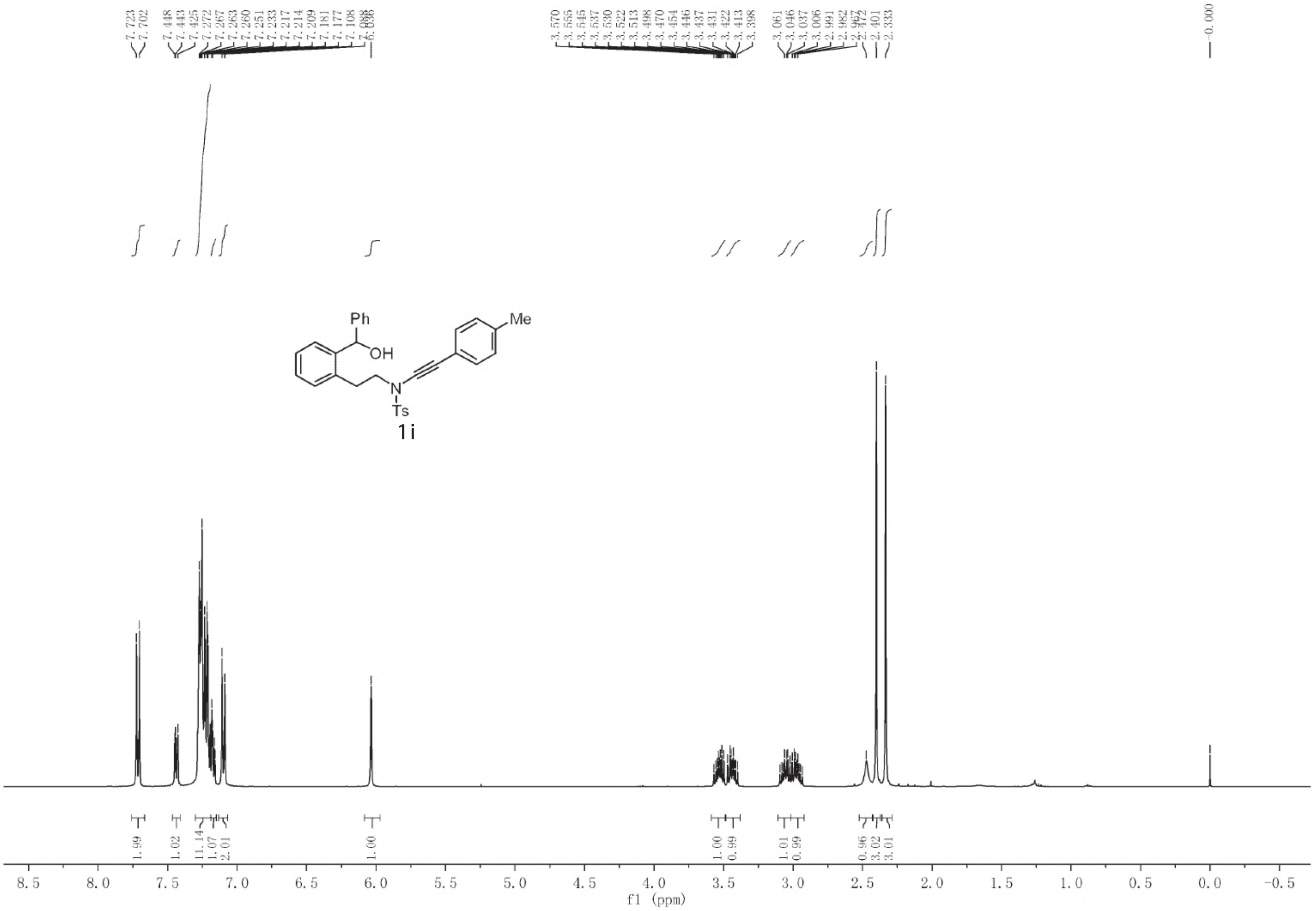

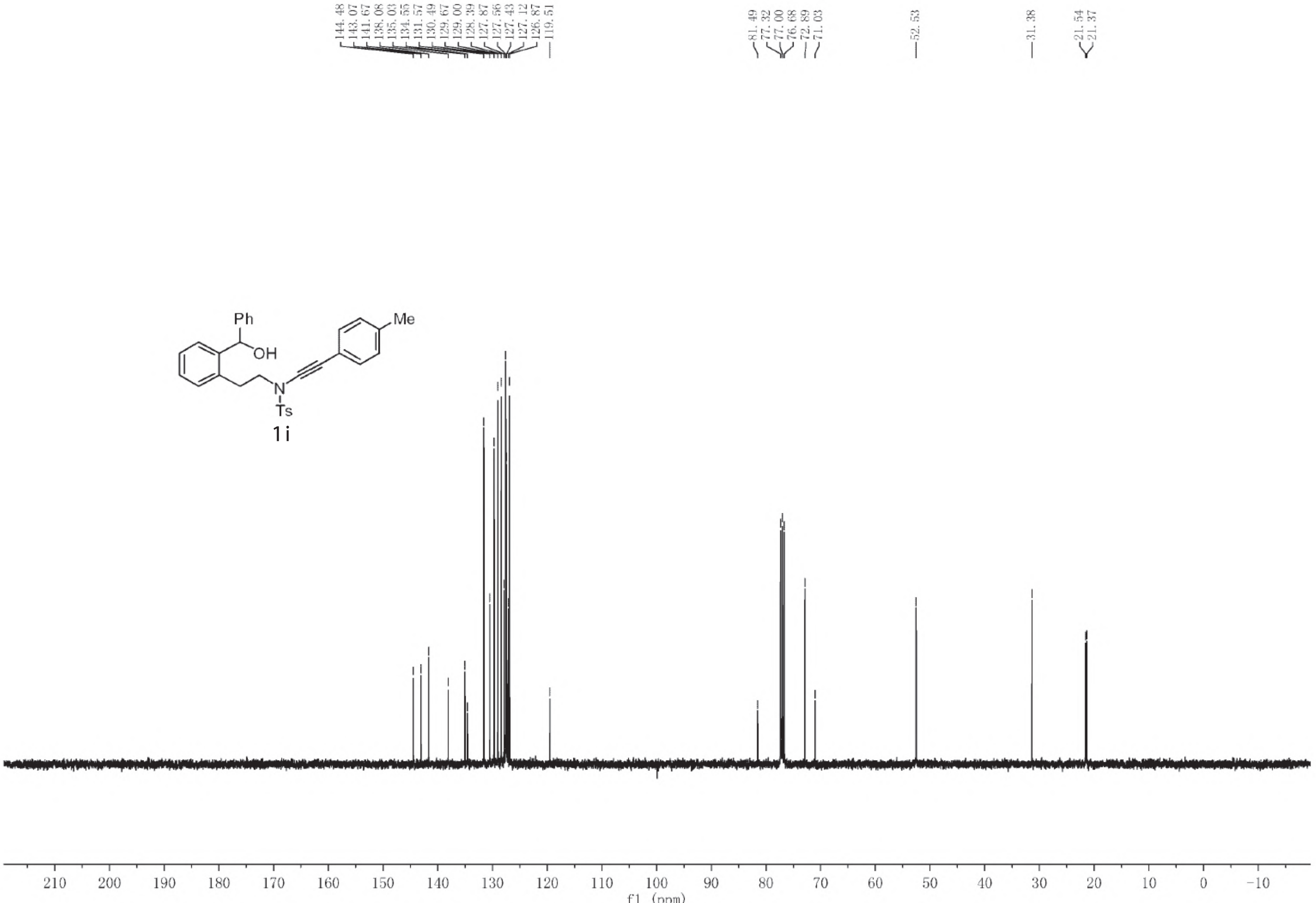

Supplementary Figure 9. ^{1}H and ^{13}C NMR spectra for 1i

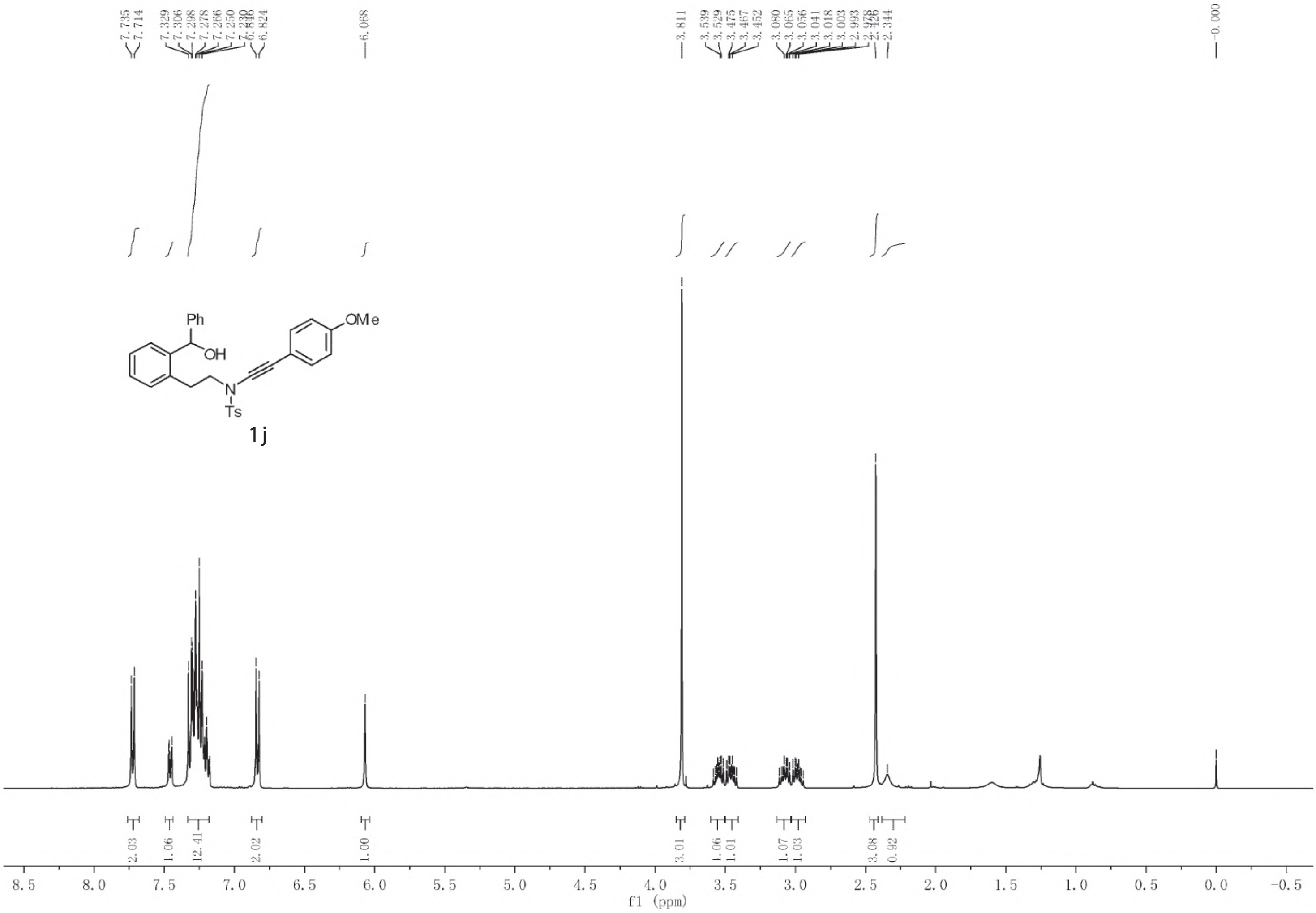

1j

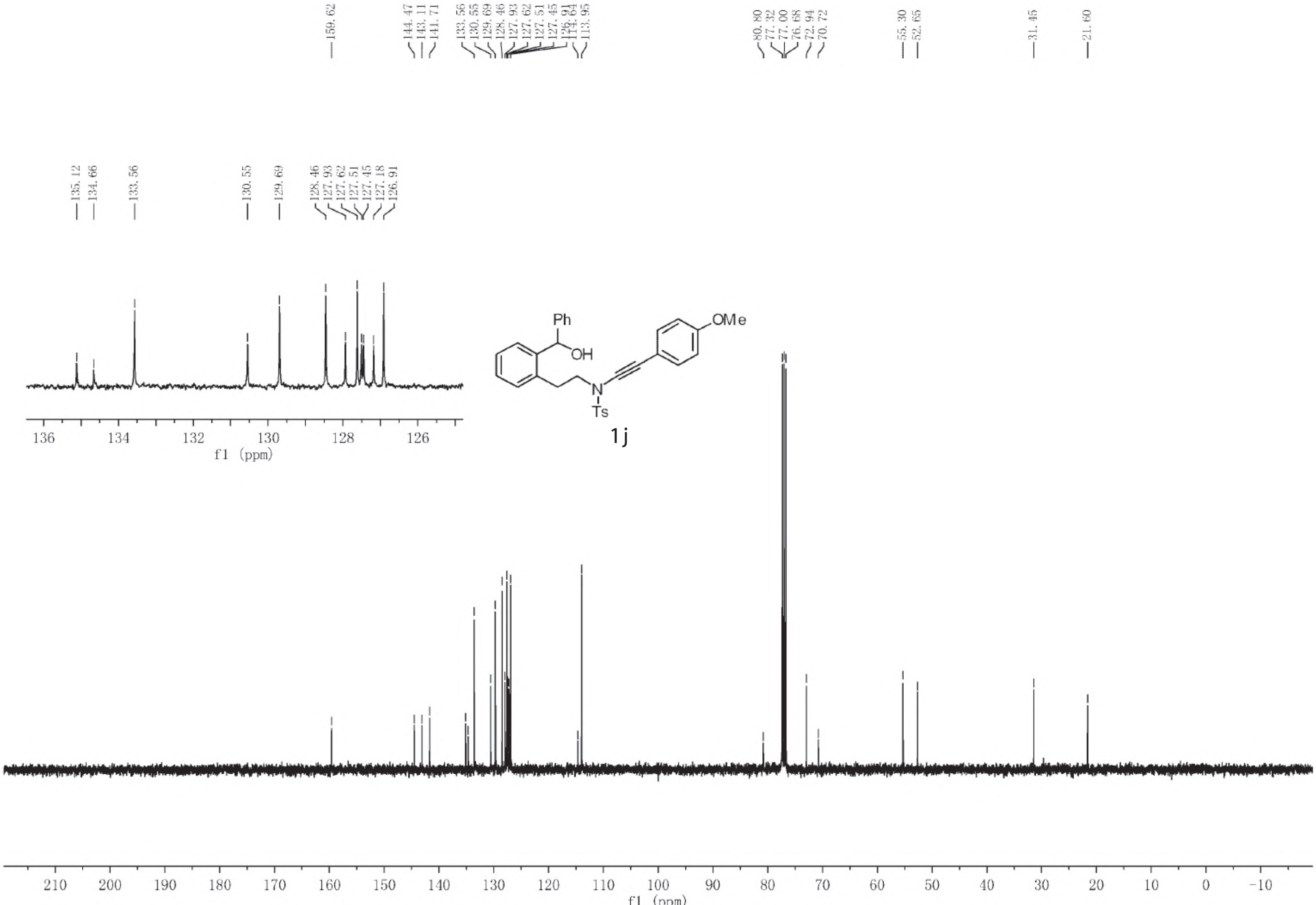

1j

Supplementary Figure 10. ^{1}H and ^{13}C NMR spectra for 1j

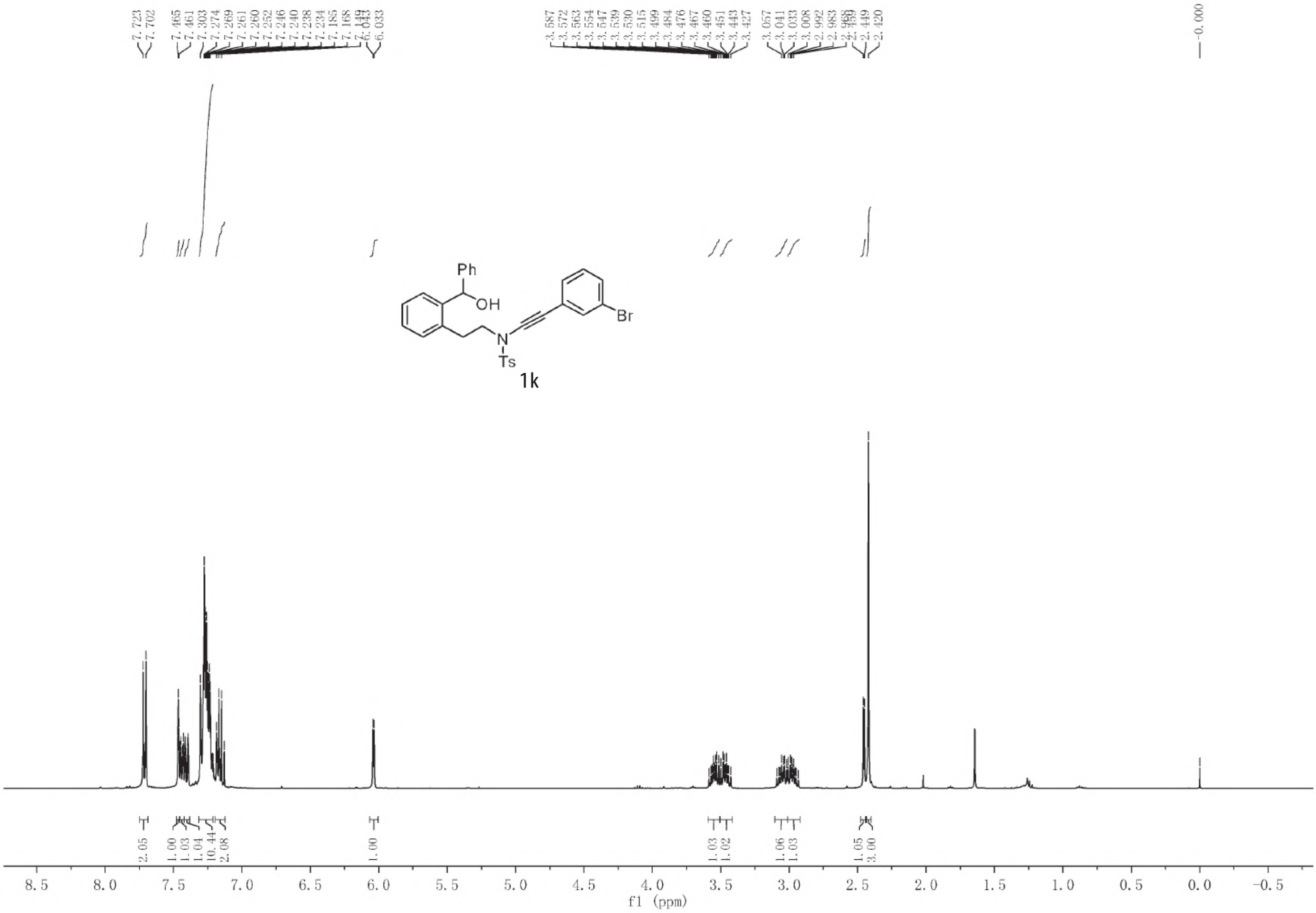

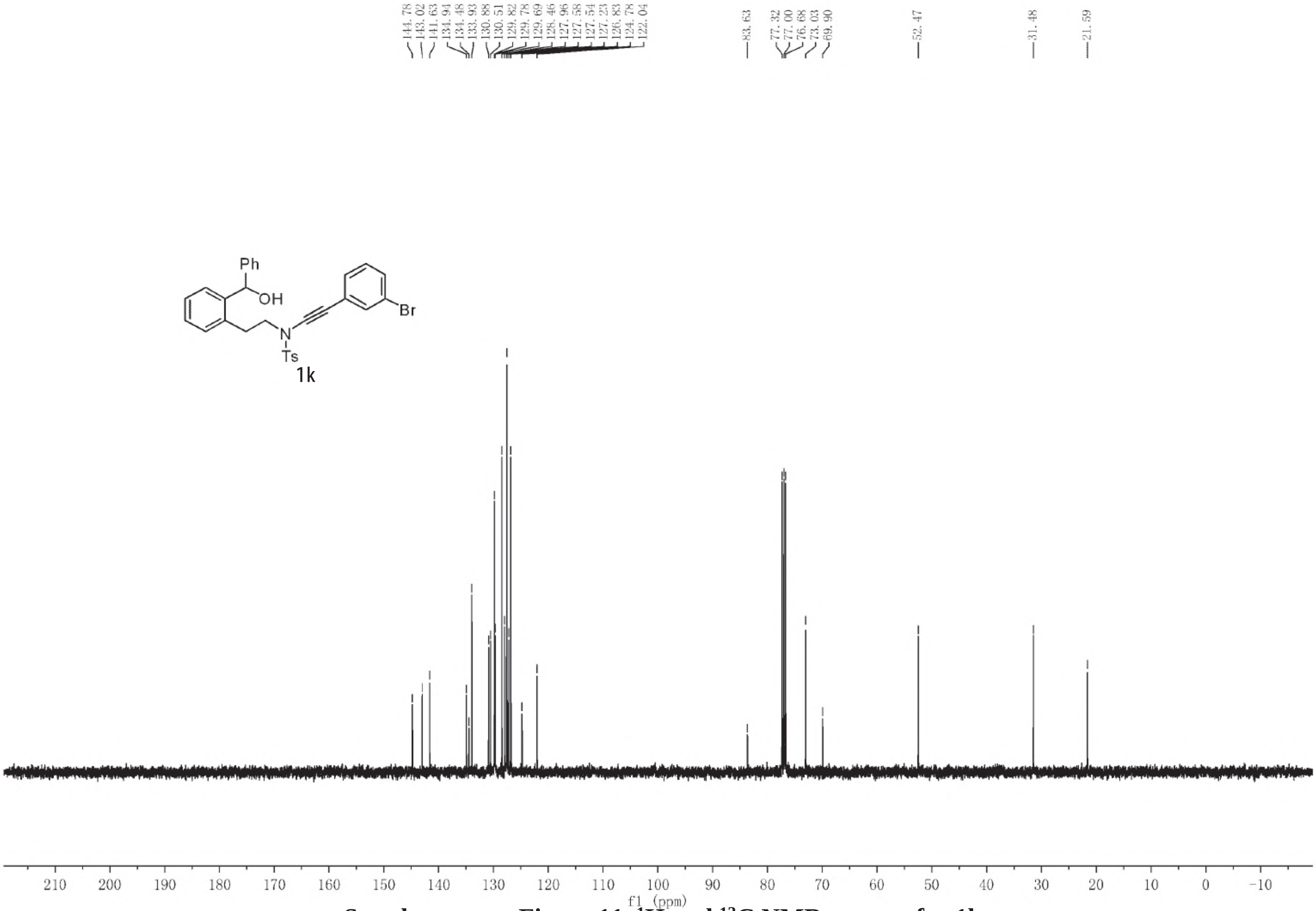

Supplementary Figure 11. ^{1}H and ^{13}C NMR spectra for 1k

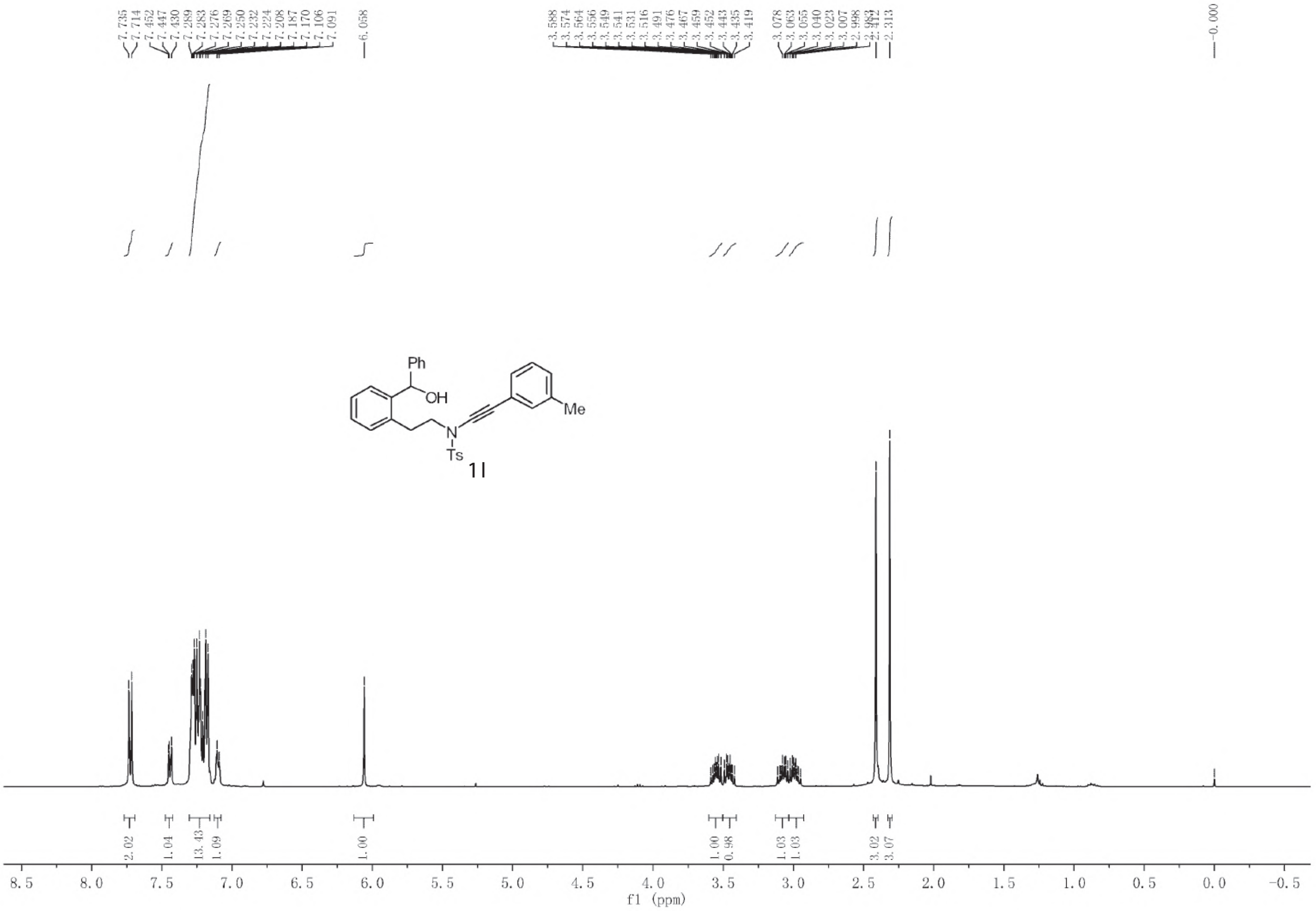

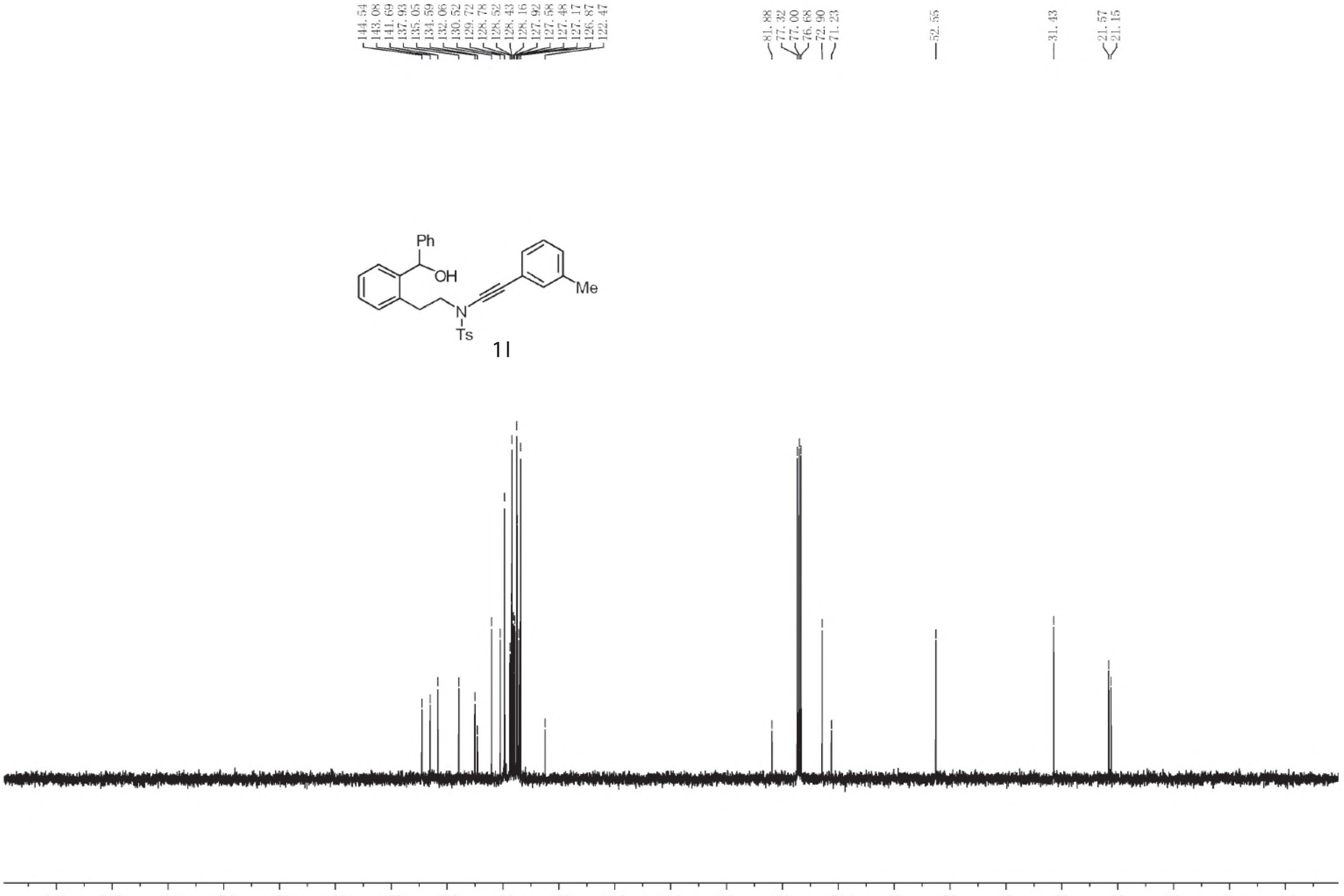

Supplementary Figure 12. ^{1}H and ^{13}C NMR spectra for 1l

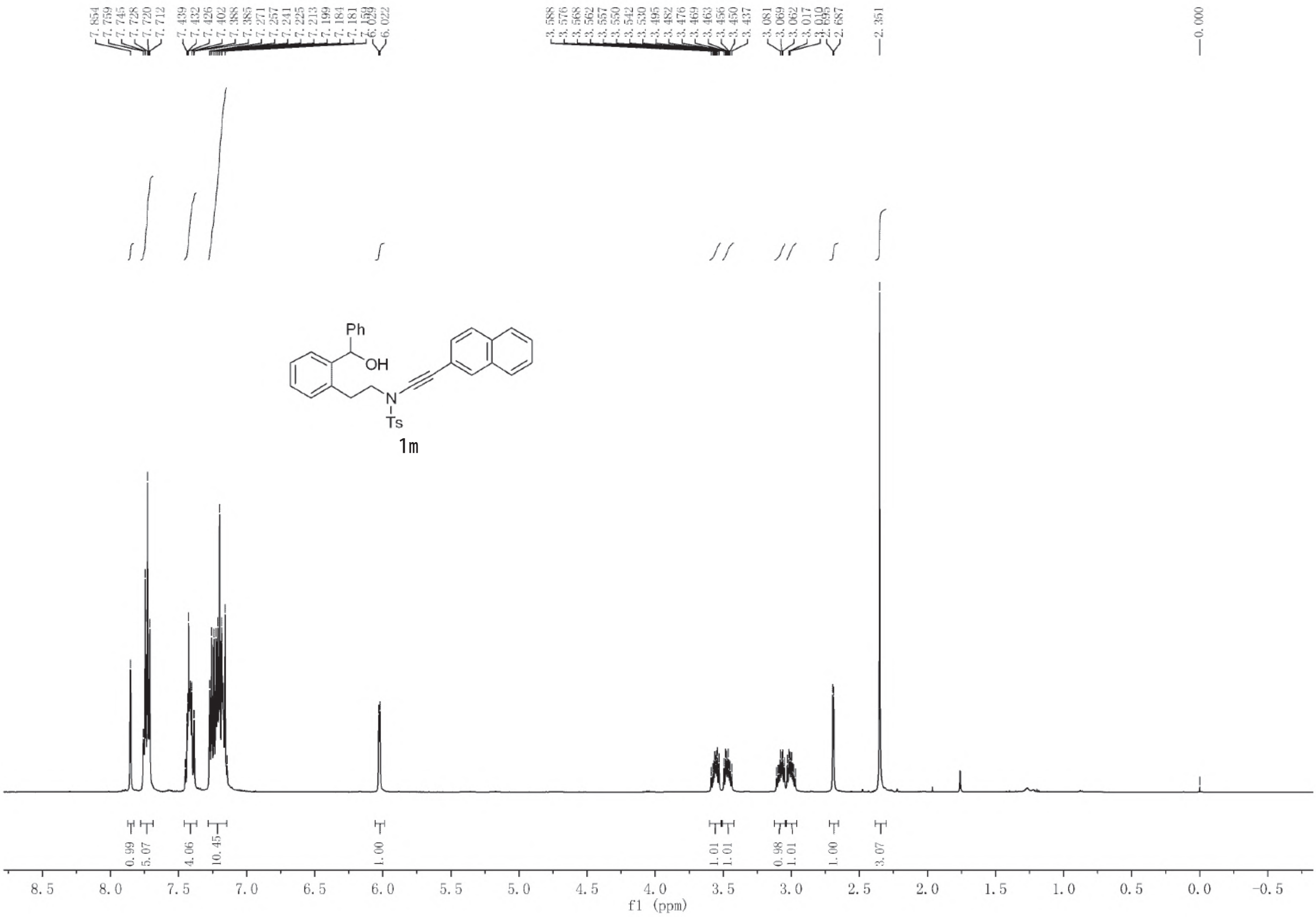

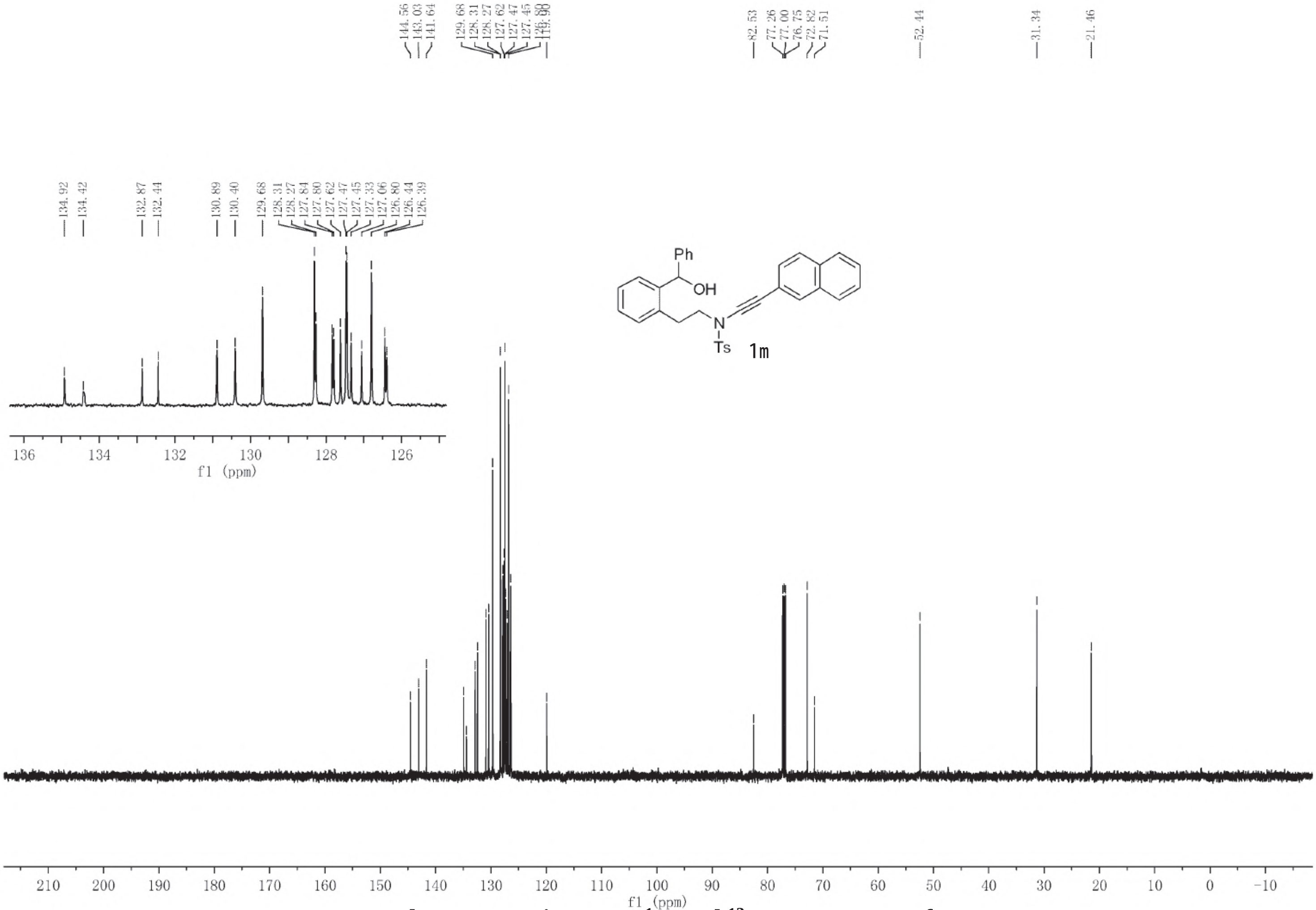

Supplementary Figure 13. ^{1}H and ^{13}C NMR spectra for 1m

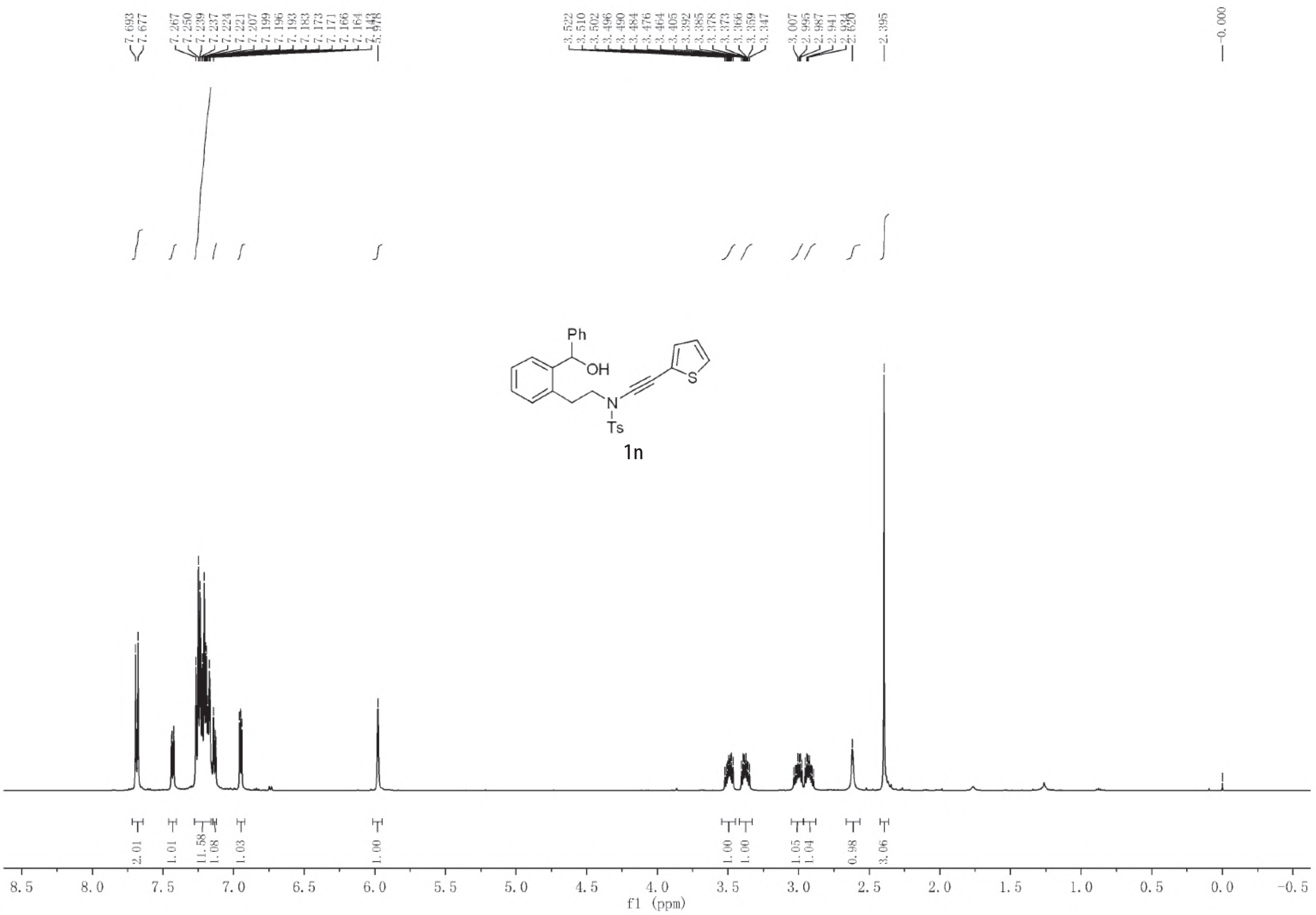

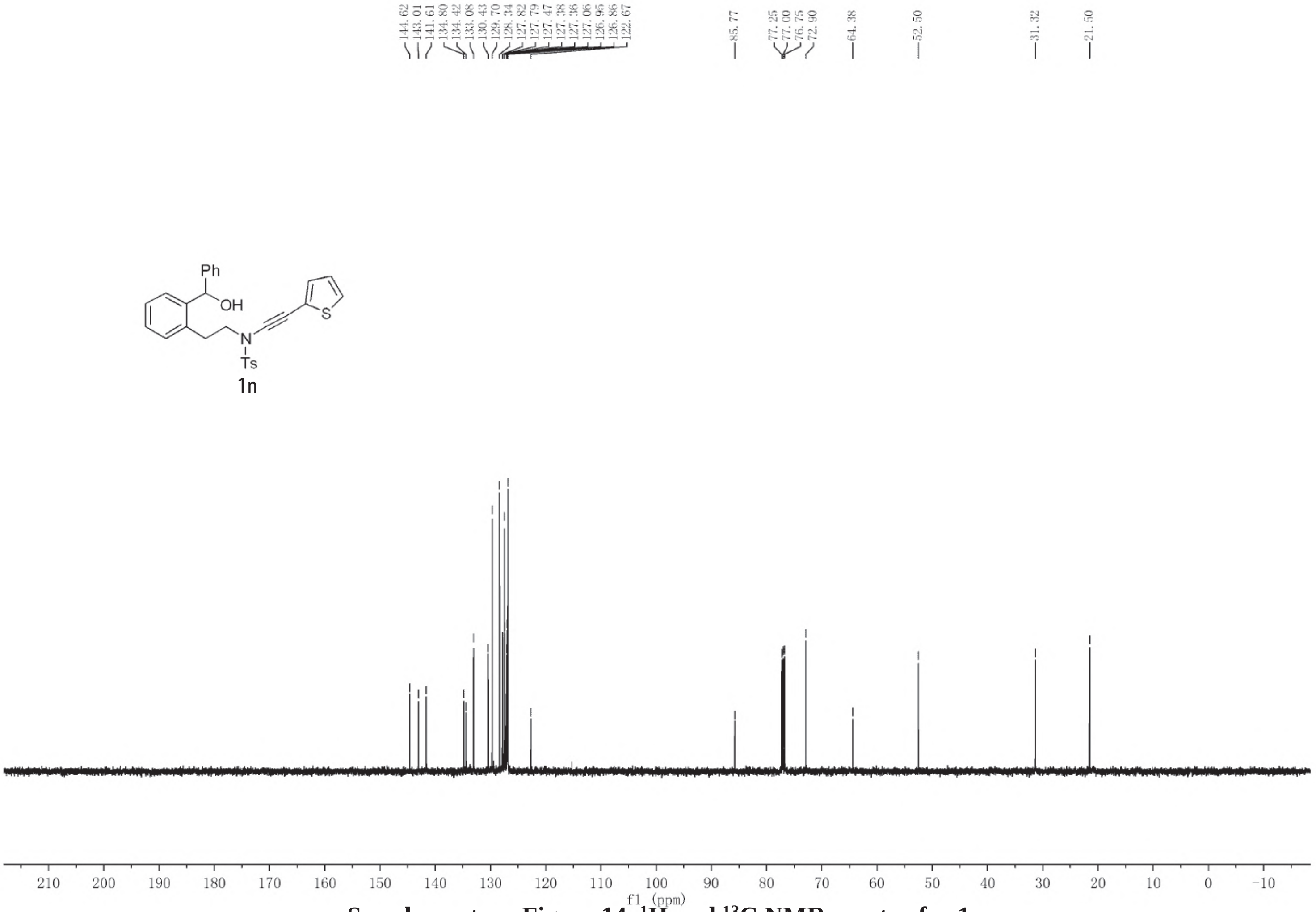

Supplementary Figure 14. ^{1}H and ^{13}C NMR spectra for 1n

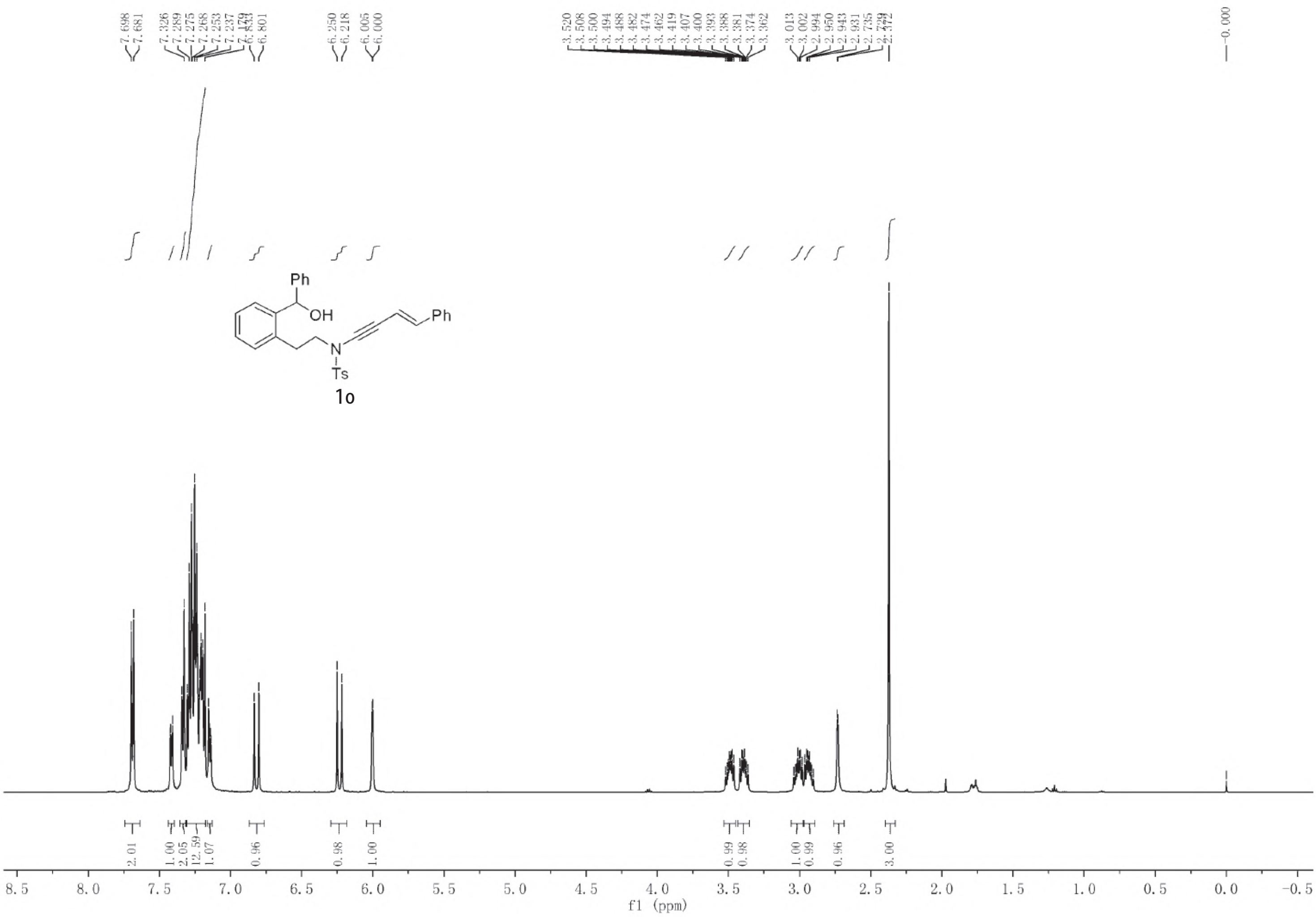

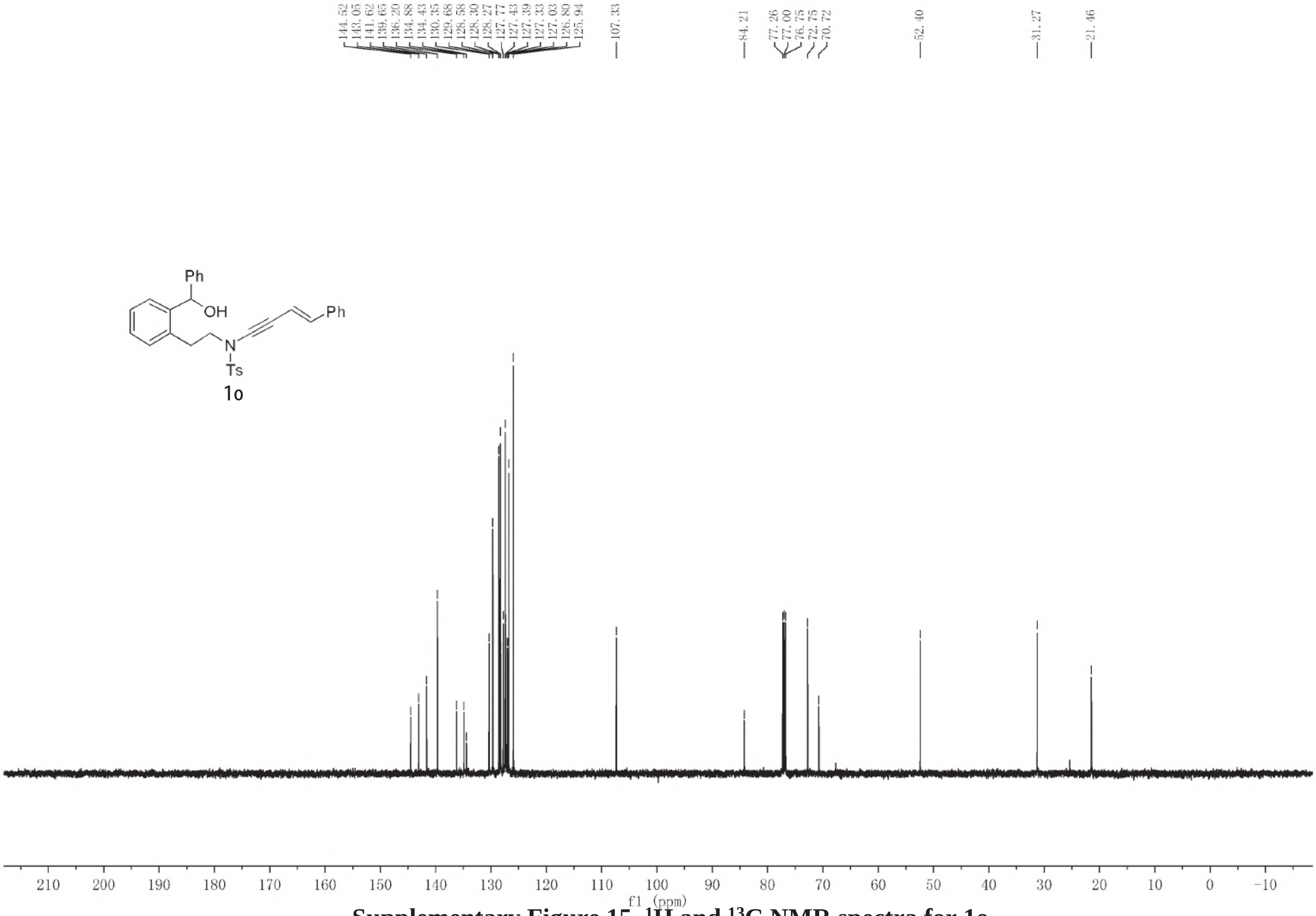

Supplementary Figure 15. ^{1}H and ^{13}C NMR spectra for 1o

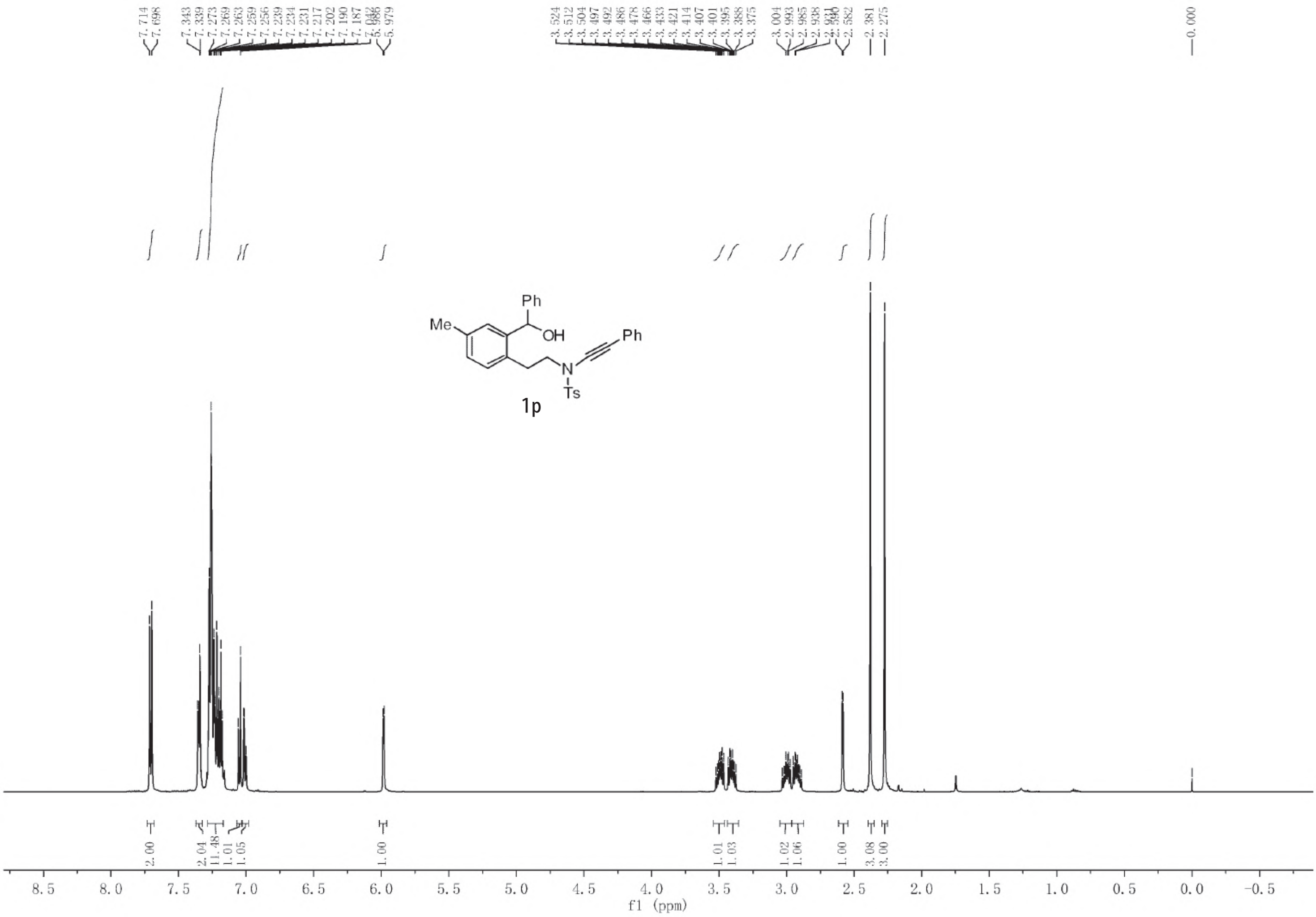

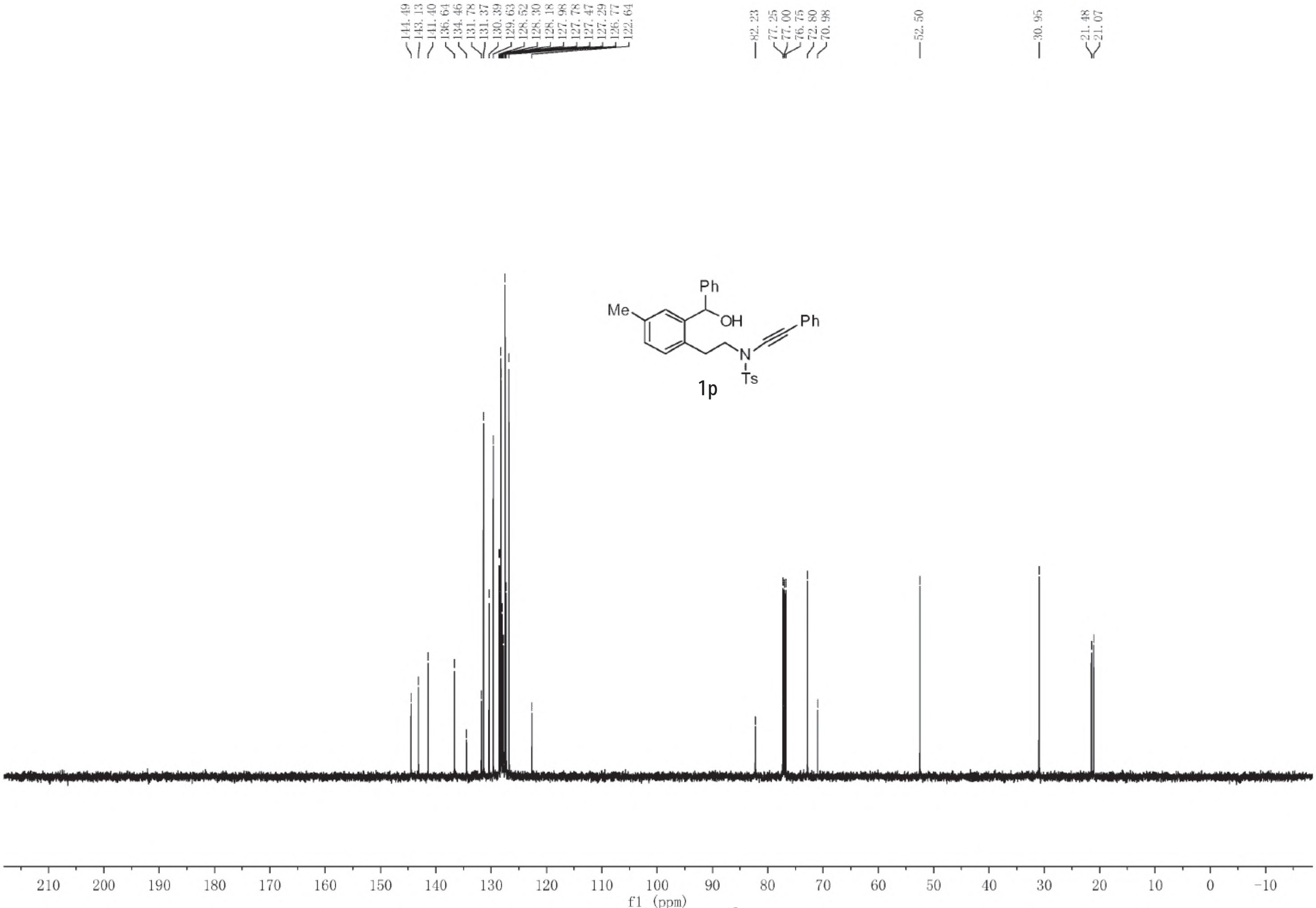

Supplementary Figure 16. ^{1}H and ^{13}C NMR spectra for 1p

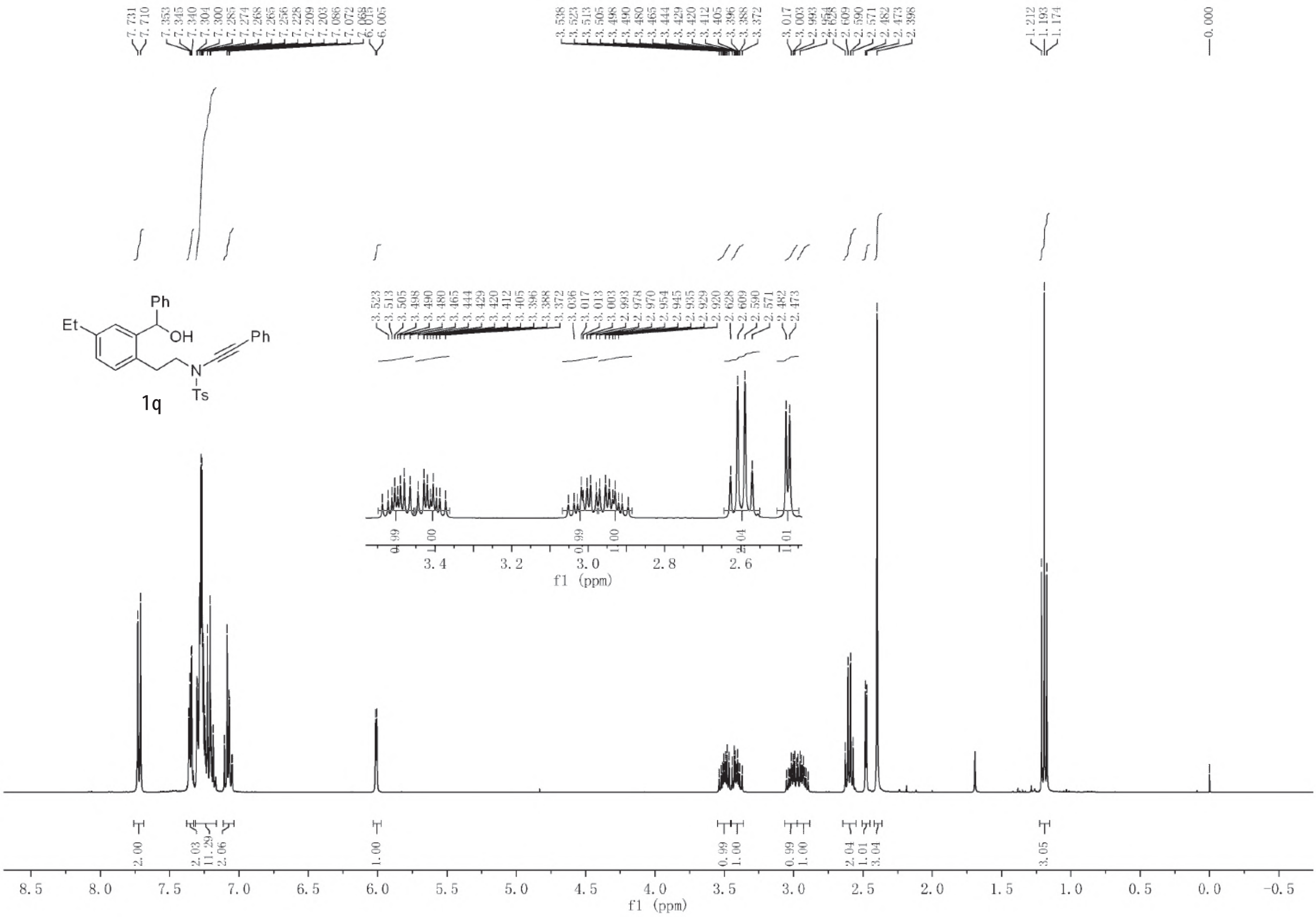

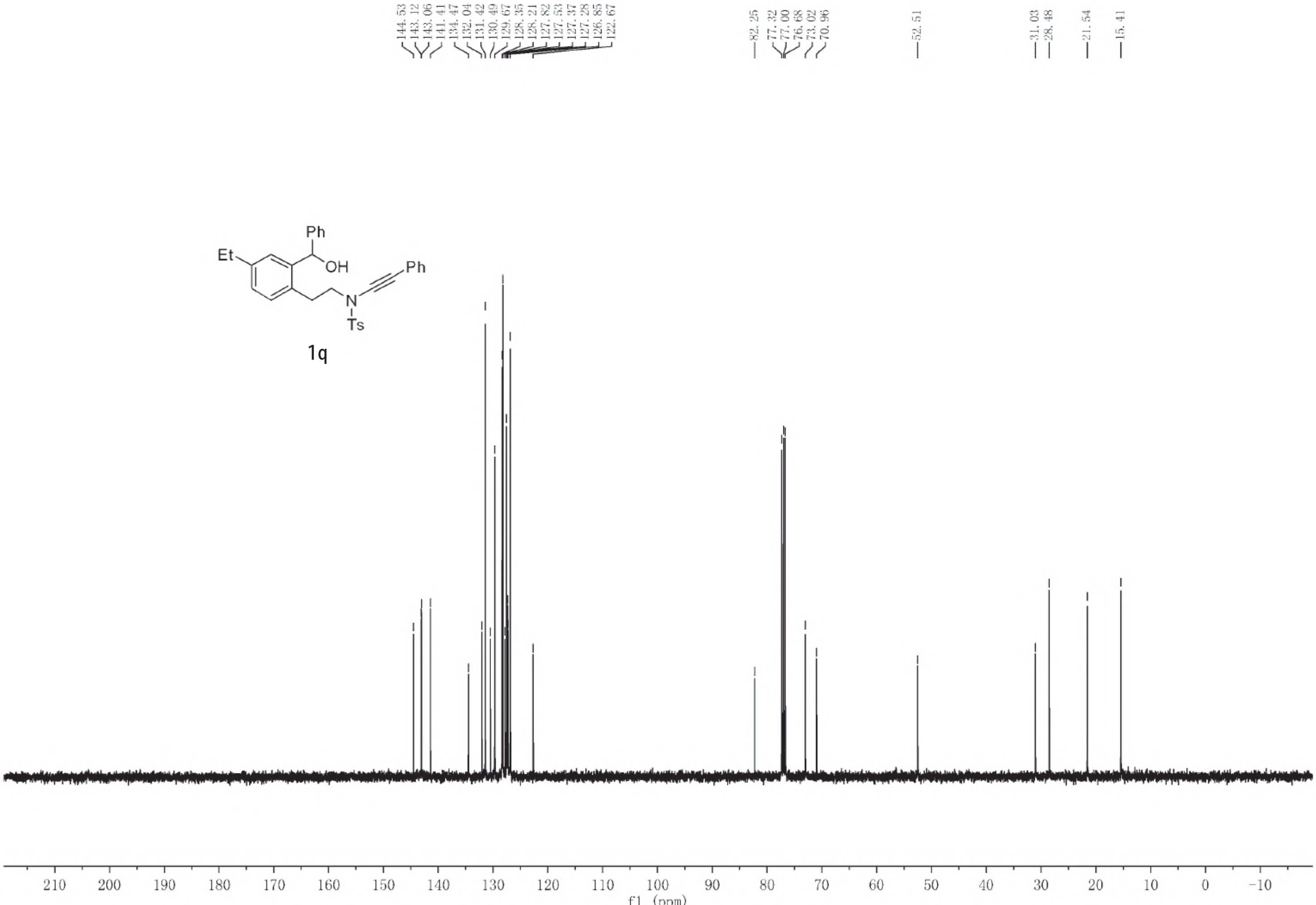

Supplementary Figure 17. ^{1}H **and** ^{13}C **NMR spectra for 1q**

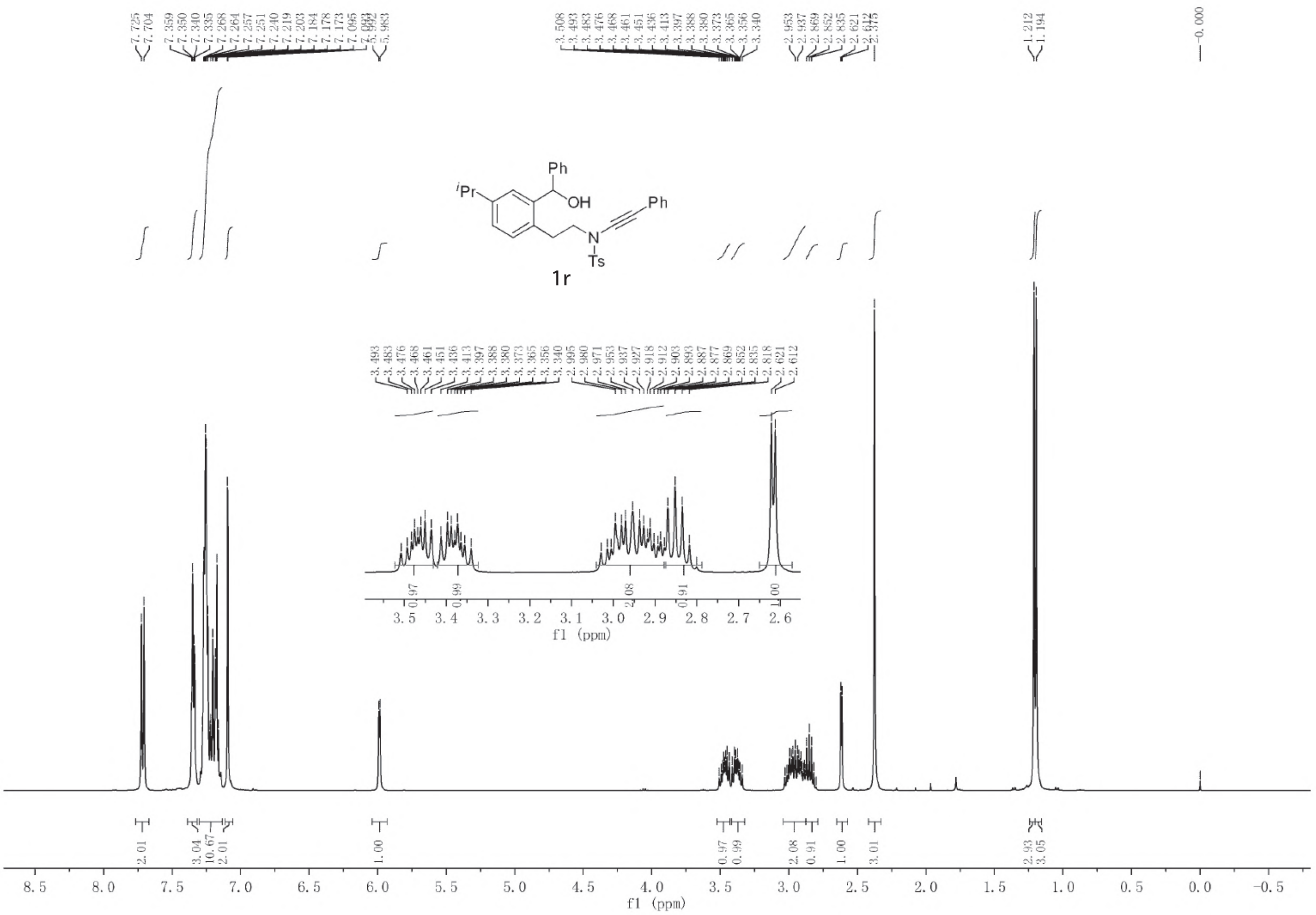

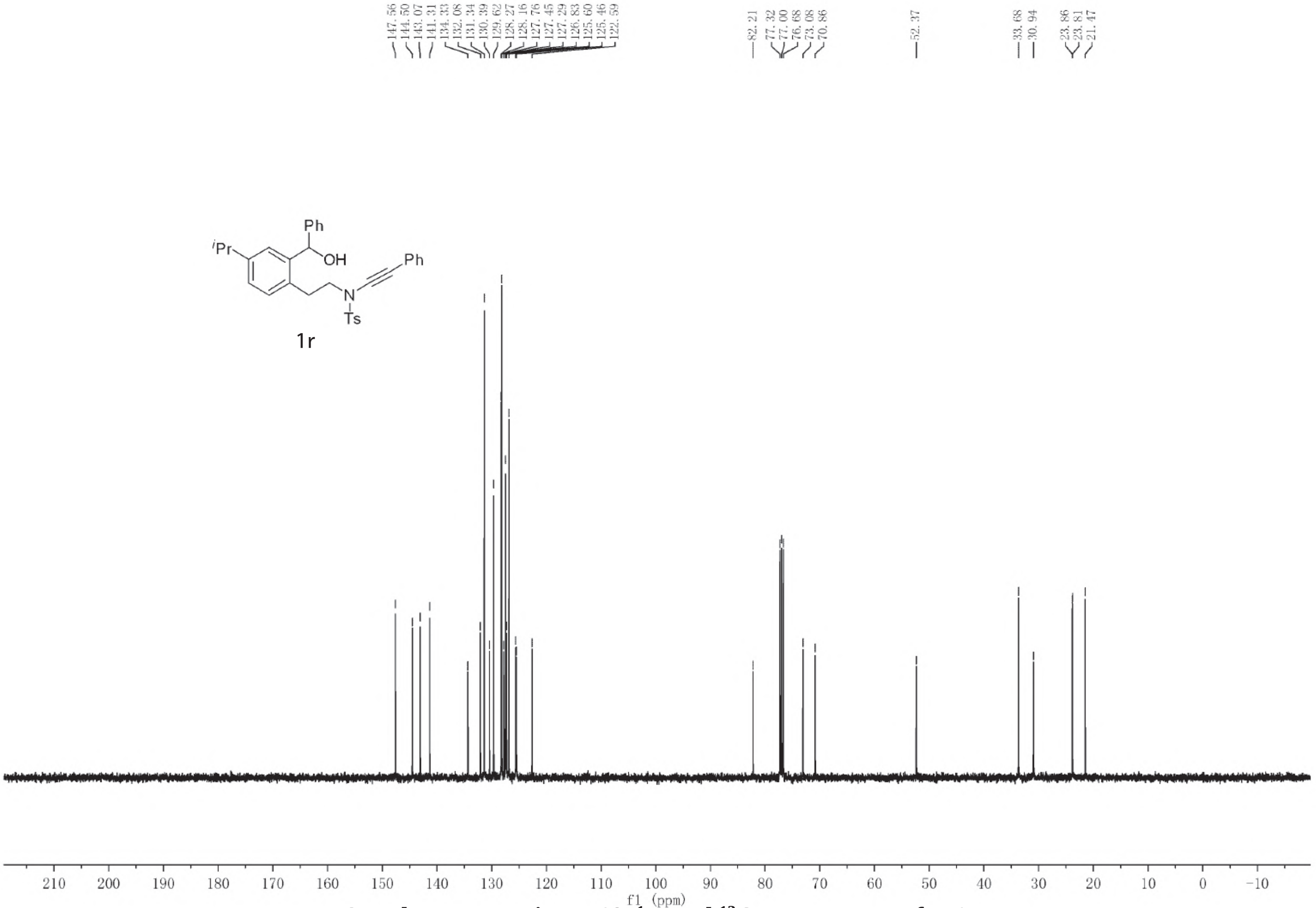

Supplementary Figure 18. ^{1}H and ^{13}C NMR spectra for 1r

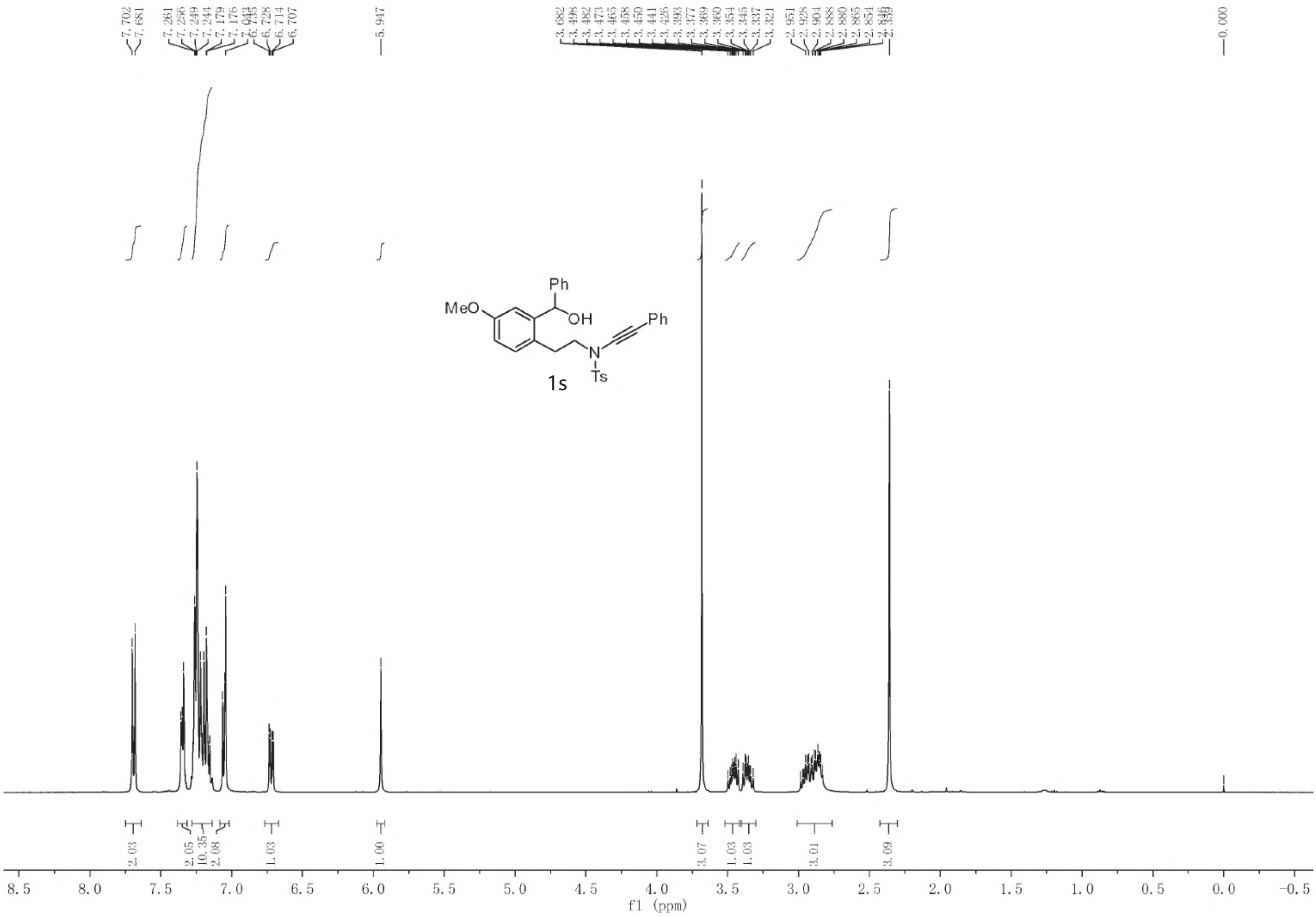

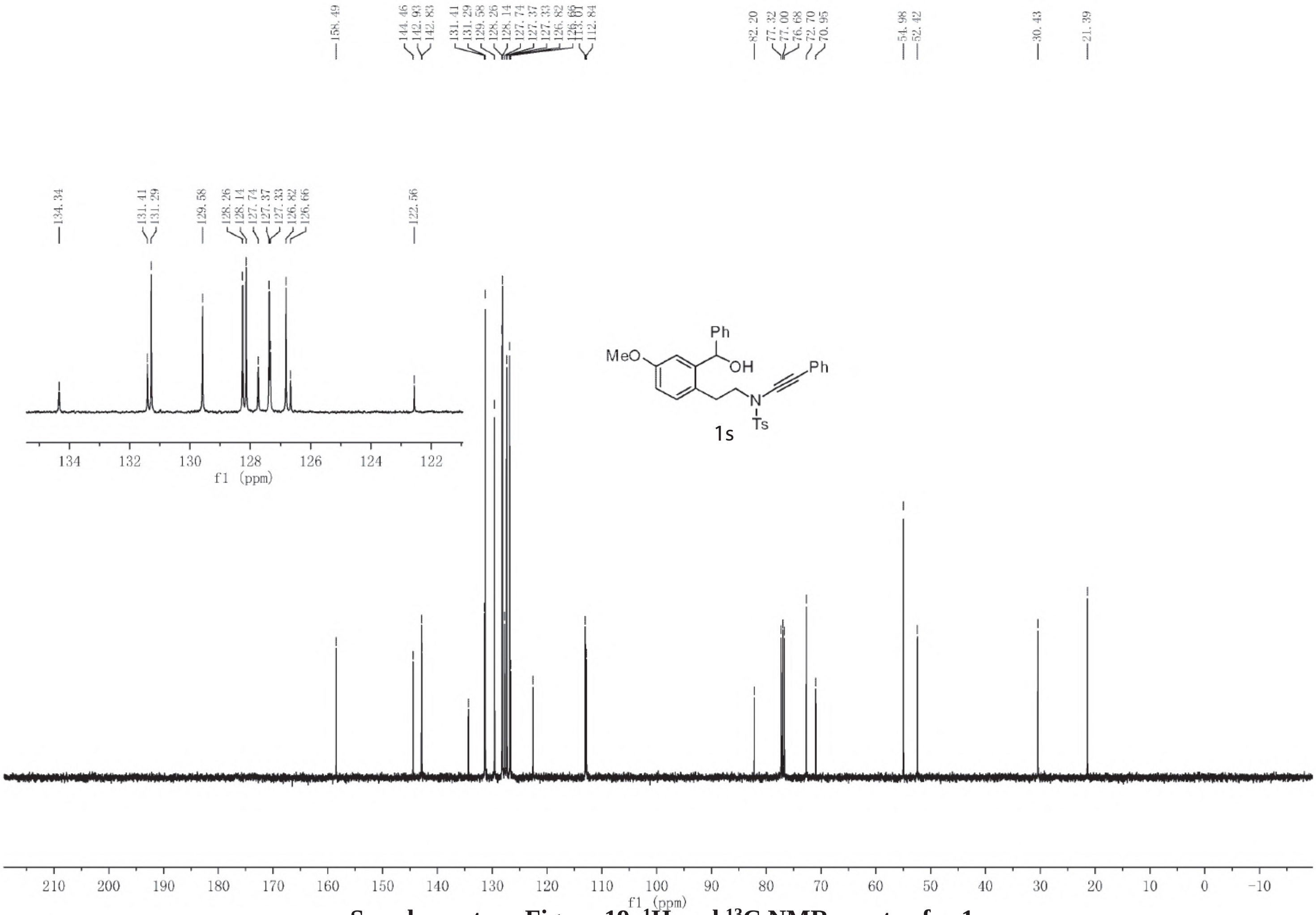

Supplementary Figure 19. ^{1}H and ^{13}C NMR spectra for 1s

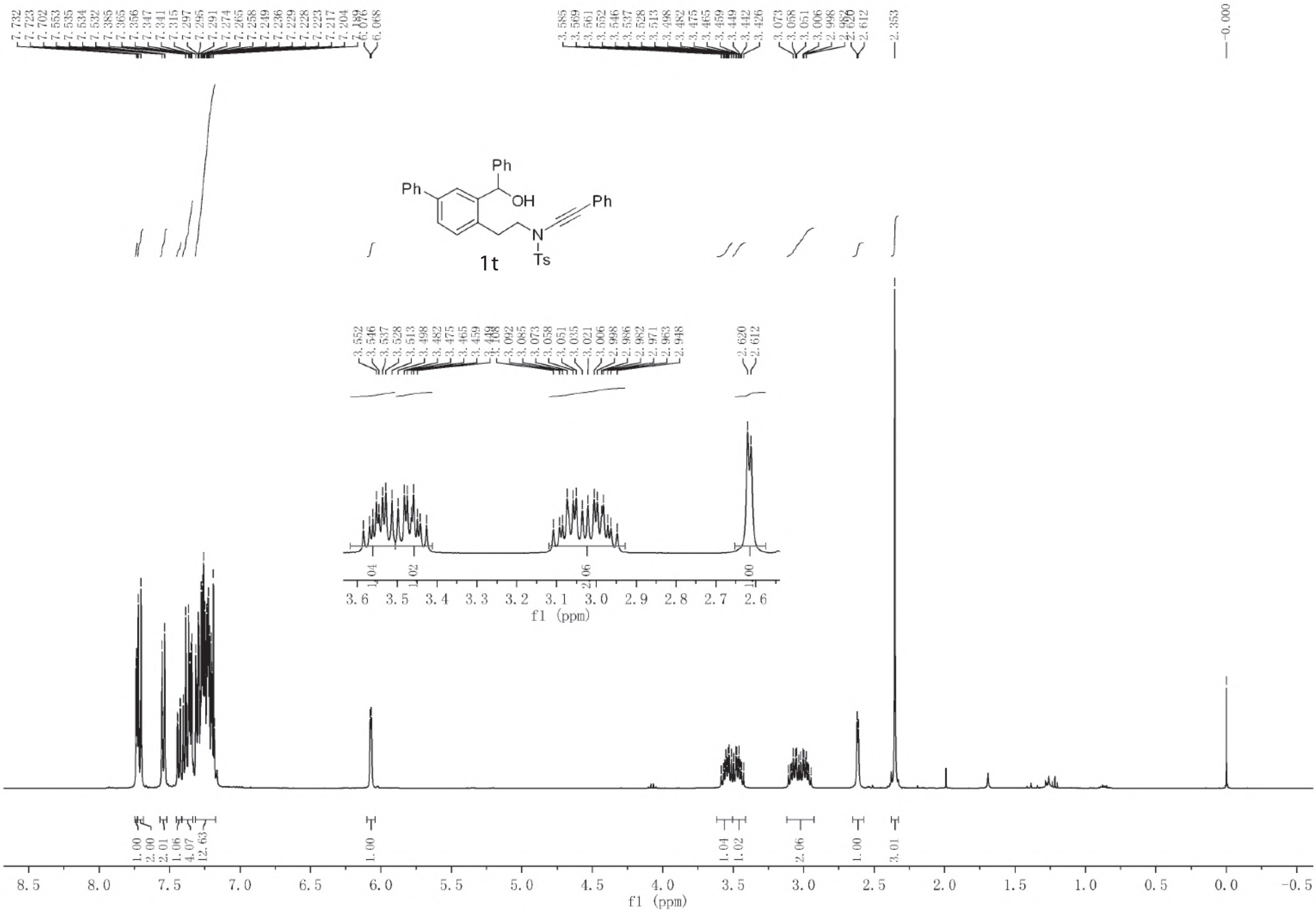

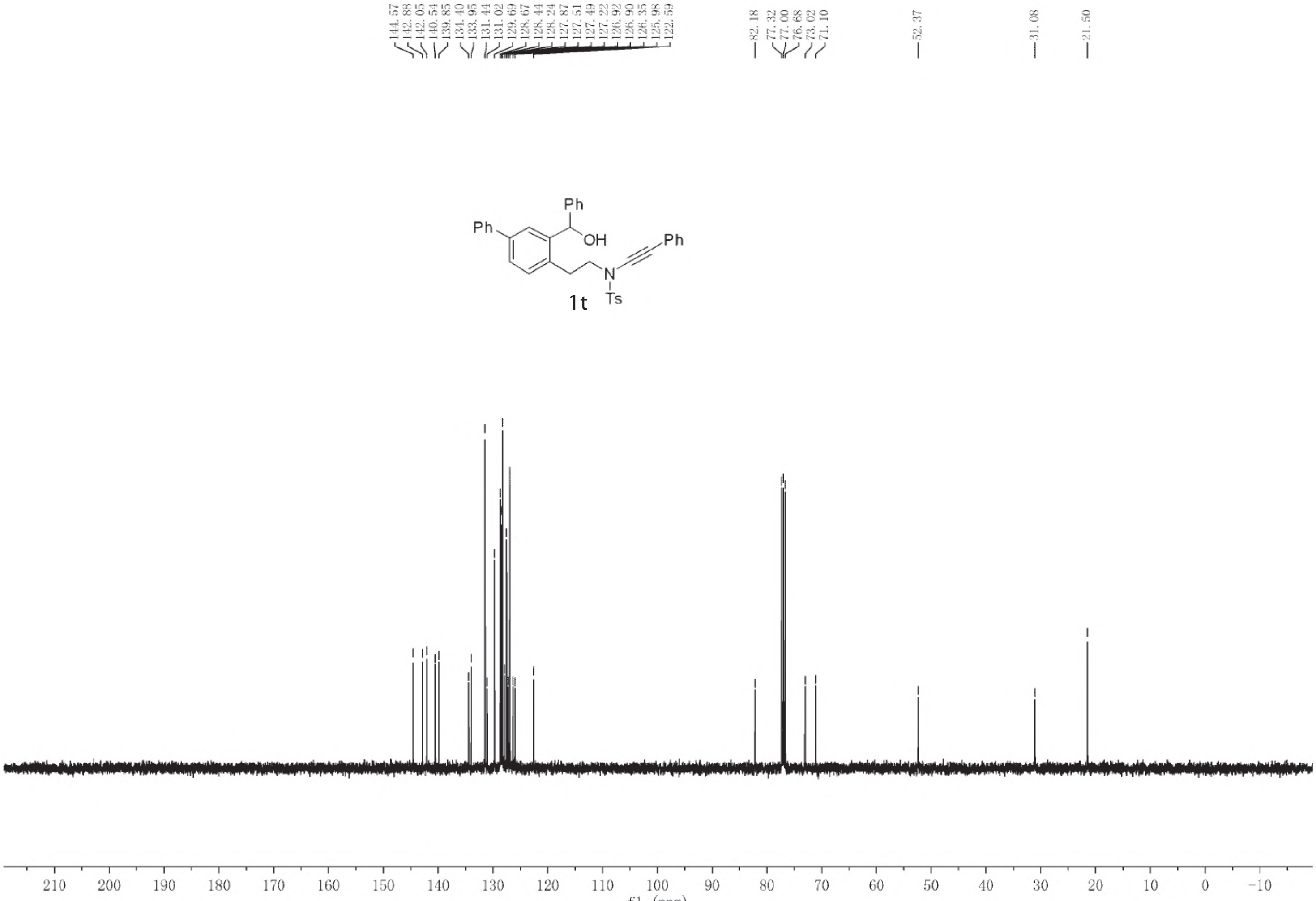

Supplementary Figure 20. ^{1}H and ^{13}C NMR spectra for 1t

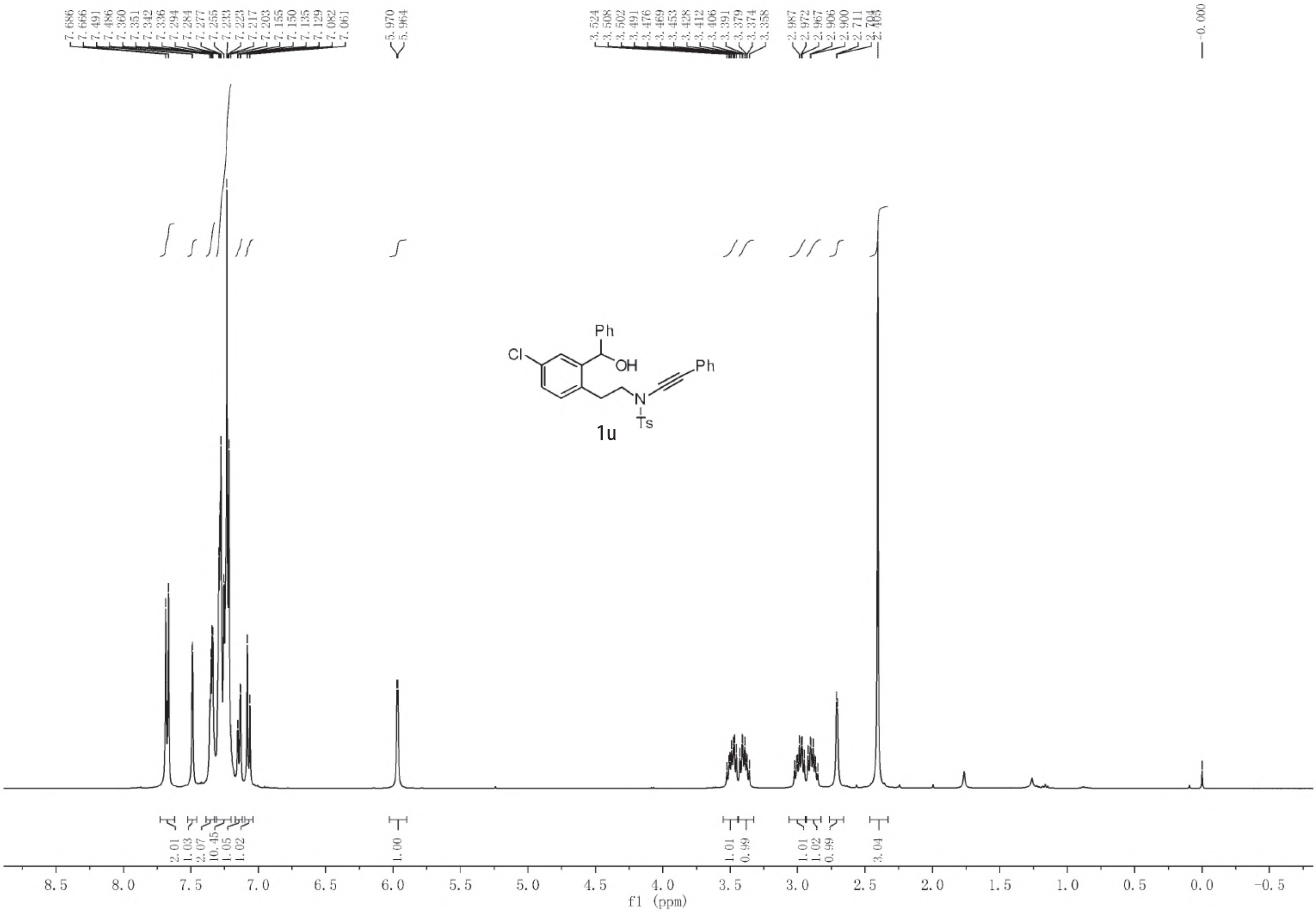

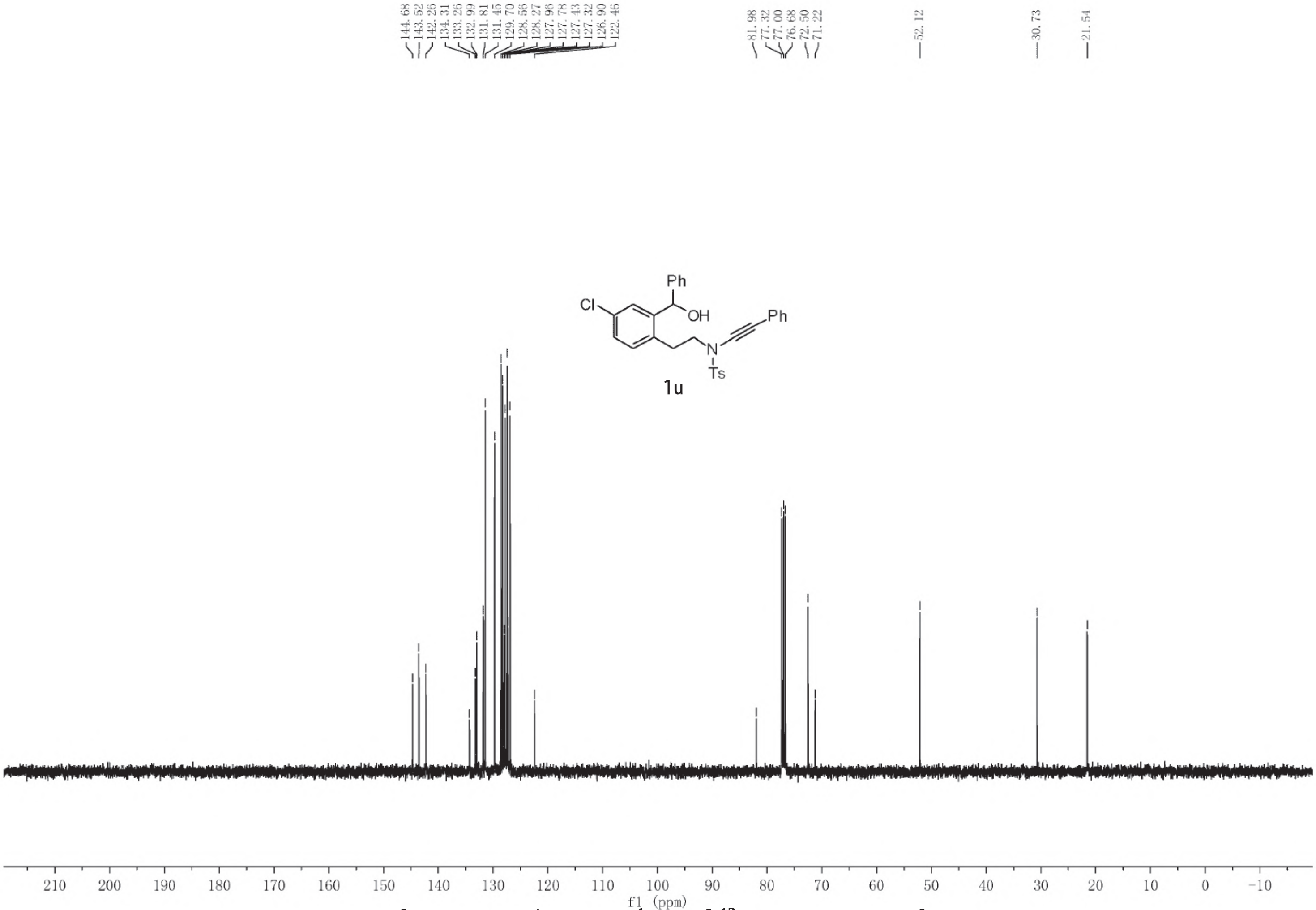

Supplementary Figure 21. ^{1}H and ^{13}C NMR spectra for 1u

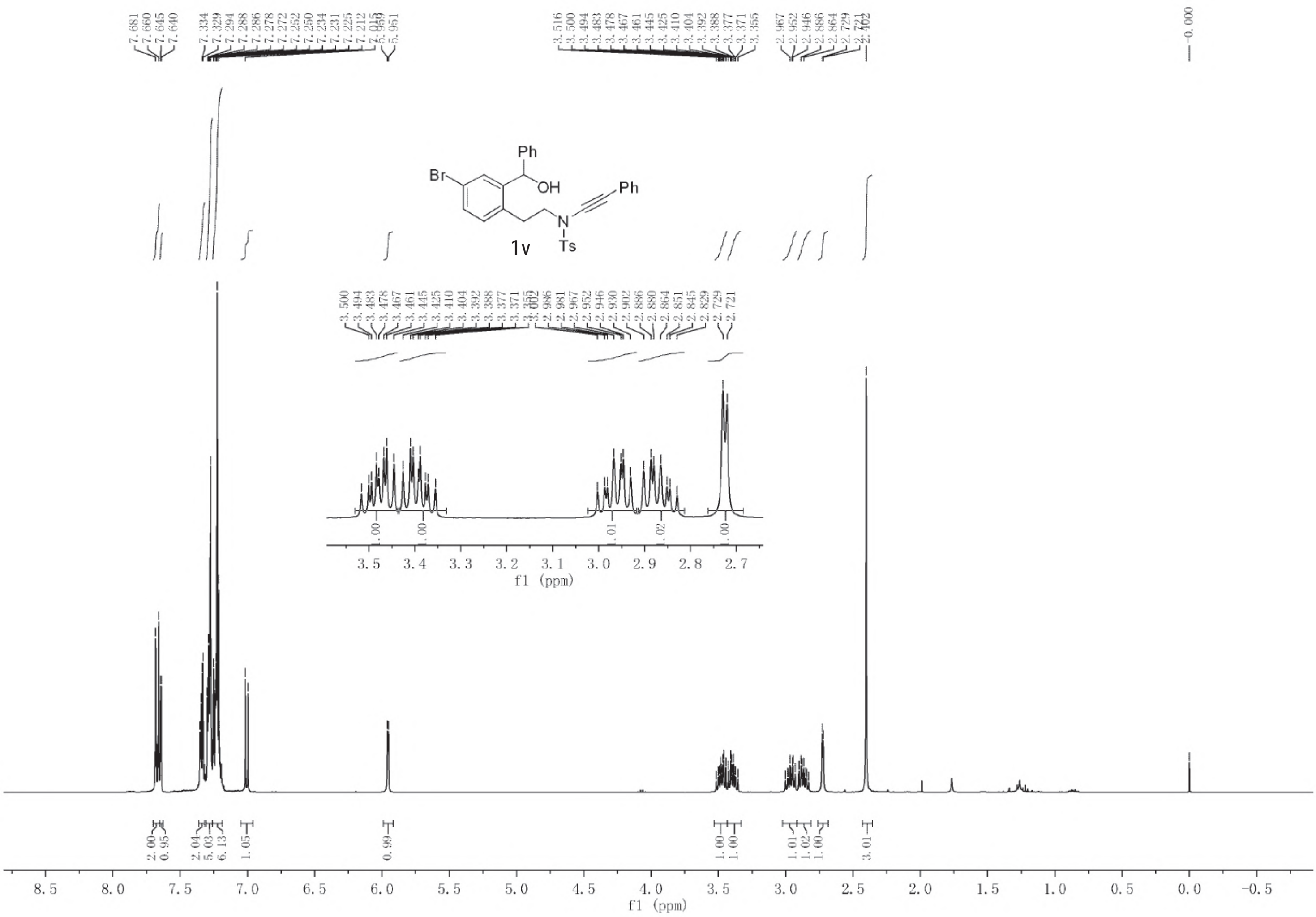

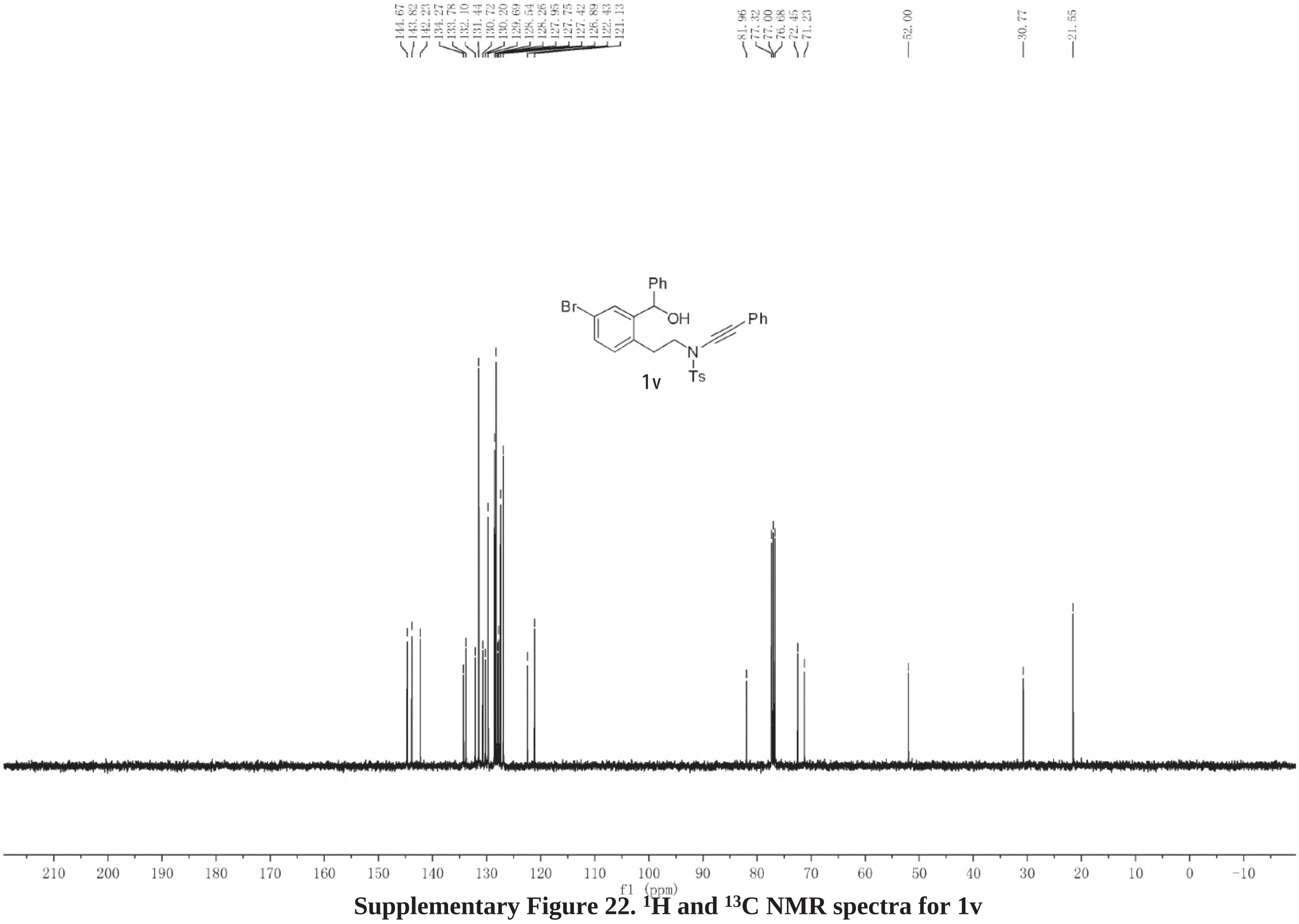

Supplementary Figure 22. ^{1}H and ^{13}C NMR spectra for 1v

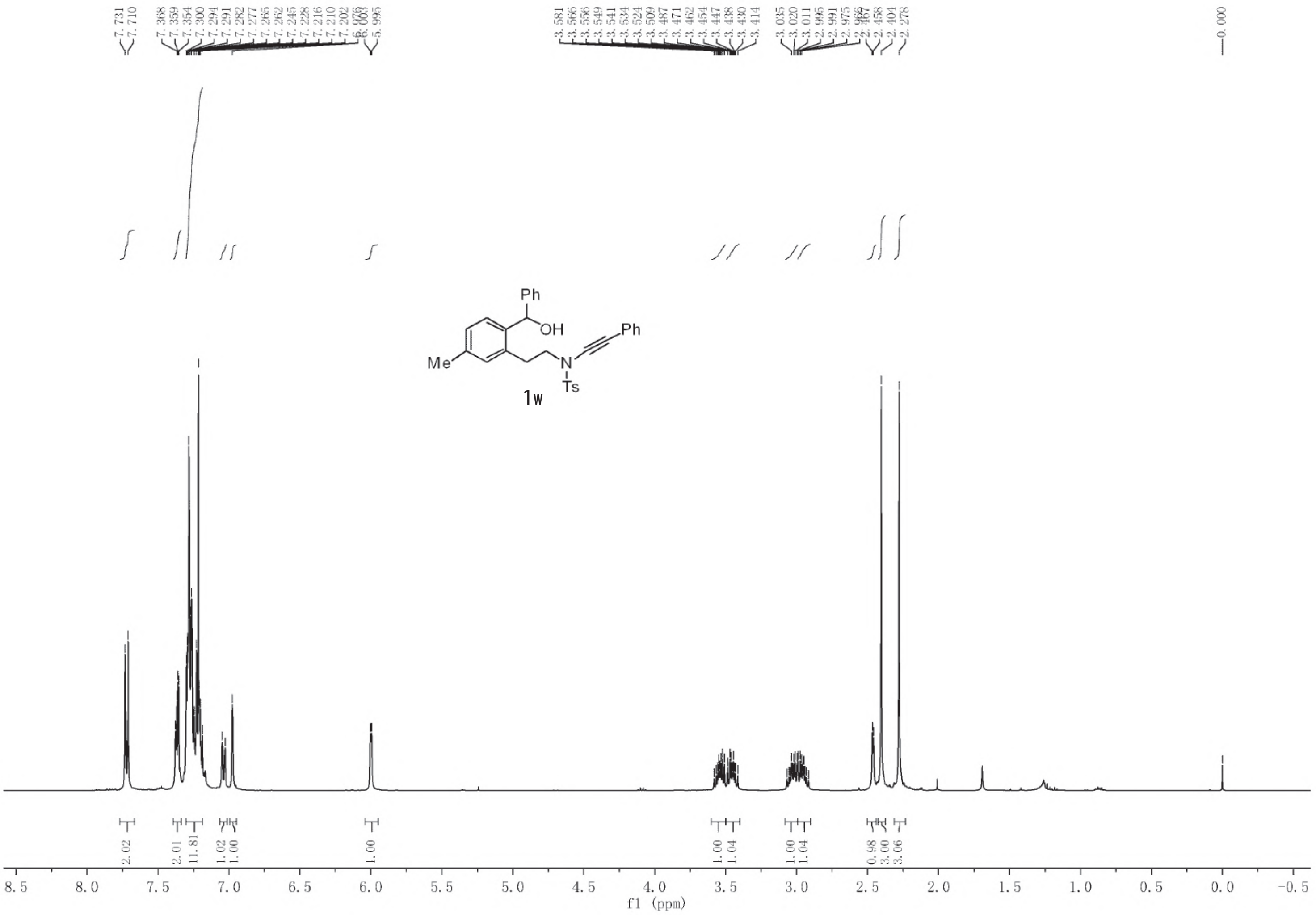

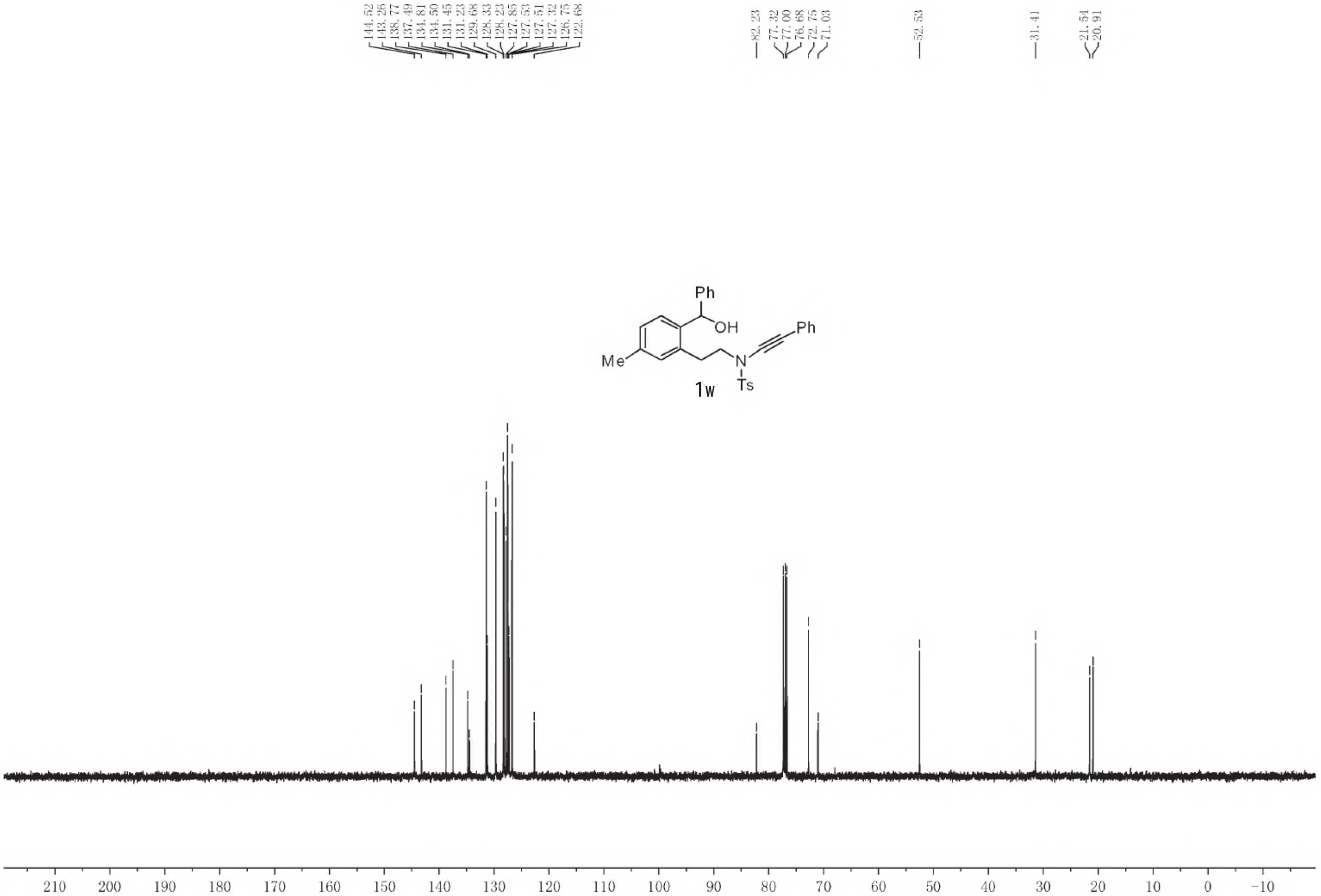

Supplementary Figure 23. ^{1}H and ^{13}C NMR spectra for 1w

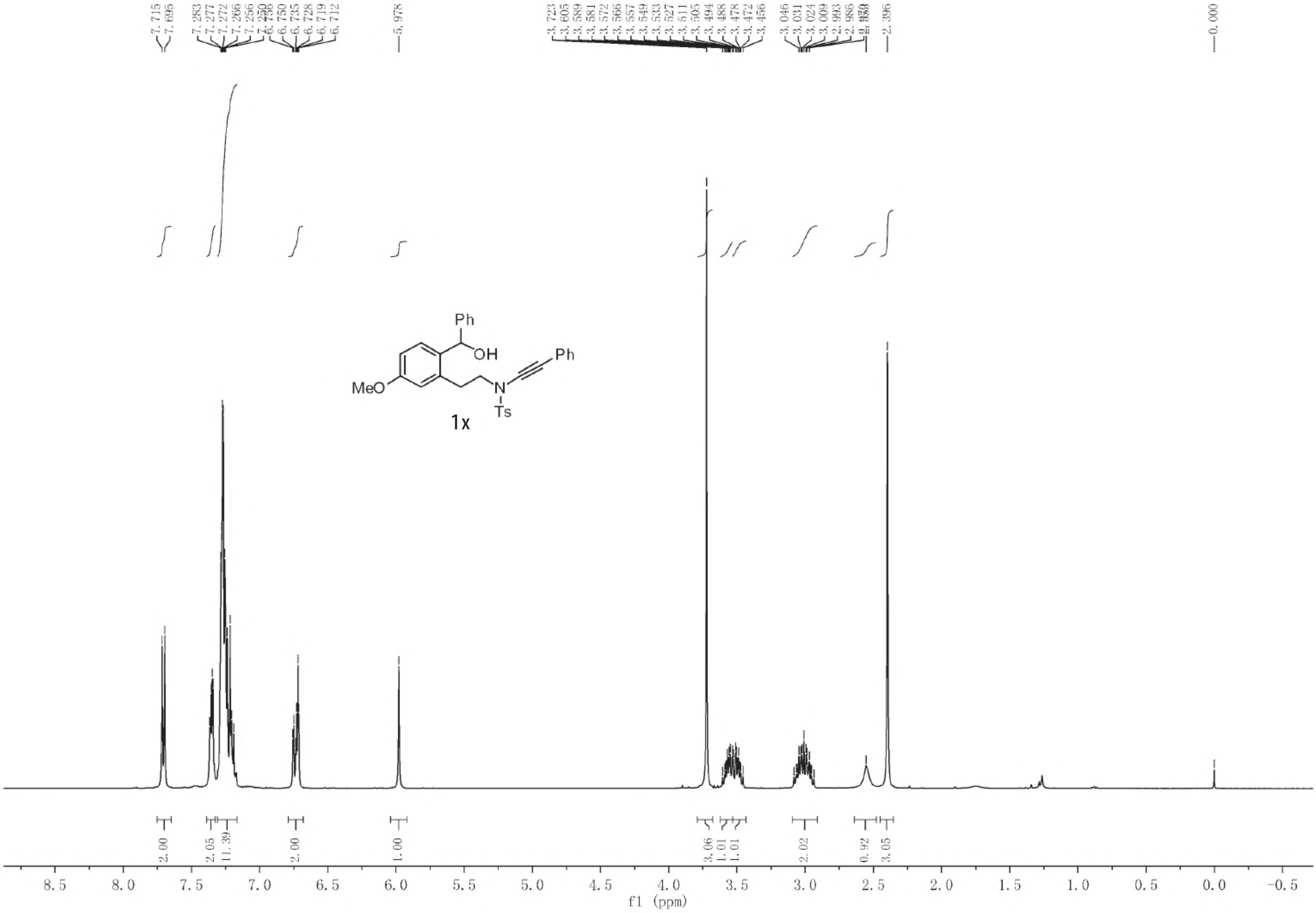

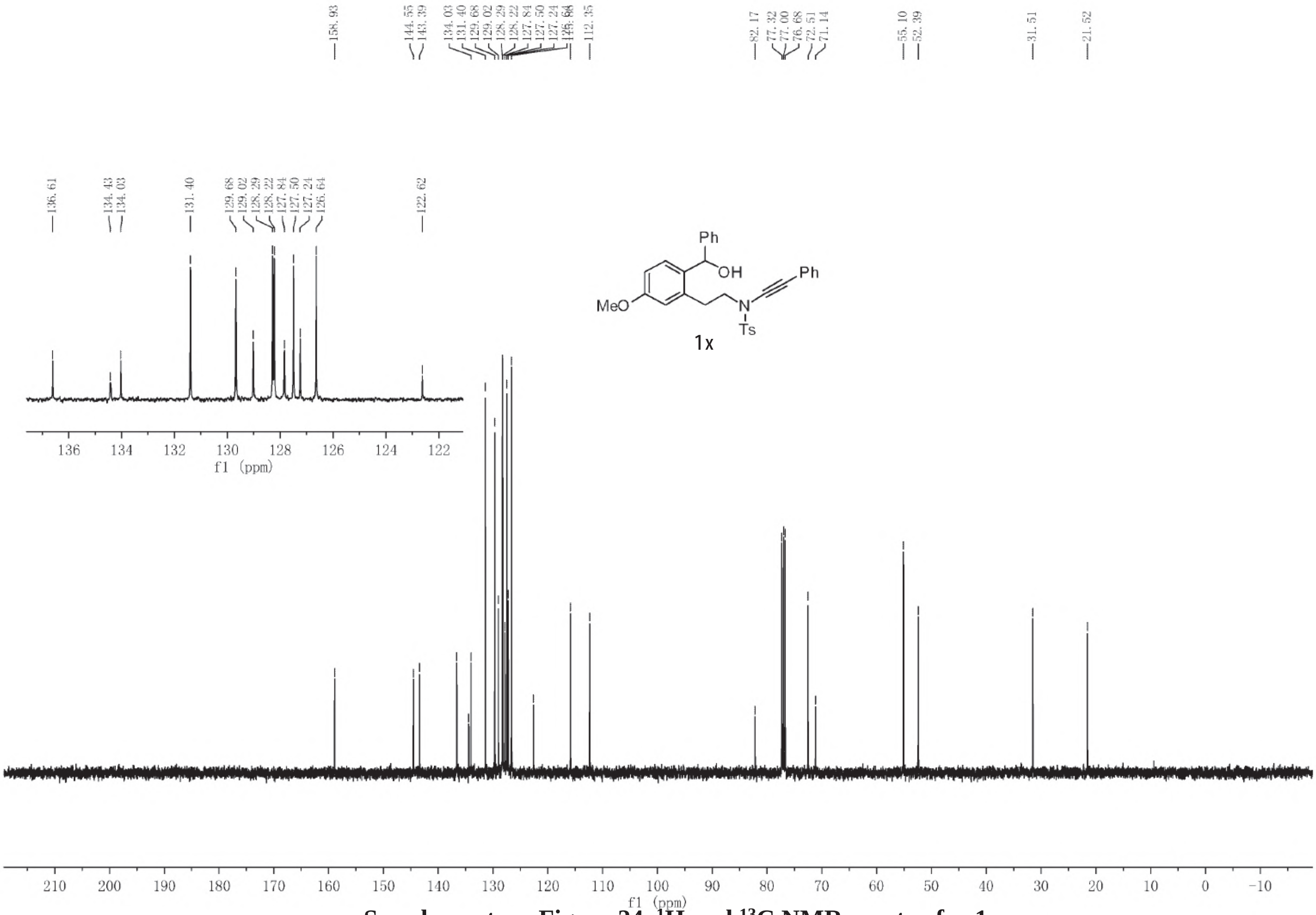

Supplementary Figure 24. ^{1}H and ^{13}C NMR spectra for 1x

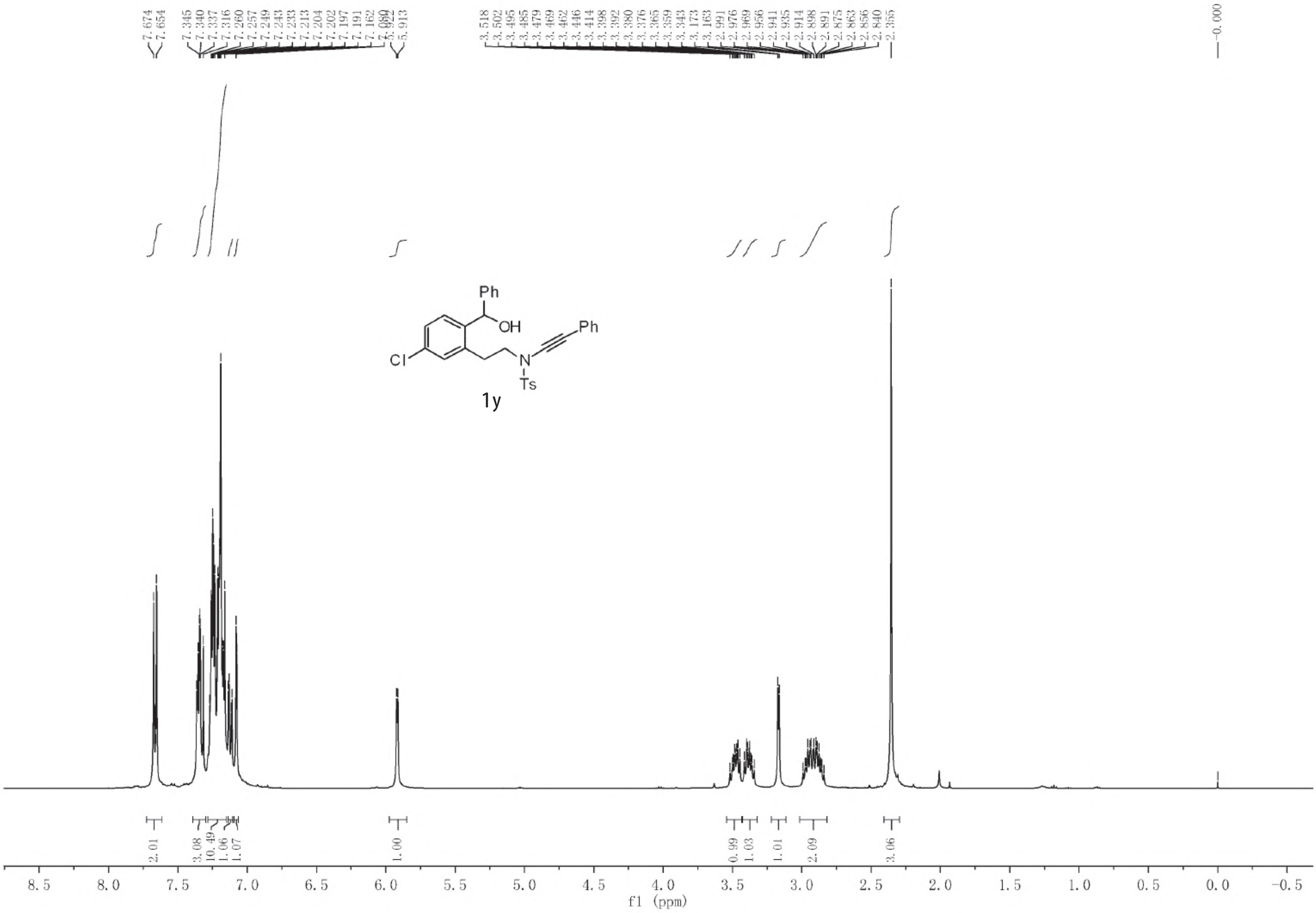

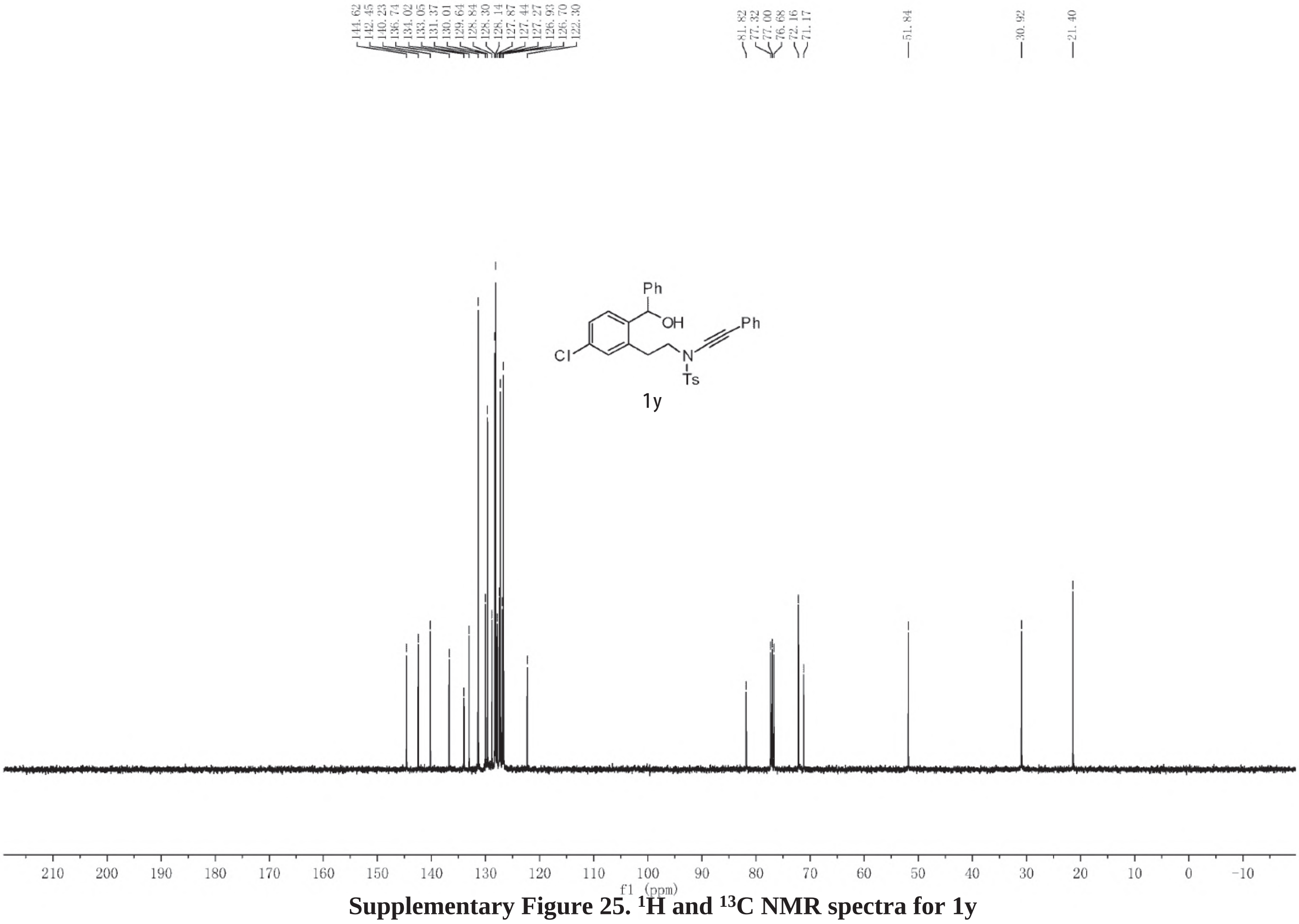

Supplementary Figure 25. ^{1}H and ^{13}C NMR spectra for 1y

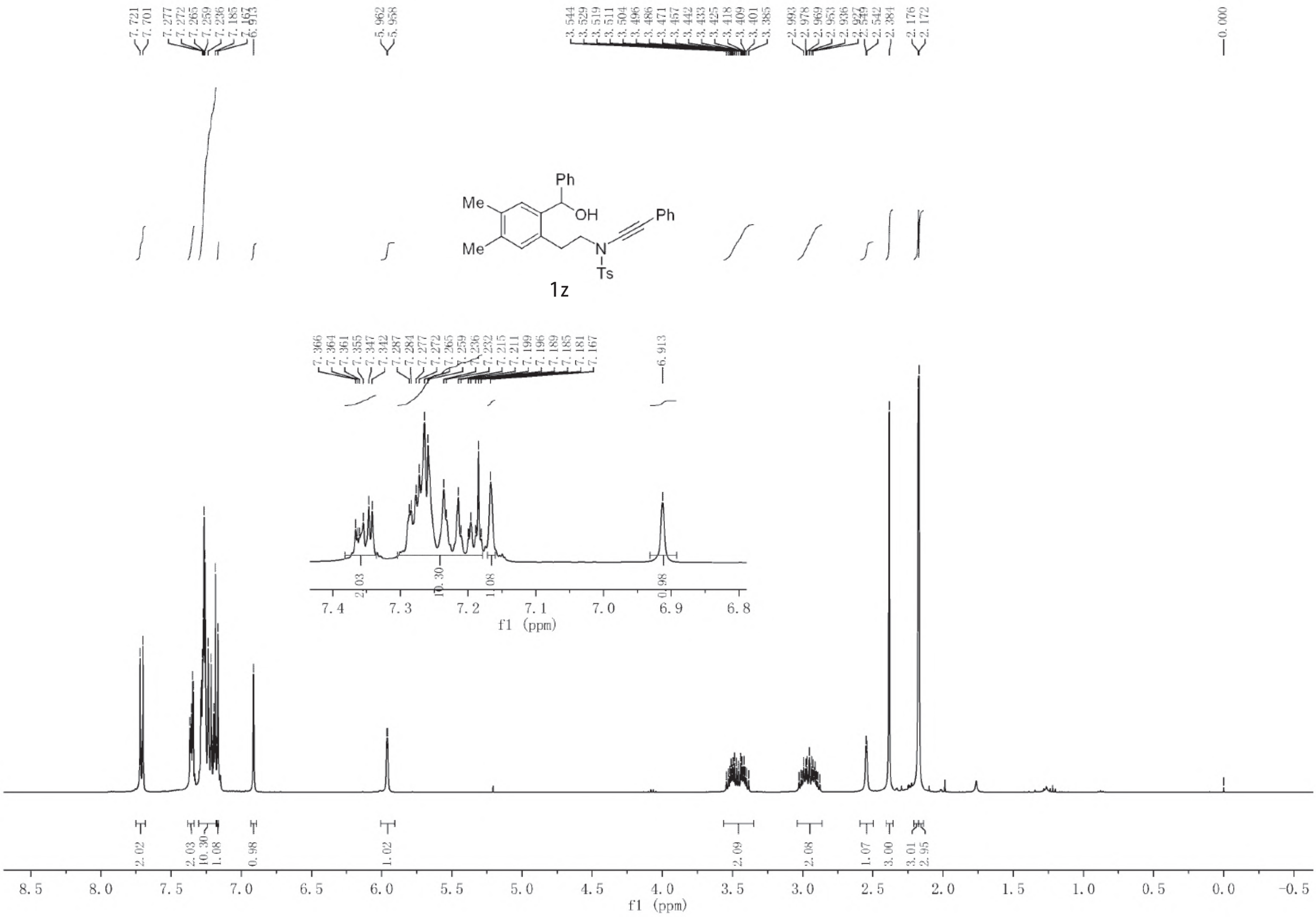

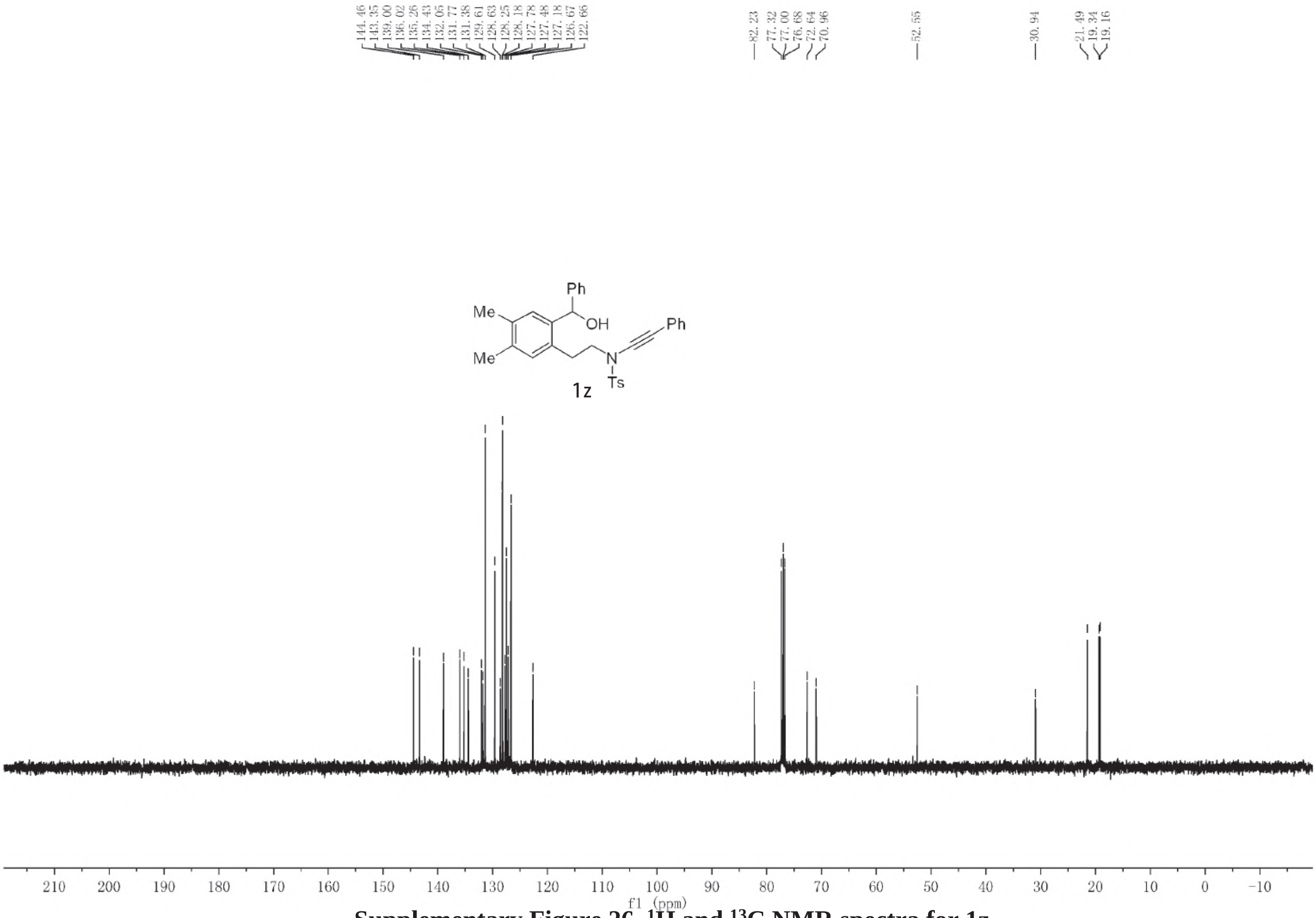

Supplementary Figure 26. ^{1}H and ^{13}C NMR spectra for 1z

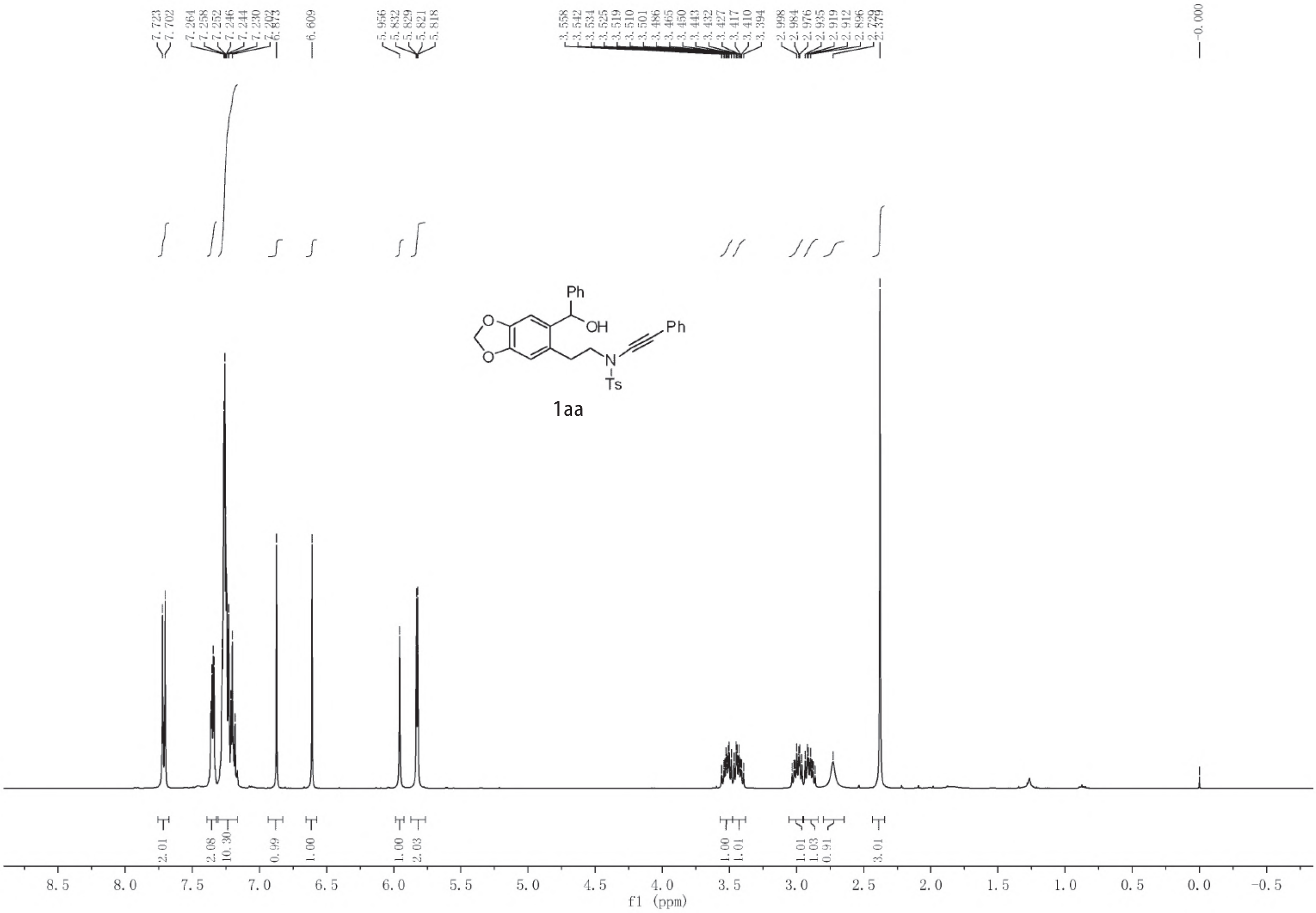

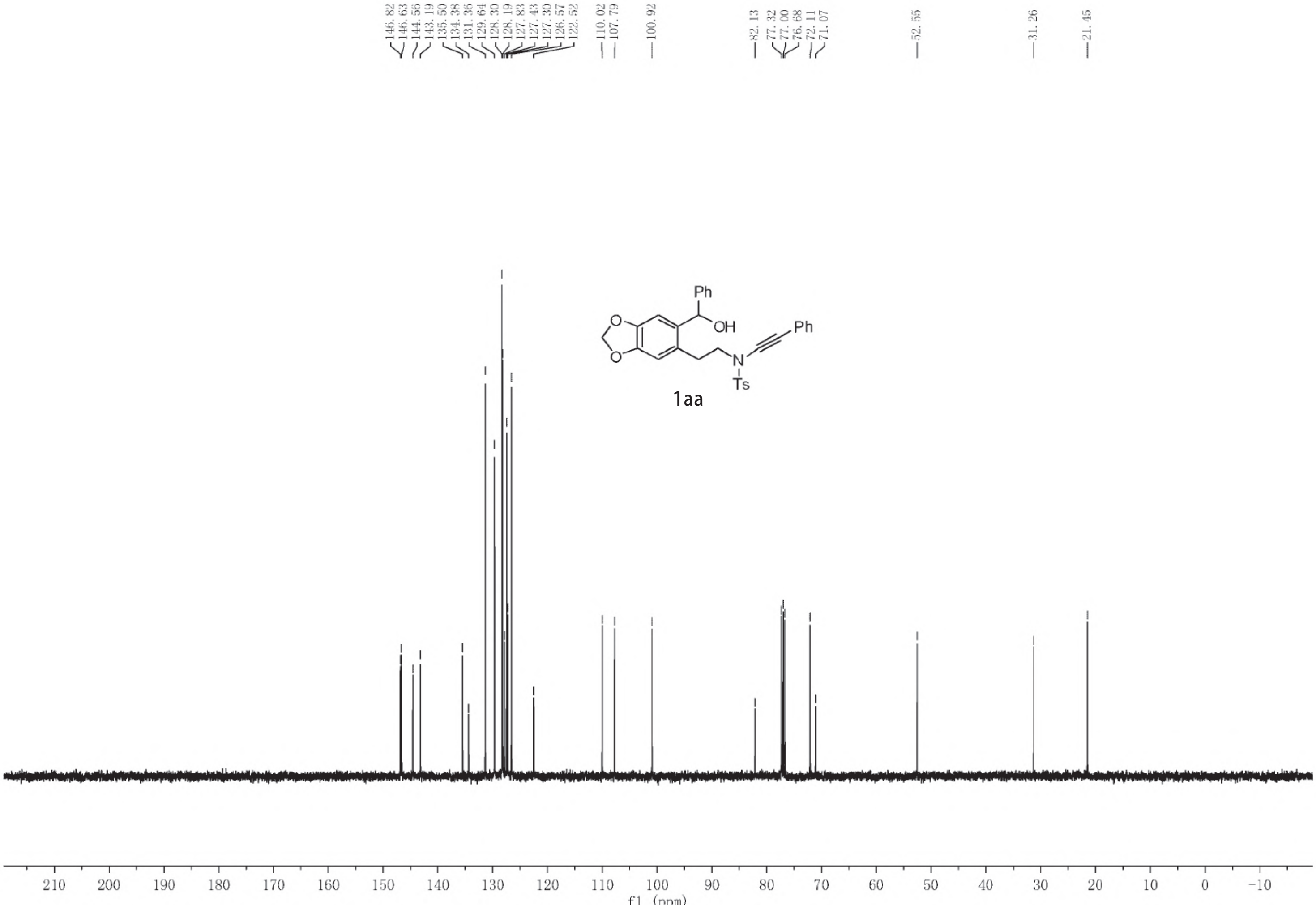

Supplementary Figure 27. ^{1}H and ^{13}C NMR spectra for 1aa

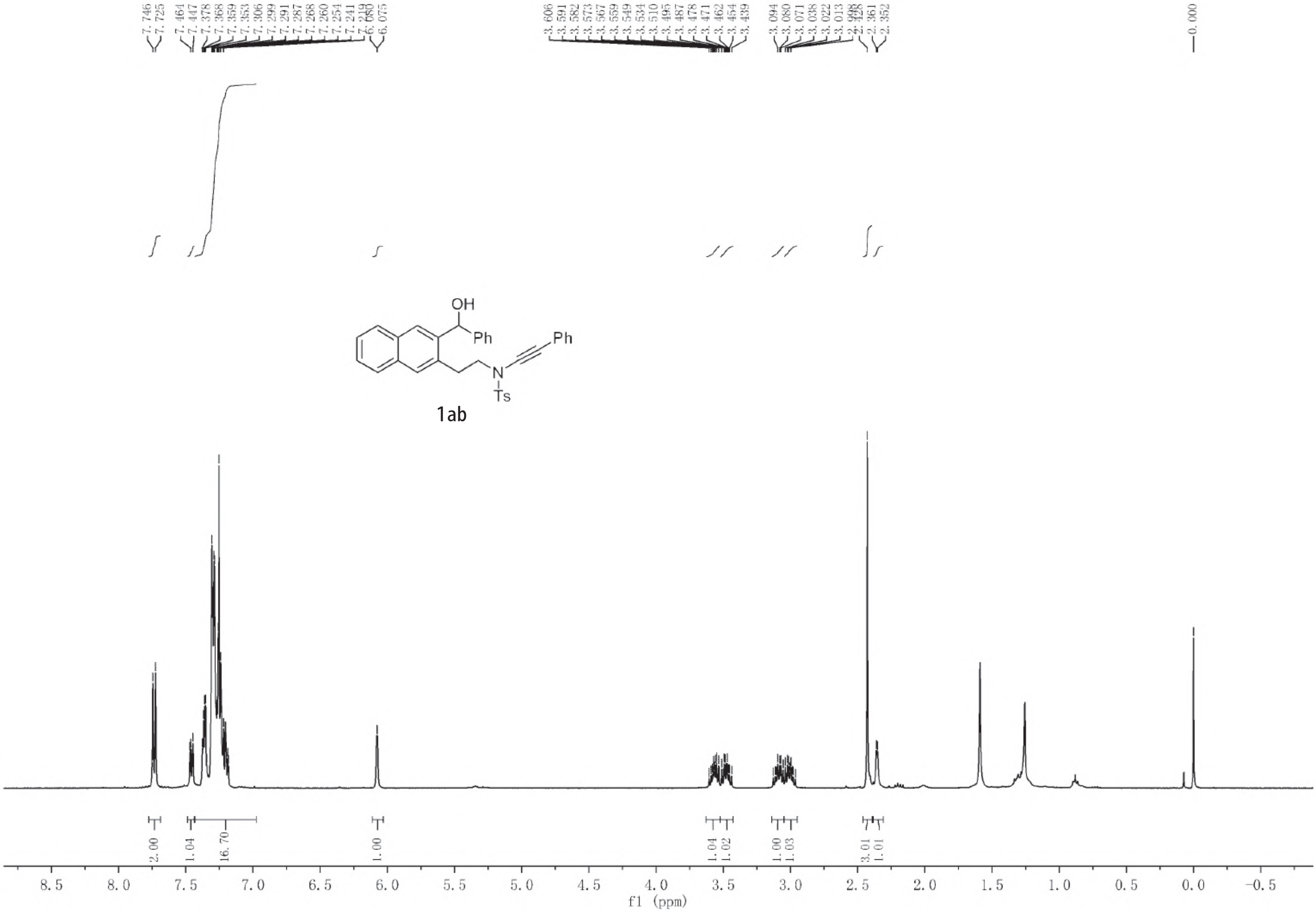

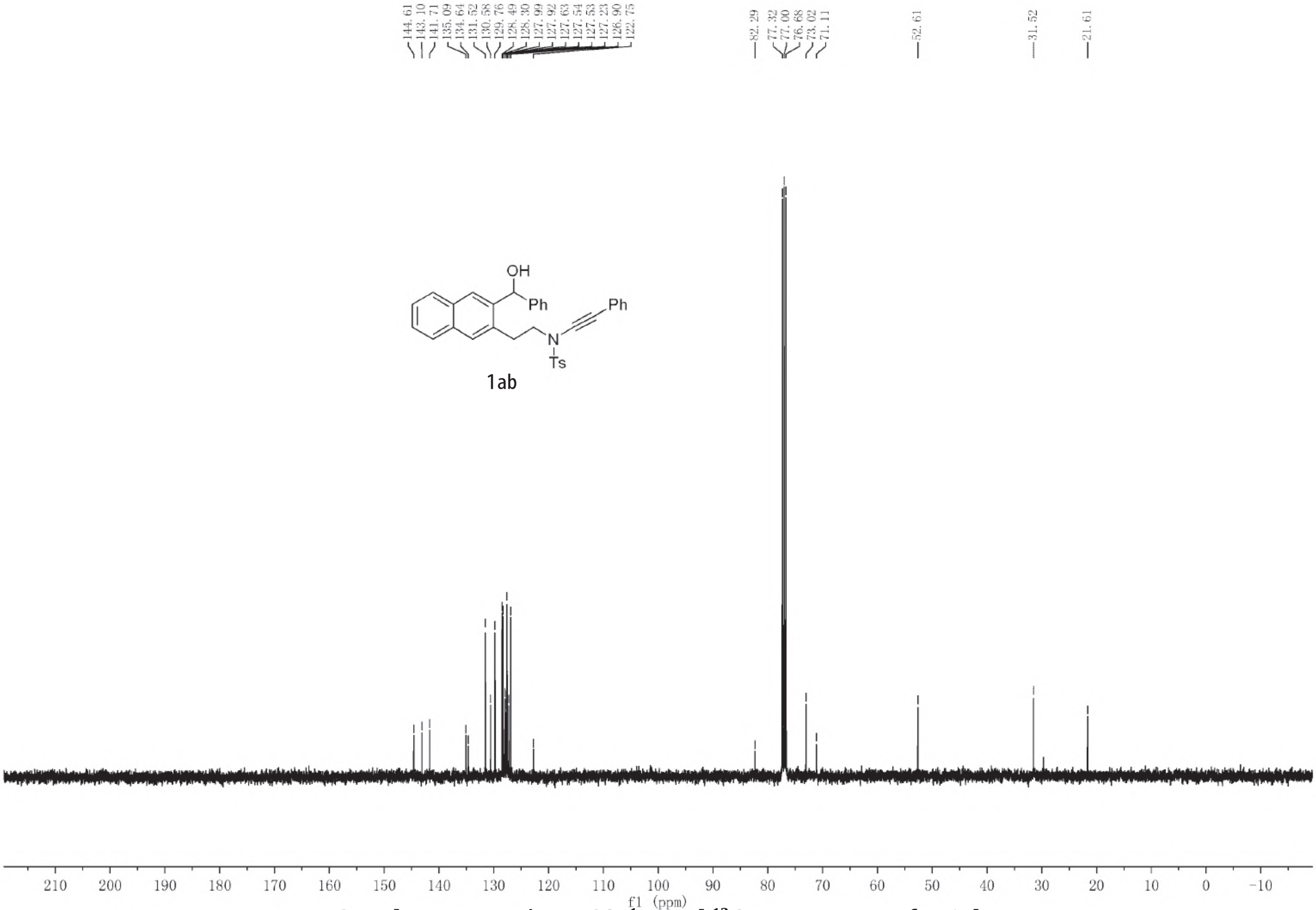

Supplementary Figure 28. ^{1}H and ^{13}C NMR spectra for 1ab

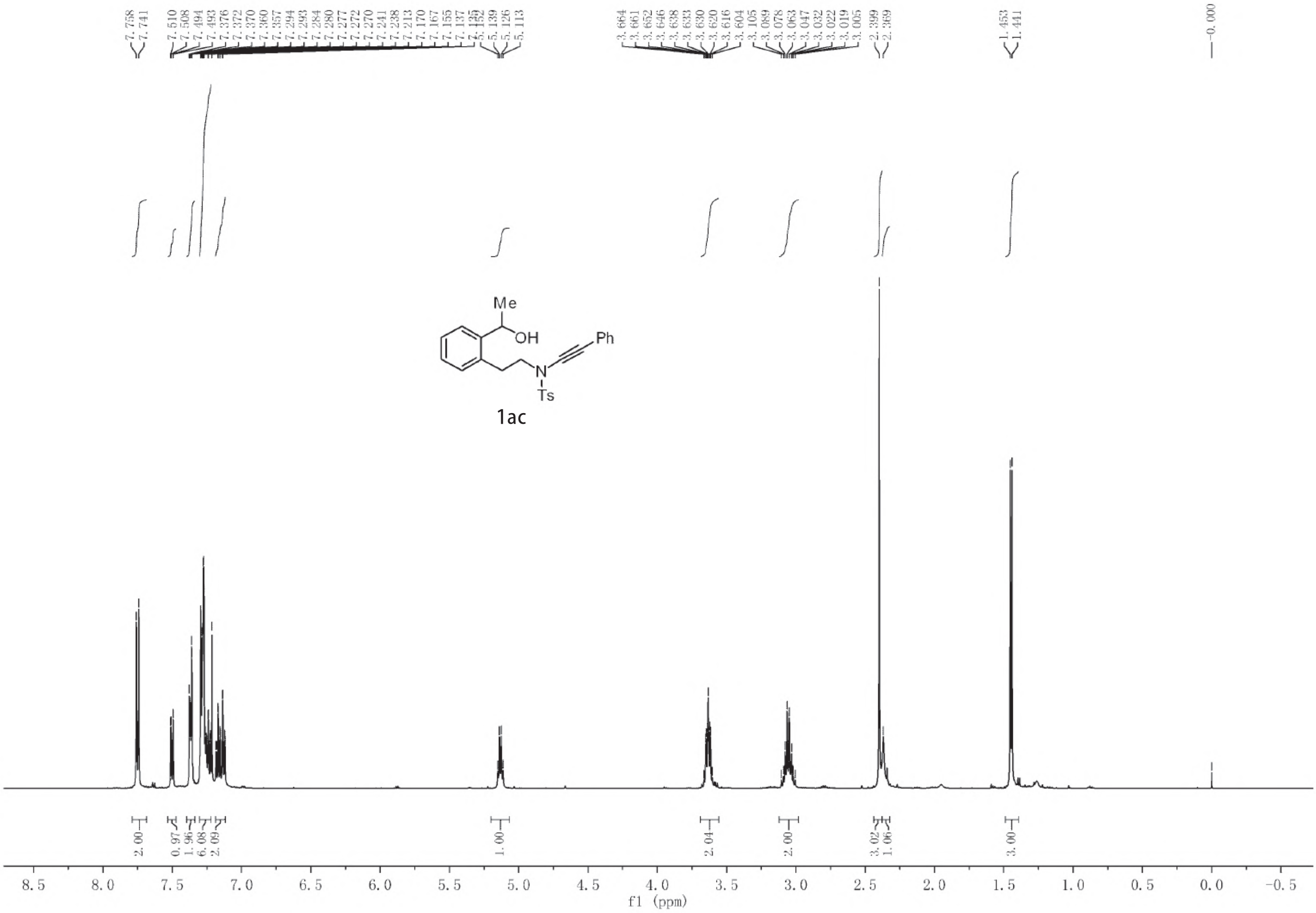

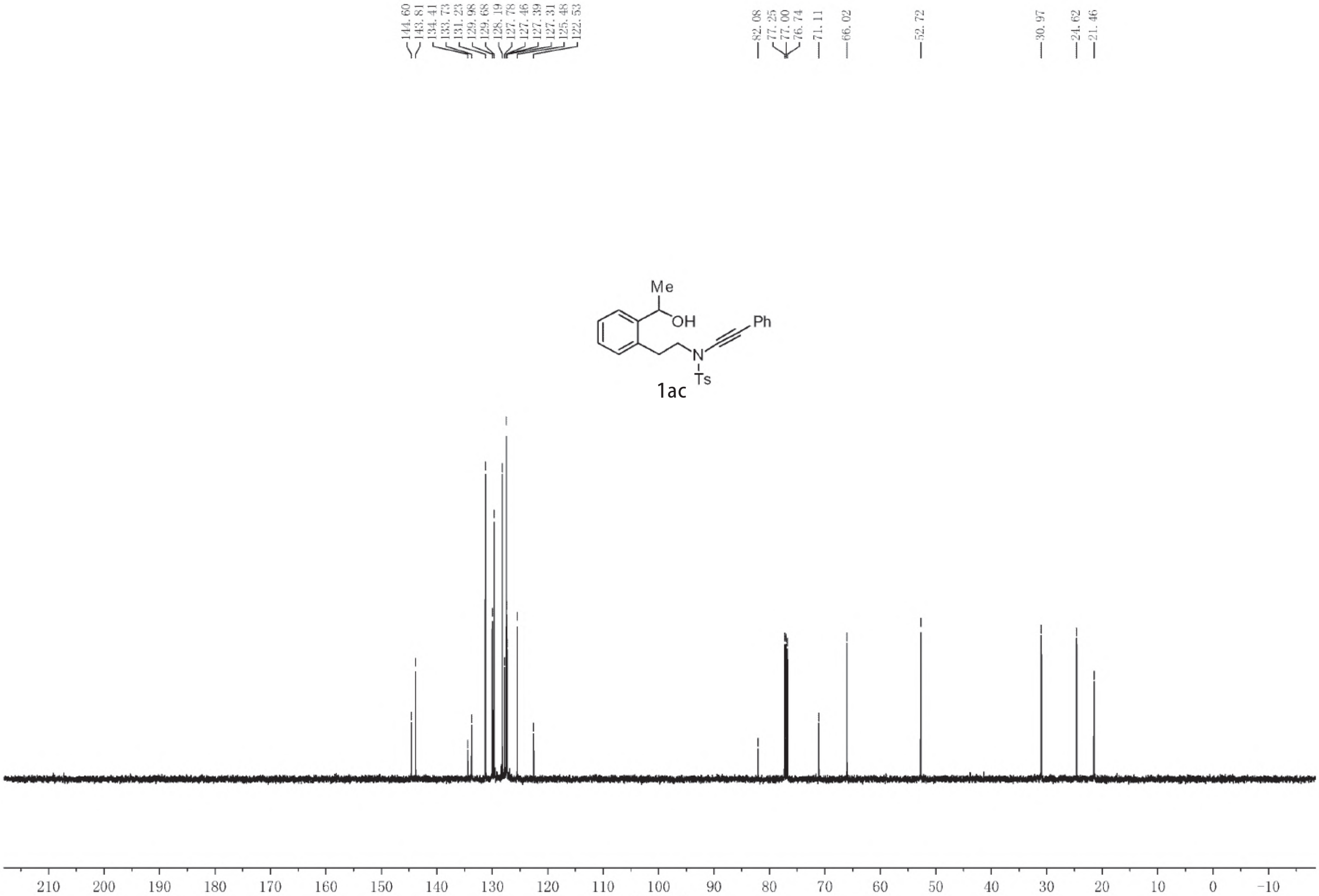

Supplementary Figure 29. ^{1}H and ^{13}C NMR spectra for 1ac

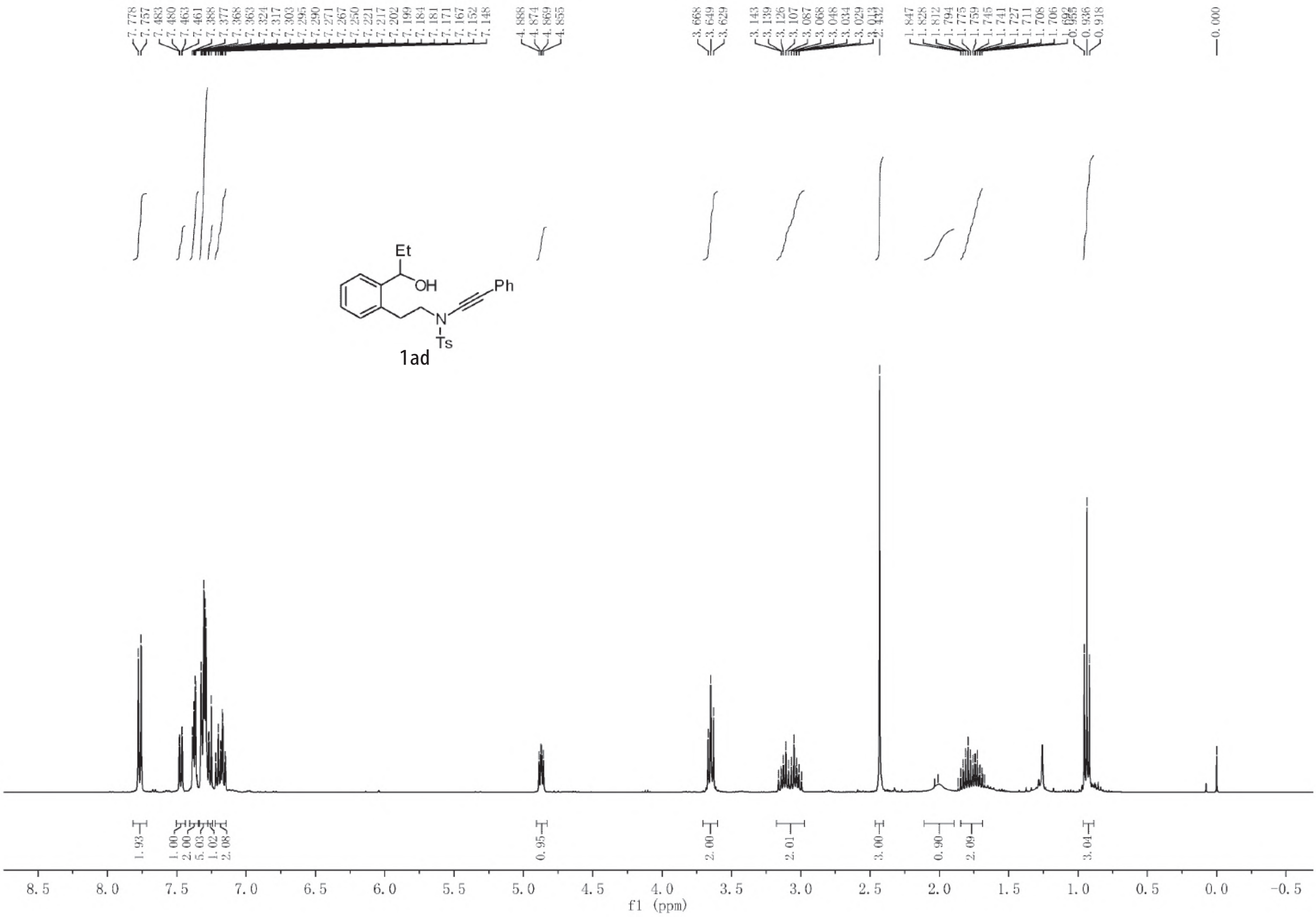

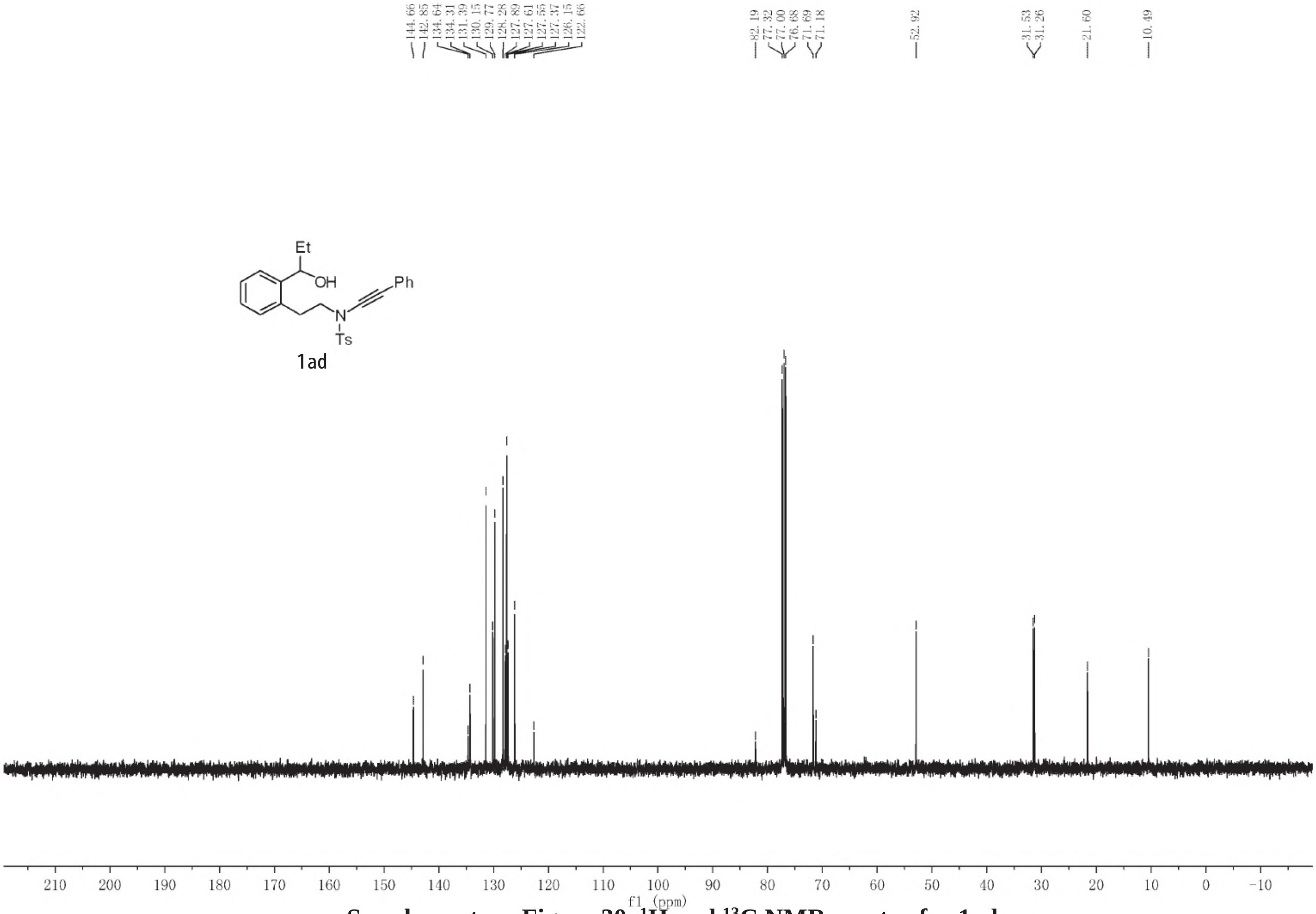

Supplementary Figure 30. ^{1}H and ^{13}C NMR spectra for 1ad

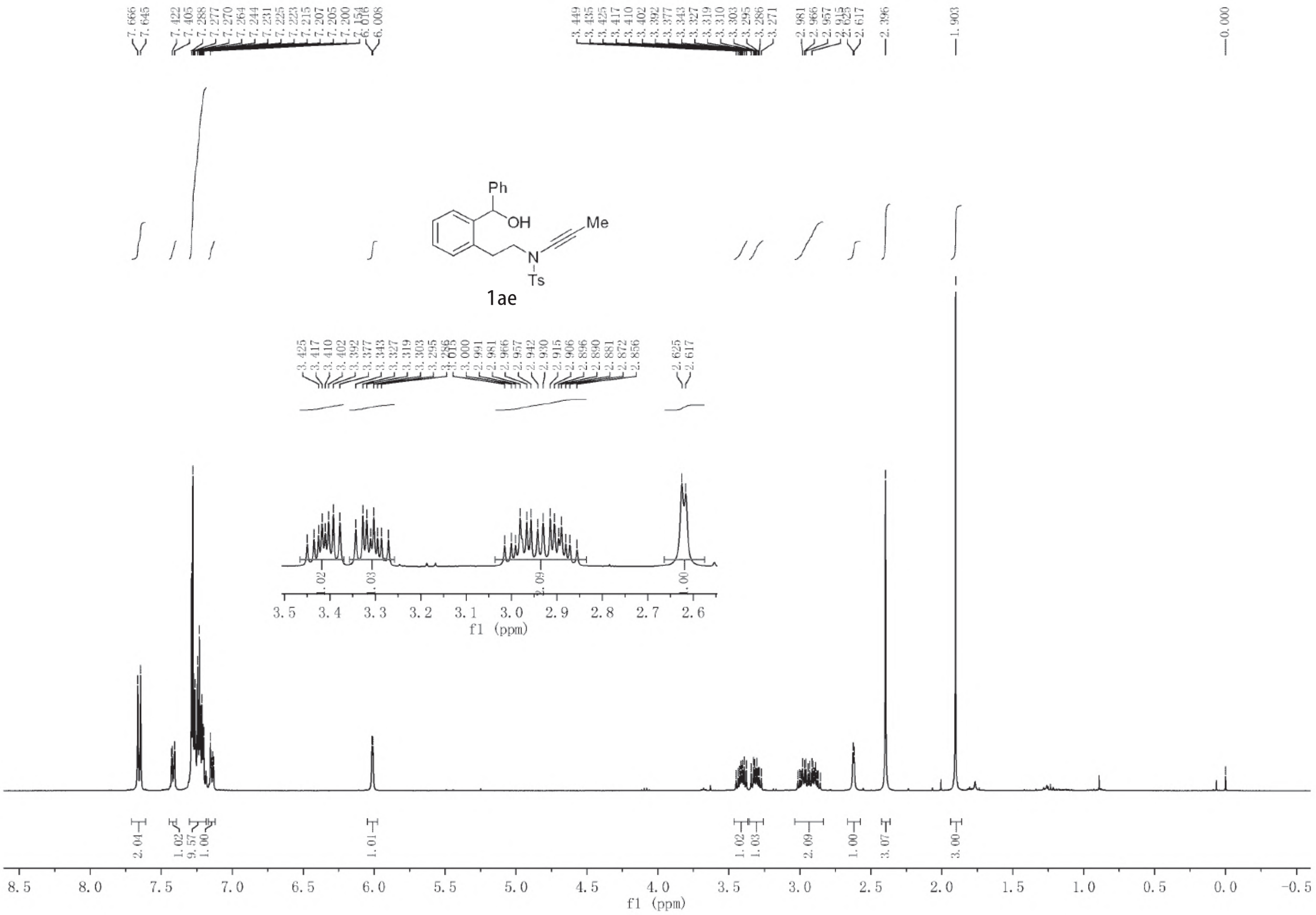

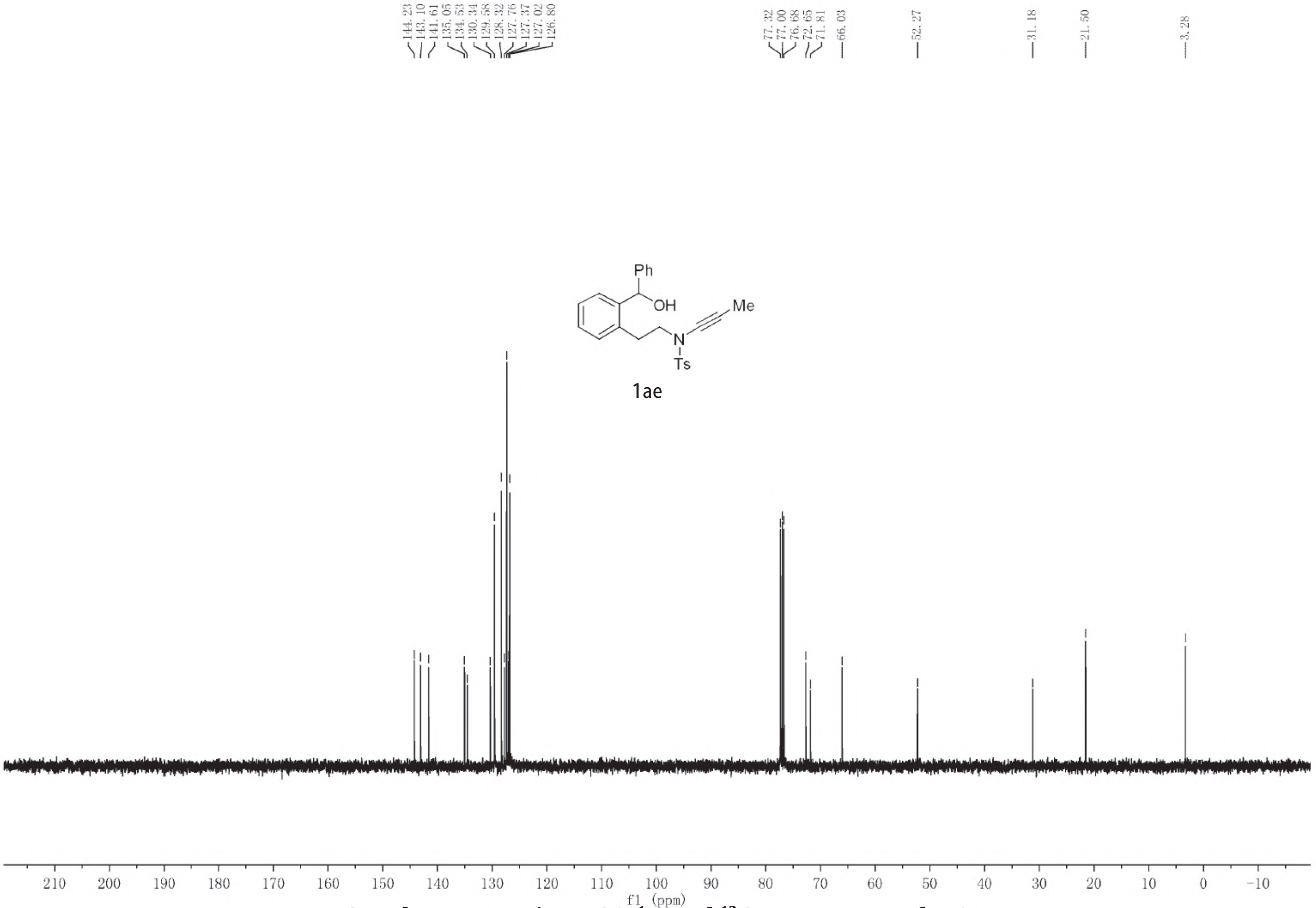

Supplementary Figure 31. ^{1}H and ^{13}C NMR spectra for 1ae

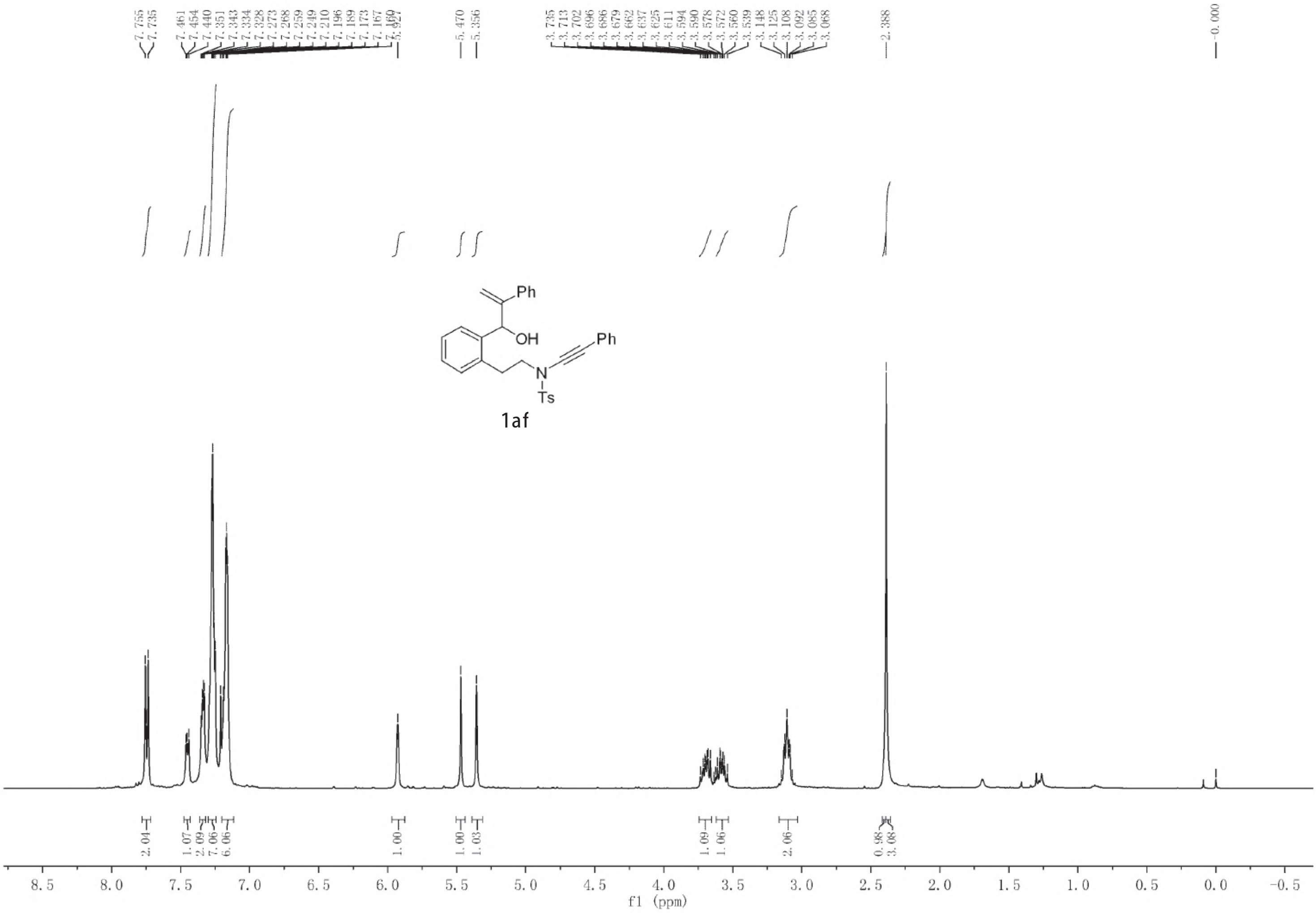

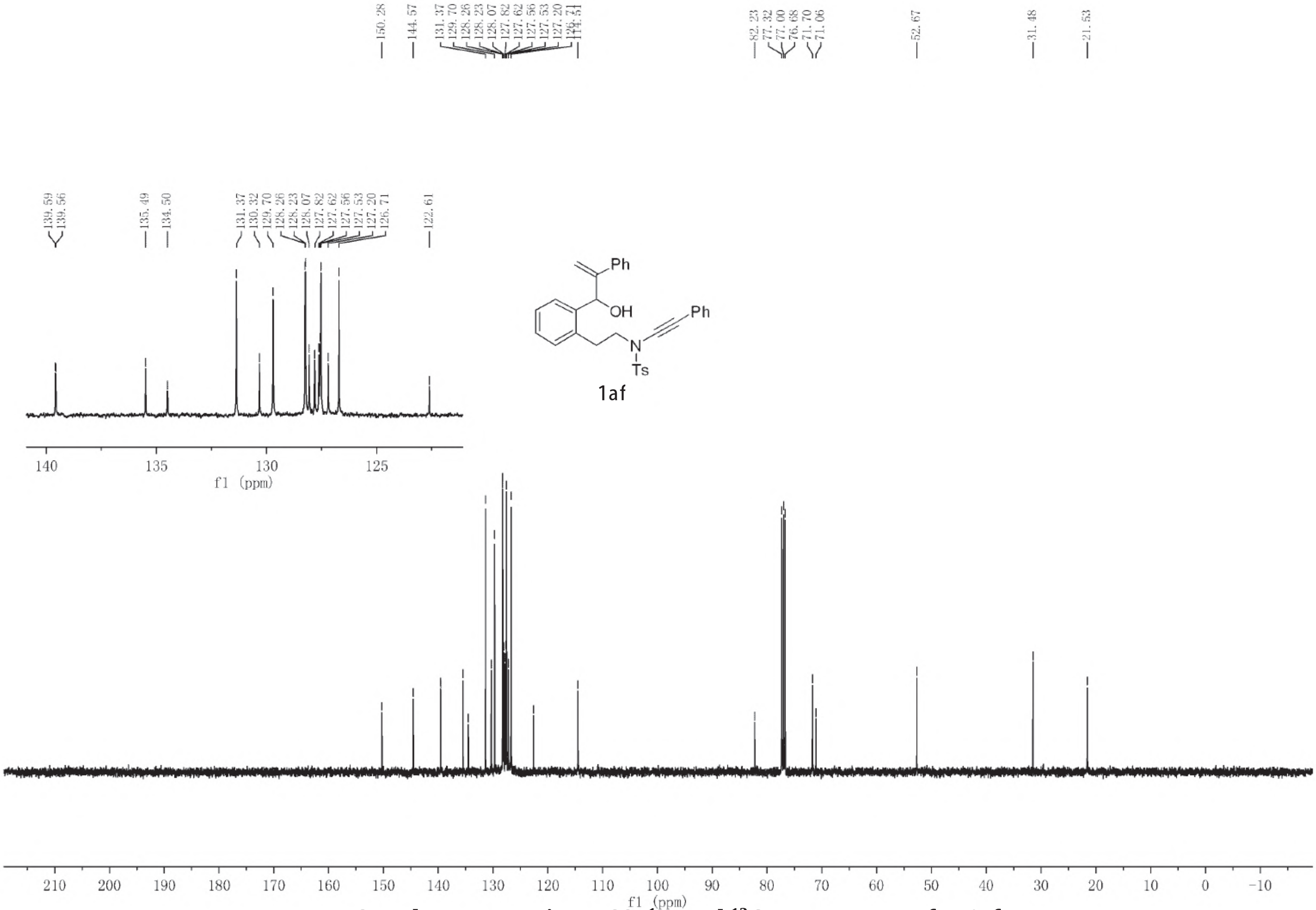

Supplementary Figure 32. ^{1}H and ^{13}C NMR spectra for 1af

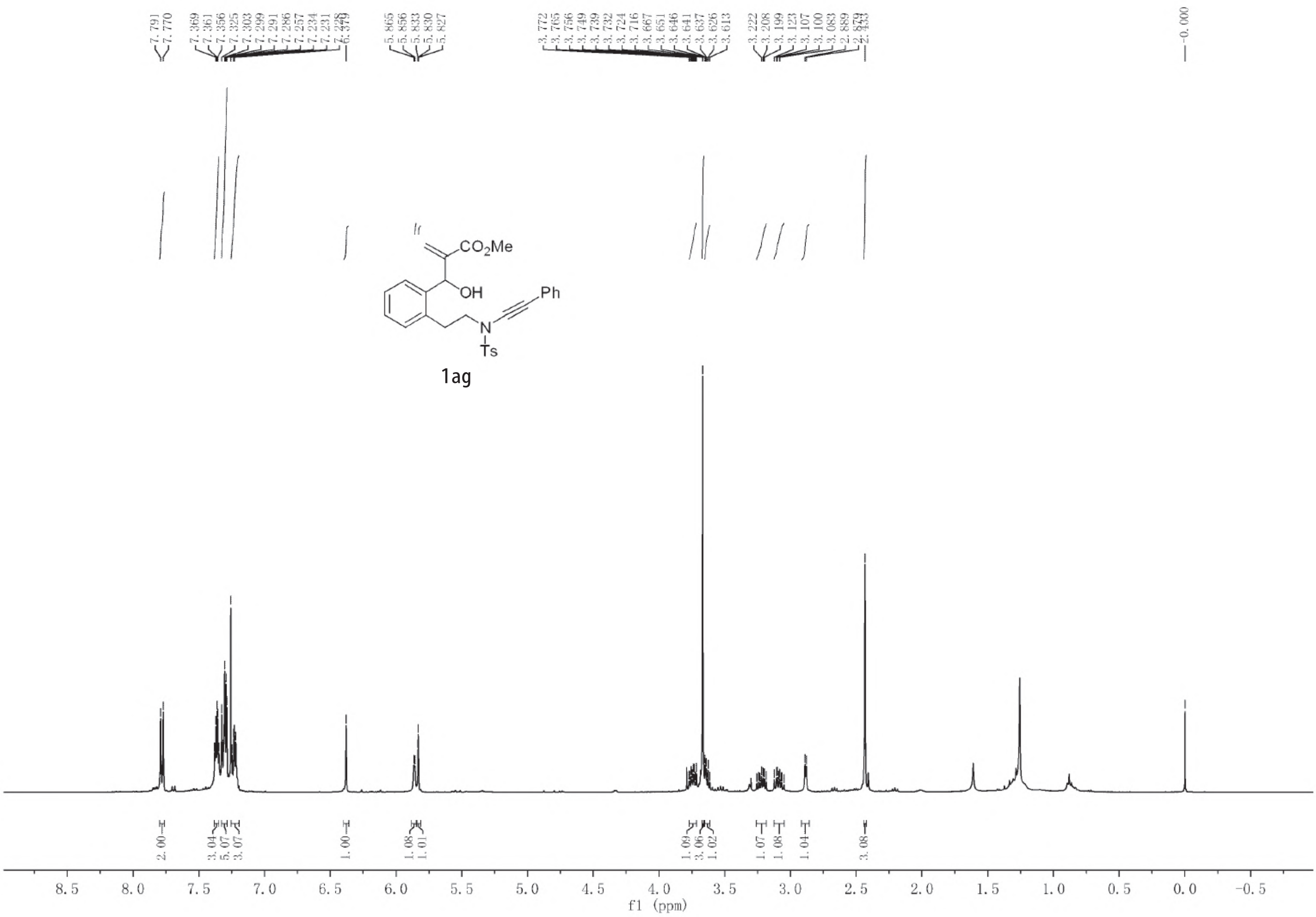

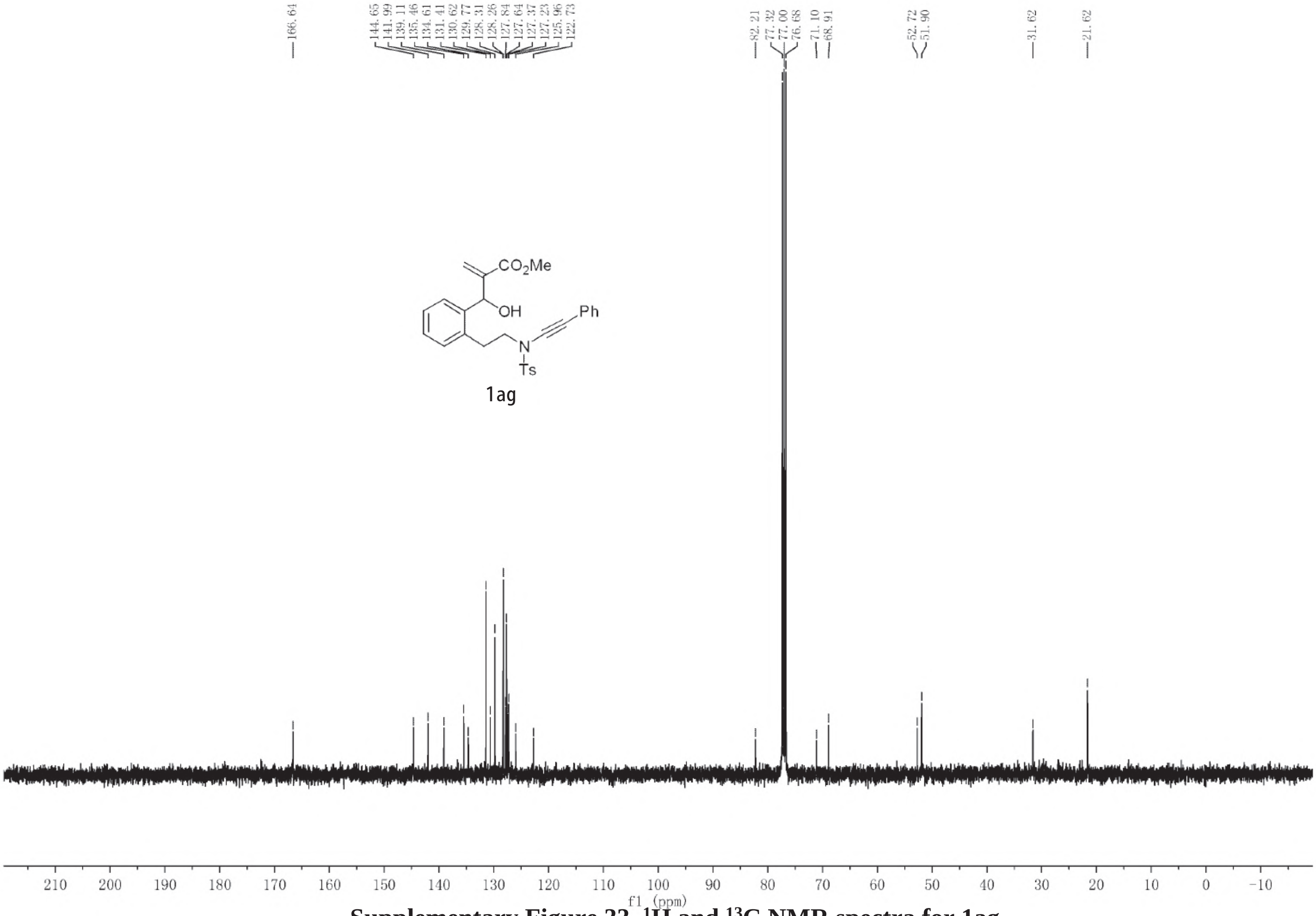

Supplementary Figure 33. ^{1}H and ^{13}C NMR spectra for 1ag

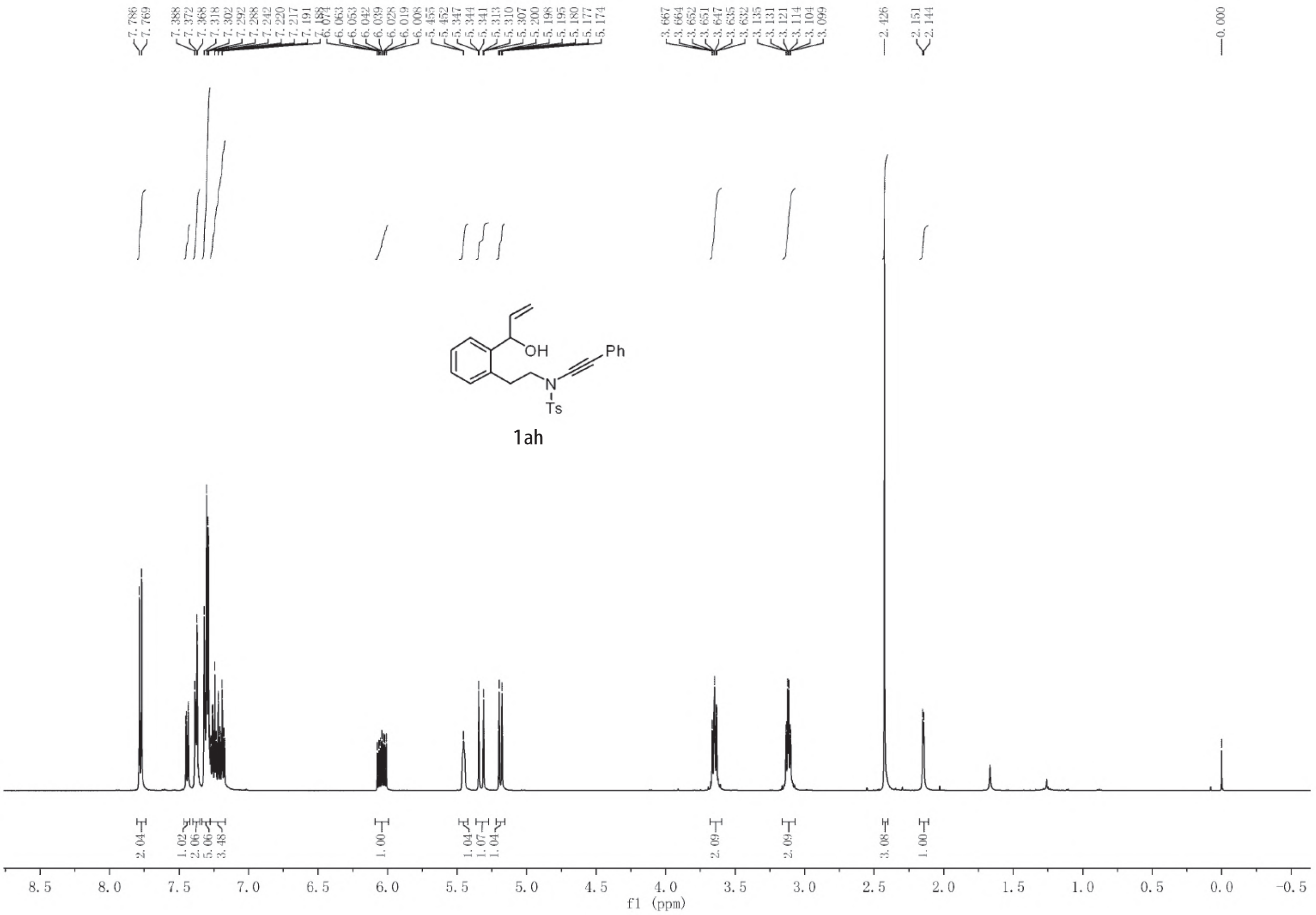

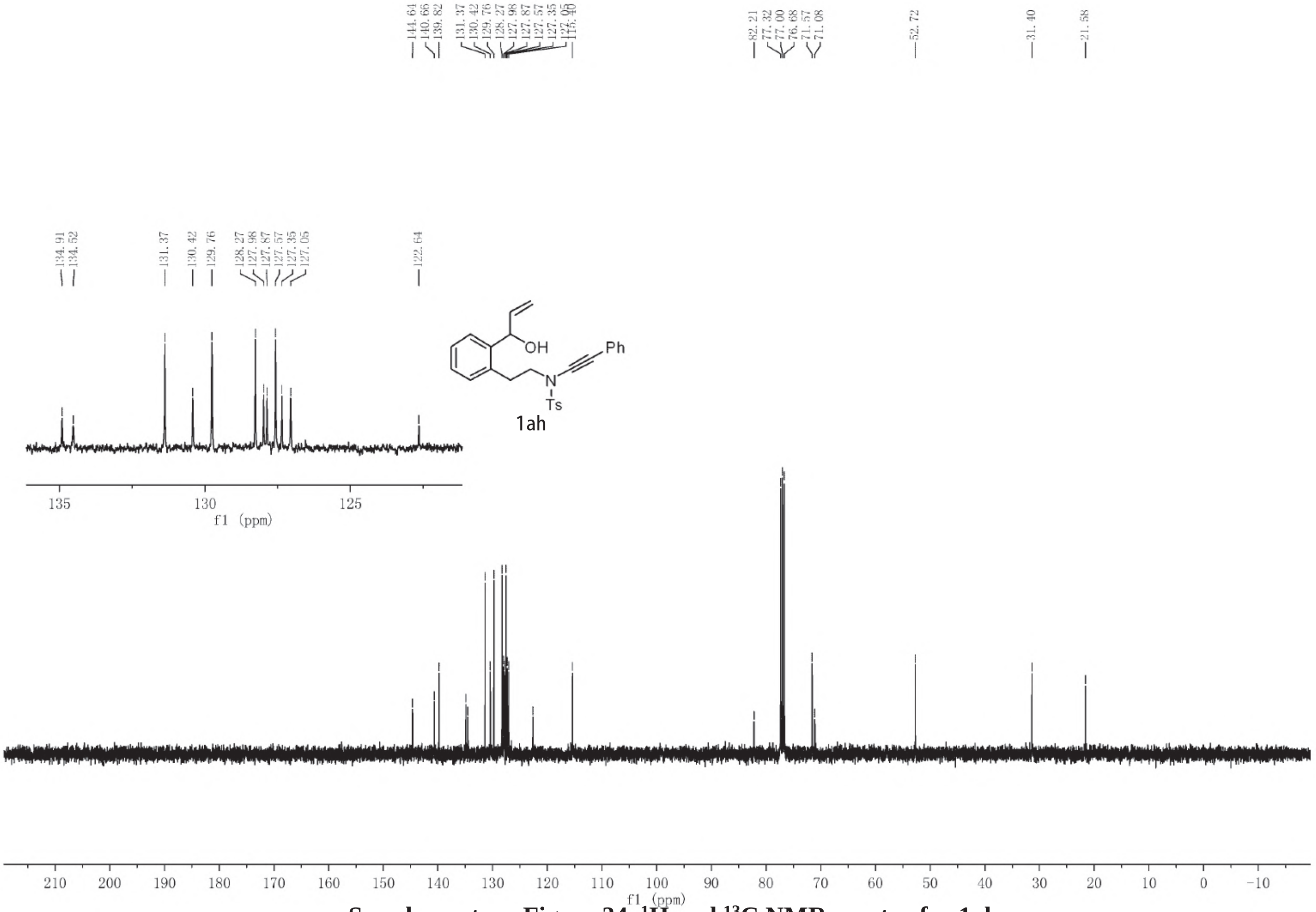

Supplementary Figure 34. ^{1}H and ^{13}C NMR spectra for 1ah

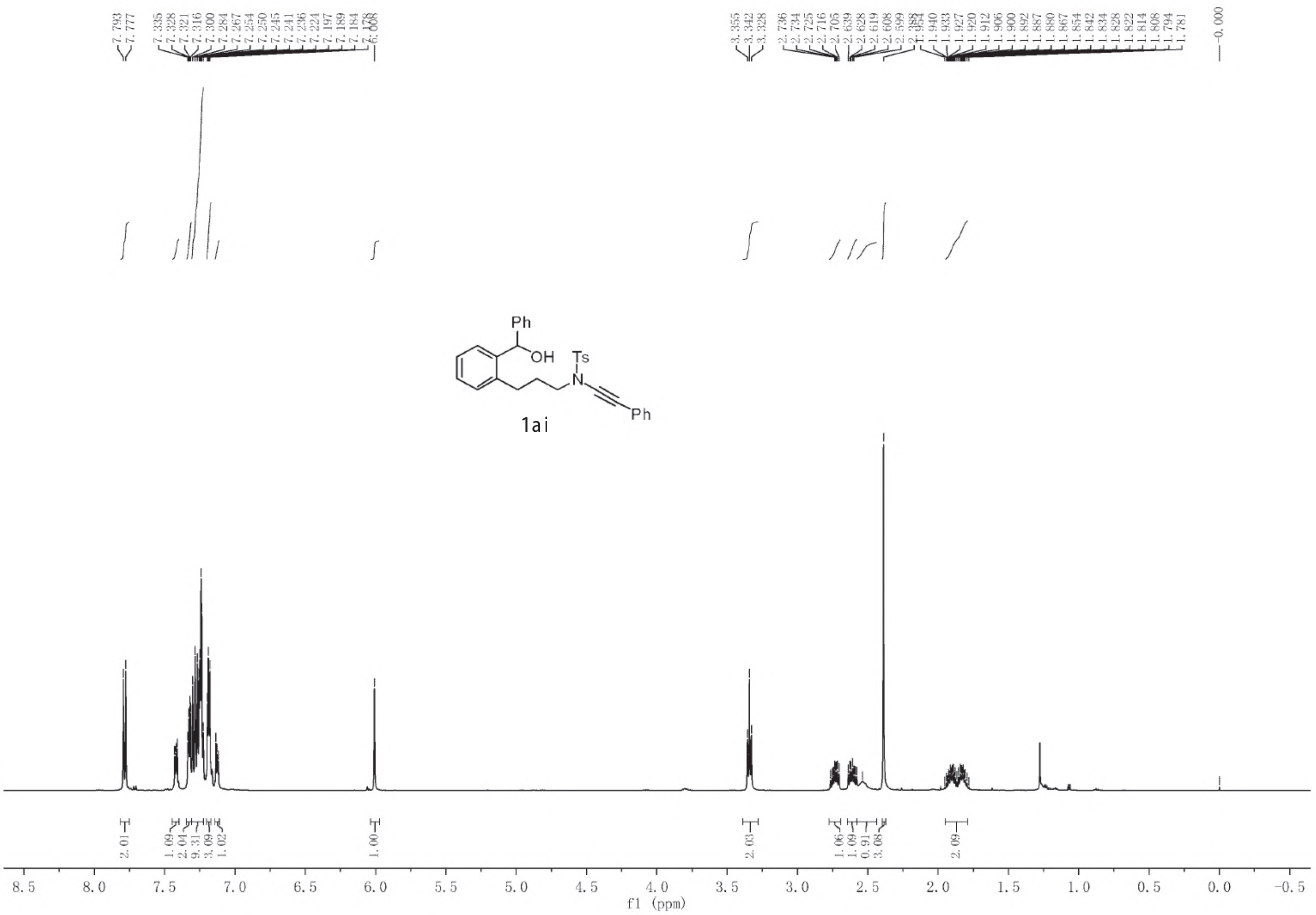

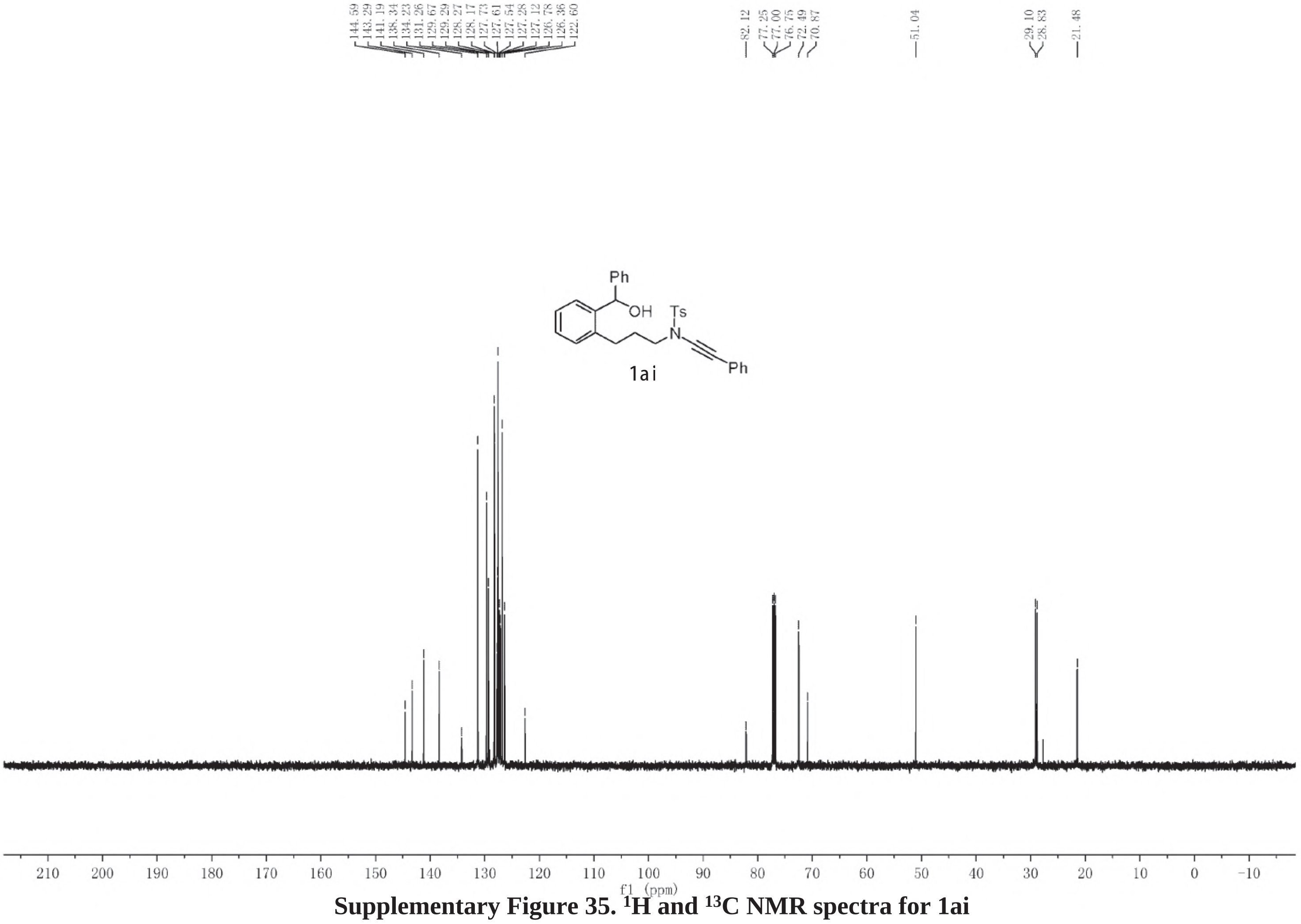

Supplementary Figure 35. ^{1}H and ^{13}C NMR spectra for 1ai

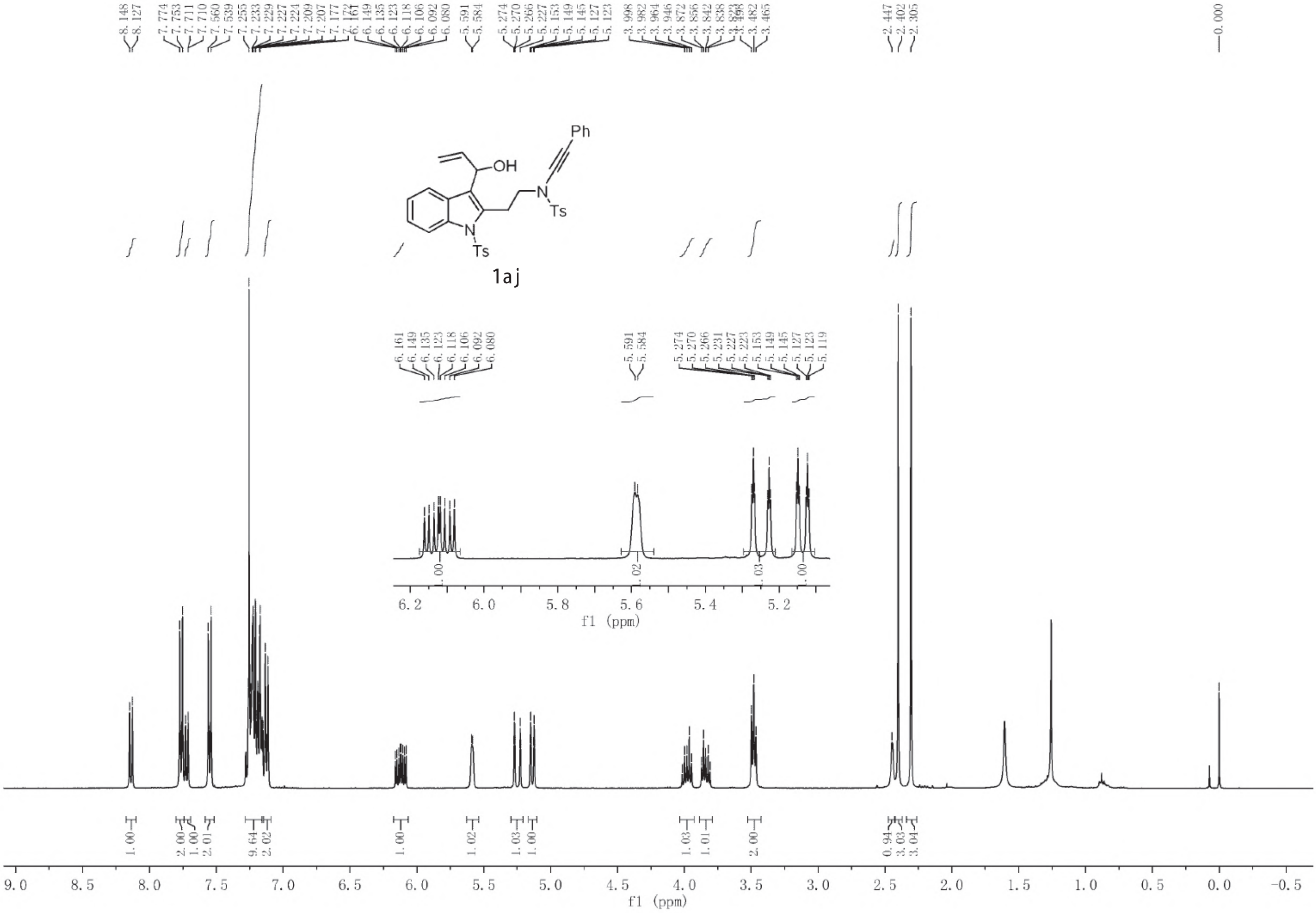

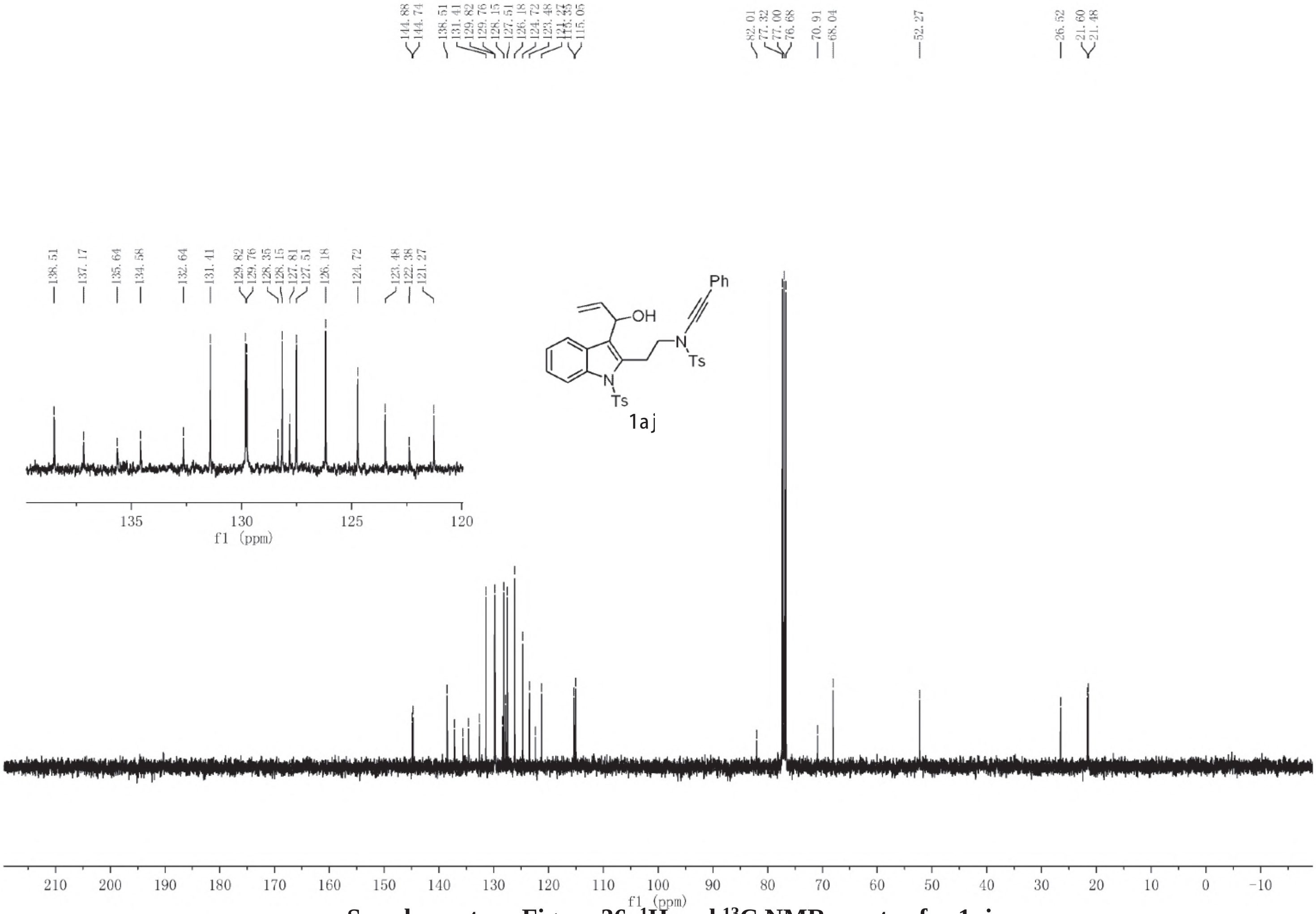

Supplementary Figure 36. ^{1}H and ^{13}C NMR spectra for 1aj

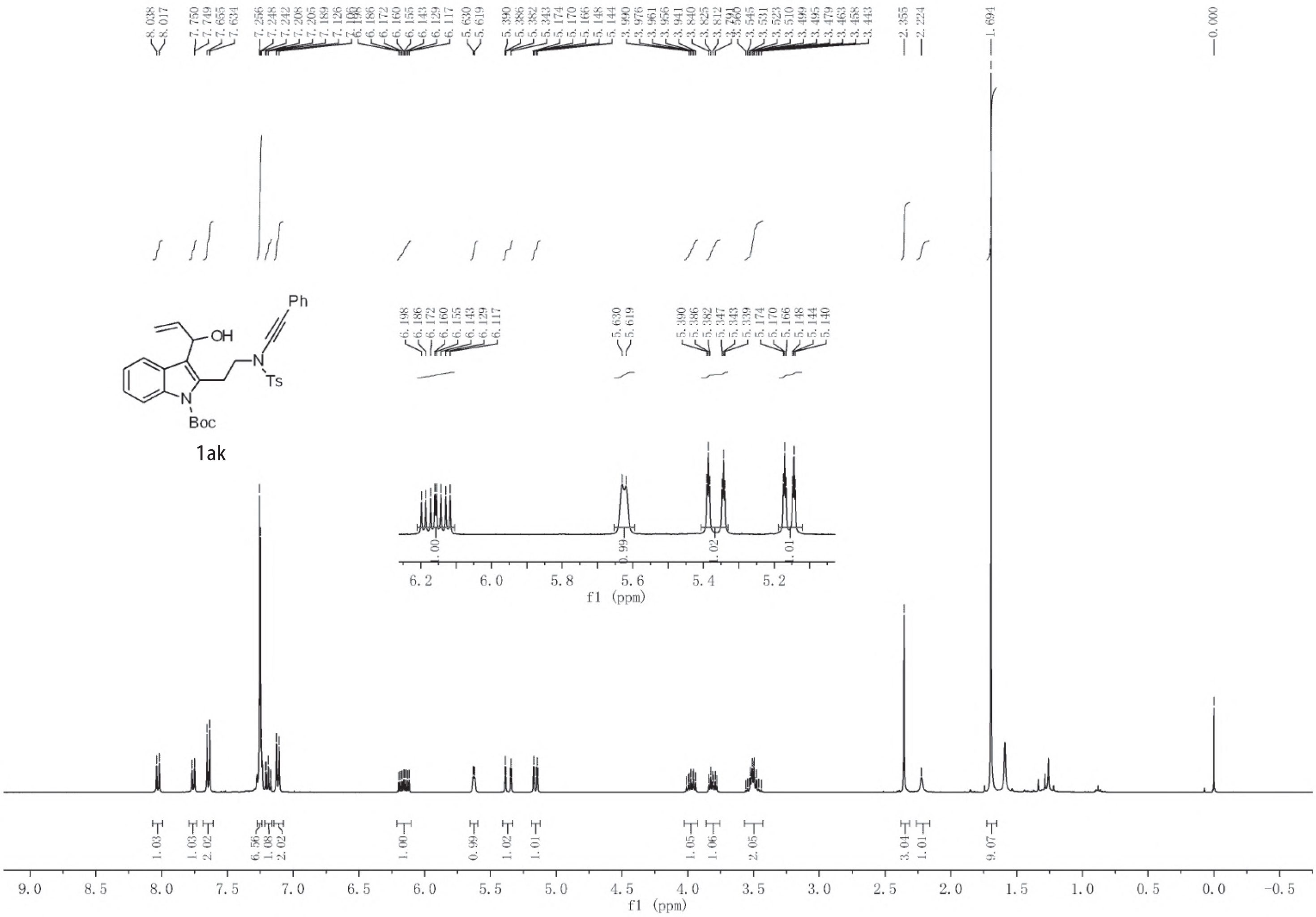

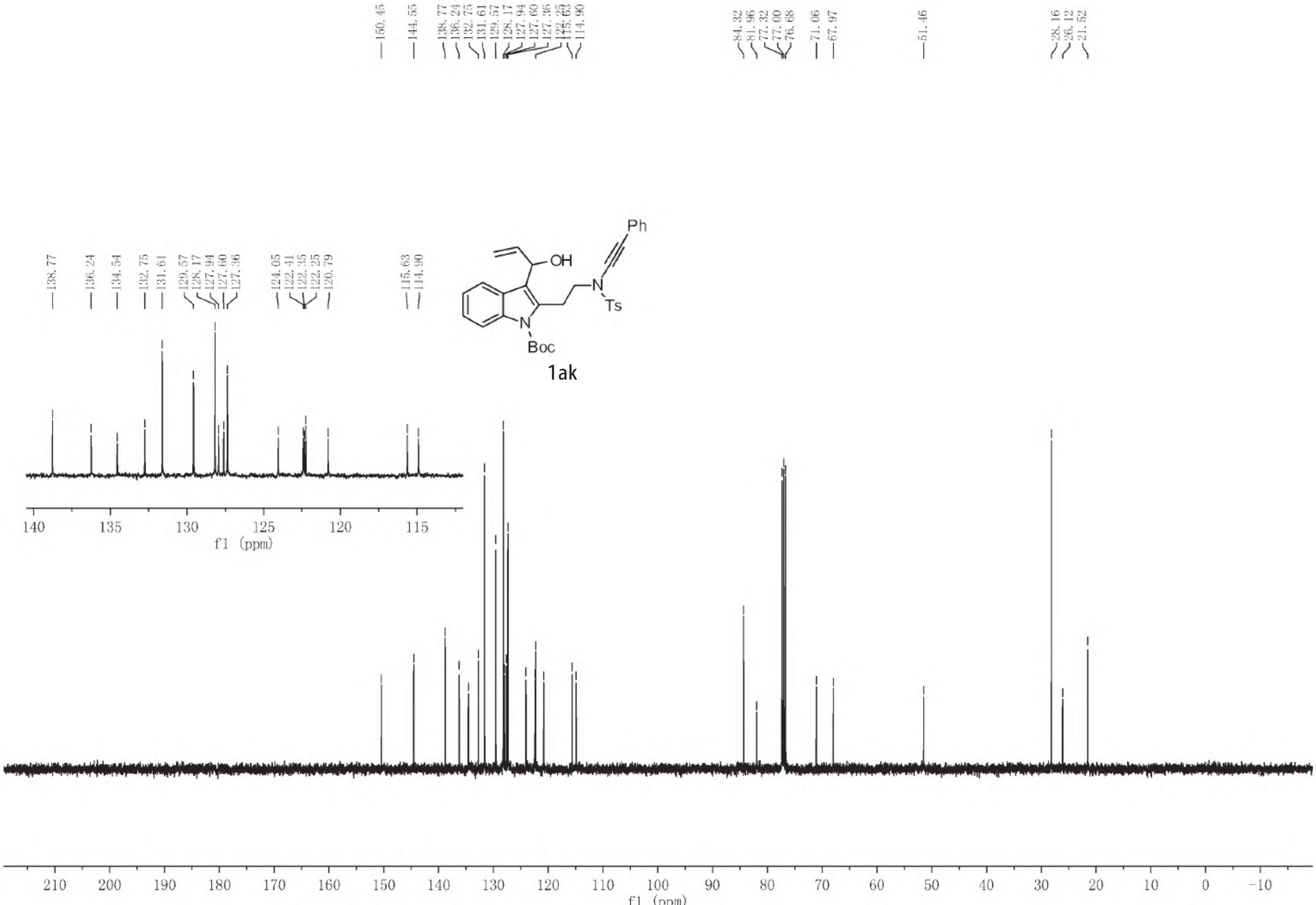

Supplementary Figure 37. ^{1}H and ^{13}C NMR spectra for 1ak

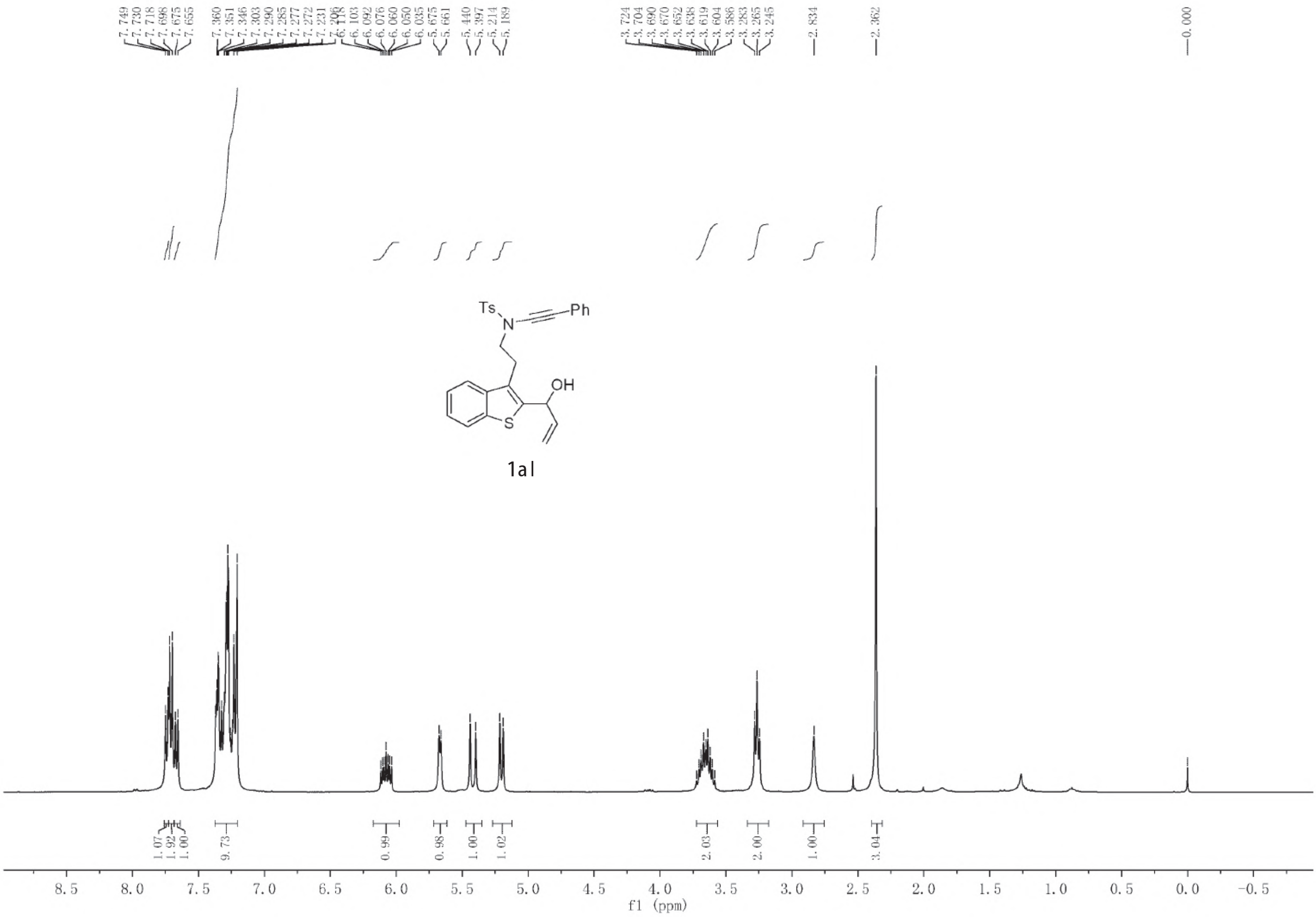

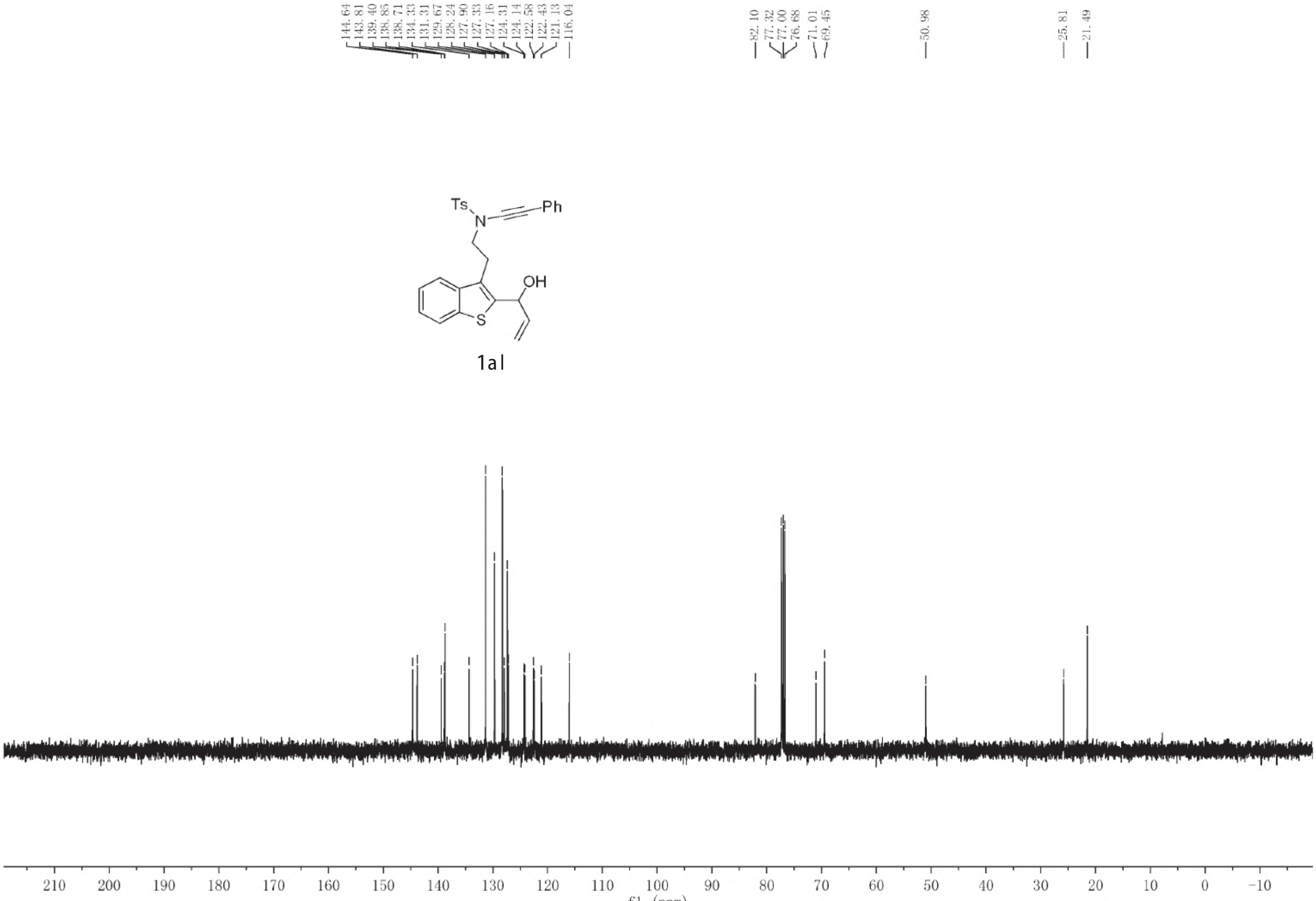

Supplementary Figure 38. ^{1}H and ^{13}C NMR spectra for 1al

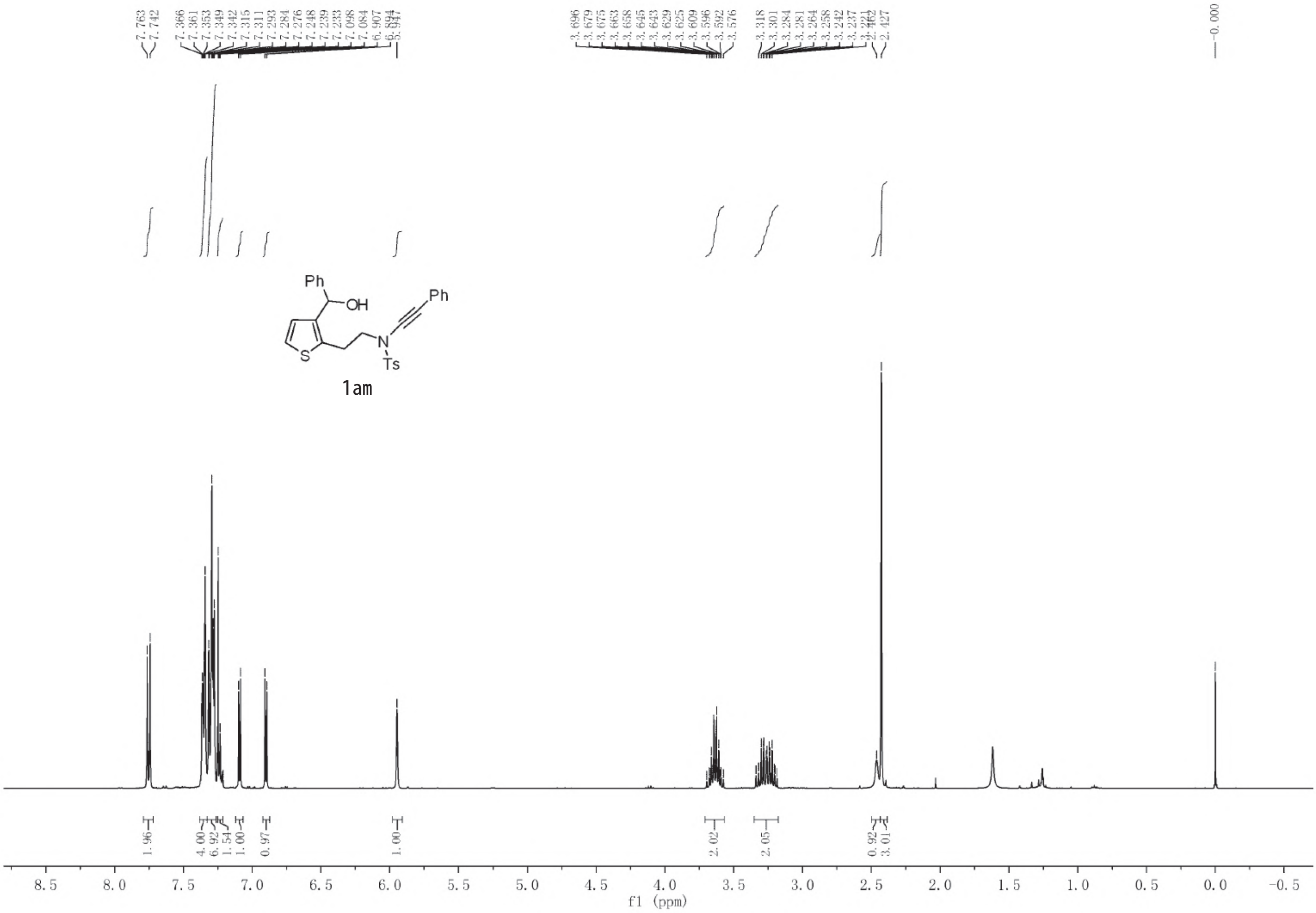

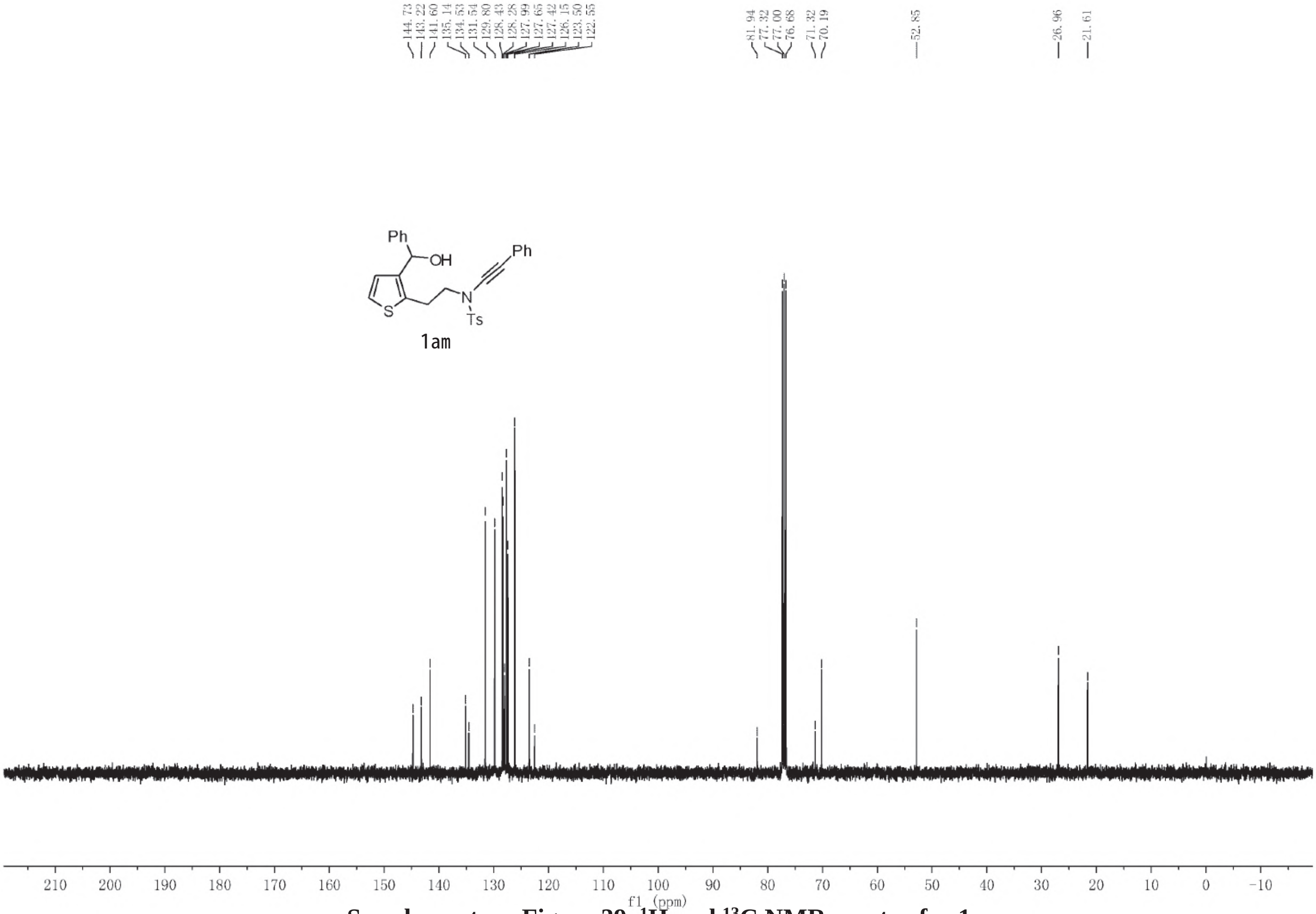

Supplementary Figure 39. ^{1}H and ^{13}C NMR spectra for 1am

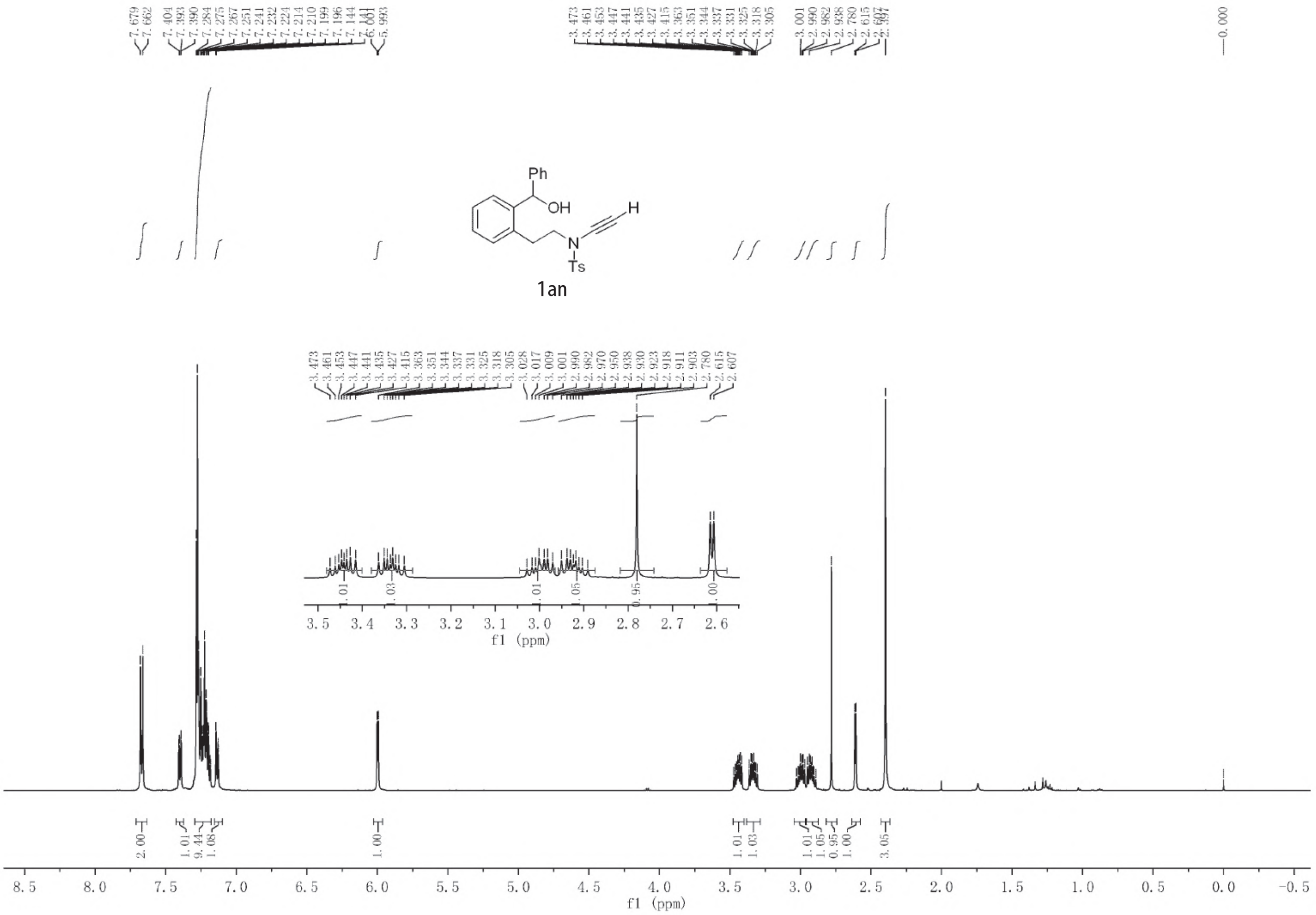

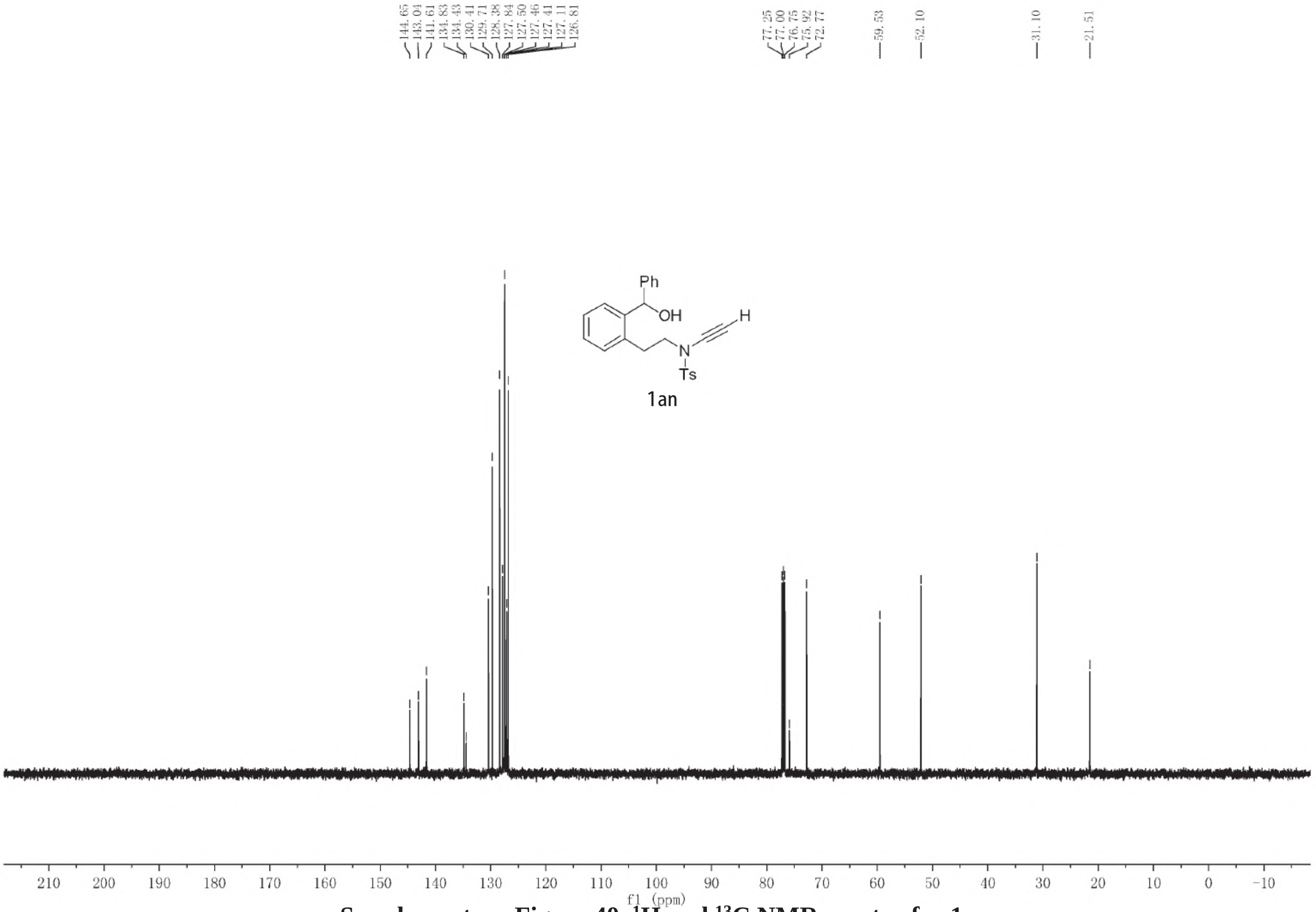

Supplementary Figure 40. ¹H and ¹³C NMR spectra for 1an

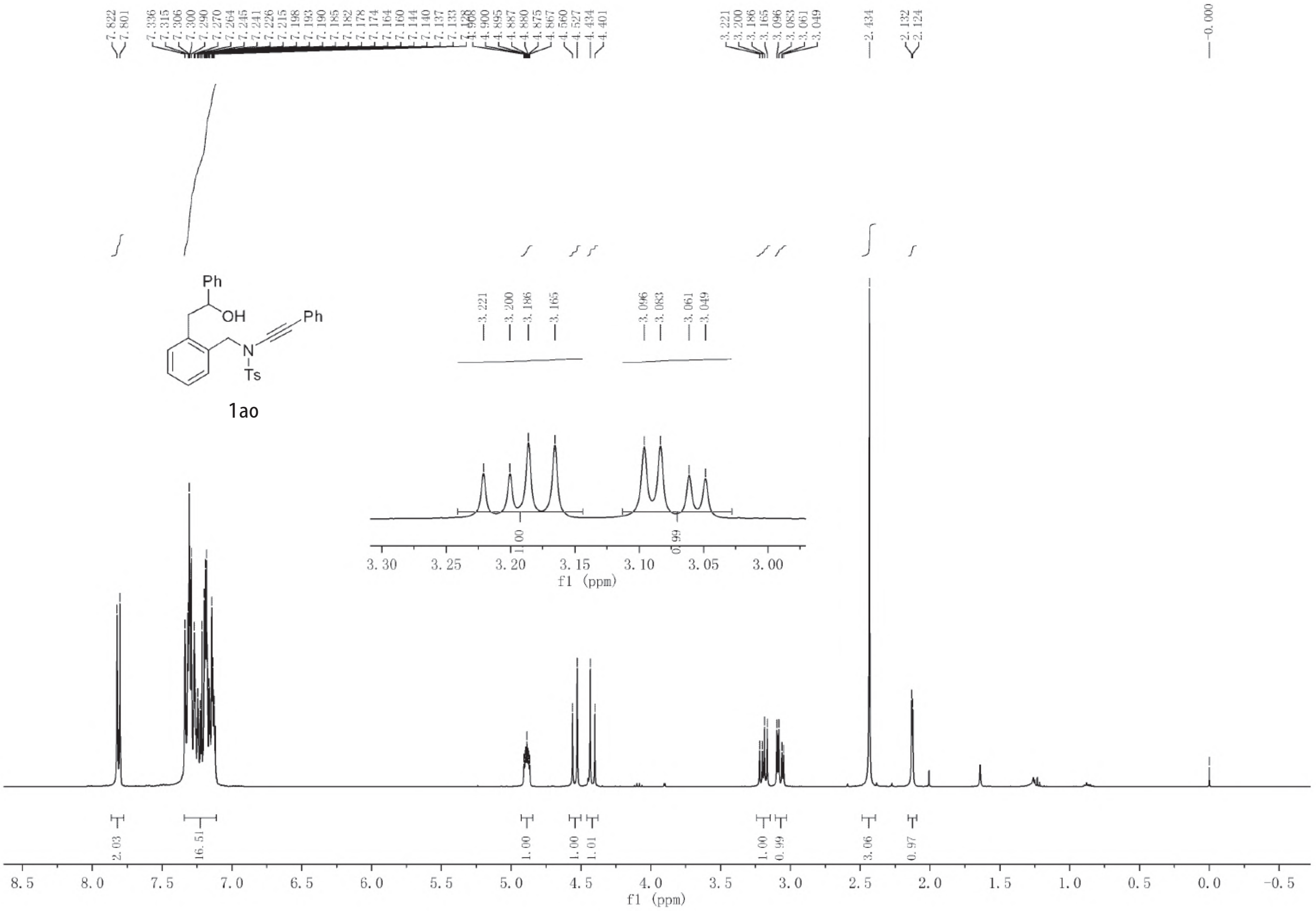

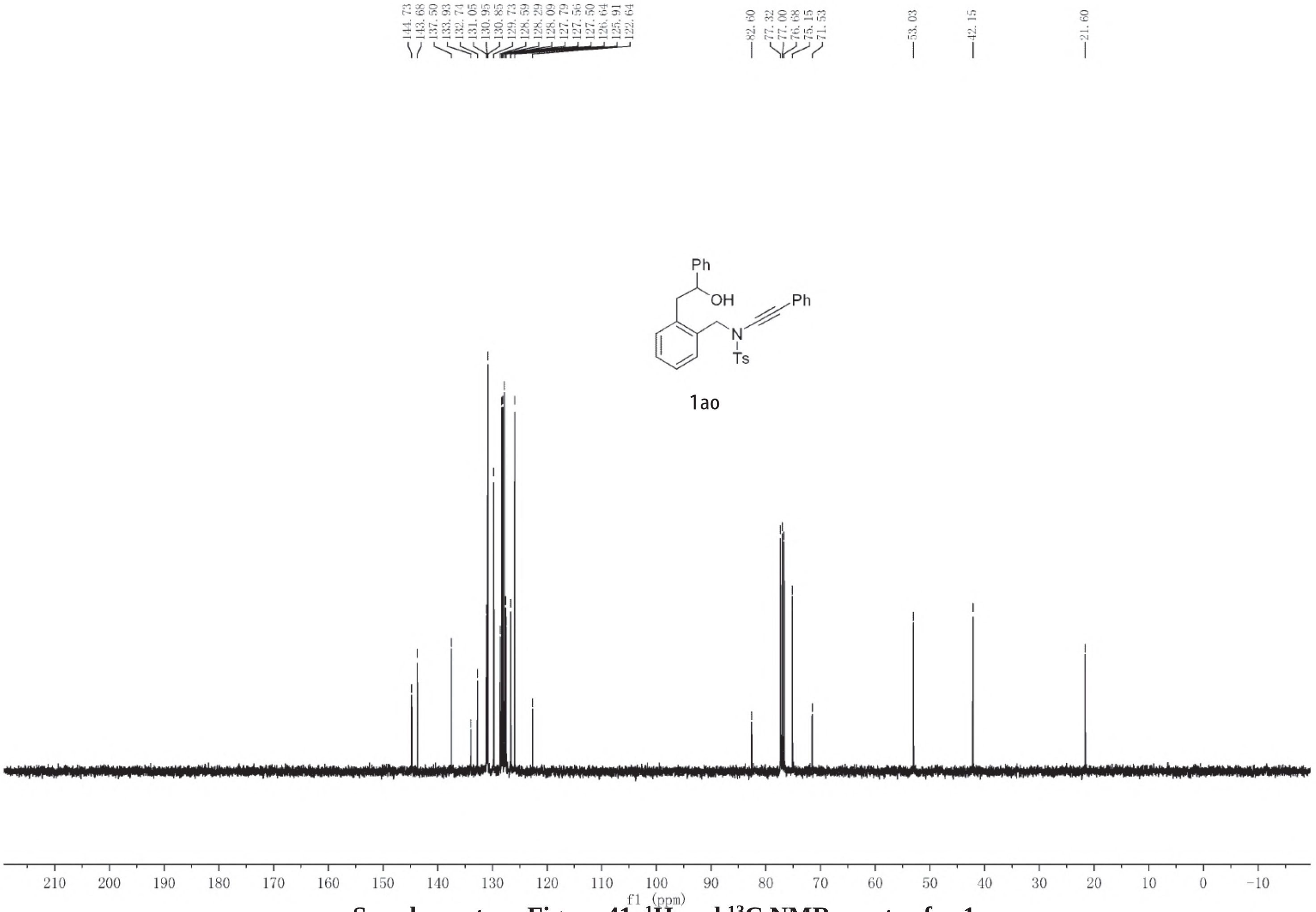

Supplementary Figure 41. ^{1}H and ^{13}C NMR spectra for 1ao

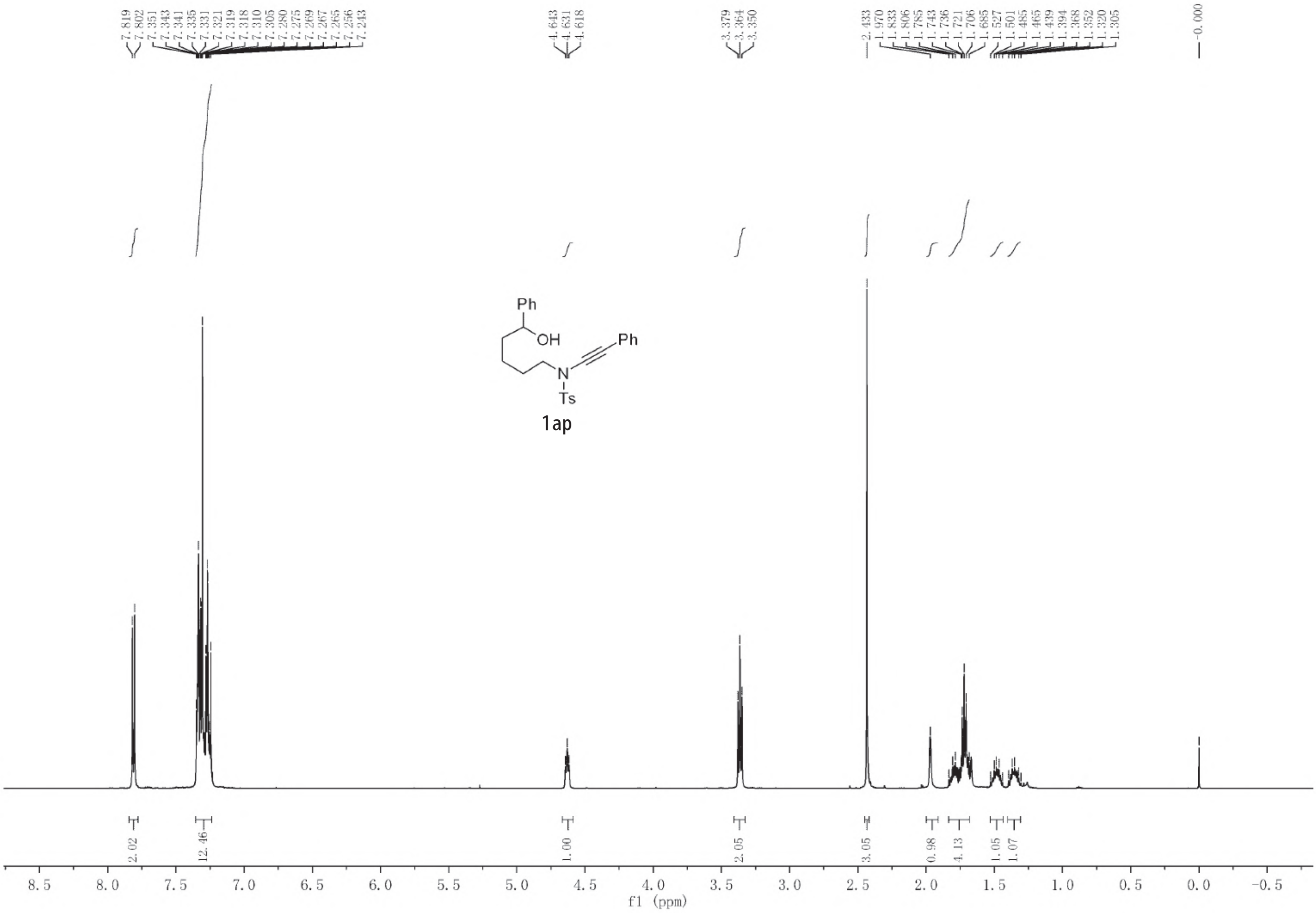

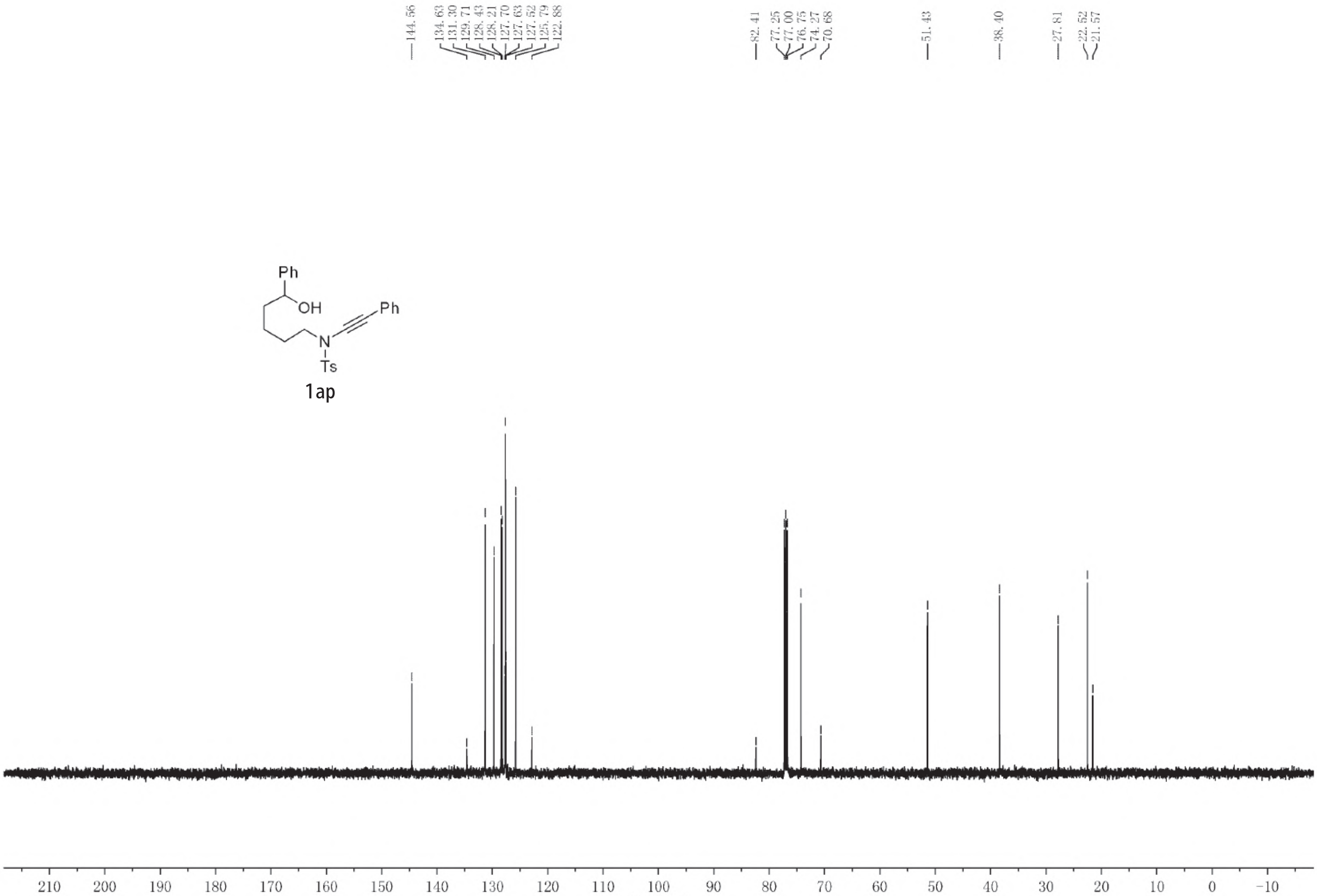

Supplementary Figure 42. ^{1}H and ^{13}C NMR spectra for 1ap

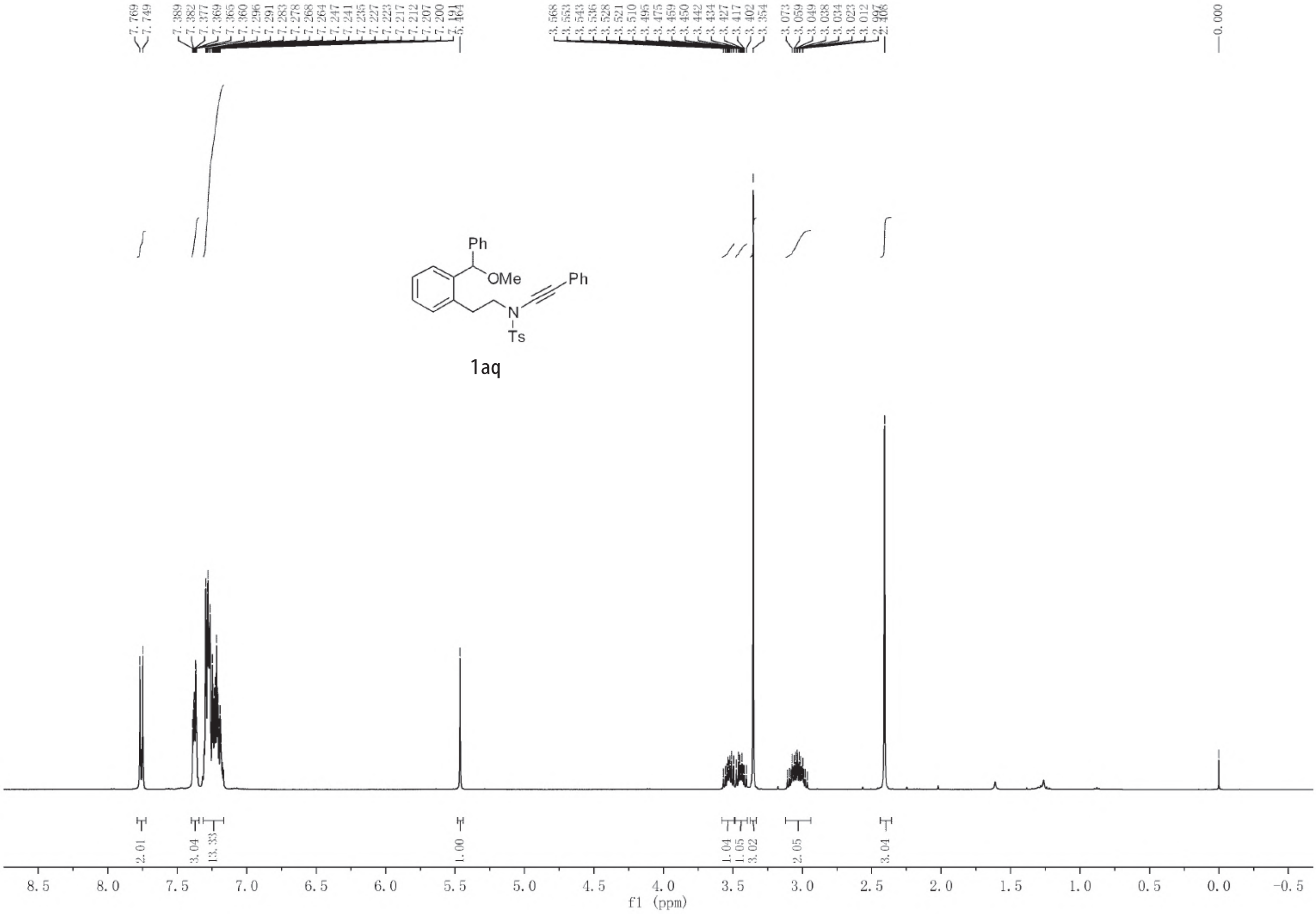

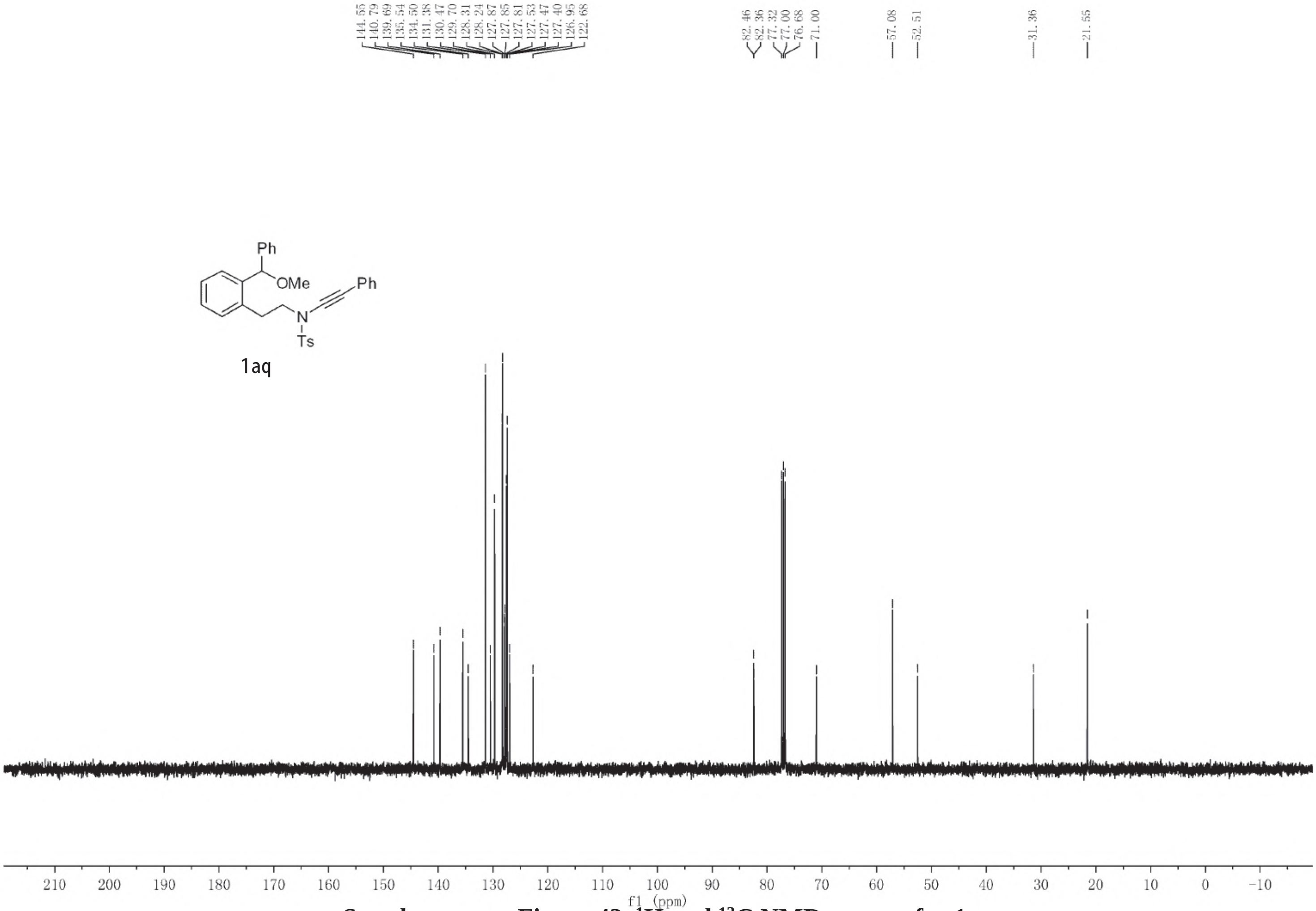

Supplementary Figure 43. ^{1}H and ^{13}C NMR spectra for 1aq

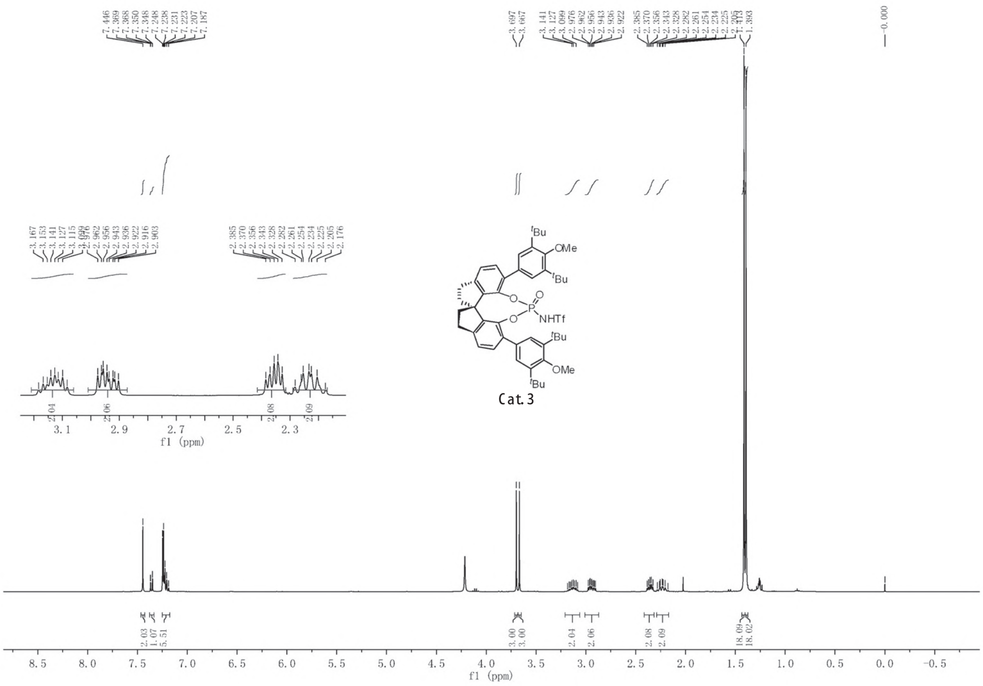

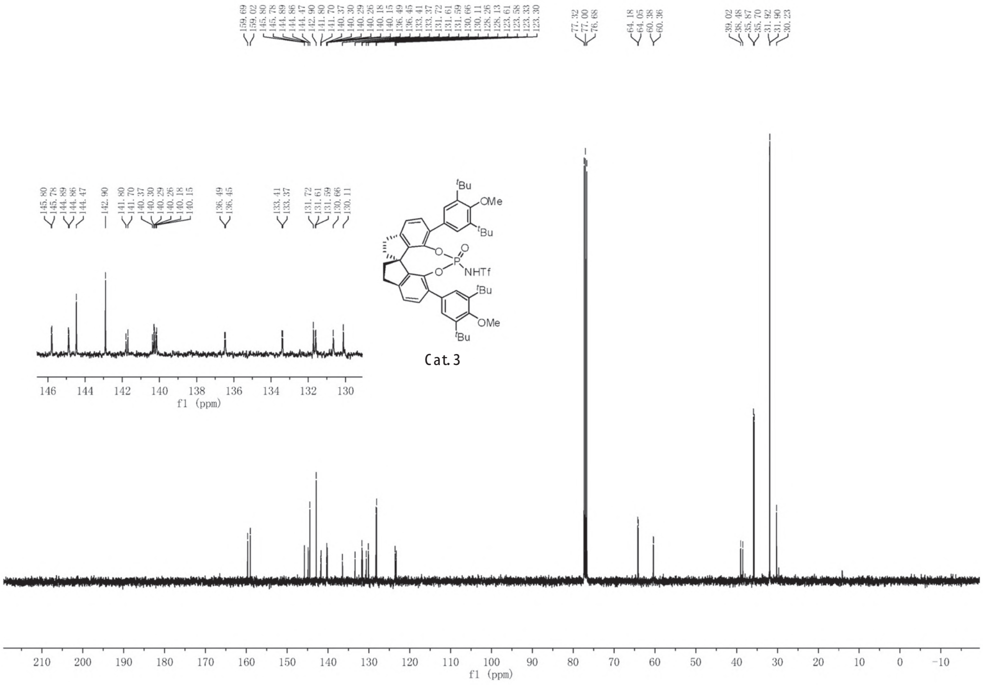

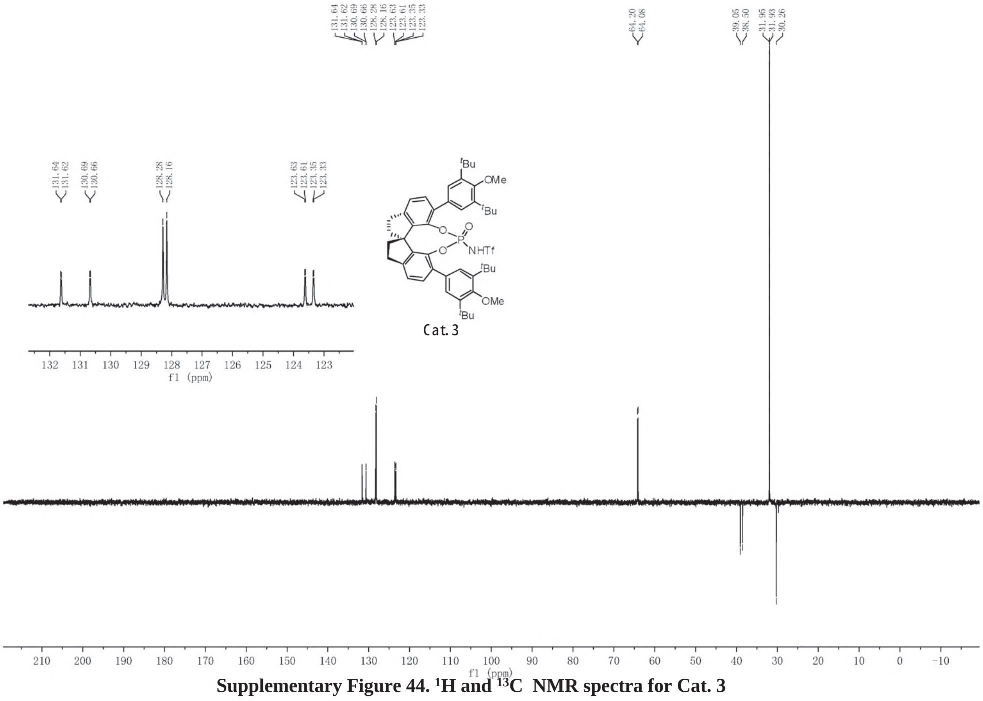

Supplementary Figure 44. ^{1}H and ^{13}C NMR spectra for Cat. 3

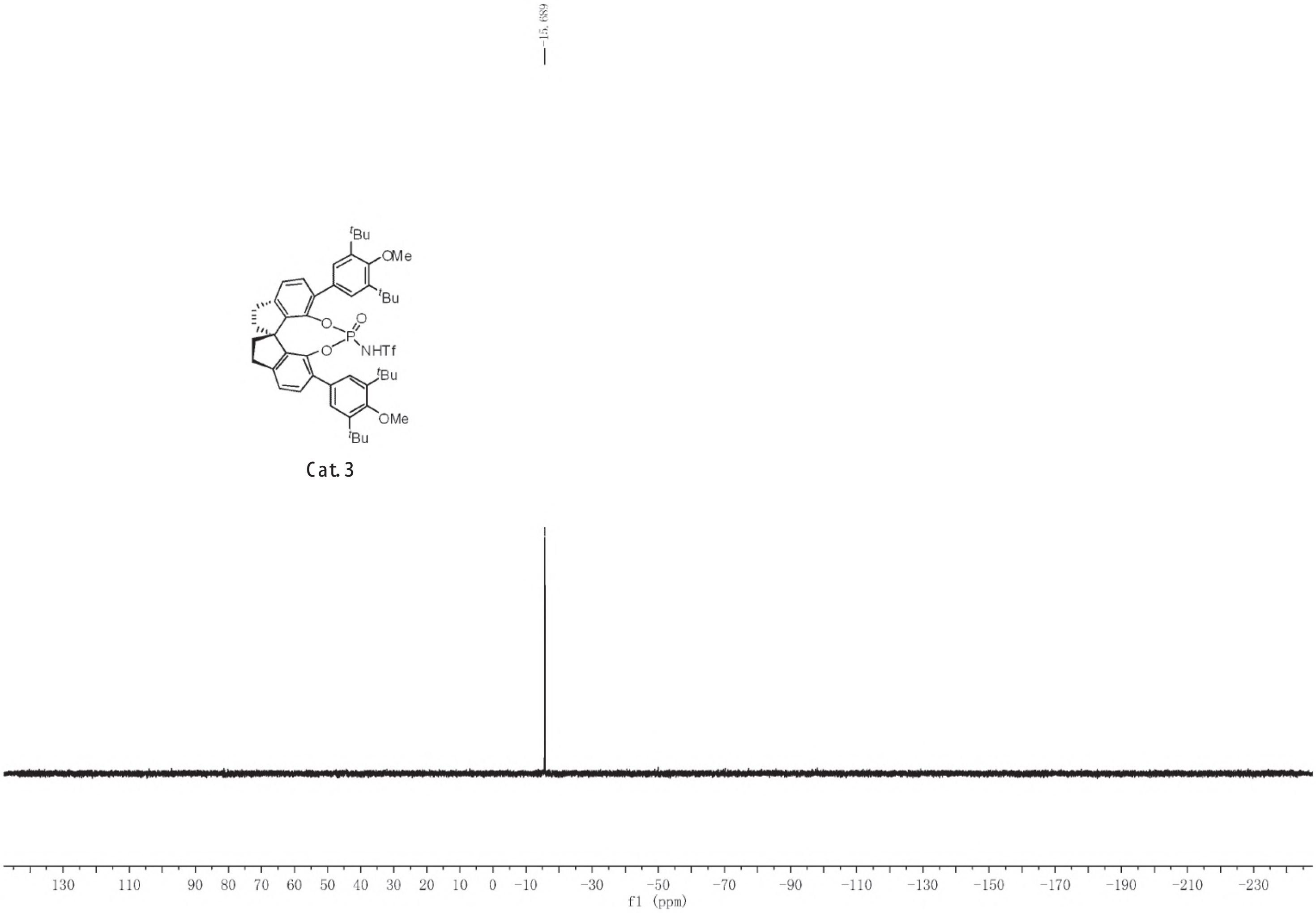

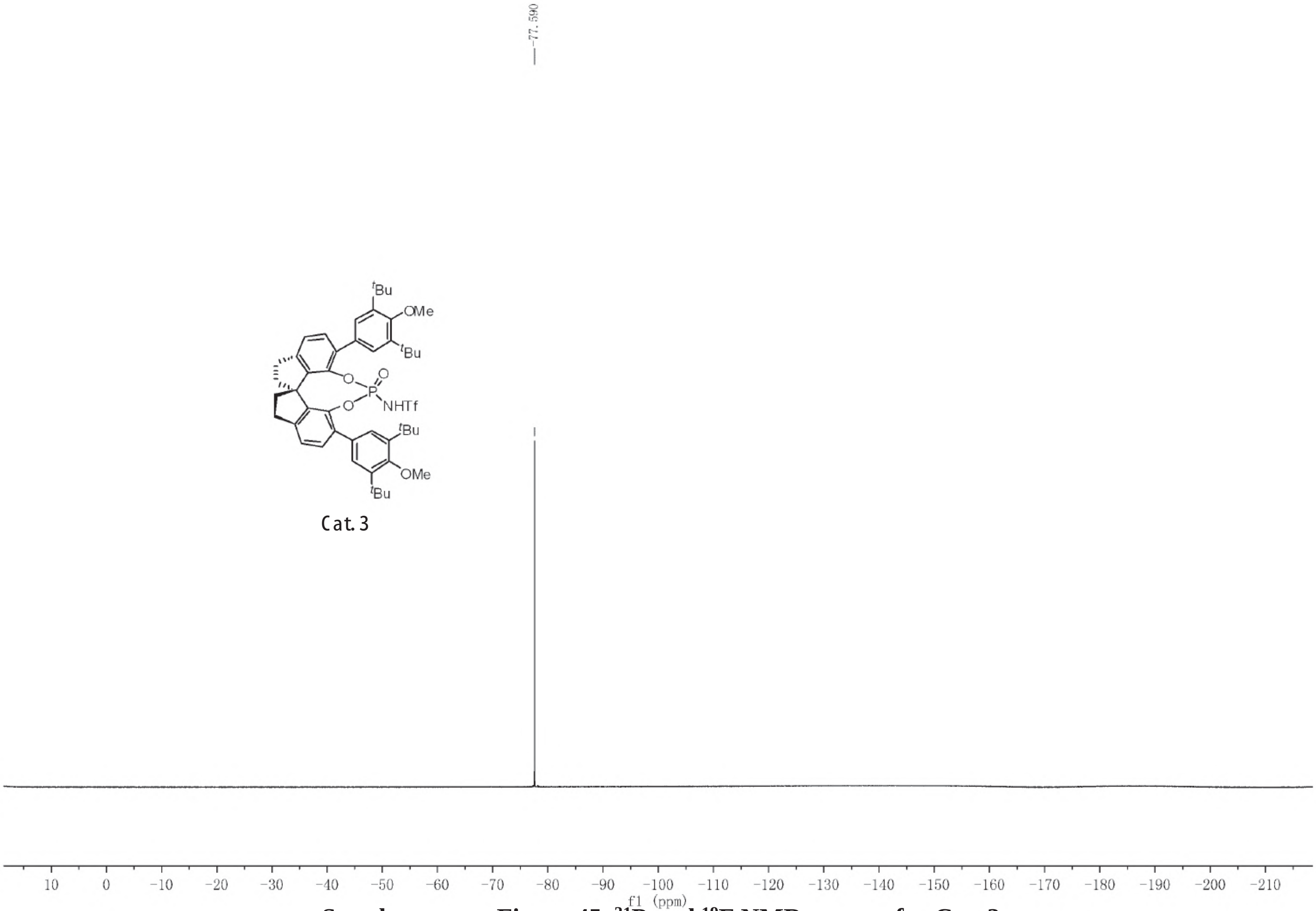

Supplementary Figure 45. ^{31}P and ^{19}F NMR spectra for Cat. 3

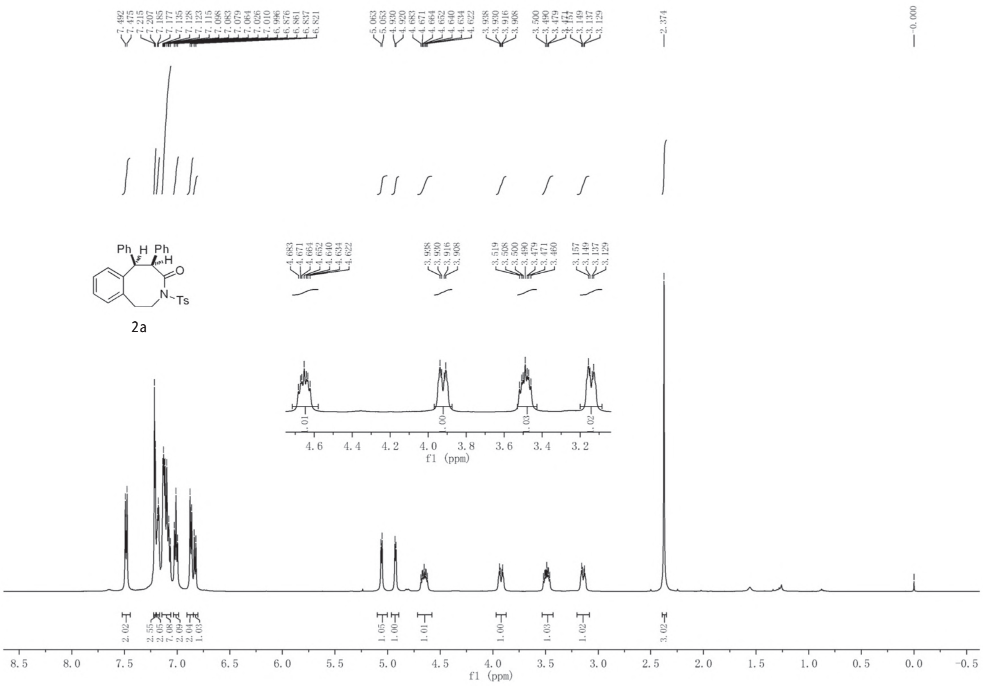

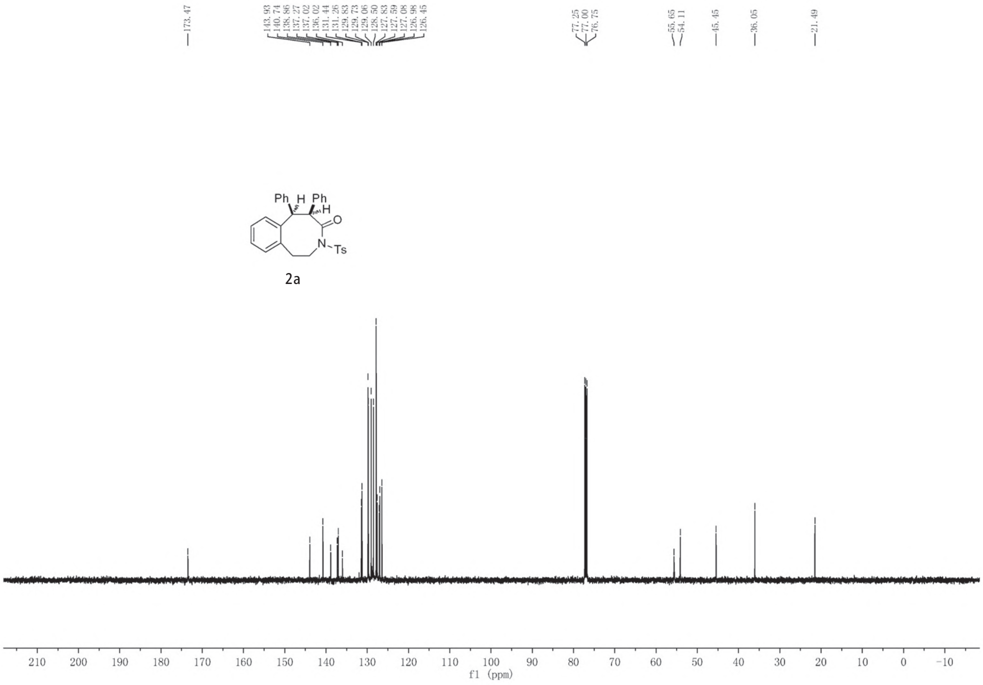

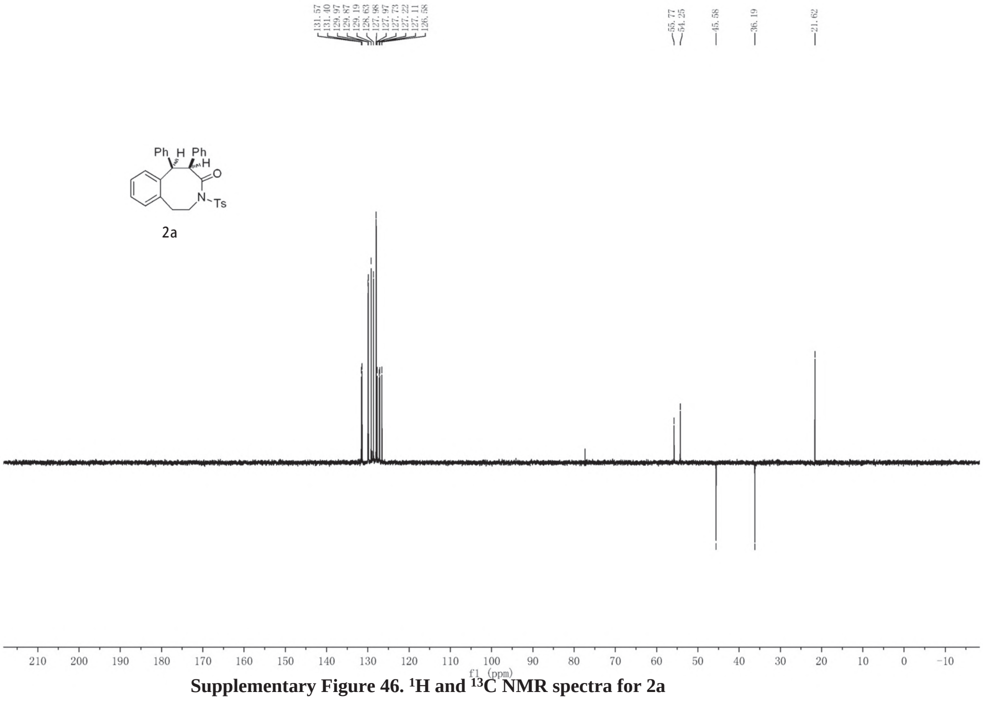

Supplementary Figure 46. ^{1}H and ^{13}C NMR spectra for 2a

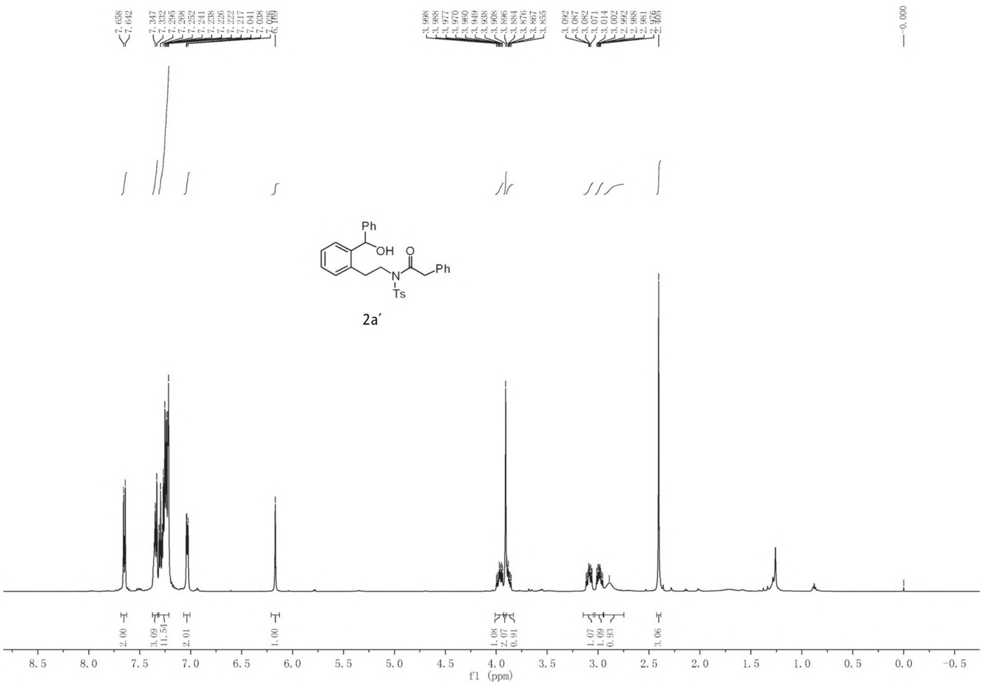

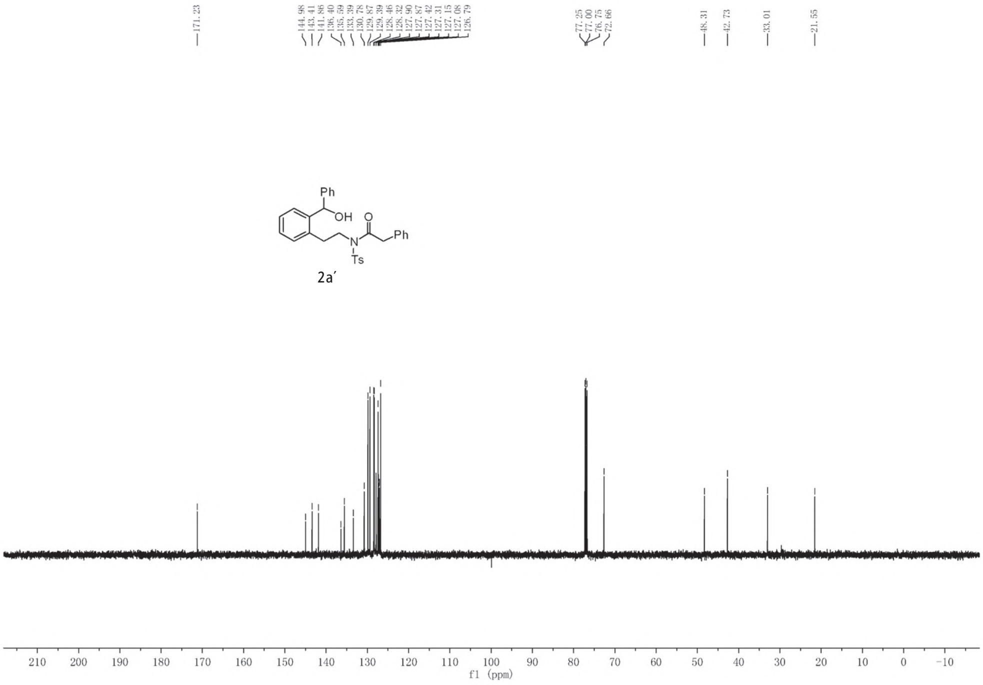

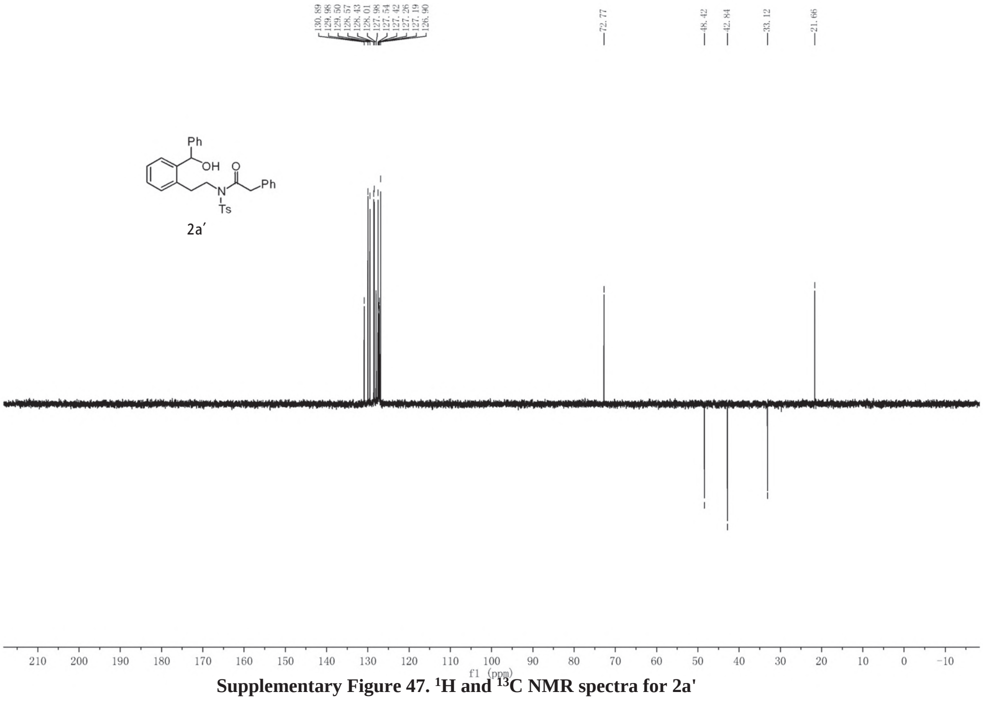

Supplementary Figure 47. ^{1}H and ^{13}C NMR spectra for 2a'

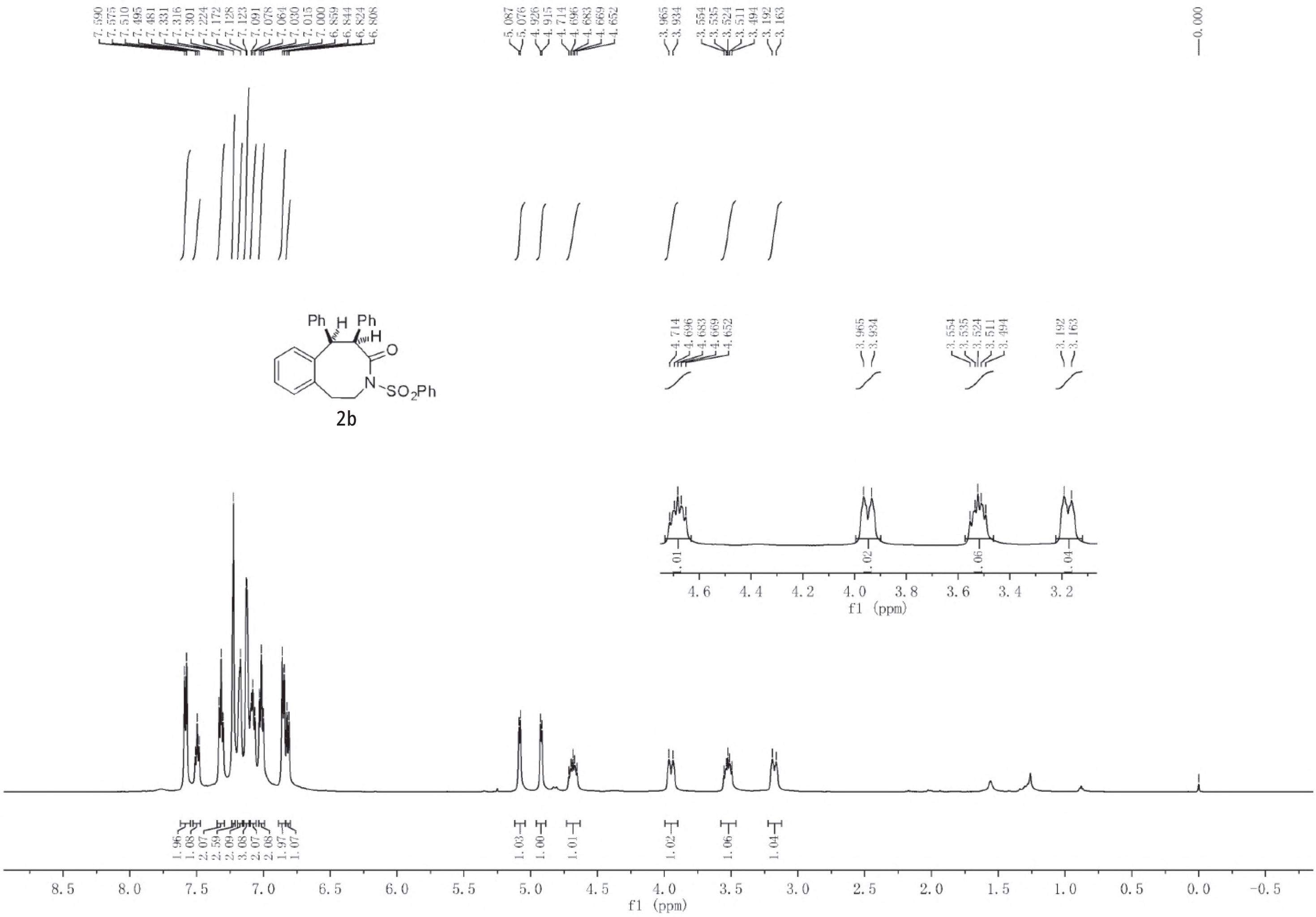

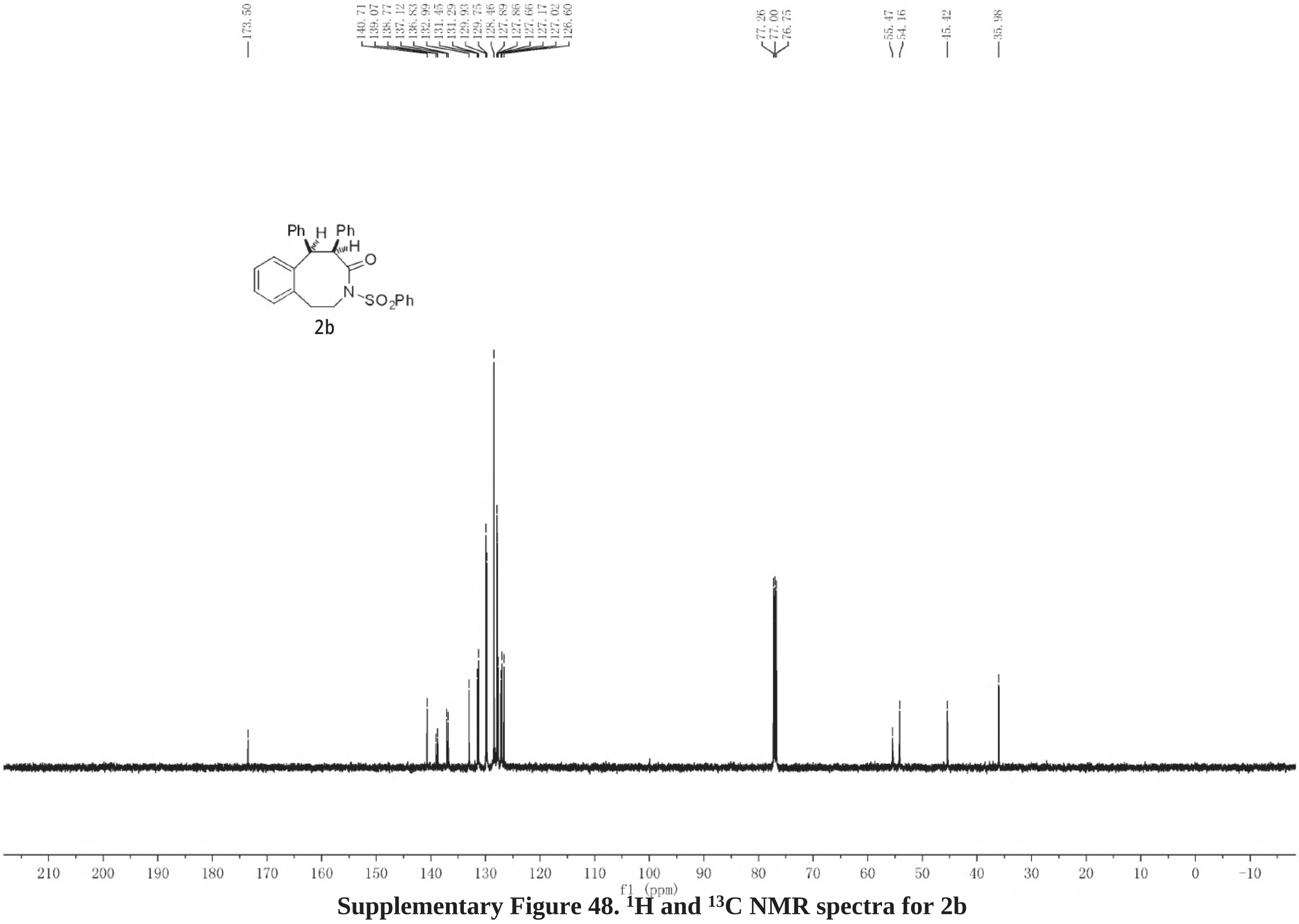

Supplementary Figure 48. ^{1}H and ^{13}C NMR spectra for 2b

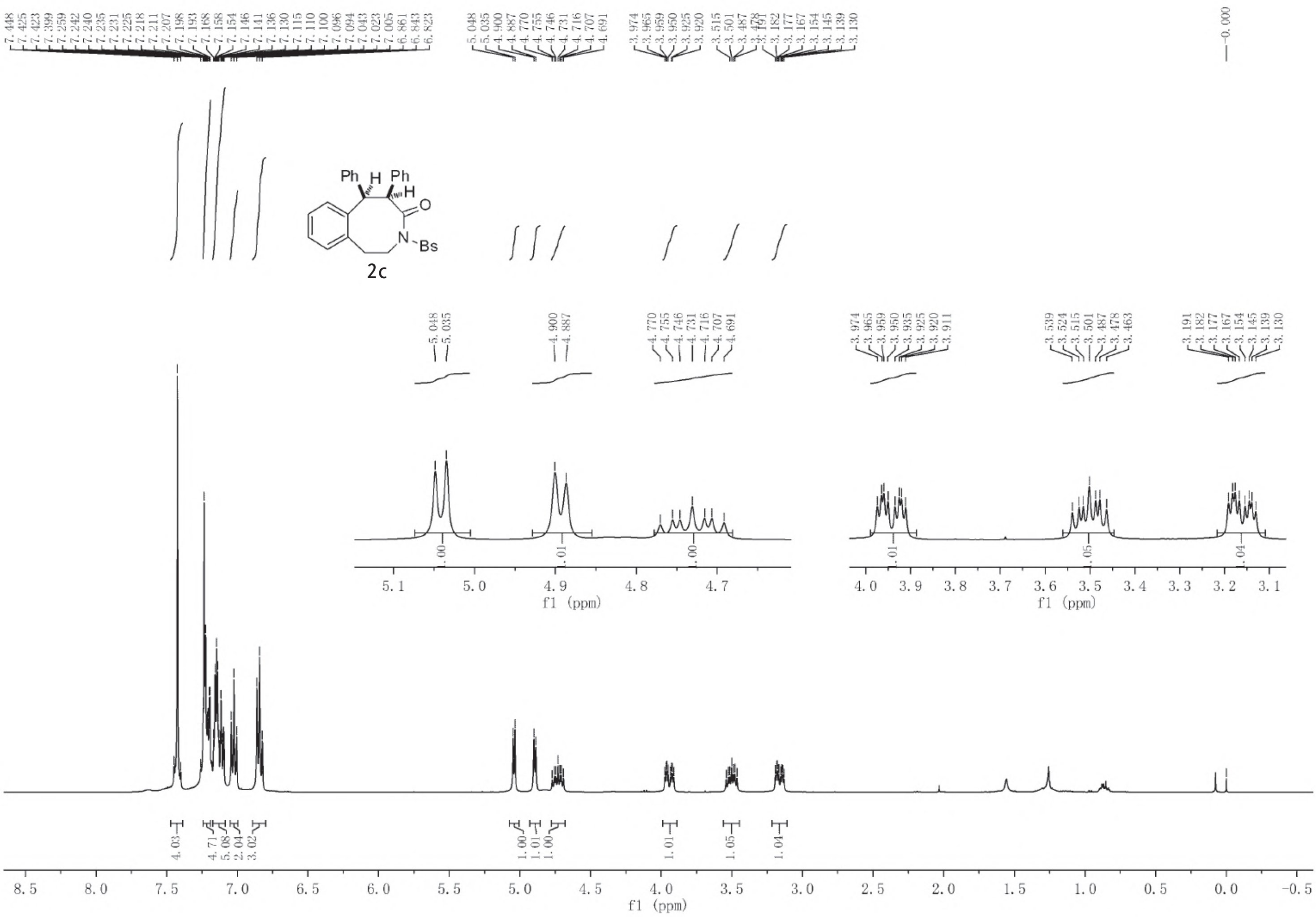

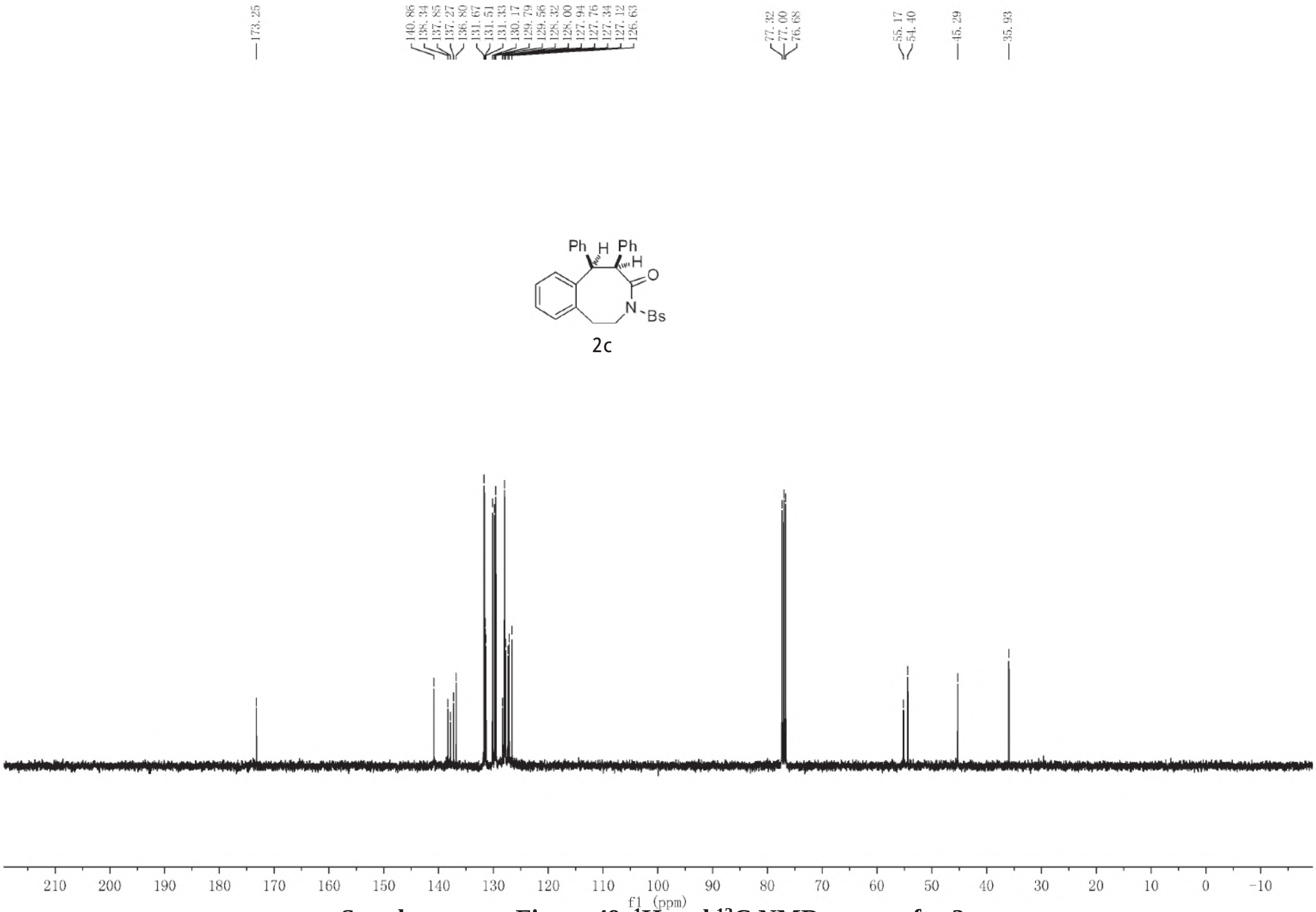

Supplementary Figure 49. ^{1}H and ^{13}C NMR spectra for 2c

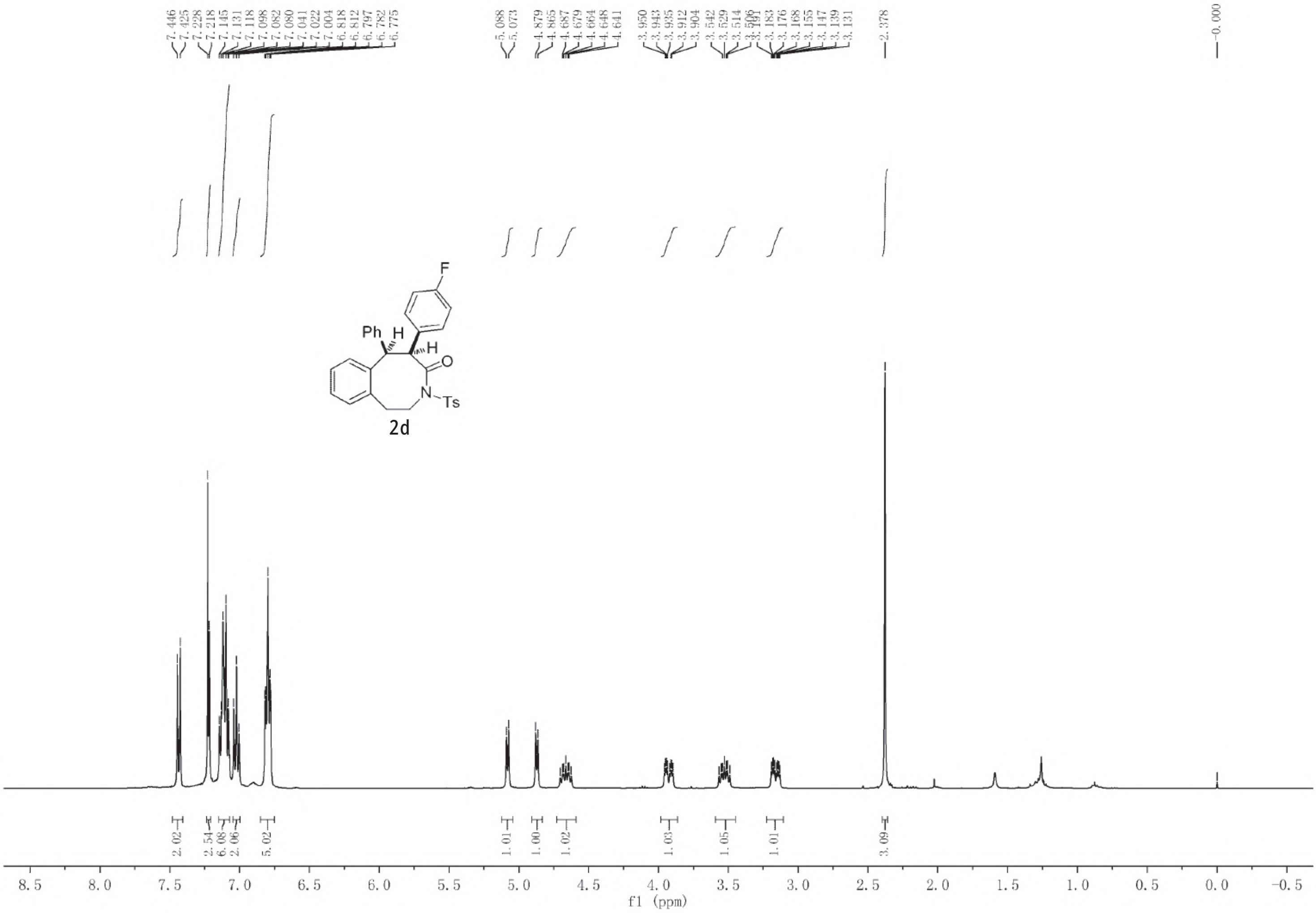

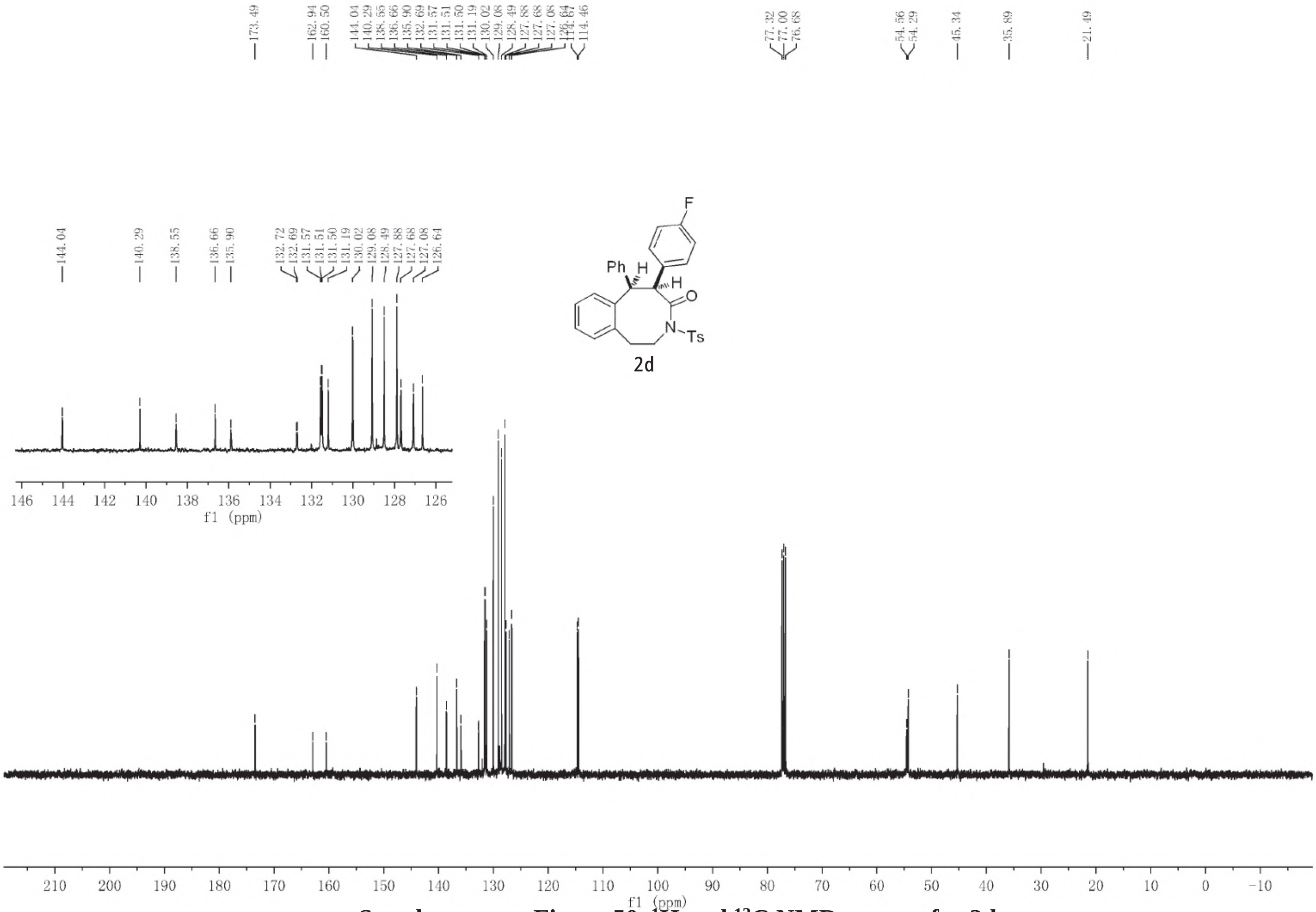

Supplementary Figure 50. ^{1}H and ^{13}C NMR spectra for 2d

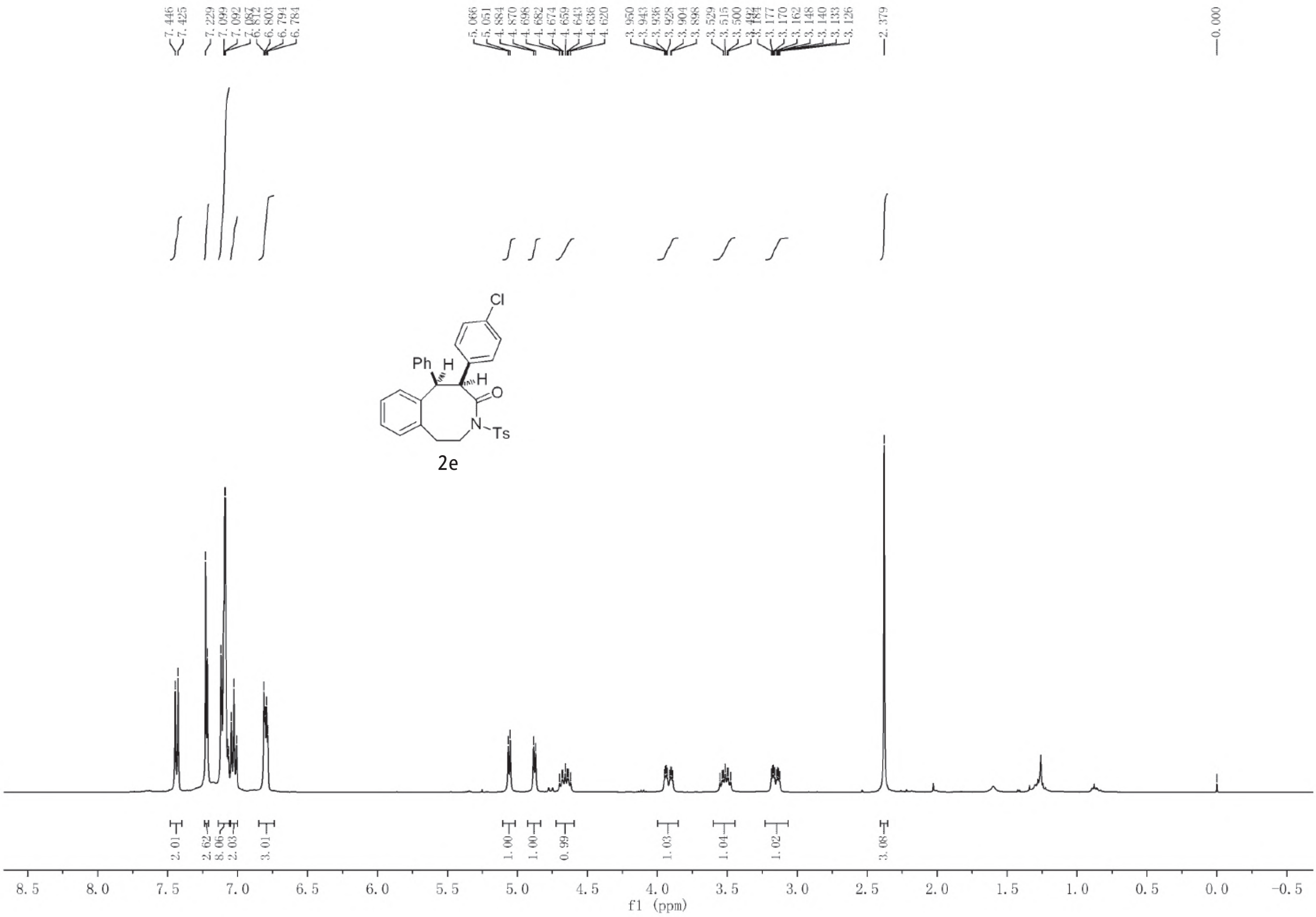

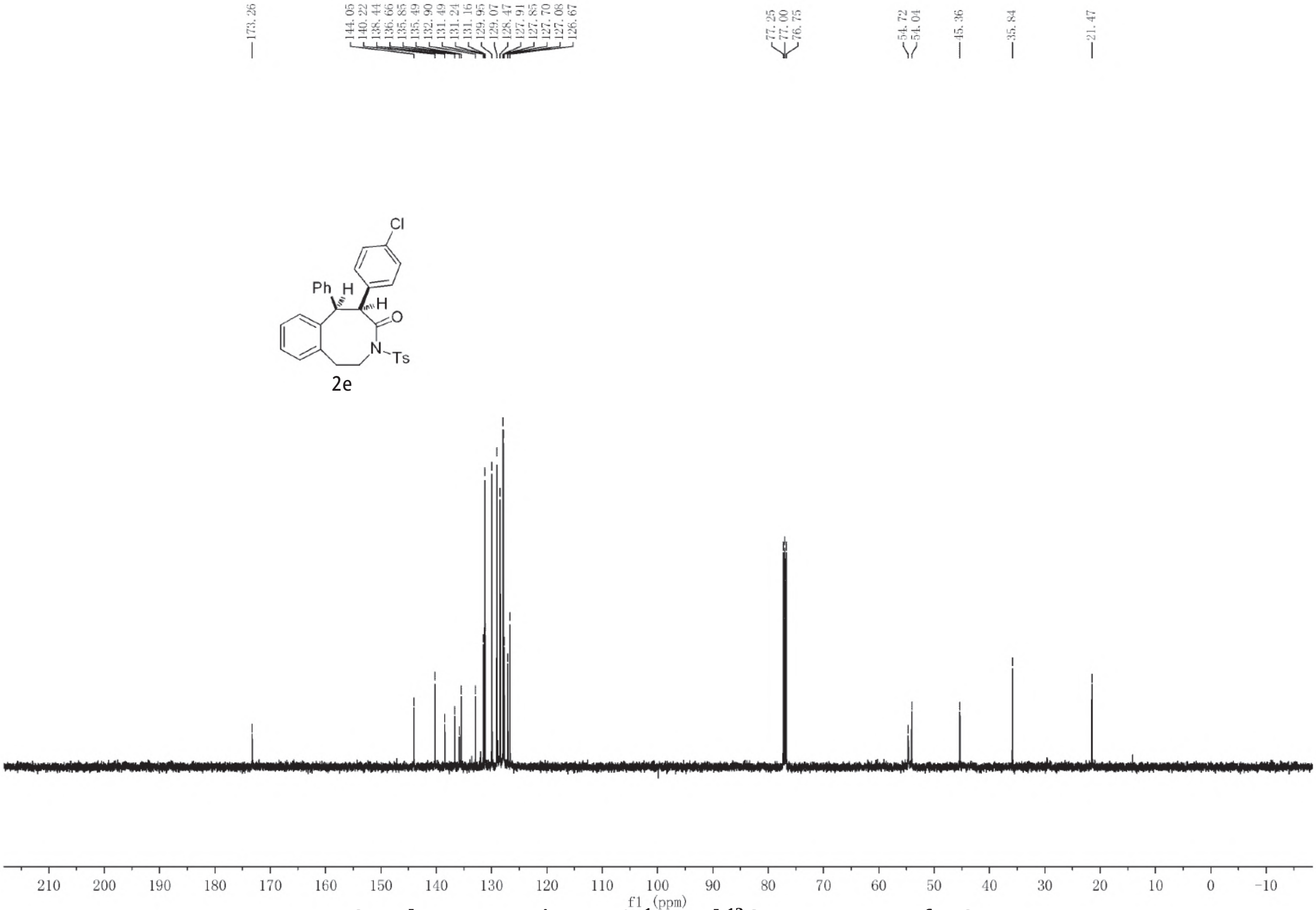

Supplementary Figure 51. ^{1}H and ^{13}C NMR spectra for 2e

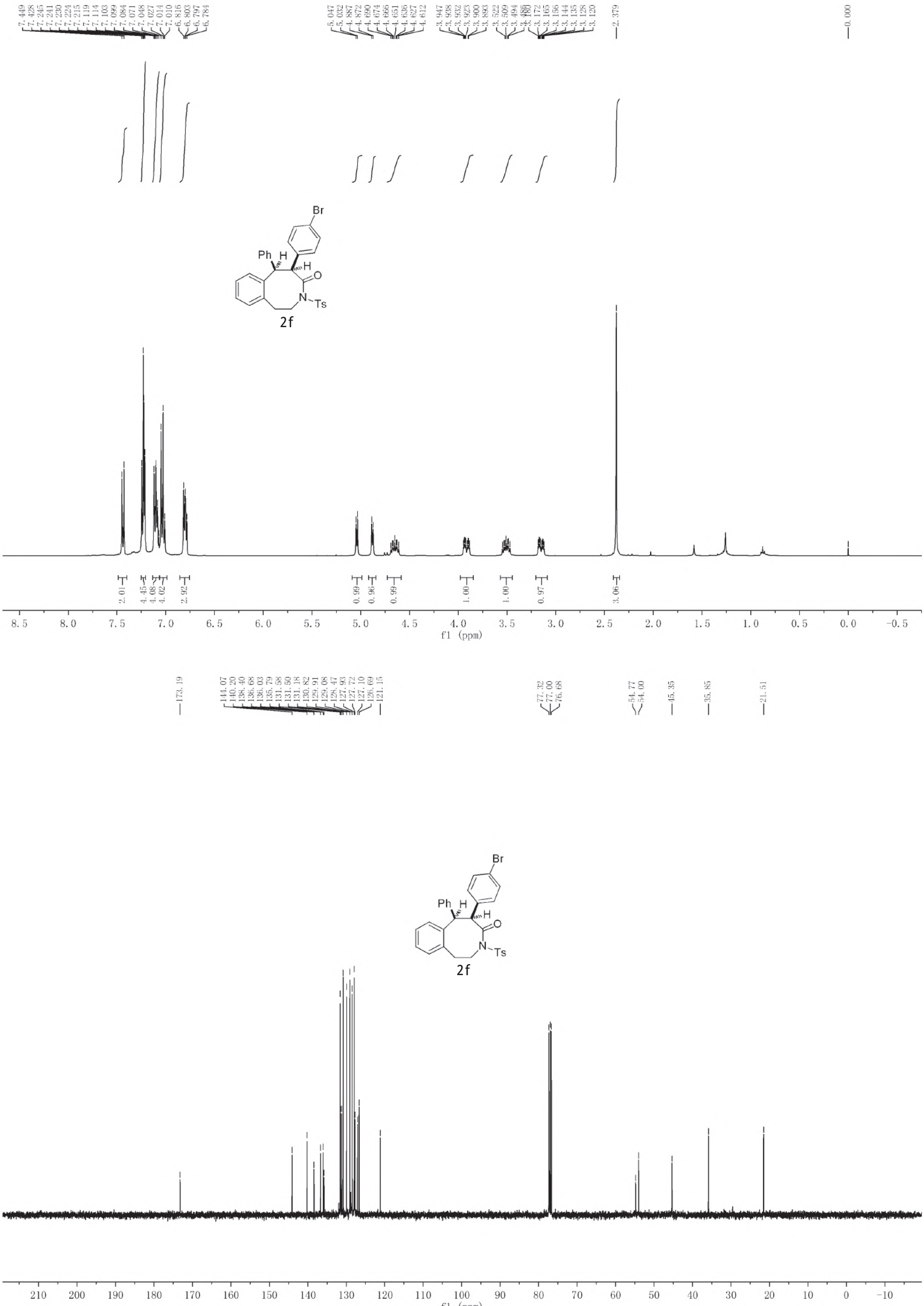

Supplementary Figure 52. ^{1}H and ^{13}C NMR spectra for 2f

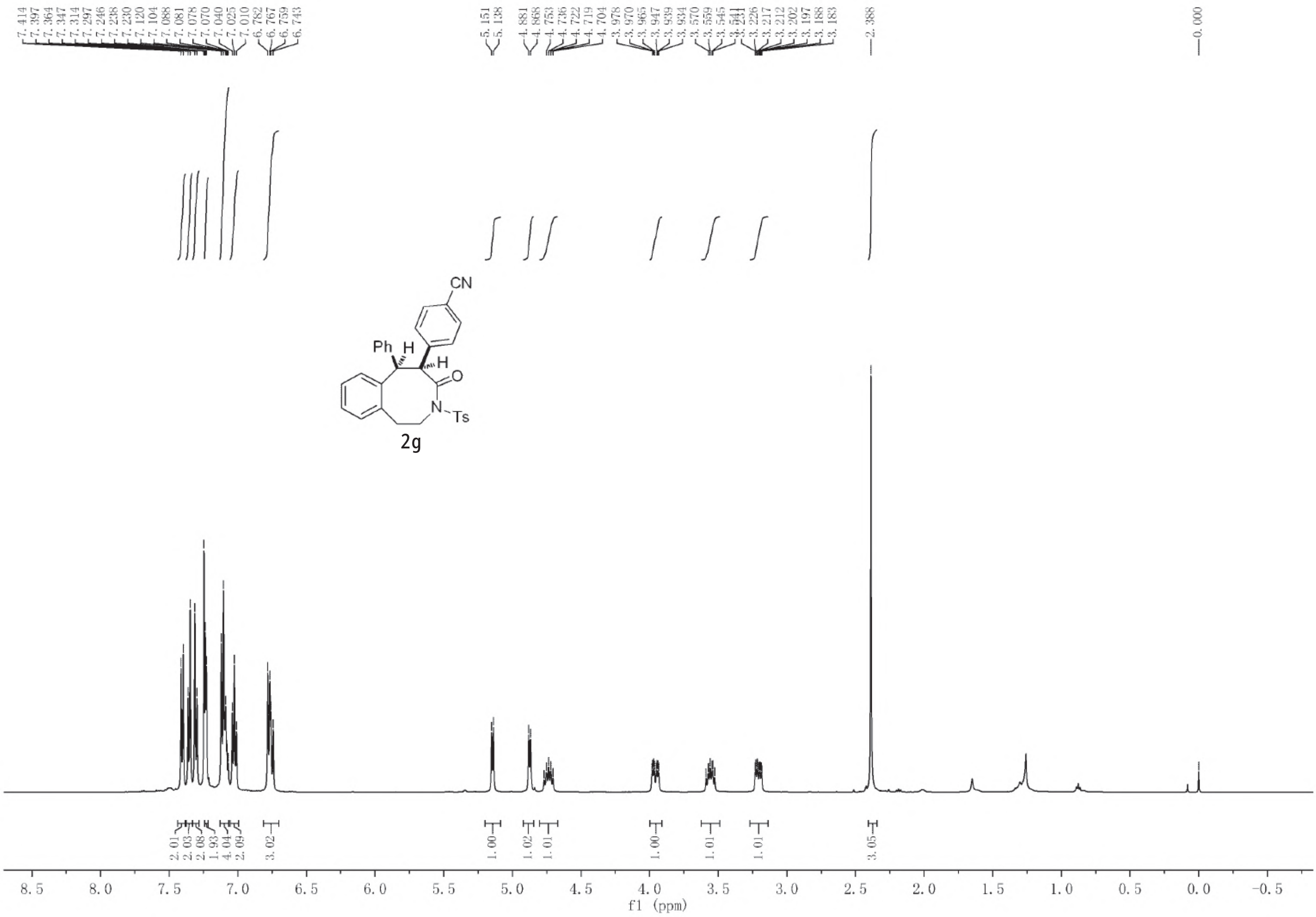

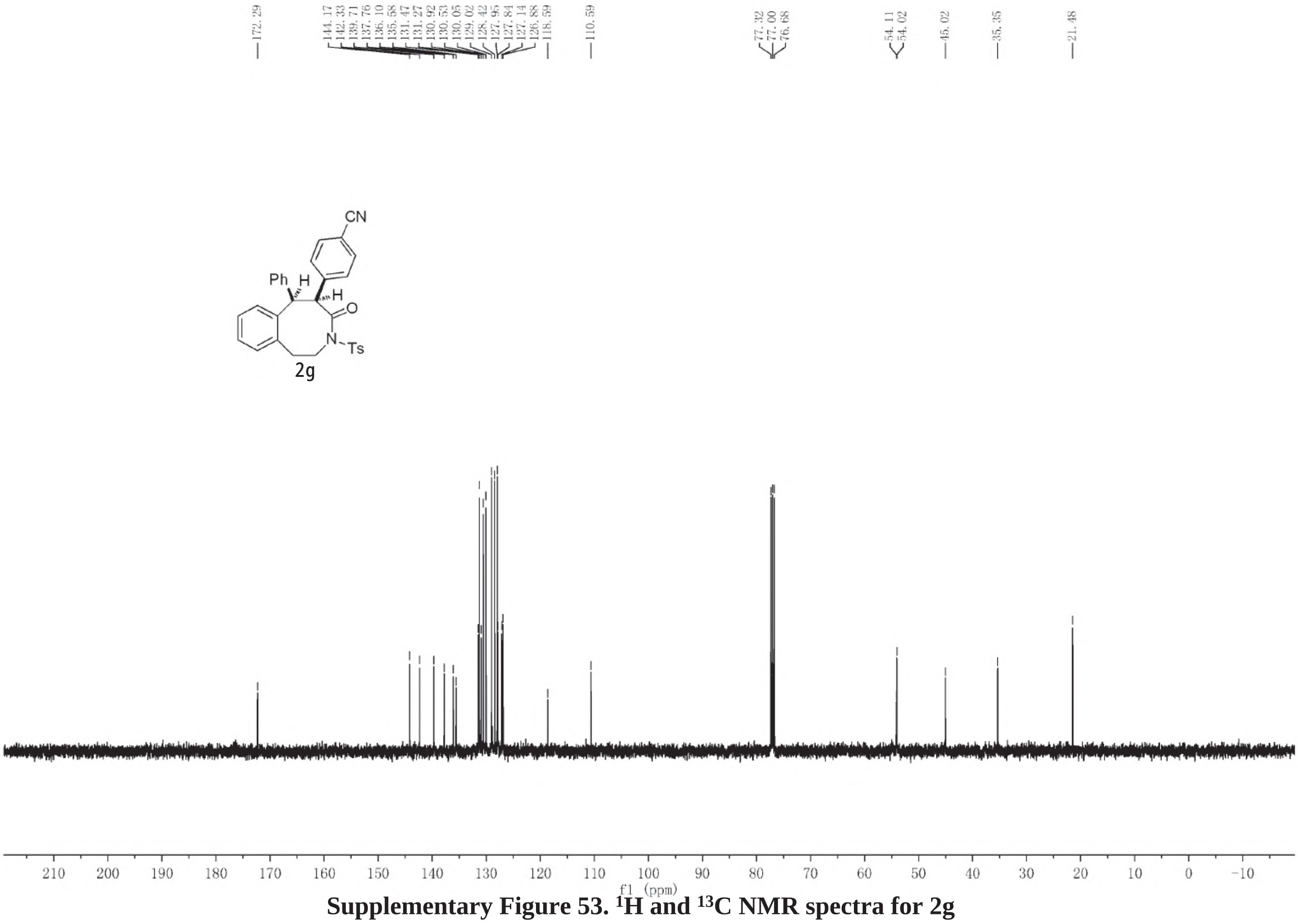

Supplementary Figure 53. ^{1}H and ^{13}C NMR spectra for 2g

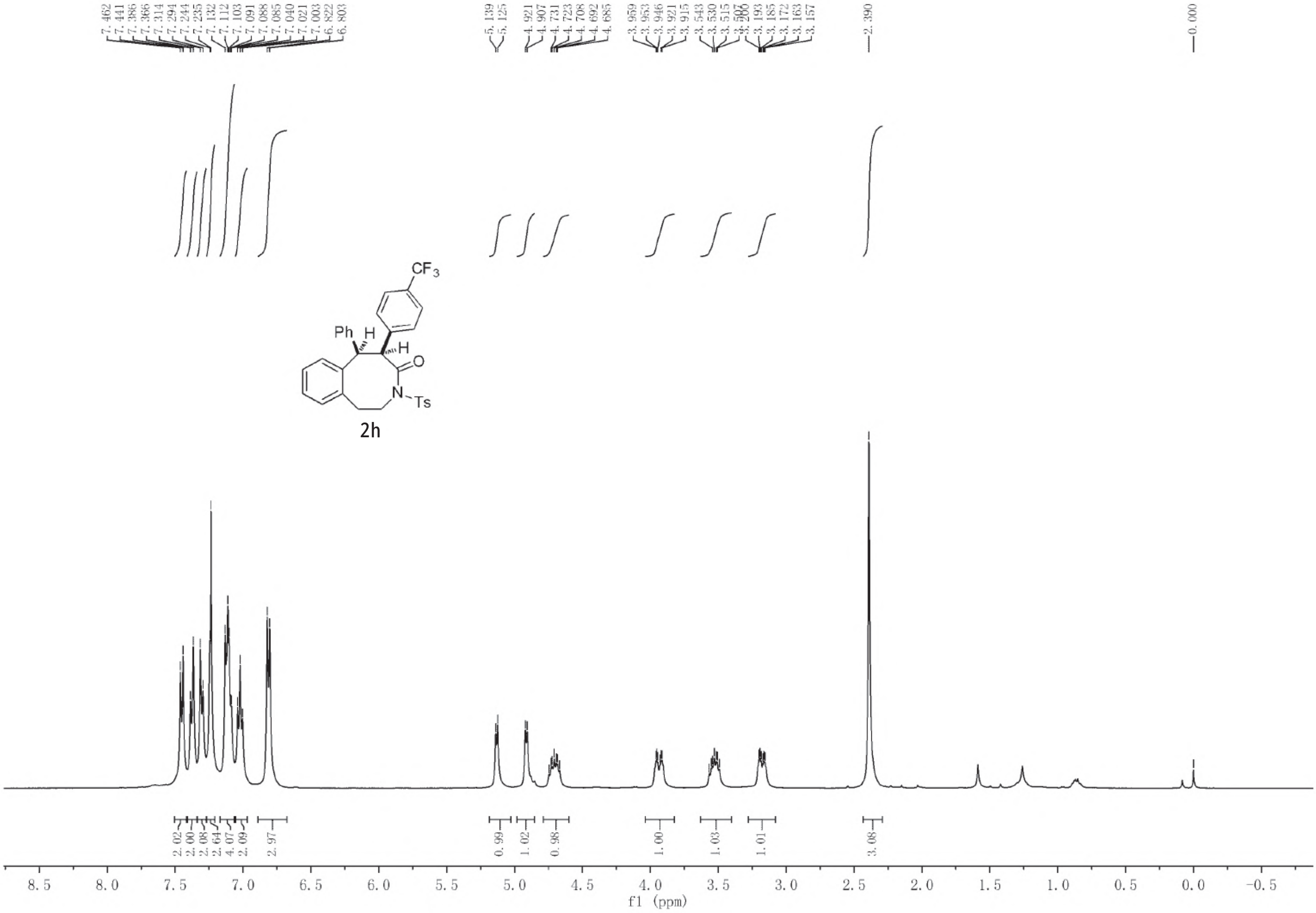

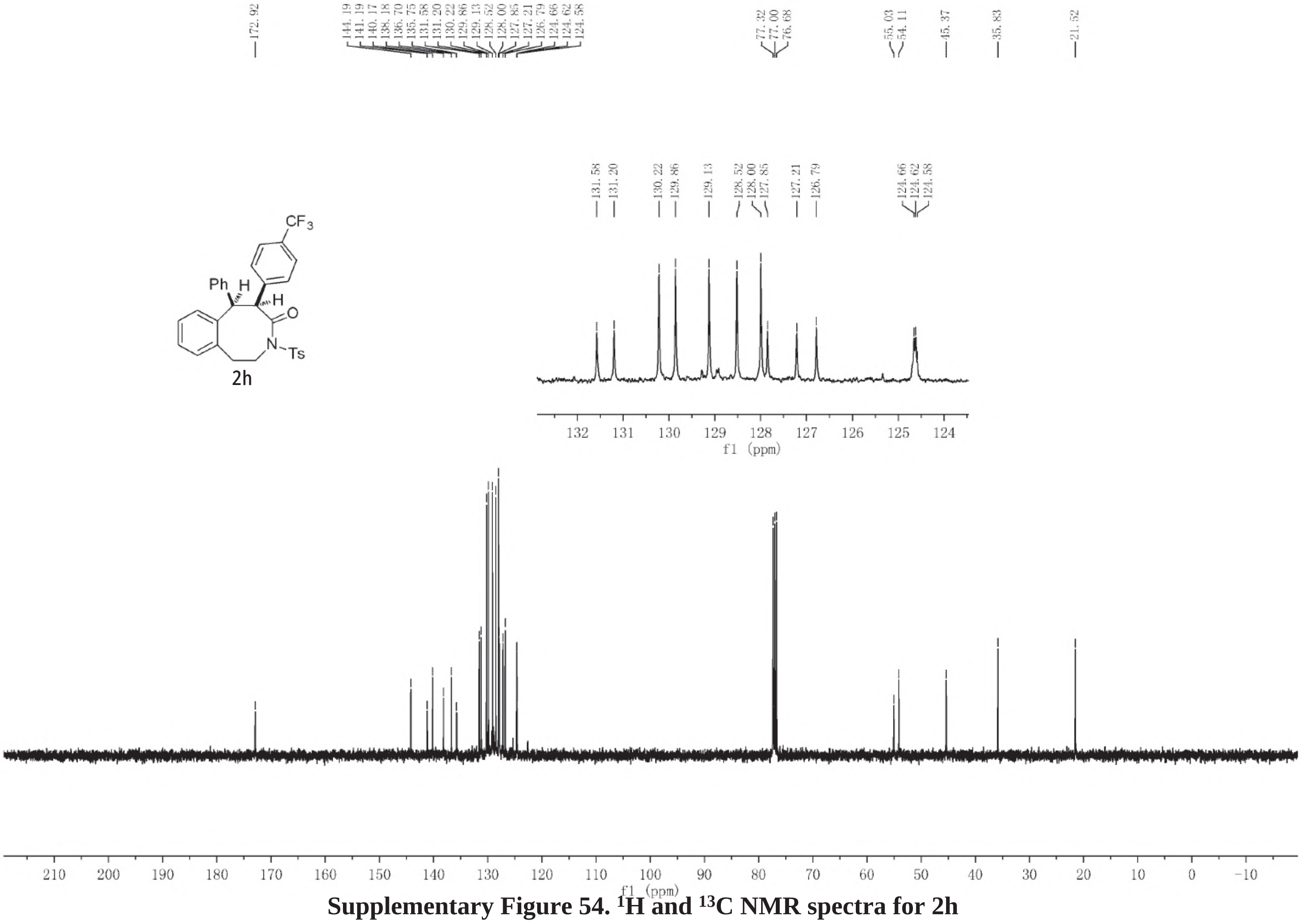

Supplementary Figure 54. ^{1}H and ^{13}C NMR spectra for 2h

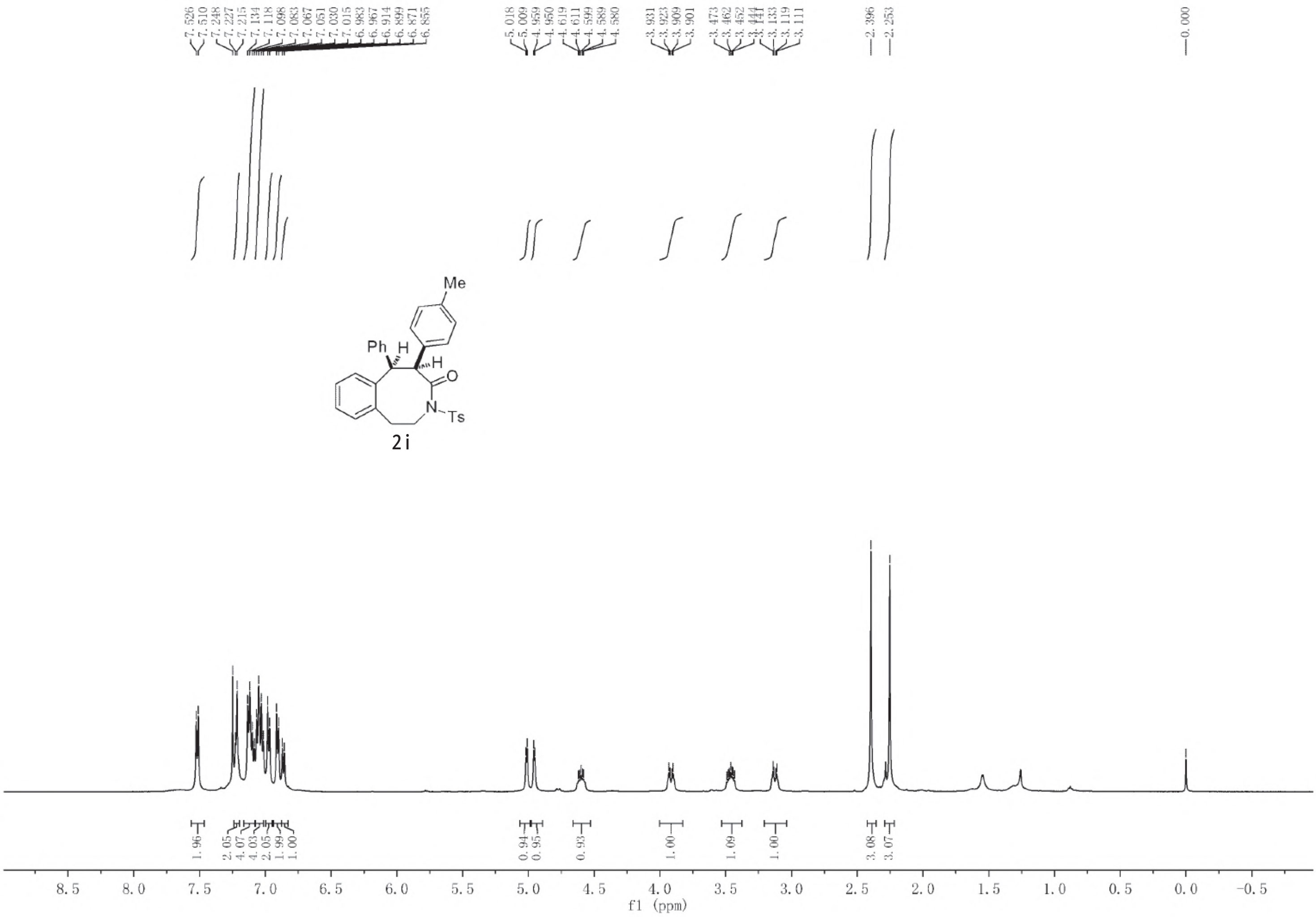

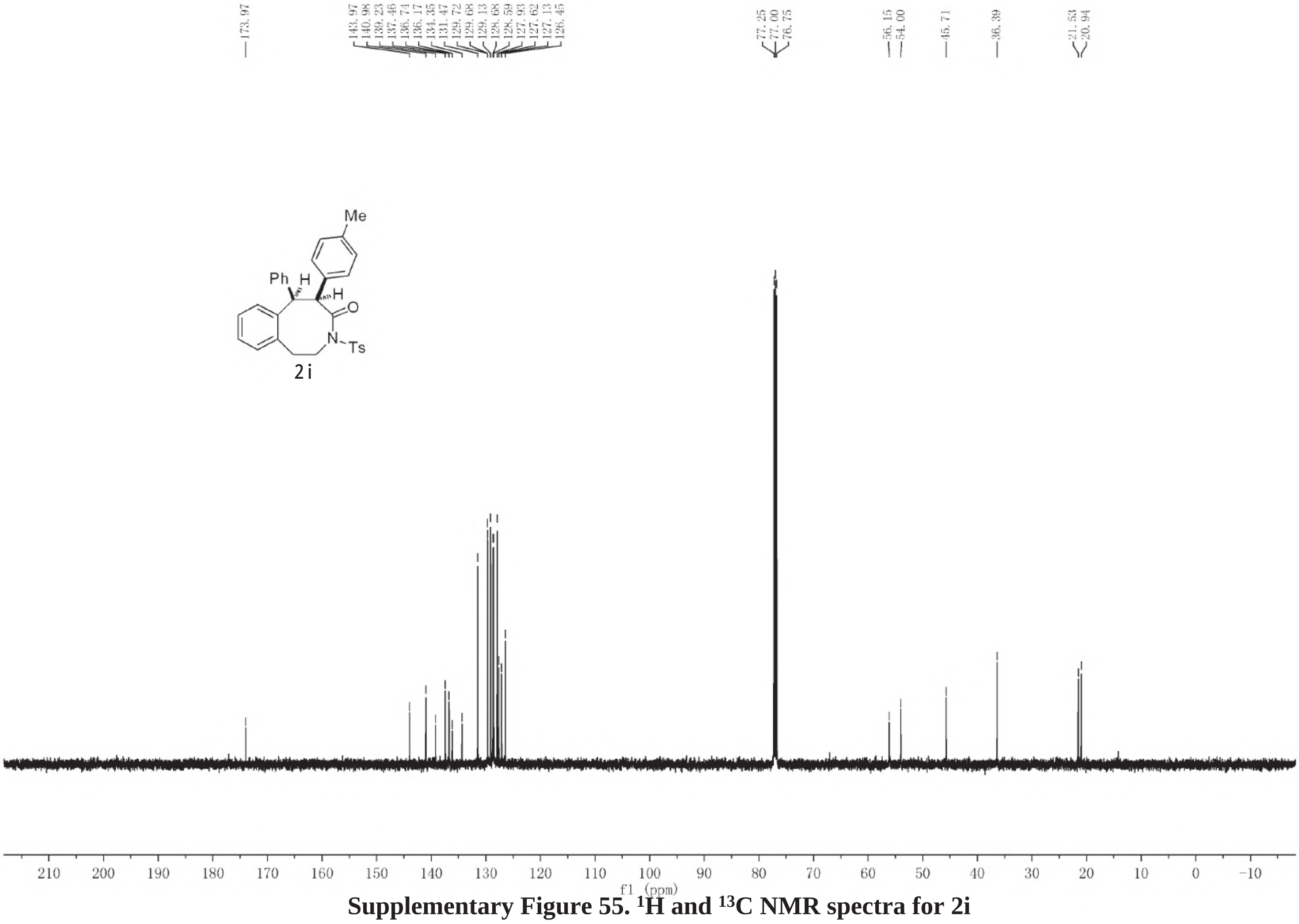

Supplementary Figure 55. ^{1}H and ^{13}C NMR spectra for 2i

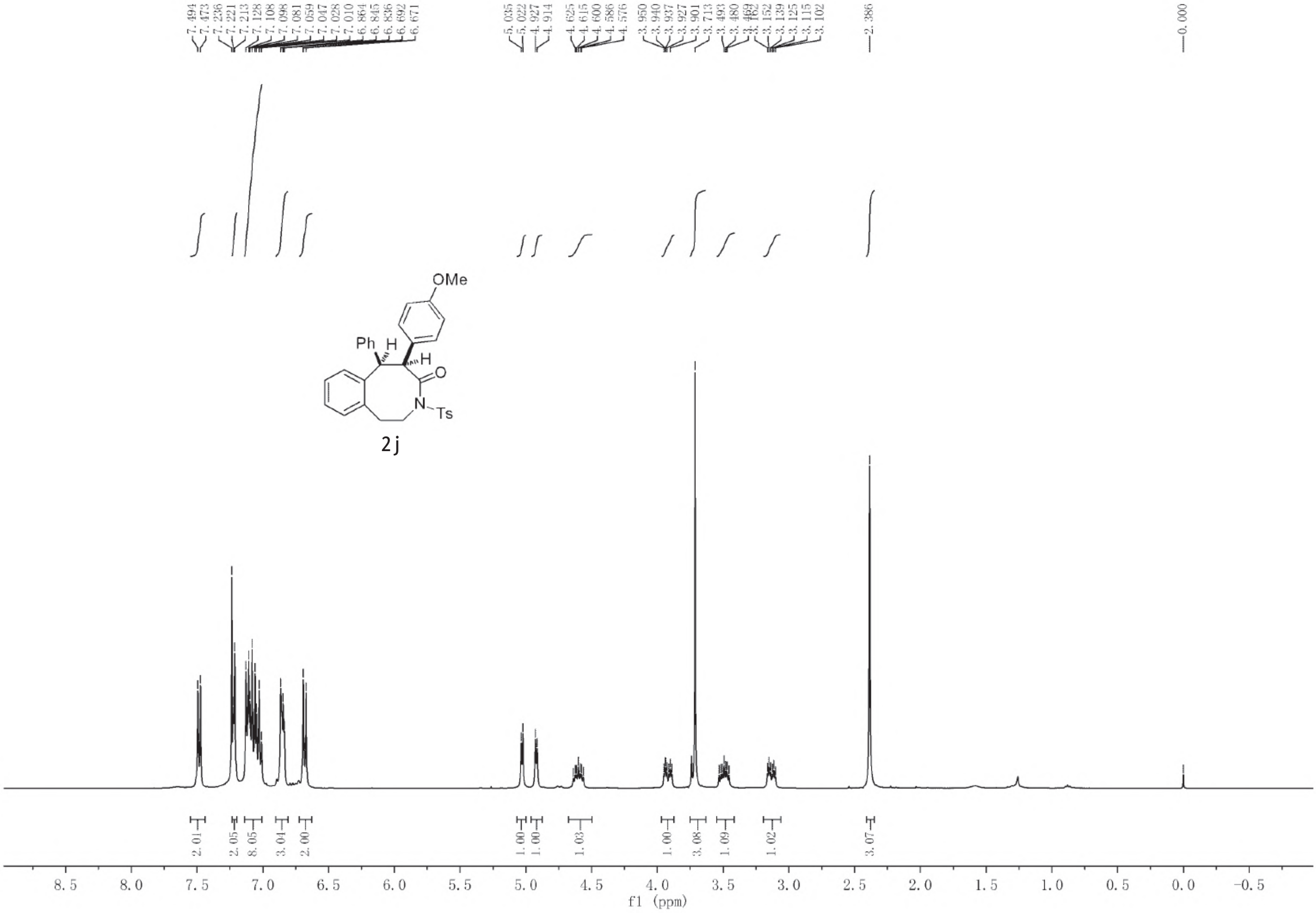

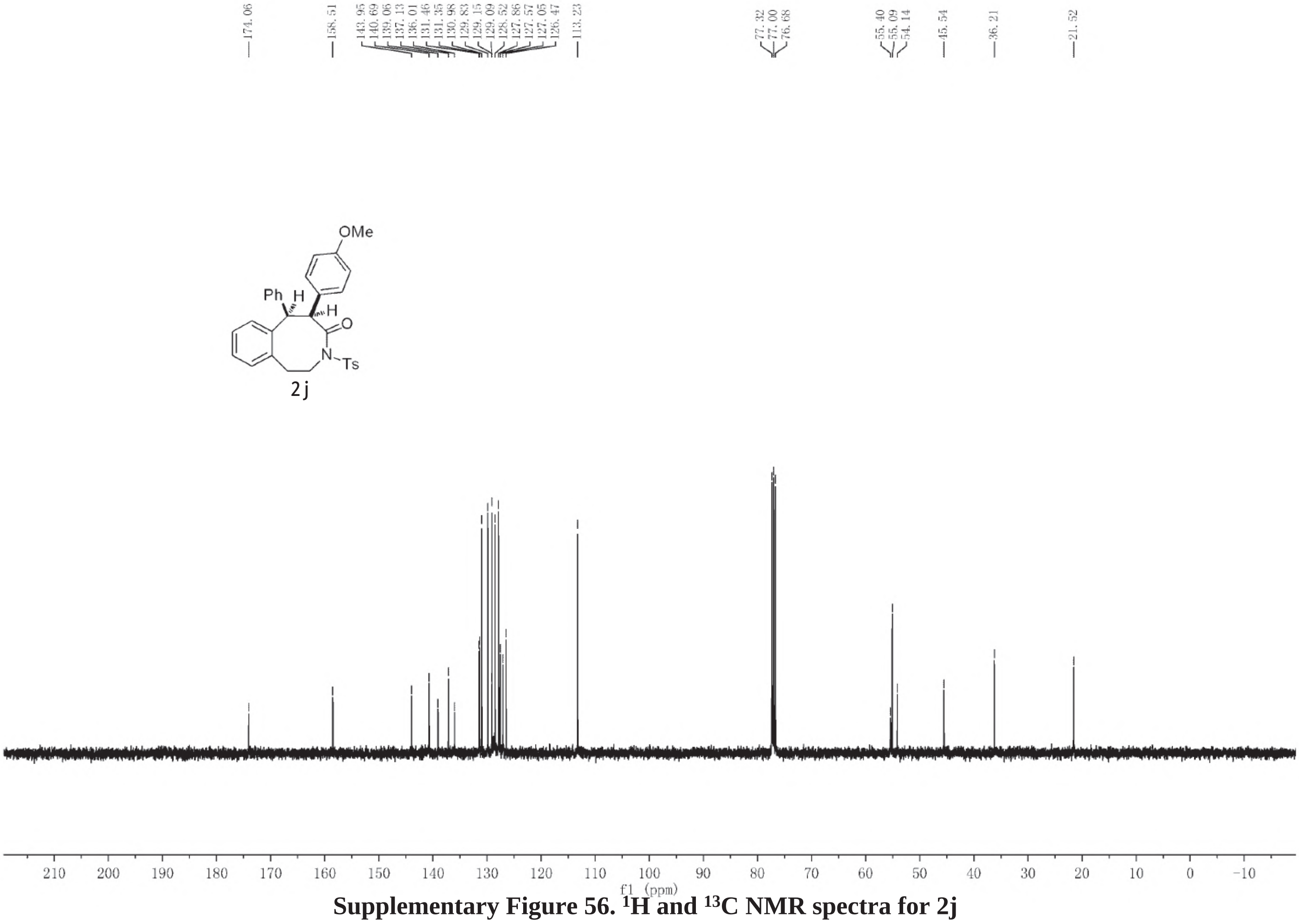

Supplementary Figure 56. ^{1}H and ^{13}C NMR spectra for 2j

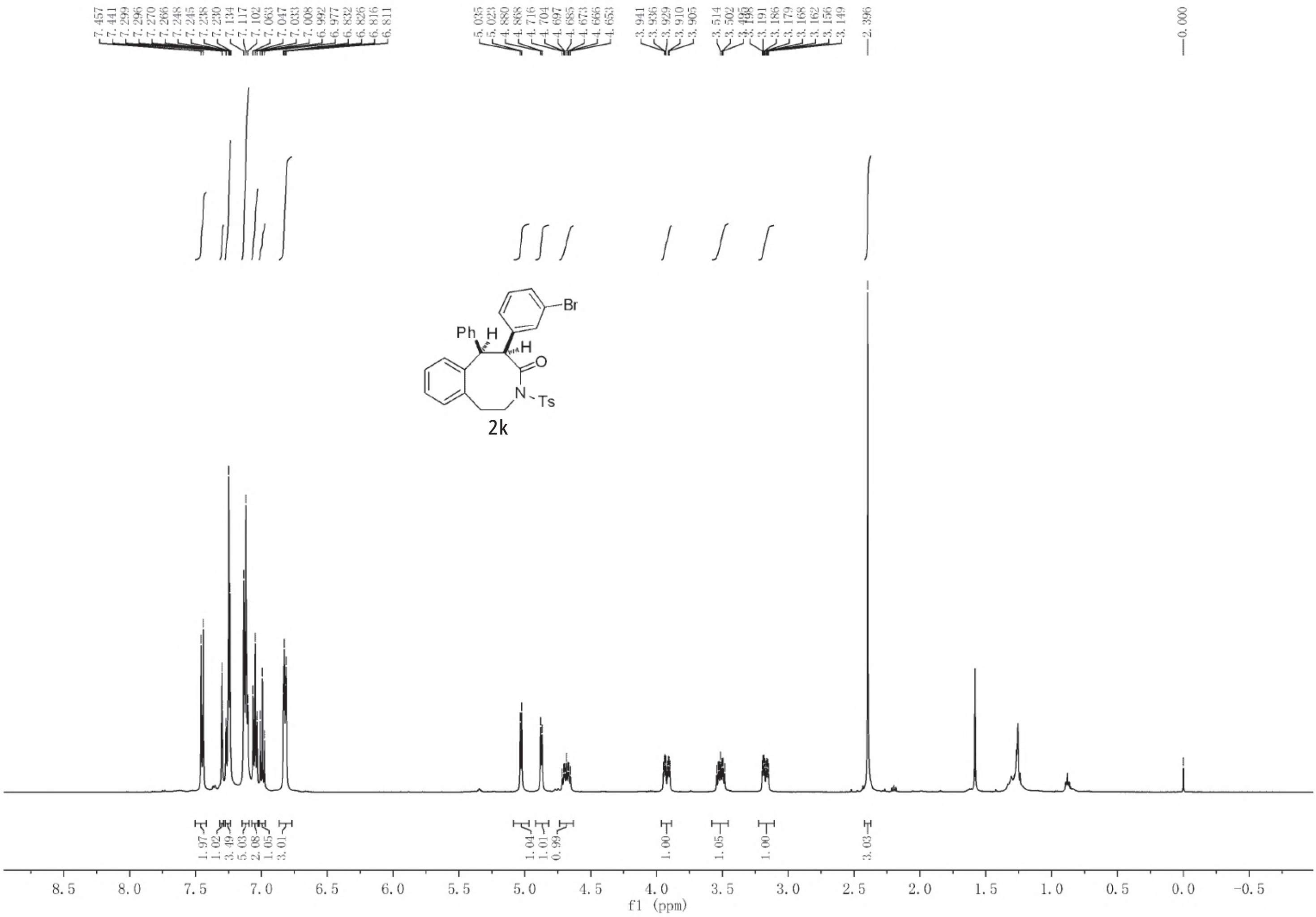

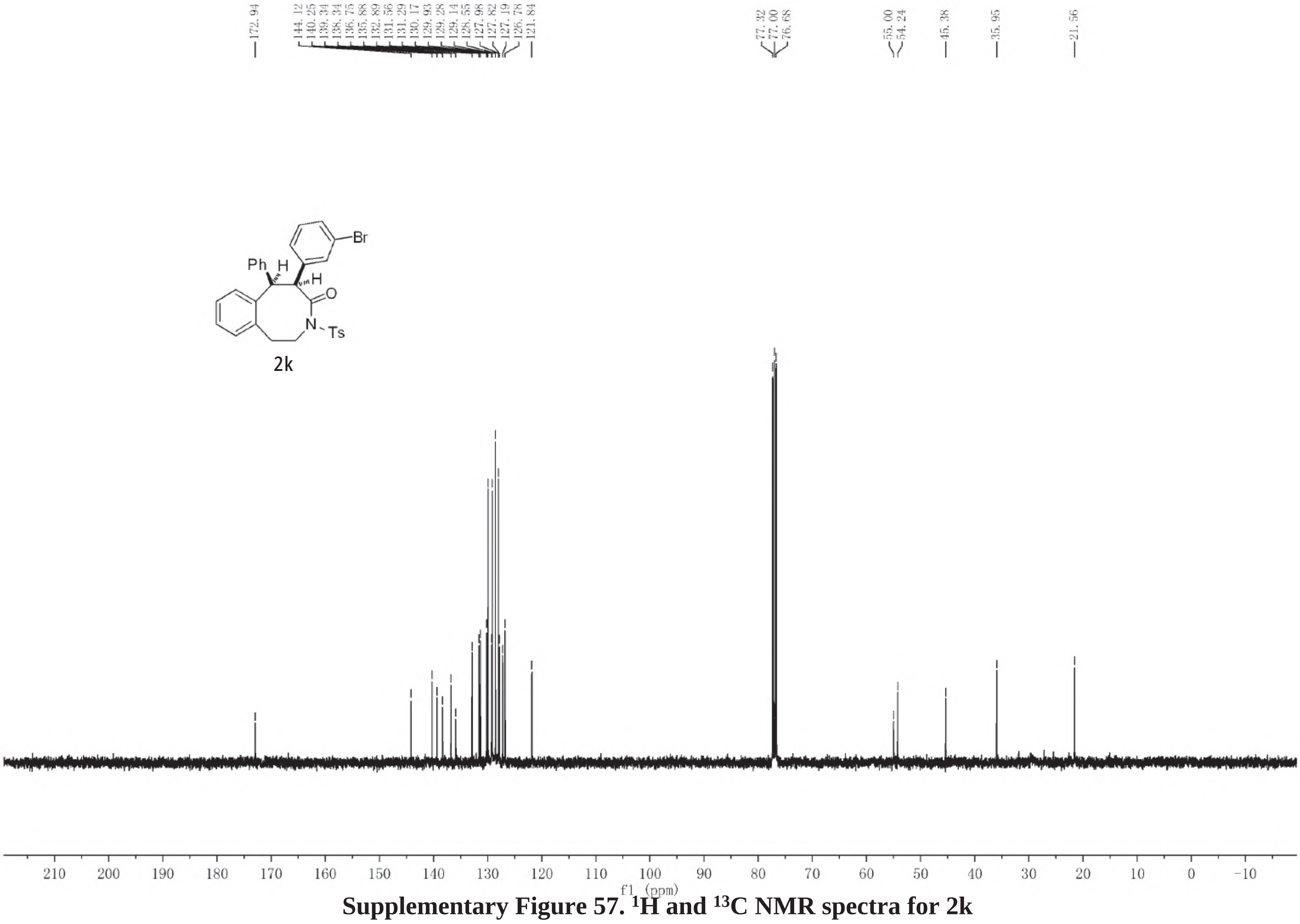

Supplementary Figure 57. ^{1}H and ^{13}C NMR spectra for 2k

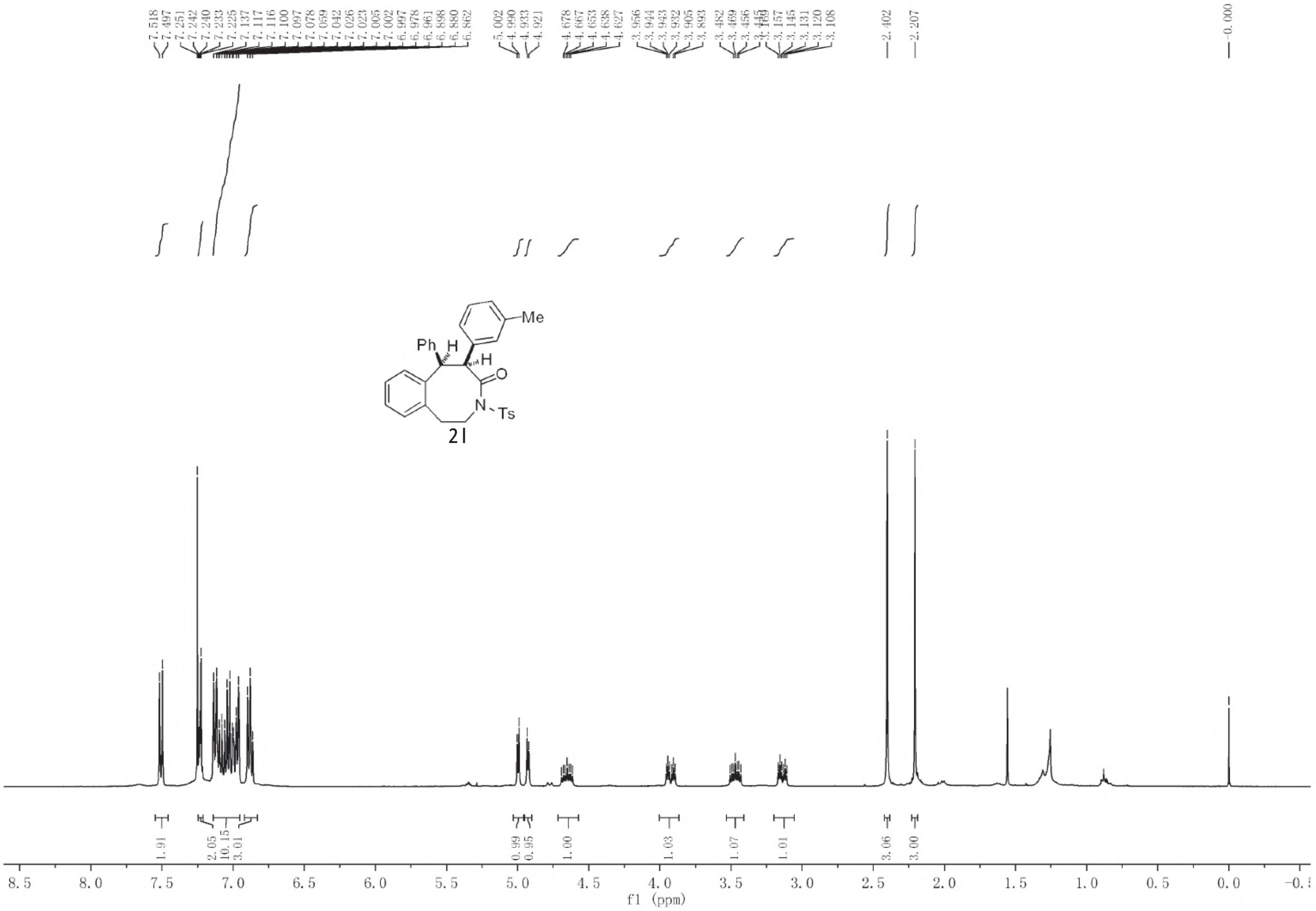

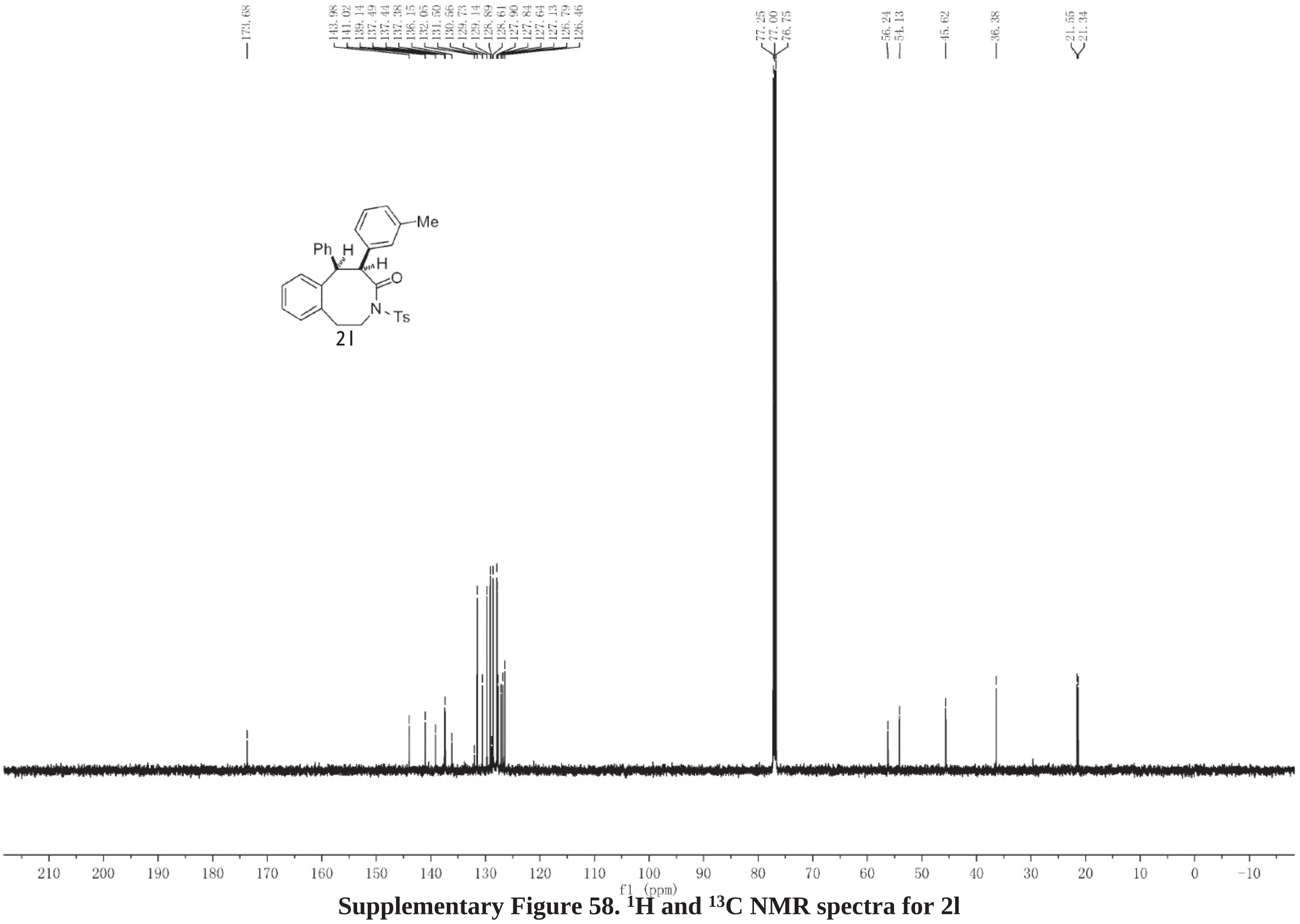

Supplementary Figure 58. ^{1}H and ^{13}C NMR spectra for 2l

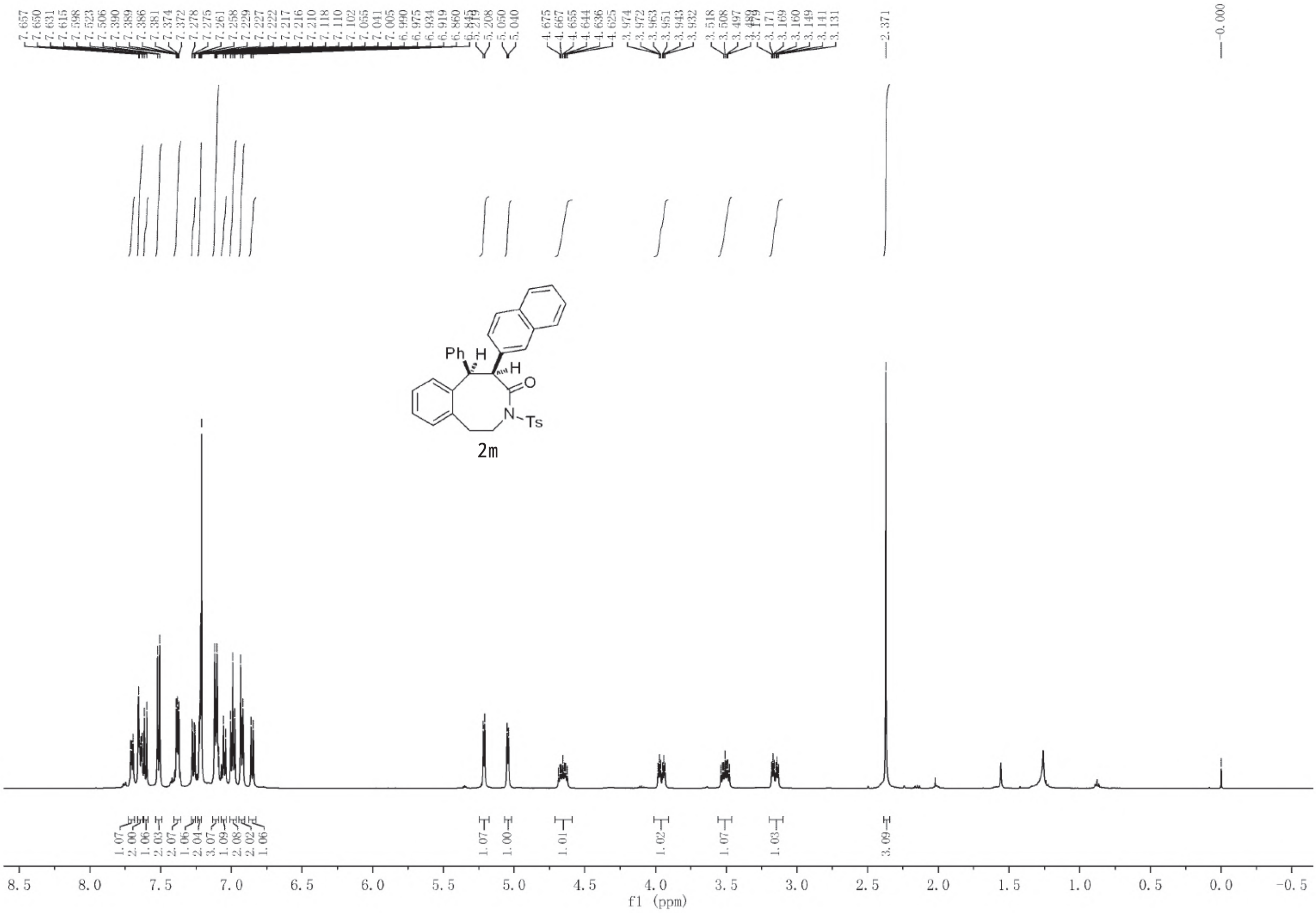

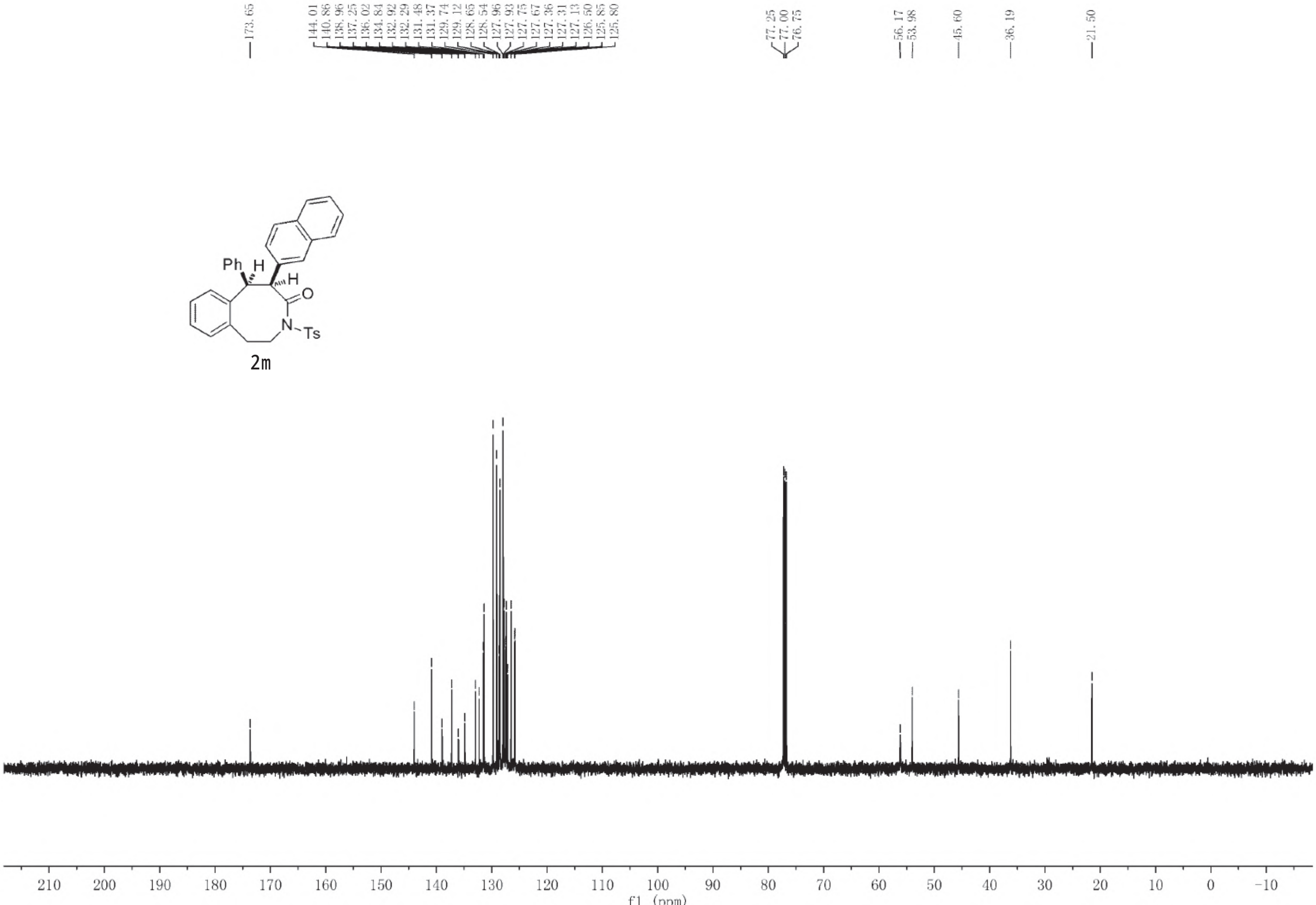

Supplementary Figure 59. ^{1}H and ^{13}C NMR spectra for 2m

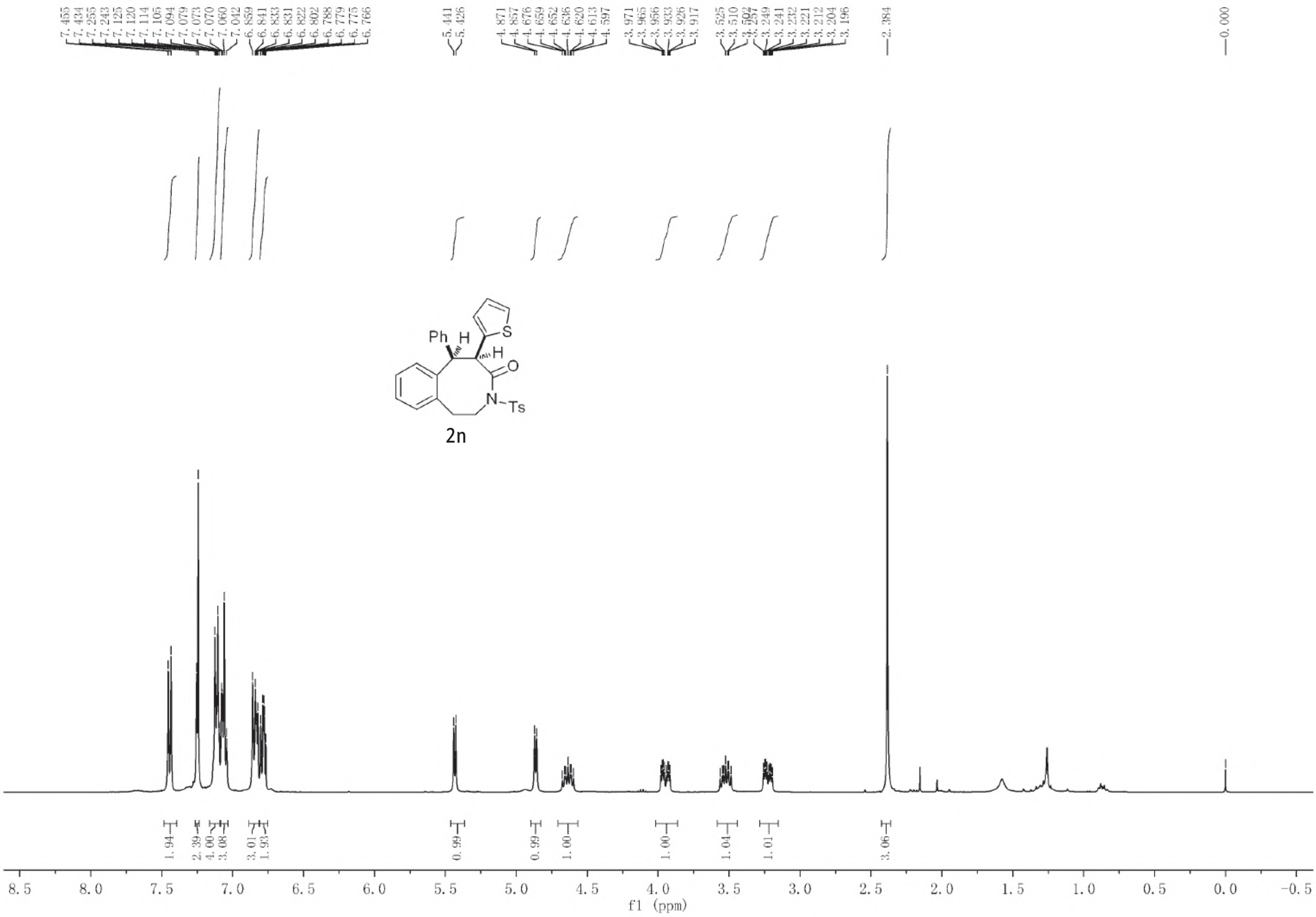

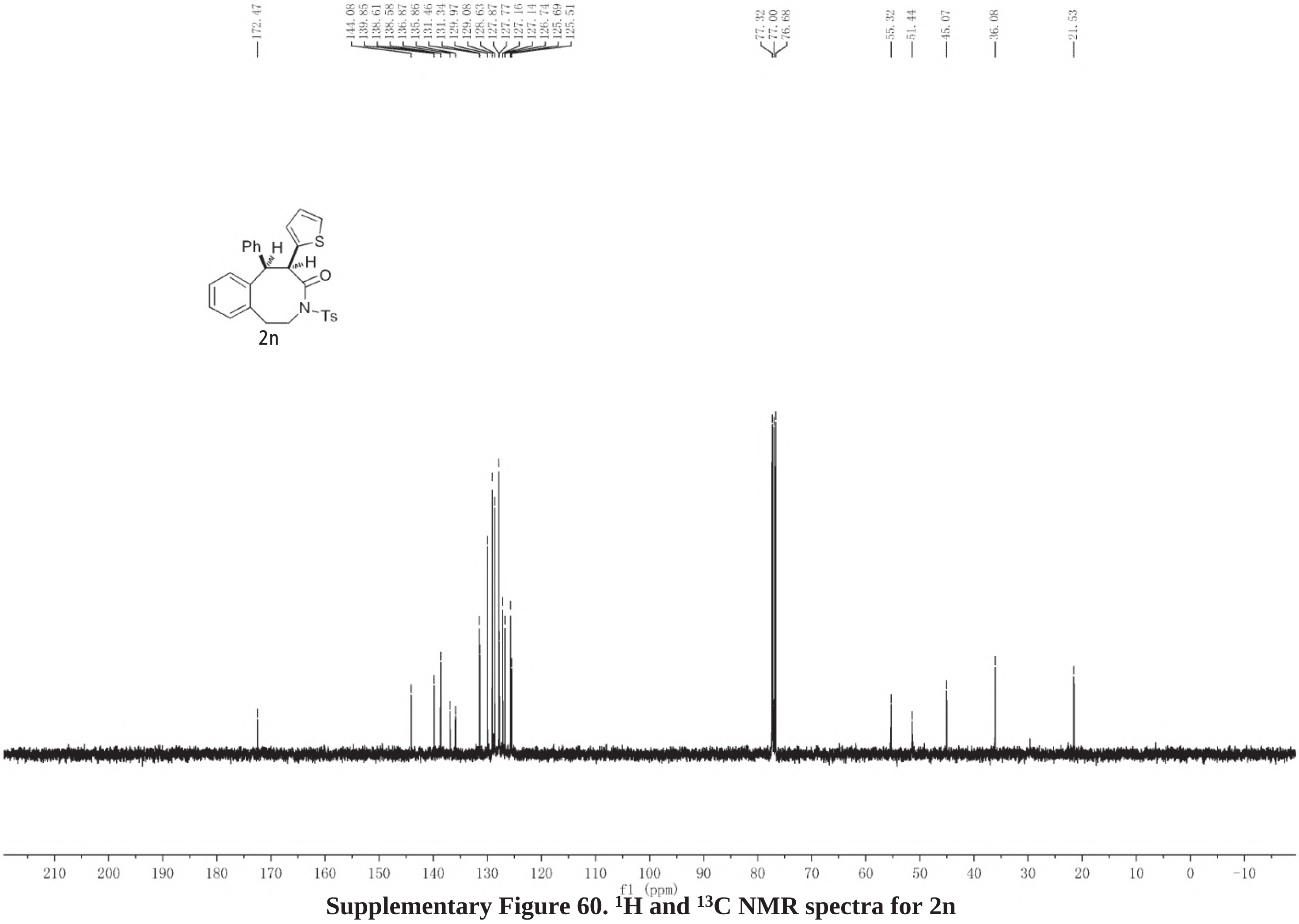

Supplementary Figure 60. ^{1}H and ^{13}C NMR spectra for 2n

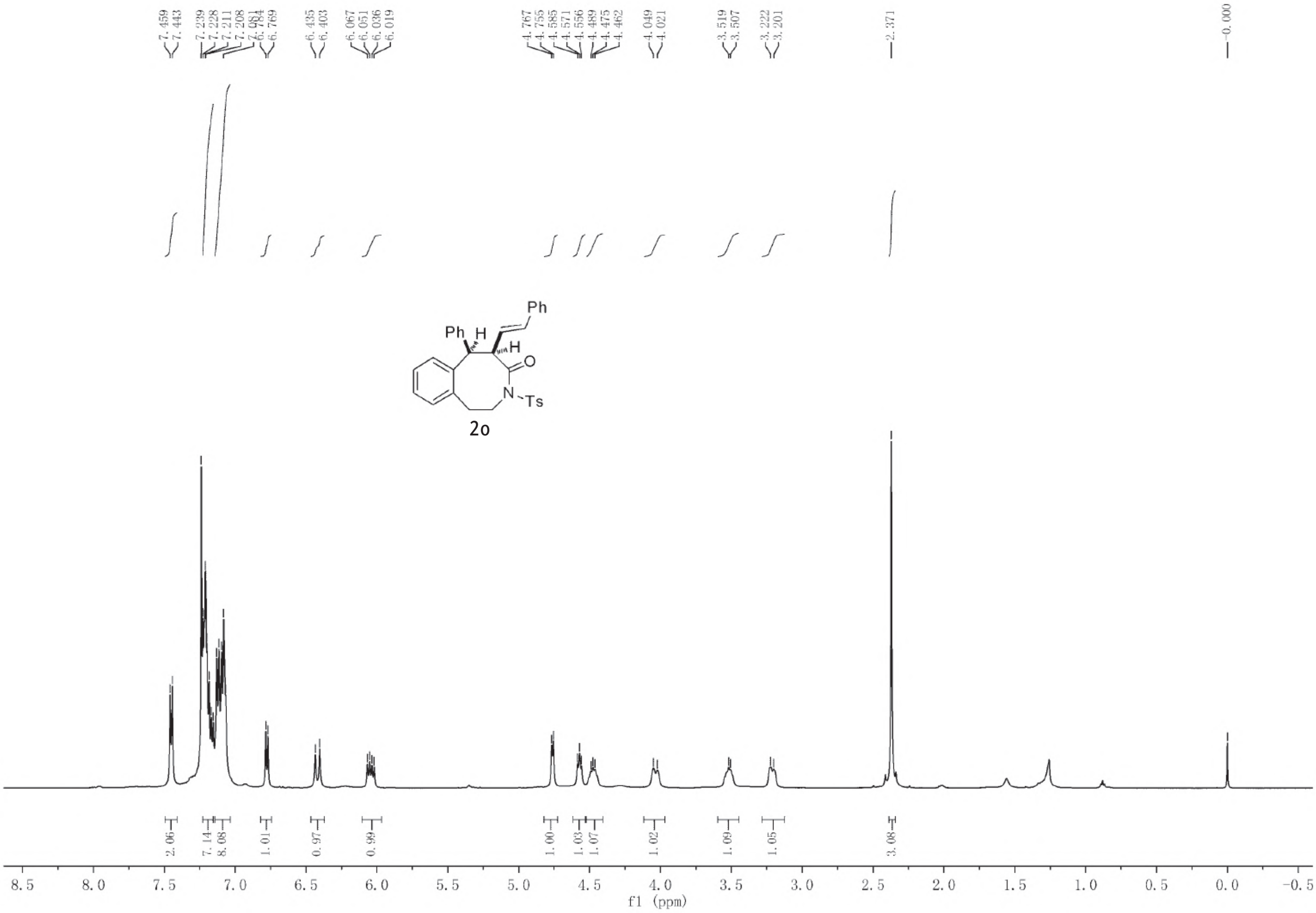

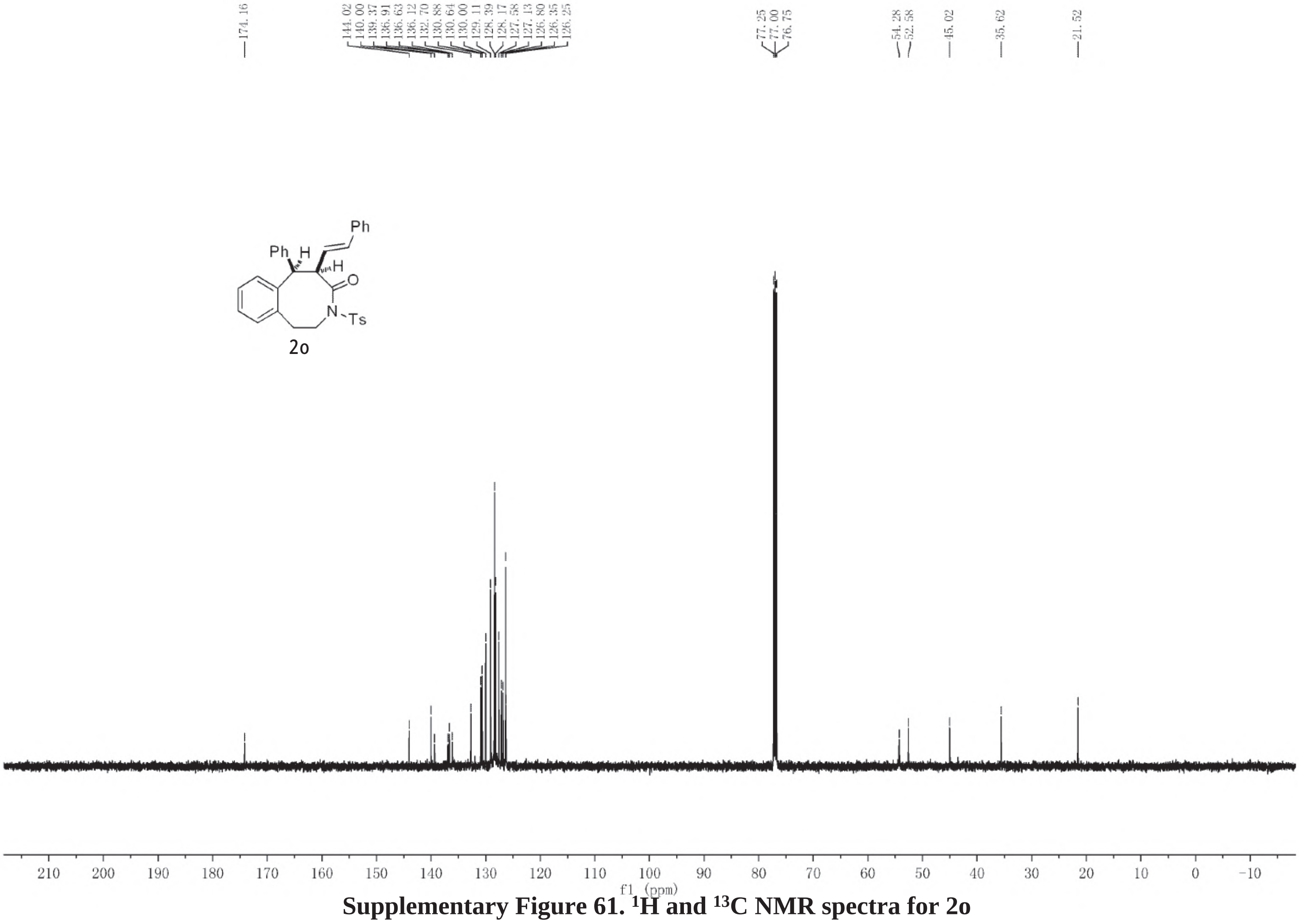

Supplementary Figure 61. ^{1}H and ^{13}C NMR spectra for 2o

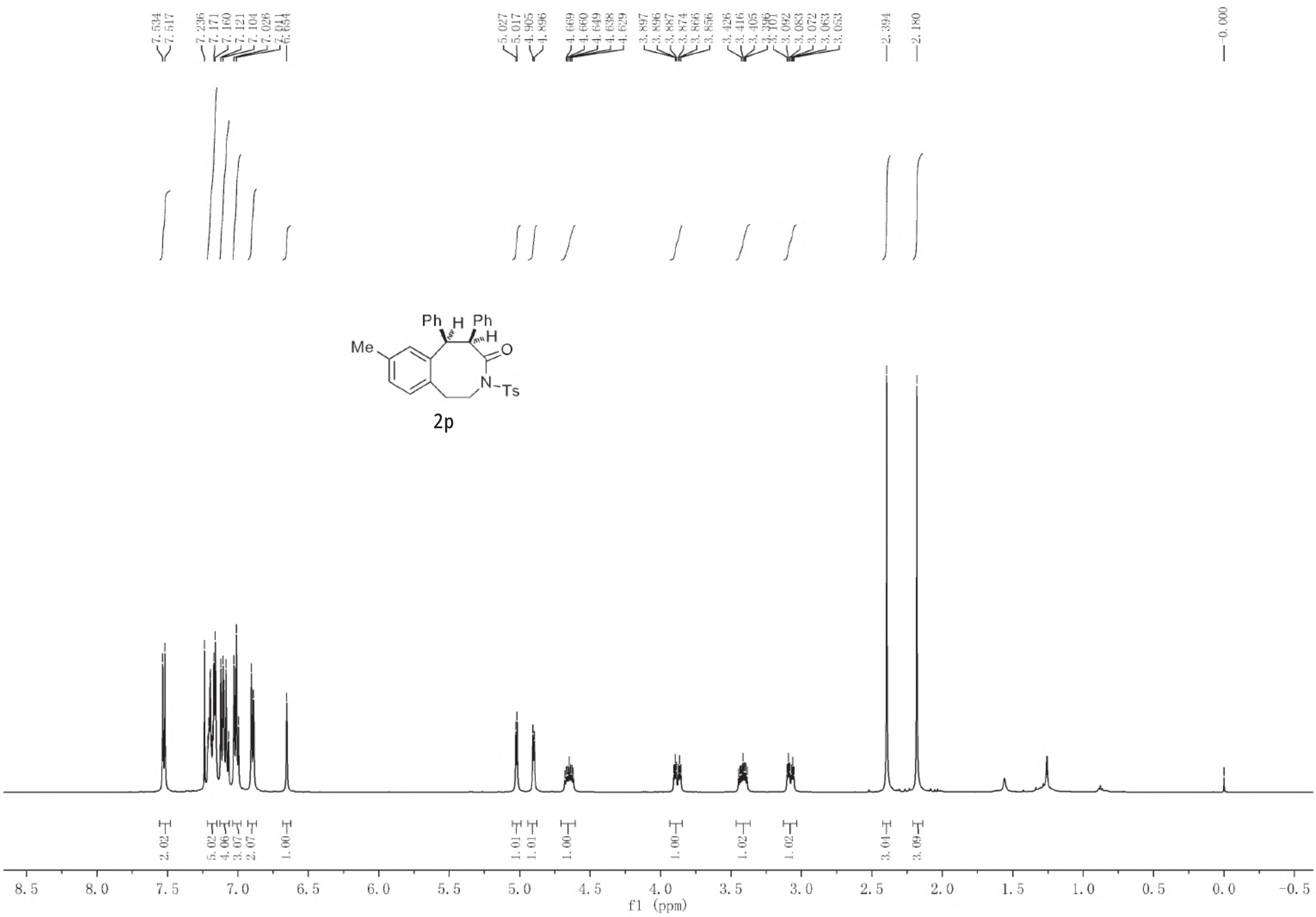

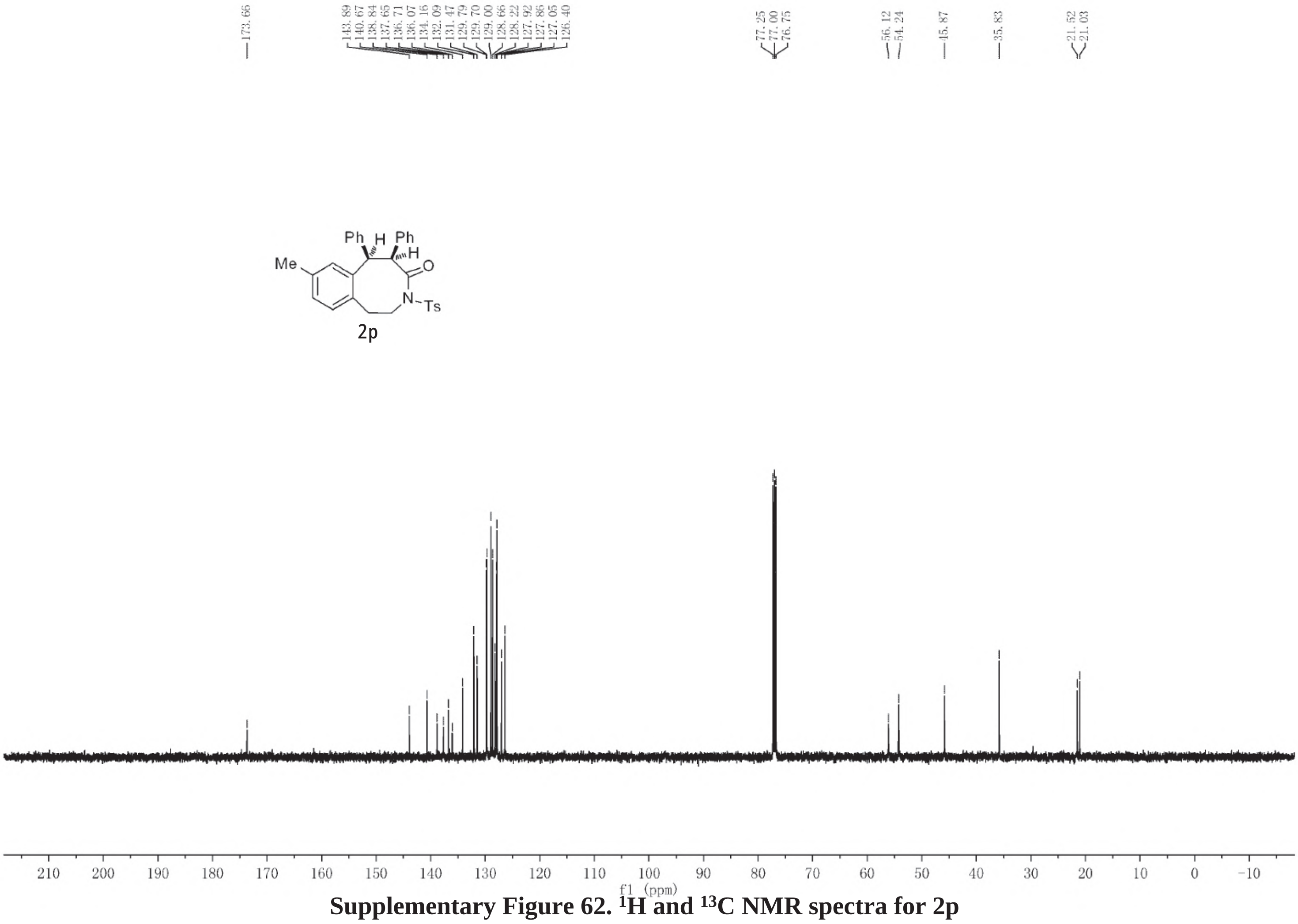

Supplementary Figure 62. ^{1}H and ^{13}C NMR spectra for 2p

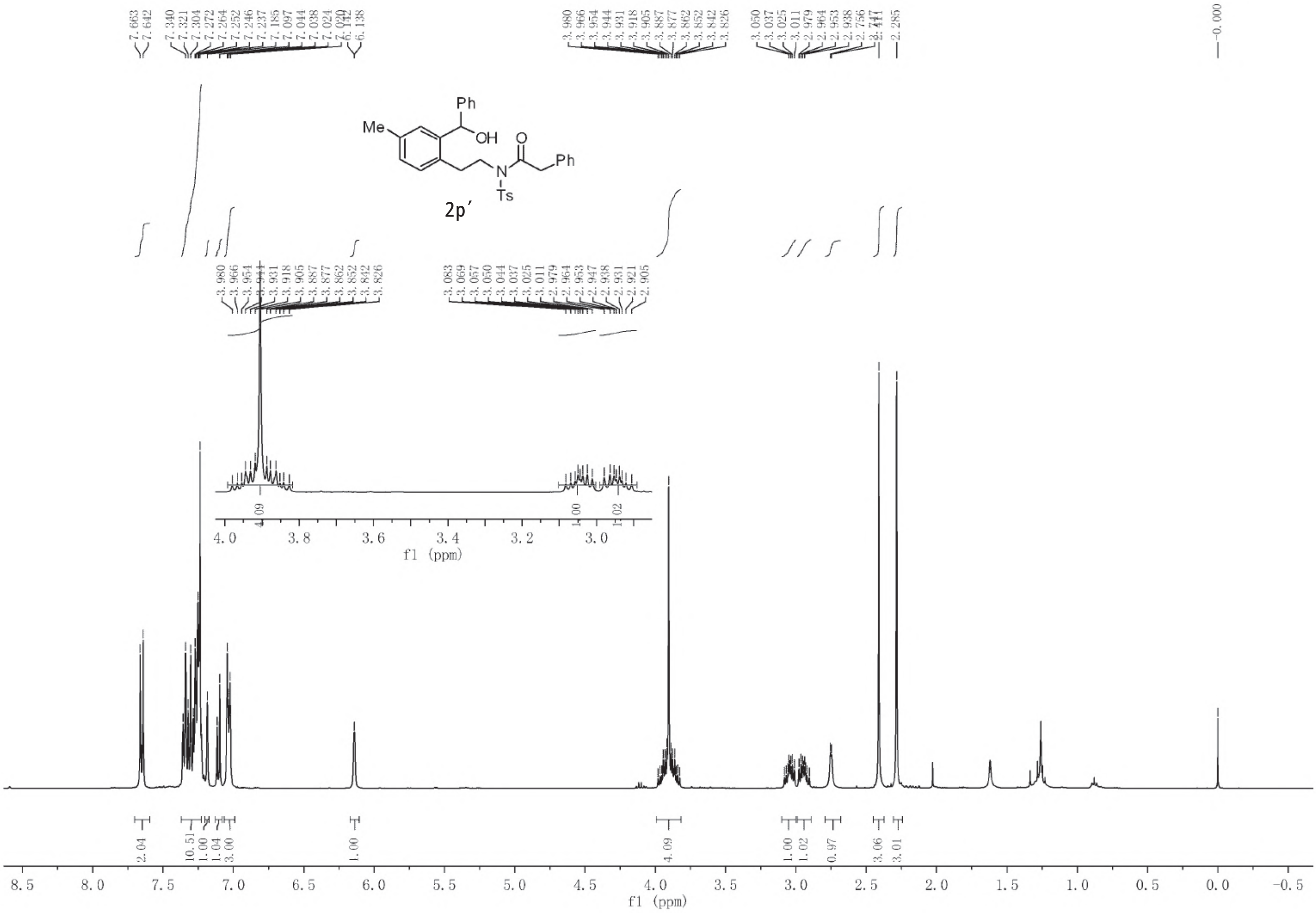

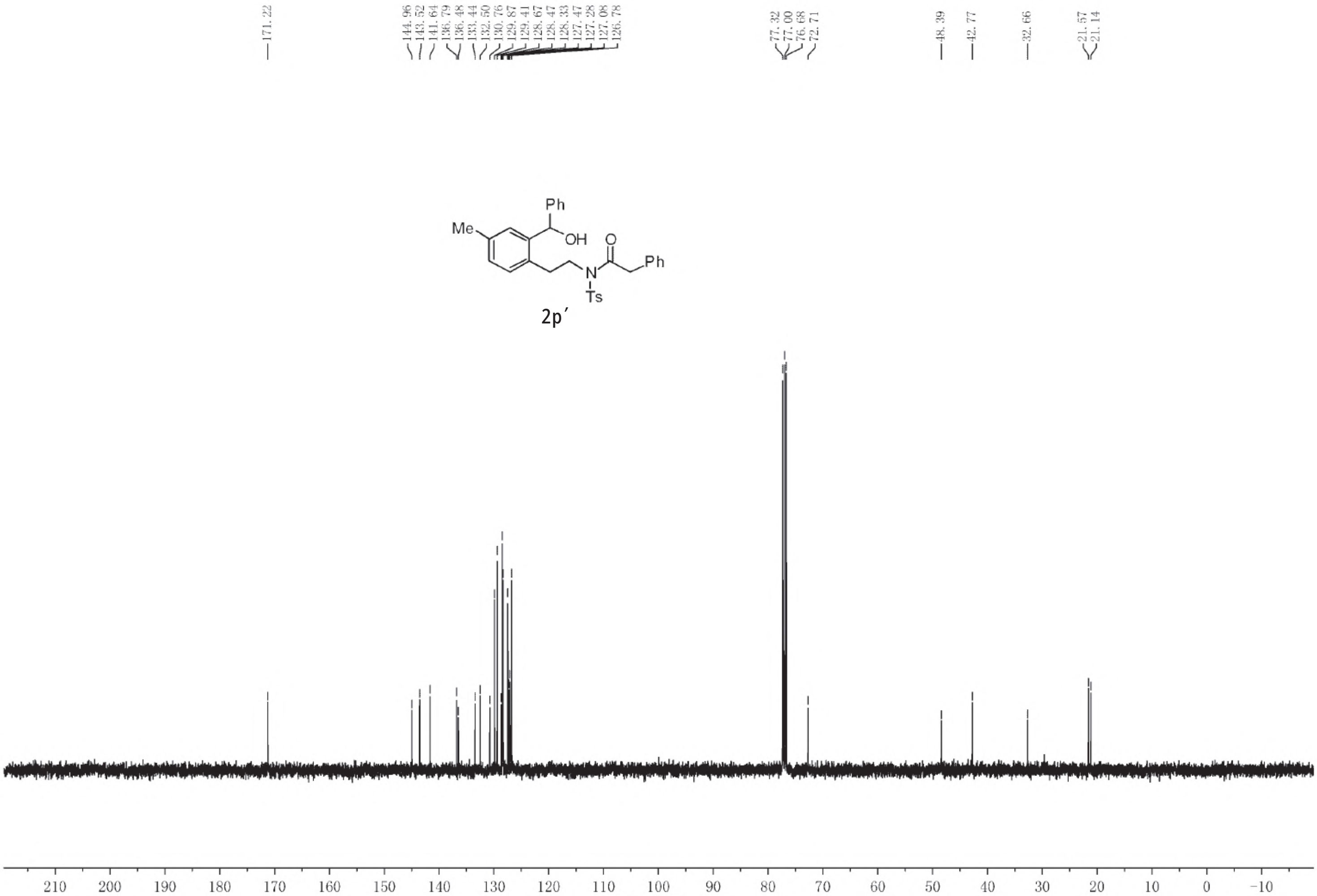

Supplementary Figure 63. ^{1}H and ^{13}C NMR spectra for 2p'

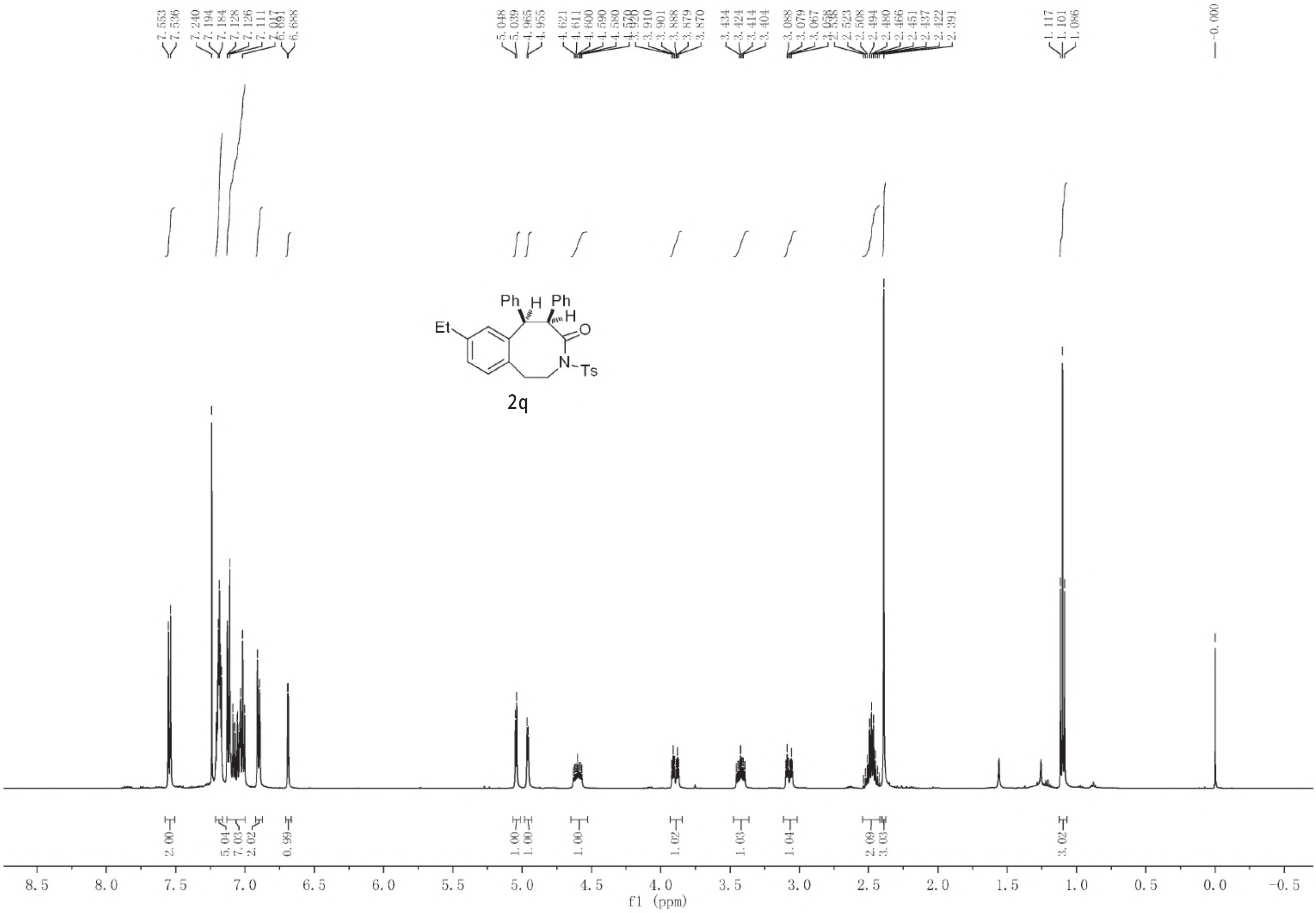

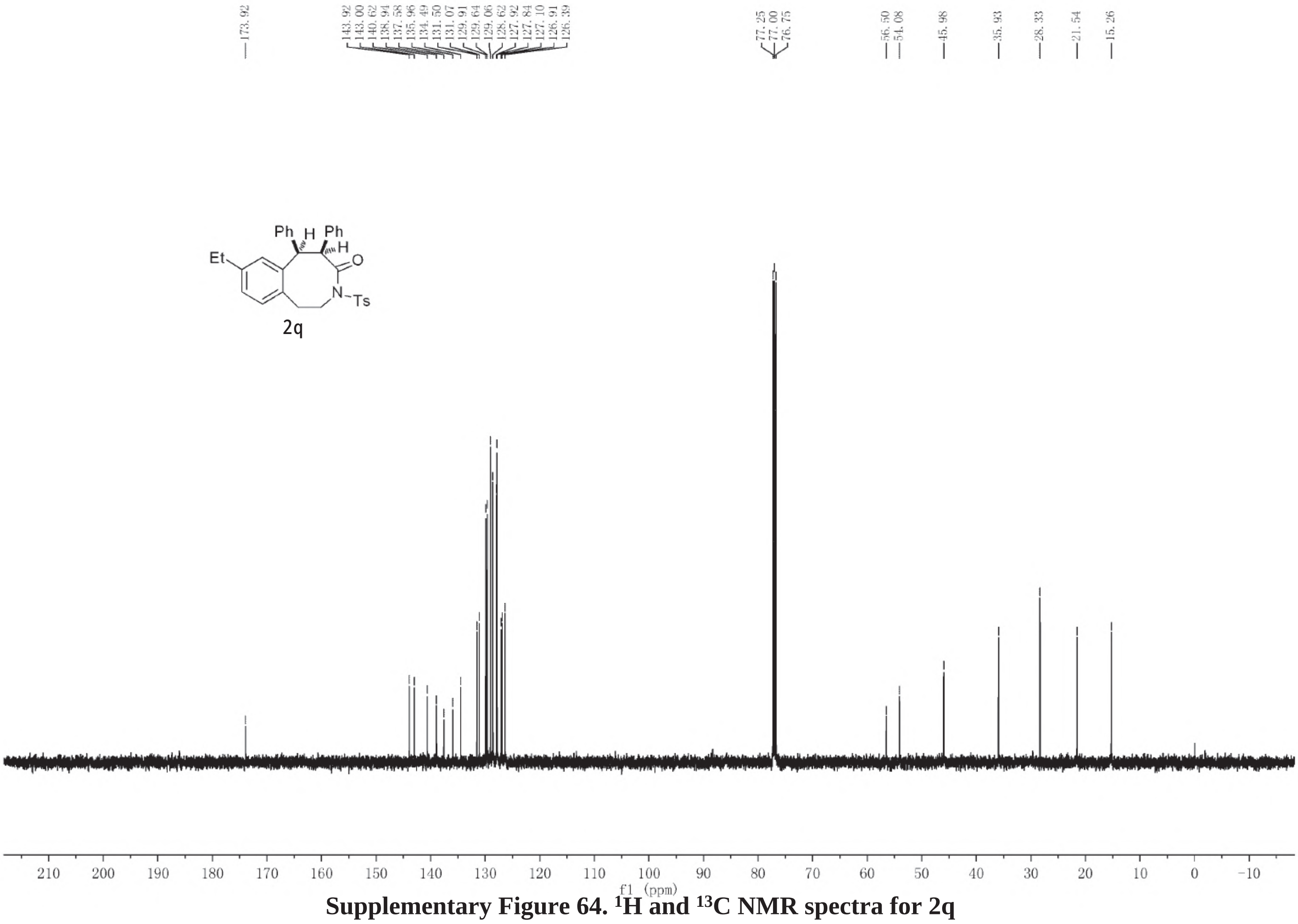

Supplementary Figure 64. ^{1}H and ^{13}C NMR spectra for 2q

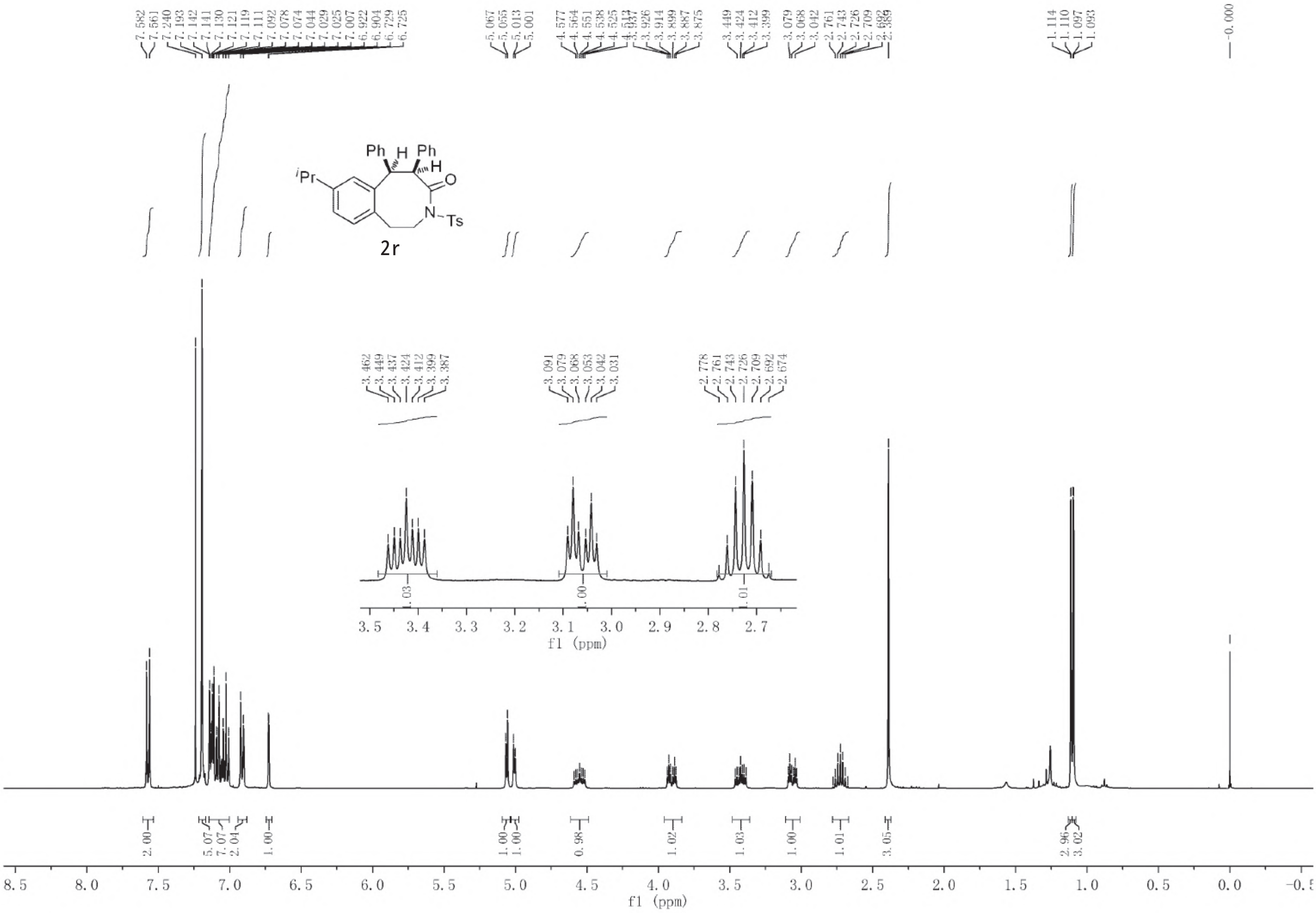

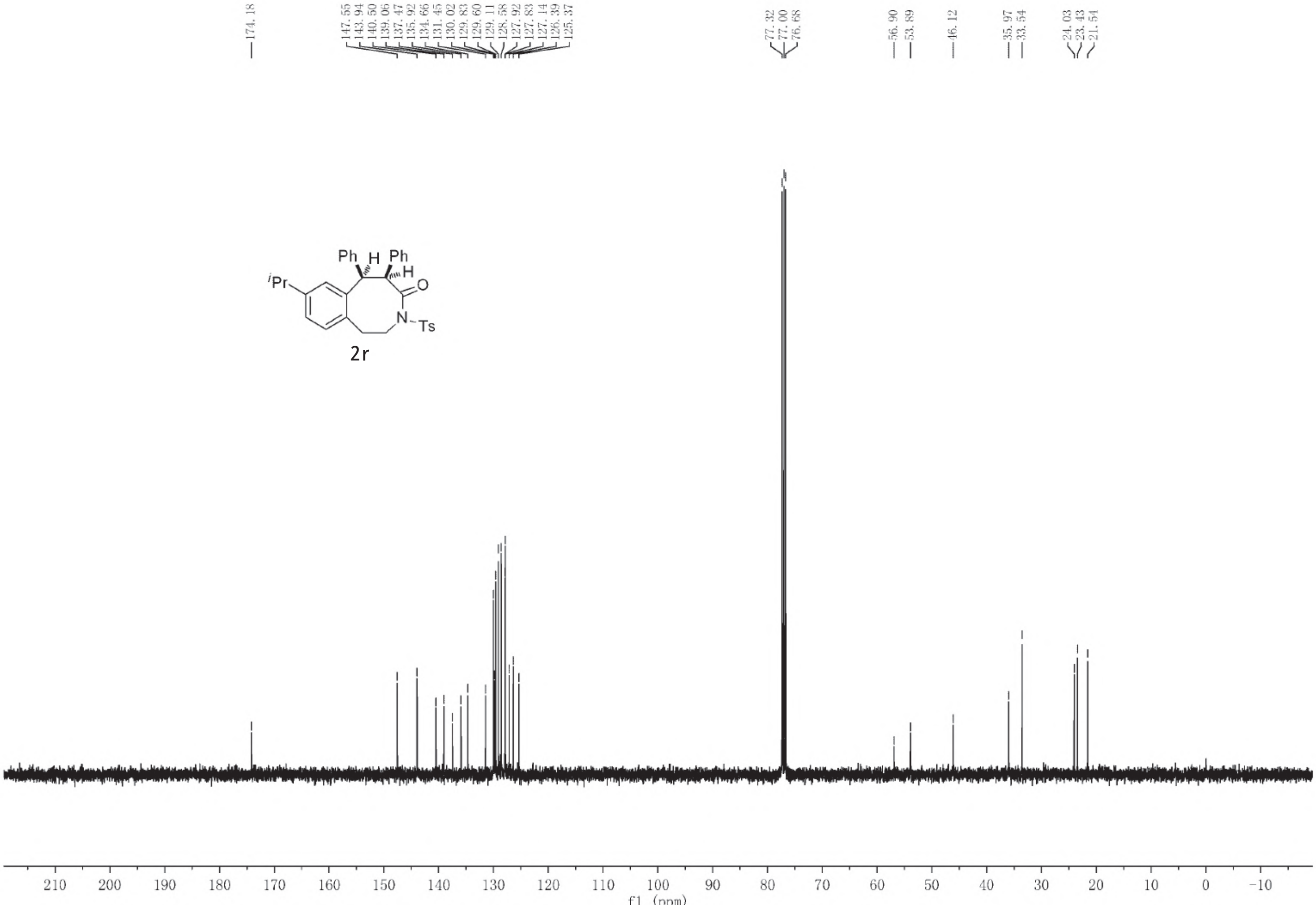

Supplementary Figure 65. ^{1}H and ^{13}C NMR spectra for 2r

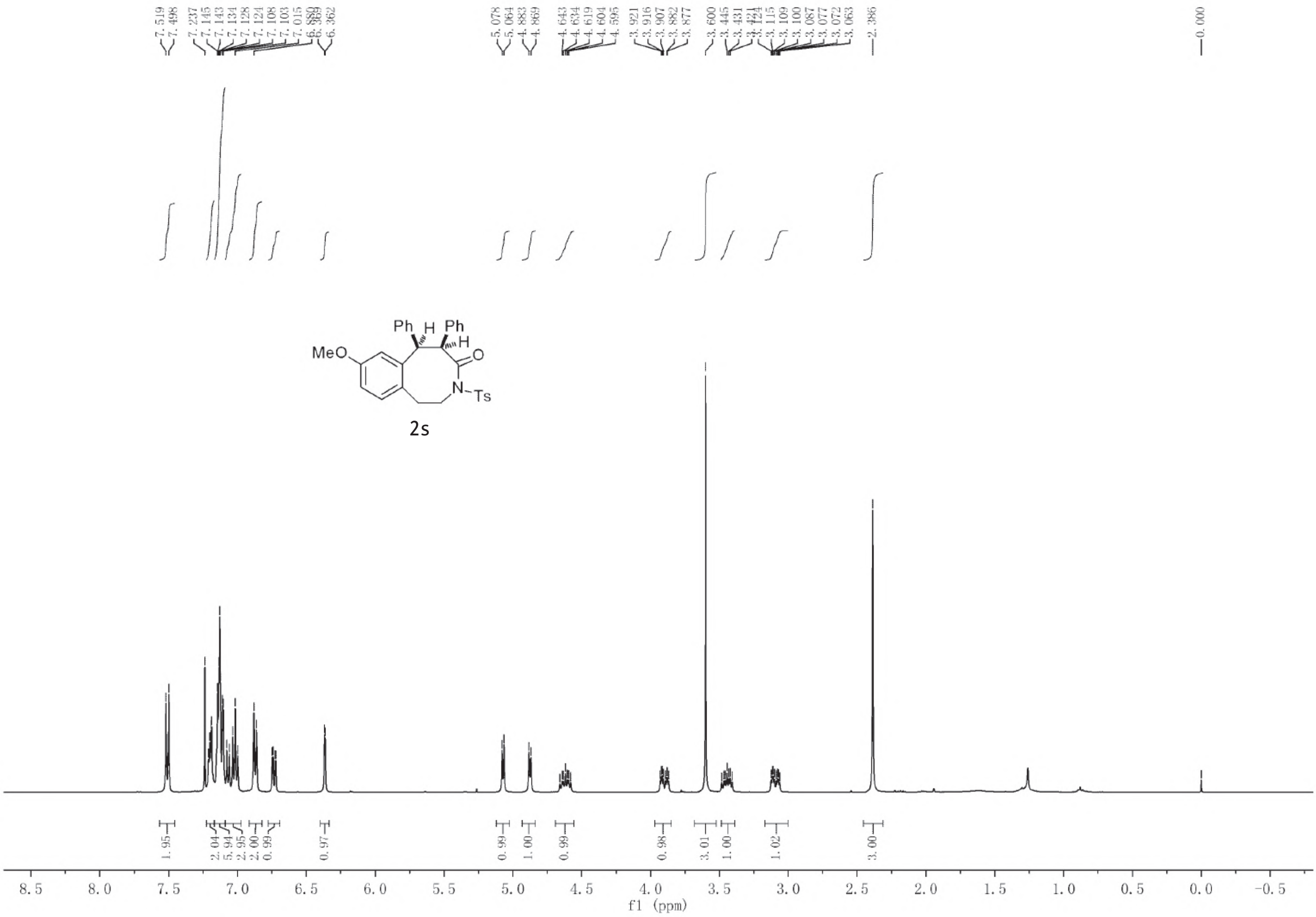

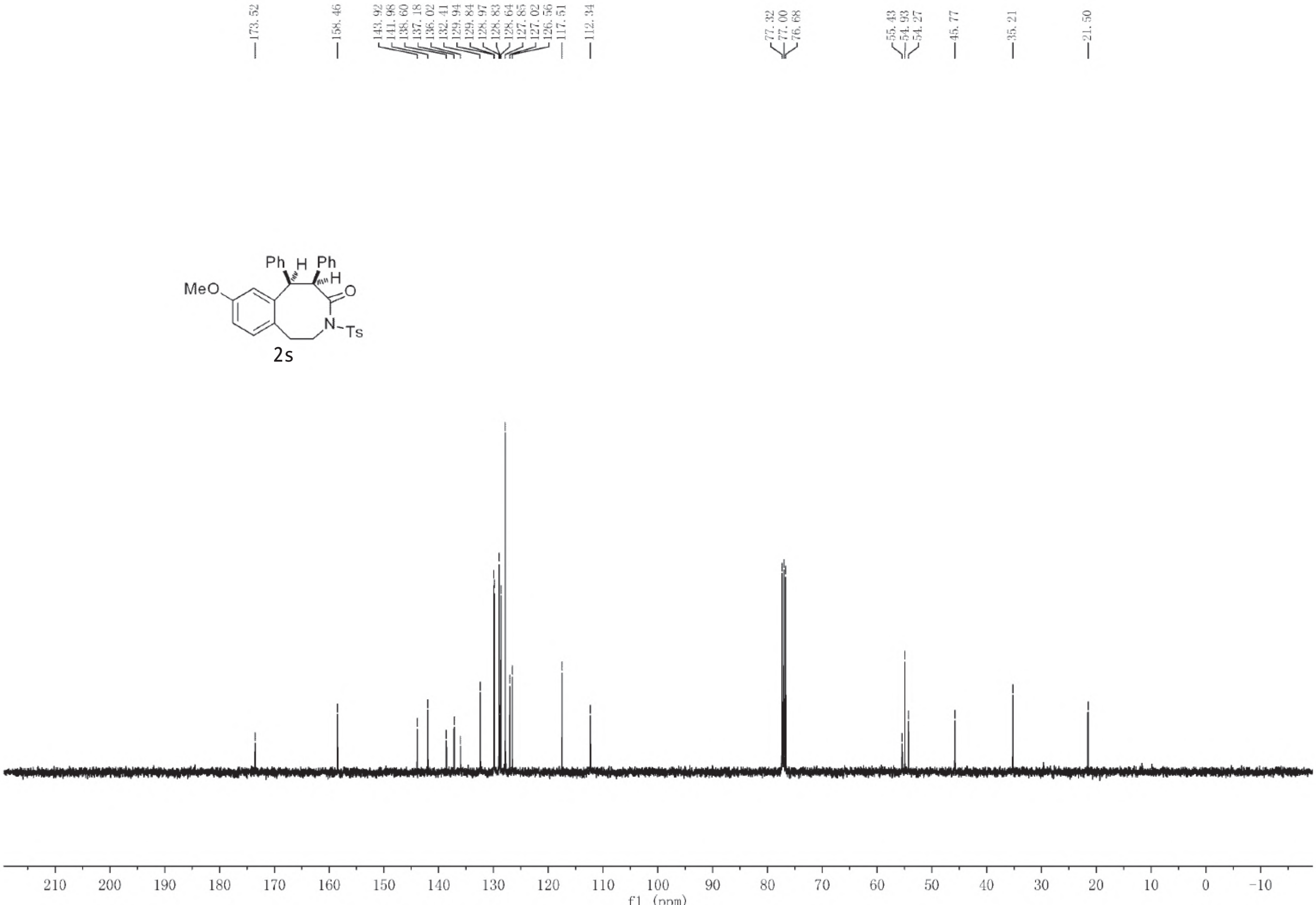

Supplementary Figure 66. ^{1}H and ^{13}C NMR spectra for 2s

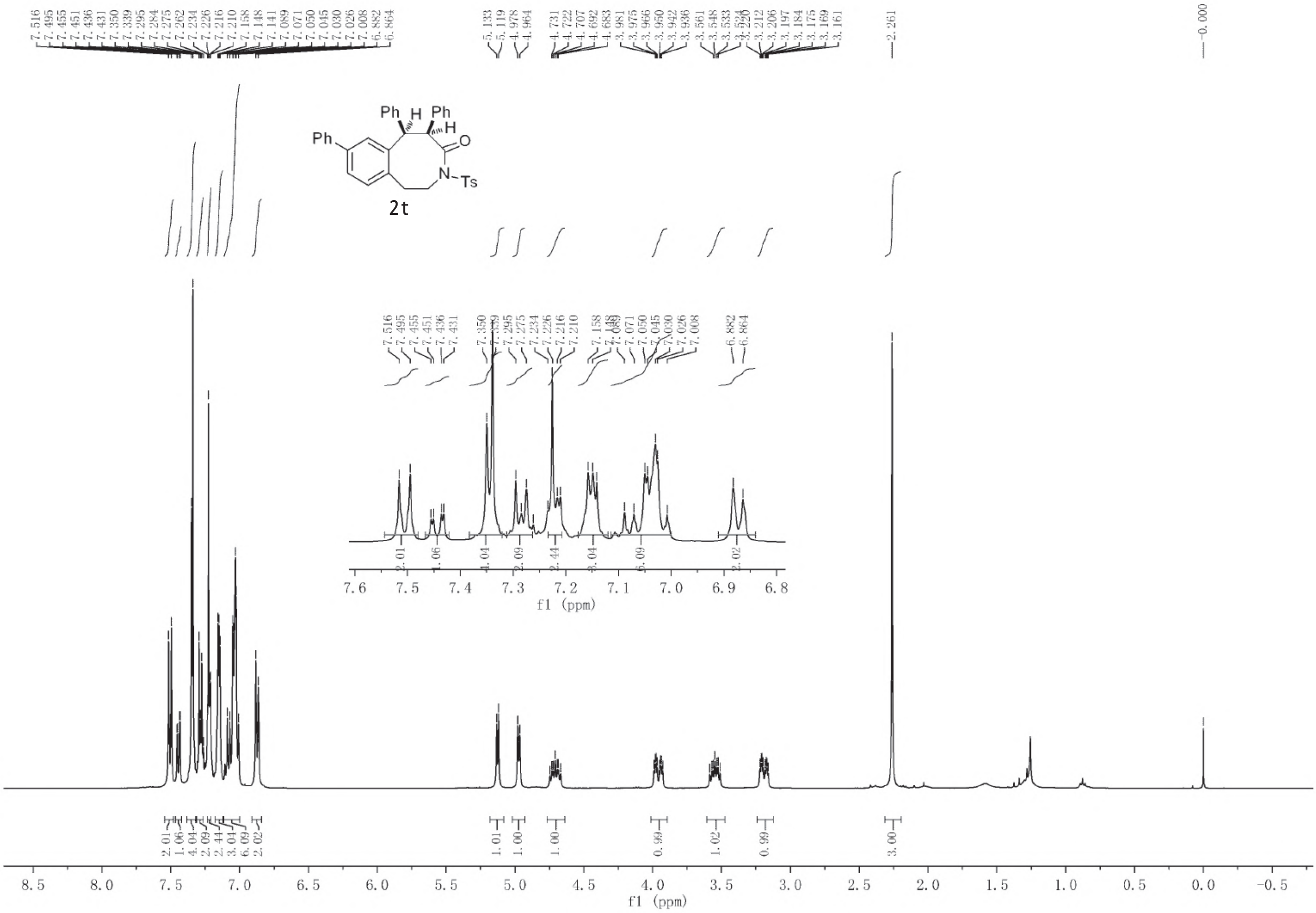

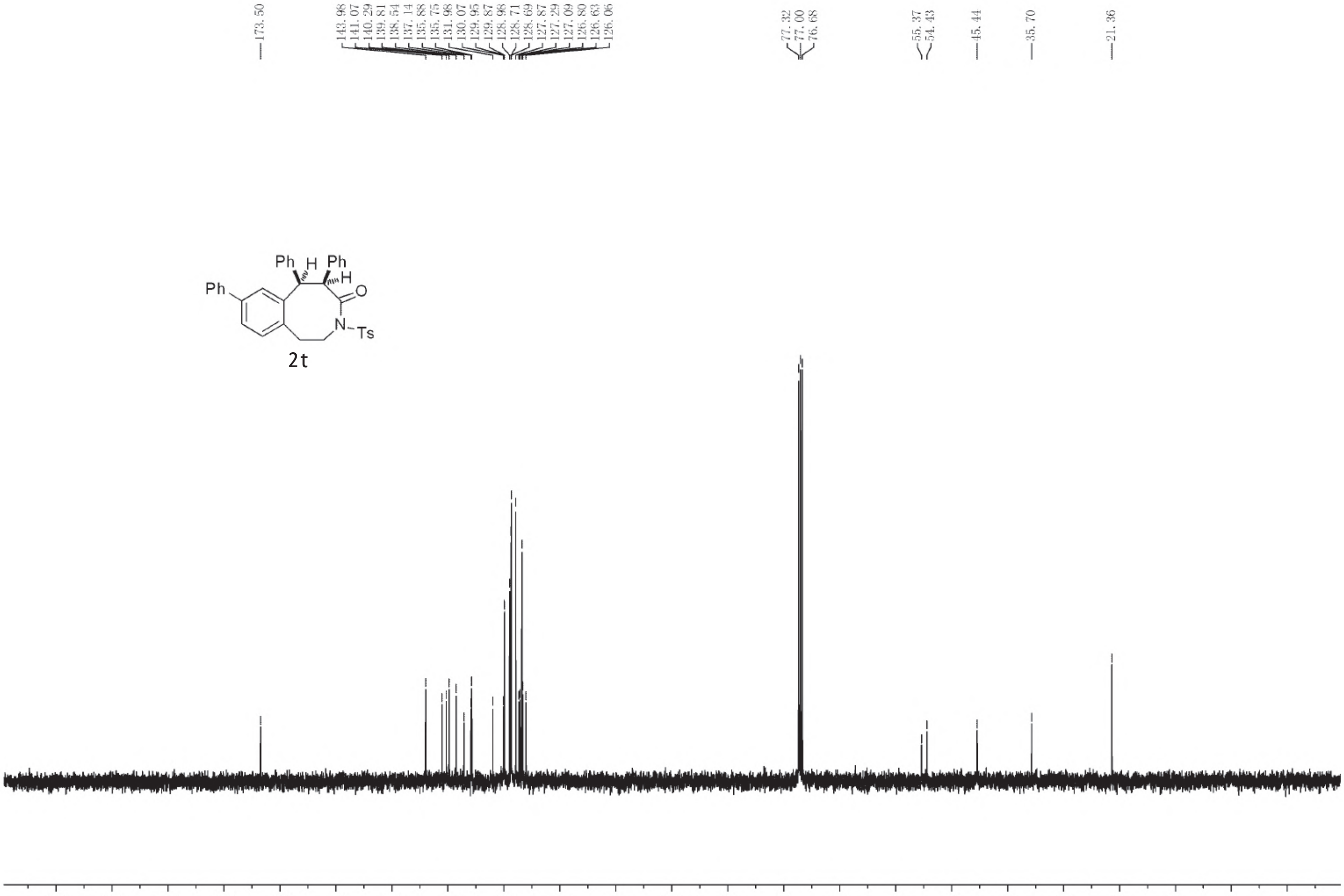

Supplementary Figure 67. ^{1}H and ^{13}C NMR spectra for 2t

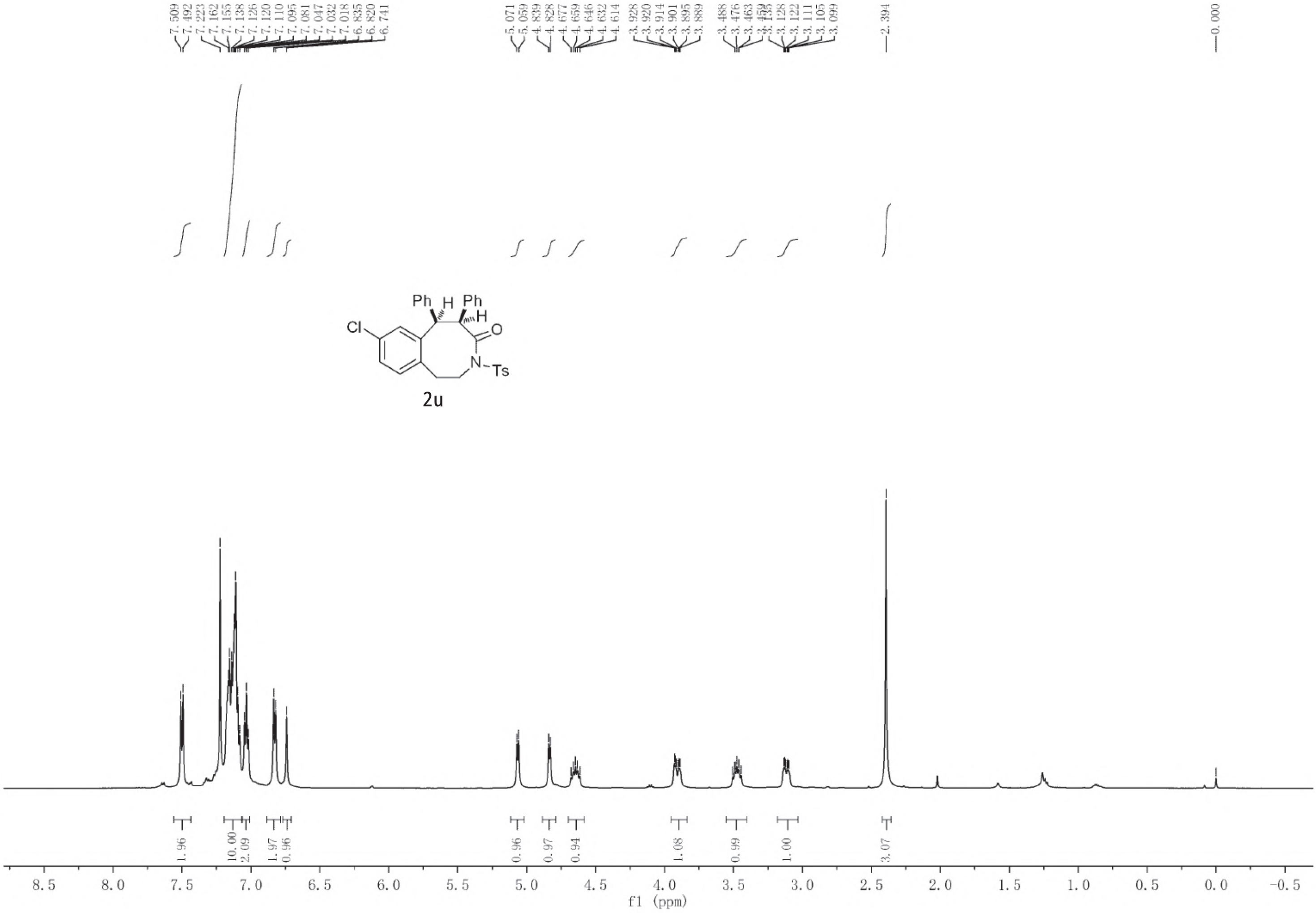

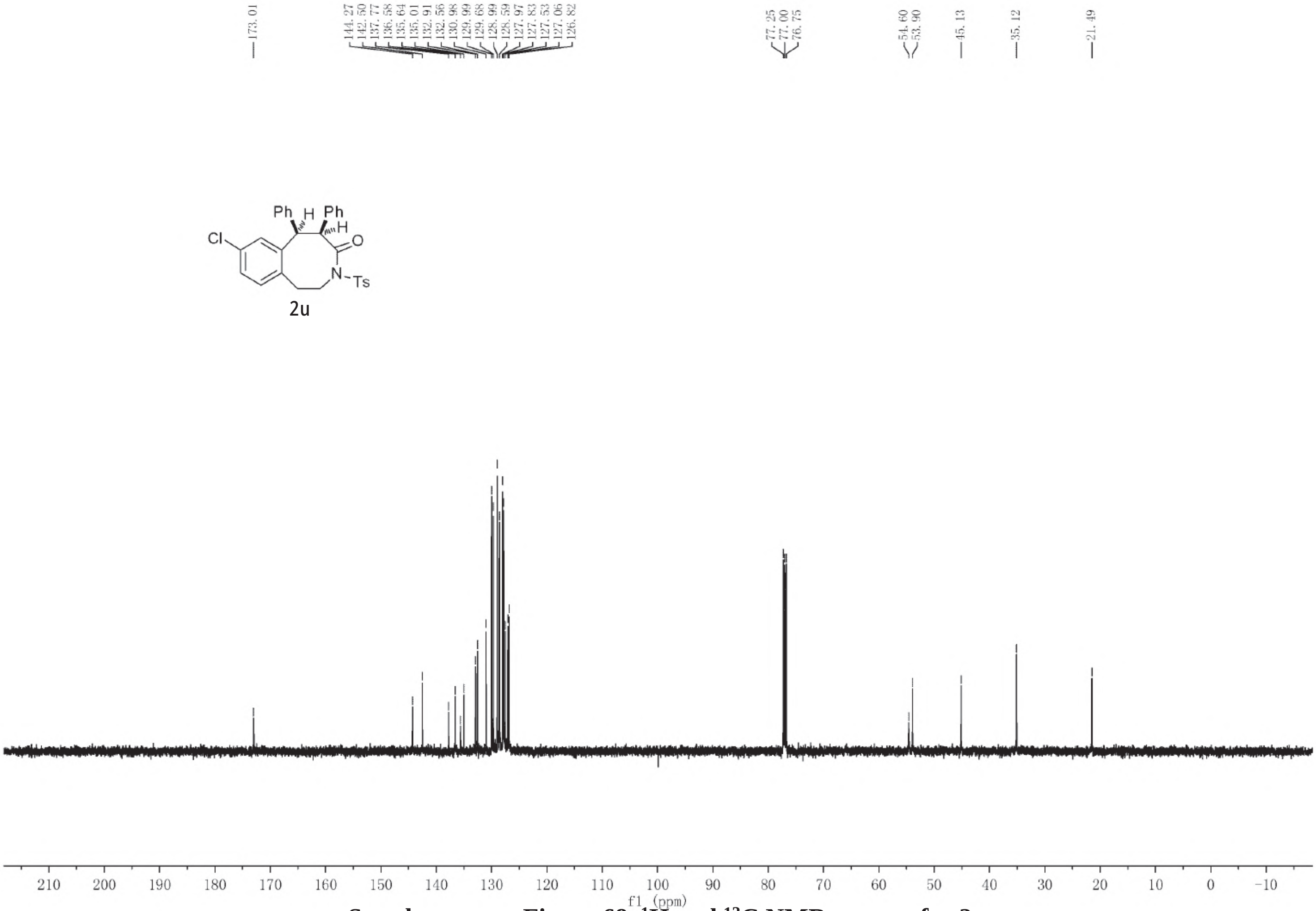

Supplementary Figure 68. ^{1}H and ^{13}C NMR spectra for 2u

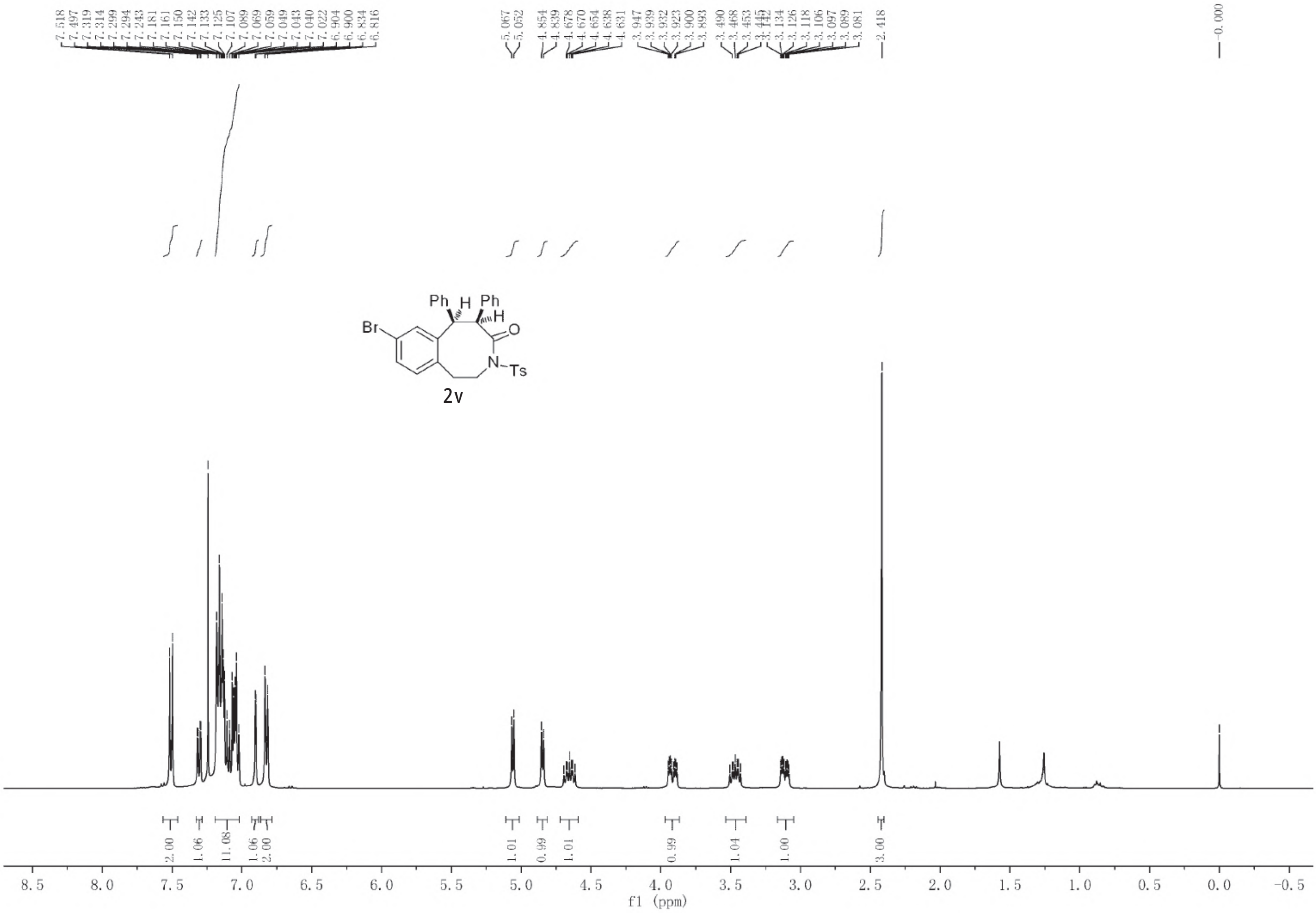

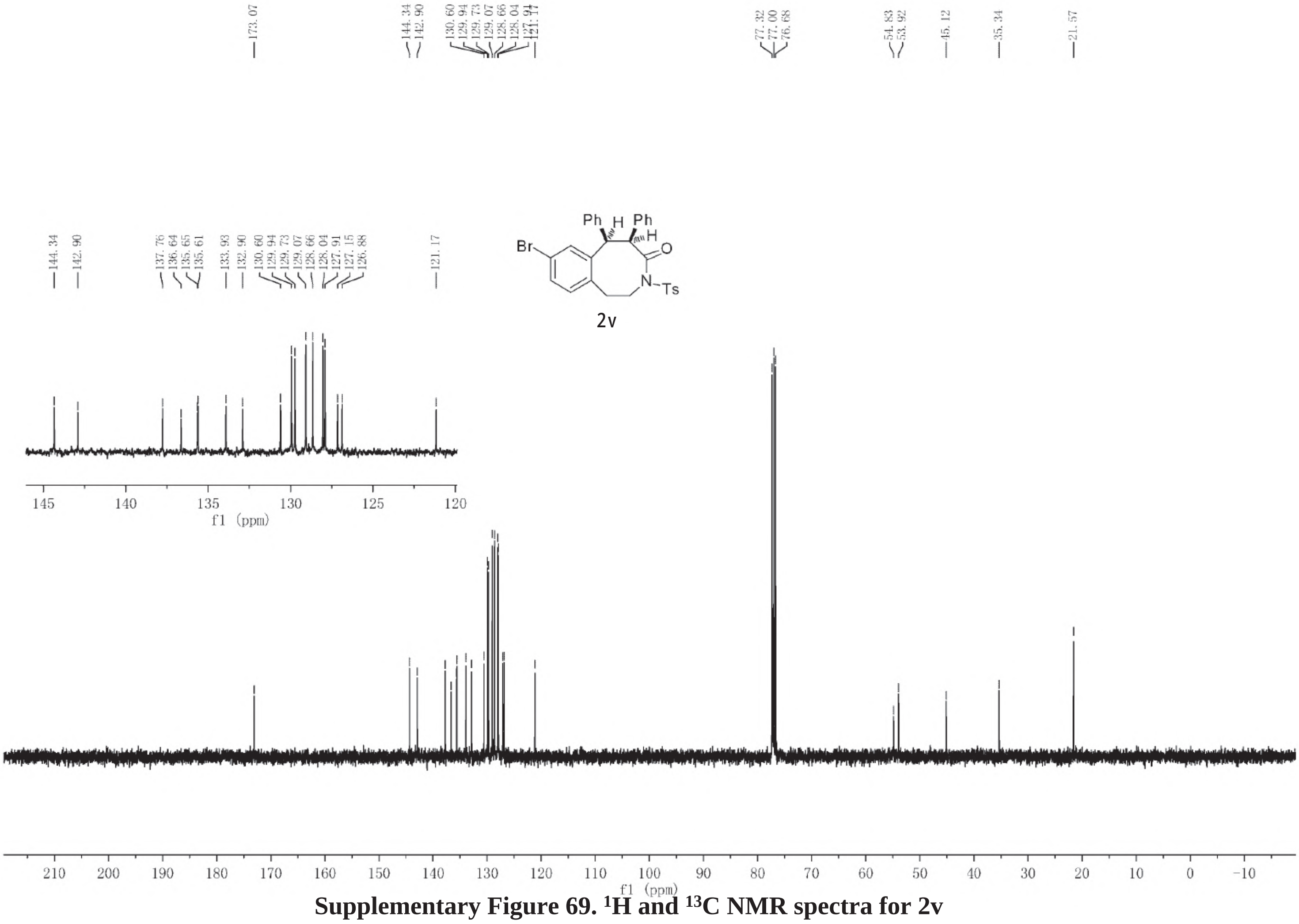

Supplementary Figure 69. ^{1}H and ^{13}C NMR spectra for 2v

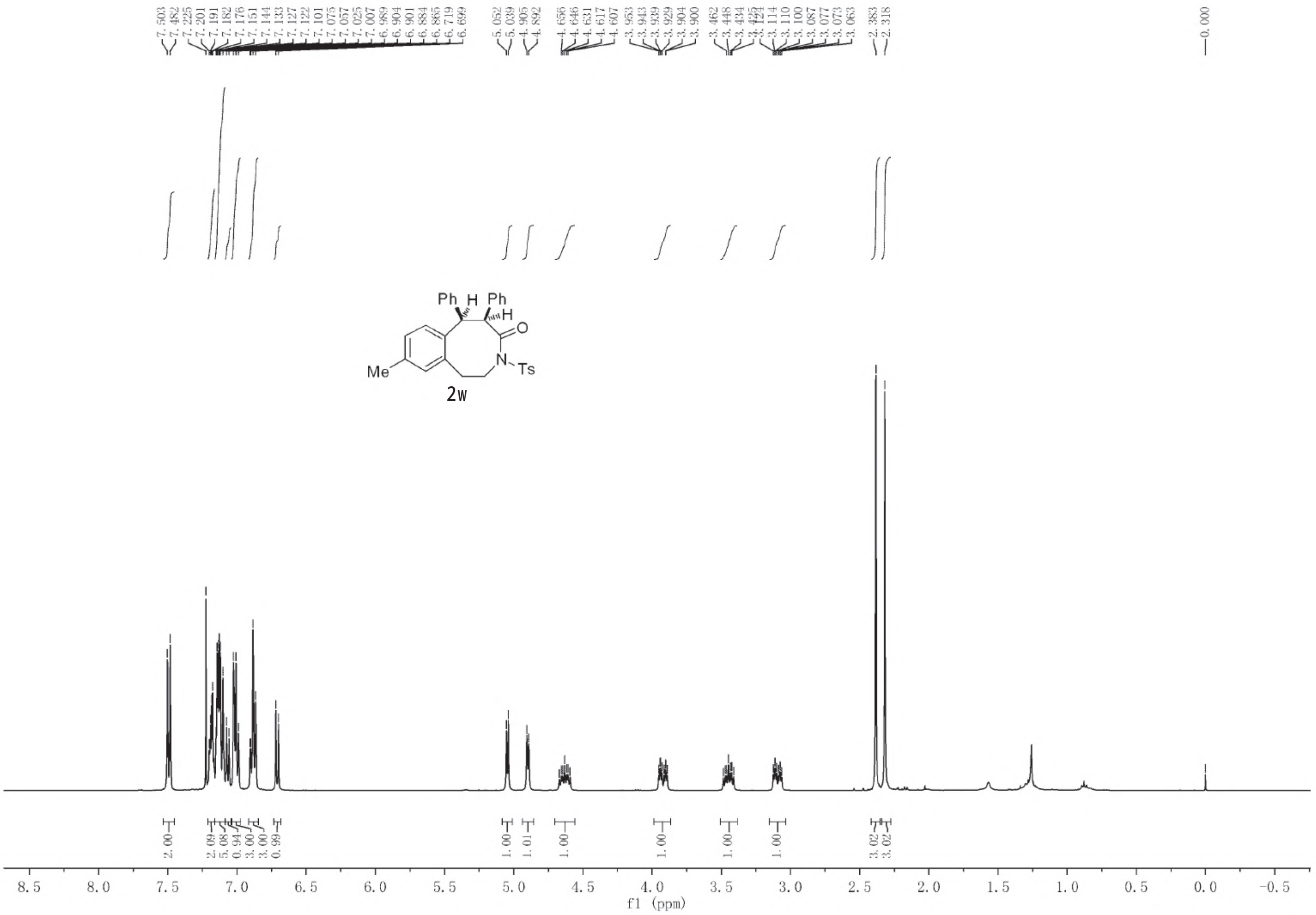

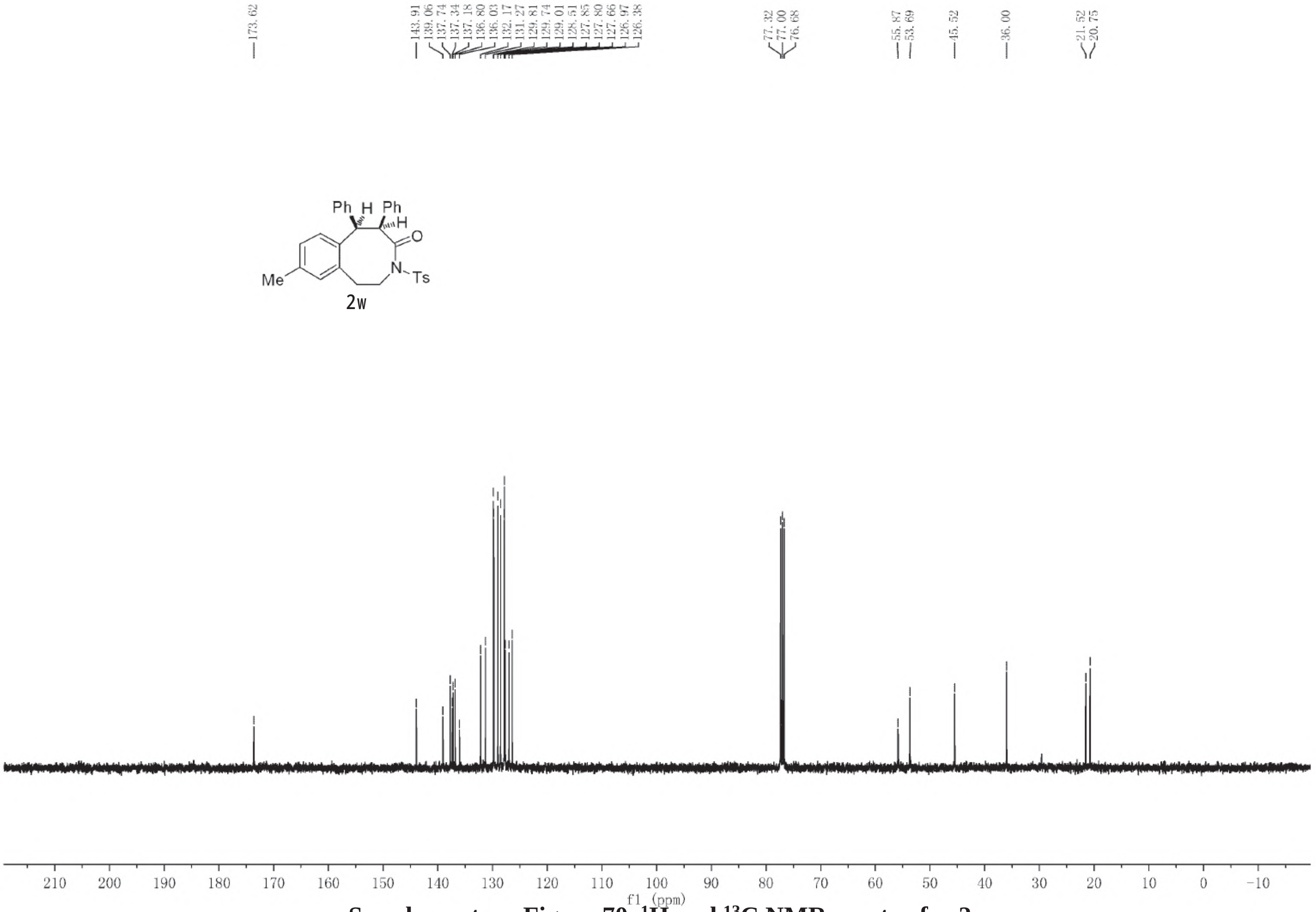

Supplementary Figure 70. ^{1}H and ^{13}C NMR spectra for 2w

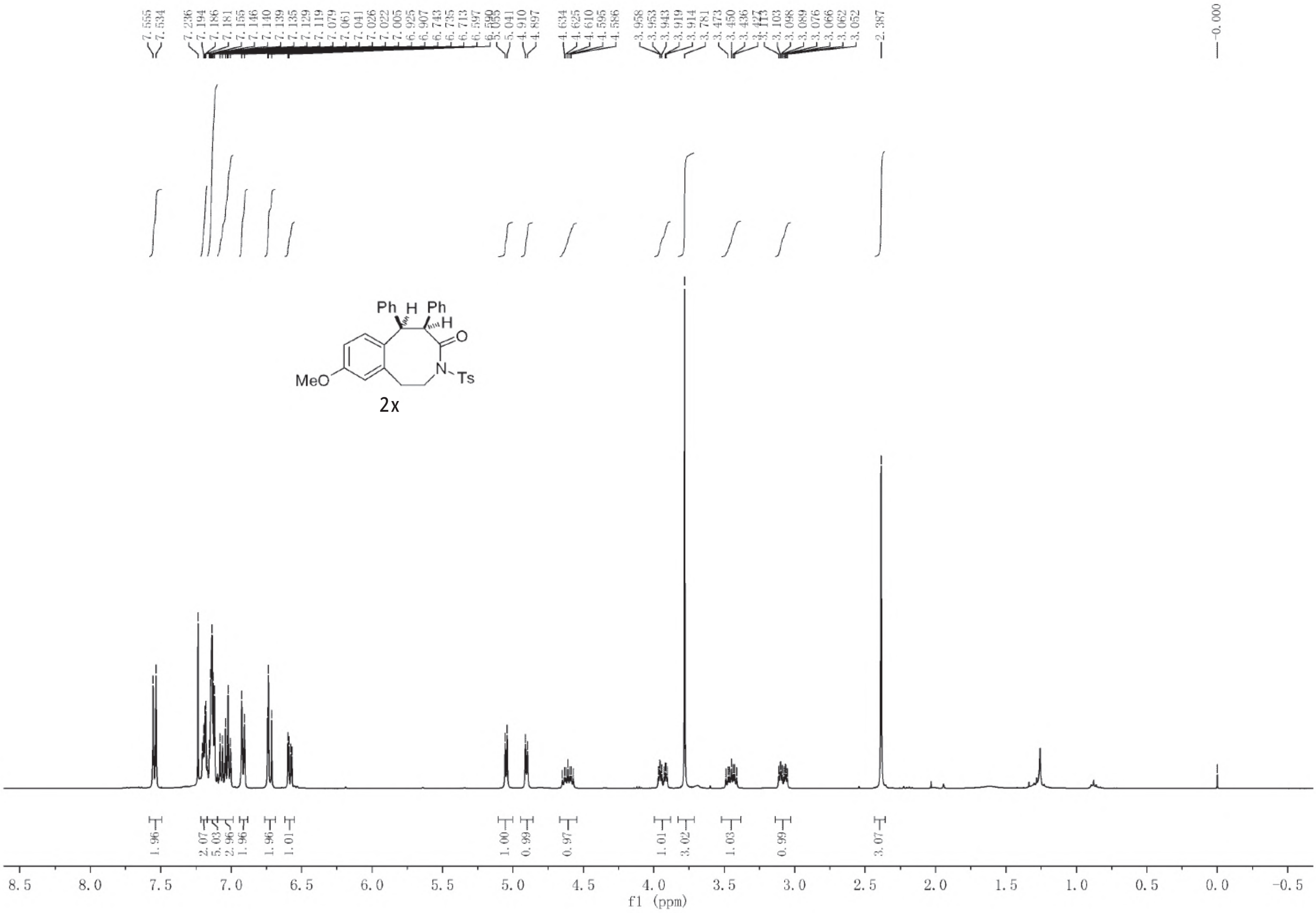

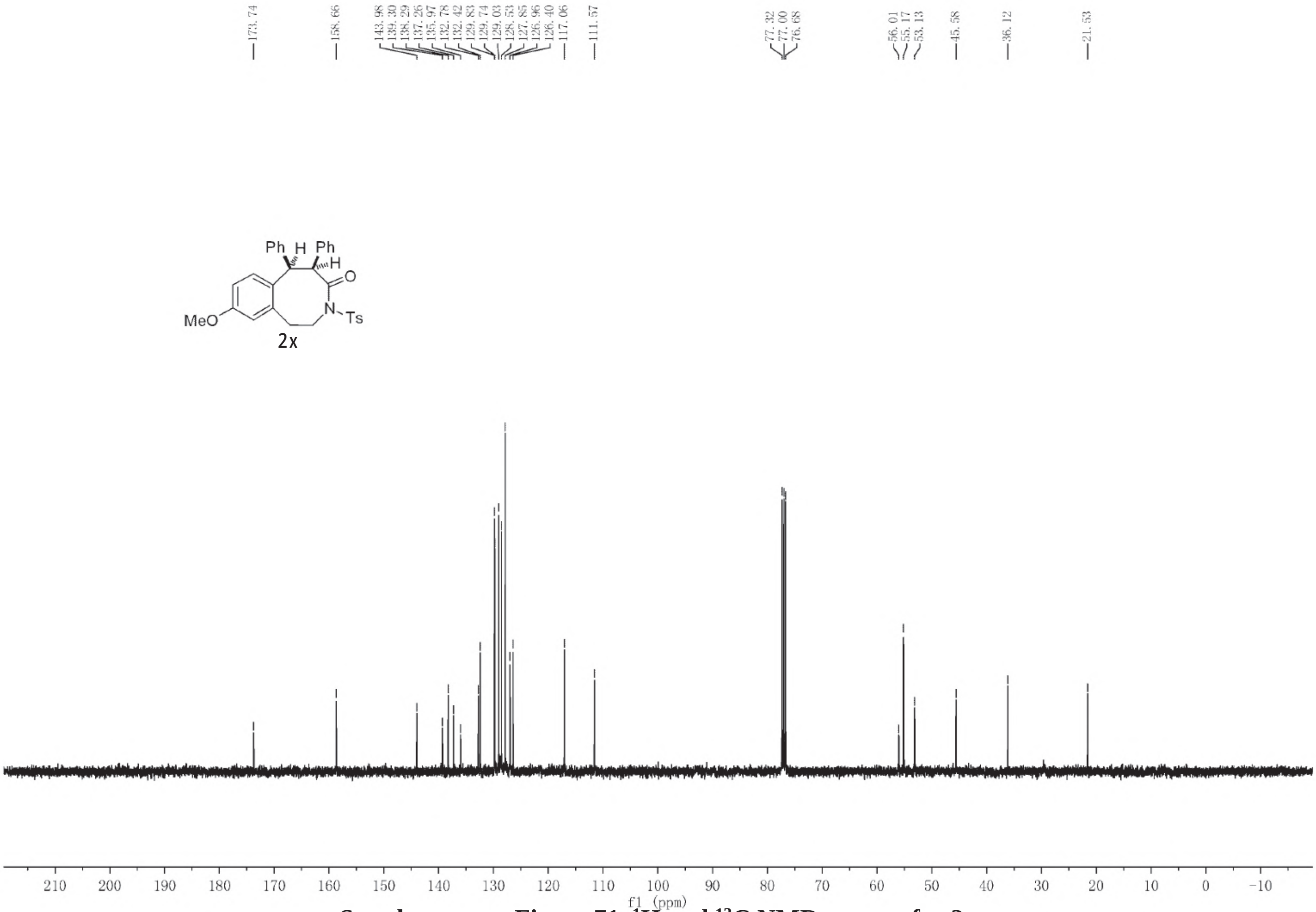

Supplementary Figure 71. ^{1}H and ^{13}C NMR spectra for 2x

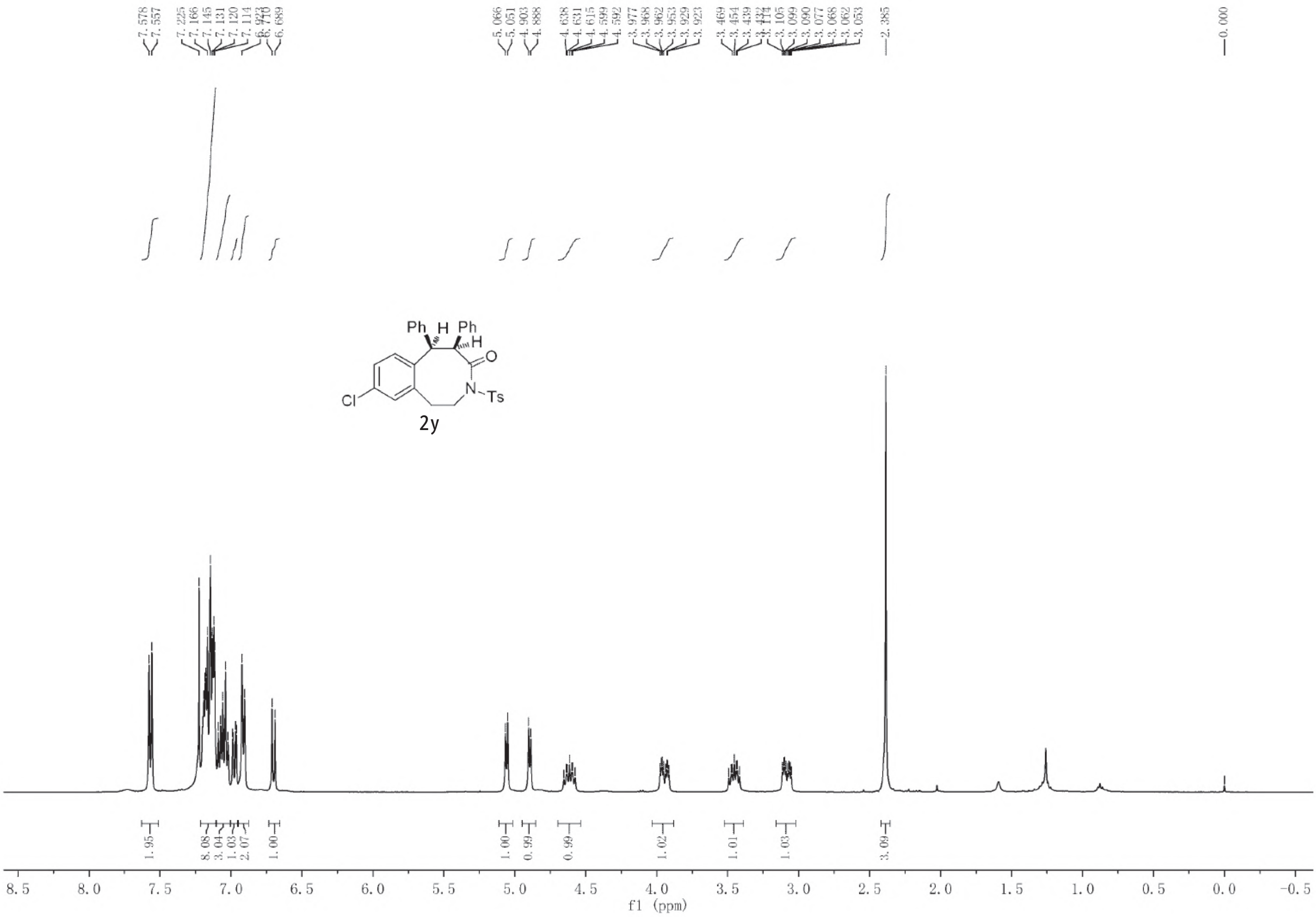

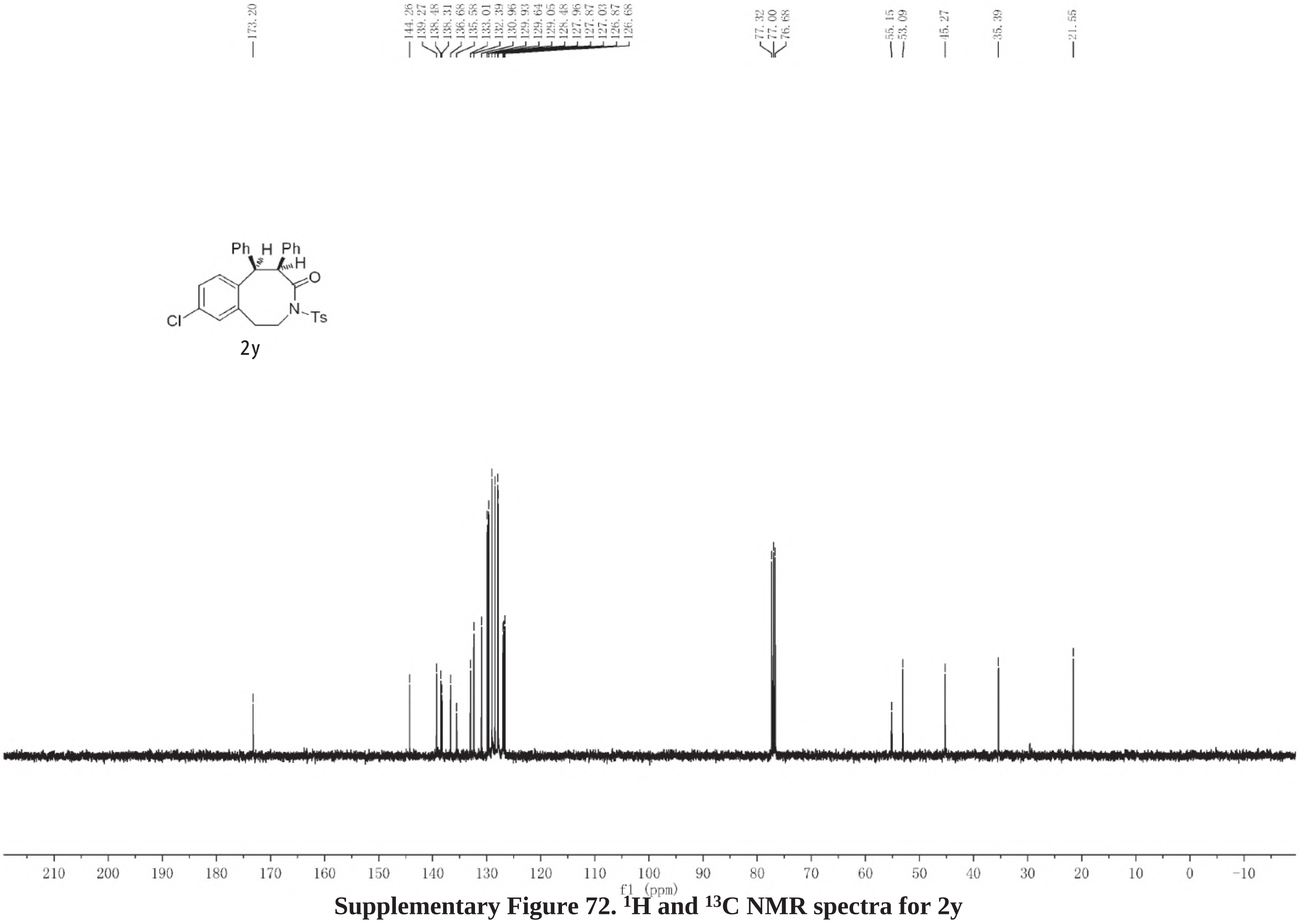

Supplementary Figure 72. ^{1}H and ^{13}C NMR spectra for 2y

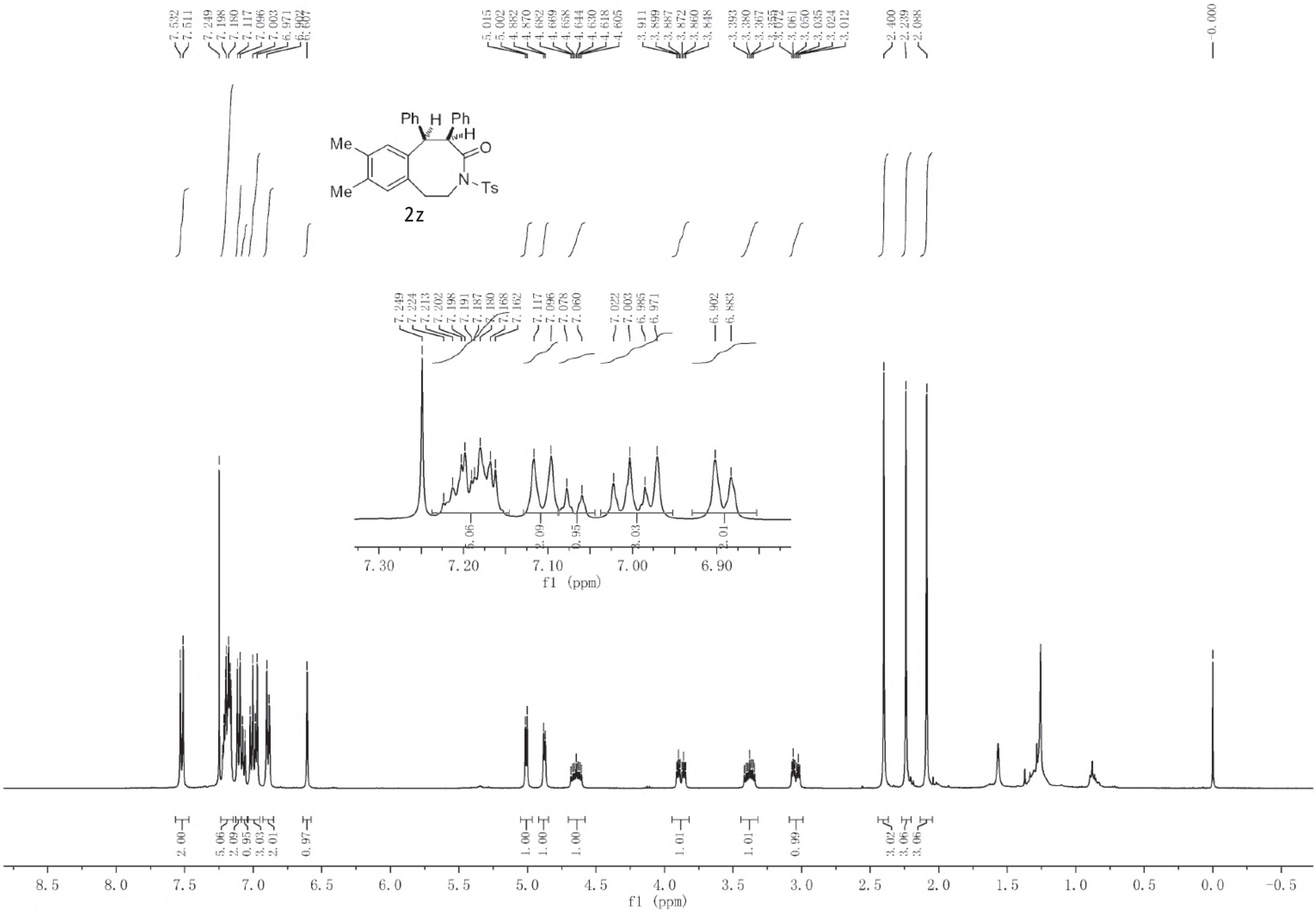

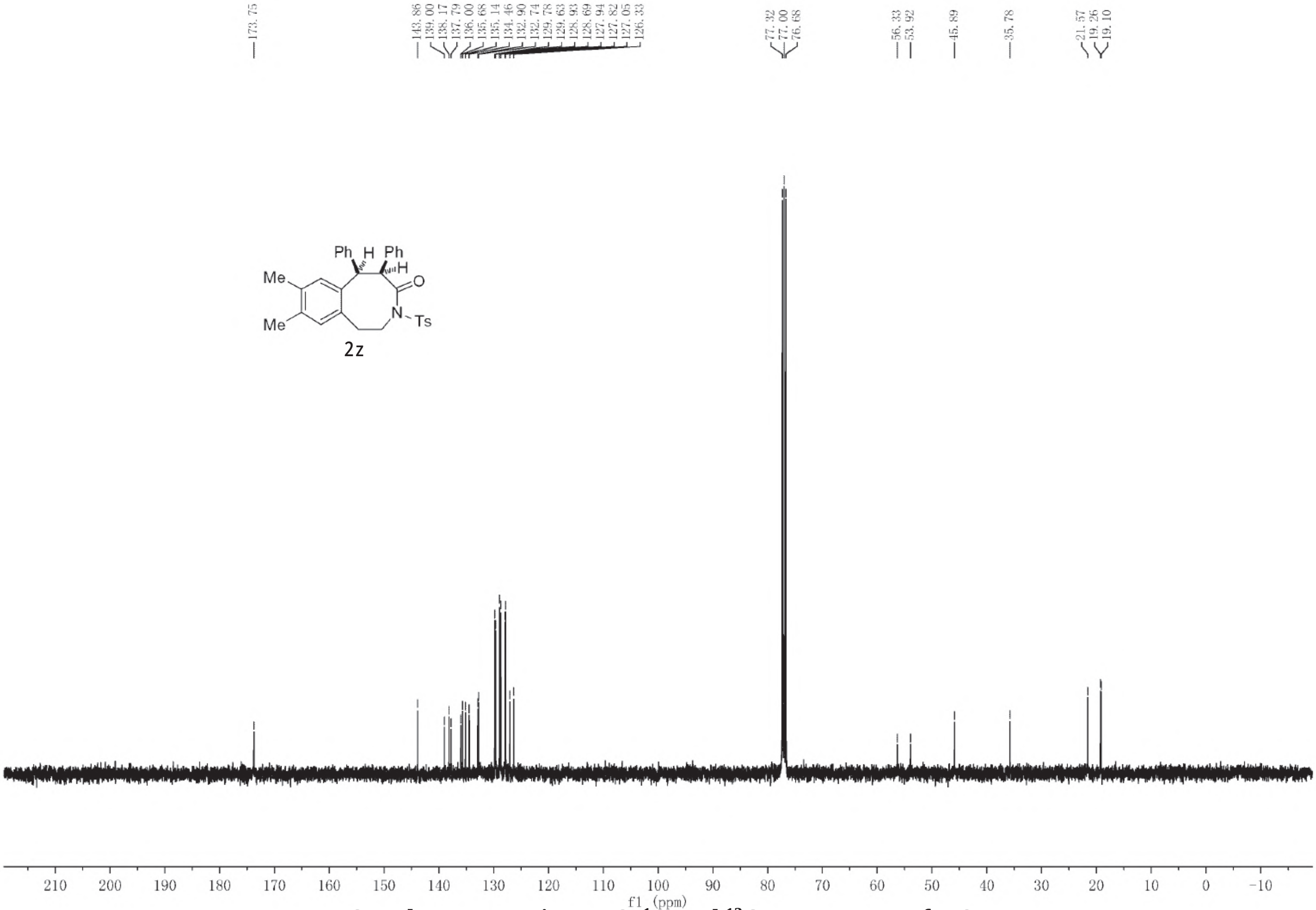

Supplementary Figure 73. ^{1}H and ^{13}C NMR spectra for 2z

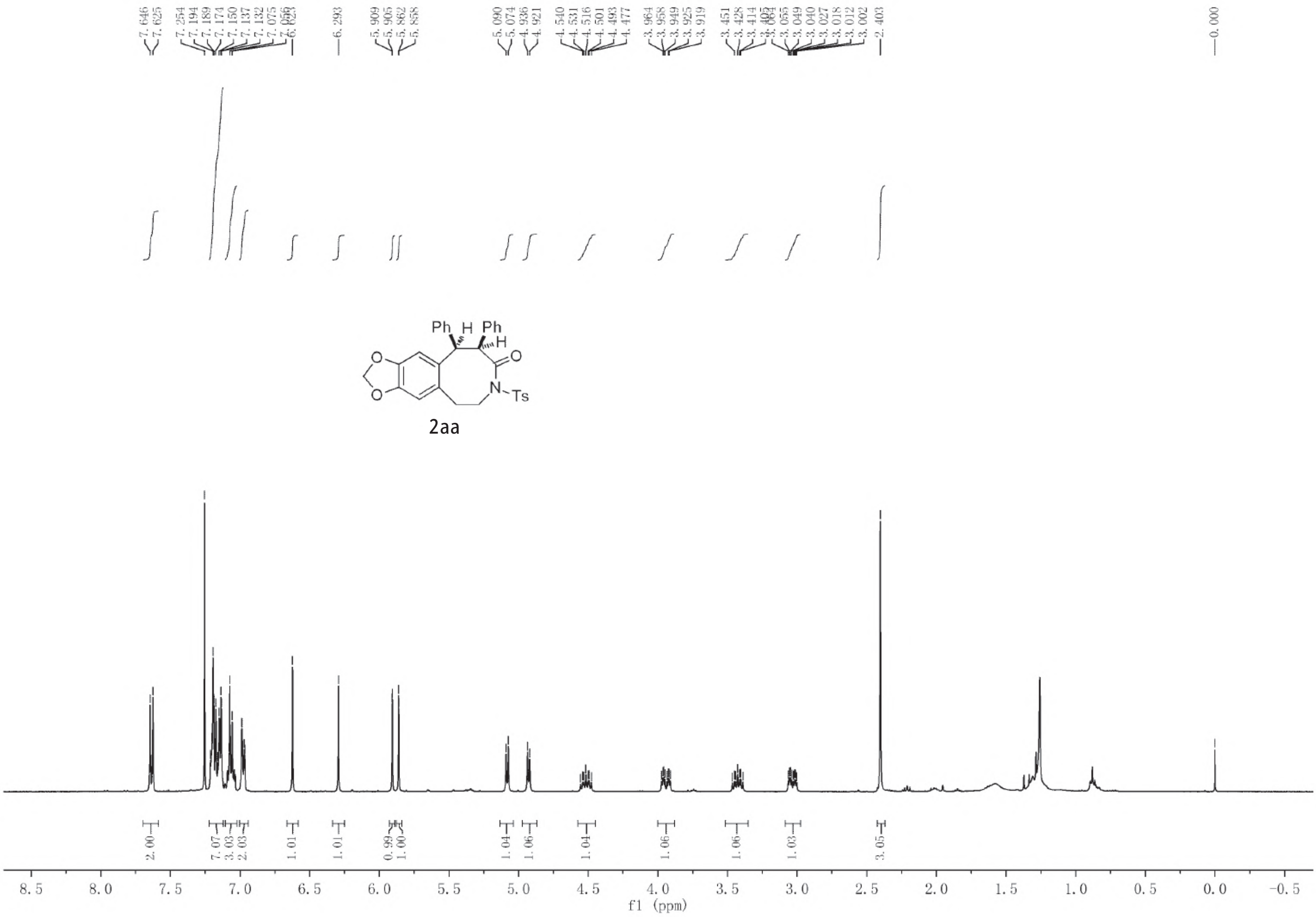

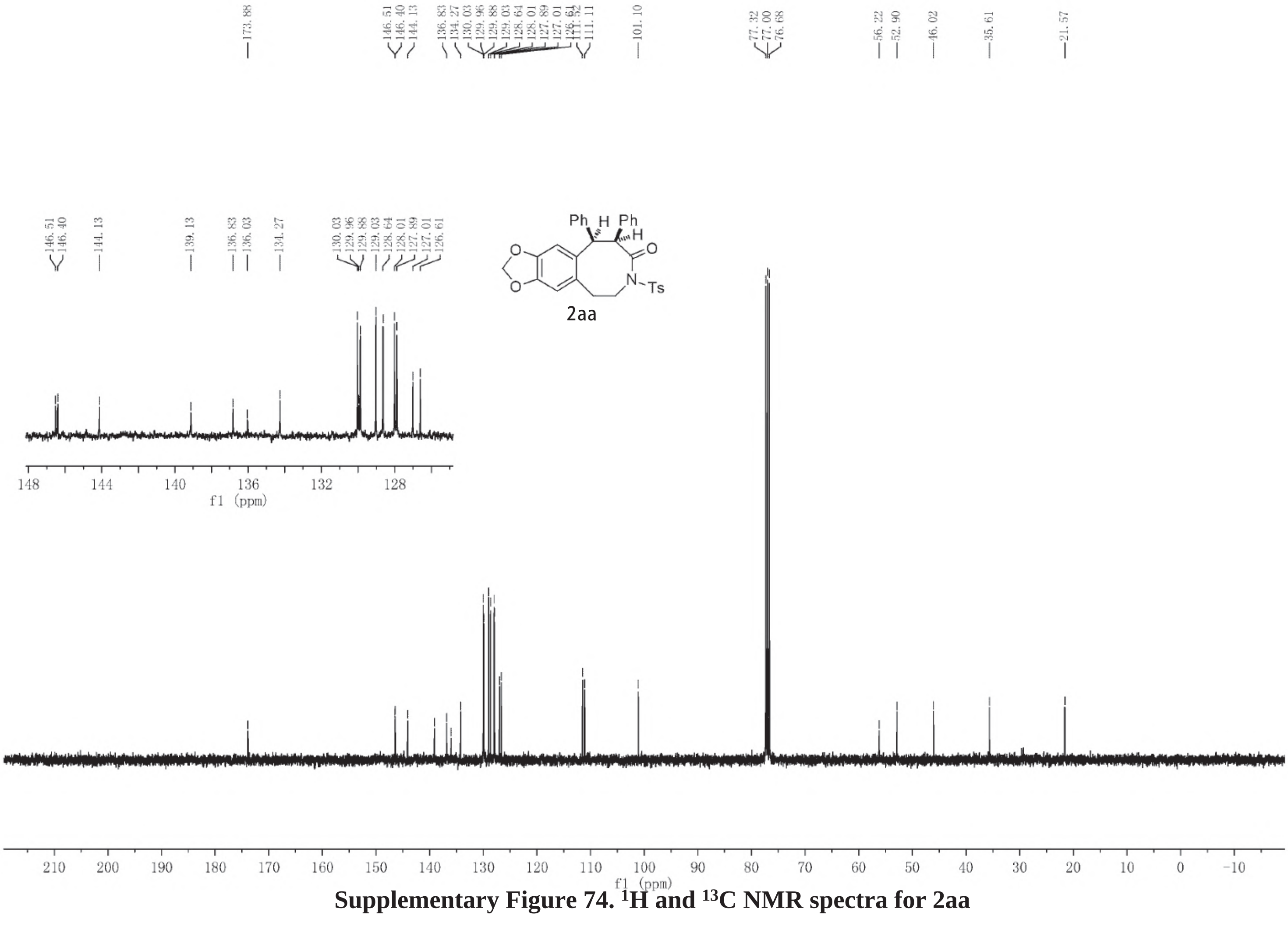

Supplementary Figure 74. ^{1}H and ^{13}C NMR spectra for 2aa

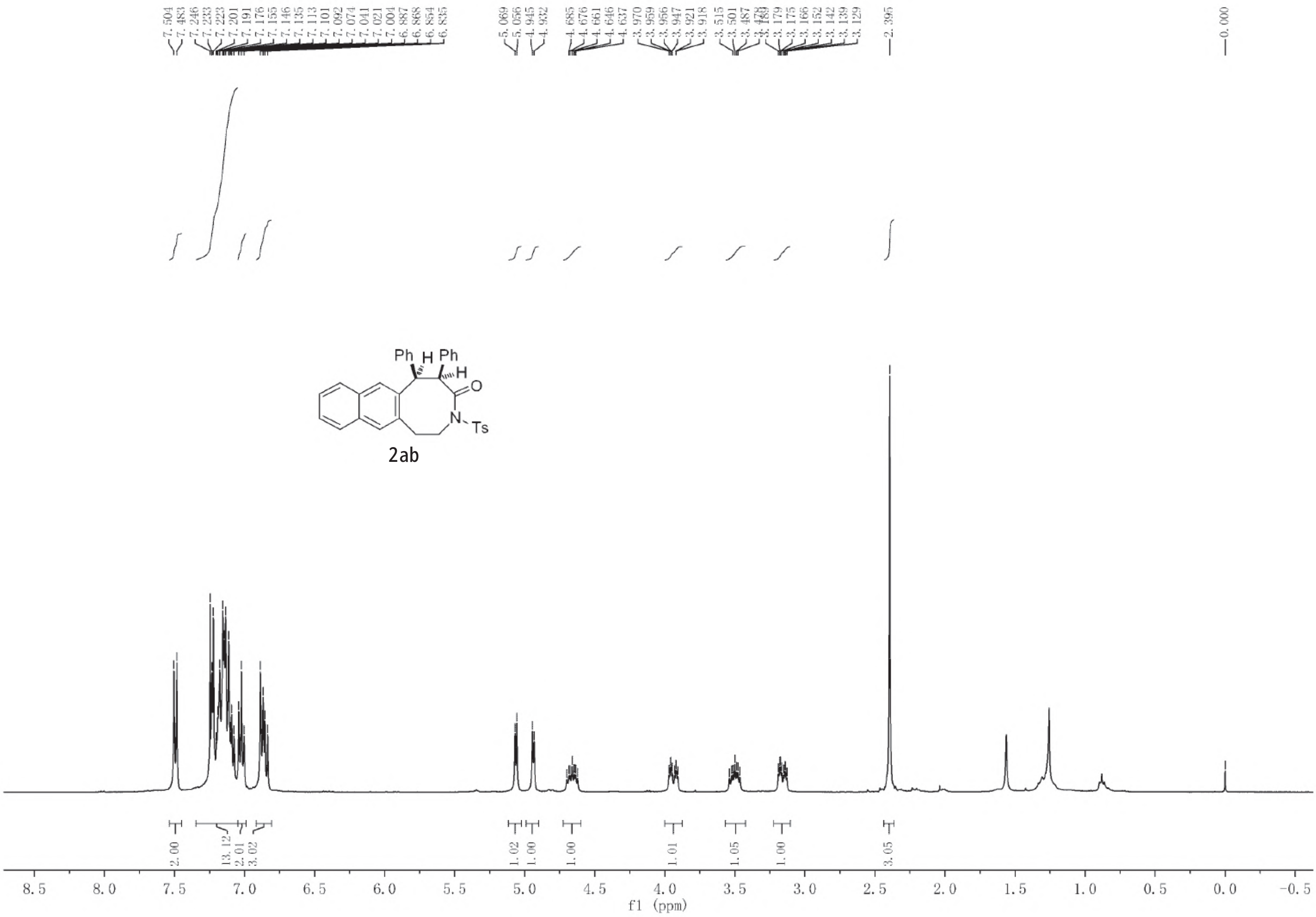

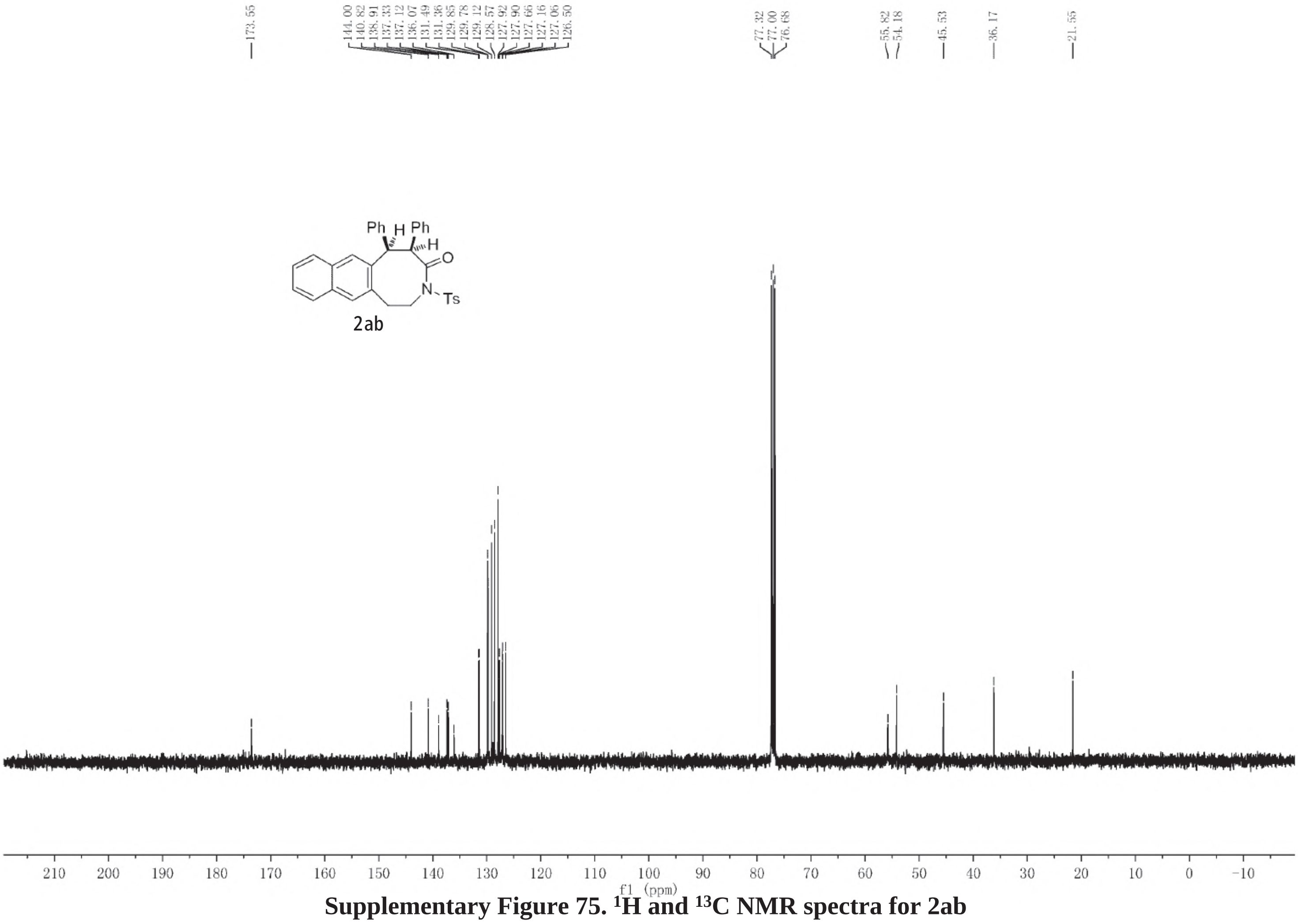

Supplementary Figure 75. ^{1}H and ^{13}C NMR spectra for 2ab

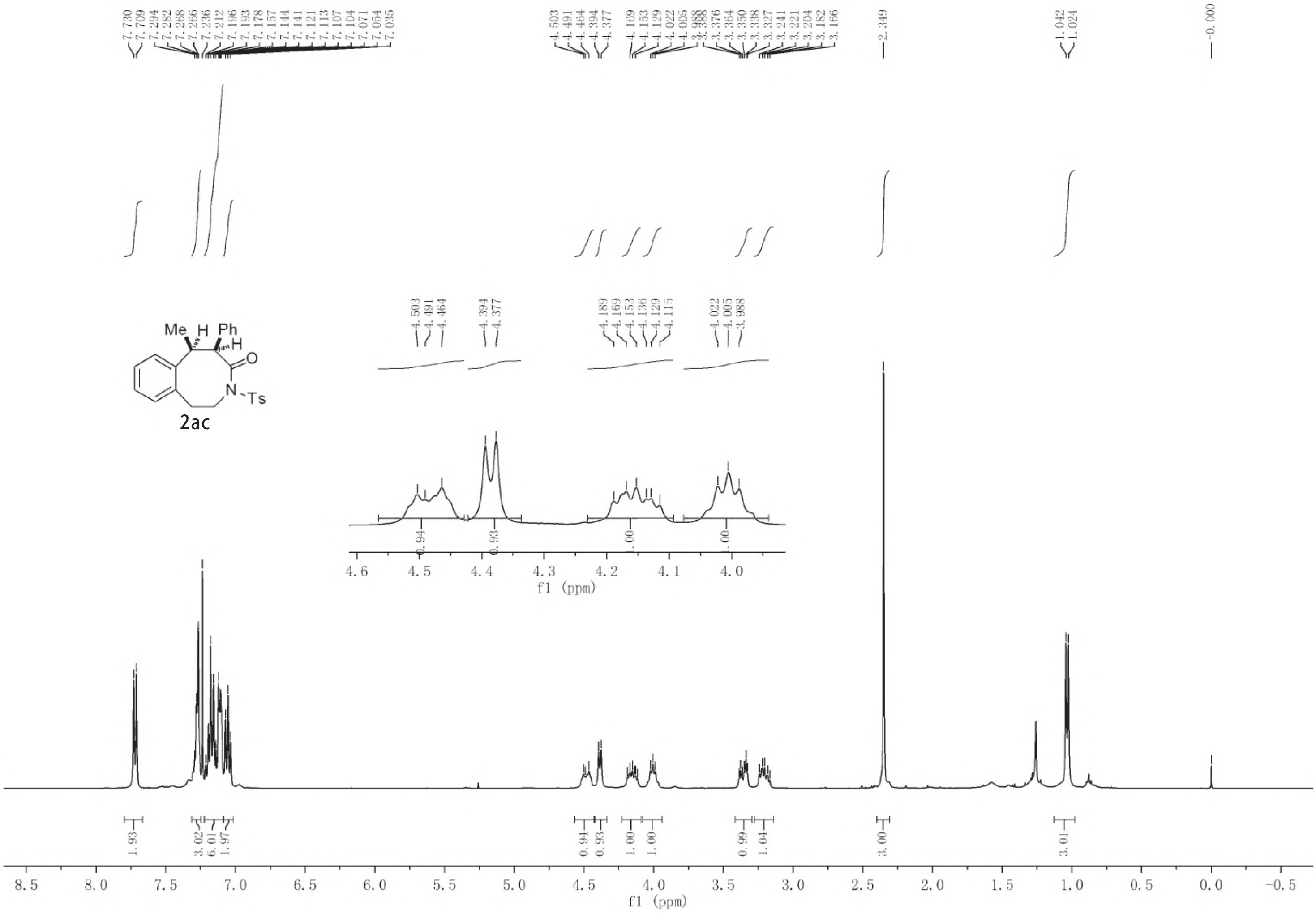

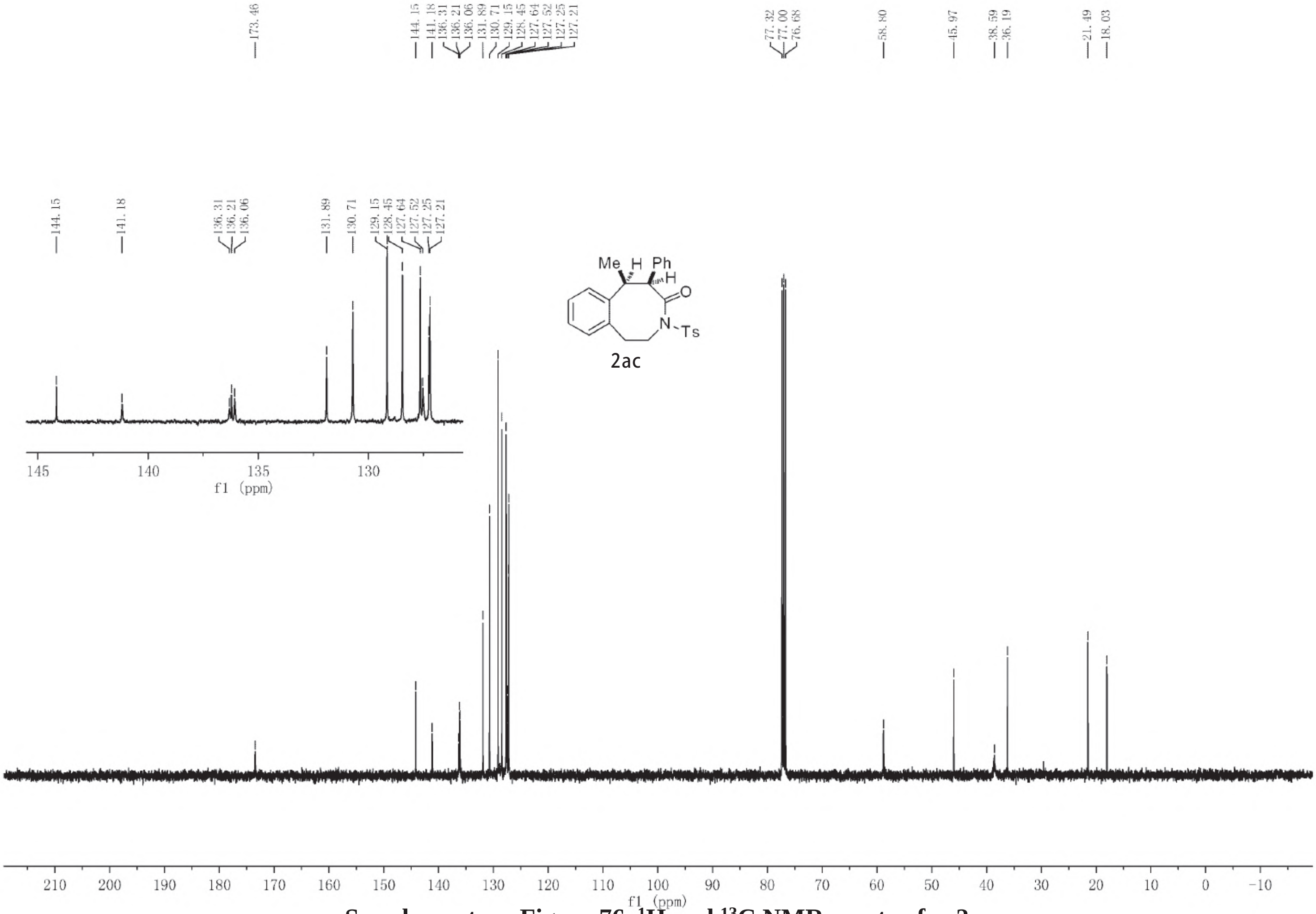

Supplementary Figure 76. ^{1}H and ^{13}C NMR spectra for 2ac

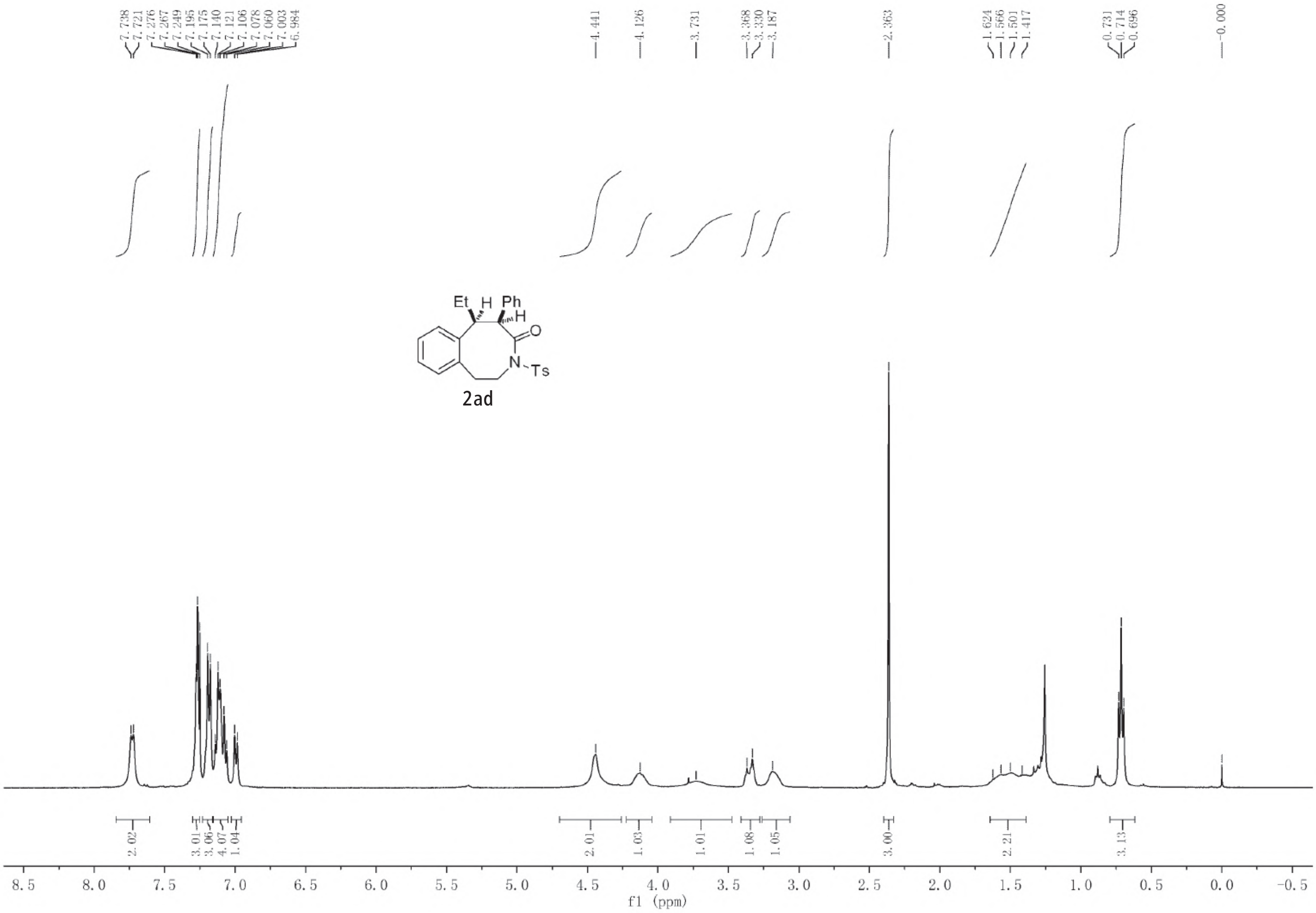

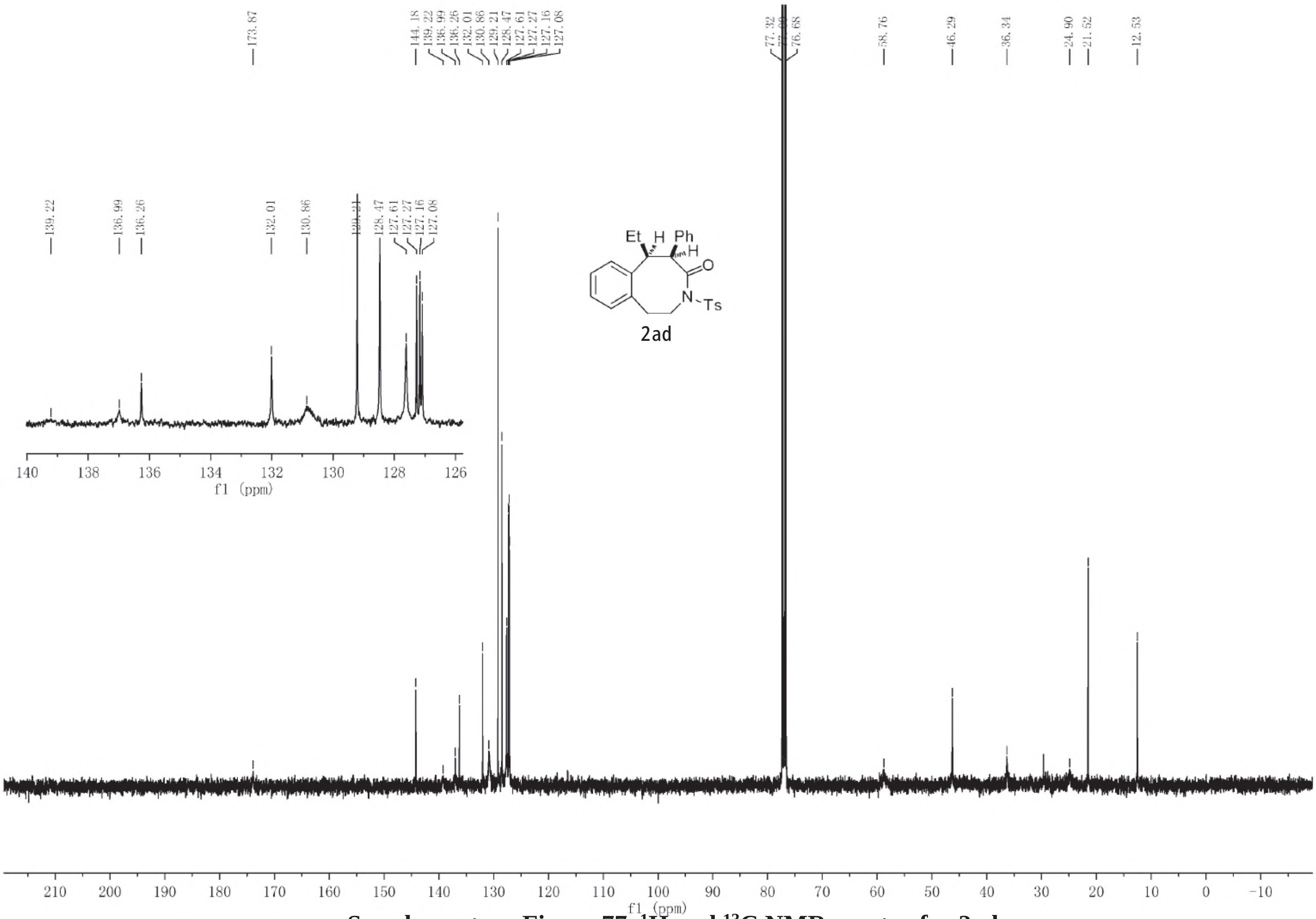

Supplementary Figure 77. ^{1}H and ^{13}C NMR spectra for 2ad

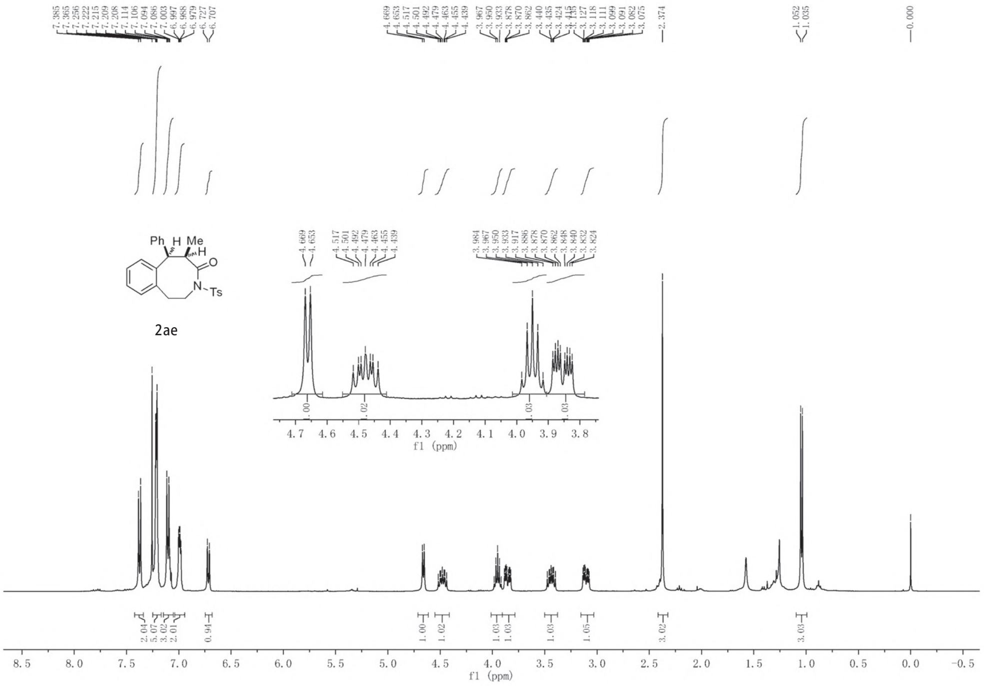

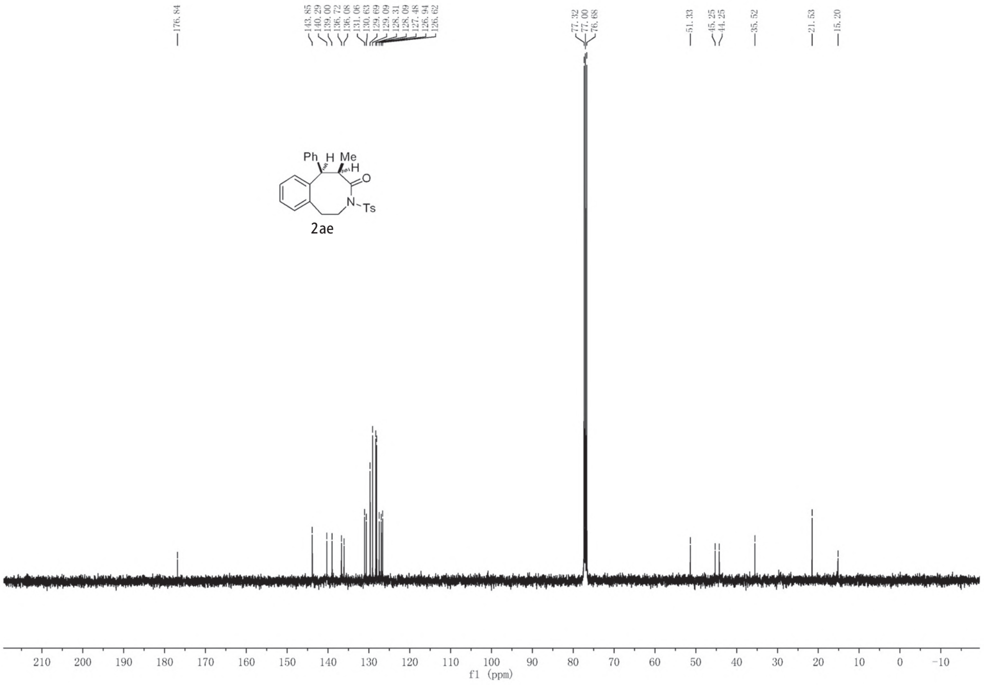

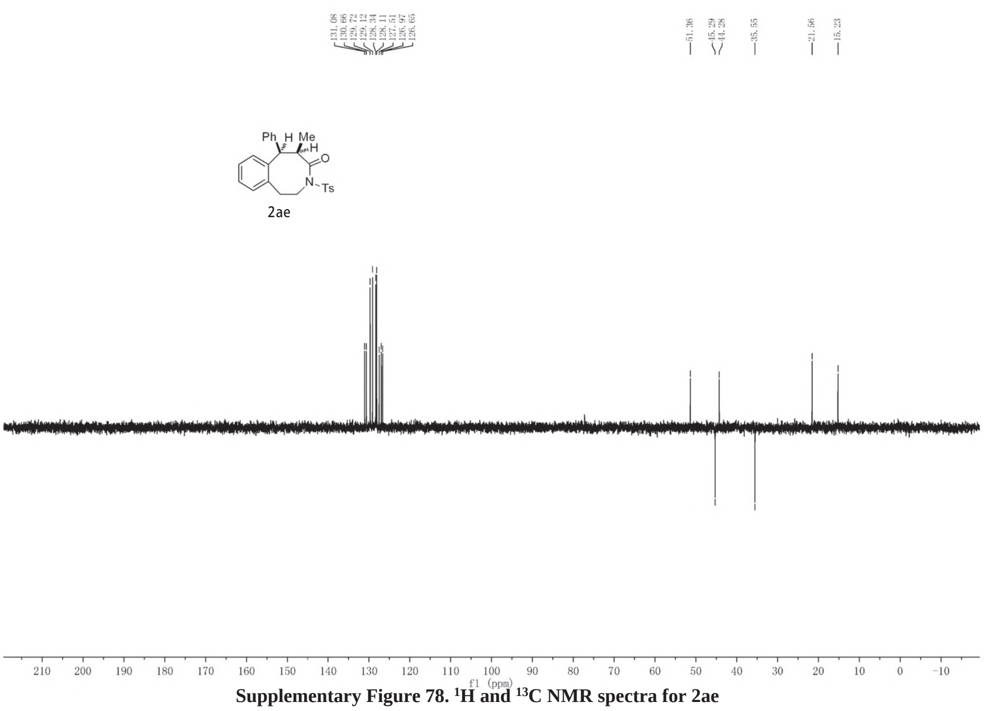

Supplementary Figure 78. ^{1}H and ^{13}C NMR spectra for 2ae

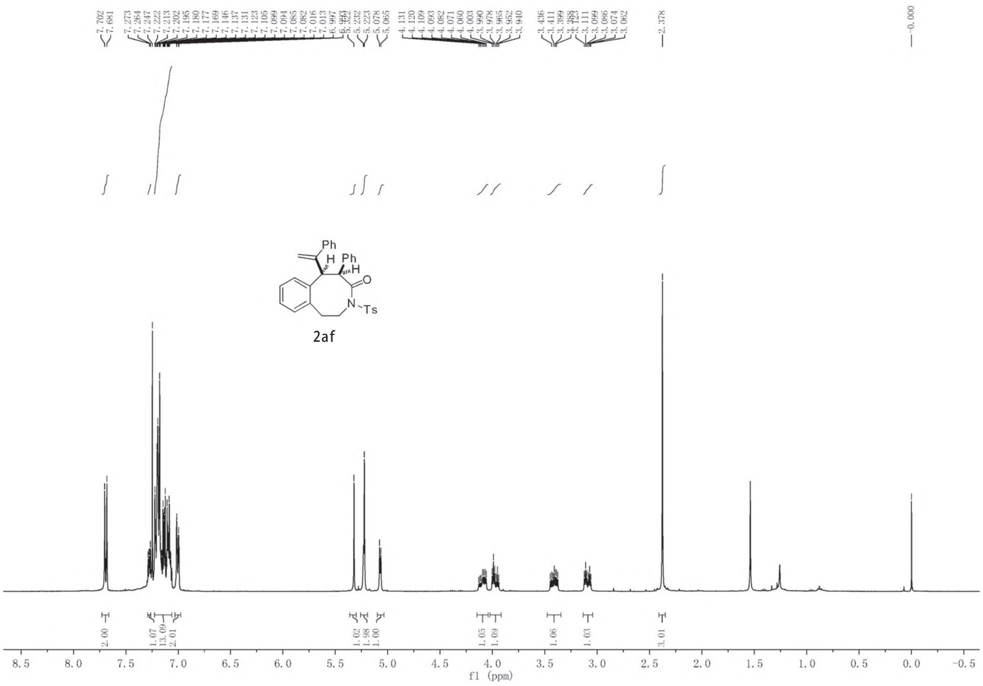

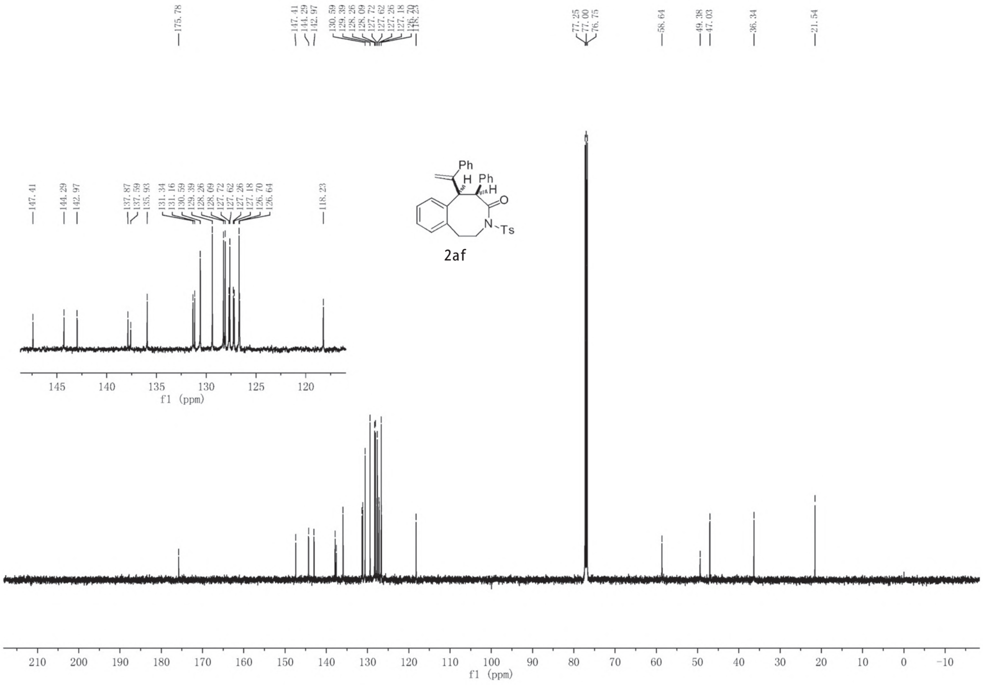

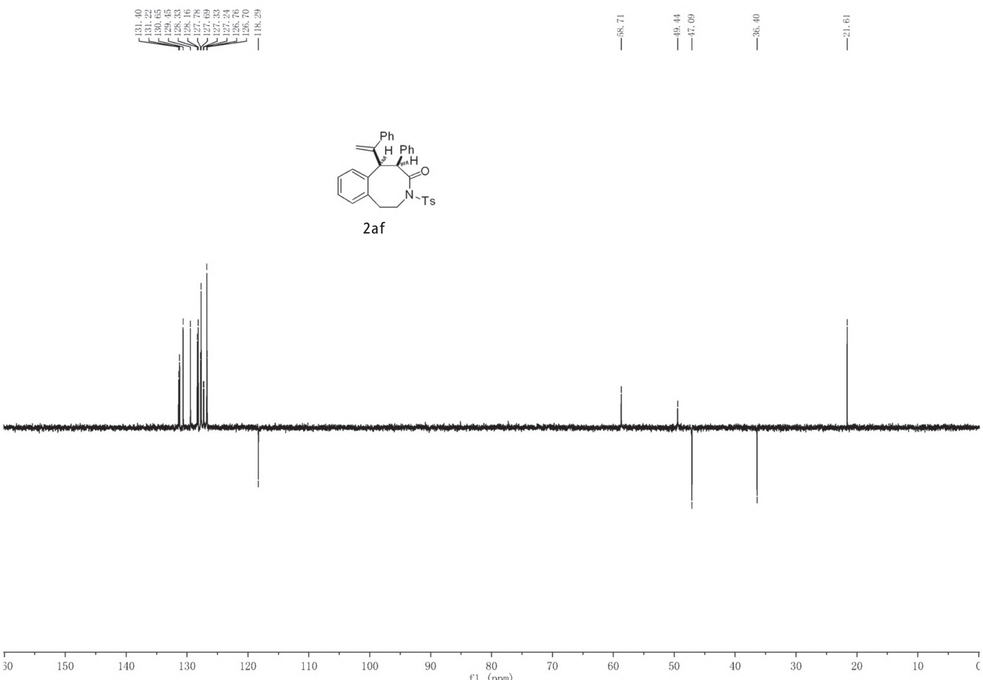

Supplementary Figure 79. ^{1}H and ^{13}C NMR spectra for 2af

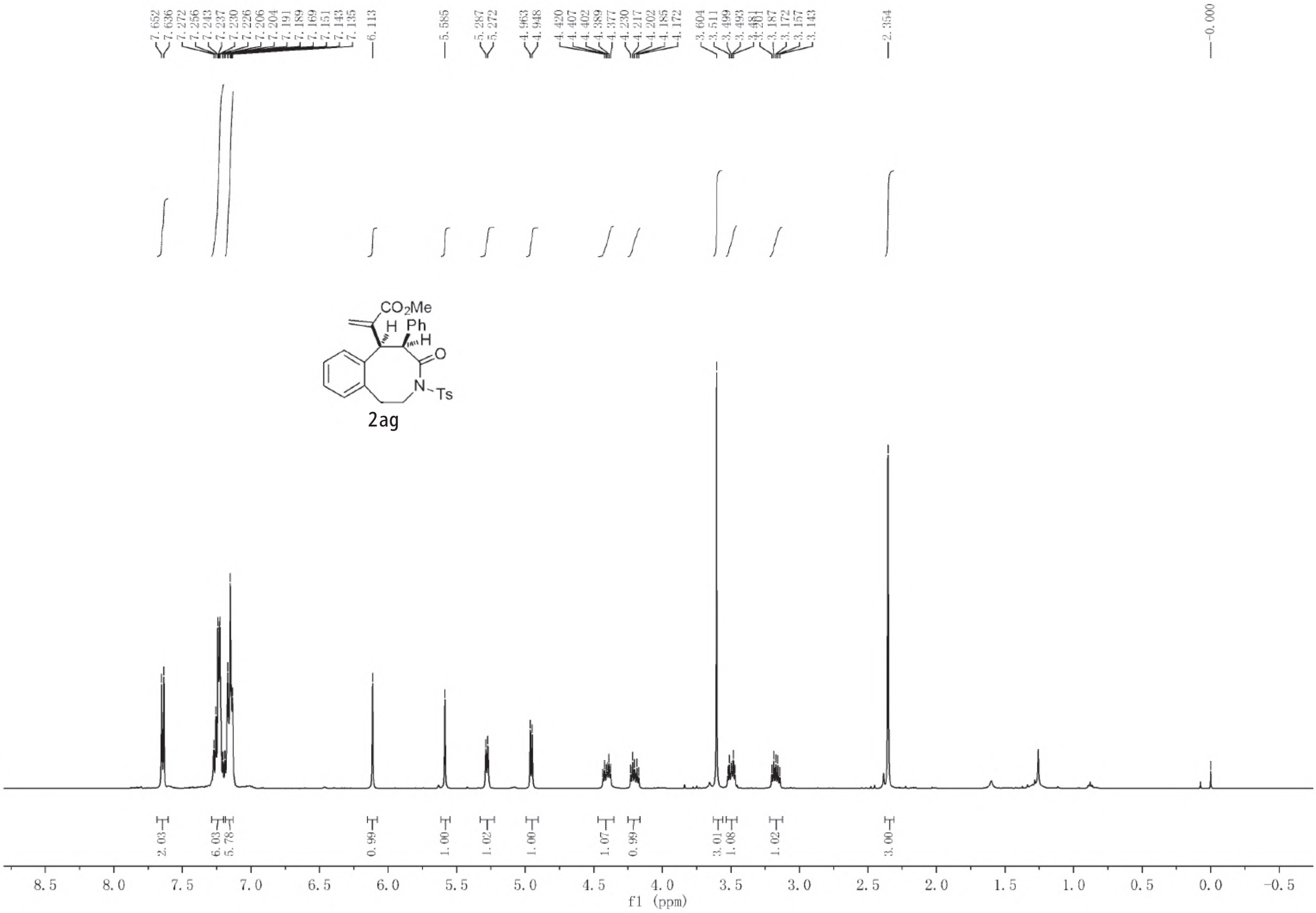

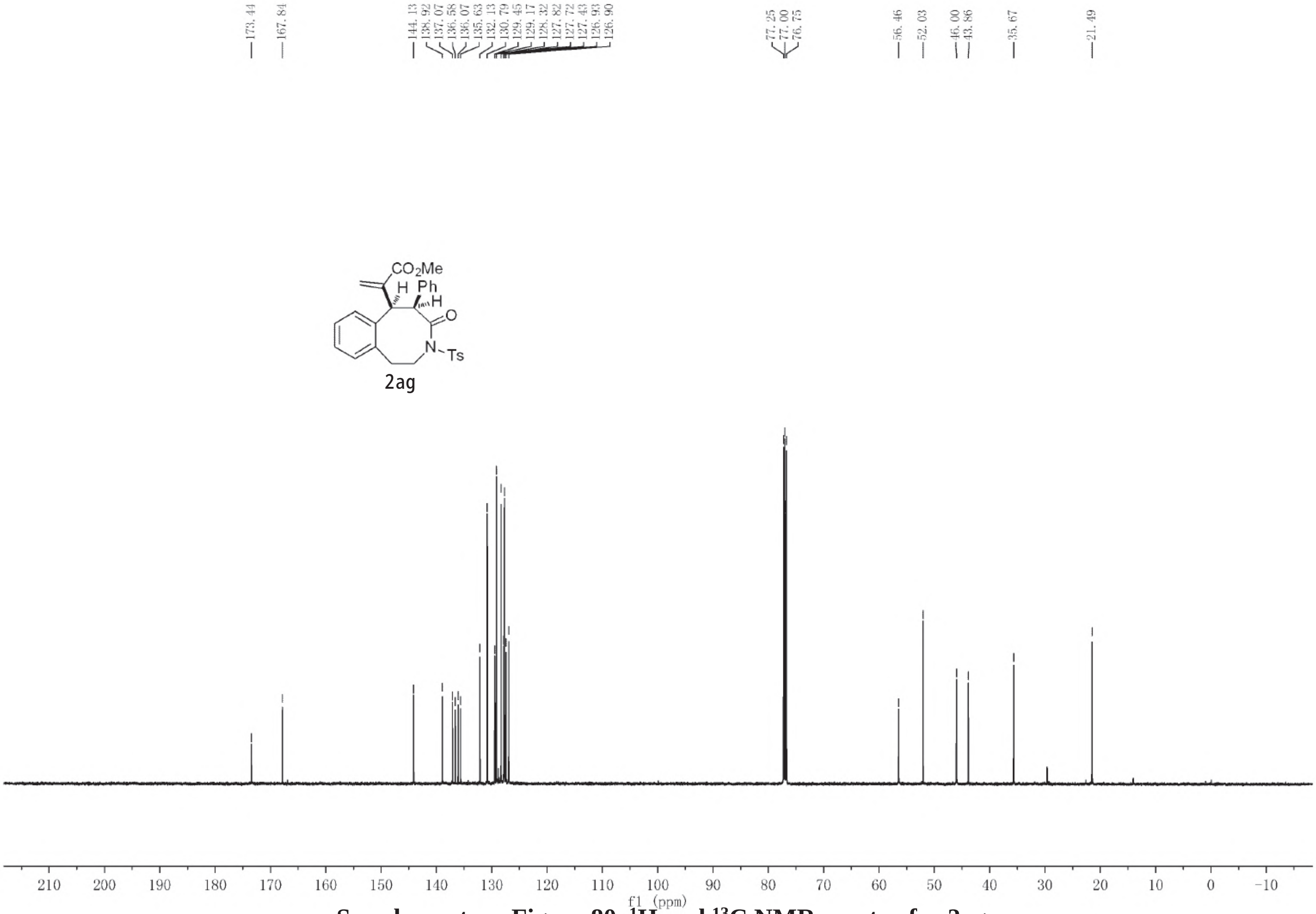

Supplementary Figure 80. ^{1}H and ^{13}C NMR spectra for 2ag

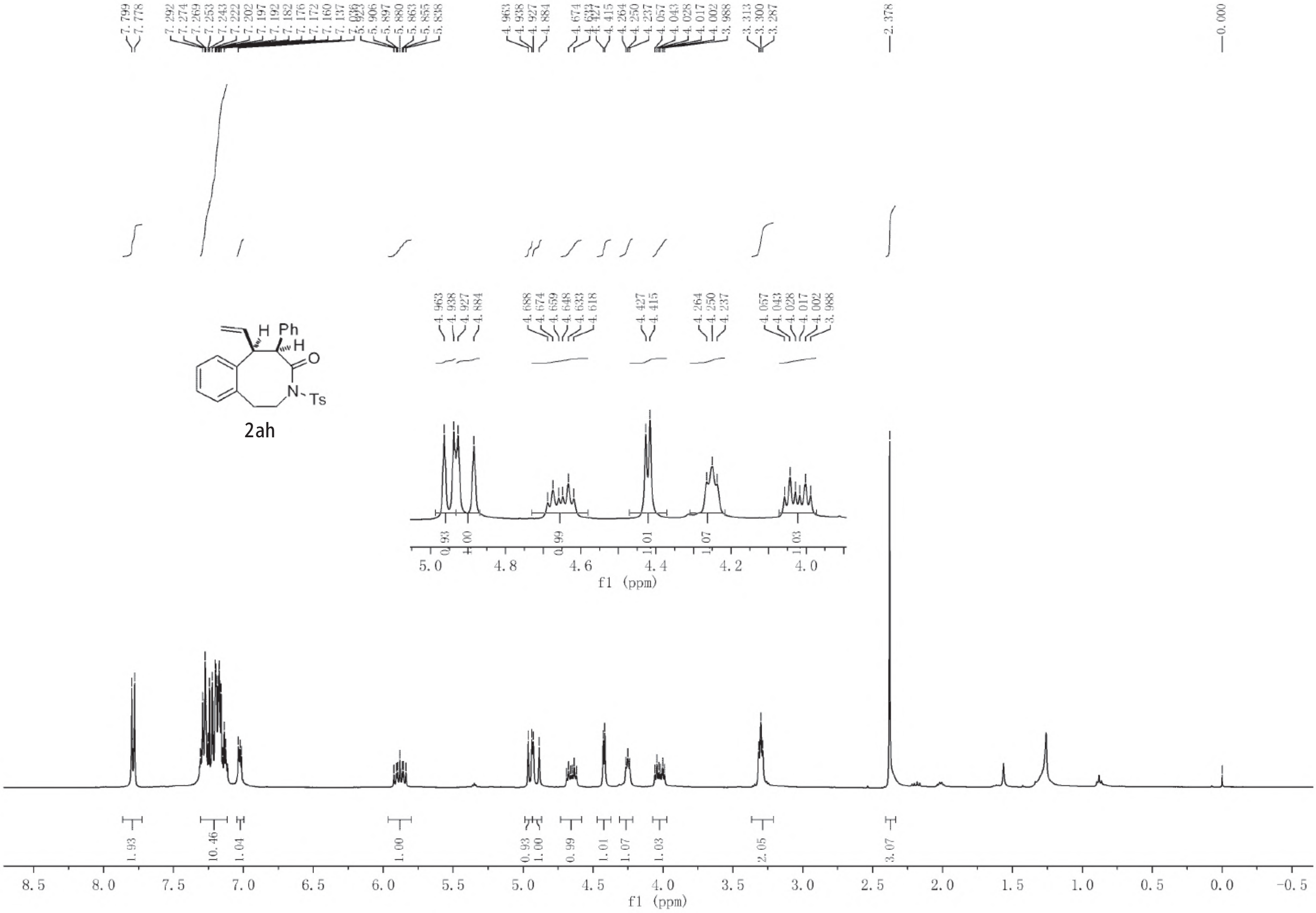

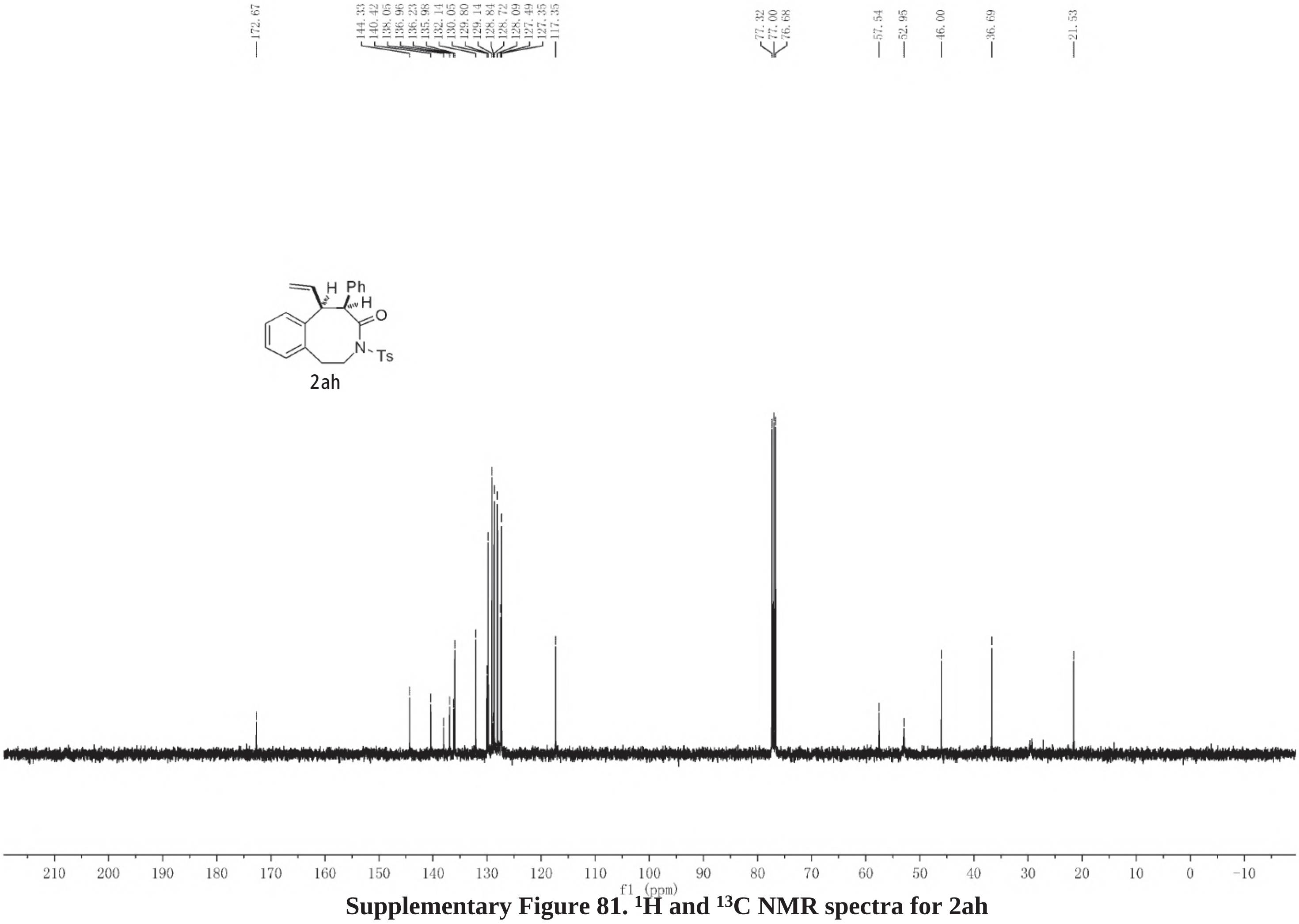

Supplementary Figure 81. ^{1}H and ^{13}C NMR spectra for 2ah

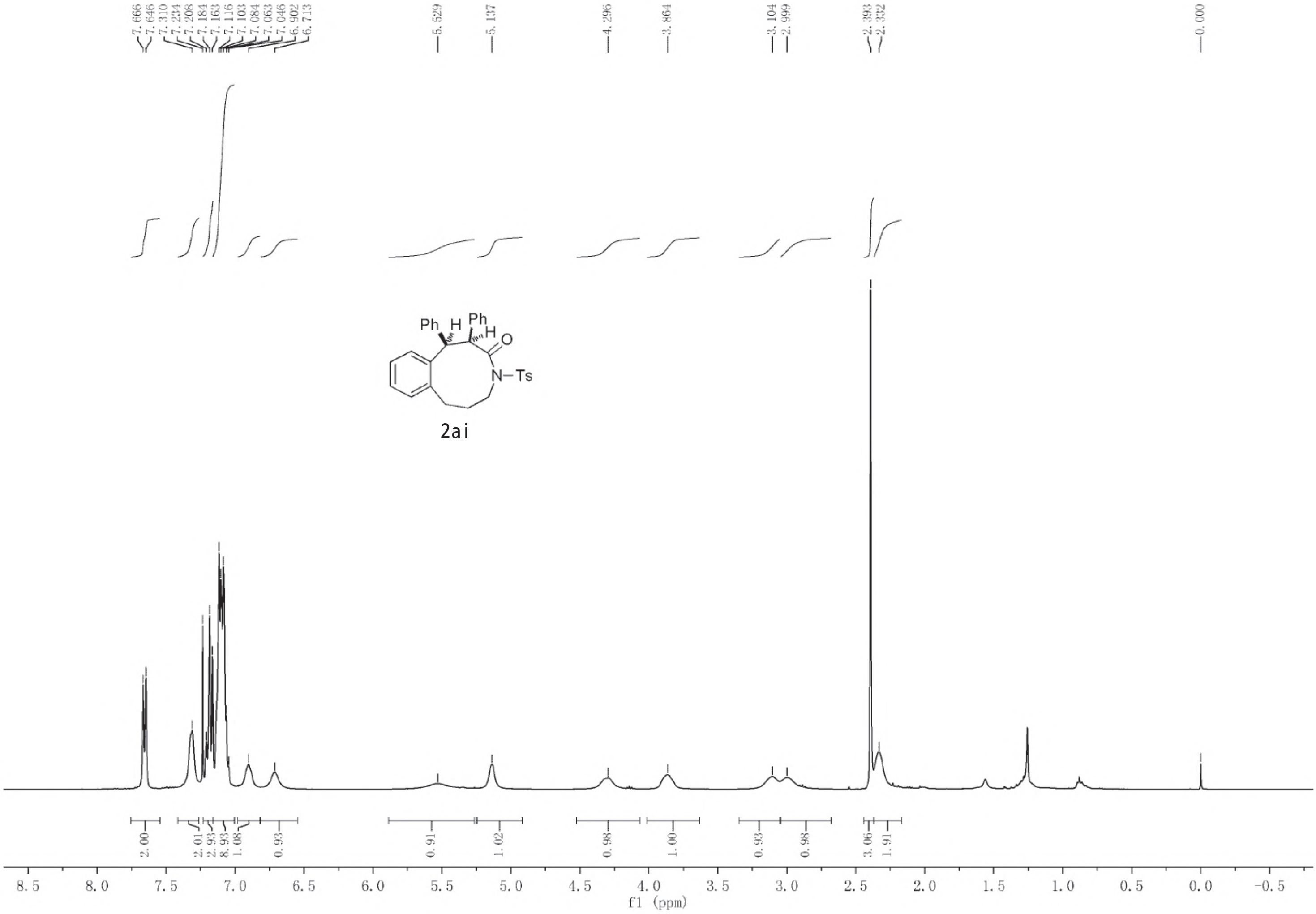

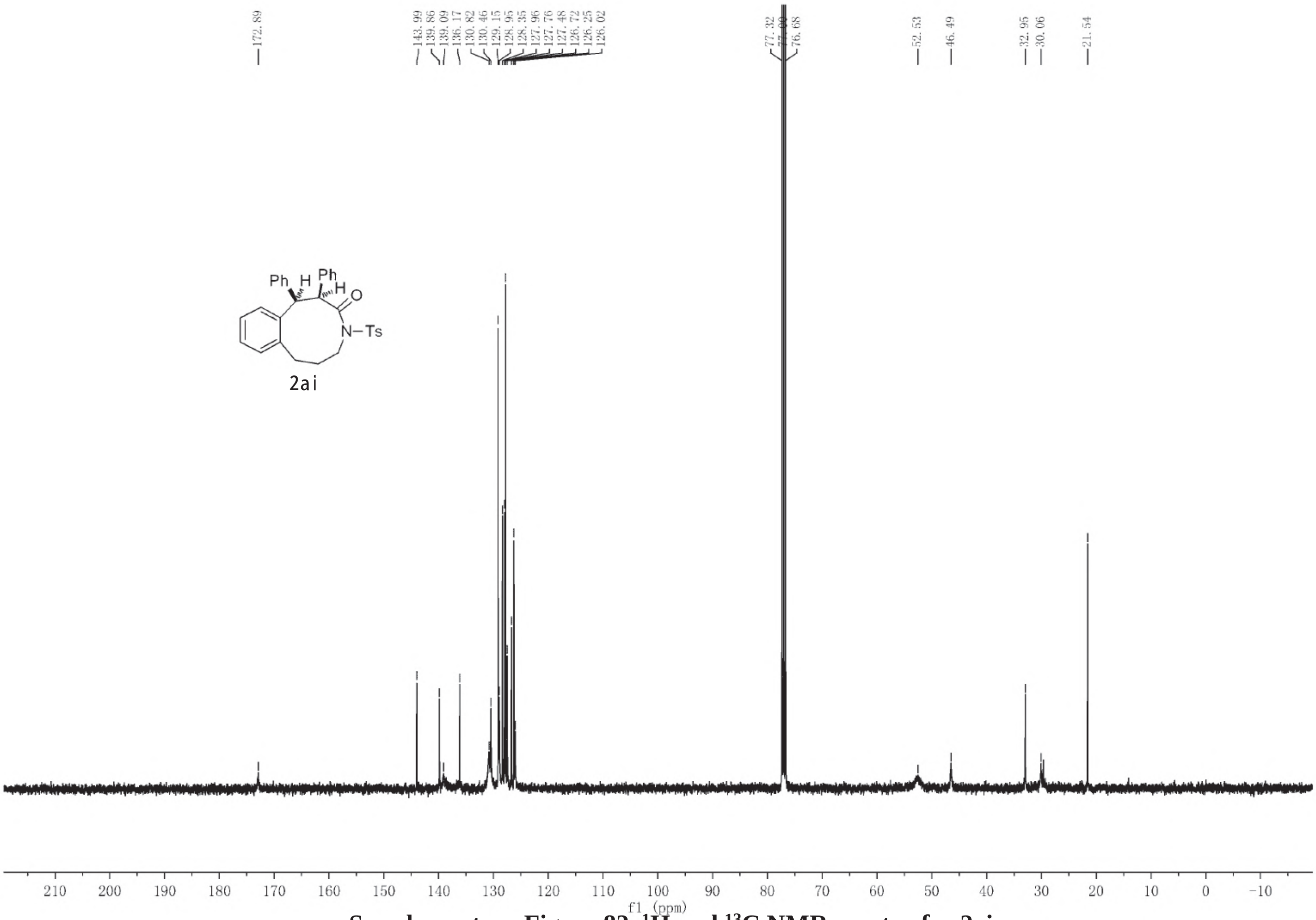

Supplementary Figure 82. ^{1}H and ^{13}C NMR spectra for 2ai

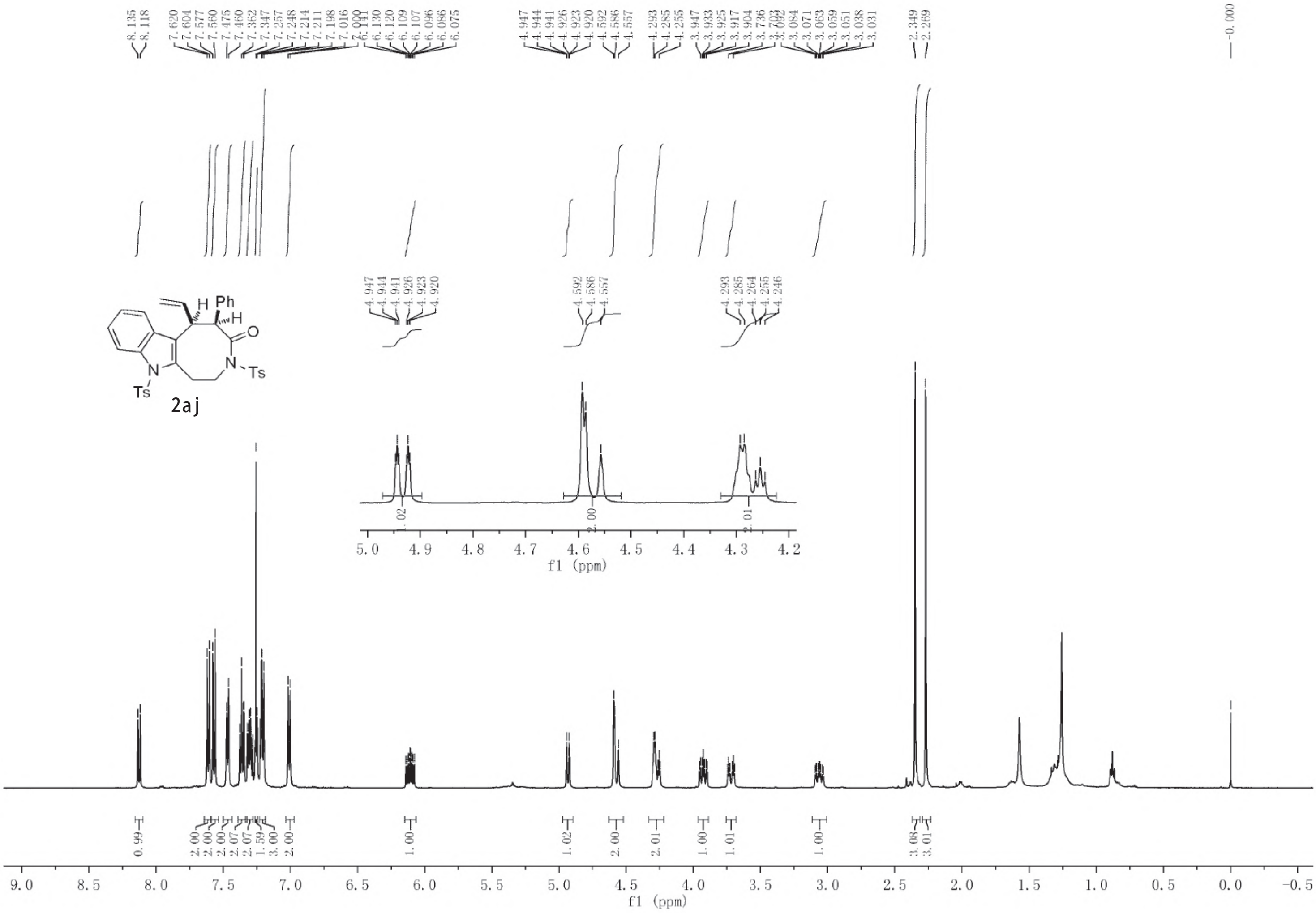

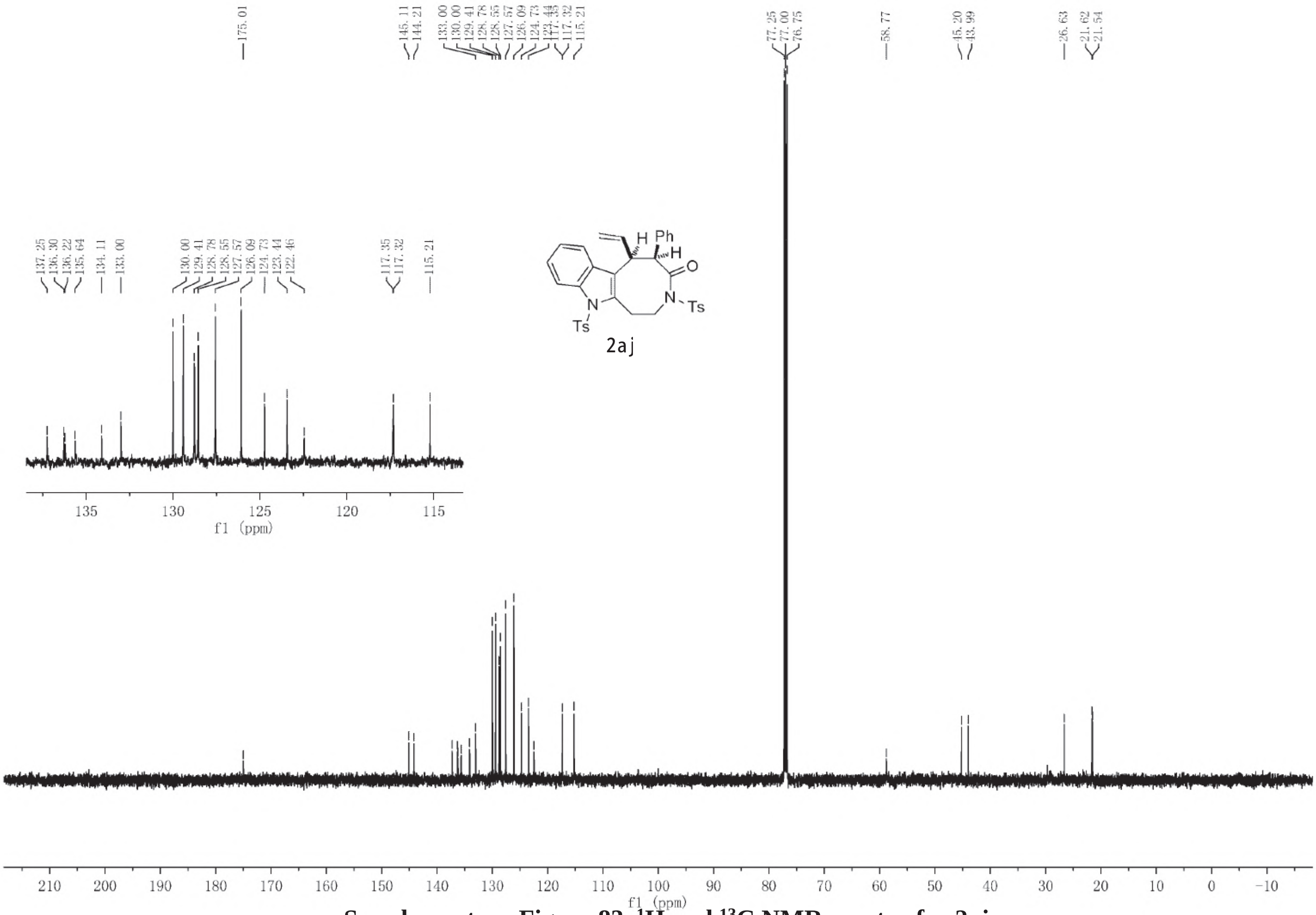

Supplementary Figure 83. ^{1}H and ^{13}C NMR spectra for 2aj

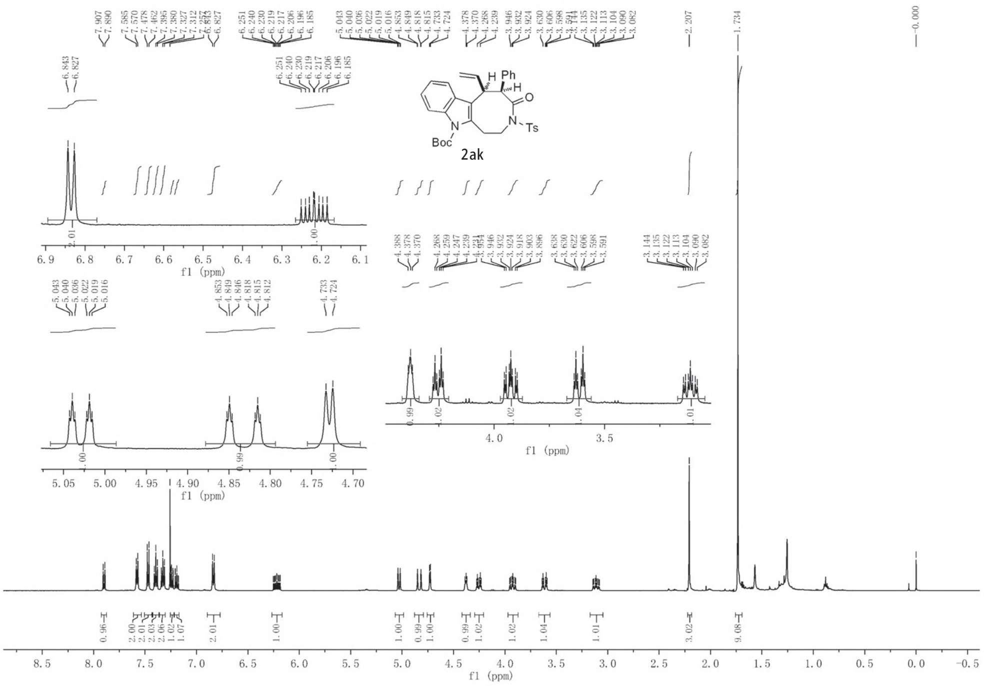

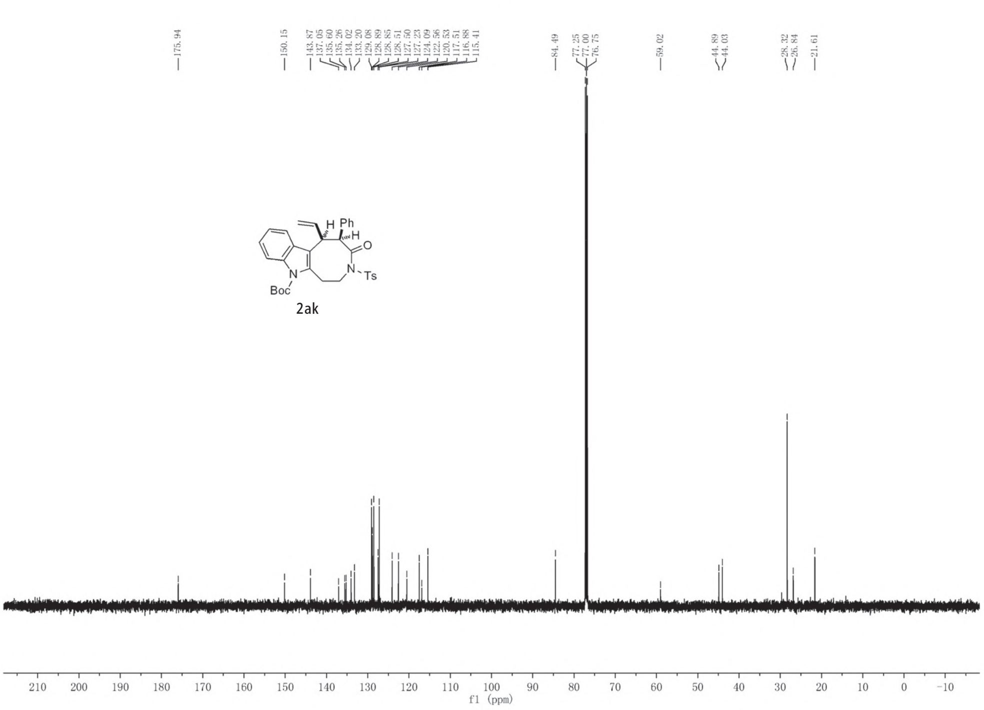

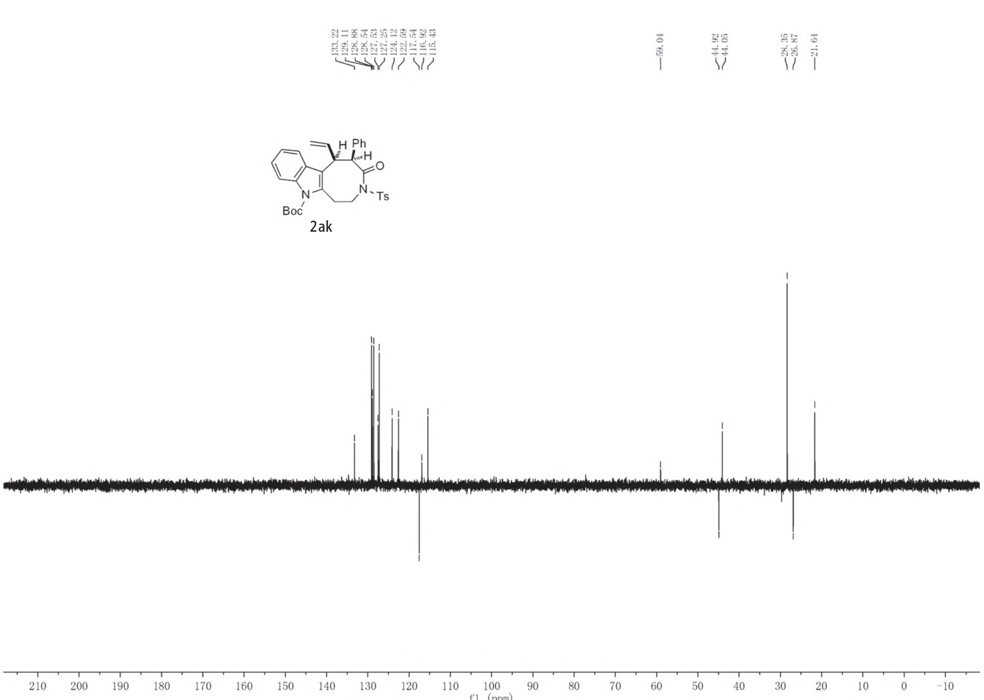

Supplementary Figure 84. ^{1}H and ^{13}C NMR spectra for 2ak

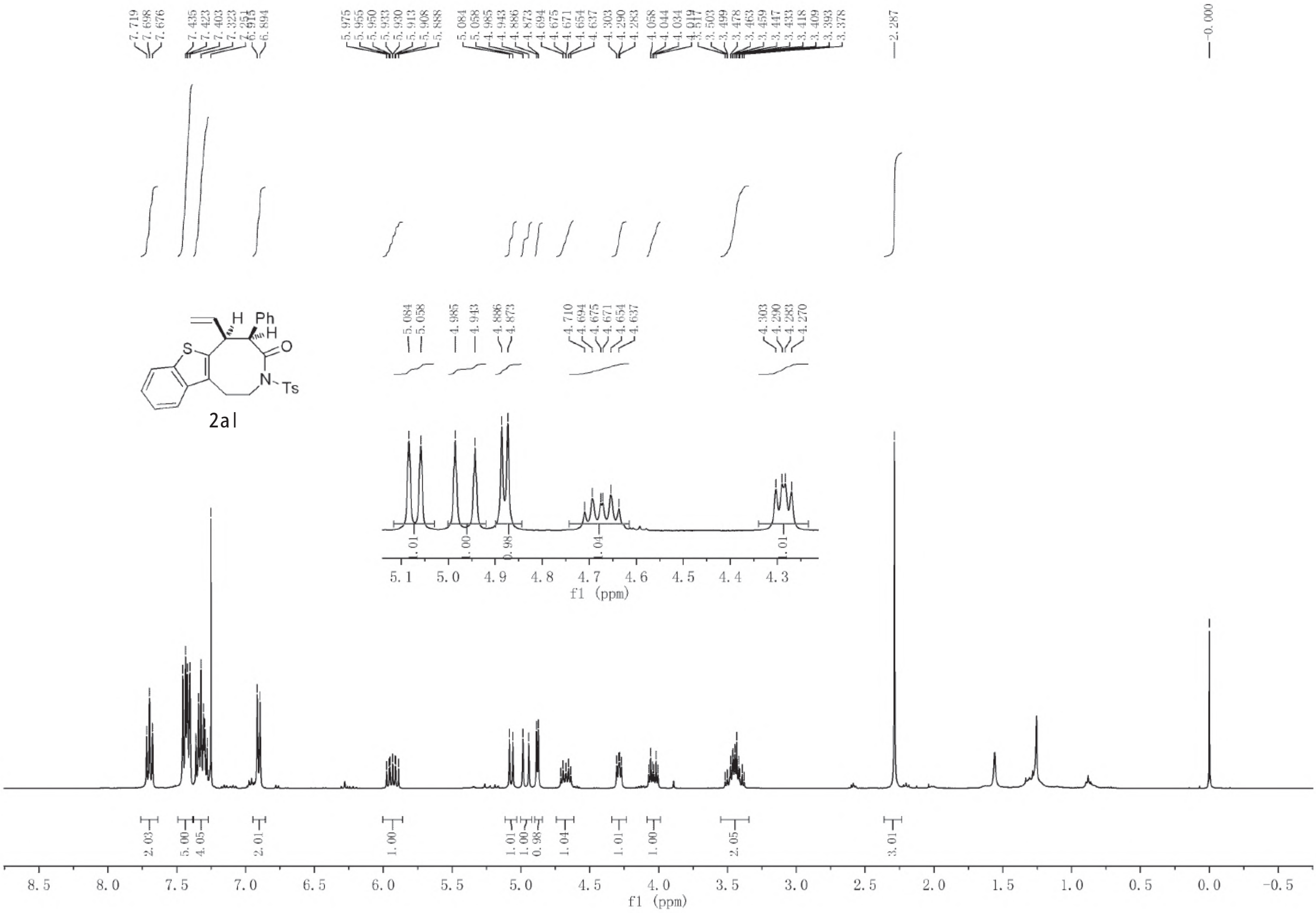

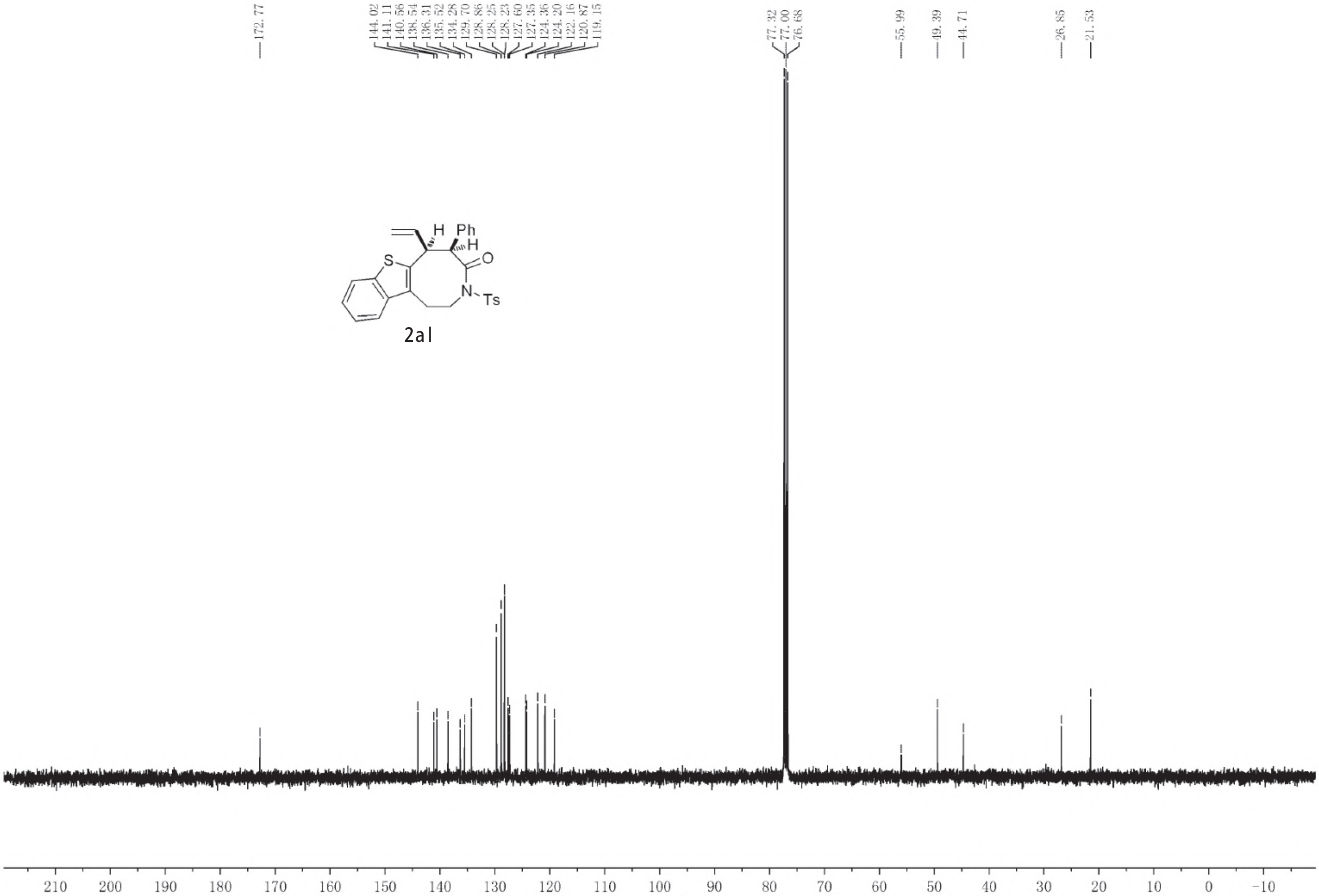

Supplementary Figure 85. ^{1}H and ^{13}C NMR spectra for 2al

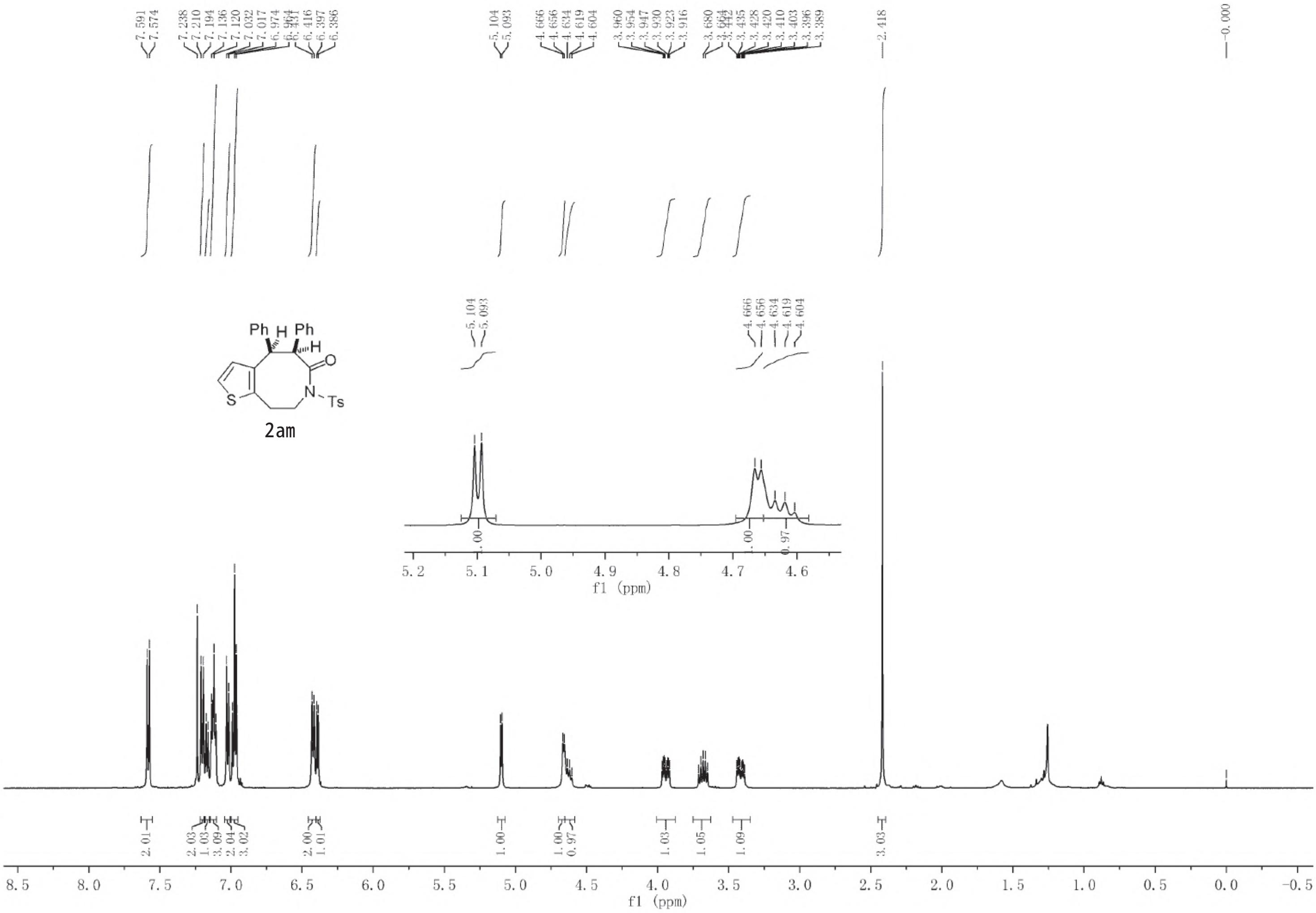

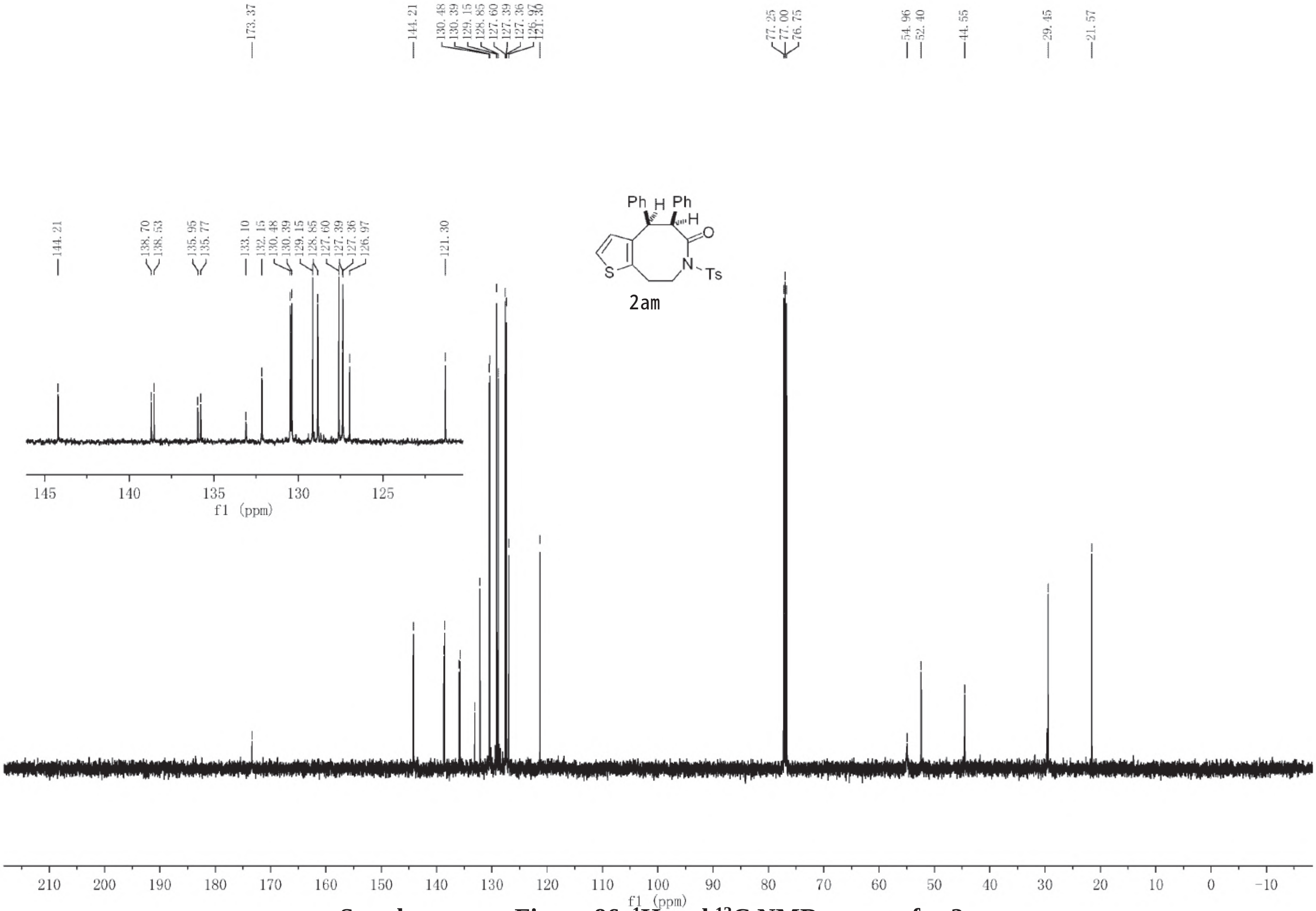

Supplementary Figure 86. ^{1}H and ^{13}C NMR spectra for 2am

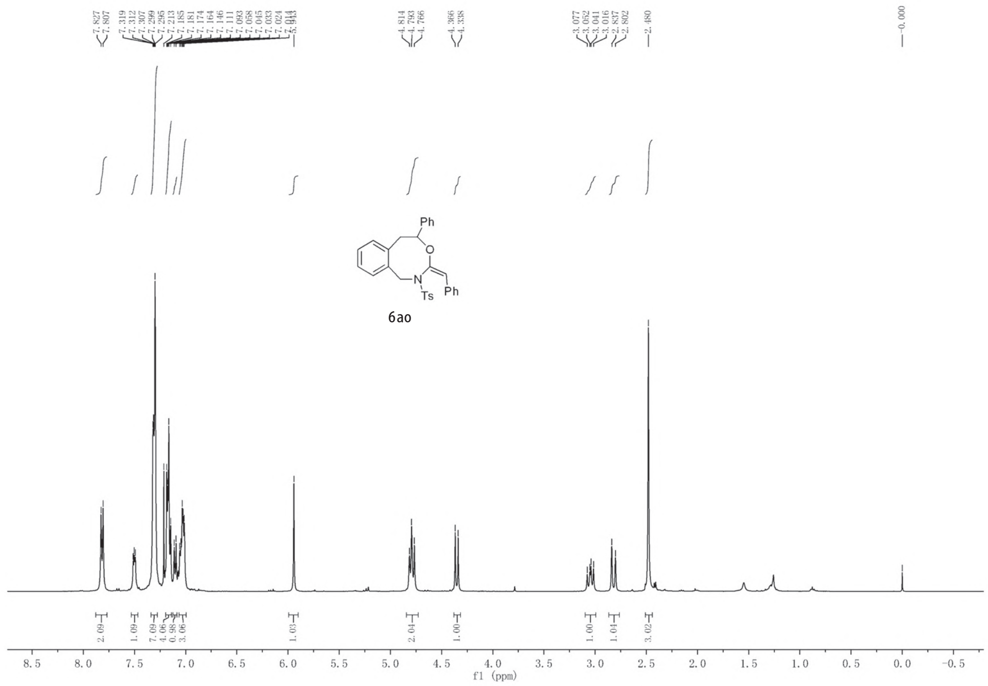

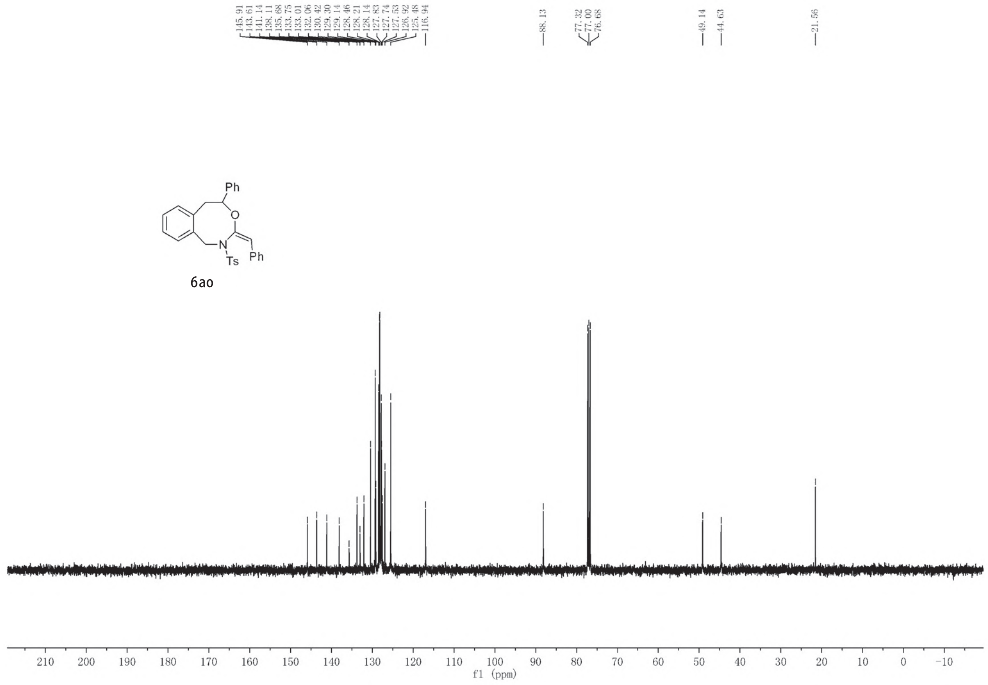

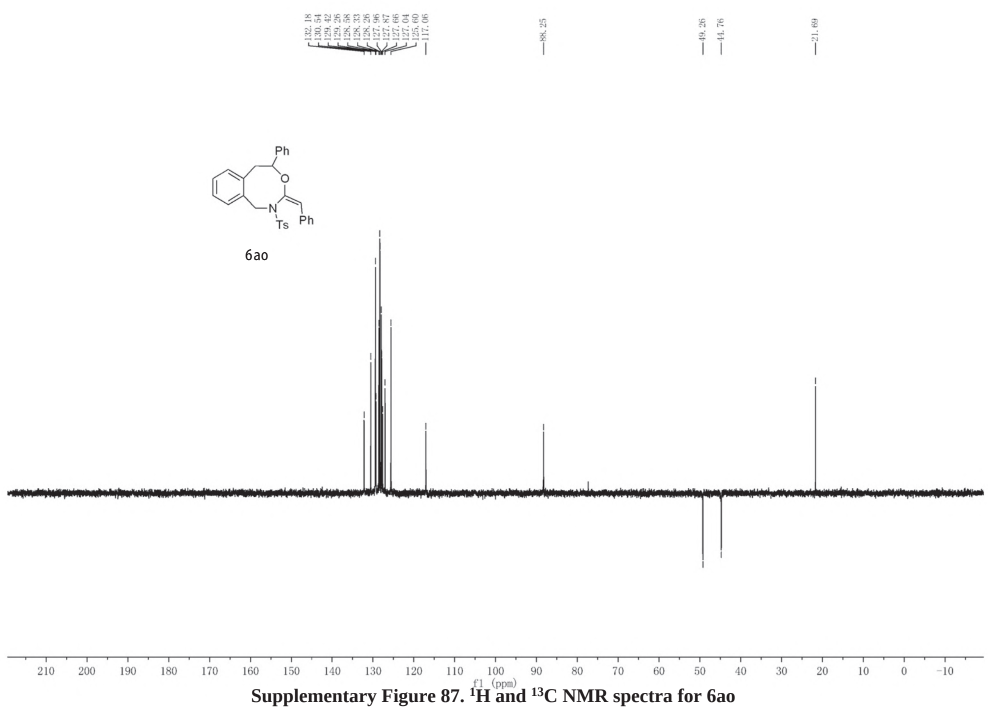

Supplementary Figure 87. ^{1}H and ^{13}C NMR spectra for 6ao

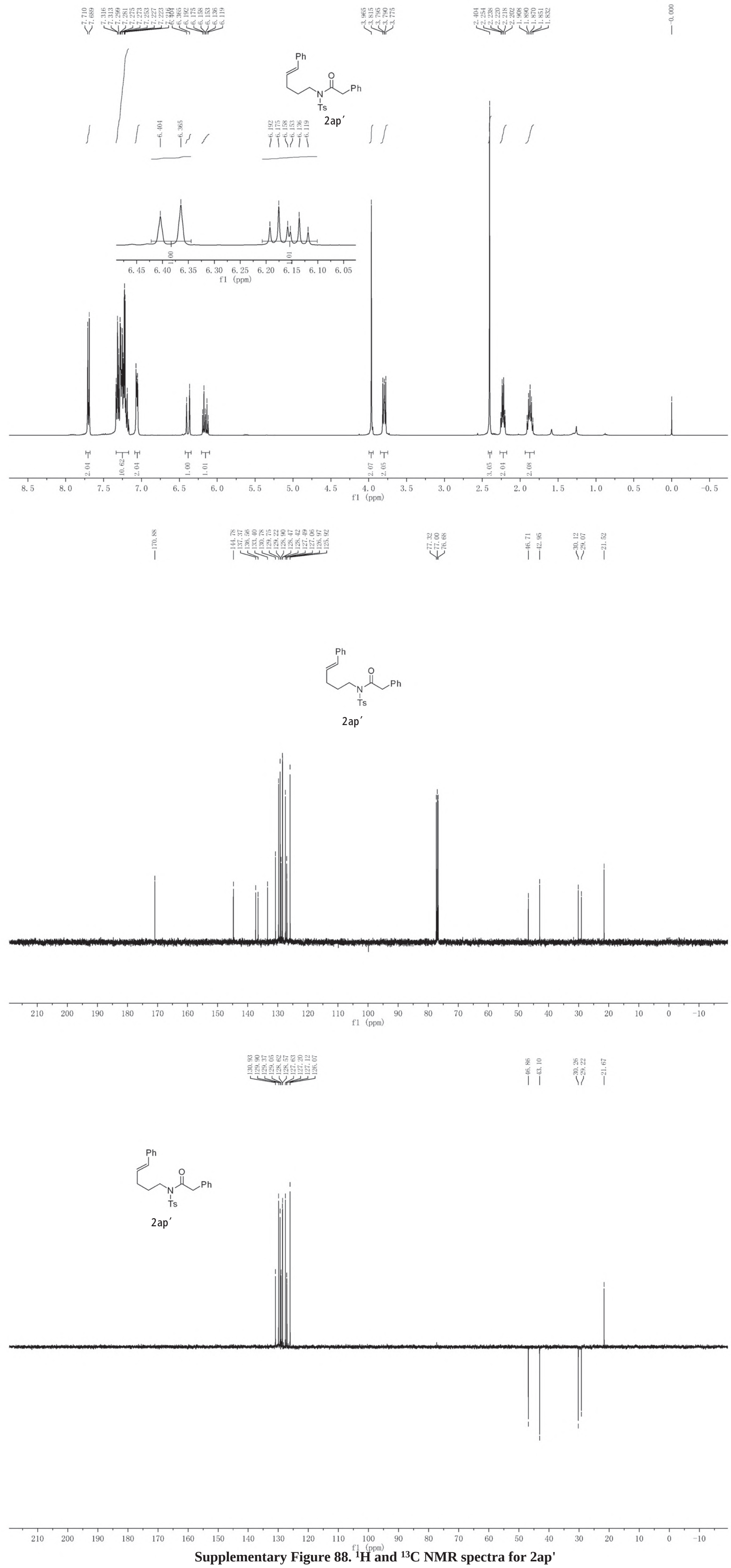

Supplementary Figure 88. ^{1}H and ^{13}C NMR spectra for 2ap'

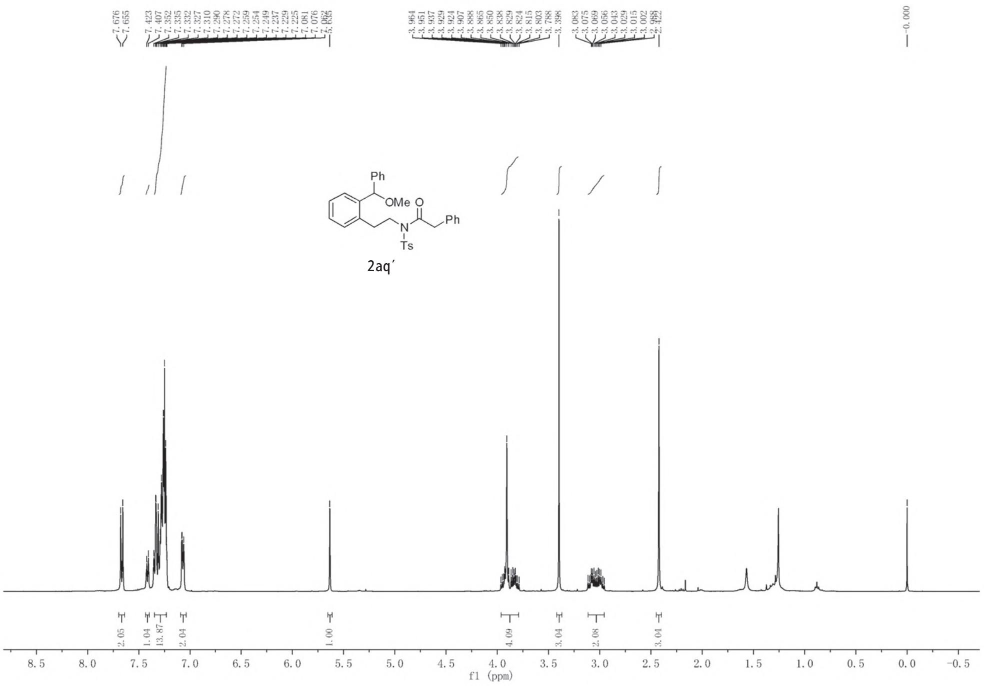

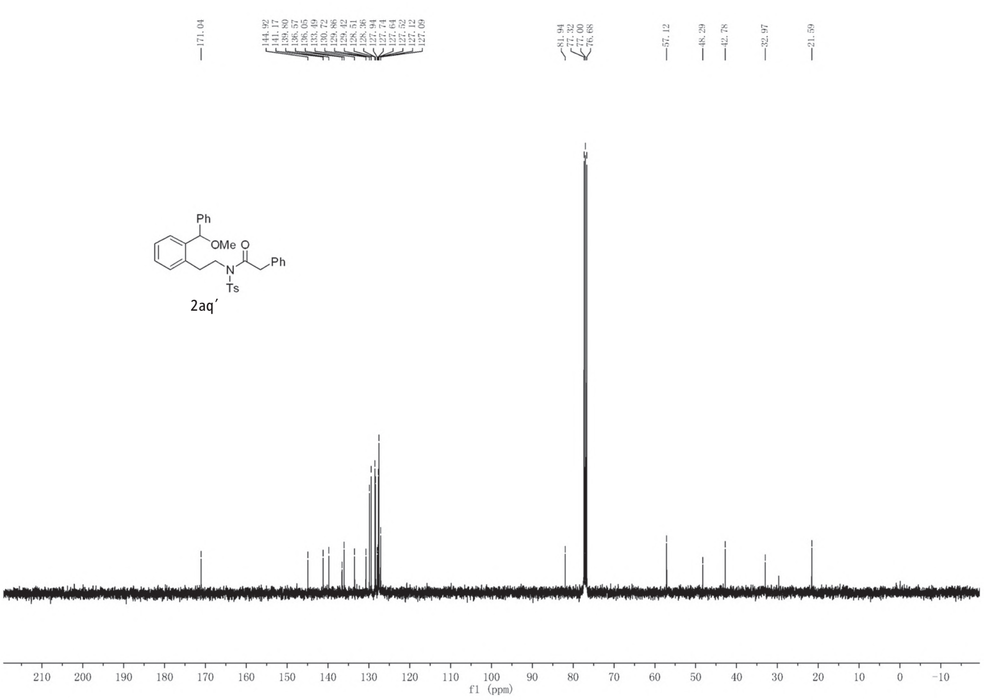

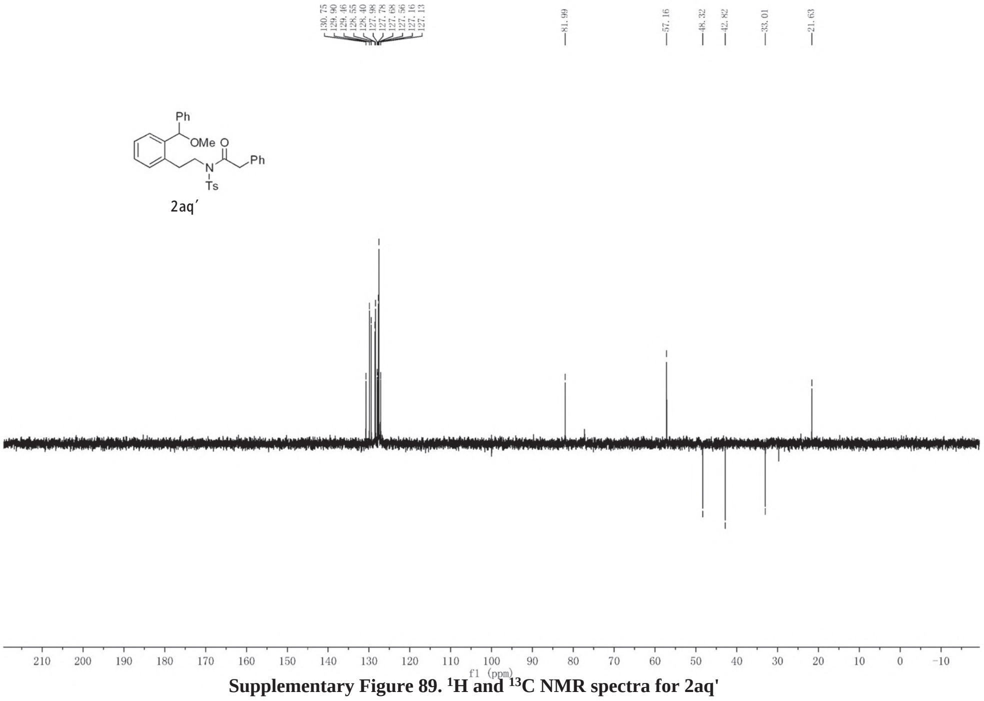

Supplementary Figure 89. ^{1}H and ^{13}C NMR spectra for 2aq'

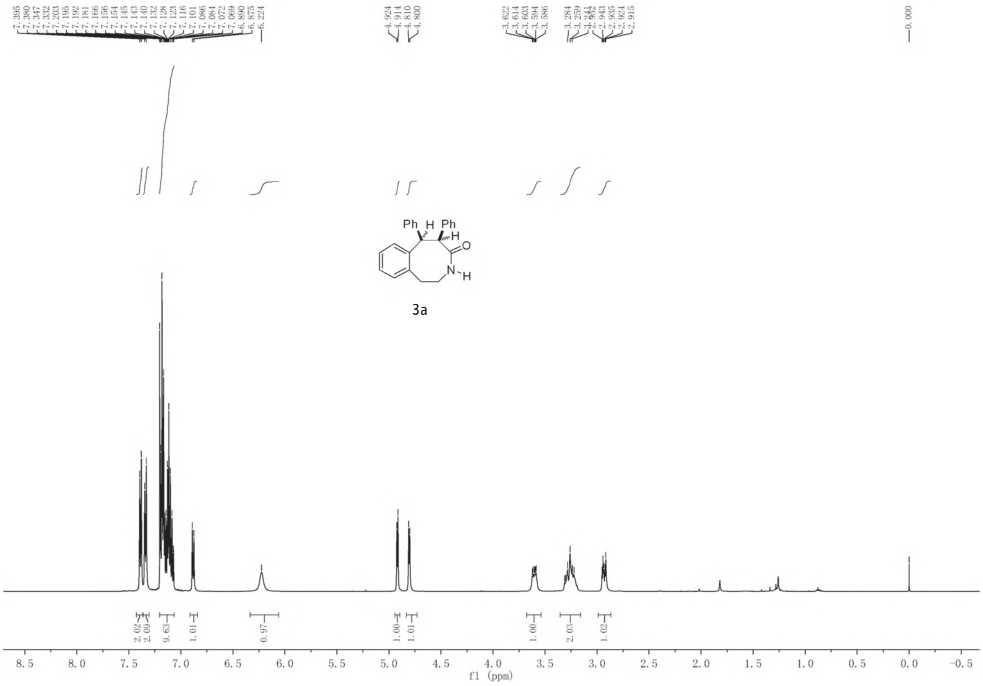

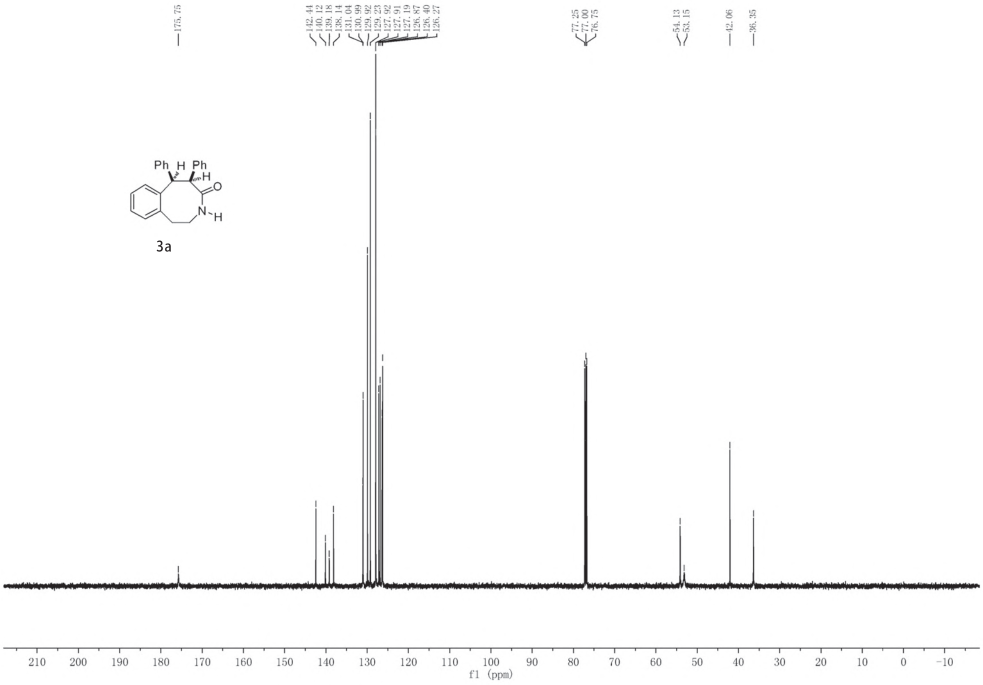

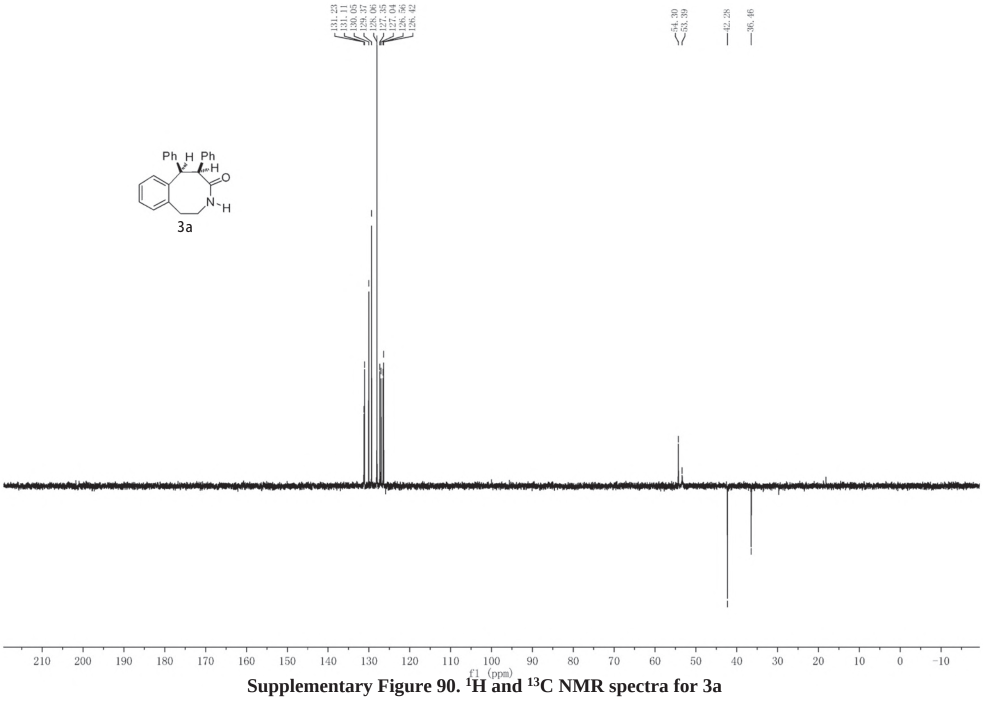

Supplementary Figure 90. ^{1}H and ^{13}C NMR spectra for 3a

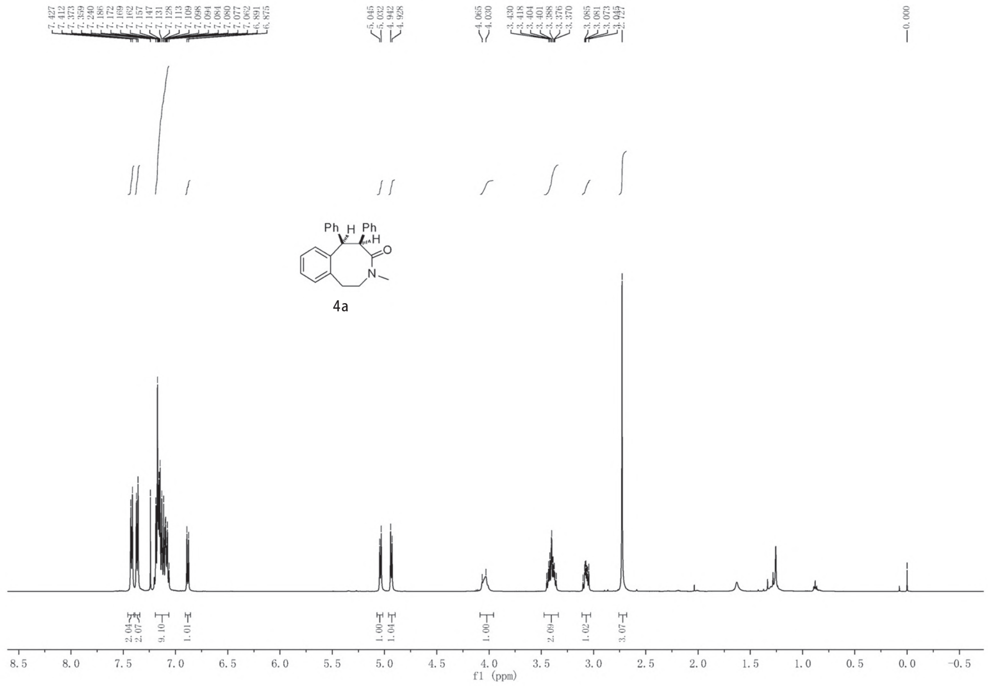

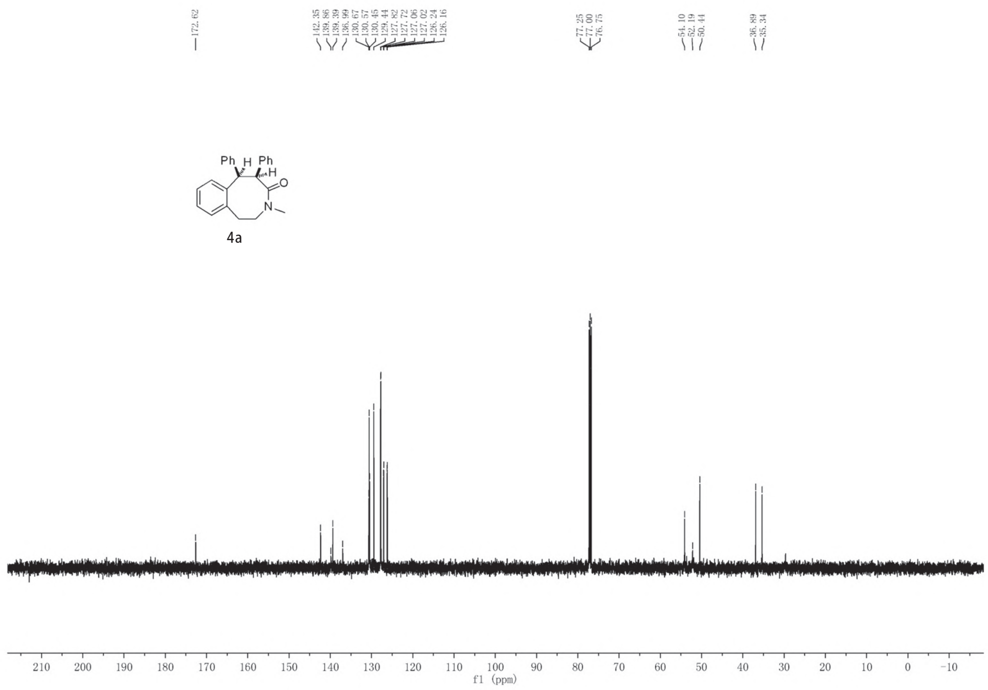

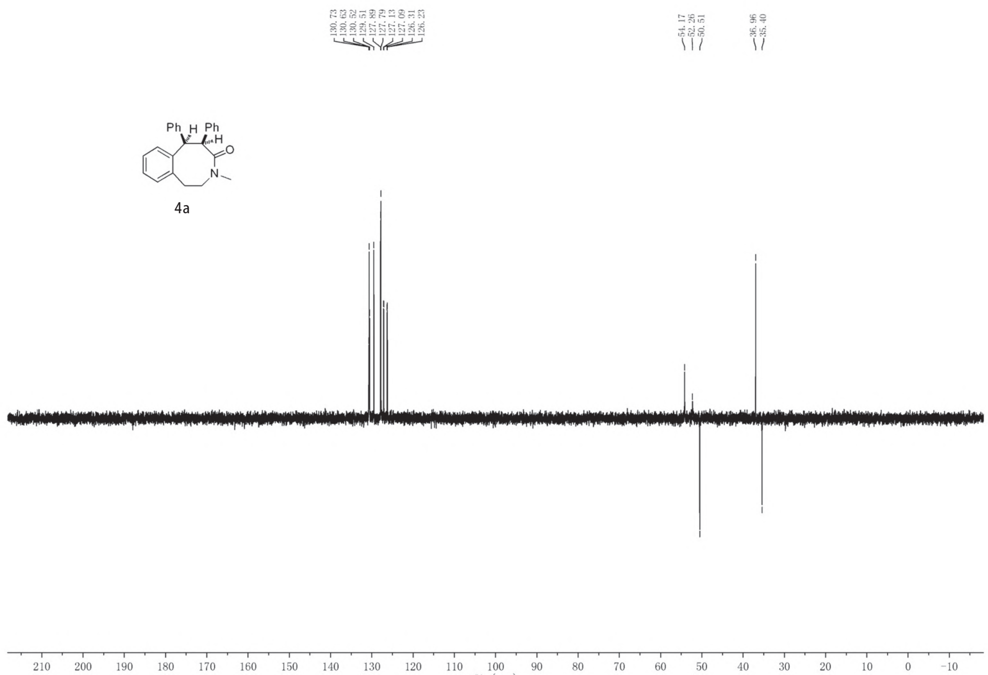

Supplementary Figure 91. ^{1}H and ^{13}C NMR spectra for 4a

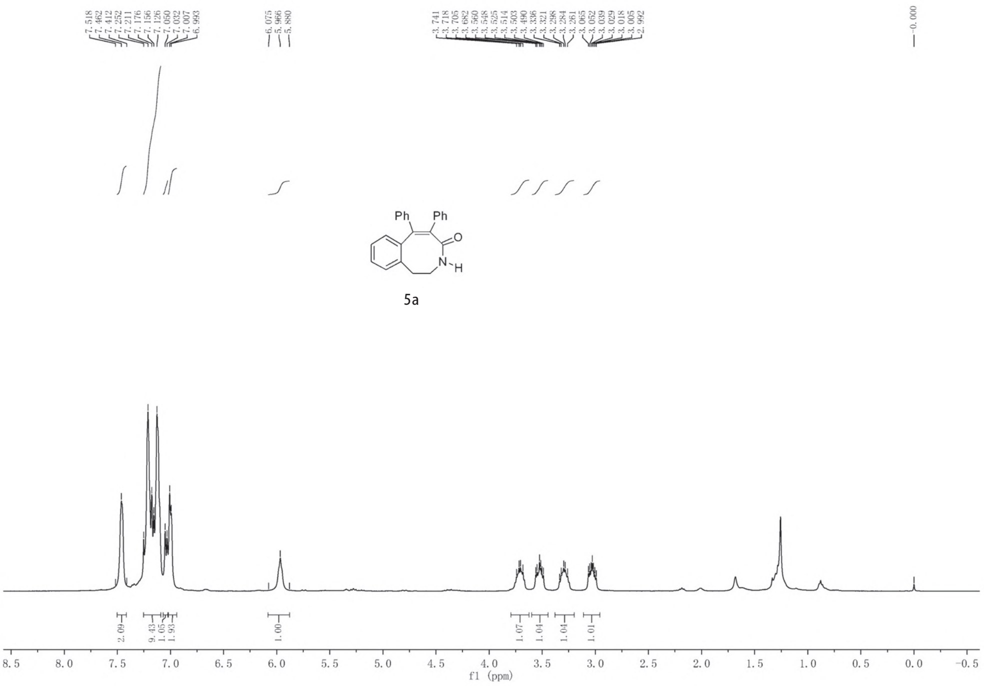

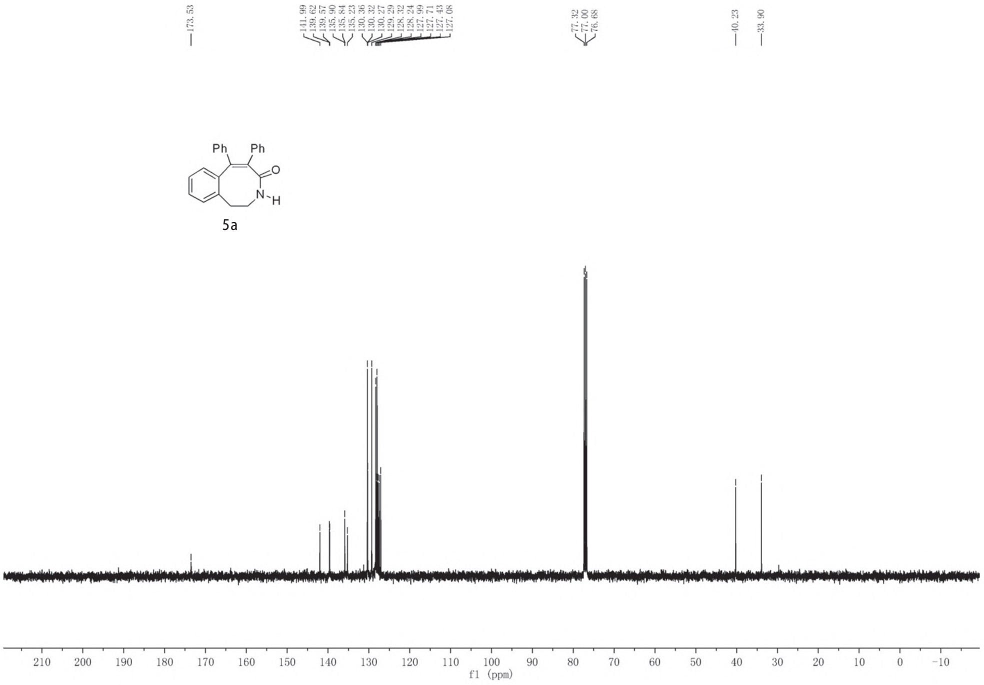

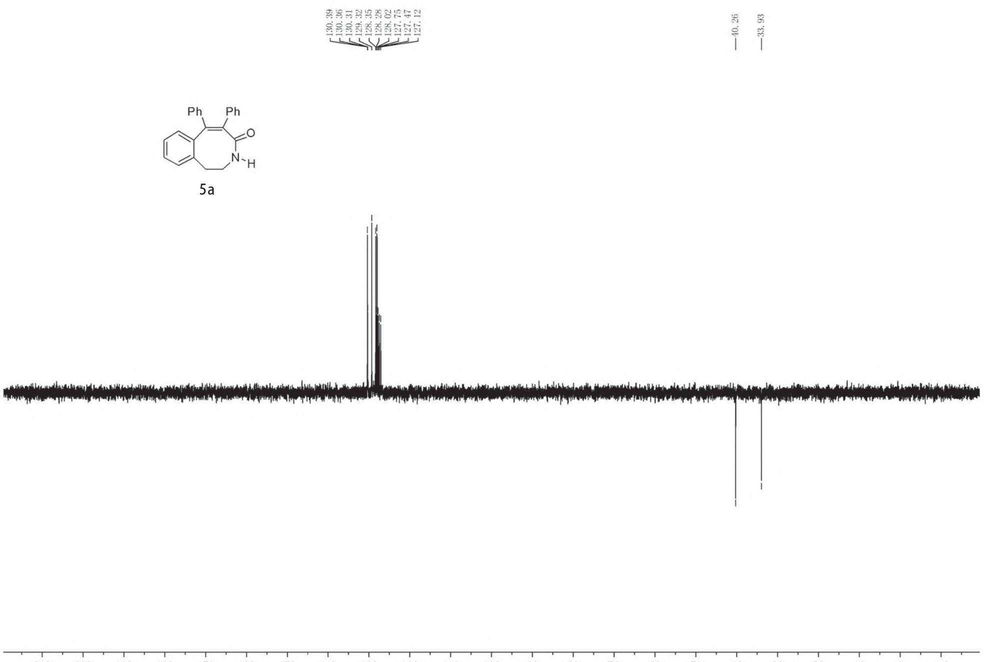

Supplementary Figure 92. ^{1}H and ^{13}C NMR spectra for 5a

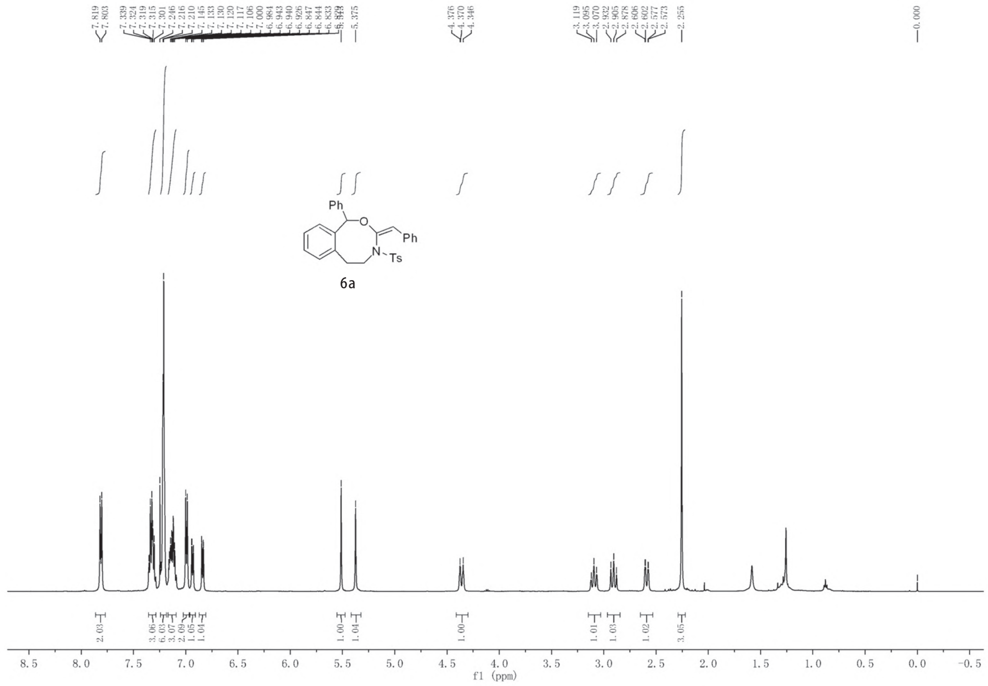

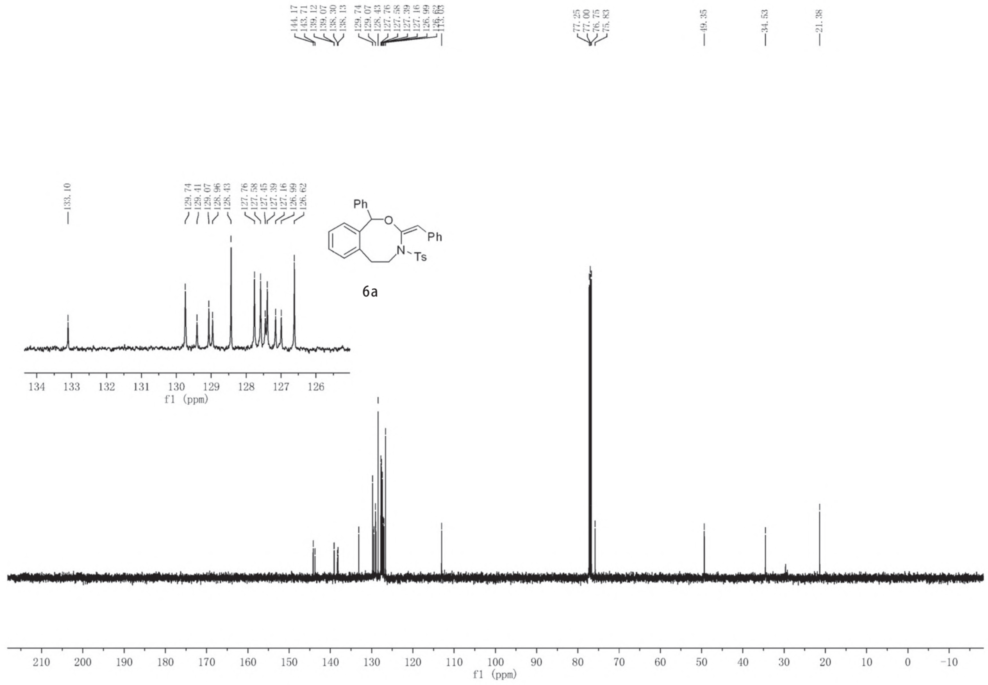

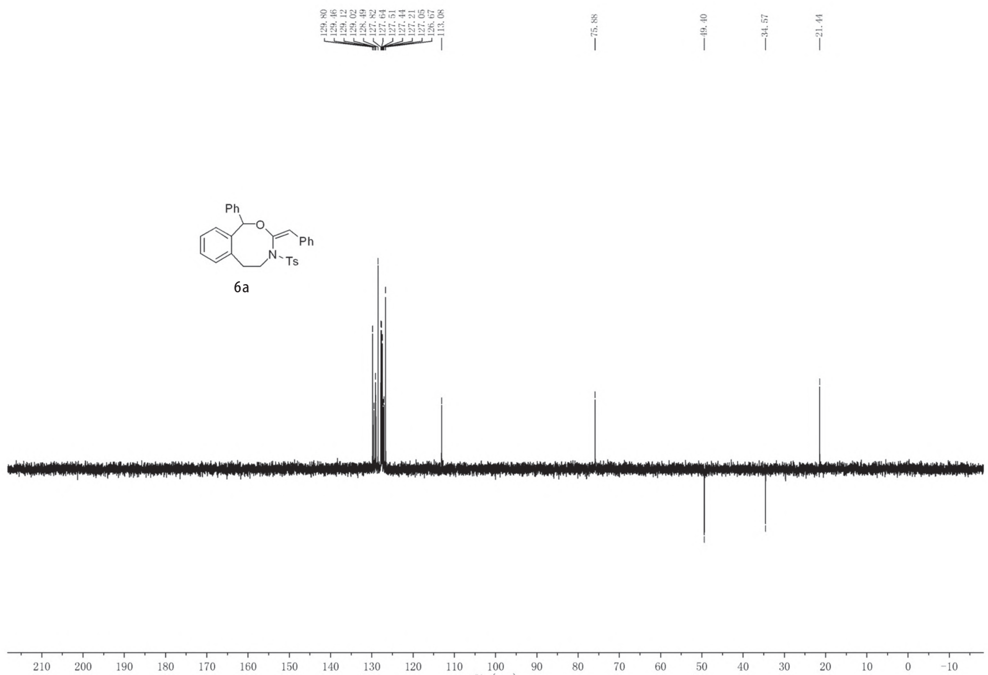

Supplementary Figure 93. ^{1}H and ^{13}C NMR spectra for 6a

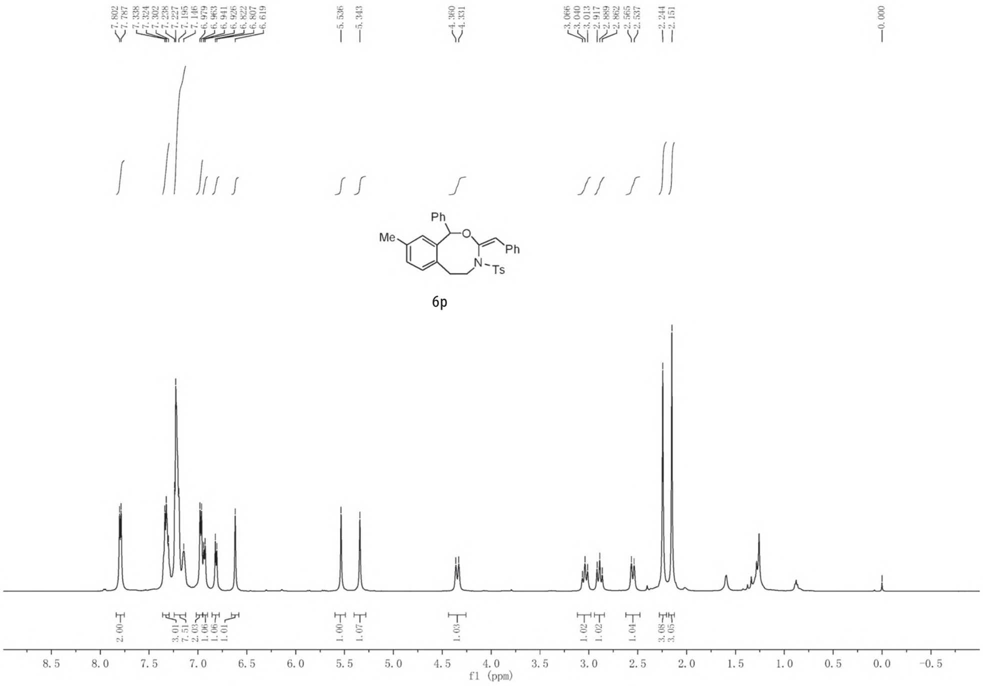

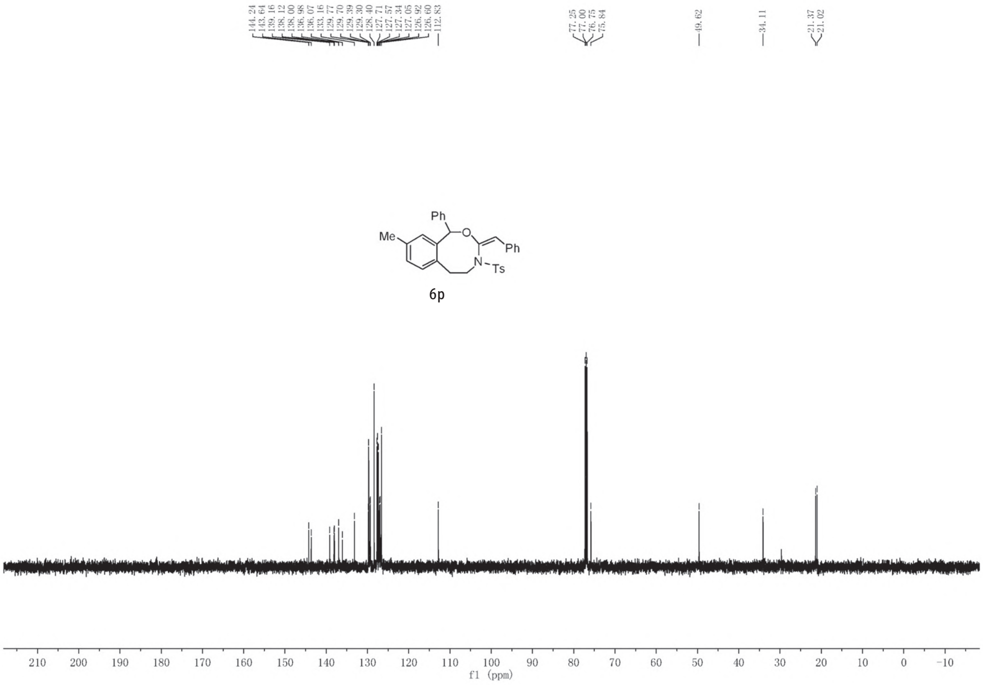

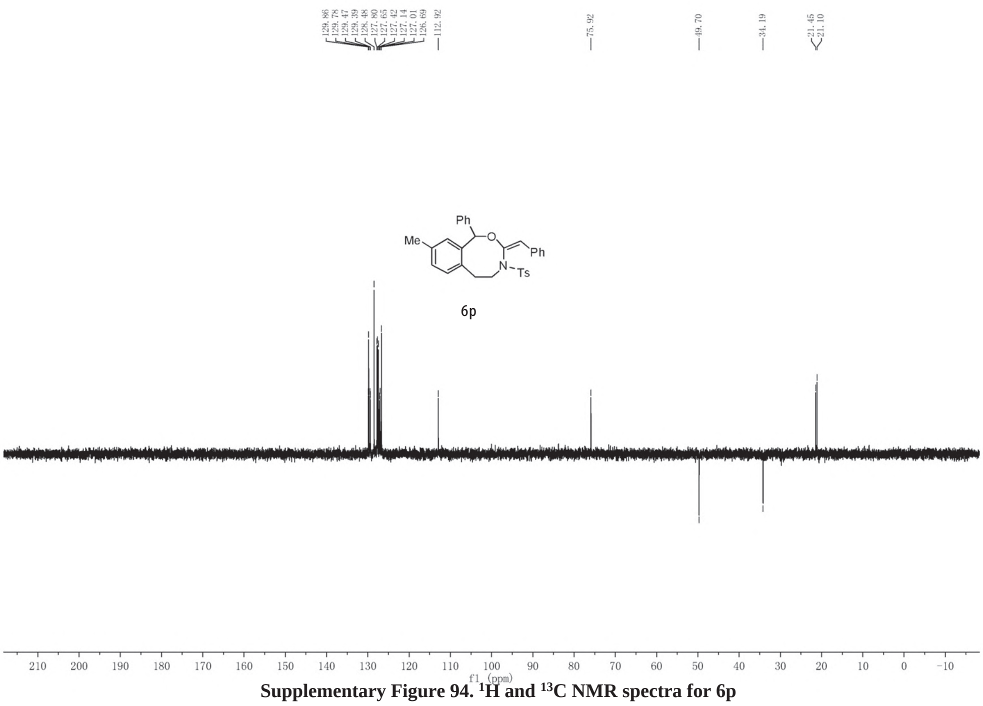

Supplementary Figure 94. ^{1}H and ^{13}C NMR spectra for 6p

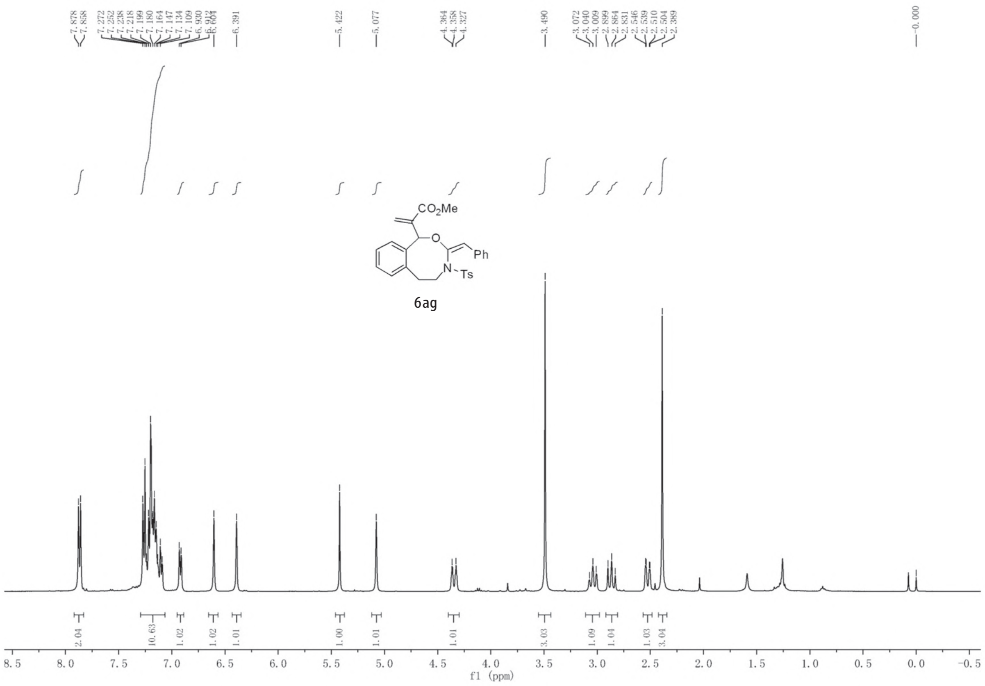

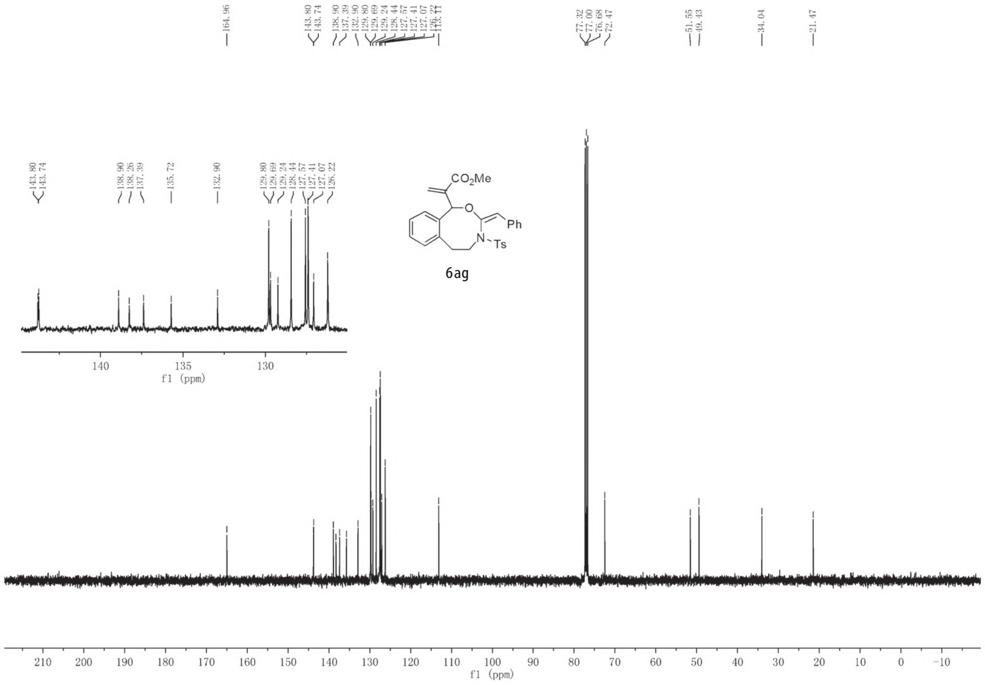

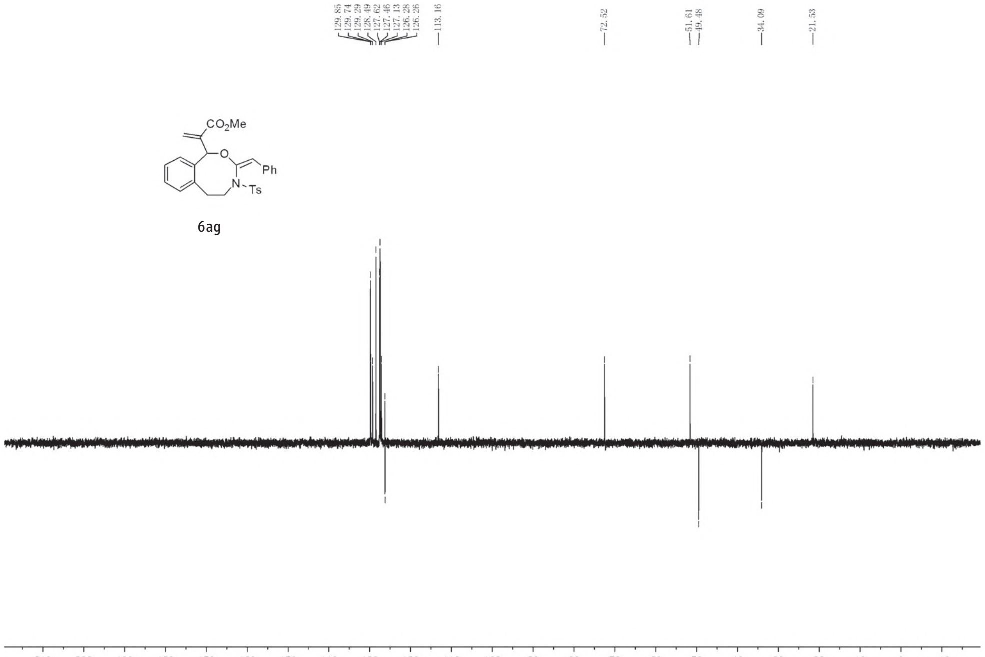

Supplementary Figure 95. ^{1}H and ^{13}C NMR spectra for 6ag

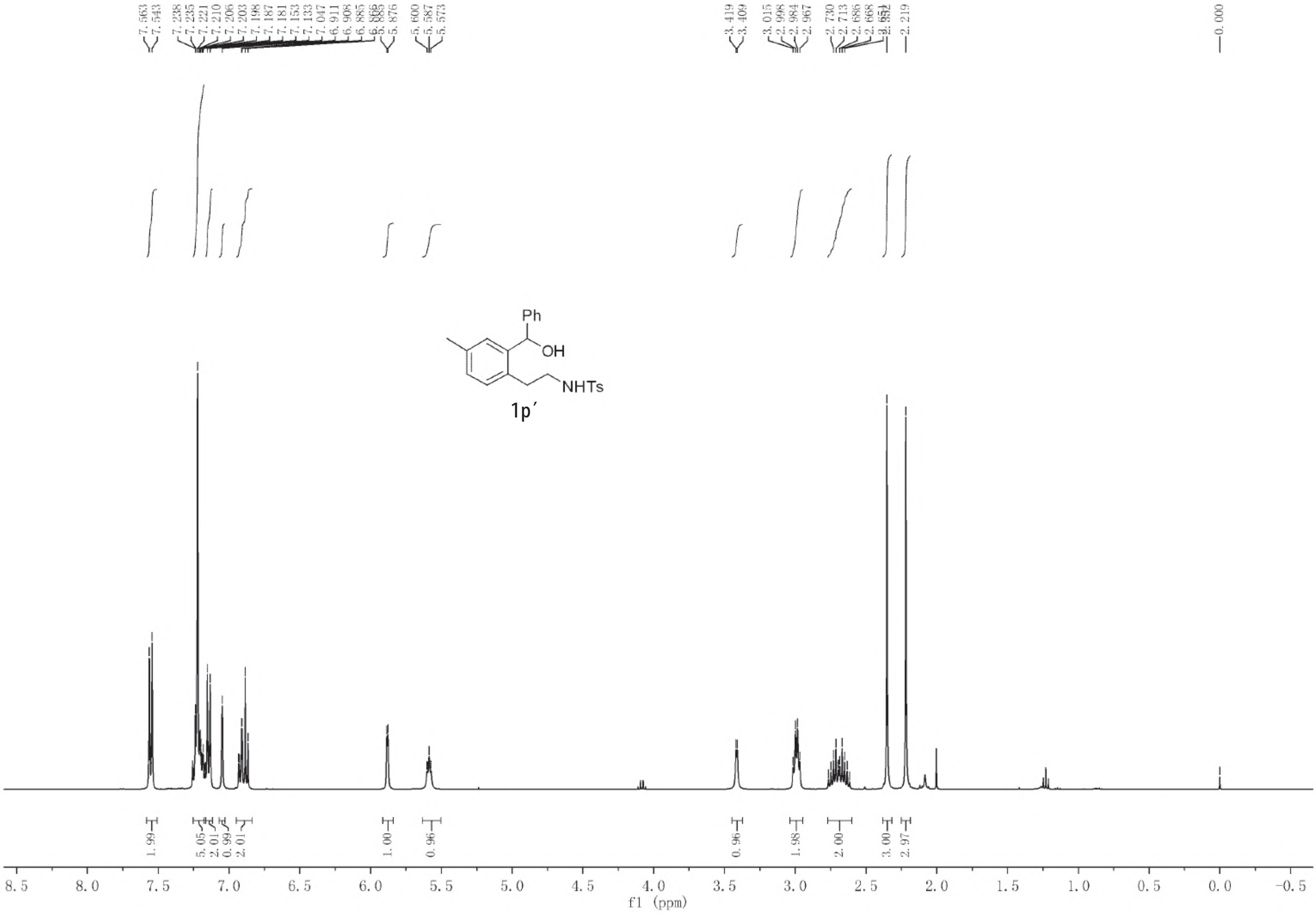

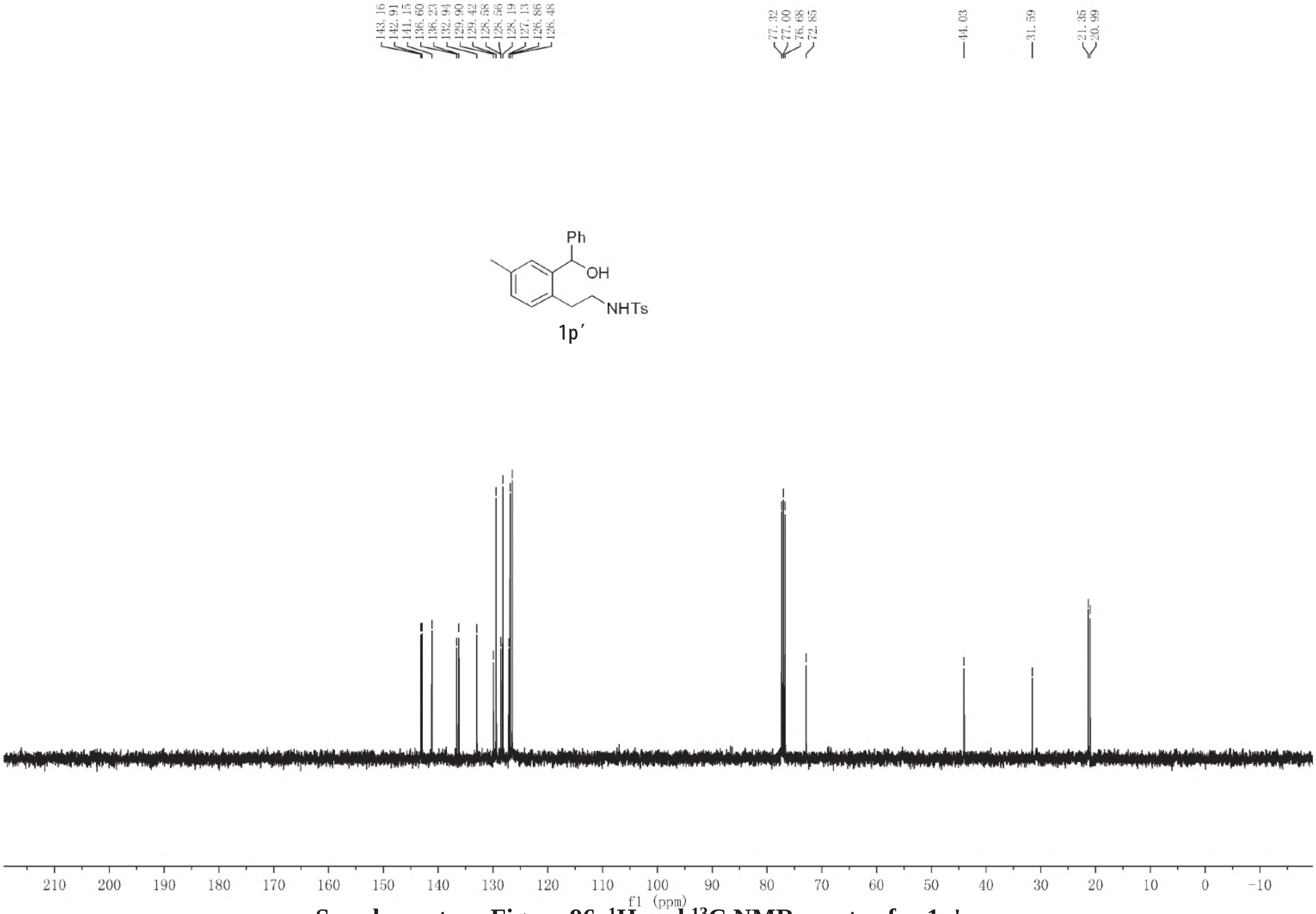

Supplementary Figure 96. ^{1}H and ^{13}C NMR spectra for 1p'

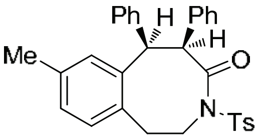

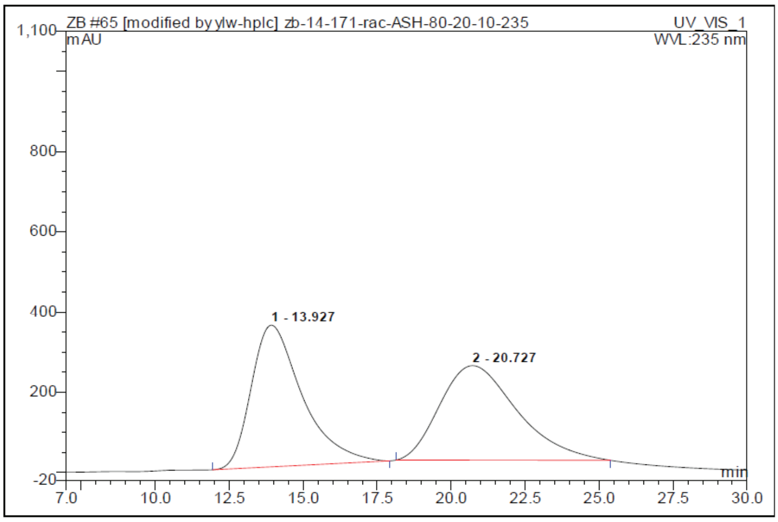

No.	Ret.Time min	Peak Name	Height mAU	Area mAU*min	Rel.Area %	Amount n.a.	Type
1	13.93	n.a.	353.050	704.688	49.70	n.a.	BMB*
2	20.73	n.a.	235.496	713.245	50.30	n.a.	BMB*
Total:			588.546	1417.933	100.00	0.000	

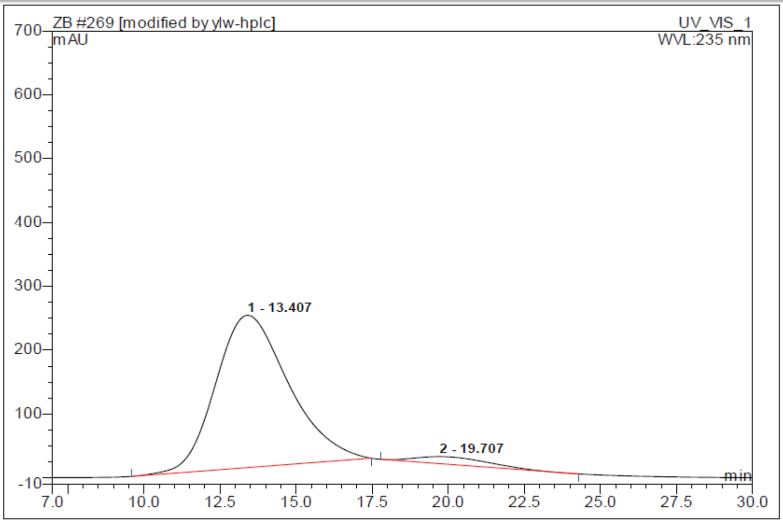

No.	Ret.Time min	Peak Name	Height mAU	Area mAU*min	Rel.Area %	Amount n.a.	Type
1	13.41	n.a.	238.562	664.657	95.39	n.a.	BMB*
2	19.71	n.a.	11.018	32.114	4.61	n.a.	BMB*
Total:			249.580	696.771	100.00	0.000	

Supplementary Figure 97. HPLC spectrum for compound 2p-*ent*

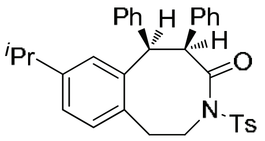

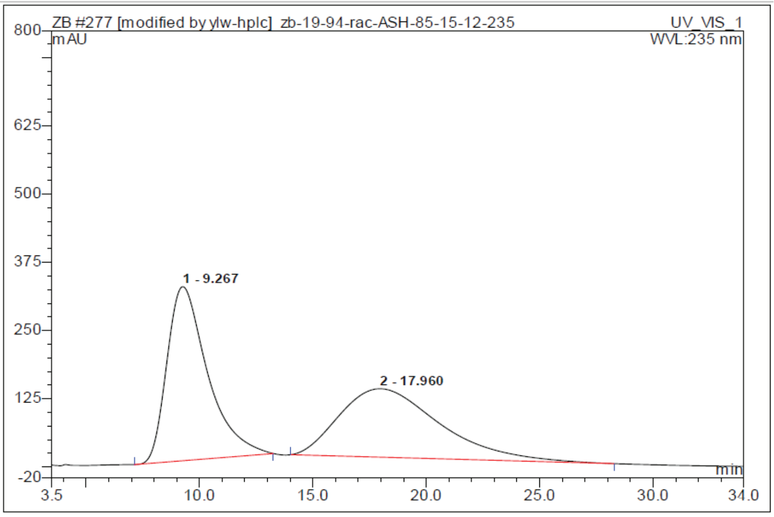

No.	Ret.Time min	Peak Name	Height mAU	Area mAU*min	Rel.Area %	Amount n.a.	Type
1	9.27	n.a.	319.115	663.684	50.83	n.a.	BMB*
2	17.96	n.a.	125.339	641.957	49.17	n.a.	BMB*
Total:			444.455	1305.641	100.00	0.000	

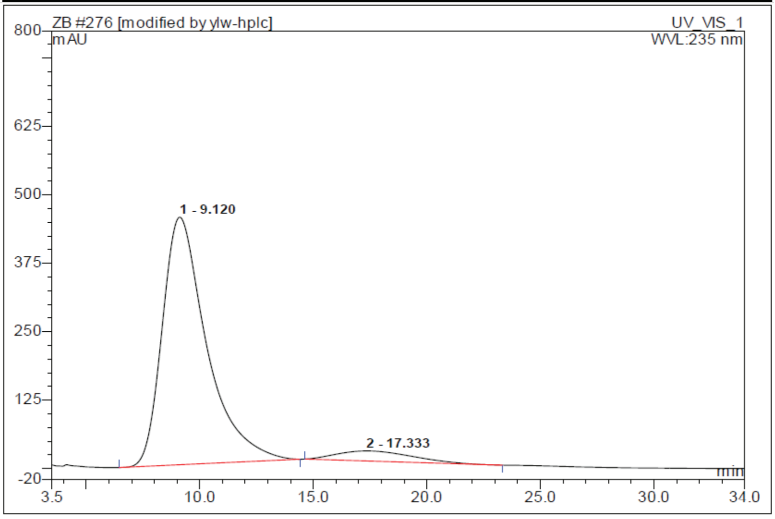

No.	Ret.Time min	Peak Name	Height mAU	Area mAU*min	Rel.Area %	Amount n.a.	Type
1	9.12	n.a.	452.239	1023.860	93.51	n.a.	BMB*
2	17.33	n.a.	18.266	71.011	6.49	n.a.	BMB*
Total:			470.505	1094.871	100.00	0.000	

Supplementary Figure 98. HPLC spectrum for compound 2r-*ent*

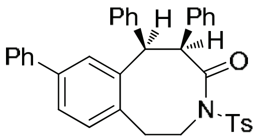

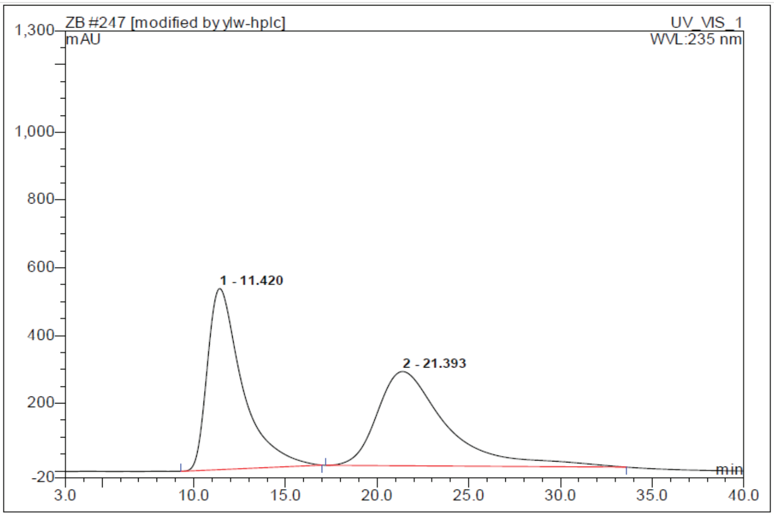

No.	Ret.Time min	Peak Name	Height mAU	Area mAU*min	Rel.Area %	Amount n.a.	Type
1	11.42	n.a.	534.200	1182.780	49.35	n.a.	BMB*
2	21.39	n.a.	278.088	1214.101	50.65	n.a.	BMB*
Total:			812.289	2396.881	100.00	0.000	

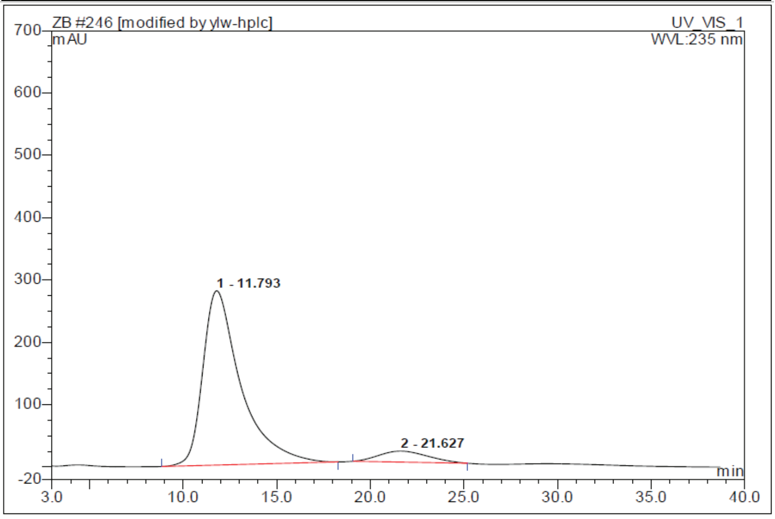

No.	Ret.Time min	Peak Name	Height mAU	Area mAU*min	Rel.Area %	Amount n.a.	Type
1	11.79	n.a.	279.579	657.566	92.50	n.a.	BMB*
2	21.63	n.a.	17.601	53.327	7.50	n.a.	BMB*
Total:			297.180	710.893	100.00	0.000	

Supplementary Figure 99. HPLC spectrum for compound 2t-*ent*

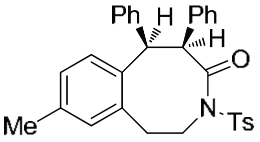

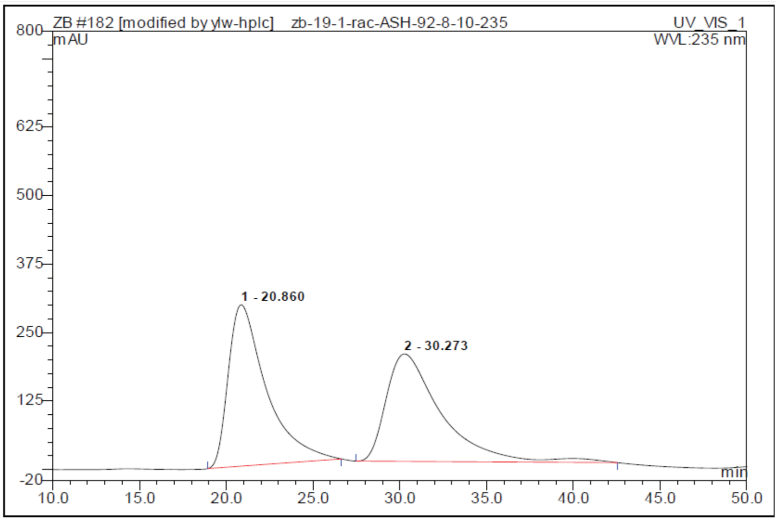

No.	Ret.Time min	Peak Name	Height mAU	Area mAU*min	Rel.Area %	Amount n.a.	Type
1	20.86	n.a.	294.475	749.115	50.89	n.a.	BMB*
2	30.27	n.a.	196.082	722.913	49.11	n.a.	BMB*
Total:			490.557	1472.028	100.00	0.000	

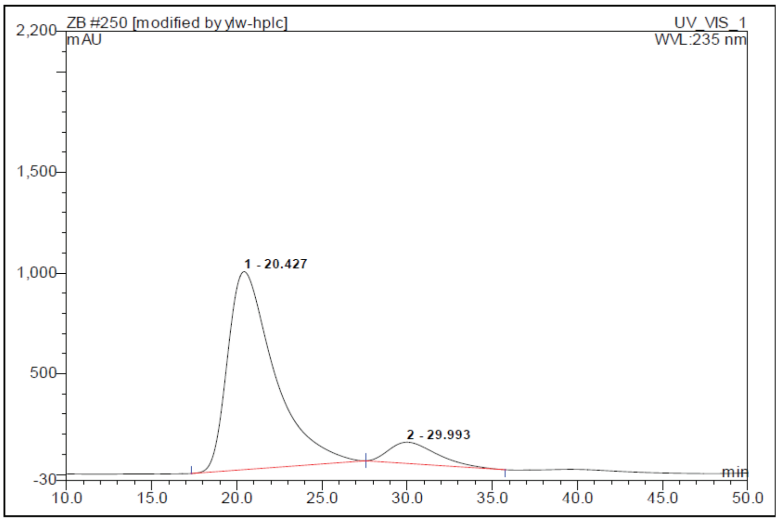

No.	Ret.Time min	Peak Name	Height mAU	Area mAU*min	Rel.Area %	Amount n.a.	Type
1	20.43	n.a.	982.941	3138.106	90.10	n.a.	BMb*
2	29.99	n.a.	105.486	344.625	9.90	n.a.	bMB*
Total:			1088.428	3482.731	100.00	0.000	

Supplementary Figure 100. HPLC spectrum for compound 2w-*ent*

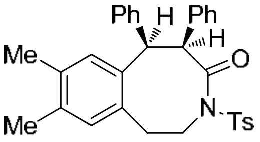

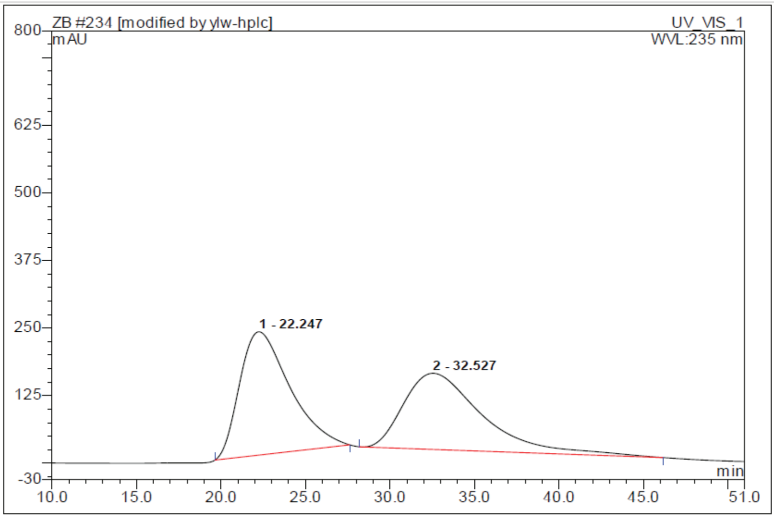

No.	Ret.Time min	Peak Name	Height mAU	Area mAU*min	Rel.Area %	Amount n.a.	Type
1	22.25	n.a.	228.036	787.109	50.96	n.a.	BMB*
2	32.53	n.a.	140.791	757.498	49.04	n.a.	BMB*
Total:			368.827	1544.607	100.00	0.000	

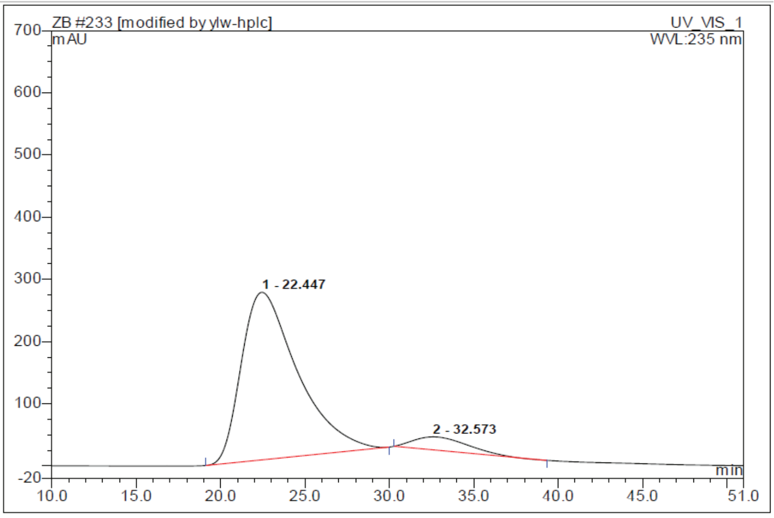

No.	Ret.Time min	Peak Name	Height mAU	Area mAU*min	Rel.Area %	Amount n.a.	Type
1	22.45	n.a.	269.337	1041.655	93.01	n.a.	BMB*
2	32.57	n.a.	20.839	78.234	6.99	n.a.	BMB*
Total:			290.176	1119.889	100.00	0.000	

Supplementary Figure 101. HPLC spectrum for compound 2z-*ent*

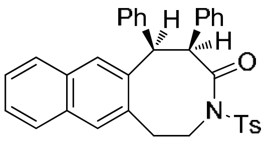

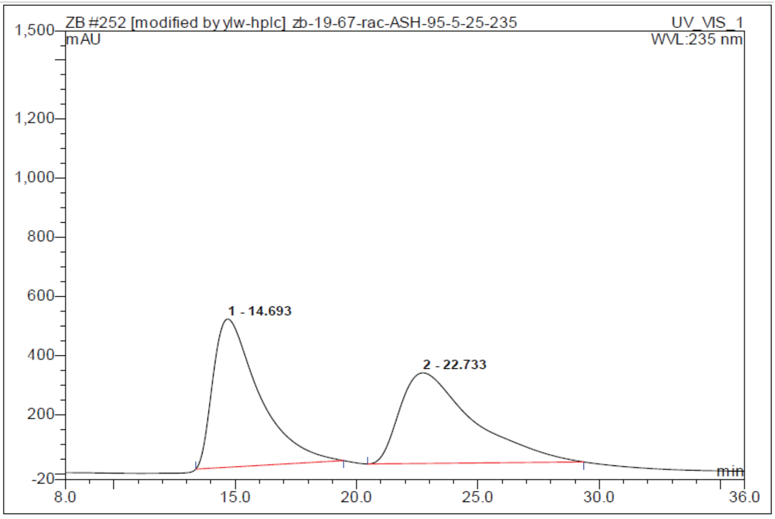

No.	Ret.Time min	Peak Name	Height mAU	Area mAU*min	Rel.Area %	Amount n.a.	Type
1	14.69	n.a.	501.866	1107.688	50.95	n.a.	BMB*
2	22.73	n.a.	306.584	1066.463	49.05	n.a.	BMB*
Total:			808.451	2174.151	100.00	0.000	

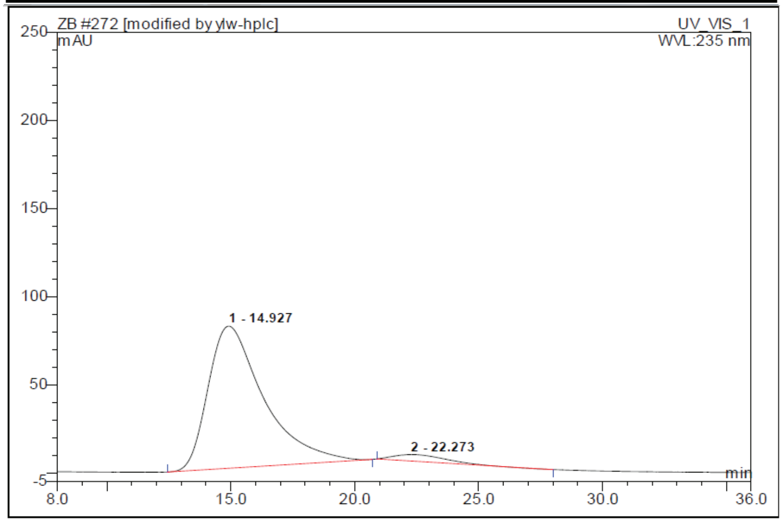

No.	Ret.Time min	Peak Name	Height mAU	Area mAU*min	Rel.Area %	Amount n.a.	Type
1	14.93	n.a.	80.709	210.113	95.99	n.a.	BMB*
2	22.27	n.a.	3.705	8.782	4.01	n.a.	BMB*
Total:			84.415	218.895	100.00	0.000	

Supplementary Figure 102. HPLC spectrum for compound 2ab-*ent*

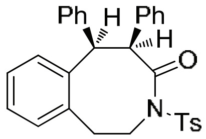

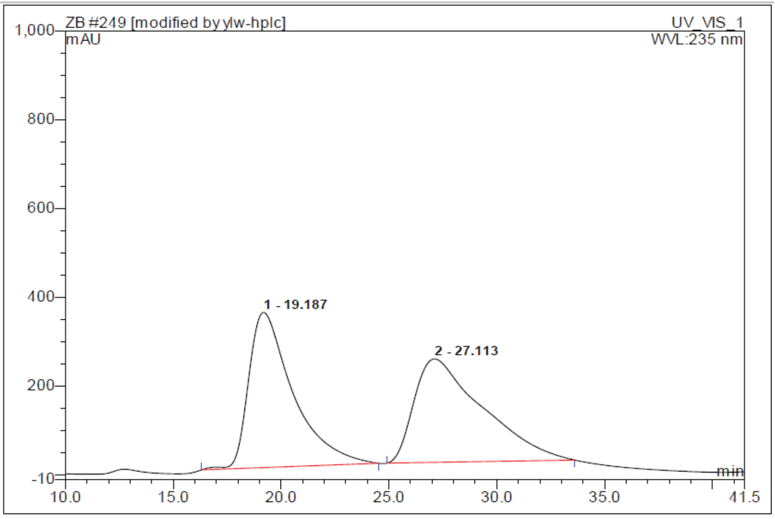

No.	Ret.Time min	Peak Name	Height mAU	Area mAU*min	Rel.Area %	Amount n.a.	Type
1	19.19	n.a.	348.898	831.918	49.36	n.a.	BMB*
2	27.11	n.a.	232.567	853.459	50.64	n.a.	BMB*
Total:			581.465	1685.376	100.00	0.000	

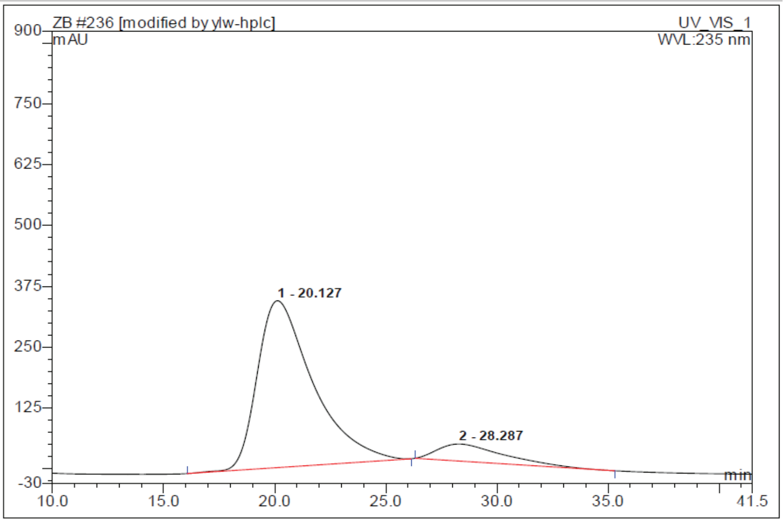

No.	Ret.Time min	Peak Name	Height mAU	Area mAU*min	Rel.Area %	Amount n.a.	Type
1	20.13	n.a.	342.851	997.626	89.07	n.a.	BMB*
2	28.29	n.a.	34.906	122.469	10.93	n.a.	BMB*
Total:			377.757	1120.095	100.00	0.000	

Supplementary Figure 103. HPLC spectrum for compound 2a-*ent*

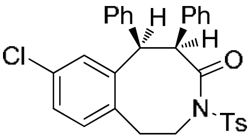

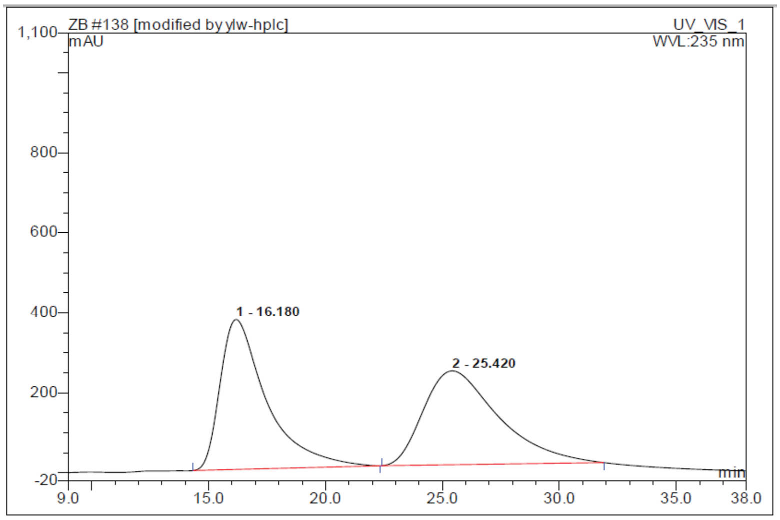

No.	Ret.Time min	Peak Name	Height mAU	Area mAU*min	Rel.Area %	Amount n.a.	Type
1	16.18	n.a.	374.636	863.325	49.76	n.a.	BMB*
2	25.42	n.a.	234.462	871.671	50.24	n.a.	BMB*
Total:			609.098	1734.995	100.00	0.000	

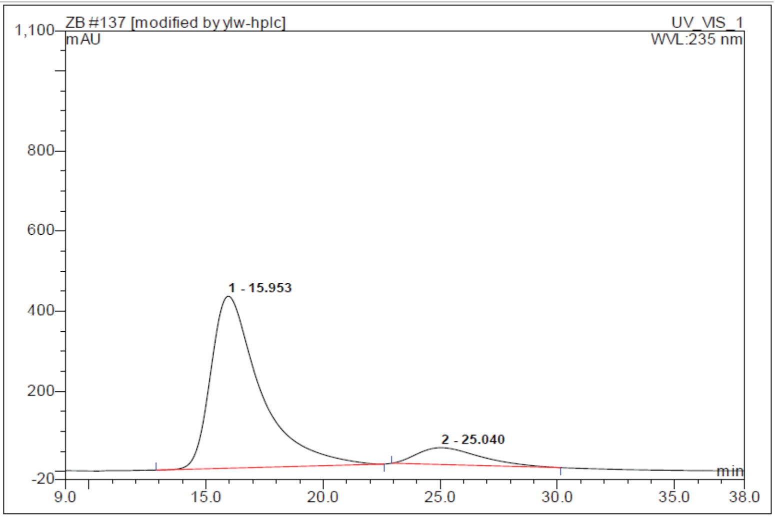

No.	Ret.Time min	Peak Name	Height mAU	Area mAU*min	Rel.Area %	Amount n.a.	Type
1	15.95	n.a.	429.203	1052.856	88.91	n.a.	BMB*
2	25.04	n.a.	41.986	131.373	11.09	n.a.	BMB*
Total:			471.188	1184.229	100.00	0.000	

Supplementary Figure 104. HPLC spectrum for compound 2u-*ent*

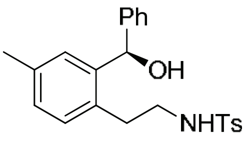

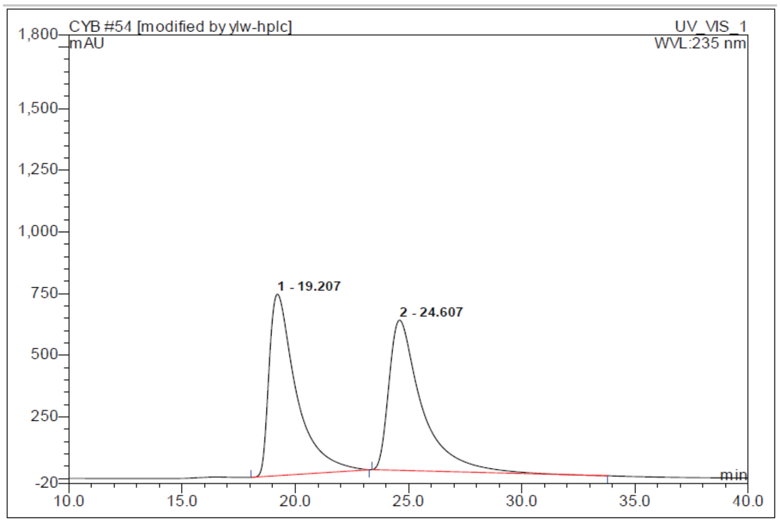

No.	Ret.Time min	Peak Name	Height mAU	Area mAU*min	Rel.Area %	Amount n.a.	Type
1	19.21	n.a.	735.733	1025.693	50.00	n.a.	BMB*
2	24.61	n.a.	608.879	1025.516	50.00	n.a.	BMB*
Total:			1344.613	2051.209	100.00	0.000	

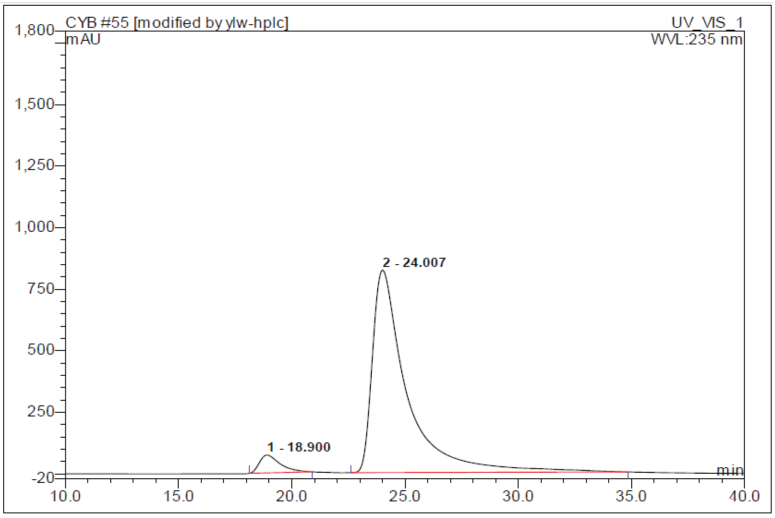

No.	Ret.Time min	Peak Name	Height mAU	Area mAU*min	Rel.Area %	Amount n.a.	Type
1	18.90	n.a.	72.345	76.914	5.00	n.a.	BMB*
2	24.01	n.a.	821.705	1461.503	95.00	n.a.	BMB*
Total:			894.050	1538.416	100.00	0.000	

Supplementary Figure 105. HPLC spectrum for compound (*R*)-1p'

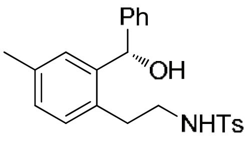

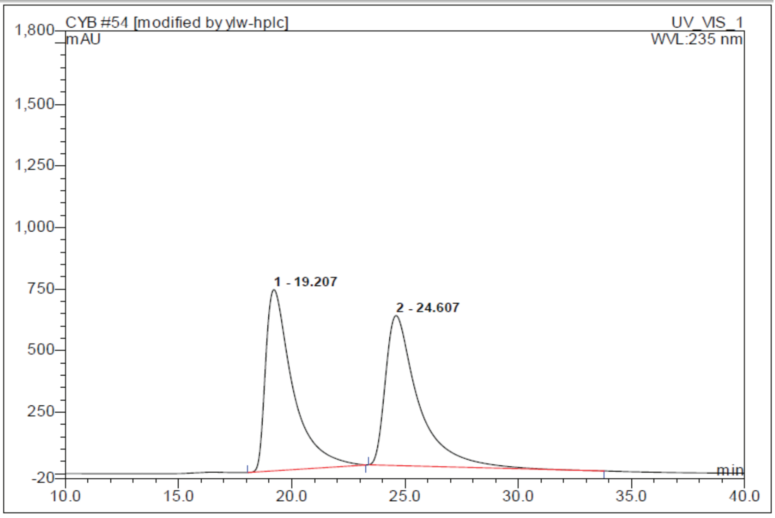

No.	Ret.Time min	Peak Name	Height mAU	Area mAU*min	Rel.Area %	Amount n.a.	Type
1	19.21	n.a.	735.733	1025.693	50.00	n.a.	BMB*
2	24.61	n.a.	608.879	1025.516	50.00	n.a.	BMB*
Total:			1344.613	2051.209	100.00	0.000	

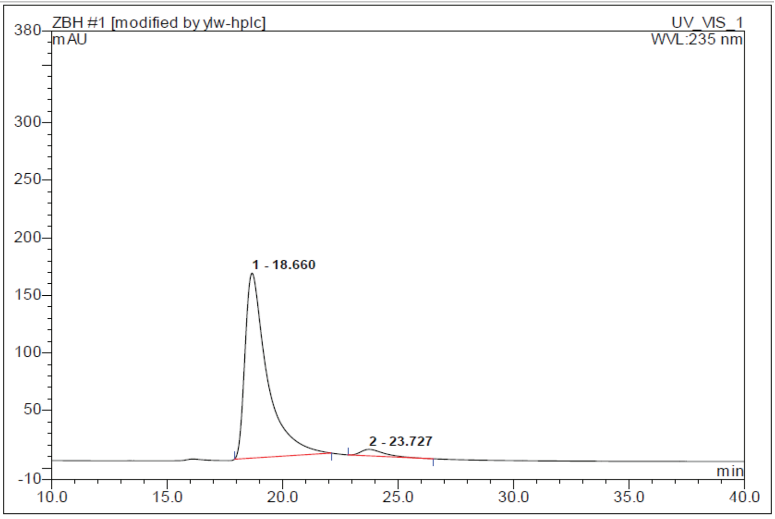

No.	Ret.Time min	Peak Name	Height mAU	Area mAU*min	Rel.Area %	Amount n.a.	Type
1	18.66	n.a.	160.709	179.191	96.48	n.a.	BMB*
2	23.73	n.a.	5.615	6.528	3.52	n.a.	BMB*
Total:			166.324	185.719	100.00	0.000	

Supplementary Figure 106. HPLC spectrum for compound (*S*)-1p'

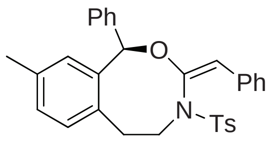

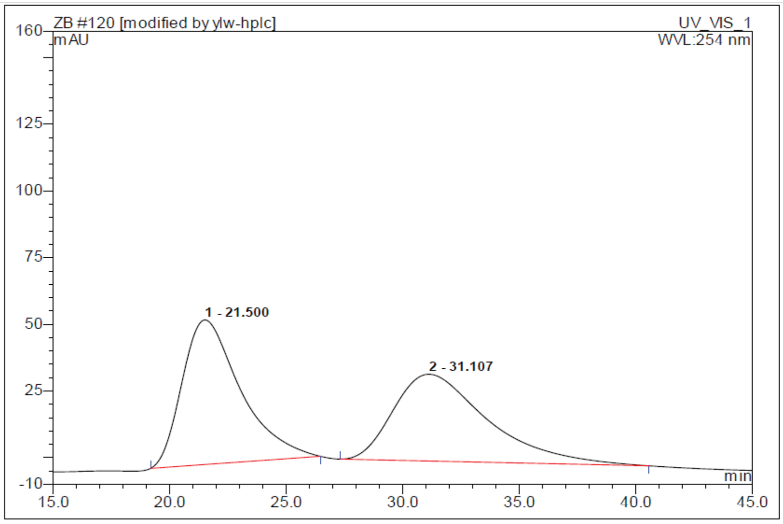

No.	Ret.Time min	Peak Name	Height mAU	Area mAU*min	Rel.Area %	Amount n.a.	Type
1	21.50	n.a.	54.216	157.360	50.94	n.a.	BMB*
2	31.11	n.a.	32.553	151.555	49.06	n.a.	BMB*
Total:			86.770	308.915	100.00	0.000	

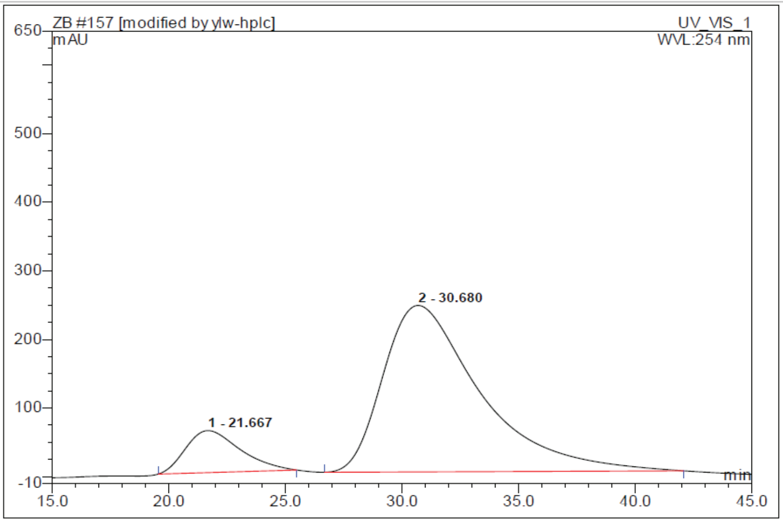

No.	Ret.Time min	Peak Name	Height mAU	Area mAU*min	Rel.Area %	Amount n.a.	Type
1	21.67	n.a.	61.269	165.574	12.37	n.a.	BMB*
2	30.68	n.a.	242.713	1172.507	87.63	n.a.	BMB*
Total:			303.982	1338.081	100.00	0.000	

Supplementary Figure 107. HPLC spectrum for compound (*R*)-6p

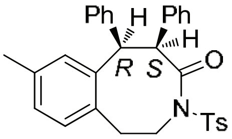

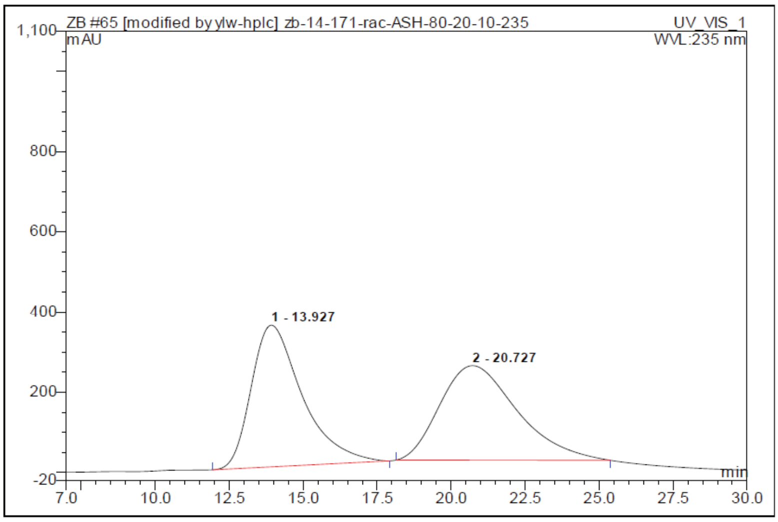

No.	Ret.Time min	Peak Name	Height mAU	Area mAU*min	Rel.Area %	Amount n.a.	Type
1	13.93	n.a.	353.050	704.688	49.70	n.a.	BMB*
2	20.73	n.a.	235.496	713.245	50.30	n.a.	BMB*
Total:			588.546	1417.933	100.00	0.000	

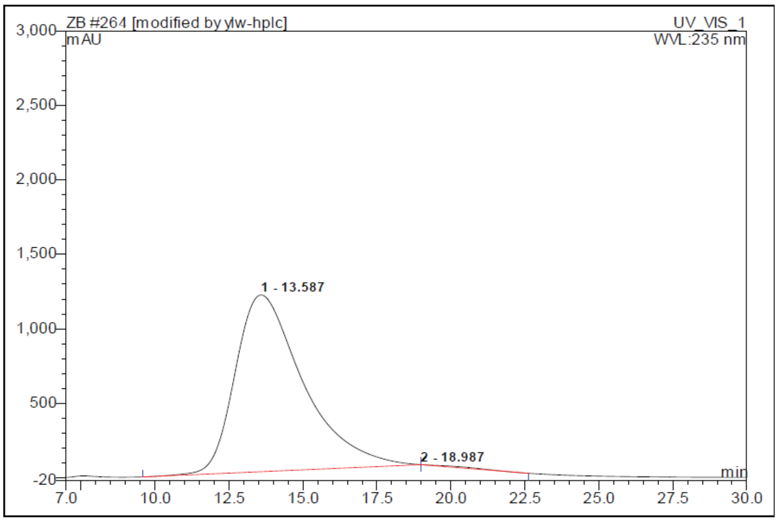

No.	Ret.Time min	Peak Name	Height mAU	Area mAU*min	Rel.Area %	Amount n.a.	Type
1	13.59	n.a.	1187.794	3170.963	99.63	n.a.	BMb*
2	18.99	n.a.	0.024	11.624	0.37	n.a.	bMB*
Total:			1187.818	3182.587	100.00	0.000	

Supplementary Figure 108. HPLC spectrum for compound (*R*, *S*)-2p, prepared from (*R*)-1p (e.r. 95:5) with Cat. 3

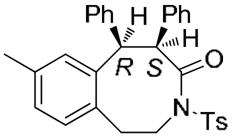

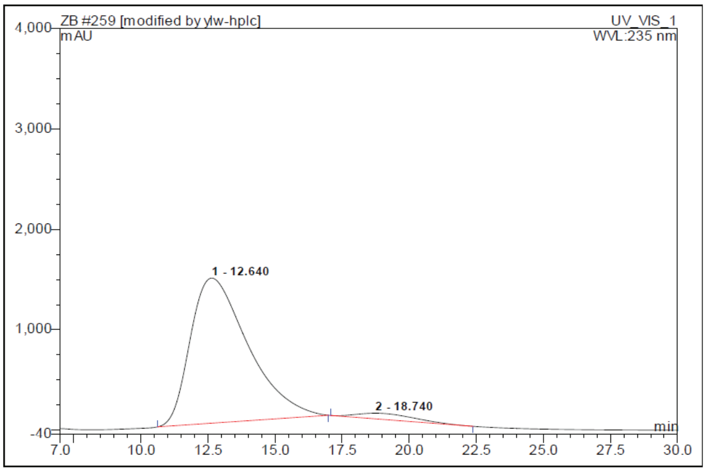

No.	Ret.Time<br>min	Peak Name	Height<br>mAU	Area<br>mAU*min	Rel.Area<br>%	Amount<br>n.a.	Type
1	12.64	n.a.	1444.777	3606.924	96.11	n.a.	BMB*
2	18.74	n.a.	56.251	145.862	3.89	n.a.	BMB*
Total:			1501.028	3752.785	100.00	0.000	

Supplementary Figure 109. HPLC spectrum for compound (*R*, *S*)-2p, prepared from (*R*)-1p (e.r. 95:5) with HOTf

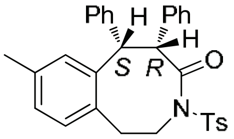

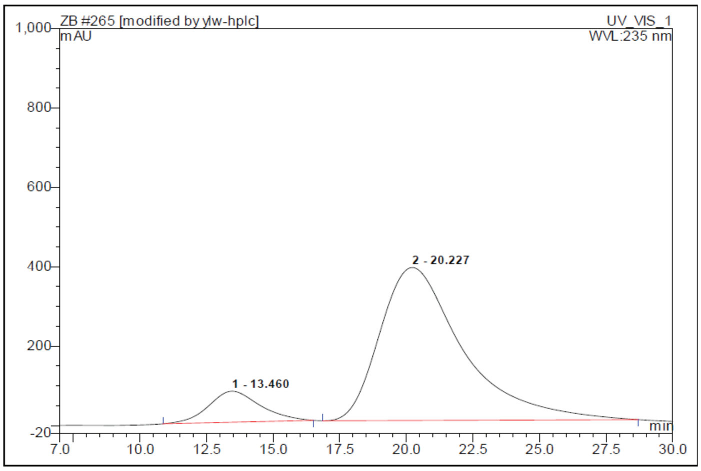

No.	Ret.Time<br>min	Peak Name	Height<br>mAU	Area<br>mAU*min	Rel.Area<br>%	Amount<br>n.a.	Type
1	13.46	n.a.	78.179	175.306	11.20	n.a.	BMB*
2	20.23	n.a.	385.289	1389.695	88.80	n.a.	BMB*
Total:			463.469	1565.001	100.00	0.000	

Supplementary Figure 110. HPLC spectrum for compound (*S*, *R*)-2p, prepared from (*S*)-1p (e.r. 96:4) with Cat. 3

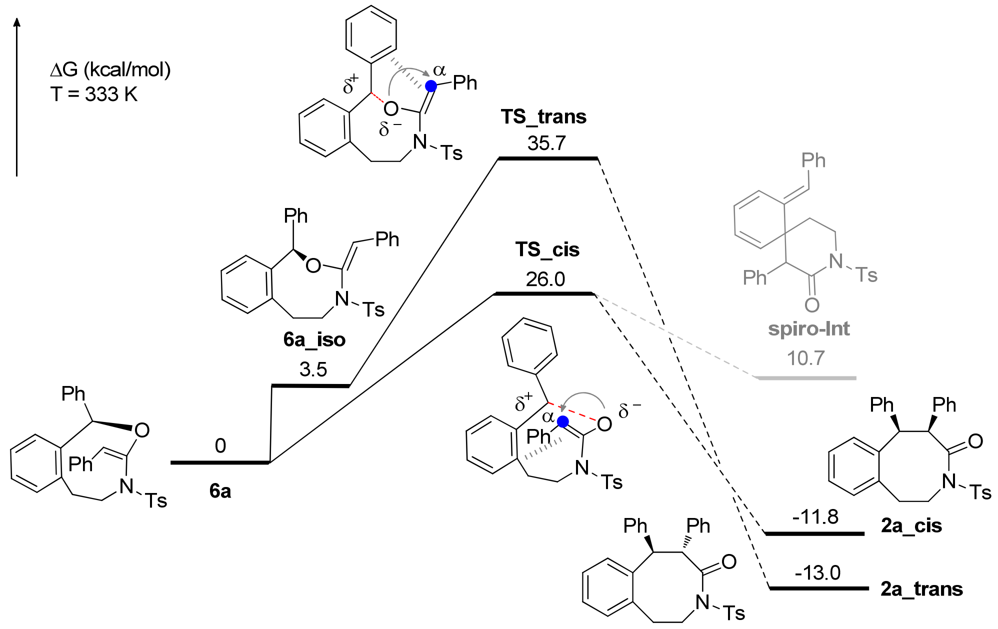

Supplementary Figure 111. Free energy profiles of the reaction using B3LYP-D3. The free energies with solvation corrections (solvent = chlorobenzene) and temperature corrections are given in kcal/mol.

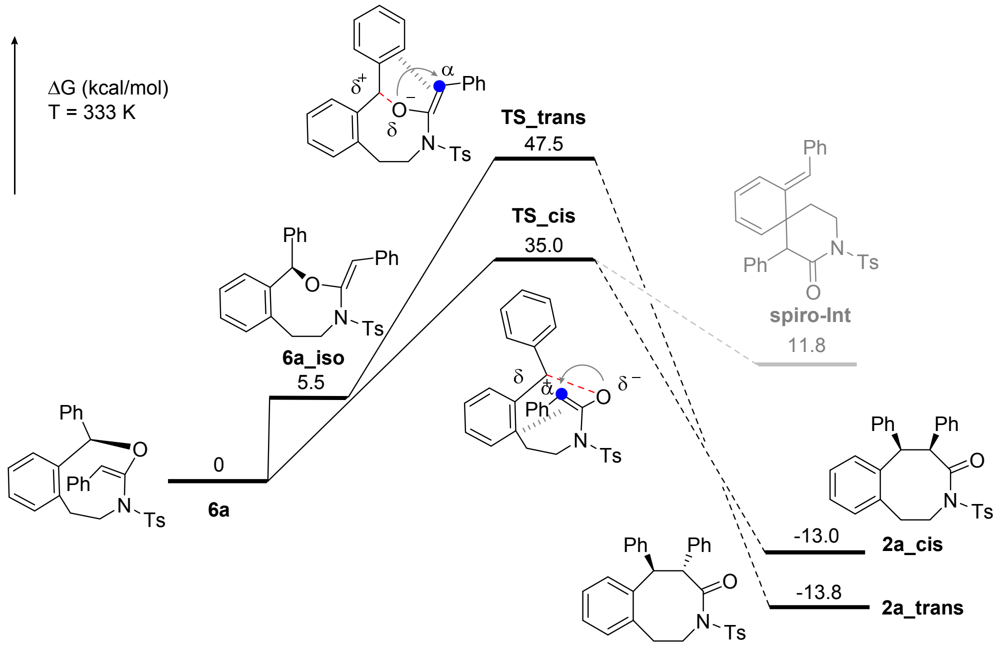

Supplementary Figure 112. Free energy profiles of the reaction using M062X. The free energies with solvation corrections (solvent = chlorobenzene) and temperature corrections are given in kcal/mol.

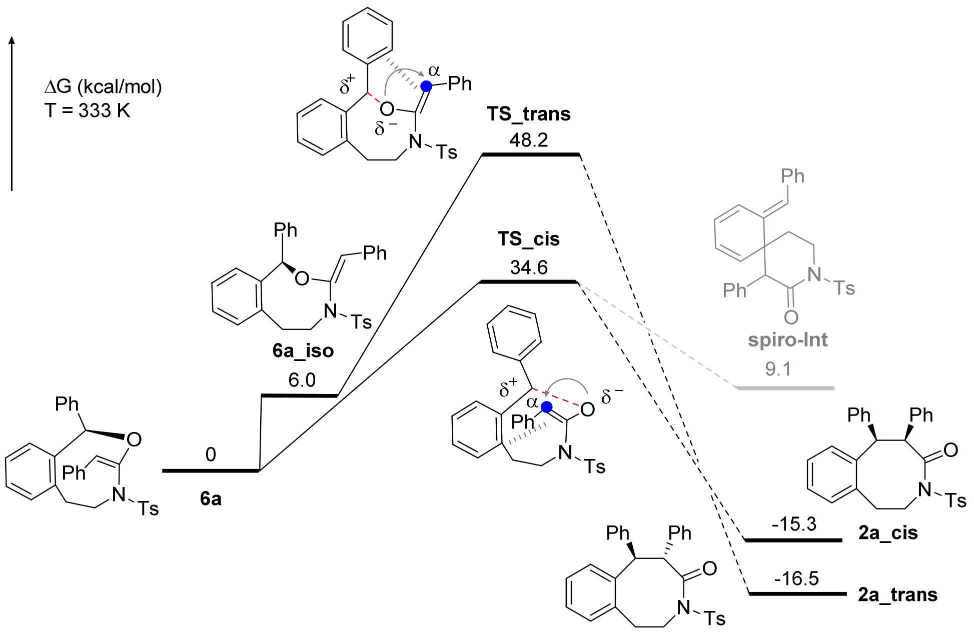

Supplementary Figure 113. Free energy profiles of the reaction using ωB97XD . The free energies with solvation corrections (solvent = chlorobenzene) and temperature corrections are given in kcal/mol.

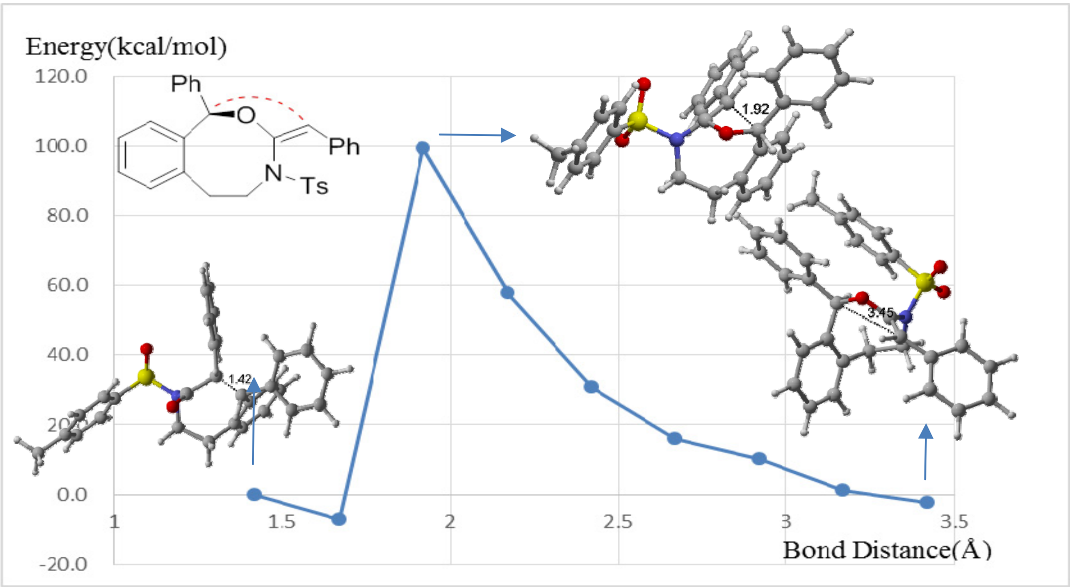

Supplementary Figure 114. Energy scans depended on C-C bond distance to form possible transition states of [1,3]-rearrangement. Due to extremely high energy barrier, direct [1,3] O-to-C rearrangement seems not to be accessible in our case at the B3LYP-D3 level of theory.

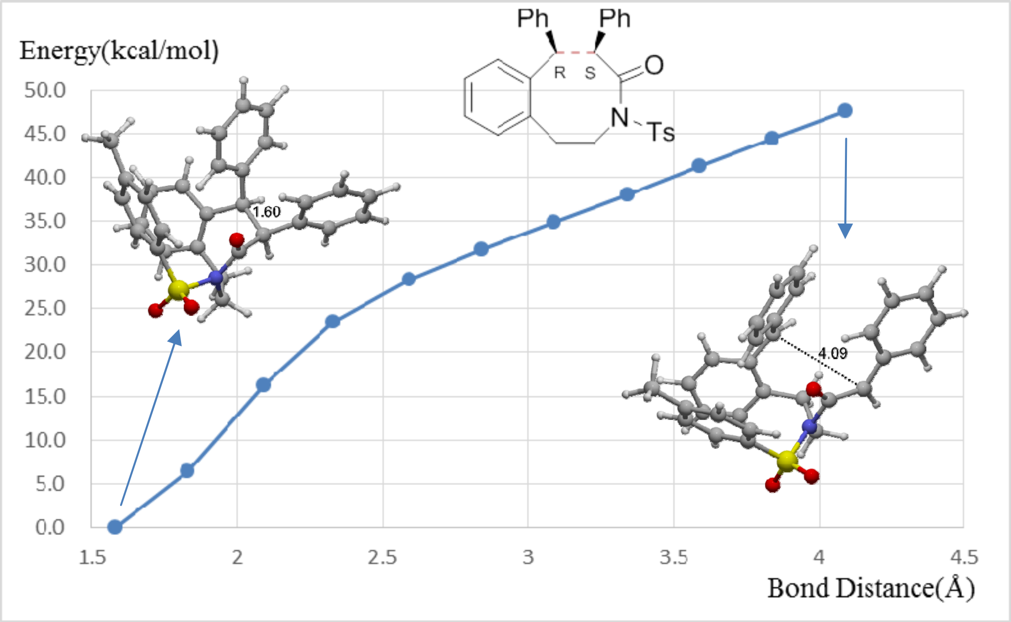

Supplementary Figure 115. Energy scans depended on C-C bond distance to form **2a-cis**. The figure indicates the process of C-C bond formation is exothermic without an apparent transition state at the B3LYP-D3 level of theory.

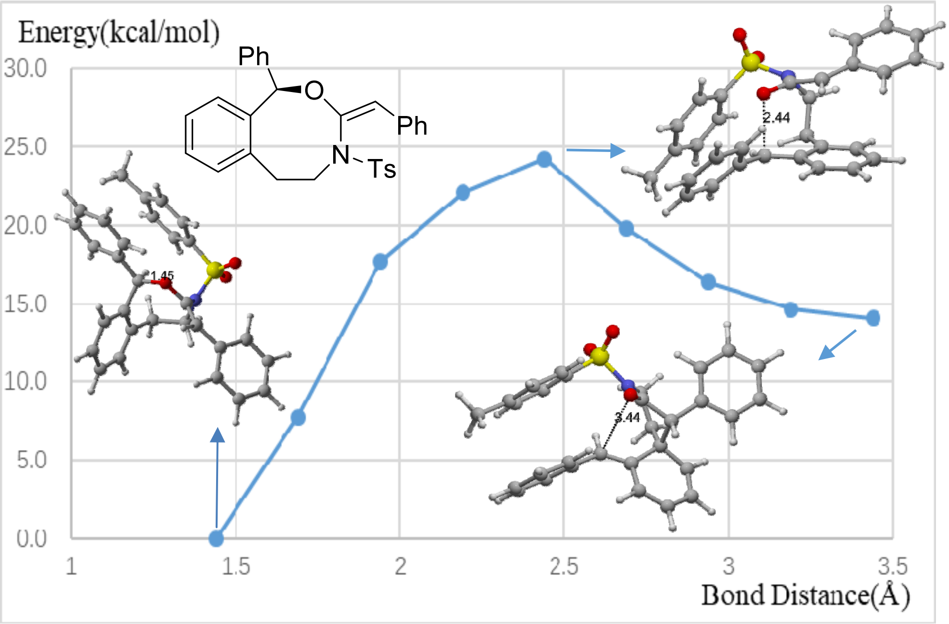

Supplementary Figure 116. Energy scans depended on C-O bond distance to form spiro-Int via [3,3]-rearrangement at the B3LYP-D3 level of theory.

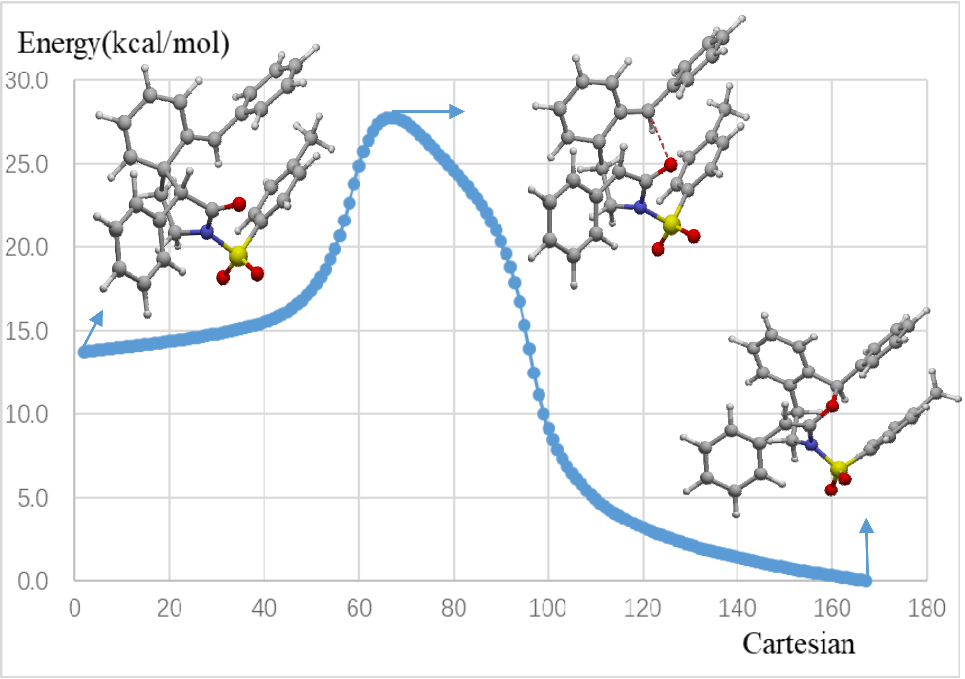

Supplementary Figure 117. The intrinsic reaction coordinate (IRC) of the calculated reaction at the B3LYP-D3 level of theory.

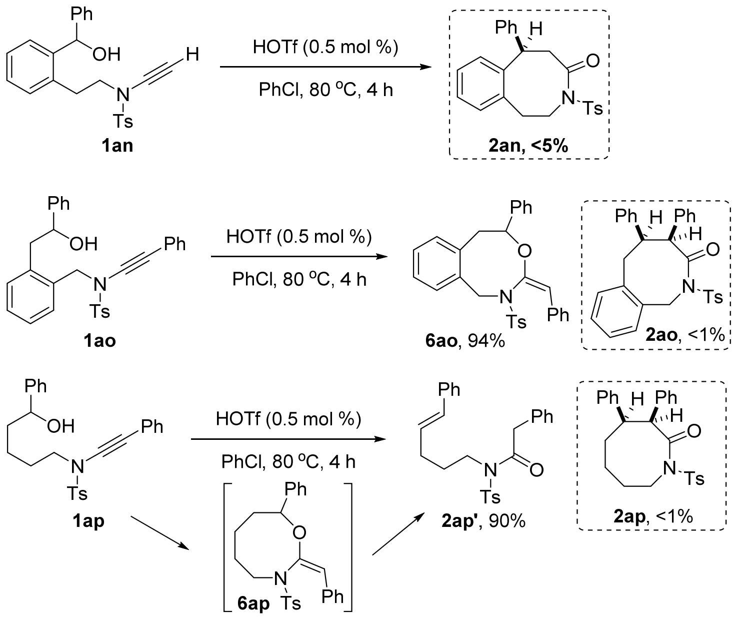

Supplementary Figure 118. Ynamides **1an-1ap** which failed to give the desired products.

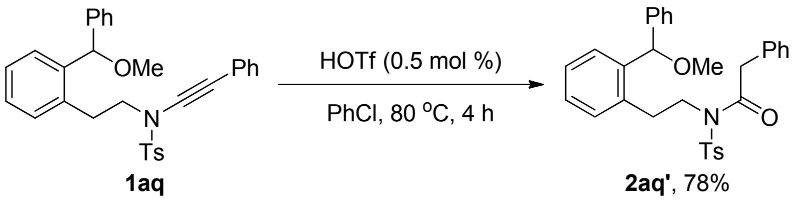

Supplementary Figure 119. HOTf-catalyzed cascade cyclization of ynamide **1aq**.

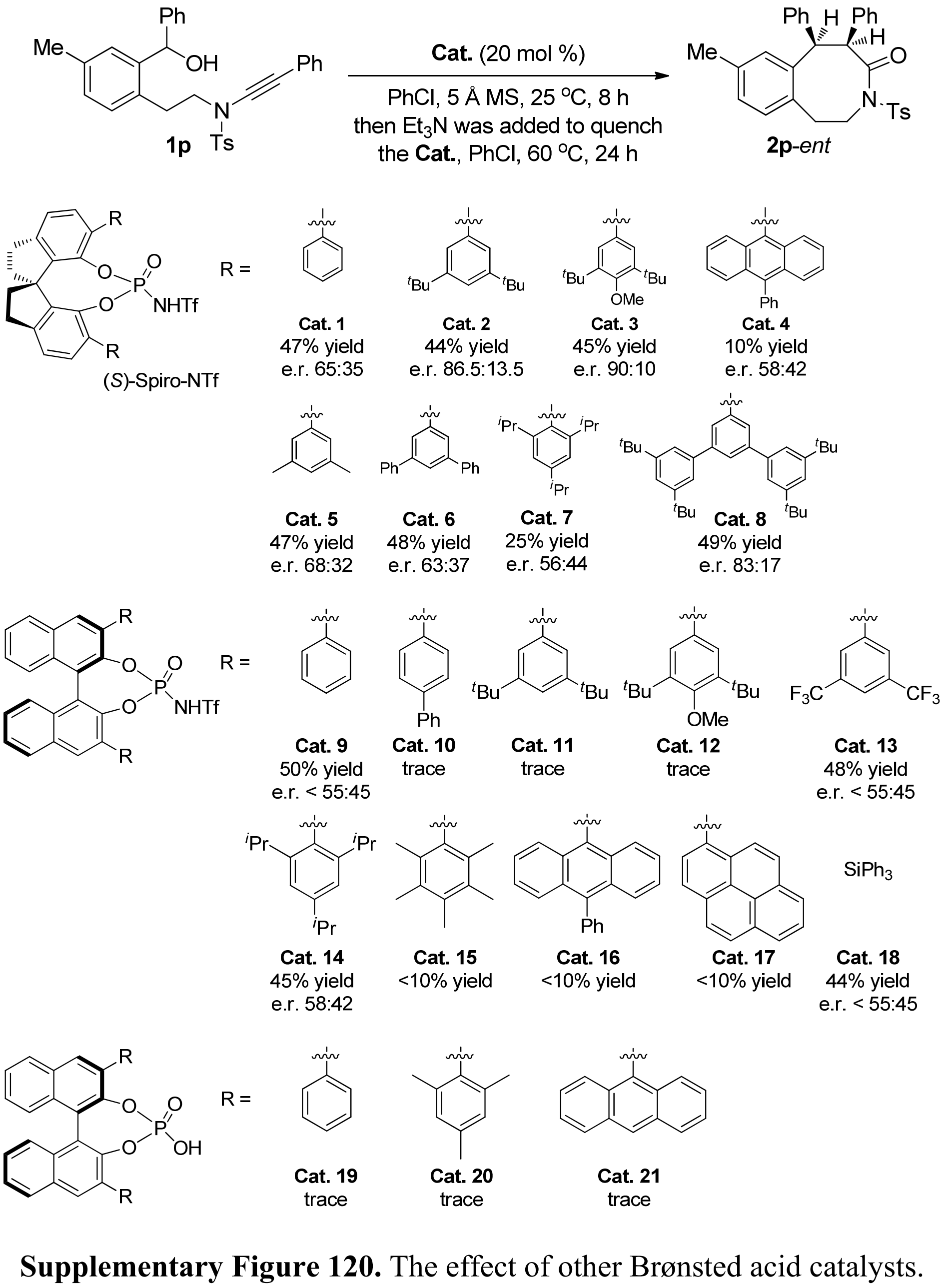

Supplementary Figure 120. The effect of other Brønsted acid catalysts.

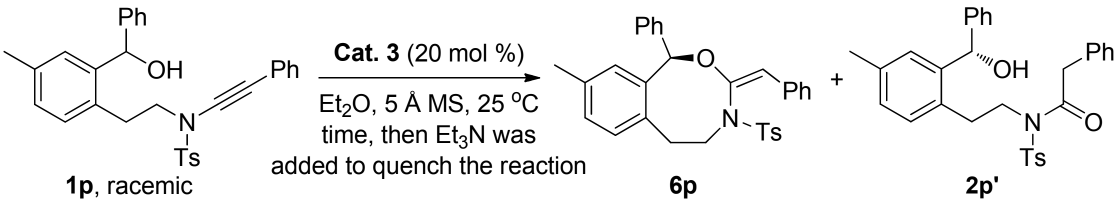

entry[a]	time	conversion	yield of **6p**	e.r. of **6p**	yield of **2p'**	e.r. of **2p'**
1	3 h	35%	18%	93:7	16%	80:20
2	8 h	84%	43%	90.5:9.5	40%	82:18
3[b]	9 h	92%	44%	87:13	41%	79:21
4[c]	12 h	100%	46%	85:15	44%	79:21

[a] Yields are measured by ^{1}H NMR using diethyl phthalate as internal standard; ers are determined by HPLC analysis on a chiral stationary phase. [b] **2p** was obtained in 7% yield. [c] **2p** was obtained in 9% yield.

Supplementary Figure 121. Control experiments on the transformation of racemic ynamide **1p**.

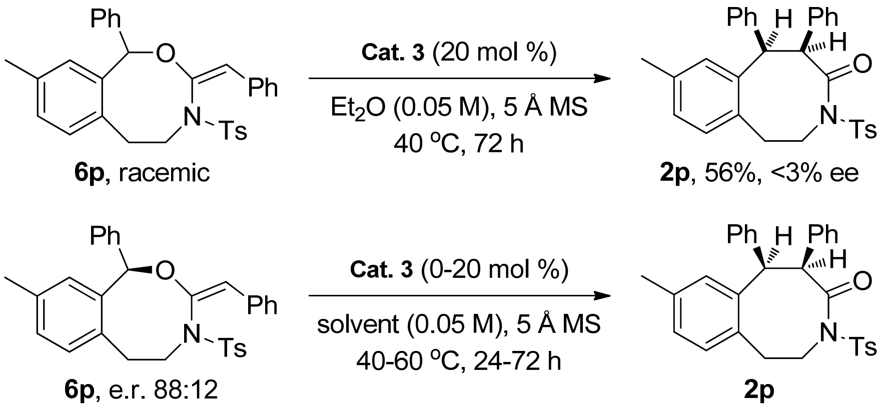

Cat. 3 (20 mol %), Et_2O, 40 °C, 72 h: **2p**, 53%, e.r. 90:10
no catalyst, Et_2O, 40 °C, 72 h: **2p**, 54%, e.r. 90:10
no catalyst, Et_2O, 40 °C, 48 h: **2p**, 36%, e.r. 90:10 (recovered **6p**, e.r. 88:12)
no catalyst, PhCl, 60 °C, 24 h: **2p**, 85%, e.r. 91:9

Supplementary Figure 122. Control experiments on the transformation of **6p** into **2p**.

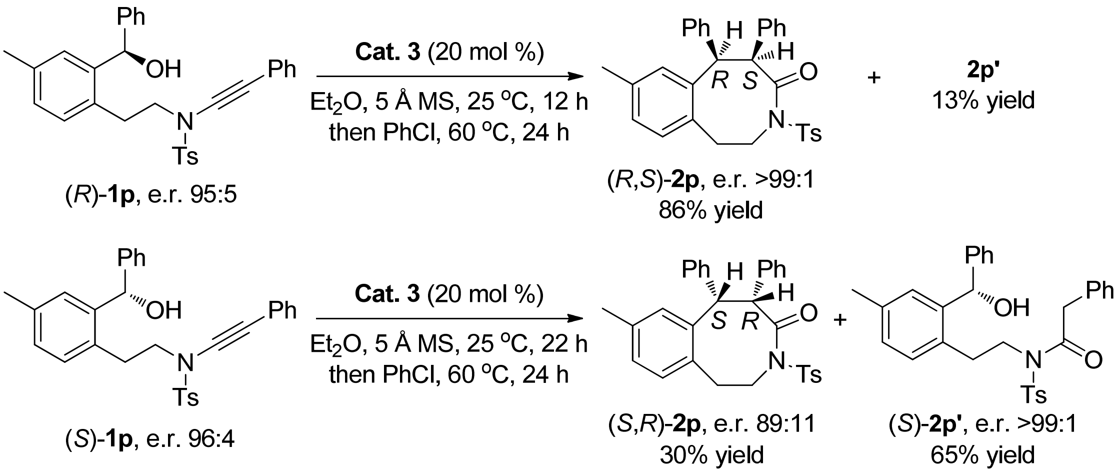

Supplementary Figure 123. Chiral Brønsted acid-catalyzed cascade cyclization of chiral ynamide **1p**.

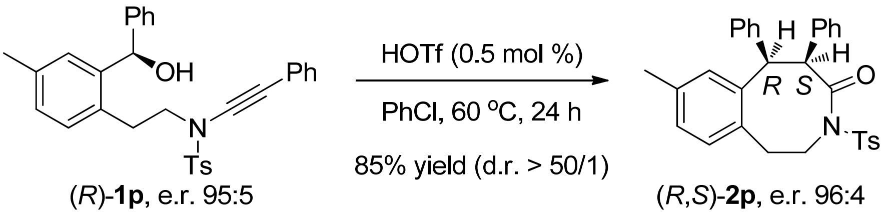

Supplementary Figure 124. HOTf-catalyzed cascade cyclization of chiral ynamide **1p**.

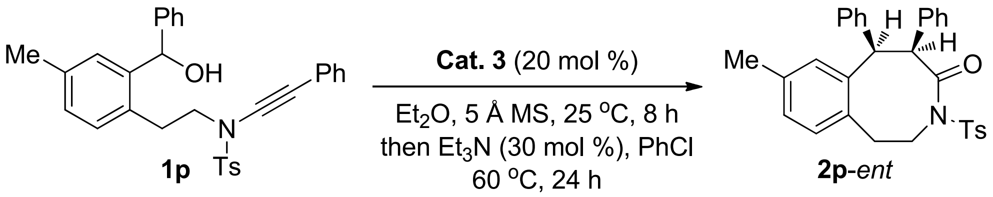

time	1	2	3	4	5
e.r.	95:5	95:5	95.5:4.5	95:5	94:6
yield%	42	44	40	42	44

Supplementary Figure 125. Studies on the reuse of the chiral catalyst.

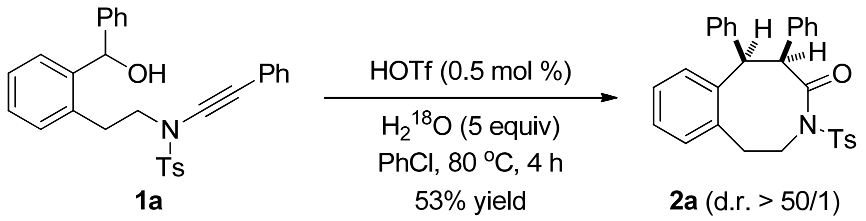

Supplementary Figure 126. $H_2{}^{18}O$ isotopic labeling study.

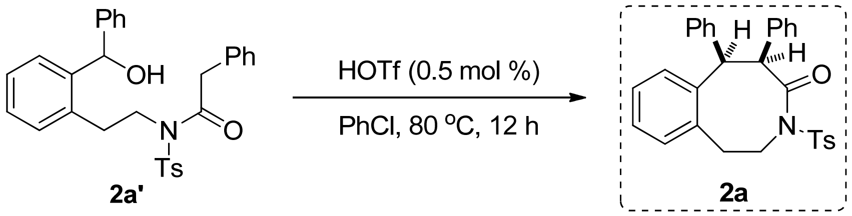

Supplementary Figure 127. Attempts on the conversion of **2a′** into **2a**.

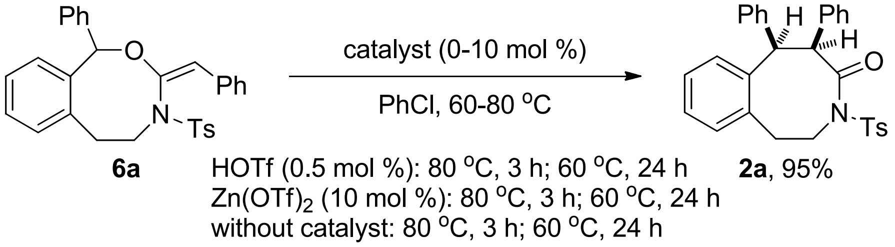

Supplementary Figure 128. Control experiments on the transformation of **6a** into **2a**.

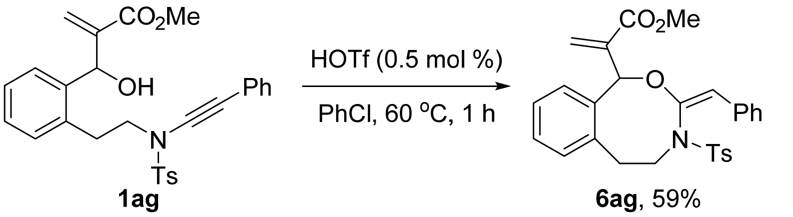

Supplementary Figure 129. HOTf-catalyzed transformation of **1ag** into **6ag**.

Supplementary Tables

Supplementary Table 1. Other reaction condition studies[a]

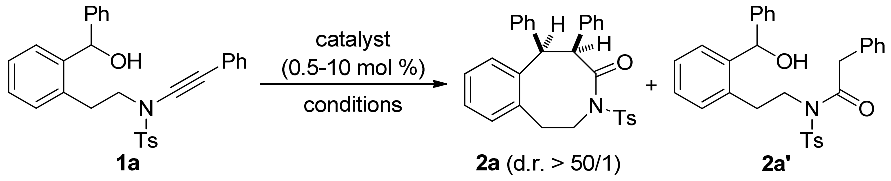

entry	catalyst	reaction conditions	yield (%)[b] 2a	2a'
1[c]	$Zn(OTf)_2$ (10 mol %)	PhCl, 80 °C, 4 h	92	<1
2[c]	MsOH (10 mol %)	PhCl, 80 °C, 4 h	81	<1
3[c,d]	HOTf (0.5 mol %)	PhCl, 80 °C, 12 h	<5	<1
4	$HNTf_2$ (10 mol %)	PhCl, 80 °C, 4 h	<5	<1
5[d]	$HNTf_2$ (1 mol %)	PhCl, 80 °C, 12 h	<5	<1
6[d]	$HNTf_2$ (0.5 mol %)	PhCl, 80 °C, 12 h	<5	<1
7[d]	/	PhCl, 80 °C, 24 h	<1	<1
8	HOTf (0.5 mol %)	DCE, 80 °C, 18 h	62	<1
9	HOTf (0.5 mol %)	$MeNO_2$, 80 °C, 4 h	56	<1
10	HOTf (0.5 mol %)	PhCl, 60 °C, 24 h	90	<1
11[d]	HOTf (0.2 mol %)	PhCl, 80 °C, 24 h	<5	<1

[a] Reaction conditions: [**1a**] = 0.05 M. [b] Measured by ^{1}H NMR using diethyl phthalate as the internal standard. [c] Using 5 Å MS (60 mg/0.1 mmol) as additive. [d] > 95% of **1a** remained unreacted.

Supplementary Table 2. The effect of reaction time when Et_3N was added to quench the first step[a]

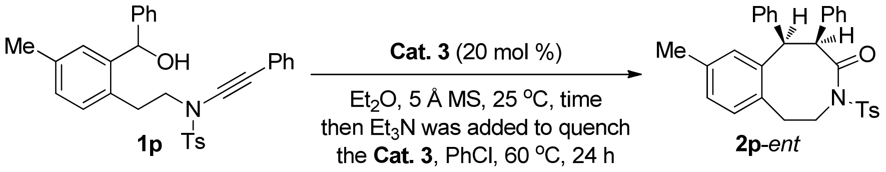

entry	time (h)	yield (%)[b]	e.r.[c]
1	3	18 (35% conversion)	96:4
2	8	42 (84% conversion)	95:5
3	9	46 (92% conversion)	91:9
4	12	57 (100% conversion)	90:10

[a] Reaction conditions: **1p** (0.1 mmol), **Cat. 3** (0.02 mmol), Et_2O (2 mL), 25 °C, 3-12 h, then Et_3N (0.03 mmol), PhCl (1 mL), 60 °C, 24 h, in vials. [b] Isolated yields. [c] Determined by HPLC analysis on a chiral stationary phase.

Supplementary Table 3. Crystal data and structure refinement for 2a. CCDC Number = 1880379

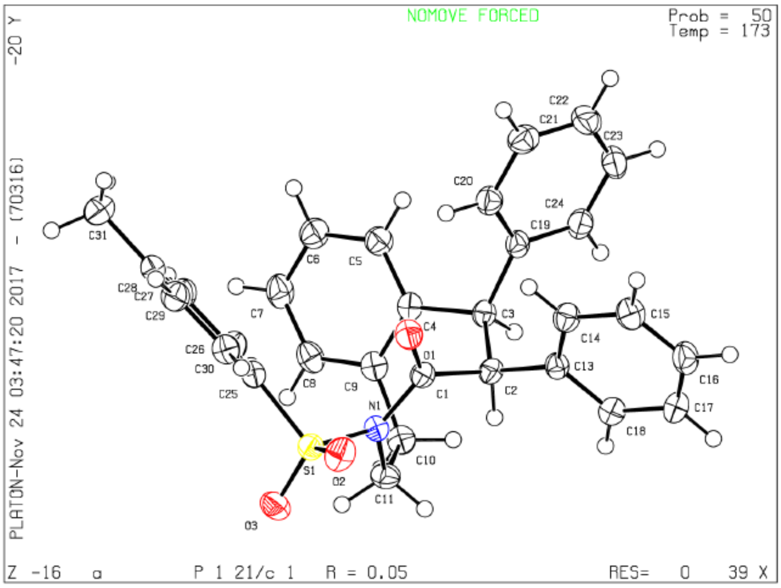

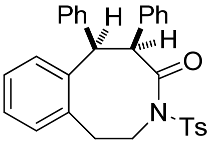

Bond precision: C-C = 0.0030 A   Wavelength=0.71073

Cell:	a=22.762(3)	b=8.4040(11)	c=12.0766(15)
	alpha=90	beta=96.442(2)	gamma=90
Temperature:	173 K		

	Calculated	Reported
Volume	2295.6(5)	2295.6(5)
Space group	P 21/c	P 1 21/c 1
Hall group	-P 2ybc	-P 2ybc
Moiety formula	C30 H27 N O3 S	C30 H27 N O3 S
Sum formula	C30 H27 N O3 S	C30 H27 N O3 S
Mr	481.59	481.58
Dx,g cm-3	1.393	1.393
Z	4	4
Mu (mm-1)	0.176	0.176
F000	1016.0	1016.0
F000’	1016.92	
h,k,lmax	29,10,15	29,10,15
Nref	5257	5212
Tmin,Tmax	0.919,0.949	
Tmin’	0.914	

Correction method= Not given

Data completeness= 0.991   Theta(max)= 27.484

R(reflections)= 0.0536(4708)   wR2(reflections)= 0.1478(5212)

S = 1.161   Npar= 317

Supplementary Table 4. Crystal data and structure refinement for 2ac. CCDC Number = 1880411

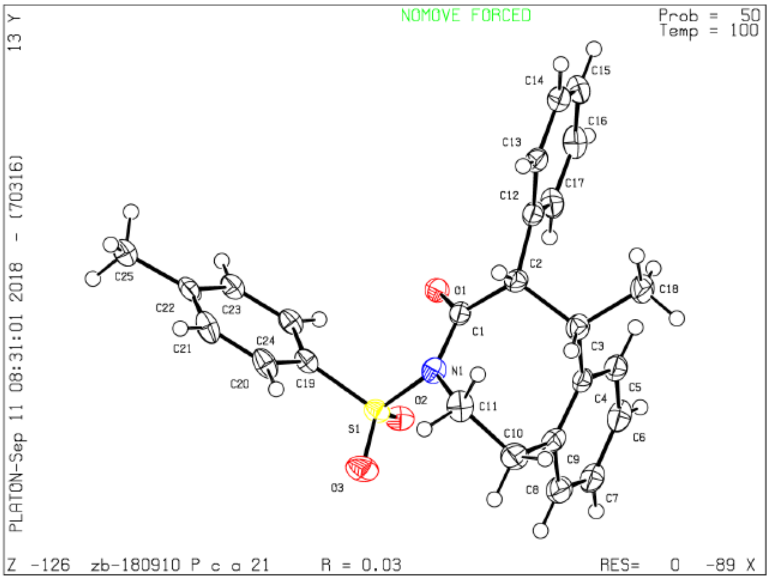

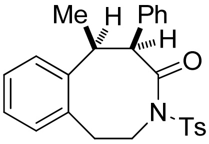

Bond precision: C-C = 0.0041 A Wavelength=1.54184

Cell:	a=15.2812(3)	b=6.2107(1)	c=22.0986(4)
	alpha=90	beta=90	gamma=90
Temperature:	100 K		

	Calculated	Reported
Volume	2097.31(7)	2097.31(7)
Space group	P c a 21	P c a 21
Hall group	P 2c -2ac	P 2c -2ac
Moiety formula	C25 H25 N O3 S	C25 H25 N O3 S
Sum formula	C25 H25 N O3 S	C25 H25 N O3 S
Mr	419.52	419.52
Dx,g cm-3	1.329	1.329
Z	4	4
Mu (mm-1)	1.587	1.587
F000	888.0	888.0
F000'	891.75	
h,k,lmax	18,7,27	18,7,26
Nref	4101[2109]	3838
Tmin,Tmax	0.859,0.881	0.815,1.000
Tmin'	0.853	

Correction method= # Reported T Limits: Tmin=0.815 Tmax=1.000
AbsCorr = MULTI-SCAN

Data completeness= 1.82/0.94 Theta(max)= 71.527

R(reflections)= 0.0318(3715) wR2(reflections)= 0.1039(3838)

S = 0.915 Npar= 273

Supplementary Table 5. Crystal data and structure refinement for 2ai. CCDC Number = 1880414

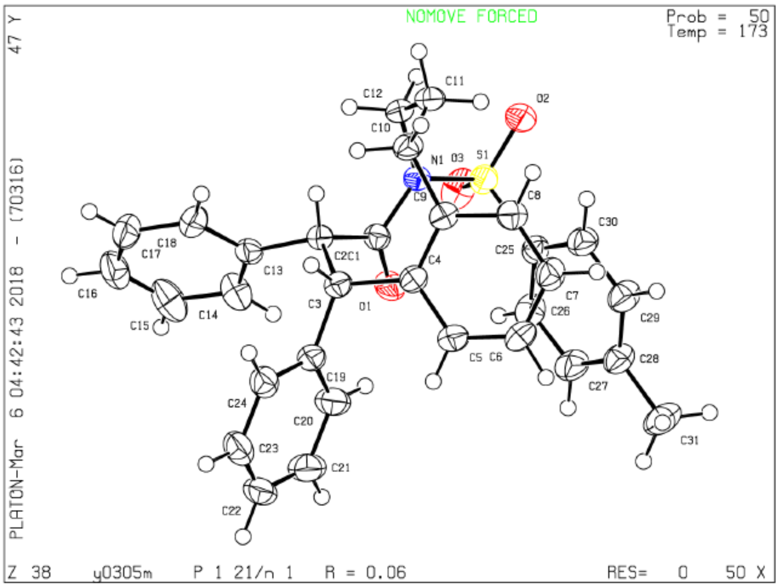

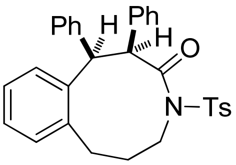

Bond precision: C-C = 0.0032 A Wavelength=0.71073

Cell:	a=8.5854(16)	b=21.341(4)	c=14.045(3)
	alpha=90	beta=104.004(3)	gamma=90
Temperature:	173 K		

	Calculated	Reported
Volume	2496.9(8)	2496.9(8)
Space group	P 21/n	P 1 21/n 1
Hall group	-P 2yn	-P 2yn
Moiety formula	C31 H29 N O3 S	C31 H29 N O3 S
Sum formula	C31 H29 N O3 S	C31 H29 N O3 S
Mr	495.61	495.61
Dx,g cm-3	1.318	1.318
Z	4	4
Mu (mm-1)	0.164	0.164
F000	1048.0	1048.0
F000'	1048.92	
h,k,lmax	11,27,18	11,27,18
Nref	5725	5671
Tmin,Tmax	0.971,0.984	
Tmin'	0.968	

Correction method= Not given

Data completeness= 0.991 Theta(max)= 27.485

R(reflections)= 0.0576(5280) wR2(reflections)= 0.1431(5671)

S = 1.160 Npar= 346

Supplementary Table 6. Crystal data and structure refinement for 2p-*ent*. CCDC Number = 1887308

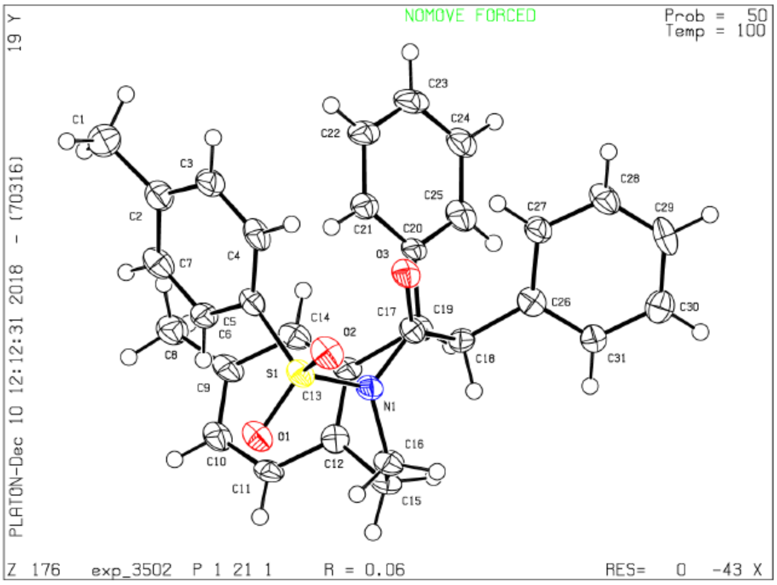

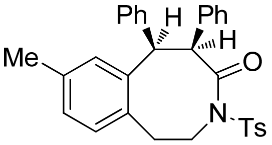

Bond precision: C-C = 0.0098 A Wavelength=1.54184

Cell:	a=11.6192(7)	b=8.1266(4)	c=13.9581(6)
	alpha=90	beta=107.818(6)	gamma=90
Temperature:	100 K		

	Calculated	Reported
Volume	1254.77(12)	1254.77(12)
Space group	P 21	P 1 21 1
Hall group	P 2yb	P 2yb
Moiety formula	C31 H29 N O3 S	C31 H29 N O3 S
Sum formula	C31 H29 N O3 S	C31 H29 N O3 S
Mr	495.61	495.61
Dx,g cm-3	1.312	1.312
Z	2	2
Mu (mm-1)	1.412	1.412
F000	524.0	524.0
F000'	526.08	
h,k,lmax	13,9,16	13,9,16
Nref	4280[2308]	3877
Tmin,Tmax	0.868,0.932	0.784,1.000
Tmin'	0.868	

Correction method= # Reported T Limits: Tmin=0.784 Tmax=1.000
AbsCorr = MULTI-SCAN

Data completeness= 1.68/0.91 Theta(max)= 65.085

R(reflections)= 0.0611(3285) wR2(reflections)= 0.1517(3877)

S = 1.036 Npar= 327

Supplementary Table 7. Crystal data and structure refinement for 6ag. CCDC Number = 1887311

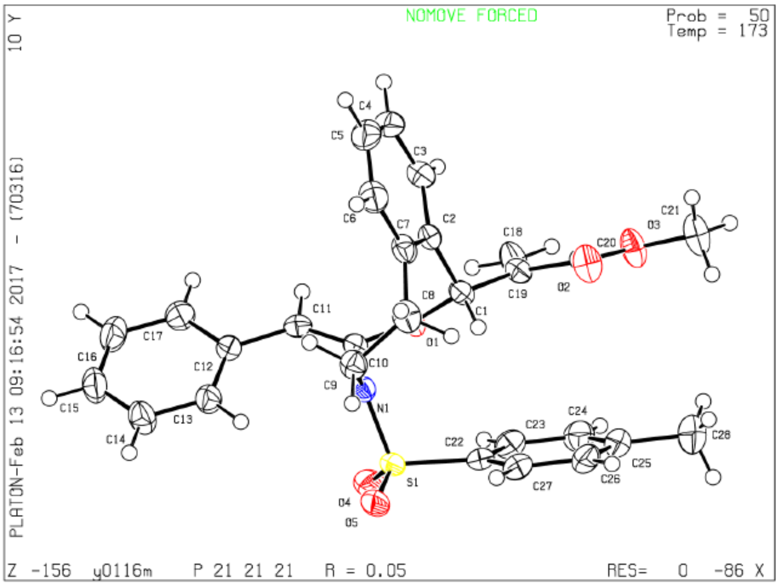

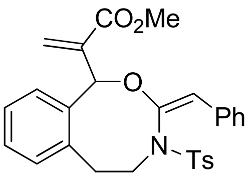

Bond precision:	C-C = 0.0045 A	Wavelength=0.71073	
Cell:	a=11.0930(19)	b=11.2707(19)	c=19.181(3)
	alpha=90	beta=90	gamma=90
Temperature:	173 K		

	Calculated	Reported
Volume	2398.1(7)	2398.2(7)
Space group	P 21 21 21	P 21 21 21
Hall group	P 2ac 2ab	P 2ac 2ab
Moiety formula	C28 H27 N O5 S	C28 H27 N O5 S
Sum formula	C28 H27 N O5 S	C28 H27 N O5 S
Mr	489.57	489.56
Dx,g cm-3	1.356	1.356
Z	4	4
Mu (mm-1)	0.176	0.176
F000	1032.0	1032.0
F000'	1032.98	
h,k,lmax	14,15,25	15,15,25
Nref	6173[3466]	5752
Tmin,Tmax	0.869,0.900	
Tmin'	0.869	

Correction method= Not given

Data completeness= 1.66/0.93 Theta(max)= 28.672

R(reflections)= 0.0466(4935) wR2(reflections)= 0.1103(5752)

S = 1.023 Npar= 318

Supplementary Table 8. The cytotoxic effects of the newly synthesized medium-sized lactam compounds against cancer cells[a]

Cmpd ID	Cell viability at 20 μM (%)				
	A375	SK-GT-4	KYSE-450	MCF-7	MDA-MB-231
**2a**	87.88	77.59	90.68	87.25	95.63
**2b**	51.93	71.03	87.66	86.65	104.44
**2c**	43.67	67.15	62.13	61.99	111.49
**2d**	11.95	55.26	86.15	61.95	110.85
**2e**	65.28	81.64	79.19	80.81	116.52
**2f**	67.52	88.55	80.15	75.58	115.89
**2g**	81.66	91.13	86.09	84.81	109.12
**2h**	39.66	98.32	92.16	64.58	121.84
**2i**	78.06	82.88	88.96	87.24	102.28
**2j**	74.75	82.53	88.68	89.13	90.63
**2k**	74.78	85.23	90.19	88.56	107.79
**2l**	67.43	90.09	85.34	81.45	98.51
**2m**	98.09	94.38	83.10	91.14	97.06
**2n**	72.46	87.47	84.59	92.49	93.68
**2o**	85.99	99.47	87.11	89.20	104.61
**2p**	52.70	86.39	80.07	78.64	99.63
**2q**	45.77	68.96	73.07	82.75	98.46
**2r**	46.93	70.24	79.37	84.03	99.54
**2s**	23.63	80.55	86.28	92.71	91.45
**2t**	73.81	71.61	82.42	94.27	82.21
**2u**	38.85	53.22	57.54	63.78	102.61
**2v**	46.54	62.52	65.04	78.32	112.98
**2w**	55.09	71.57	81.04	76.14	94.17
**2x**	77.52	71.09	83.22	91.31	91.38
**2y**	45.26	62.19	67.50	63.29	104.77
**2z**	69.59	85.32	75.67	95.72	86.32
**2aa**	19.13	60.79	87.83	88.07	94.56
**2ab**	54.69	77.65	71.69	81.17	101.28
**2ac**	19.11	30.03	71.57	50.08	74.08
**2ad**	23.90	87.75	89.07	67.61	86.27
**2ae**	76.10	73.44	84.68	88.10	84.18
**2ah**	15.80	24.88	62.02	57.02	63.87
**2ai**	85.76	78.22	81.78	95.87	94.42
**2aj**	86.04	87.62	71.29	104.29	93.82
**2ak**	17.55	94.65	89.75	99.09	94.83
**2al**	18.01	74.82	91.44	71.96	89.83
**2am**	18.14	97.43	93.84	46.68	99.89
**2p-*ent***	87.19	43.30	68.71	99.45	93.46
**3a**	75.00	37.16	83.94	98.68	96.47
**4a**	68.80	104.40	90.05	77.19	93.89
**5a**	22.70	109.48	87.84	103.03	104.69

[a] Results are average of two experiments.

Supplementary Table 9. Selectivity factor for this chiral Brønsted acid-catalyzed kinetic resolution

**Product**	**Conversion (%)**	**Ee of product (%)**	***S* from product**
**2p**-*ent*	48	90	49.4
**2r**-*ent*	45	87	30.6
**2t**-*ent*	54	85	81.8
**2w**-*ent*	55	80	39.5
**2z**-*ent*	50	86	36.6
**2ab**-*ent*	51	92	93.0
**2a**-*ent*	51	78	20.0
**2u**-*ent*	49	78	18.0

Supplementary Methods

General Information

Unless otherwise noted, materials were obtained commercially and used without further purification. All the solvents were treated according to general methods. Flash column chromatography was performed over silica gel (300-400 mesh). ^{1}H NMR spectra and ^{13}C NMR spectra were recorded on a Bruker AV-400 spectrometer and a Bruker AV-500 spectrometer in chloroform-d_3. For ^{1}H NMR spectra, chemical shifts are reported in ppm with the internal TMS signal at 0.0 ppm as a standard. For ^{13}C NMR spectra, chemical shifts are reported in ppm with the internal chloroform signal at 77.0 ppm as a standard. Infrared spectra were recorded on a Nicolet AVATER FTIR330 spectrometer as thin film and are reported in reciprocal centimeter (cm^{-1}). Mass spectra were recorded with Micromass QTOF2 Quadrupole/Time-of-Flight Tandem mass spectrometer using electron spray ionization.

Cell Viability Assay

We also tested the newly synthesized 3-benzazocinones for their bioactivity as antitumor agents. The cytotoxic effects of these compounds were evaluated against a panel of cancer cells, including melanoma cells A375, esophageal cancer cells SK-GT-4 and KYSE-450, and breast cancer cells MCF-7 and MDA-MB-231 by the use of cell viability assay, using a commercially available proliferation assay kit (Promega, US). Briefly, the cells were plated in 96-well culture plates at an appropriate density in culture medium and allowed to attach overnight. After treatment of vehicle (0.1% DMSO as control) or test compounds for indicated times and concentrations, 20 μL of MTS reaction solution (3-(4, 5-dimethylthiazol-2-yl)-5-(3-carboxymethoxyphenyl)-2- (4-sulfophenyl)-2H-tetrazolium, inner salt; MTS (a) and 100 μg/mL phenazine methosulfate; PES) was added to each well. The absorbance values were read at 490 nm wavelength with a spectrophotometer (Varioskan Flash, Thermo, US) after 1 to 4 hours incubation. The cell viability was calculated as: cell survival = (ODcompd. - ODblank)/(ODcontrol - ODblank)*100%. The relevant results are summarized in Supplementary Table 8.

Compounds **1a-1p, 1u, 1w, 1y-z** were prepared according to the following procedures.[1-3]

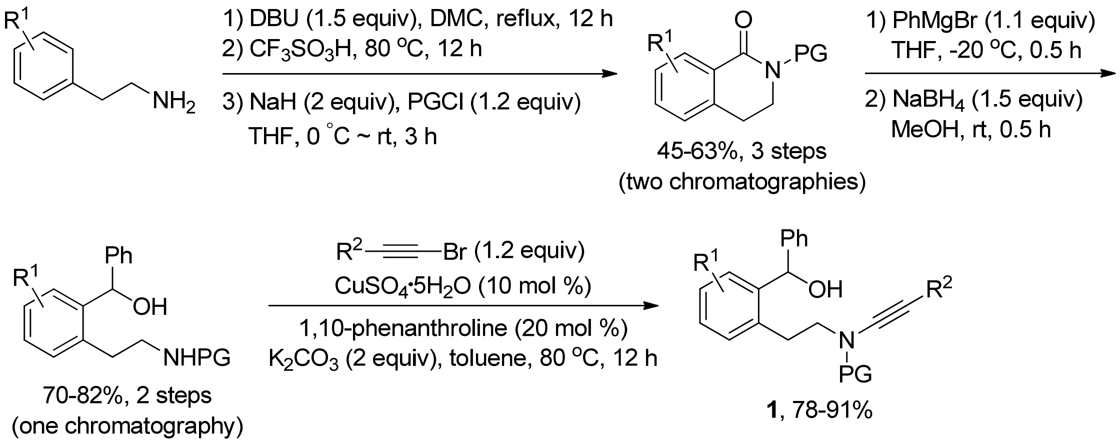

Supplementary Figure 130. Procedures for the preparation of ynamides **1a-1p, 1u, 1w, 1y-z**.

N-(2-(hydroxy(phenyl)methyl)phenethyl)-4-methyl-*N*-(phenylethynyl)benzenesulfonamide (1a)

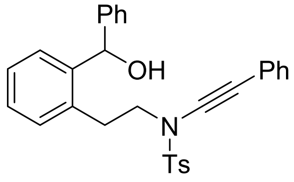

1a

Pale yellow oil. ^{1}H NMR (500 MHz, $CDCl_3$) δ 7.71 (d, *J* = 8.5 Hz, 2H), 7.42 (dd, *J* = 7.5 Hz, *J* = 2.0 Hz, 1H), 7.38 – 7.33 (m, 2H), 7.28 – 7.14 (m, 13H), 6.01 (s, 1H), 3.52 (ddd, *J* = 16.0 Hz, *J* = 10.0 Hz, *J* = 6.0 Hz, 1H), 3.42 (ddd, *J* = 16.0 Hz, *J* = 9.5 Hz, *J* = 6.0 Hz, 1H), 3.04 (ddd, *J* = 15.5 Hz, *J* = 9.5 Hz, *J* = 6.0 Hz, 1H), 2.96 (ddd, *J* = 16.0 Hz, *J* = 10.0 Hz, *J* = 6.0 Hz, 1H), 2.66 (s, 1H), 2.38 (s, 3H); ^{13}C NMR (125 MHz, $CDCl_3$) δ 144.5, 143.0, 141.6, 134.9, 134.4, 131.4, 130.4, 129.7, 128.3, 128.2, 127.8(2), 127.8(0), 127.5, 127.4, 127.3, 127.1, 126.8, 122.6, 82.2, 72.8, 71.0, 52.4, 31.3, 21.5; IR (neat): 3438(bs), 2930, 2234(s), 1493, 1364, 1168, 1089, 755, 691, 597, 546; HRESIMS Calcd for $[C_{30}H_{27}NNaO_3S]^+$ (M + Na^+) 504.1604, found 504.1604.

N-(2-(hydroxy(phenyl)methyl)phenethyl)-*N*-(phenylethynyl)benzenesulfonamide (1b)

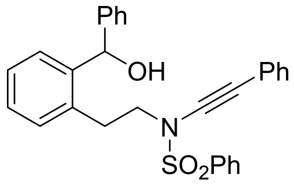

1b

Pale yellow oil. ^{1}H NMR (500 MHz, $CDCl_3$) δ 7.85 (d, *J* = 7.5 Hz, 2H), 7.65 – 7.59 (m, 1H), 7.53 – 7.48 (m, 2H), 7.46 (dd, *J* = 7.0 Hz, *J* = 1.5 Hz, 1H), 7.38 – 7.34 (m, 2H), 7.32 – 7.28 (m, 5H), 7.27 – 7.18 (m, 6H), 6.07 (s, 1H), 3.58 (ddd, *J* = 16.0 Hz, *J* = 10.0 Hz, *J* = 6.0 Hz, 1H), 3.48 (ddd, *J* = 16.0 Hz, *J* = 10.0 Hz, *J* = 6.5 Hz, 1H), 3.09 (ddd, *J* = 15.5 Hz, *J* = 9.5 Hz, *J* = 6.0 Hz, 1H), 3.00 (ddd, *J* = 16.0 Hz, *J* = 10.0 Hz, *J* = 6.5 Hz, 1H), 2.36 (s, 1H); ^{13}C NMR (125 MHz, $CDCl_3$) δ 143.0, 141.7, 137.5, 135.0, 133.6, 131.6, 130.6, 129.1, 128.5, 128.3, 128.0, 127.9, 127.6, 127.5, 127.3, 126.9, 122.6, 82.0, 73.0, 71.1, 52.6, 31.5; IR (neat): 3440(br), 2955, 2922, 2235(s), 1448, 1364, 1170, 753, 688, 571; HRESIMS Calcd for $[C_{29}H_{25}NNaO_3S]^+$ (M + Na^+) 490.1447, found 490.1449.

4-bromo-*N*-(2-(hydroxy(phenyl)methyl)phenethyl)-*N*-(phenylethynyl)benzenesulfonamide (1c)

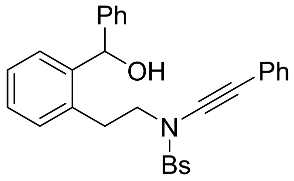

1c

Yellow oil. ^{1}H NMR (400 MHz, $CDCl_3$) δ 7.65 (d, *J* = 8.8 Hz, 2H), 7.59 (d, *J* = 8.8 Hz, 2H), 7.43 (dd, *J* = 7.6 Hz, *J* = 1.6 Hz, 1H), 7.40 – 7.34 (m, 2H), 7.33 – 7.12 (m, 11H), 6.03 (s, 1H), 3.53 (ddd, *J* = 15.6 Hz, *J* = 9.6 Hz, *J* = 6.4 Hz, 1H), 3.44 (ddd, *J* = 15.6 Hz, *J* = 9.2 Hz, *J* = 6.4 Hz, 1H), 3.12 – 2.91 (m, 2H), 2.56 (s, 1H); ^{13}C NMR (100 MHz, $CDCl_3$) δ 143.0, 141.6, 136.3, 134.8, 132.4, 131.6, 130.6, 128.9, 128.8, 128.4, 128.3, 128.1, 127.9, 127.6, 127.5, 127.2, 126.8, 122.3, 81.6, 73.1, 71.4, 52.6, 31.4; IR (neat): 3455(br), 2923, 2236(s), 1573, 1390, 1367, 1171, 756, 739, 609; HRESIMS Calcd for $[C_{29}H_{24}BrNNaO_3S]^+$ (M + Na^+) 568.0552, found 568.0560.

N-((4-fluorophenyl)ethynyl)-N-(2-(hydroxy(phenyl)methyl)phenethyl)-4-methylbenzenesulfonamide (1d)

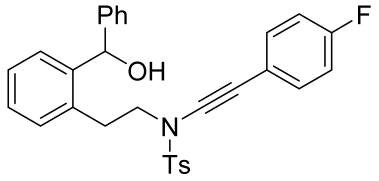

1d

Pale yellow oil. ^{1}H NMR (500 MHz, $CDCl_3$) δ 7.71 (d, J = 8.5 Hz, 2H), 7.43 (dd, J = 7.5 Hz, J = 2.0 Hz, 1H), 7.35 – 7.31 (m, 2H), 7.30 – 7.20 (m, 9H), 7.18 (dd, J = 7.0 Hz, J = 1.5 Hz, 1H), 7.02 – 6.95 (m, 2H), 6.04 (d, J = 4.0 Hz, 1H), 3.54 (ddd, J = 16.0 Hz, J = 10.0 Hz, J = 6.0 Hz, 1H), 3.46 (ddd, J = 15.5 Hz, J = 9.5 Hz, J = 6.0 Hz, 1H), 3.06 (ddd, J = 15.5 Hz, J = 10.0 Hz, J = 6.0 Hz, 1H), 2.98 (ddd, J = 15.5 Hz, J = 9.5 Hz, J = 6.0 Hz, 1H), 2.46 (d, J = 4.0 Hz, 1H), 2.42 (s, 3H); ^{13}C NMR (125 MHz, $CDCl_3$) δ 162.3 (d, J = 247.8 Hz), 144.6, 143.1, 141.7, 135.0, 134.6, 133.6 (d, J = 8.3 Hz), 130.5, 129.8, 128.4, 127.9, 127.6, 127.5, 127.2, 126.8, 118.7 (d, J = 3.6 Hz), 115.5 (d, J = 22.0 Hz), 81.9, 73.0, 70.0, 52.5, 31.5, 21.6; IR (neat): 3449(bs), 2954, 2923, 2229(s), 1460, 1376, 1167, 699, 658, 543; HRESIMS Calcd for $[C_{30}H_{26}FNNaO_3S]^+$ (M + Na^+) 522.1510, found 522.1511.

N-((4-chlorophenyl)ethynyl)-N-(2-(hydroxy(phenyl)methyl)phenethyl)-4-methylbenzenesulfonamide (1e)

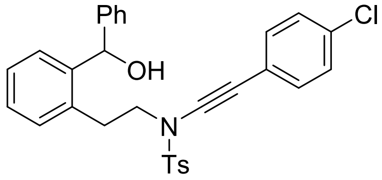

1e

Pale yellow oil. ^{1}H NMR (400 MHz, $CDCl_3$) δ 7.69 (d, J = 8.4 Hz, 2H), 7.43 – 7.37 (m, 1H), 7.31 – 7.11 (m, 14H), 5.99 (s, 1H), 3.51 (ddd, J = 15.6 Hz, J = 9.6 Hz, J = 6.0 Hz, 1H), 3.42 (ddd, J = 15.6 Hz, J = 9.2 Hz, J = 6.0 Hz, 1H), 3.08 – 2.90 (m, 2H), 2.79 (s, 1H), 2.38 (s, 3H); ^{13}C NMR (100 MHz, $CDCl_3$) δ 144.6, 143.0, 141.6, 134.8, 134.3, 133.7, 132.5, 130.4, 129.7, 128.5, 128.3, 127.8, 127.5, 127.4, 127.3, 127.0, 126.7, 121.1, 83.1, 72.8, 70.0, 52.3, 31.3, 21.5; IR (neat): 3536(bs), 3061, 3026, 2235(s), 1597, 1492, 1448,

1363, 1168, 755, 547; HRESIMS Calcd for $[C_{30}H_{26}ClNNaO_3S]^+$ (M + Na^+) 538.1214, found 538.1220.

N-((4-bromophenyl)ethynyl)-N-(2-(hydroxy(phenyl)methyl)phenethyl)-4-methylbenzenesulfonamide (1f)

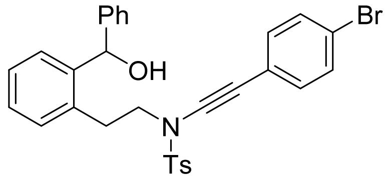

1f

Pale yellow oil. ^{1}H NMR (400 MHz, $CDCl_3$) δ 7.71 (d, J = 8.4 Hz, 2H), 7.47 – 7.39 (m, 3H), 7.31 – 7.16 (m, 12H), 6.05 (s, 1H), 3.55 (ddd, J = 16.0 Hz, J = 9.6 Hz, J = 6.0 Hz, 1H), 3.47 (ddd, J = 15.6 Hz, J = 9.2 Hz, J = 6.4 Hz, 1H), 3.07 (ddd, J = 15.2 Hz, J = 9.6 Hz, J = 6.4 Hz, 1H), 2.98 (ddd, J = 16.0 Hz, J = 9.6 Hz, J = 6.0 Hz, 1H), 2.42 (s, 3H), 2.40 (s, 1H); ^{13}C NMR (100 MHz, $CDCl_3$) δ 144.7, 143.1, 141.7, 135.0, 134.6, 132.8, 131.5, 130.5, 129.8, 128.5, 128.0, 127.5(9), 127.5(7), 127.5(3), 127.2, 126.8, 122.0, 121.7, 83.4, 73.0, 70.2, 52.5, 31.5, 21.6; IR (neat): 3438(bs), 3063, 2924, 2236(s), 1599, 1488, 1364, 1168, 1088, 701, 657, 562; HRESIMS Calcd for $[C_{30}H_{26}BrNNaO_3S]^+$ (M + Na^+) 582.0709, found 582.0709.

N-((4-cyanophenyl)ethynyl)-N-(2-(hydroxy(phenyl)methyl)phenethyl)-4-methylbenzenesulfonamide (1g)

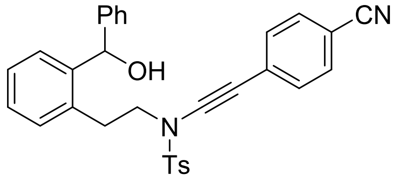

1g

Pale yellow oil. ^{1}H NMR (400 MHz, $CDCl_3$) δ 7.71 (d, J = 8.4 Hz, 2H), 7.52 (d, J = 8.4 Hz, 2H), 7.44 – 7.39 (m, 1H), 7.36 (d, J = 8.4 Hz, 2H), 7.31 – 7.19 (m, 9H), 7.18 – 7.13 (m, 1H), 6.02 (s, 1H), 3.61 – 3.42 (m, 2H), 3.10 – 2.93 (m, 2H), 2.78 (s, 1H), 2.41 (s, 3H); ^{13}C NMR (100 MHz, $CDCl_3$) δ 145.0, 143.0, 141.6, 134.7, 134.2, 131.9, 130.9, 130.4, 129.8, 128.3, 127.9, 127.8, 127.7, 127.4, 127.3, 127.2, 126.7, 118.5, 110.4, 87.0, 72.9,

70.6, 52.3, 31.4, 21.5; IR (neat): 3464(bs), 3063, 2224(s), 1450, 1367, 1169, 1089, 700, 660, 543; HRESIMS Calcd for $[C_{31}H_{26}N_2NaO_3S]^+$ (M + Na^+) 529.1556, found 529.1554.

N-(2-(hydroxy(phenyl)methyl)phenethyl)-4-methyl-N-((4-(trifluoromethyl)phenyl)ethynyl)benzenesulfonamide (1h)

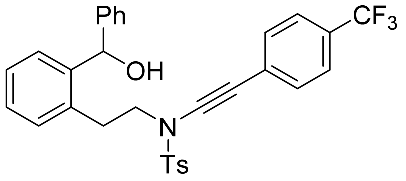

1h

Pale yellow oil. ^{1}H NMR (500 MHz, $CDCl_3$) δ 7.71 (d, J = 8.5 Hz, 2H), 7.52 (d, J = 8.0 Hz, 2H), 7.45 – 7.37 (m, 3H), 7.29 – 7.19 (m, 9H), 7.18 – 7.15 (m, 1H), 6.02 (d, J = 3.0 Hz, 1H), 3.55 (ddd, J = 16.0 Hz, J = 10.0 Hz, J = 6.5 Hz, 1H), 3.47 (ddd, J = 15.5 Hz, J = 9.5 Hz, J = 6.0 Hz, 1H), 3.06 (ddd, J = 15.5 Hz, J = 9.5 Hz, J = 6.0 Hz, 1H), 2.99 (ddd, J = 16.0 Hz, J = 10.0 Hz, J = 6.5 Hz, 1H), 2.67 (d, J = 3.5 Hz, 1H), 2.40 (s, 3H); ^{13}C NMR (125 MHz, $CDCl_3$) δ 144.9, 143.0, 141.7, 134.9, 134.4, 131.0, 130.5, 129.8, 128.4, 127.9, 127.6, 127.5, 127.4, 127.2, 126.8, 126.7, 125.1 (q, J = 3.5 Hz), 125.0, 122.8, 84.9, 73.0, 70.4, 52.4, 31.5, 21.5; IR (neat): 3434(bs), 2927, 2234(s), 1598, 1367, 1322, 1065, 742, 601, 563; HRESIMS Calcd for $[C_{31}H_{26}F_3NNaO_3S]^+$ (M + Na^+) 572.1478, found 572.1481.

N-(2-(hydroxy(phenyl)methyl)phenethyl)-4-methyl-N-(p-tolylethynyl)benzenesulfonamide (1i)

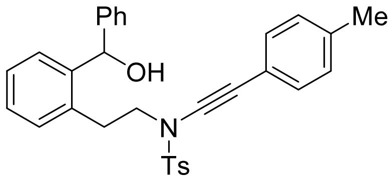

1i

Pale yellow oil. ^{1}H NMR (400 MHz, $CDCl_3$) δ 7.71 (d, J = 8.4 Hz, 2H), 7.46 – 7.42 (m, 1H), 7.30 – 7.19 (m, 11H), 7.19 – 7.15 (m, 1H), 7.10 (d, J = 8.0 Hz, 2H), 6.04 (s, 1H), 3.53 (ddd, J = 16.0 Hz, J = 10.0 Hz, J = 6.0 Hz, 1H), 3.43 (ddd, J = 15.6 Hz, J = 9.6 Hz, J = 6.4 Hz, 1H), 3.06 (ddd, J = 15.6 Hz, J = 9.6 Hz, J = 6.0 Hz, 1H), 2.97 (ddd, J = 16.0

Hz, J = 10.0 Hz, J = 6.4 Hz, 1H), 2.47 (s, 1H), 2.40 (s, 3H), 2.33 (s, 3H); ^{13}C NMR (100 MHz, $CDCl_3$) δ 144.5, 143.1, 141.7, 138.1, 135.0, 134.6, 131.6, 130.5, 129.7, 129.0, 128.4, 127.9, 127.6, 127.4, 127.1, 126.9, 119.5, 81.5, 72.9, 71.0, 52.5, 31.4, 21.5, 21.4; IR (neat): 3438(bs), 2954, 2923, 2235(s), 1597, 1364, 1167, 1089, 755, 691, 587; HRESIMS Calcd for $[C_{31}H_{29}NNaO_3S]^+$ (M + Na^+) 518.1760, found 518.1758.

***N*-(2-(hydroxy(phenyl)methyl)phenethyl)-*N*-((4-methoxyphenyl)ethynyl)-4-methylbenzenesulfonamide (1j)**

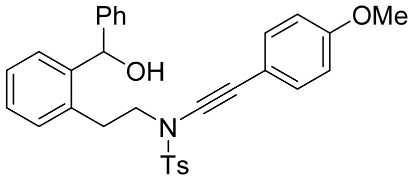

1j

Pale yellow oil. ^{1}H NMR (400 MHz, $CDCl_3$) δ 7.72 (d, J = 8.4 Hz, 2H), 7.49 – 7.43 (m, 1H), 7.34 – 7.18 (m, 12H), 6.84 (d, J = 8.8 Hz, 2H), 6.07 (s, 1H), 3.81 (s, 3H), 3.55 (ddd, J = 15.6 Hz, J = 9.6 Hz, J = 6.0 Hz, 1H), 3.45 (ddd, J = 15.6 Hz, J = 9.6 Hz, J = 6.4 Hz, 1H), 3.08 (ddd, J = 15.6 Hz, J = 9.6 Hz, J = 6.0 Hz, 1H), 2.98 (ddd, J = 16.0 Hz, J = 10.0 Hz, J = 6.0 Hz, 1H), 2.43 (s, 3H), 2.34 (s, 1H); ^{13}C NMR (100 MHz, $CDCl_3$) δ 159.6, 144.5, 143.1, 141.7, 135.1, 134.7, 133.6, 130.6, 129.7, 128.5, 127.9, 127.6, 127.5, 127.4, 127.2, 126.9, 114.6, 114.0, 80.8, 72.9, 70.7, 55.3, 52.7, 31.5, 21.6; IR (neat): 3421(bs), 2935, 2848, 2242(s), 1511, 1361, 1248, 1167, 1030, 666, 545; HRESIMS Calcd for $[C_{31}H_{29}NNaO_4S]^+$ (M + Na^+) 534.1710, found 534.1710.

***N*-((3-bromophenyl)ethynyl)-*N*-(2-(hydroxy(phenyl)methyl)phenethyl)-4-methylbenzenesulfonamide (1k)**

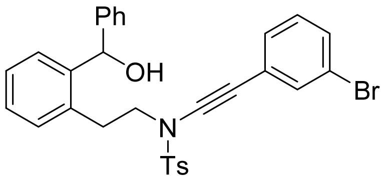

1k

Pale yellow oil. ^{1}H NMR (400 MHz, $CDCl_3$) δ 7.71 (d, J = 8.4 Hz, 2H), 7.48 – 7.46 (m, 1H), 7.45 – 7.42 (m, 1H), 7.41 – 7.38 (m, 1H), 7.31 – 7.21 (m, 10H), 7.19 – 7.12 (m, 2H),

6.04 (d, J = 4.0 Hz, 1H), 3.55 (ddd, J = 16.0 Hz, J = 9.6 Hz, J = 6.0 Hz, 1H), 3.46 (ddd, J = 15.6, 9.2, 6.0 Hz, 1H), 3.05 (ddd, J = 15.6 Hz, J = 9.2 Hz, J = 6.4 Hz, 1H), 2.97 (ddd, J = 16.0, 10.0, 6.4 Hz, 1H), 2.45 (d, J = 4.0 Hz, 1H), 2.42 (s, 3H); ^{13}C NMR (100 MHz, $CDCl_3$) δ 144.8, 143.0, 141.6, 134.9, 134.5, 133.9, 130.9, 130.5, 129.8, 129.7, 129.6, 128.5, 128.0, 127.6, 127.5, 127.2, 126.8, 124.8, 122.0, 83.6, 73.0, 69.9, 52.5, 31.5, 21.6; IR (neat): 3434(bs), 2922, 2855, 2234(s), 1449, 1367, 1167, 1088, 664, 544; HRESIMS Calcd for $[C_{30}H_{26}BrNNaO_3S]^+$ (M + Na^+) 582.0709, found 582.0708.

_N_-(2-(hydroxy(phenyl)methyl)phenethyl)-4-methyl-_N_-(_m_-tolylethynyl)benzenesulfonamide (1l)

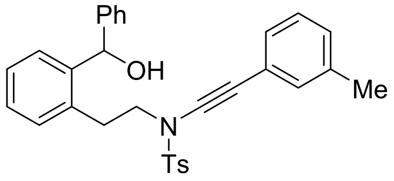

1l

Pale yellow oil. ^{1}H NMR (400 MHz, $CDCl_3$) δ 7.72 (d, J = 8.4 Hz, 2H), 7.47 – 7.41 (m, 1H), 7.31 – 7.16 (m, 13H), 7.13 – 7.07 (m, 1H), 6.06 (s, 1H), 3.55 (ddd, J = 15.6 Hz, J = 9.6 Hz, J = 5.6 Hz, 1H), 3.46 (ddd, J = 15.6 Hz, J = 9.6 Hz, J = 6.0 Hz, 1H), 3.08 (ddd, J = 15.2 Hz, J = 9.2 Hz, J = 6.0 Hz, 1H), 2.99 (ddd, J = 16.0 Hz, J = 10.0 Hz, J = 6.4 Hz, 1H), 2.41 (s, 3H), 2.31 (s, 3H); ^{13}C NMR (100 MHz, $CDCl_3$) δ 144.5, 143.1, 141.7, 137.9, 135.1, 134.6, 132.1, 130.5, 129.7, 128.8, 128.5, 128.4, 128.2, 127.9, 127.6, 127.5, 127.2, 126.9, 122.5, 81.9, 72.9, 71.2, 52.6, 31.4, 21.6, 21.2; IR (neat): 3466(bs), 2925, 2850, 2237(s), 1496, 1447, 1359, 1167, 654, 591; HRESIMS Calcd for $[C_{31}H_{29}NNaO_3S]^+$ (M + Na^+) 518.1760, found 518.1762.

_N_-(2-(hydroxy(phenyl)methyl)phenethyl)-4-methyl-_N_-(naphthalen-2-ylethynyl)benzenesulfonamide (1m)

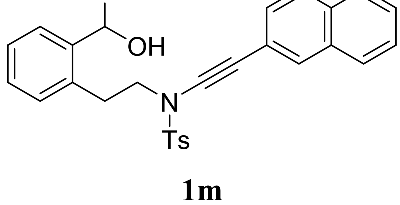

1m

Pale yellow oil. ^{1}H NMR (500 MHz, $CDCl_3$) δ 7.85 (s, 1H), 7.78 – 7.69 (m, 5H), 7.46 – 7.37 (m, 4H), 7.28 – 7.14 (m, 10H), 6.03 (d, J = 3.5 Hz, 1H), 3.56 (ddd, J = 15.5 Hz, J = 10.0 Hz, J = 6.0 Hz, 1H), 3.47 (ddd, J = 16.0 Hz, J = 9.5 Hz, J = 6.5 Hz, 1H), 3.08 (ddd, J = 15.5 Hz, J = 9.5 Hz, J = 6.0 Hz, 1H), 3.00 (ddd, J = 16.0 Hz, J = 9.5 Hz, J = 6.0 Hz, 1H), 2.69 (d, J = 4.0 Hz, 1H), 2.35 (s, 3H); ^{13}C NMR (125 MHz, $CDCl_3$) δ 144.6, 143.0, 141.6, 134.9, 134.4, 132.9, 132.4, 130.9, 130.4, 129.7, 128.3, 128.2, 127.8(4), 127.8(0), 127.6, 127.5, 127.4, 127.3, 127.1, 126.8, 126.4, 126.3, 119.9, 82.5, 72.8, 71.5, 52.4, 31.3, 21.5; IR (neat): 3518(bs), 2954, 2924, 2234(s), 1596, 1364, 1168, 1089, 756, 702, 696; HRESIMS Calcd for $[C_{34}H_{29}NNaO_3S]^+$ (M + Na^+) 554.1760, found 554.1760.

_N_-(2-(hydroxy(phenyl)methyl)phenethyl)-4-methyl-_N_-(thiophen-2-ylethynyl)benzenesulfonamide (1n)

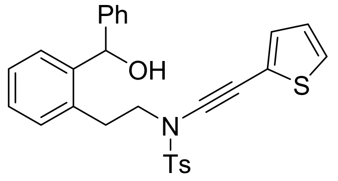

1n

Pale yellow oil. ^{1}H NMR (500 MHz, $CDCl_3$) δ 7.68 (d, J = 8.0 Hz, 2H), 7.43 (dd, J = 7.5 Hz, J = 1.5 Hz, 1H), 7.28 – 7.16 (m, 11H), 7.13 (dd, J = 7.0 Hz, J = 1.5 Hz, 1H), 6.95 (dd, J = 5.0 Hz, J = 3.5 Hz, 1H), 5.98 (s, 1H), 3.49 (ddd, J = 16.0 Hz, J = 10.0 Hz, J = 6.0 Hz, 1H), 3.38 (ddd, J = 16.0 Hz, J = 10.0 Hz, J = 6.5 Hz, 1H), 3.01 (ddd, J = 15.5 Hz, J = 10.0 Hz, J = 6.0 Hz, 1H), 2.92 (ddd, J = 16.5 Hz, J = 10.0 Hz, J = 6.5 Hz, 1H), 2.62 (s, 1H), 2.40 (s, 3H); ^{13}C NMR (125 MHz, $CDCl_3$) δ 144.6, 143.0, 141.6, 134.8, 134.4, 133.1, 130.4, 129.7, 128.3, 127.8, 127.7, 127.5, 127.3(8), 127.3(6), 127.1, 127.0, 126.9, 122.7, 85.8, 72.9, 64.4, 52.5, 31.3, 21.5; IR (neat): 3451(bs), 2920, 2848, 2229(s), 1478, 1359, 1165, 1090, 671, 547; HRESIMS Calcd for $[C_{28}H_{25}NNaO_3S_2]^+$ (M + Na^+) 510.1168, found 510.1172.

(_E_)-_N_-(2-(hydroxy(phenyl)methyl)phenethyl)-4-methyl-_N_-(4-phenylbut-3-en-1-yn-1-yl)benzenesulfonamide (1o)

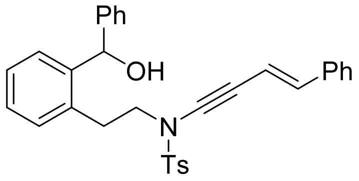

1o

Pale yellow oil. ^{1}H NMR (500 MHz, $CDCl_3$) δ 7.69 (d, *J* = 8.5 Hz, 2H), 7.44 – 7.39 (m, 1H), 7.33 (d, *J* = 7.5 Hz, 2H), 7.31 – 7.18 (m, 12H), 7.16 – 7.13 (m, 1H), 6.82 (d, *J* = 16.0 Hz, 1H), 6.24 (d, *J* = 16.0 Hz, 1H), 6.00 (d, *J* = 2.5 Hz, 1H), 3.49 (ddd, *J* = 16.0 Hz, *J* = 10.0 Hz, *J* = 6.0 Hz, 1H), 3.39 (ddd, *J* = 15.5 Hz, *J* = 9.5 Hz, *J* = 6.0 Hz, 1H), 3.01 (ddd, *J* = 15.5 Hz, *J* = 10.0 Hz, *J* = 6.0 Hz, 1H), 2.93 (ddd, *J* = 16.0 Hz, *J* = 10.0 Hz, *J* = 6.5 Hz, 1H), 2.73 (d, *J* = 3.0 Hz, 1H), 2.37 (s, 3H); ^{13}C NMR (125 MHz, $CDCl_3$) δ 144.5, 143.1, 141.6, 139.7, 136.2, 134.9, 134.4, 130.4, 129.7, 128.6, 128.3, 128.2, 127.8, 127.4(3), 127.3(9), 127.3(3), 127.0, 126.8, 125.9, 107.3, 84.2, 72.8, 70.7, 52.4, 31.3, 21.5; IR (neat): 3534(bs), 3027, 2923, 2217(s), 1596, 1448, 1361, 1166, 1088, 749, 692, 542; HRESIMS Calcd for $[C_{32}H_{29}NNaO_3S]^+$ (M + Na^+) 530.1760, found 530.1761.

***N*-(2-(hydroxy(phenyl)methyl)-4-methylphenethyl)-4-methyl-*N*-(phenylethynyl)benzenesulfonamide (1p)**

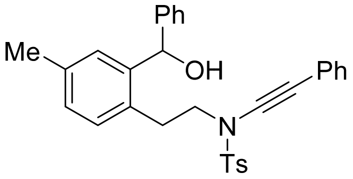

1p

Pale yellow oil. ^{1}H NMR (500 MHz, $CDCl_3$) δ 7.71 (d, *J* = 8.0 Hz, 2H), 7.37 – 7.33 (m, 2H), 7.28 – 7.17 (m, 11H), 7.05 (d, *J* = 7.5 Hz, 1H), 7.01 (dd, *J* = 8.0 Hz, *J* = 1.5 Hz, 1H), 5.98 (d, *J* = 3.5 Hz, 1H), 3.49 (ddd, *J* = 16.0 Hz, *J* = 10.0 Hz, *J* = 6.0 Hz, 1H), 3.40 (ddd, *J* = 16.0 Hz, *J* = 9.5 Hz, *J* = 6.0 Hz, 1H), 3.00 (ddd, *J* = 15.0 Hz, *J* = 9.5 Hz, *J* = 6.0 Hz, 1H), 2.92 (ddd, *J* = 16.0 Hz, *J* = 10.0 Hz, *J* = 6.5 Hz, 1H), 2.59 (d, *J* = 4.0 Hz, 1H), 2.38 (s, 3H), 2.28 (s, 3H); ^{13}C NMR (125 MHz, $CDCl_3$) δ 144.5, 143.1, 141.4, 136.6, 134.5, 131.8, 131.4, 130.4, 129.6, 128.5, 128.3, 128.2, 128.0, 127.8, 127.5, 127.3, 126.8, 122.6, 82.2, 72.8, 71.0, 52.5, 31.0, 21.5, 21.1; IR (neat): 3553(bs), 2923, 2877, 2234(s), 1597,

1493, 1363, 1090, 754, 691, 546; HRESIMS Calcd for $[C_{31}H_{29}NNaO_3S]^+$ (M + Na^+) 518.1760, found 518.1762.

Compounds **1q-1s, 1x** were prepared according to the following procedures.[1,3,4]

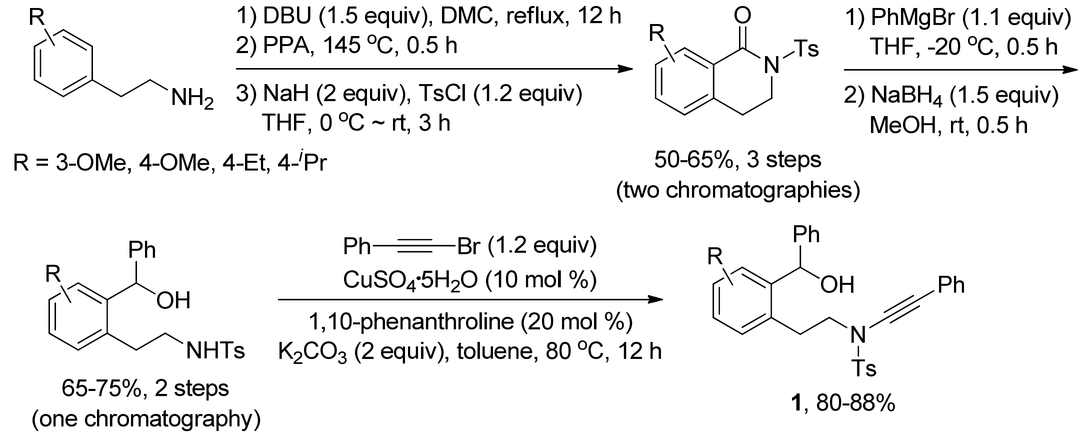

Supplementary Figure 131. Procedures for the preparation of ynamides **1q-1s, 1x**.

N-(4-ethyl-2-(hydroxy(phenyl)methyl)phenethyl)-4-methyl-*N*-(phenylethynyl)benzenesulfonamide (1q)

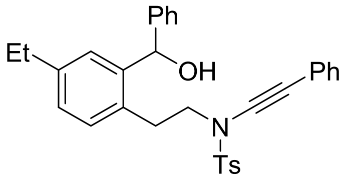

1q

Pale yellow oil. ^{1}H NMR (400 MHz, $CDCl_3$) δ 7.72 (d, *J* = 8.4 Hz, 2H), 7.38 – 7.33 (m, 2H), 7.31 – 7.16 (m, 11H), 7.11 – 7.04 (m, 2H), 6.01 (d, *J* = 4.0 Hz, 1H), 3.50 (ddd, *J* = 16.0 Hz, *J* = 10.0 Hz, *J* = 6.0 Hz, 1H), 3.41 (ddd, *J* = 15.6 Hz, *J* = 9.6 Hz, *J* = 6.0 Hz, 1H), 3.01 (ddd, *J* = 15.6 Hz, *J* = 10.0 Hz, *J* = 6.4 Hz, 1H), 2.93 (ddd, *J* = 16.4 Hz, *J* = 10.0 Hz, *J* = 6.4 Hz, 1H), 2.60 (q, *J* = 7.6 Hz, 2H), 2.48 (d, *J* = 3.6 Hz, 1H), 2.40 (s, 3H), 1.19 (t, *J* = 7.6 Hz, 3H); ^{13}C NMR (100 MHz, $CDCl_3$) δ 144.5, 143.1, 143.0, 141.4, 134.5, 132.0, 131.4, 130.5, 129.7, 128.4, 128.2, 127.8, 127.5, 127.4, 127.3, 126.9, 122.7, 82.3, 73.0, 71.0, 52.5, 31.0, 28.5, 21.5, 15.4; IR (neat): 3540(bs), 2963, 2928, 2235(s), 1493, 1363, 1167, 1090, 755, 691, 584; HRESIMS Calcd for $[C_{32}H_{31}NNaO_3S]^+$ (M + Na^+) 532.1917, found 532.1915.

_N_-(2-(hydroxy(phenyl)methyl)-4-isopropylphenethyl)-4-methyl-_N_-(phenylethynyl)benzenesulfonamide (1r)

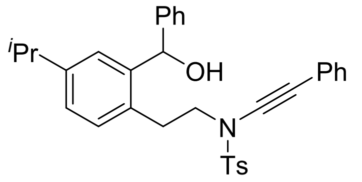

1r

Yellow oil. ^{1}H NMR (400 MHz, $CDCl_3$) δ 7.71 (d, J = 8.4 Hz, 2H), 7.38 – 7.32 (m, 3H), 7.30 – 7.14 (m, 10H), 7.11 – 7.06 (m, 2H), 5.99 (d, J = 3.6 Hz, 1H), 3.47 (ddd, J = 16.0 Hz, J = 10.0 Hz, J = 6.0 Hz, 1H), 3.38 (ddd, J = 16.0 Hz, J = 10.0 Hz, J = 6.4 Hz, 1H), 3.04 – 2.88 (m, 2H), 2.88 – 2.79 (m, 1H), 2.62 (d, J = 3.6 Hz, 1H), 2.38 (s, 3H), 1.20 (d, J = 7.2 Hz, 6H); ^{13}C NMR (100 MHz, $CDCl_3$) δ 147.6, 144.5, 143.1, 141.3, 134.3, 132.1, 131.3, 130.4, 129.6, 128.3, 128.2, 127.8, 127.5, 127.3, 126.8, 125.6, 125.5, 122.6, 82.2, 73.1, 70.9, 52.4, 33.7, 30.9, 23.9, 23.8, 21.5; IR (neat): 3438(bs), 2959, 2925, 2235(s), 1493, 1364, 1168, 756, 691, 586; HRESIMS Calcd for $[C_{33}H_{33}NNaO_3S]^+$ (M + Na^+) 546.2073, found 546.2072.

_N_-(2-(hydroxy(phenyl)methyl)-4-methoxyphenethyl)-4-methyl-_N_-(phenylethynyl)benzenesulfonamide (1s)

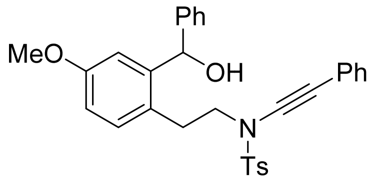

1s

Yellow oil. ^{1}H NMR (400 MHz, $CDCl_3$) δ 7.69 (d, J = 8.4 Hz, 2H), 7.38 – 7.32 (m, 2H), 7.28 – 7.14 (m, 10H), 7.08 – 7.01 (m, 2H), 6.72 (dd, J = 8.4 Hz, J = 2.8 Hz, 1H), 5.95 (s, 1H), 3.68 (s, 3H), 3.46 (ddd, J = 16.0 Hz, J = 10.0 Hz, J = 6.4 Hz, 1H), 3.36 (ddd, J = 15.6 Hz, J = 9.6 Hz, J = 6.4 Hz, 1H), 3.01 – 2.76 (m, 3H), 2.36 (s, 3H); ^{13}C NMR (100 MHz, $CDCl_3$) δ 158.5, 144.5, 142.9, 142.8, 134.3, 131.4, 131.3, 129.6, 128.3, 128.1, 127.7, 127.4, 127.3, 126.8, 126.7, 122.6, 113.0, 112.8, 82.2, 72.7, 71.0, 55.0, 52.4, 30.4,

21.4; IR (neat): 3533(bs), 2926, 2234(s), 1495, 1364, 1167, 1089, 1043, 756, 586; HRESIMS Calcd for $[C_{31}H_{29}NNaO_4S]^+$ (M + Na^+) 534.1710, found 534.1714.

_N_-(2-(3-(hydroxy(phenyl)methyl)-[1,1'-biphenyl]-4-yl)ethyl)-4-methyl-_N_-(phenylethynyl)benzenesulfonamide (1t)

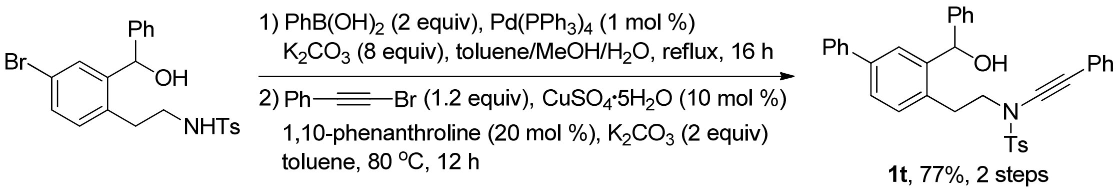

Supplementary Figure 132. Procedures for the preparation of ynamide **1t**.

Compound **1t** was prepared according to the above known procedures.[1,3,5,6] Pale yellow oil. ^{1}H NMR (400 MHz, $CDCl_3$) δ 7.73 (d, J = 2.0 Hz, 1H), 7.71 (d, J = 8.4 Hz, 2H), 7.57 – 7.52 (m, 2H), 7.43 (dd, J = 7.6 Hz, J = 2.0 Hz, 1H), 7.41 – 7.33 (m, 4H), 7.32 – 7.17 (m, 12H), 6.07 (d, J = 3.2 Hz, 1H), 3.55 (ddd, J = 15.6 Hz, J = 9.6 Hz, J = 6.4 Hz, 1H), 3.46 (ddd, J = 15.6 Hz, J = 9.2 Hz, J = 6.4 Hz, 1H), 3.12 – 2.94 (m, 2H), 2.62 (d, J = 3.2 Hz, 1H), 2.35 (s, 3H); ^{13}C NMR (100 MHz, $CDCl_3$) δ 144.6, 142.9, 142.1, 140.5, 139.9, 134.4, 134.0, 131.4, 131.0, 129.7, 128.7, 128.4, 128.2, 127.9, 127.5(1), 127.4(9), 127.2, 126.9(2), 126.9(0), 126.4, 126.0, 122.6, 82.2, 73.0, 71.1, 52.4, 31.1, 21.5; IR (neat): 3444(bs), 2955, 2922, 2234(s), 1455, 1364, 1167, 1018, 757, 698, 546; HRESIMS Calcd for $[C_{36}H_{31}NNaO_3S]^+$ (M + Na^+) 580.1917, found 580.1917.

_N_-(4-chloro-2-(hydroxy(phenyl)methyl)phenethyl)-4-methyl-_N_-(phenylethynyl)benzenesulfonamide (1u)

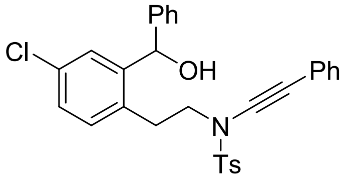

1u

Pale yellow oil. ^{1}H NMR (400 MHz, $CDCl_3$) δ 7.68 (d, J = 8.0 Hz, 2H), 7.49 (d, J = 2.0 Hz, 1H), 7.38 – 7.32 (m, 2H), 7.31 – 7.20 (m, 10H), 7.14 (dd, J = 8.0 Hz, J = 2.0 Hz, 1H), 7.07 (d, J = 8.4 Hz, 1H), 5.97 (d, J = 2.4 Hz, 1H), 3.49 (ddd, J = 15.2 Hz, J = 9.2 Hz, J = 6.4 Hz, 1H), 3.39 (ddd, J = 14.8 Hz, J = 8.8 Hz, J = 6.4 Hz, 1H), 3.05 – 2.94 (m, 1H),

2.94 – 2.83 (m, 1H), 2.71 (d, $J = 2.8$ Hz, 1H), 2.41 (s, 3H); ^{13}C NMR (100 MHz, $CDCl_3$) δ 144.7, 143.5, 142.3, 134.3, 133.3, 133.0, 131.8, 131.5, 129.7, 128.6, 128.3, 128.0, 127.8, 127.4, 127.3, 126.9, 122.5, 82.0, 72.5, 71.2, 52.1, 30.7, 21.5; IR (neat): 3438(bs), 2955, 2923, 2234(s), 1460, 1364, 1167, 1090, 756, 669; HRESIMS Calcd for $[C_{30}H_{26}ClNNaO_3S]^+$ (M + Na^+) 538.1214, found 538.1214.

_N_-(4-bromo-2-(hydroxy(phenyl)methyl)phenethyl)-4-methyl-_N_-(phenylethynyl)benzenesulfonamide (1v)

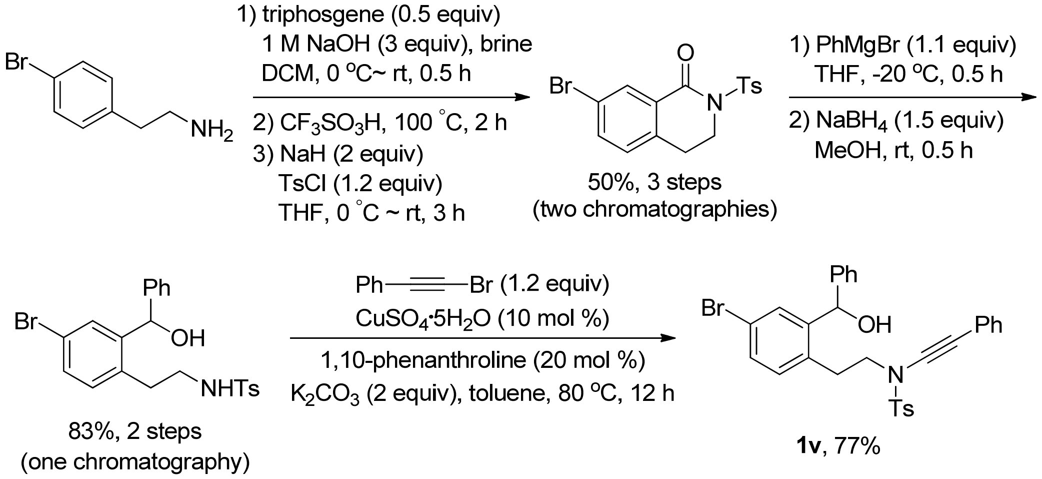

Supplementary Figure 133. Procedures for the preparation of ynamide **1v**.

Compound **1v** was prepared according to the above known procedures.[1,3,6] Yellow oil. ^{1}H NMR (400 MHz, $CDCl_3$) δ 7.67 (d, $J = 8.4$ Hz, 2H), 7.64 (d, $J = 2.0$ Hz, 1H), 7.36 – 7.32 (m, 2H), 7.31 – 7.26 (m, 5H), 7.26 – 7.19 (m, 6H), 7.01 (d, $J = 8.0$ Hz, 1H), 5.96 (d, $J = 3.2$ Hz, 1H), 3.48 (ddd, $J = 15.2$ Hz, $J = 8.8$ Hz, $J = 6.4$ Hz, 1H), 3.39 (ddd, $J = 14.8$ Hz, $J = 8.4$ Hz, $J = 6.0$ Hz, 1H), 3.01 – 2.92 (m, 1H), 2.91 – 2.81 (m, 1H), 2.73 (d, $J = 3.2$ Hz, 1H), 2.40 (s, 3H); ^{13}C NMR (100 MHz, $CDCl_3$) δ 144.7, 143.8, 142.2, 134.3, 133.8, 132.1, 131.4, 130.7, 130.2, 129.7, 128.5, 128.3, 128.0, 127.8, 127.4, 126.9, 122.4, 121.1, 82.0, 72.5, 71.2, 52.0, 30.8, 21.6; IR (neat): 3447(bs), 3030, 2925, 2235(s), 1492, 1453, 1362, 1167, 755, 546; HRESIMS Calcd for $[C_{30}H_{26}BrNNaO_3S]^+$ (M + Na^+) 582.0709, found 582.0713.

_N_-(2-(hydroxy(phenyl)methyl)-5-methylphenethyl)-4-methyl-_N_-(phenylethynyl)benzenesulfonamide (1w)

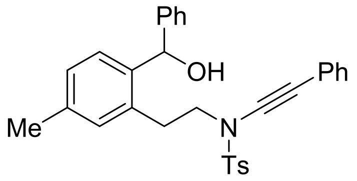

1w

Pale yellow oil. ^{1}H NMR (400 MHz, $CDCl_3$) δ 7.72 (d, *J* = 8.4 Hz, 2H), 7.39 – 7.34 (m, 2H), 7.30 – 7.19 (m, 11H), 7.04 (d, *J* = 8.0 Hz, 1H), 6.98 (s, 1H), 6.00 (d, *J* = 3.2 Hz, 1H), 3.55 (ddd, *J* = 16.0 Hz, *J* = 10.0 Hz, *J* = 6.0 Hz, 1H), 3.45 (ddd, *J* = 16.0 Hz, *J* = 10.0 Hz, *J* = 6.4 Hz, 1H), 3.03 (ddd, *J* = 15.6 Hz, *J* = 9.6 Hz, *J* = 6.0 Hz, 1H), 2.95 (ddd, *J* = 16.4 Hz, *J* = 10.0 Hz, *J* = 6.4 Hz, 1H), 2.46 (d, *J* = 3.6 Hz, 1H), 2.40 (s, 3H), 2.28 (s, 3H); ^{13}C NMR (100 MHz, $CDCl_3$) δ 144.5, 143.3, 138.8, 137.5, 134.8, 134.5, 131.5, 131.2, 129.7, 128.3, 128.2, 127.9, 127.5(3), 127.5(1), 127.3, 126.8, 122.7, 82.2, 72.8, 71.0, 52.5, 31.4, 21.5, 20.9; IR (neat): 3435(bs), 2954, 2924, 2235(s), 1597, 1453, 1364, 1168, 1089, 756, 692, 546; HRESIMS Calcd for $[C_{31}H_{29}NNaO_3S]^+$ (M + Na^+) 518.1760, found 518.1761.

***N*-(2-(hydroxy(phenyl)methyl)-5-methoxyphenethyl)-4-methyl-*N*-(phenylethynyl)benzenesulfonamide (1x)**

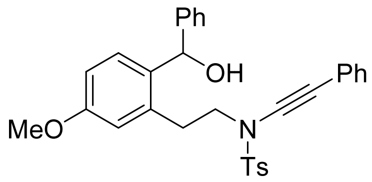

1x

Pale yellow oil. ^{1}H NMR (400 MHz, $CDCl_3$) δ 7.71 (d, *J* = 8.0 Hz, 2H), 7.39 – 7.32 (m, 2H), 7.31 – 7.17 (m, 11H), 6.78 – 6.68 (m, 2H), 5.98 (s, 1H), 3.72 (s, 3H), 3.57 (ddd, *J* = 15.6 Hz, *J* = 9.6 Hz, *J* = 6.4 Hz, 1H), 3.49 (ddd, *J* = 15.6 Hz, *J* = 8.8 Hz, *J* = 6.4 Hz, 1H), 3.10 – 2.92 (m, 2H), 2.55 (s, 1H), 2.40 (s, 3H); ^{13}C NMR (100 MHz, $CDCl_3$) δ 158.9, 144.6, 143.4, 136.6, 134.4, 134.0, 131.4, 129.7, 129.0, 128.3, 128.2, 127.8, 127.5, 127.2, 126.6, 122.6, 115.9, 112.4, 82.2, 72.5, 71.1, 55.1, 52.4, 31.5, 21.5; IR (neat): 3449(bs), 3065, 2928, 2235(s), 1578, 1363, 1170, 1090, 756, 692, 572; HRESIMS Calcd for $[C_{31}H_{29}NNaO_4S]^+$ (M + Na^+) 534.1710, found 534.1710.

***N*-(5-chloro-2-(hydroxy(phenyl)methyl)phenethyl)-4-methyl-*N*-(phenylethynyl)benzenesulfonamide (1y)**

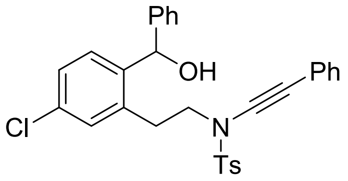

1y

Pale yellow oil. ^{1}H NMR (400 MHz, $CDCl_3$) δ 7.66 (d, *J* = 8.0 Hz, 2H), 7.39 – 7.30 (m, 3H), 7.28 – 7.15 (m, 10H), 7.12 (dd, *J* = 8.4 Hz, *J* = 2.0 Hz, 1H), 7.08 (d, *J* = 2.0 Hz, 1H), 5.92 (d, *J* = 3.6 Hz, 1H), 3.48 (ddd, *J* = 15.6 Hz, *J* = 9.2 Hz, *J* = 6.4 Hz, 1H), 3.38 (ddd, *J* = 15.2 Hz, *J* = 8.8 Hz, *J* = 6.4 Hz, 1H), 3.17 (d, *J* = 4.0 Hz, 1H), 3.01 – 2.82 (m, 2H), 2.36 (s, 3H); ^{13}C NMR (100 MHz, $CDCl_3$) δ 144.6, 142.5, 140.2, 136.7, 134.0, 133.1, 131.4, 130.0, 129.6, 128.8, 128.3, 128.1, 127.9, 127.4, 127.3, 126.9, 126.7, 122.3, 81.8, 72.2, 71.2, 51.8, 30.9, 21.4; IR (neat): 3452(bs), 2954, 2923, 2234(s), 1455, 1364, 1168, 1089, 755, 546; HRESIMS Calcd for $[C_{30}H_{26}ClNNaO_3S]^+$ (M + Na^+) 538.1214, found 538.1211.

***N*-(2-(hydroxy(phenyl)methyl)-4,5-dimethylphenethyl)-4-methyl-*N*-(phenylethynyl)benzenesulfonamide (1z)**

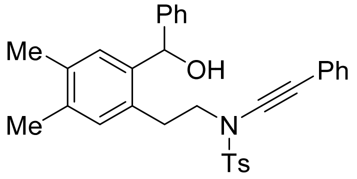

1z

Pale yellow oil. ^{1}H NMR (400 MHz, $CDCl_3$) δ 7.71 (d, *J* = 8.0 Hz, 2H), 7.38 – 7.33 (m, 2H), 7.30 – 7.18 (m, 10H), 7.17 (s, 1H), 6.91 (s, 1H), 5.96 (d, *J* = 1.6 Hz, 1H), 3.56 – 3.35 (m, 2H), 3.04 – 2.86 (m, 2H), 2.55 (d, *J* = 2.8 Hz, 1H), 2.38 (s, 3H), 2.18 (s, 3H), 2.17 (s, 3H); ^{13}C NMR (100 MHz, $CDCl_3$) δ 144.5, 143.4, 139.0, 136.0, 135.3, 134.4, 132.1, 131.8, 131.4, 129.6, 128.6, 128.3, 128.2, 127.8, 127.5, 127.2, 126.7, 122.7, 82.2, 72.6, 71.0, 52.6, 30.9, 21.5, 19.3, 19.2; IR (neat): 3534(bs), 3060, 2922, 2235(s), 1597, 1450, 1363, 1168, 756, 738, 547; HRESIMS Calcd for $[C_{32}H_{31}NNaO_3S]^+$ (M + Na^+) 532.1917, found 532.1915.

N-(2-(6-(hydroxy(phenyl)methyl)benzo[d][1,3]dioxol-5-yl)ethyl)-4-methyl-N-(phenylethynyl)benzenesulfonamide (1aa)

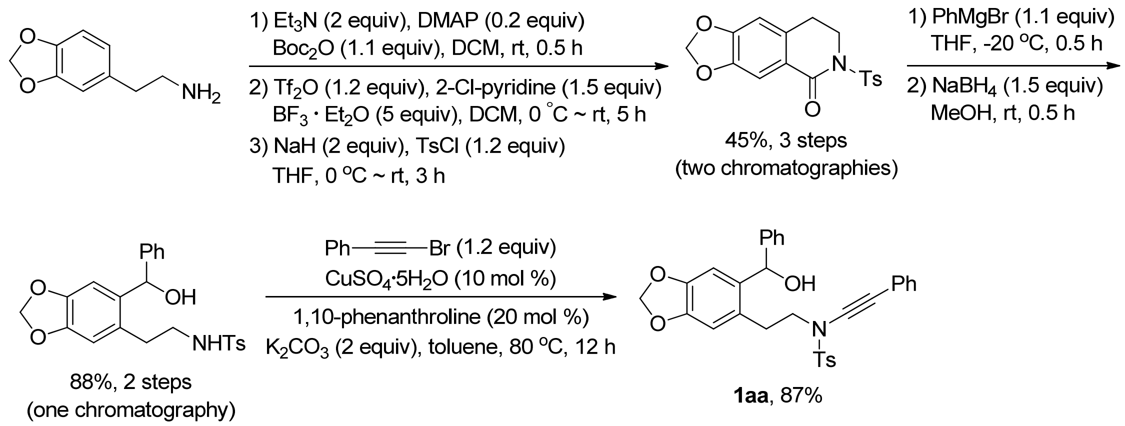

Supplementary Figure 134. Procedures for the preparation of ynamide **1aa**.

Compound **1aa** was prepared according to the above known procedures.[1,3,7] Yellow solid. (mp 170-171 °C). ^{1}H NMR (400 MHz, $CDCl_3$) δ 7.71 (d, J = 8.4 Hz, 2H), 7.39 – 7.32 (m, 2H), 7.31 – 7.17 (m, 10H), 6.87 (s, 1H), 6.61 (s, 1H), 5.96 (s, 1H), 5.82 (dd, J = 4.4 Hz, J = 1.2 Hz, 2H), 3.52 (ddd, J = 15.6 Hz, J = 9.6 Hz, J = 6.4 Hz, 1H), 3.43 (ddd, J = 15.2 Hz, J = 8.8 Hz, J = 6.0 Hz, 1H), 3.00 (ddd, J = 14.8 Hz, J = 9.2 Hz, J = 6.4 Hz, 1H), 2.90 (ddd, J = 15.6 Hz, J = 9.2 Hz, J = 6.4 Hz, 1H), 2.73 (s, 1H), 2.38 (s, 3H); ^{13}C NMR (100 MHz, $CDCl_3$) δ 146.8, 146.6, 144.6, 143.2, 135.5, 134.4, 131.4, 129.6, 128.3, 128.2, 127.8, 127.4, 127.3, 126.6, 122.5, 110.0, 107.8, 100.9, 82.1, 72.1, 71.1, 52.6, 31.3, 21.5; IR (neat): 3445(bs), 2923, 2234(s), 1503, 1484, 1363, 1168, 1040, 676, 576; HRESIMS Calcd for $[C_{31}H_{27}NNaO_5S]^+$ (M + Na^+) 548.1502, found 548.1506.

N-(2-(3-(hydroxy(phenyl)methyl)naphthalen-2-yl)ethyl)-4-methyl-N-(phenylethynyl)benzenesulfonamide (1ab)

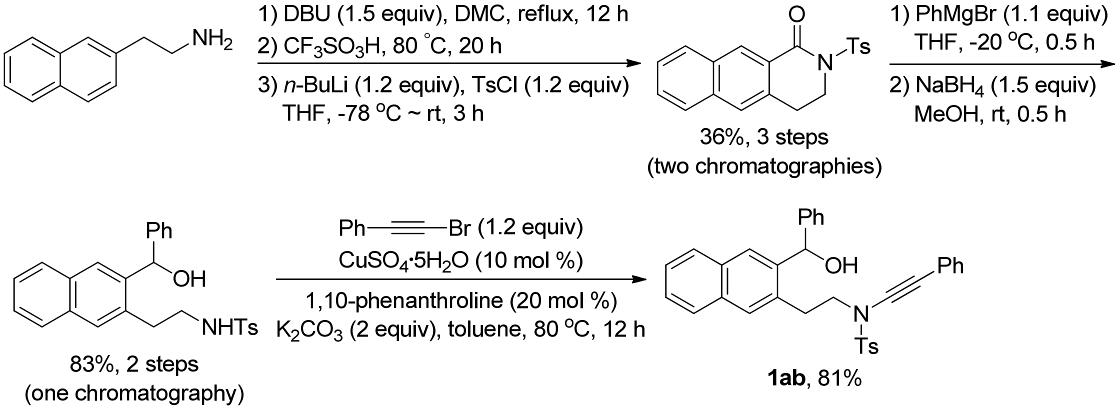

Supplementary Figure 135. Procedures for the preparation of ynamide **1ab**.

Compound **1ab** was prepared according to the above known procedures.[1-3,8] Pale yellow oil. ^{1}H NMR (400 MHz, $CDCl_3$) δ 7.74 (d, *J* = 8.4 Hz, 2H), 7.49 – 7.44 (m, 1H), 7.43 – 6.98 (m, 17H), 6.08 (d, *J* = 2.0 Hz, 1H), 3.57 (ddd, *J* = 15.6 Hz, *J* = 9.6 Hz, *J* = 6.0 Hz, 1H), 3.47 (ddd, *J* = 15.6 Hz, *J* = 9.2 Hz, *J* = 6.0 Hz, 1H), 3.09 (ddd, *J* = 15.2 Hz, *J* = 9.6 Hz, *J* = 6.0 Hz, 1H), 3.00 (ddd, *J* = 16.0 Hz, *J* = 10.0 Hz, *J* = 6.0 Hz, 1H), 2.43 (s, 3H), 2.36 (d, *J* = 3.6 Hz, 1H); ^{13}C NMR (100 MHz, $CDCl_3$) δ 144.6, 143.1, 141.7, 135.1, 134.6, 131.5, 130.6, 129.8, 128.5, 128.3, 128.0, 127.9, 127.6, 127.5(4), 127.5(3), 127.2, 126.9, 122.8, 82.3, 73.0, 71.1, 52.6, 31.5, 21.6; IR (neat): 3440(bs), 2917, 2848, 2234(s), 1493, 1363, 1168, 1089, 692, 597, 546; HRESIMS Calcd for $[C_{34}H_{29}NNaO_3S]^+$ (M + Na^+) 554.1760, found 554.1761.

Compounds **1ac-1ad**, **1af** and **1ah** were prepared according to the following procedures.[1-3]

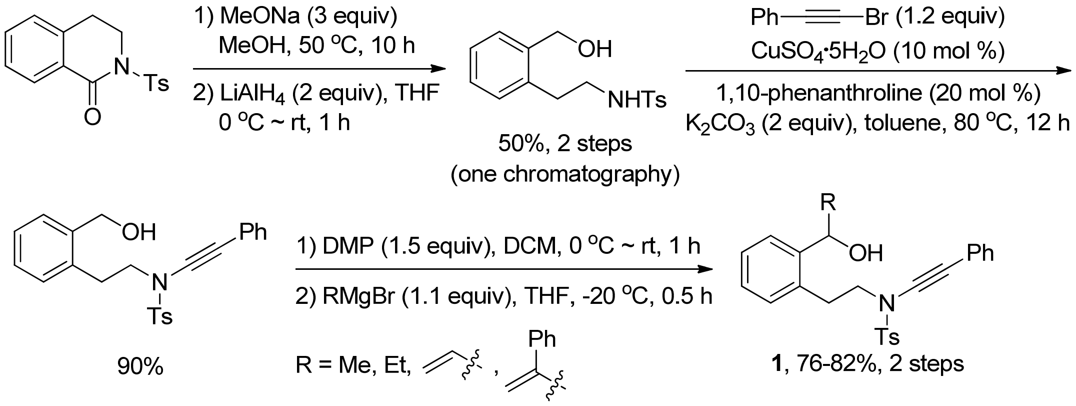

Supplementary Figure 136. Procedures for the preparation of ynamides **1ac-1ad**, **1af** and **1ah**.

***N*-(2-(1-hydroxyethyl)phenethyl)-4-methyl-*N*-(phenylethynyl)benzenesulfonamide (1ac)**

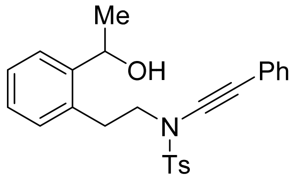

1ac

Pale yellow oil. ^{1}H NMR (500 MHz, $CDCl_3$) δ 7.75 (d, *J* = 8.5 Hz, 2H), 7.50 (dd, *J* = 8.0 Hz, *J* = 1.0 Hz, 1H), 7.40 – 7.34 (m, 2H), 7.30 – 7.22 (m, 6H), 7.19 – 7.11 (m, 2H), 5.13 (q, *J* = 6.5 Hz, 1H), 3.69 – 3.56 (m, 2H), 3.11 – 2.98 (m, 2H), 2.40 (s, 3H), 2.37 (s, 1H), 1.45 (d, *J* = 6.0 Hz, 3H); ^{13}C NMR (125 MHz, $CDCl_3$) δ 144.6, 143.8, 134.4, 133.7, 131.2, 130.0, 129.7, 128.2, 127.8, 127.5, 127.4, 127.3, 125.5, 122.5, 82.1, 71.1, 66.0, 52.7, 31.0, 24.6, 21.5; IR (neat): 3443(bs), 2971, 2926, 2235(s), 1597, 1443, 1364, 1168, 1089, 756, 546; HRESIMS Calcd for $[C_{25}H_{25}NNaO_3S]^+$ (M + Na^+) 442.1447, found 442.1447.

***N*-(2-(1-hydroxypropyl)phenethyl)-4-methyl-*N*-(phenylethynyl)benzenesulfonamide (1ad)**

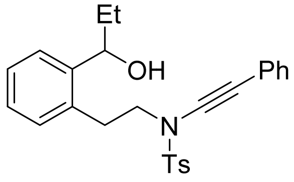

1ad

Pale yellow oil. ^{1}H NMR (400 MHz, $CDCl_3$) δ 7.77 (d, *J* = 8.4 Hz, 2H), 7.47 (dd, *J* = 8.0 Hz, *J* = 1.2 Hz, 1H), 7.41 – 7.35 (m, 2H), 7.34 – 7.28 (m, 5H), 7.28 – 7.24 (m, 1H), 7.22 – 7.14 (m, 2H), 4.87 (dd, *J* = 7.6 Hz, *J* = 5.6 Hz, 1H), 3.65 (t, *J* = 7.6 Hz, 2H), 3.18 – 2.98 (m, 2H), 2.43 (s, 3H), 2.01 (s, 1H), 1.88 – 1.68 (m, 2H), 0.94 (t, *J* = 7.6 Hz, 3H); ^{13}C NMR (100 MHz, $CDCl_3$) δ 144.7, 142.9, 134.6, 134.3, 131.4, 130.2, 129.8, 128.3, 127.9, 127.6, 127.5, 127.4, 126.2, 122.7, 82.2, 71.7, 71.2, 52.9, 31.5, 31.3, 21.6, 10.5; IR (neat):

3448(bs), 2918, 2232(s), 1494, 1454, 1362, 1166, 1089, 753, 587, 545; HRESIMS Calcd for $[C_{26}H_{27}NNaO_3S]^+$ $(M + Na^+)$ 456.1604, found 456.1601.

Compounds **1ae** and **1an** were prepared according to the following procedures.[1-3]

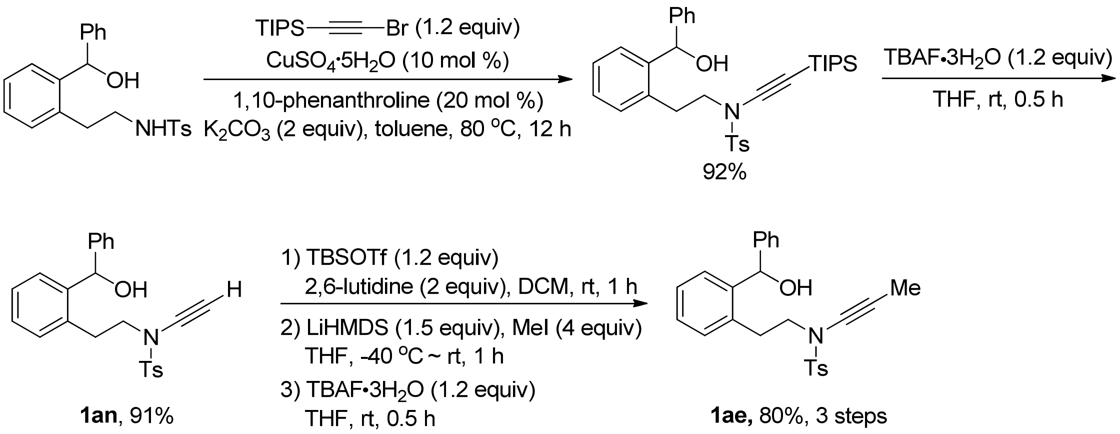

Supplementary Figure 137. Procedures for the preparation of ynamides **1ae** and **1an**.

_N_-(2-(hydroxy(phenyl)methyl)phenethyl)-4-methyl-_N_-(prop-1-yn-1-yl)benzenesulfonamide (1ae)

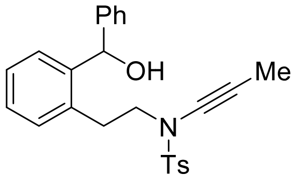

1ae

Colorless oil. ^{1}H NMR (400 MHz, $CDCl_3$) δ 7.66 (d, J = 8.4 Hz, 2H), 7.44 – 7.40 (m, 1H), 7.30 – 7.18 (m, 9H), 7.16 – 7.12 (m, 1H), 6.01 (d, J = 3.2 Hz, 1H), 3.41 (ddd, J = 15.6 Hz, J = 9.6 Hz, J = 5.6 Hz, 1H), 3.31 (ddd, J = 16.0 Hz, J = 9.6 Hz, J = 6.4 Hz, 1H), 3.03 – 2.84 (m, 2H), 2.62 (d, J = 3.2 Hz, 1H), 2.40 (s, 3H), 1.90 (s, 3H); ^{13}C NMR (100 MHz, $CDCl_3$) δ 144.2, 143.1, 141.6, 135.1, 134.5, 130.3, 129.6, 128.3, 127.8, 127.4, 127.0, 126.8, 72.7, 71.8, 66.0, 52.3, 31.2, 21.5, 3.3; IR (neat): 3533(bs), 3029, 2922, 2260(s), 1452, 1359, 1166, 1018, 760, 700, 592; HRESIMS Calcd for $[C_{25}H_{25}NNaO_3S]^+$ $(M + Na^+)$ 442.1447, found 442.1448.

***N*-(2-(1-hydroxy-2-phenylallyl)phenethyl)-4-methyl-*N*-(phenylethynyl)benzenesulfonamide (1af)**

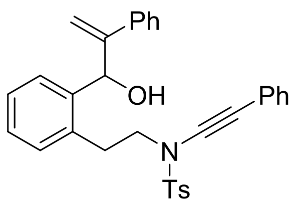

1af

Pale yellow oil. ^{1}H NMR (400 MHz, $CDCl_3$) δ 7.75 (d, J = 8.0 Hz, 2H), 7.48 – 7.42 (m, 1H), 7.36 – 7.32 (m, 2H), 7.30 – 7.23 (m, 7H), 7.20 – 7.12 (m, 6H), 5.93 (s, 1H), 5.47 (s, 1H), 5.36 (s, 1H), 3.75 – 3.65 (m, 1H), 3.63 – 3.52 (m, 1H), 3.17 – 3.04 (m, 2H), 2.38 (s, 1H), 2.39 (s, 3H); ^{13}C NMR (100 MHz, $CDCl_3$) δ 150.3, 144.6, 139.5(9), 139.5(6), 135.5, 134.5, 131.4, 130.3, 129.7, 128.3, 128.2, 128.1, 127.8, 127.6(2), 127.5(6), 127.5(3), 127.2, 126.7, 122.6, 114.5, 82.2, 71.7, 71.1, 52.7, 31.5, 21.5; IR (neat): 3444(bs), 2955, 2923, 2233(s), 1462, 1364, 1170, 1085, 761, 554, ; HRESIMS Calcd for $[C_{32}H_{29}NNaO_3S]^+$ (M + Na^+) 530.1760, found 530.1761.

Methyl-2-(hydroxy(2-(2-((4-methyl-*N*-(phenylethynyl)phenyl)sulfonamido)ethyl)phenyl)methyl)acrylate (1ag)

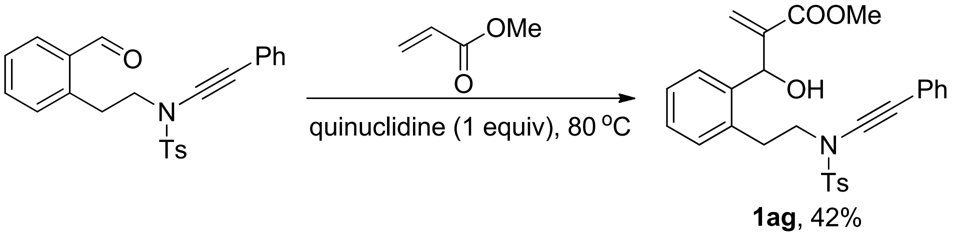

Supplementary Figure 138. Synthesis of ynamide **1ag**.

Compound **1ag** was prepared according to the above known procedure.[9] Pale yellow oil. ^{1}H NMR (400 MHz, $CDCl_3$) δ 7.78 (d, J = 8.4 Hz, 2H), 7.38 – 7.35 (m, 3H), 7.33 – 7.28 (m, 5H), 7.25 – 7.19 (m, 3H), 6.39 – 6.36 (m, 1H), 5.86 (d, J = 3.6 Hz, 1H), 5.84 – 5.81 (m, 1H), 3.77 – 3.71 (m, 1H), 3.67 (s, 3H), 3.65 – 3.61 (m, 1H), 3.22 (ddd, J = 15.2 Hz, J = 9.2 Hz, J = 5.6 Hz, 1H), 3.09 (ddd, J = 16.0 Hz, J = 9.2 Hz, J = 6.4 Hz, 1H), 2.88 (d, J = 4.0 Hz, 1H), 2.43 (s, 3H); ^{13}C NMR (100 MHz, $CDCl_3$) δ 166.6, 144.7, 142.0, 139.1, 135.5, 134.6, 131.4, 130.6, 129.8, 128.3, 128.2, 127.8, 127.6, 127.4, 127.2, 126.0, 122.7,

82.2, 71.1, 68.9, 52.7, 51.9, 31.6, 21.6; IR (neat): 3439(bs), 2923, 2235(s), 1716(s), 1492, 1441, 1364, 1168, 756, 692, 546; HRESIMS Calcd for $[C_{28}H_{27}NNaO_5S]^+$ (M + Na^+) 512.1502, found 512.1507.

N-(2-(1-hydroxyallyl)phenethyl)-4-methyl-N-(phenylethynyl)benzenesulfonamide (1ah)

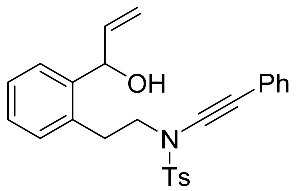

1ah

Pale yellow oil. ^{1}H NMR (500 MHz, $CDCl_3$) δ 7.78 (d, J = 8.5 Hz, 2H), 7.44 (dd, J = 7.5 Hz, J = 1.5 Hz, 1H), 7.40 – 7.35 (m, 2H), 7.34 – 7.28 (m, 5H), 7.27 – 7.17 (m, 3H), 6.04 (ddd, J = 17.5 Hz, J = 10.5 Hz, J = 5.5 Hz, 1H), 5.48 – 5.42 (m, 1H), 5.36 – 5.28 (m, 1H), 5.22 – 5.15 (m, 1H), 3.69 – 3.59 (m, 2H), 3.17 – 3.05 (m, 2H), 2.43 (s, 3H), 2.15 (d, J = 3.5 Hz, 1H); ^{13}C NMR (100 MHz, $CDCl_3$) δ 144.6, 140.7, 139.8, 134.9, 134.5, 131.4, 130.4, 129.8, 128.3, 128.0, 127.9, 127.6, 127.4, 127.1, 122.6, 115.4, 82.2, 71.6, 71.1, 52.7, 31.4, 21.6; IR (neat): 3414(bs), 2922, 2853, 2234(s), 1491, 1449, 1364, 1167, 1088, 754, 606; HRESIMS Calcd for $[C_{26}H_{25}NNaO_3S]^+$ (M + Na^+) 454.1447, found 454.1453.

N-(3-(2-(hydroxy(phenyl)methyl)phenyl)propyl)-4-methyl-N-(phenylethynyl)benzenesulfonamide (1ai)

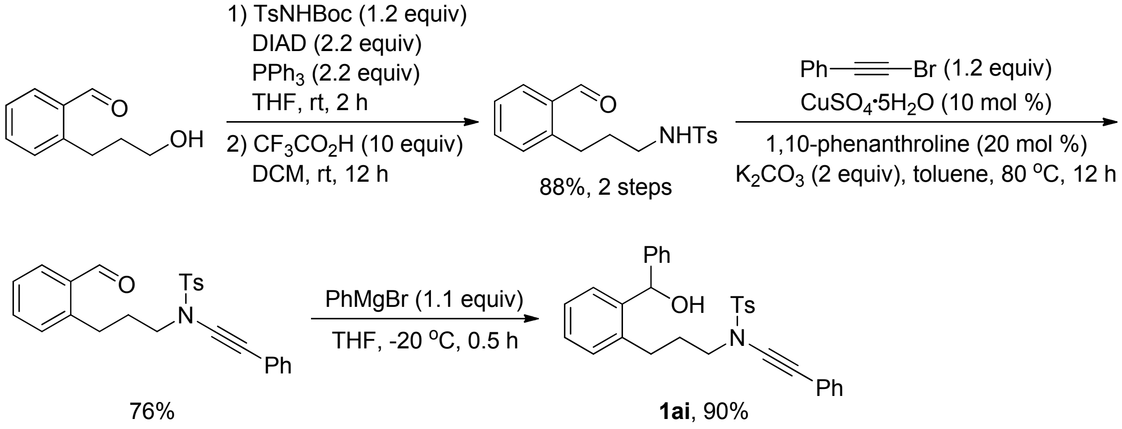

Supplementary Figure 139. Procedures for the preparation of ynamide **1ai**.

Compound **1ai** was prepared according to the above known procedures.[1] Pale yellow oil. ^{1}H NMR (500 MHz, $CDCl_3$) δ 7.79 (d, *J* = 8.0 Hz, 2H), 7.45 – 7.40 (m, 1H), 7.35 – 7.31 (m, 2H), 7.31 – 7.22 (m, 9H), 7.20 – 7.17 (m, 3H), 7.14 – 7.11 (m, 1H), 6.01 (s, 1H), 3.34 (t, *J* = 6.5 Hz, 2H), 2.74 (ddd, *J* = 15.5 Hz, *J* = 10.0 Hz, *J* = 6.0 Hz, 1H), 2.61 (ddd, *J* = 15.5 Hz, *J* = 10.0 Hz, *J* = 5.5 Hz, 1H), 2.54 (s, 1H), 2.39 (s, 3H), 1.96 – 1.79 (m, 2H); ^{13}C NMR (125 MHz, $CDCl_3$) δ 144.6, 143.3, 141.2, 138.3, 134.2, 131.3, 129.7, 129.3, 128.3, 128.2, 127.7, 127.6, 127.5, 127.3, 127.1, 126.8, 126.4, 122.6, 82.1, 72.5, 70.9, 51.0, 29.1, 28.8, 21.5; IR (neat): 3539(bs), 3061, 2925, 2236(s), 1595, 1450, 1364, 1168, 756, 676, 547; HRESIMS Calcd for $[C_{31}H_{29}NNaO_3S]^+$ (M + Na^+) 518.1760, found 518.1763.

_N_-(2-(3-(1-hydroxyallyl)-1-tosyl-1_H_-indol-2-yl)ethyl)-4-methyl-_N_-(phenylethynyl)benzenesulfonamide (1aj)

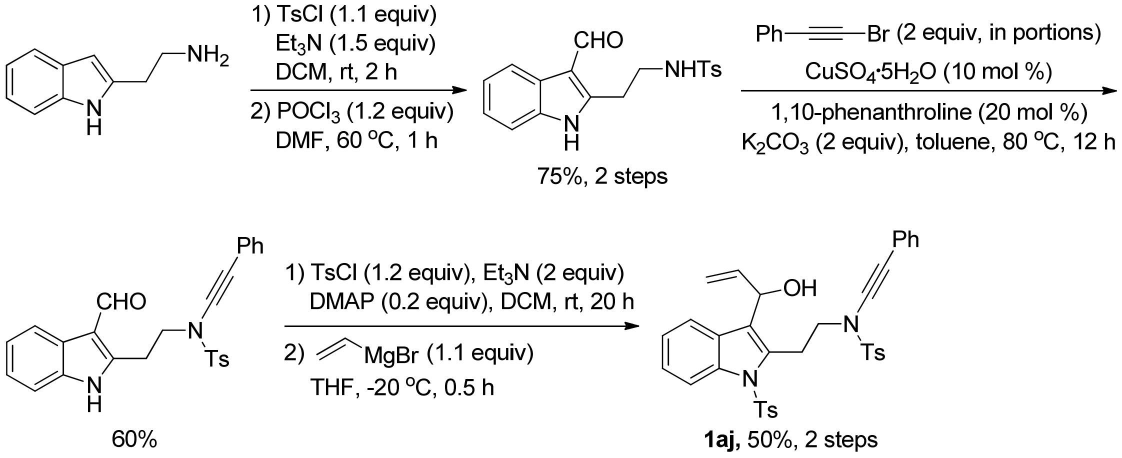

Supplementary Figure 140. Procedures for the preparation of ynamide **1aj**.

Compound **1aj** was prepared according to the above known procedures.[1,10] Pale yellow oil. ^{1}H NMR (400 MHz, $CDCl_3$) δ 8.14 (d, *J* = 8.4 Hz, 1H), 7.76 (d, *J* = 8.4 Hz, 2H), 7.74 – 7.69 (m, 1H), 7.55 (d, *J* = 8.4 Hz, 2H), 7.28 – 7.16 (m, 9H), 7.12 (d, *J* = 8.0 Hz, 2H), 6.12 (ddd, *J* = 17.2 Hz, *J* = 10.4 Hz, *J* = 4.8 Hz, 1H), 5.59 (d, *J* = 2.8 Hz, 1H), 5.29 – 5.21 (m, 1H), 5.16 – 5.11 (m, 1H), 4.03 – 3.93 (m, 1H), 3.88 – 3.79 (m, 1H), 3.48 (t, *J* = 6.4 Hz, 2H), 2.45 (s, 1H), 2.40 (s, 3H), 2.31 (s, 3H); ^{13}C NMR (100 MHz, $CDCl_3$) δ 144.9, 144.7, 138.5, 137.2, 135.6, 134.6, 132.6, 131.4, 129.8, 129.7, 128.4, 128.2, 127.8, 127.5, 126.2, 124.7, 123.5, 122.4, 121.3, 115.4, 115.1, 82.0, 70.9, 68.0, 52.3, 26.5, 21.6, 21.5;

IR (neat): 3446(bs), 2923, 2850, 2235(s), 1452, 1362, 1170, 1086, 676, 574; HRESIMS Calcd for $[C_{35}H_{32}N_2NaO_5S_2]^+$ (M + Na^+) 647.1645, found 647.1652.

***tert*-butyl-3-(1-hydroxyallyl)-2-(2-((4-methyl-*N*-(phenylethynyl)phenyl)sulfonamido)ethyl)-1*H*-indole-1-carboxylate (1ak)**

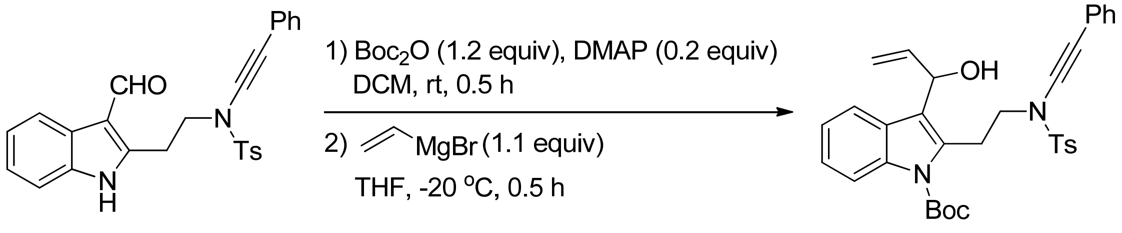

1ak, 74%, 2 steps

Supplementary Figure 141. Procedures for the preparation of ynamide **1ak**.

Compound **1ak** was prepared according to the above known procedures.[1,10] Pale yellow oil. ^{1}H NMR (400 MHz, $CDCl_3$) δ 8.03 (d, J = 8.4 Hz, 1H), 7.76 (dd, J = 8.0 Hz, J = 0.8 Hz, 1H), 7.64 (d, J = 8.4 Hz, 2H), 7.27 – 7.23 (m, 6H), 7.22 – 7.16 (m, 1H), 7.12 (d, J = 8.0 Hz, 2H), 6.16 (ddd, J = 17.2 Hz, J = 10.4 Hz, J = 4.8 Hz, 1H), 5.62 (d, J = 4.4 Hz, 1H), 5.40 – 5.33 (m, 1H), 5.19 – 5.12 (m, 1H), 4.02 – 3.92 (m, 1H), 3.85 – 3.75 (m, 1H), 3.57 – 3.43 (m, 2H), 2.36 (s, 3H), 2.22 (s, 1H), 1.69 (s, 9H); ^{13}C NMR (100 MHz, $CDCl_3$) δ 150.5, 144.6, 138.8, 136.2, 134.5, 132.8, 131.6, 129.6, 128.2, 127.9, 127.6, 127.4, 124.1, 122.4, 122.3, 122.2, 120.8, 115.6, 114.9, 84.3, 82.0, 71.1, 68.0, 51.5, 28.2, 26.1, 21.5; IR (neat): 3439(bs), 2980, 2926, 2237(s), 1640(s), 1454, 1367, 1319, 1167, 1118, 754, 574; HRESIMS Calcd for $[C_{33}H_{34}N_2NaO_5S]^+$ (M + Na^+) 593.2081, found 593.2080.

***N*-(2-(2-(1-hydroxyallyl)benzo[*b*]thiophen-3-yl)ethyl)-4-methyl-*N*-(phenylethynyl)benzenesulfonamide (1al)**

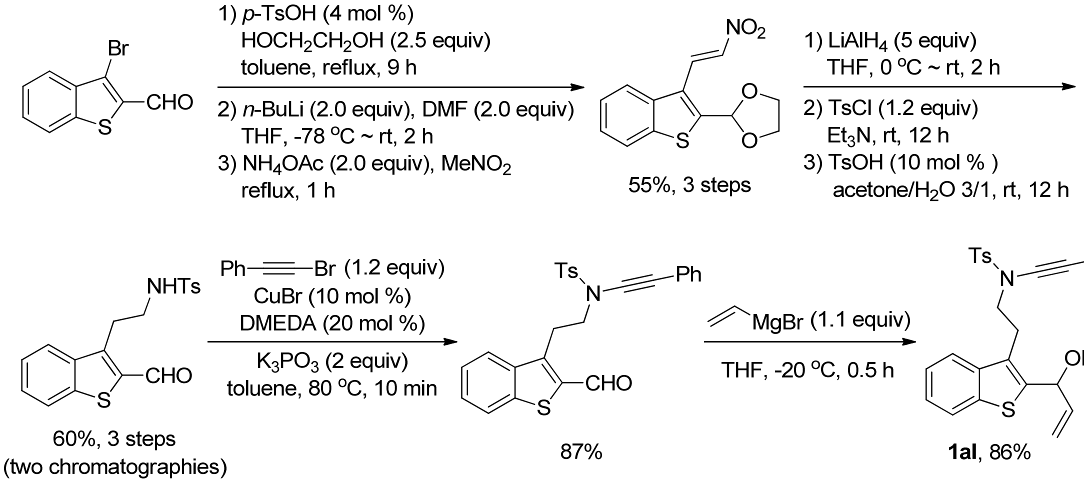

Supplementary Figure 142. Procedures for the preparation of ynamide **1al**.

Compound **1al** was prepared according to the above known procedures.[11-13] Pale yellow oil. ^{1}H NMR (400 MHz, CDCl$_3$) δ 7.74 (d, *J* = 7.6 Hz, 1H), 7.71 (d, *J* = 8.0 Hz, 2H), 7.66 (d, *J* = 8.0 Hz, 1H), 7.37 – 7.20 (m, 9H), 6.16 – 5.99 (m, 1H), 5.67 (d, *J* = 5.6 Hz, 1H), 5.42 (d, *J* = 17.2 Hz, 1H), 5.20 (d, *J* = 10.0 Hz, 1H), 3.74 – 3.55 (m, 2H), 3.26 (t, *J* = 7.2 Hz, 2H), 2.83 (s, 1H), 2.36 (s, 3H); ^{13}C NMR (100 MHz, CDCl$_3$) δ 144.6, 143.8, 139.4, 138.9, 138.7, 134.3, 131.3, 129.7, 128.2, 127.9, 127.3, 127.2, 124.3, 124.1, 122.6, 122.4, 121.1, 116.0, 82.1, 71.0, 69.5, 51.0, 25.8, 21.5; IR (neat): 3446(bs), 3057, 2918, 2235(s), 1434, 1362, 1170, 1085, 674, 547; HRESIMS Calcd for $[C_{28}H_{25}NNaO_3S_2]^+$ (M + Na$^+$) 510.1168, found 510.1167.

_N_-(2-(3-(hydroxy(phenyl)methyl)thiophen-2-yl)ethyl)-4-methyl-_N_-(phenylethynyl)benzenesulfonamide (1am)

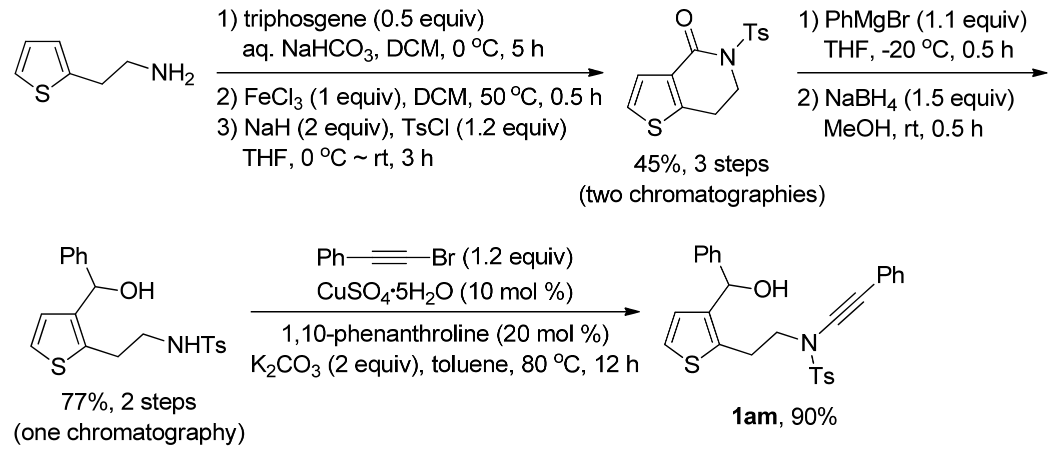

Supplementary Figure 143. Procedures for the preparation of ynamide **1am**.

Compound **1am** was prepared according to the above known procedures.[1,3,14] Pale yellow oil. ^{1}H NMR (400 MHz, $CDCl_3$) δ 7.75 (d, *J* = 8.4 Hz, 2H), 7.38 – 7.33 (m, 4H), 7.32 – 7.26 (m, 7H), 7.26 – 7.21 (m, 1H), 7.09 (d, *J* = 5.6 Hz, 1H), 6.90 (d, *J* = 5.2 Hz, 1H), 5.95 (s, 1H), 3.70 – 3.57 (m, 2H), 3.35 – 3.17 (m, 2H), 2.46 (s, 1H), 2.43 (s, 3H); ^{13}C NMR (100 MHz, $CDCl_3$) δ 144.7, 143.2, 141.6, 135.1, 134.5, 131.5, 129.8, 128.4, 128.3, 128.0, 127.7, 127.4, 126.2, 123.5, 122.6, 81.9, 71.3, 70.2, 52.9, 27.0, 21.6; IR (neat): 3463(bs), 2920, 2858, 2237(s), 1496, 1362, 1167, 1088, 759, 671; HRESIMS Calcd for $[C_{28}H_{25}NNaO_3S_2]^+$ (M + Na$^+$) 510.1168, found 510.1168.

_N_-ethynyl-_N_-(2-(hydroxy(phenyl)methyl)phenethyl)-4-methylbenzenesulfonamide (1an)

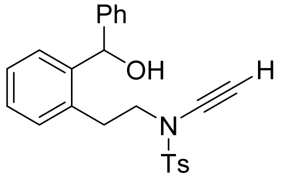

1an

Pale yellow oil. ^{1}H NMR (500 MHz, $CDCl_3$) δ 7.67 (d, *J* = 8.5 Hz, 2H), 7.40 (dd, *J* = 7.5 Hz, *J* = 2.0 Hz, 1H), 7.29 – 7.18 (m, 9H), 7.14 (dd, *J* = 7.0 Hz, *J* = 2.0 Hz, 1H), 6.00 (d, *J* = 4.0 Hz, 1H), 3.44 (ddd, *J* = 16.0 Hz, *J* = 10.0 Hz, *J* = 6.0 Hz, 1H), 3.33 (ddd, *J* = 16.0 Hz, *J* = 9.5 Hz, *J* = 6.0 Hz, 1H), 3.04 – 2.96 (m, 1H), 2.92 (ddd, *J* = 16.0 Hz, *J* = 10.0 Hz, *J* = 6.0 Hz, 1H), 2.78 (s, 1H), 2.61 (d, *J* = 4.0 Hz, 1H), 2.40 (s, 3H); ^{13}C NMR (125 MHz, $CDCl_3$) δ 144.7, 143.0, 141.6, 134.8, 134.4, 130.4, 129.7, 128.4, 127.8, 127.5, 127.4(6), 127.4(1), 127.1, 126.8, 75.9, 72.8, 59.5, 52.1, 31.1, 21.5; IR (neat): 3440(bs), 2924, 2136(s), 1597, 1451, 1361, 1168, 700, 597, 544; HRESIMS Calcd for $[C_{24}H_{23}NNaO_3S]^+$ (M + Na$^+$) 428.1291, found 428.1292.

_N_-(2-(2-hydroxy-2-phenylethyl)benzyl)-4-methyl-_N_-(phenylethynyl)benzenesulfonamide (1ao)

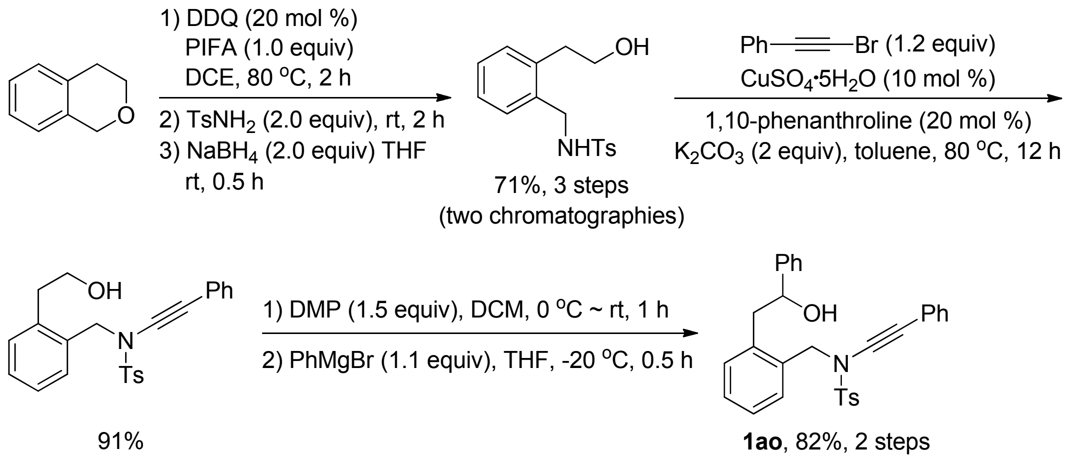

Supplementary Figure 144. Procedures for the preparation of ynamide **1ao**.

Compound **1ao** was prepared according to the above known procedures.[1,15,16] Pale yellow oil. ^{1}H NMR (400 MHz, $CDCl_3$) δ 7.81 (d, *J* = 8.4 Hz, 2H), 7.35 – 7.11 (m, 16H), 4.92 – 4.85 (m, 1H), 4.54 (d, *J* = 13.2 Hz, 1H), 4.42 (d, *J* = 13.2 Hz, 1H), 3.19 (dd, *J* = 14.0 Hz, *J* = 8.4 Hz, 1H), 3.07 (dd, *J* = 14.0 Hz, *J* = 5.2 Hz, 1H), 2.43 (s, 3H), 2.13 (d, *J* = 3.2 Hz, 1H); ^{13}C NMR (100 MHz, $CDCl_3$) δ 144.7, 143.7, 137.5, 133.9, 132.7, 131.0, 130.9, 130.8, 129.7, 128.6, 128.3, 128.1, 127.8, 127.6, 127.5, 126.6, 125.9, 122.6, 82.6, 75.2, 71.5, 53.0, 42.2, 21.6; IR (neat): 3444(bs), 2922, 2954, 2233(s), 1454, 1363, 1167, 1020, 690, 544, ; HRESIMS Calcd for $[C_{30}H_{27}NNaO_3S]^+$ (M + Na^+) 504.1604, found 504.1604.

N-(5-hydroxy-5-phenylpentyl)-4-methyl-*N*-(phenylethynyl)benzenesulfonamide (1ap)

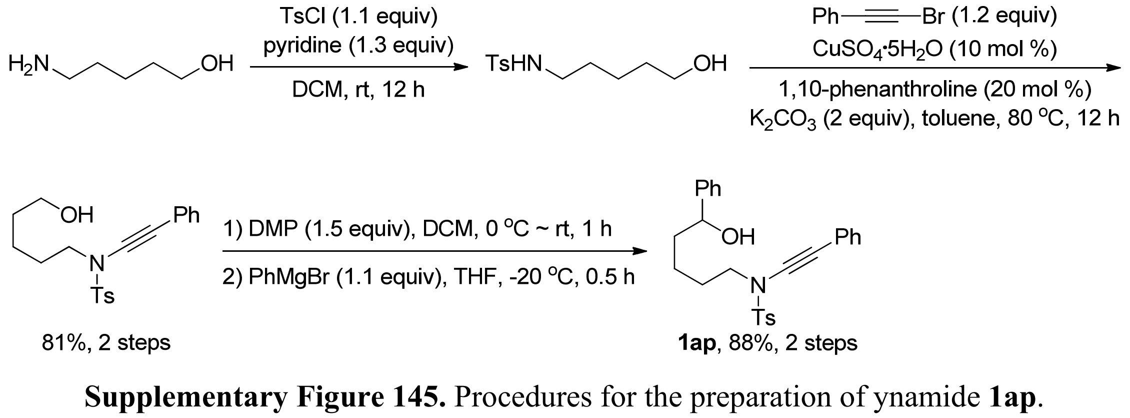

Supplementary Figure 145. Procedures for the preparation of ynamide **1ap**.

Compound **1ap** was prepared according to the above known procedures.[1,17] Pale yellow oil. ^{1}H NMR (500 MHz, $CDCl_3$) δ 7.81 (d, *J* = 8.5 Hz, 2H), 7.35 – 7.24 (m, 12H), 4.63 (t, *J* = 6.5 Hz, 1H), 3.36 (t, *J* = 7.0 Hz, 2H), 2.43 (s, 3H), 1.97 (s, 1H), 1.84 – 1.68 (m, 4H),

1.53 – 1.44 (m, 1H), 1.40 – 1.30 (m, 1H); ^{13}C NMR (125 MHz, $CDCl_3$) δ 144.6, 134.6, 131.3, 129.7, 128.4, 128.2, 127.7, 127.6, 127.5, 125.8, 122.9, 82.4, 74.3, 70.7, 51.4, 38.4, 27.8, 22.5, 21.6; IR (neat): 3419(bs), 2939, 2236(s), 1597, 1453, 1363, 1168, 1090, 756, 585; HRESIMS Calcd for $[C_{26}H_{27}NNaO_3S]^+$ (M + Na^+) 456.1604, found 456.1606.

_N_-(2-(methoxy(phenyl)methyl)phenethyl)-4-methyl-_N_-(phenylethynyl)benzenesulfonamide (1aq)

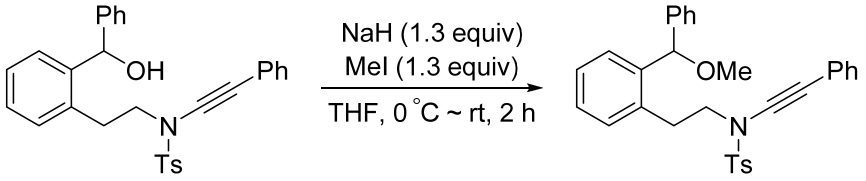

1aq, 87%

Supplementary Figure 146. Synthesis of ynamide **1aq**.

Compound **1aq** was prepared according to the above known procedure. Yellow oil. ^{1}H NMR (400 MHz, $CDCl_3$) δ 7.76 (d, J = 8.0 Hz, 2H), 7.40 – 7.34 (m, 3H), 7.32 – 7.16 (m, 13H), 5.46 (s, 1H), 3.53 (ddd, J = 16.0 Hz, J = 10.0 Hz, J = 6.0 Hz, 1H), 3.44 (ddd, J = 16.4 Hz, J = 10.0 Hz, J = 6.4 Hz, 1H), 3.35 (s, 3H), 3.12 – 2.94 (m, 2H), 2.41 (s, 3H); ^{13}C NMR (100 MHz, $CDCl_3$) δ 144.6, 140.8, 139.7, 135.5, 134.5, 131.4, 130.5, 129.7, 128.3, 128.2, 127.8(7), 127.8(5), 127.8(1), 127.5(3), 127.4(7), 127.4(0), 127.0, 122.7, 82.5, 82.4, 71.0, 57.1, 52.5, 31.4, 21.6; IR (neat): 2927, 2821, 2234(s), 1492, 1451, 1367, 1169, 1089, 755, 692, 546; HRESIMS Calcd for $[C_{31}H_{29}NNaO_3S]^+$ (M + Na^+) 518.1760, found 518.1762.

(_S_)-_N_-(1,10-bis(3,5-di-tert-butyl-4-methoxyphenyl)-12-oxido-4,5,6,7-tetrahydrodiindeno[7,1-de:1',7'-_fg_][1,3,2]dioxaphosphocin-12-yl)-1,1,1-trifluoromethanesulfonamide (Cat. 3)

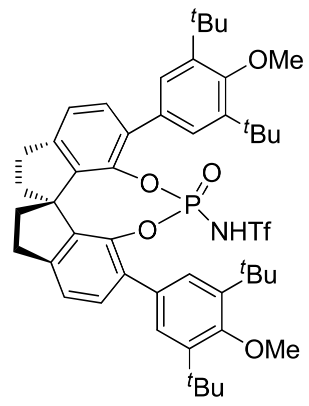

Cat. 3

Compound **Cat. 3** was prepared according to the reported procedure.[18] White solid (mp 156-157 °C). ^{1}H NMR (400 MHz, $CDCl_3$) δ 7.45 (s, 2H), 7.36 (dd, J = 7.6 Hz, J = 0.4 Hz, 1H), 7.25 – 7.18 (m, 5H), 3.70 (s, 3H), 3.67 (s, 3H), 3.21 – 3.06 (m, 2H), 3.00 – 2.88 (m, 2H), 2.41 – 2.32 (m, 2H), 2.29 – 2.16 (m, 2H), 1.41 (s, 18H), 1.39 (s, 18H); ^{13}C NMR (125 MHz, $CDCl_3$) ^{13}C NMR (100 MHz, $CDCl_3$) δ 159.7, 159.0, 145.8 (d, J = 2.4 Hz), 144.9 (d, J = 2.4 Hz), 144.5, 142.9, 141.7 (d, J = 10.0 Hz), 140.4, 140.3 (d, J = 0.9 Hz), 140.2, 140.1 (d, J = 3.4 Hz), 136.5 (d, J = 3.9 Hz), 133.4 (d, J = 4.1 Hz), 131.7, 131.6 (d, J = 2.1 Hz), 130.7, 130.1, 128.3, 128.1, 123.6 (d, J = 2.4 Hz), 123.3 (d, J = 2.3 Hz), 64.2, 64.1, 60.3(7), 60.3(6), 39.0, 38.5, 35.9, 35.7, 31.9(2), 31.9(0), 30.2; ^{31}P NMR (162 MHz, $CDCl_3$) δ -15.7(s); ^{19}F NMR (376 MHz, $CDCl_3$) δ -77.6(s); IR (neat): 3452(bs), 2923, 1462, 1413, 1397, 1302, 1263, 1211, 1186, 1093, 1021, 891, 628; HRESIMS Calcd for $[C_{48}H_{59}F_3NNaO_7PS]^+$ (M + Na^+) 904.3594, found 904.3595.

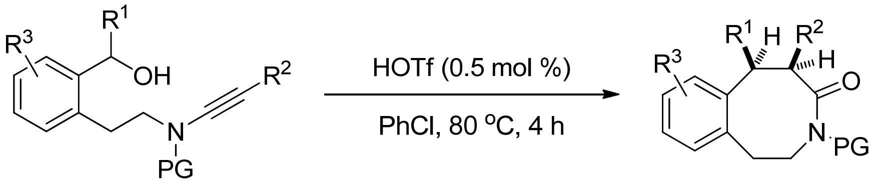

Supplementary Figure 147. Synthesis of 3-benzazocinones **2**.

General procedure for the synthesis of 3-benzazocinones 2:

To a mixture of the ynamide **1** (0.20 mmol) in PhCl (3.75 mL) at room temperature, HOTf (0.001 mmol/0.25 mL) in 0.25 mL PhCl was added. Then, the reaction mixture was stirred at 80 °C and the progress of the reaction was monitored by TLC. The reaction typically took 4 h. Upon completion, the mixture was concentrated and the residue was purified by chromatography on silica gel (eluent: hexanes/ethyl acetate) to afford the desired 3-benzazocinones **2**.

5,6-diphenyl-3-tosyl-2,3,5,6-tetrahydrobenzo[*d*]azocin-4(1*H*)-one (2a)

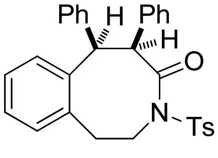

2a

Compound **2a** was prepared in 94% yield (90.6 mg) according to the general procedure (Table 2, entry 1). Pale yellow solid (mp 177-178 °C). ^{1}H NMR (500 MHz, $CDCl_3$) δ 7.48 (d, *J* = 8.5 Hz, 2H), 7.21 (d, *J* = 4.0 Hz, 2H), 7.19 – 7.16 (m, 2H), 7.15 – 7.05 (m, 7H), 7.04 – 6.98 (m, 2H), 6.87 (d, *J* = 7.5 Hz, 2H), 6.83 (d, *J* = 8.0 Hz, 1H), 5.06 (d, *J* = 5.0 Hz, 1H), 4.93 (d, *J* = 5.0 Hz, 1H), 4.70 – 4.59 (m, 1H), 3.97 – 3.87 (m, 1H), 3.54 – 3.44 (m, 1H), 3.19 – 3.10 (m, 1H), 2.37 (s, 3H); ^{13}C NMR (125 MHz, $CDCl_3$) δ 173.5, 143.9, 140.7, 138.9, 137.3, 137.0, 136.0, 131.4, 131.3, 129.8, 129.7, 129.1, 128.5, 127.8, 127.6, 127.1, 127.0, 126.5, 55.7, 54.1, 45.5, 36.1, 21.5; IR (neat): 2962, 2927, 1643(s), 1454, 1260, 1164, 1087, 1018, 799, 699, 561; HRESIMS Calcd for $[C_{30}H_{27}NNaO_3S]^+$ (M + Na^+) 504.1604, found 504.1604.

***N*-(2-(hydroxy(phenyl)methyl)phenethyl)-2-phenyl-*N*-tosylacetamide (2a′)**

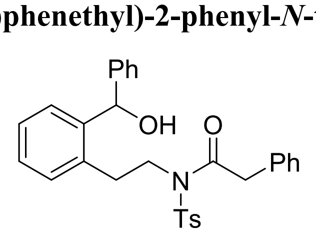

2a′

Pale yellow oil. ^{1}H NMR (500 MHz, $CDCl_3$) δ 7.65 (d, *J* = 8.0 Hz, 2H), 7.37 – 7.32 (m, 3H), 7.31 – 7.21 (m, 11H), 7.06 – 7.01 (m, 2H), 6.17 (s, 1H), 3.97 (ddd, *J* = 16.0 Hz, *J* =

11.0 Hz, J = 5.5 Hz, 1H), 3.91 (s, 2H), 3.90 – 3.83 (m, 1H), 3.09 (ddd, J = 15.5 Hz, J = 10.5 Hz, J = 5.5 Hz, 1H), 2.98 (ddd, J = 16.5 Hz, J = 11.0 Hz, J = 6.0 Hz, 1H), 2.89 (s, 1H), 2.41 (s, 3H); ^{13}C NMR (125 MHz, $CDCl_3$) δ 171.2, 145.0, 143.4, 141.9, 136.4, 135.6, 133.4, 130.8, 129.9, 129.4, 128.5, 128.3, 127.9, 127.8, 127.4, 127.3, 127.2, 127.1, 126.8, 72.7, 48.3, 42.7, 33.0, 21.6; IR (neat): 3465, 2956, 2922, 1692(s), 1597, 1453, 1351, 1158, 1084, 724, 697; HRESIMS Calcd for $[C_{30}H_{29}NNaO_4S]^+$ (M + Na^+) 522.1710, found 522.1716.

5,6-diphenyl-3-(phenylsulfonyl)-2,3,5,6-tetrahydrobenzo[*d*]azocin-4(1*H*)-one (2b)

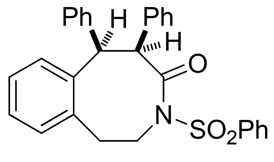

2b

Compound **2b** was prepared in 89% yield (83.2 mg) according to the general procedure except by using 1 mol % of HOTf as catalyst (Table 2, entry 2). Pale yellow oil. ^{1}H NMR (500 MHz, $CDCl_3$) δ 7.58 (d, J = 7.5 Hz, 2H), 7.52 – 7.47 (m, 1H), 7.35 – 7.29 (m, 2H), 7.24 – 7.20 (m, 2H), 7.19 – 7.16 (m, 2H), 7.14 – 7.11 (m, 3H), 7.10 – 7.05 (m, 2H), 7.04 – 6.98 (m, 2H), 6.85 (d, J = 7.5 Hz, 2H), 6.82 (d, J = 8.0 Hz, 1H), 5.08 (d, J = 5.5 Hz, 1H), 4.92 (d, J = 5.5 Hz, 1H), 4.73 – 4.63 (m, 1H), 3.99 – 3.89 (m, 1H), 3.57 – 3.47 (m, 1H), 3.22 – 3.13 (m, 1H); ^{13}C NMR (125 MHz, $CDCl_3$) δ 173.5, 140.7, 139.1, 138.8, 137.1, 136.8, 133.0, 131.5, 131.3, 129.9, 129.8, 128.5, 127.8(9), 127.8(6), 127.7, 127.2, 127.0, 126.6, 55.5, 54.2, 45.4, 36.0; IR (neat): 2925, 2848, 1696(s), 1494, 1449, 1350, 1167, 1088, 699, 596; HRESIMS Calcd for $[C_{29}H_{25}NNaO_3S]^+$ (M + Na^+) 490.1447, found 490.1447.

3-((4-bromophenyl)sulfonyl)-5,6-diphenyl-2,3,5,6-tetrahydrobenzo[*d*]azocin-4(1*H*)-one (2c)

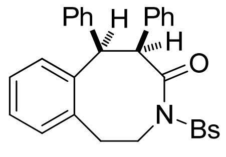

2c

Compound **2c** was prepared in 73% yield (79.7 mg) according to the general procedure except by using 1 mol % of HOTf as catalyst (Table 2, entry 3). Pale yellow solid (mp 190-191 °C). ^{1}H NMR (400 MHz, $CDCl_3$) δ 7.47 – 7.38 (m, 4H), 7.26 – 7.19 (m, 4H), 7.18 – 7.08 (m, 5H), 7.05 – 6.99 (m, 2H), 6.89 – 6.80 (m, 3H), 5.04 (d, *J* = 5.2 Hz, 1H), 4.89 (d, *J* = 5.2 Hz, 1H), 4.78 – 4.68 (m, 1H), 3.94 (ddd, *J* = 15.6 Hz, *J* = 6.0 Hz, *J* = 2.4 Hz, 1H), 3.55 – 3.45 (m, 1H), 3.16 (ddd, *J* = 14.8 Hz, *J* = 5.6 Hz, *J* = 2.0 Hz, 1H); ^{13}C NMR (100 MHz, $CDCl_3$) δ 173.3, 140.9, 138.3, 137.9, 137.3, 136.8, 131.7, 131.5, 131.3, 130.2, 129.8, 129.6, 128.3, 128.0, 127.9, 127.8, 127.3, 127.1, 126.6, 55.2, 54.4, 45.3, 35.9; IR (neat): 3028, 2927, 1698(s), 1573, 1390, 1351, 1168, 1084, 737, 700, 542; HRESIMS Calcd for $[C_{29}H_{24}BrNNaO_3S]^+$ (M + Na^+) 568.0552, found 568.0551.

5-(4-fluorophenyl)-6-phenyl-3-tosyl-2,3,5,6-tetrahydrobenzo[*d*]azocin-4(1*H*)-one (2d)

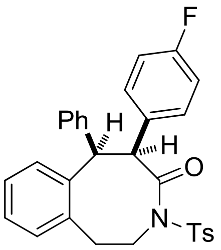

2d

Compound **2d** was prepared in 87% yield (86.9 mg) according to the general procedure (Table 2, entry 4). Pale yellow oil. ^{1}H NMR (400 MHz, $CDCl_3$) δ 7.44 (d, *J* = 8.4 Hz, 2H), 7.22 (d, *J* = 4.0 Hz, 2H), 7.16 – 7.06 (m, 6H), 7.05 – 6.99 (m, 2H), 6.84 – 6.75 (m, 5H), 5.08 (d, *J* = 6.0 Hz, 1H), 4.87 (d, *J* = 5.6 Hz, 1H), 4.72 – 4.60 (m, 1H), 3.93 (ddd, *J* = 15.2 Hz, *J* = 6.0 Hz, *J* = 3.2 Hz, 1H), 3.58 – 3.48 (m, 1H), 3.16 (ddd, *J* = 14.4 Hz, *J* = 6.0 Hz, *J* = 3.2 Hz, 1H), 2.38 (s, 3H); ^{13}C NMR (100 MHz, $CDCl_3$) δ 173.5, 161.7 (*J* = 244.8 Hz), 144.0, 140.3, 138.6, 136.7, 135.9, 132.7 (*J* = 3.3 Hz), 131.5(3) (*J* = 7.7 Hz), 131.5(1), 131.2, 130.0, 129.1, 128.5, 127.9, 127.7, 127.1, 126.6, 114.6 (*J* = 21.0 Hz), 54.6, 54.3, 45.3, 35.9, 21.5; IR (neat): 2925, 1698(s), 1508, 1346, 1165, 1119, 1086, 736, 703, 546; HRESIMS Calcd for $[C_{30}H_{26}FNNaO_3S]^+$ (M + Na^+) 522.1510, found 522.1504.

5-(4-chlorophenyl)-6-phenyl-3-tosyl-2,3,5,6-tetrahydrobenzo[*d*]azocin-4(1*H*)-one (2e)

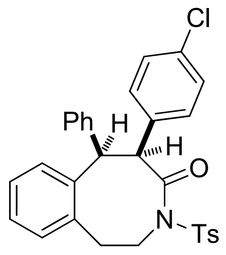

2e

Compound **2e** was prepared in 93% yield (96.0 mg) according to the general procedure (Table 2, entry 5). Pale yellow solid (mp 179-180 °C). ^{1}H NMR (400 MHz, $CDCl_3$) δ 7.44 (d, *J* = 8.4 Hz, 2H), 7.22 (m, *J* = 5.2 Hz, 2H), 7.13 – 7.06 (m, 8H), 7.05 – 7.00 (m, 2H), 6.84 – 6.77 (m, 3H), 5.06 (d, *J* = 6.0 Hz, 1H), 4.88 (d, *J* = 5.6 Hz, 1H), 4.72 – 4.59 (m, 1H), 3.92 (ddd, *J* = 15.2 Hz, *J* = 5.6 Hz, *J* = 2.8 Hz, 1H), 3.59 – 3.42 (m, 1H), 3.15 (ddd, *J* = 14.4 Hz, *J* = 5.6 Hz, *J* = 2.8 Hz, 1H), 2.38 (s, 3H); ^{13}C NMR (125 MHz, $CDCl_3$) δ 173.3, 144.1, 140.2, 138.4, 136.7, 135.9, 135.5, 132.9, 131.5, 131.2, 131.1, 130.0, 129.1, 128.5, 127.9, 127.8, 127.7, 127.1, 126.7, 54.7, 54.0, 45.4, 35.8, 21.5; IR (neat): 2924, 1697(s), 1596, 1492, 1346, 1164, 1088, 747, 703, 543; HRESIMS Calcd for $[C_{30}H_{26}ClNNaO_3S]^+$ (M + Na^+) 538.1214, found 538.1214.

5-(4-bromophenyl)-6-phenyl-3-tosyl-2,3,5,6-tetrahydrobenzo[*d*]azocin-4(1H)-one (2f)

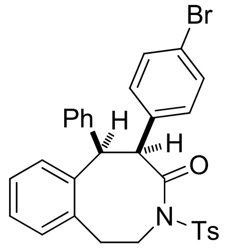

2f

Compound **2f** was prepared in 88% yield (98.6 mg) according to the general procedure (Table 2, entry 6). Yellow solid (mp 199-200 °C). ^{1}H NMR (400 MHz, $CDCl_3$) δ 7.44 (d, *J* = 8.4 Hz, 2H), 7.26 – 7.21 (m, 4H), 7.14 – 7.07 (m, 4H), 7.06 – 6.98 (m, 4H), 6.84 – 6.76 (m, 3H), 5.04 (d, *J* = 6.0 Hz, 1H), 4.88 (d, *J* = 6.0 Hz, 1H), 4.71 – 4.58 (m, 1H), 3.92 (ddd, *J* = 15.6 Hz, *J* = 6.0 Hz, *J* = 2.4 Hz, 1H), 3.57 – 3.45 (m, 1H), 3.15 (ddd, *J* = 14.4 Hz, *J* = 6.0 Hz, *J* = 2.8 Hz, 1H), 2.38 (s, 3H); ^{13}C NMR (100 MHz, $CDCl_3$) δ 173.2, 144.1, 140.2, 138.4, 136.7, 136.0, 135.8, 131.6, 131.5, 131.2, 130.8, 129.9, 129.1, 128.5, 127.9,

127.7, 127.1, 126.7, 121.2, 54.8, 54.0, 45.4, 35.9, 21.5; IR (neat): 2925, 1698(s), 1489, 1346, 1165, 1118, 1086, 1011, 702, 542; HRESIMS Calcd for $[C_{30}H_{26}BrNNaO_3S]^+$ (M + Na^+) 582.0709, found 582.0709.

4-(4-oxo-6-phenyl-3-tosyl-1,2,3,4,5,6-hexahydrobenzo[*d*]azocin-5-yl)benzonitrile (2g)

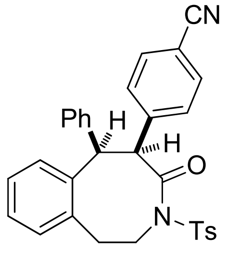

2g

Compound **2g** was prepared in 96% yield (97.3 mg) according to the general procedure (Table 2, entry 7). Pale yellow solid (mp 181-182 °C). ^{1}H NMR (500 MHz, $CDCl_3$) δ 7.41 (d, *J* = 8.5 Hz, 2H), 7.36 (d, *J* = 8.5 Hz, 2H), 7.31 (d, *J* = 8.5 Hz, 2H), 7.25 – 7.21 (m, 2H), 7.14 – 7.06 (m, 4H), 7.05 – 6.99 (m, 2H), 6.81 – 6.71 (m, 3H), 5.14 (d, *J* = 6.5 Hz, 1H), 4.87 (d, *J* = 6.5 Hz, 1H), 4.79 – 4.67 (m, 1H), 3.96 (ddd, *J* = 15.5 Hz, *J* = 6.5 Hz, *J* = 4.0 Hz, 1H), 3.61 – 3.50 (m, 1H), 3.21 (ddd, *J* = 14.5 Hz, *J* = 7.0 Hz, *J* = 4.5 Hz, 1H), 2.39 (s, 3H); ^{13}C NMR (100 MHz, $CDCl_3$) δ 172.3, 144.2, 142.3, 139.7, 137.8, 136.1, 135.6, 131.5, 131.3, 130.9, 130.5, 130.1, 129.0, 128.4, 128.0, 127.8, 127.1, 126.9, 118.6, 110.6, 54.1, 54.0, 45.0, 35.4, 21.5; IR (neat): 2925, 2227, 1635(s), 1453, 1345, 1164, 1118, 1087, 733, 543; HRESIMS Calcd for $[C_{31}H_{26}N_2NaO_3S]^+$ (M + Na^+) 529.1556, found 529.1555.

6-phenyl-3-tosyl-5-(4-(trifluoromethyl)phenyl)-2,3,5,6-tetrahydrobenzo[*d*]azocin-4(1*H*)-one (2h)

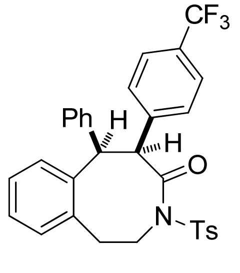

2h

Compound **2h** was prepared in 96% yield (105.5 mg) according to the general procedure (Table 2, entry 8). Pale yellow oil. ^{1}H NMR (400 MHz, $CDCl_3$) δ 7.45 (d, *J* = 8.4 Hz, 2H), 7.38 (d, *J* = 8.0 Hz, 2H), 7.30 (d, *J* = 8.0 Hz, 2H), 7.27 – 7.20 (m, 2H), 7.17 – 7.07 (m, 4H), 7.06 – 6.97 (m, 2H), 6.87 – 6.69 (m, 3H), 5.13 (d, *J* = 5.6 Hz, 1H), 4.91 (d, *J* = 5.6 Hz, 1H), 4.78 – 4.60 (m, 1H), 4.02 – 3.82 (m, 1H), 3.62 – 3.41 (m, 1H), 3.27 – 3.08 (m, 1H), 2.39 (s, 3H); ^{13}C NMR (100 MHz, $CDCl_3$) δ 172.9, 144.2, 141.2, 140.2, 138.2, 136.7, 135.8, 131.6, 131.2, 130.2, 129.9, 129.1, 128.5, 128.0, 127.9, 127.2, 126.8, 124.6 (q, *J* = 3.5 Hz), 55.0, 54.1, 45.4, 35.8, 21.5; IR (neat): 2927, 1698(s), 1454, 1325, 1165, 1120, 1069, 737, 677, 542; HRESIMS Calcd for $[C_{31}H_{26}F_3NNaO_3S]^+$ (M + Na$^+$) 572.1478, found 572.1479.

6-phenyl-5-(*p*-tolyl)-3-tosyl-2,3,5,6-tetrahydrobenzo[*d*]azocin-4(1*H*)-one (2i)

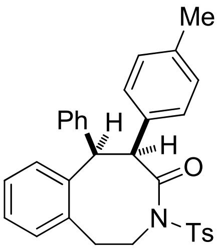

2i

Compound **2i** was prepared in 82% yield (81.3 mg) according to the general procedure (Table 2, entry 9). Pale yellow solid (mp 174-175 °C). ^{1}H NMR (500 MHz, $CDCl_3$) δ 7.52 (d, *J* = 8.0 Hz, 2H), 7.25 – 7.22 (m, 2H), 7.15 – 7.01 (m, 8H), 6.97 (d, *J* = 8.0 Hz, 2H), 6.91 (d, *J* = 7.5 Hz, 2H), 6.86 (d, *J* = 8.0 Hz, 1H), 5.01 (d, *J* = 4.5 Hz, 1H), 4.95 (d, *J* = 4.5 Hz, 1H), 4.66 – 4.53 (m, 1H), 3.97 – 3.86 (m, 1H), 3.52 – 3.38 (m, 1H), 3.18 – 3.07 (m, 1H), 2.40 (s, 3H), 2.25 (s, 3H); ^{13}C NMR (125 MHz, $CDCl_3$) δ 174.0, 144.0, 141.0, 139.2, 137.5, 136.7, 136.2, 134.4, 131.5, 129.7, 129.6, 129.1, 128.7, 128.6, 127.9, 127.6, 127.1, 126.5, 56.2, 54.0, 45.7, 36.4, 21.5, 20.9; IR (neat): 2923, 1698(s), 1597, 1494, 1347, 1165, 1119, 1087, 735, 547; HRESIMS Calcd for $[C_{31}H_{29}NNaO_3S]^+$ (M + Na$^+$) 518.1760, found 518.1759.

5-(4-methoxyphenyl)-6-phenyl-3-tosyl-2,3,5,6-tetrahydrobenzo[*d*]azocin-4(1*H*)-one (2j)

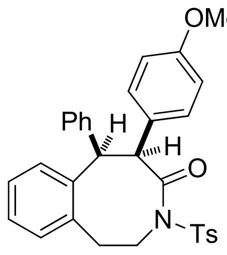

2j

Compound **2j** was prepared in 89% yield (91.1 mg) according to the general procedure (Table 2, entry 10). Pale yellow solid (mp 181-182 °C). ^{1}H NMR (400 MHz, $CDCl_3$) δ 7.48 (d, J = 8.4 Hz, 2H), 7.23 – 7.19 (m, 2H), 7.14 – 7.00 (m, 8H), 6.88 – 6.81 (m, 3H), 6.68 (d, J = 8.4 Hz, 2H), 5.03 (d, J = 5.2 Hz, 1H), 4.92 (d, J = 5.2 Hz, 1H), 4.66 – 4.52 (m, 1H), 3.92 (ddd, J = 15.6 Hz, J = 5.2 Hz, J = 1.2 Hz, 1H), 3.71 (s, 3H), 3.55 – 3.42 (m, 1H), 3.20 – 3.07 (m, 1H), 2.39 (s, 3H); ^{13}C NMR (100 MHz, $CDCl_3$) δ 174.1, 158.5, 144.0, 140.7, 139.1, 137.1, 136.0, 131.5, 131.4, 131.0, 129.8, 129.2, 129.1, 128.5, 127.9, 127.6, 127.1, 126.5, 113.2, 55.4, 55.1, 54.1, 45.5, 36.2, 21.5; IR (neat): 2924, 1697(s), 1512, 1347, 1251, 1165, 1118, 1087, 735, 549; HRESIMS Calcd for $[C_{31}H_{29}NNaO_4S]^+$ (M + Na^+) 534.1710, found 534.1711.

5-(3-bromophenyl)-6-phenyl-3-tosyl-2,3,5,6-tetrahydrobenzo[*d*]azocin-4(1*H*)-one (2k)

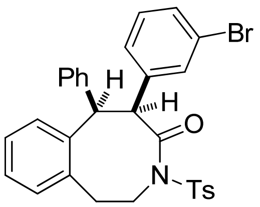

2k

Compound **2k** was prepared in 80% yield (89.7 mg) according to the general procedure (Table 2, entry 11). Pale yellow solid (mp 193-194 °C). ^{1}H NMR (500 MHz, $CDCl_3$) δ 7.45 (d, J = 8.0 Hz, 2H), 7.31 – 7.29 (m, 1H), 7.28 – 7.22 (m, 3H), 7.15 – 7.10 (m, 5H), 7.07 – 7.03 (m, 2H), 7.01 – 6.97 (m, 1H), 6.85 – 6.79 (m, 3H), 5.03 (d, J = 6.0 Hz, 1H), 4.87 (d, J = 6.0 Hz, 1H), 4.73 – 4.63 (m, 1H), 3.92 (ddd, J = 15.5 Hz, J = 6.0 Hz, J = 2.5 Hz, 1H), 3.57 – 3.46 (m, 1H), 3.17 (ddd, J = 15.0 Hz, J = 6.0 Hz, J = 2.5 Hz, 1H), 2.40 (s, 3H); ^{13}C NMR (100 MHz, $CDCl_3$) δ 172.9, 144.1, 140.3, 139.3, 138.3, 136.8, 135.9, 132.9, 131.6, 131.3, 130.2, 129.9, 129.3, 129.1, 128.6, 128.0, 127.8, 127.2, 126. 8, 121.8,

55.0, 54.2, 45.4, 36.0, 21.6; IR (neat): 2921, 1697(s), 1596, 1453, 1345, 1165, 1119, 735, 591; HRESIMS Calcd for $[C_{30}H_{26}BrNNaO_3S]^+$ (M + Na^+) 582.0709, found 582.0706.

6-phenyl-5-(*m*-tolyl)-3-tosyl-2,3,5,6-tetrahydrobenzo[*d*]azocin-4(1*H*)-one (2l)

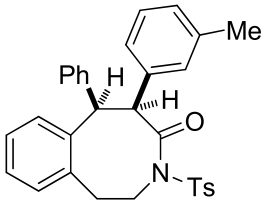

2l

Compound **2l** was prepared in 79% yield (78.3 mg) according to the general procedure (Table 2, entry 12). Pale yellow solid (mp 182-183 °C). ^{1}H NMR (400 MHz, $CDCl_3$) δ 7.51 (d, J = 8.4 Hz, 2H), 7.25 – 7.21 (m, 2H), 7.15 – 6.95 (m, 10H), 6.91 – 6.85 (m, 3H), 5.00 (d, J = 4.8 Hz, 1H), 4.93 (d, J = 4.8 Hz, 1H), 4.71 – 4.59 (m, 1H), 3.92 (ddd, J = 16.0 Hz, J = 5.2 Hz, J = 4.8 Hz, 1H), 3.53 – 3.42 (m, 1H), 3.18 – 3.09 (m, 1H), 2.40 (s, 3H), 2.21 (s, 3H); ^{13}C NMR (125 MHz, $CDCl_3$) δ 173.7, 144.0, 141.0, 139.1, 137.5, 137.4, 137.3, 136.2, 132.0, 131.5, 130.6, 129.7, 129.1, 128.9, 128.6, 127.9, 127.8, 127.6, 127.1, 126.8, 126.5, 56.2, 54.1, 45.6, 36.4, 21.5, 21.3; IR (neat): 2922, 1699(s), 1454, 1347, 1165, 1120, 1088, 913, 734, 671; HRESIMS Calcd for $[C_{31}H_{29}NNaO_3S]^+$ (M + Na^+) 518.1760, found 518.1762.

5-(naphthalen-2-yl)-6-phenyl-3-tosyl-2,3,5,6-tetrahydrobenzo[*d*]azocin-4(1*H*)-one (2m)

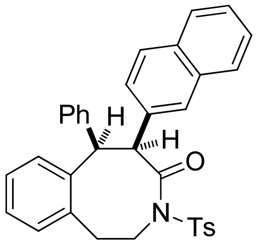

2m

Compound **2m** was prepared in 99% yield (105.3 mg) according to the general procedure (Table 2, entry 13). Pale yellow solid (mp 202-203 °C). ^{1}H NMR (500 MHz, $CDCl_3$) δ 7.73 – 7.69 (m, 1H), 7.67 – 7.62 (m, 2H), 7.61 (d, J = 8.5 Hz, 1H), 7.52 (d, J = 8.5 Hz,

2H), 7.41 – 7.35 (m, 2H), 7.27 (dd, J = 8.5 Hz, J = 1.5 Hz, 1H), 7.24 – 7.21 (m, 2H), 7.14 – 7.09 (m, 3H), 7.08 – 7.03 (m, 1H), 7.02 – 6.96 (m, 2H), 6.93 (d, J = 7.5 Hz, 2H), 6.85 (d, J = 7.5 Hz, 1H), 5.21 (d, J = 5.5 Hz, 1H), 5.05 (d, J = 5.0 Hz, 1H), 4.71 – 4.60 (m, 1H), 3.96 (ddd, J = 15.5 Hz, J = 5.0 Hz, J = 4.0 Hz, 1H), 3.55 – 3.46 (m, 1H), 3.16 (ddd, J = 15.0 Hz, J = 5.0 Hz, J = 4.0 Hz, 1H), 2.37 (s, 3H); ^{13}C NMR (125 MHz, $CDCl_3$) δ 173.7, 144.0, 140.9, 139.0, 137.3, 136.0, 134.8, 132.9, 132.3, 131.5, 131.4, 129.7, 129.1, 128.7, 128.5, 128.0, 127.9, 127.8, 127.7, 127.4, 127.3, 127.1, 126.5, 125.9, 125.8, 56.2, 54.0, 45.6, 36.2, 21.5; IR (neat): 2919, 1633(s), 1449, 1341, 1162, 1115, 1084, 700, 670, 544; HRESIMS Calcd for $[C_{34}H_{29}NNaO_3S]^+$ (M + Na^+) 554.1760, found 554.1761.

6-phenyl-5-(thiophen-2-yl)-3-tosyl-2,3,5,6-tetrahydrobenzo[*d*]azocin-4(1*H*)-one (2n)

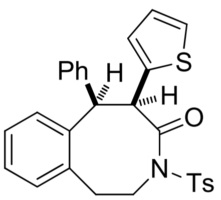

2n

Compound **2n** was prepared in 81% yield (80.0 mg) according to the general procedure (Table 2, entry 14). Pale yellow oil. ^{1}H NMR (400 MHz, $CDCl_3$) δ 7.44 (d, J = 8.4 Hz, 2H), 7.25 (d, J = 4.8 Hz, 2H), 7.15 – 7.09 (m, 4H), 7.09 – 7.02 (m, 3H), 6.88 – 6.81 (m, 3H), 6.81 – 6.75 (m, 2H), 5.43 (d, J = 6.0 Hz, 1H), 4.86 (d, J = 5.6 Hz, 1H), 4.70 – 4.57 (m, 1H), 3.95 (ddd, J = 15.6 Hz, J = 6.0 Hz, J = 3.6 Hz, 1H), 3.58 – 3.46 (m, 1H), 3.23 (ddd, J = 14.4 Hz, J = 6.4 Hz, J = 3.2 Hz, 1H), 2.38 (s, 3H); ^{13}C NMR (100 MHz, $CDCl_3$) δ 172.5, 144.1, 139.9, 138.6, 138.5, 136.9, 135.9, 131.5, 131.3, 130.0, 129.1, 128.6, 127.9, 127.8, 127.2, 127.1, 126.7, 125.7, 125.5, 55.3, 51.4, 45.1, 36.1, 21.5; IR (neat): 2930, 1640(s), 1457, 1396, 1349, 1152, 1086, 736, 680, 559; HRESIMS Calcd for $[C_{28}H_{25}NNaO_3S_2]^+$ (M + Na^+) 510.1168, found 510.1170.

6-phenyl-5-((*E*)-styryl)-3-tosyl-2,3,5,6-tetrahydrobenzo[*d*]azocin-4(1*H*)-one (2o)

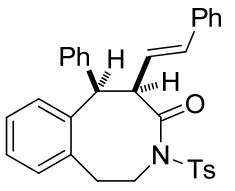

2o

Compound **2o** was prepared in 94% yield (95.4 mg) according to the general procedure (Table 2, entry 15). Pale yellow oil. ^{1}H NMR (500 MHz, $CDCl_3$) δ 7.45 (d, *J* = 8.0 Hz, 2H), 7.23 – 7.15 (m, 7H), 7.15 – 7.02 (m, 8H), 6.78 (d, *J* = 7.5 Hz, 1H), 6.42 (d, *J* = 16.0 Hz, 1H), 6.04 (dd, *J* = 15.5 Hz, *J* = 8.0 Hz, 1H), 4.76 (d, *J* = 6.0 Hz, 1H), 4.62 – 4.53 (m, 1H), 4.52 – 4.41 (m, 1H), 4.08 – 3.98 (m, 1H), 3.57 – 3.46 (m, 1H), 3.26 – 3.16 (m, 1H), 2.37 (s, 3H); ^{13}C NMR (125 MHz, $CDCl_3$) δ 174.2, 144.0, 140.0, 139.4, 136.9, 136.6, 136.1, 132.7, 130.9, 130.6, 130.0, 129.1, 128.4, 128.2, 127.6, 127.1, 126.8, 126.4, 126.3, 54.3, 52.6, 45.0, 35.6, 21.5; IR (neat): 2925, 1692(s), 1494, 1449, 1347, 1165, 1118, 1086, 909, 733, 545; HRESIMS Calcd for $[C_{32}H_{29}NNaO_3S]^+$ (M + Na^+) 530.1760, found 530.1766.

8-methyl-5,6-diphenyl-3-tosyl-2,3,5,6-tetrahydrobenzo[*d*]azocin-4(1*H*)-one (2p)

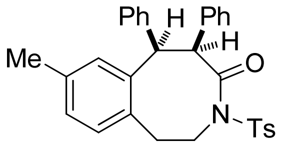

2p

Compound **2p** was prepared in 84% yield (83.3 mg) according to the general procedure (Table 2, entry 16). Pale yellow solid (mp 179-180 °C). ^{1}H NMR (500 MHz, $CDCl_3$) δ 7.53 (d, *J* = 8.5 Hz, 2H), 7.23 – 7.14 (m, 5H), 7.13 – 7.05 (m, 4H), 7.03 – 6.98 (m, 3H), 6.90 (d, *J* = 7.5 Hz, 2H), 6.65 (s, 1H), 5.02 (d, *J* = 5.0 Hz, 1H), 4.90 (d, *J* = 4.5 Hz, 1H), 4.70 – 4.59 (m, 1H), 3.88 (ddd, *J* = 16.0 Hz, *J* = 5.0 Hz, *J* = 4.5 Hz, 1H), 3.46 – 3.37 (m, 1H), 3.11 – 3.03 (m, 1H), 2.39 (s, 3H), 2.18 (s, 3H); ^{13}C NMR (125 MHz, $CDCl_3$) δ 173.7, 143.9, 140.7, 138.8, 137.7, 136.7, 136.1, 134.2, 132.1, 131.5, 129.8, 129.7, 129.0, 128.7, 128.2, 127.9, 127.8, 127.1, 126.4, 56.1, 54.2, 45.9, 35.8, 21.5, 21.0; IR (neat): 2924, 1698(s), 1496, 1454, 1347, 1166, 1118, 1087, 699, 540; HRESIMS Calcd for $[C_{31}H_{29}NNaO_3S]^+$ (M + Na^+) 518.1760, found 518.1760.

N-(2-(hydroxy(phenyl)methyl)-4-methylphenethyl)-2-phenyl-N-tosylacetamide (2p′)

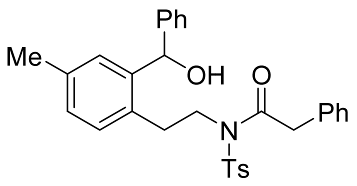

2p′

Pale yellow oil. ^{1}H NMR (400 MHz, $CDCl_3$) δ 7.65 (d, J = 8.4 Hz, 2H), 7.37 – 7.23 (m, 10H), 7.20 – 7.17 (m, 1H), 7.11 (d, J = 7.6 Hz, 1H), 7.06 – 6.99 (m, 3H), 6.14 (d, J = 1.6 Hz, 1H), 4.00 – 3.81 (m, 4H), 3.05 (ddd, J = 15.6 Hz, J = 10.4 Hz, J = 5.6 Hz, 1H), 2.94 (ddd, J = 16.4 Hz, J = 10.4 Hz, J = 6.0 Hz, 1H), 2.75 (d, J = 3.6 Hz, 1H), 2.41 (s, 3H), 2.29 (s, 3H); ^{13}C NMR (100 MHz, $CDCl_3$) δ 171.2, 145.0, 143.5, 141.6, 136.8, 136.5, 133.4, 132.5, 130.8, 129.9, 129.4, 128.7, 128.5, 128.3, 127.5, 127.3, 127.1, 126.8, 72.7, 48.4, 42.8, 32.7, 21.6, 21.1; IR (neat): 3486, 2924, 1694(s), 1495, 1453, 1355, 1161, 1087, 1032, 698, 587; HRESIMS Calcd for $[C_{31}H_{31}NNaO_4S]^+$ (M + Na$^+$) 536.1866, found 536.1865.

8-ethyl-5,6-diphenyl-3-tosyl-2,3,5,6-tetrahydrobenzo[*d*]azocin-4(1*H*)-one (2q)

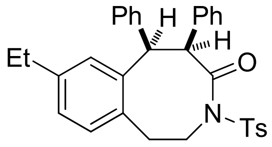

2q

Compound **2q** was prepared in 90% yield (91.8 mg) according to the general procedure (Table 2, entry 17). Pale yellow solid (mp 170-171 °C). ^{1}H NMR (500 MHz, $CDCl_3$) δ 7.54 (d, J = 8.5 Hz, 2H), 7.22 – 7.16 (m, 5H), 7.13 – 7.00 (m, 7H), 6.90 (d, J = 7.5 Hz, 2H), 6.69 (d, J = 1.5 Hz, 1H), 5.04 (d, J = 4.5 Hz, 1H), 4.96 (d, J = 5.0 Hz, 1H), 4.60 (ddd, J = 15.5 Hz, J = 10.0 Hz, J = 5.0 Hz, 1H), 3.93 – 3.86 (m, 1H), 3.42 (ddd, J = 15.0 Hz, J = 10.0 Hz, J = 5.0 Hz, 1H), 3.11 – 3.01 (m, 1H), 2.54 – 2.42 (m, 2H), 2.39 (s, 3H), 1.10 (t, J = 8.0 Hz, 3H); ^{13}C NMR (125 MHz, $CDCl_3$) δ 173.9, 143.9, 143.0, 140.6, 138.9, 137.6, 136.0, 134.5, 131.5, 131.1, 129.9, 129.6, 129.1, 128.6, 127.9, 127.8, 127.1, 126.9, 126.4, 56.5, 54.1, 46.0, 35.9, 28.3, 21.5, 15.3; IR (neat): 2965, 2927, 1643(s), 1455, 1346,

1166, 1118, 731, 548; HRESIMS Calcd for $[C_{32}H_{31}NNaO_3S]^+$ (M + Na^+) 532.1917, found 532.1914.

8-isopropyl-5,6-diphenyl-3-tosyl-2,3,5,6-tetrahydrobenzo[*d*]azocin-4(1*H*)-one (2r)

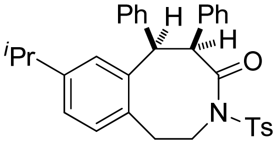

2r

Compound **2r** was prepared in 89% yield (93.3 mg) according to the general procedure (Table 2, entry 18). Pale yellow solid (mp 175-176 °C). ^{1}H NMR (400 MHz, $CDCl_3$) δ 7.57 (d, J = 8.4 Hz, 2H), 7.22 – 7.17 (m, 5H), 7.15 – 7.00 (m, 7H), 6.94 – 6.89 (m, 2H), 6.73 (d, J = 1.6 Hz, 1H), 5.06 (d, J = 4.8 Hz, 1H), 5.01 (d, J = 4.8 Hz, 1H), 4.55 (ddd, J = 15.2 Hz, J = 10.0 Hz, J = 4.8 Hz, 1H), 3.95 – 3.85 (m, 1H), 3.42 (ddd, J = 15.2 Hz, J = 10.0 Hz, J = 5.2 Hz, 1H), 3.10 – 3.02 (m, 1H), 2.79 – 2.66 (m, 1H), 2.39 (s, 3H), 1.11 (d, J = 1.6 Hz, 3H), 1.10 (d, J = 1.6 Hz, 3H); ^{13}C NMR (100 MHz, $CDCl_3$) δ 174.2, 147.6, 143.9, 140.5, 139.1, 137.5, 135.9, 134.7, 131.5, 130.0, 129.8, 129.6, 129.1, 128.6, 127.9, 127.8, 127.1, 126.4, 125.4, 56.9, 53.9, 46.1, 36.0, 33.5, 24.0, 23.4, 21.5; IR (neat): 2957, 1642(s), 1496, 1457, 1347, 1167, 1118, 911, 731, 676; HRESIMS Calcd for $[C_{33}H_{33}NNaO_3S]^+$ (M + Na^+) 546.2073, found 546.2072.

8-methoxy-5,6-diphenyl-3-tosyl-2,3,5,6-tetrahydrobenzo[*d*]azocin-4(1*H*)-one (2s)

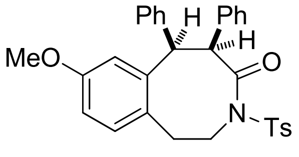

2s

Compound **2s** was prepared in 83% yield (85.0 mg) according to the general procedure except by using 10 mol % of $Zn(OTf)_2$ as catalyst and 5 Å MS (60 mg/0.1 mmol) as additive (Table 2, entry 19). Pale yellow solid (mp 188-189 °C). ^{1}H NMR (400 MHz, $CDCl_3$) δ 7.51 (d, J = 8.4 Hz, 2H), 7.23 – 7.17 (m, 2H), 7.16 – 7.09 (m, 6H), 7.09 – 6.97 (m, 3H), 6.87 (d, J = 7.2 Hz, 2H), 6.73 (dd, J = 8.4 Hz, J = 2.8 Hz, 1H), 6.37 (d, J = 2.8 Hz, 1H), 5.07 (d, J = 5.6 Hz, 1H), 4.88 (d, J = 5.6 Hz, 1H), 4.67 – 4.56 (m, 1H), 3.90

(ddd, J = 15.2 Hz, J = 5.6 Hz, J = 3.6 Hz, 1H), 3.60 (s, 3H), 3.49 – 3.39 (m, 1H), 3.09 (ddd, J = 14.8 Hz, J = 6.0 Hz, J = 3.6 Hz, 1H), 2.39 (s, 3H); ^{13}C NMR (100 MHz, $CDCl_3$) δ 173.5, 158.5, 143.9, 142.0, 138.6, 137.2, 136.0, 132.4, 129.9, 129.8, 129.0, 128.8, 128.6, 127.9, 127.0, 126.6, 117.5, 112.3, 55.4, 54.9, 54.3, 45.8, 35.2, 21.5; IR (neat): 2929, 1698(s), 1494, 1455, 1345, 1165, 1118, 1086, 732, 548; HRESIMS Calcd for $[C_{31}H_{29}NNaO_4S]^+$ (M + Na^+) 534.1710, found 534.1712.

5,6,8-triphenyl-3-tosyl-2,3,5,6-tetrahydrobenzo[*d*]azocin-4(1*H*)-one (2t)

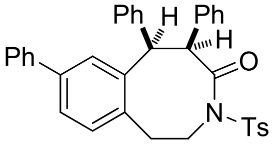

2t

Compound **2t** was prepared in 91% yield (101.6 mg) according to the general procedure (Table 2, entry 20). Pale yellow solid (mp 168-169 °C). ^{1}H NMR (400 MHz, $CDCl_3$) δ 7.51 (d, J = 8.4 Hz, 2H), 7.44 (dd, J = 7.6 Hz, J = 1.6 Hz, 1H), 7.38 – 7.32 (m, 4H), 7.32 – 7.26 (m, 2H), 7.23 – 7.20 (m, 2H), 7.17 – 7.12 (m, 3H), 7.11 – 7.00 (m, 6H), 6.87 (d, J = 7.2 Hz, 2H), 5.13 (d, J = 5.6 Hz, 1H), 4.97 (d, J = 5.6 Hz, 1H), 4.76 – 4.65 (m, 1H), 3.96 (ddd, J = 15.6 Hz, J = 5.6 Hz, J = 3.2 Hz, 1H), 3.60 – 3.49 (m, 1H), 3.19 (ddd, J = 14.4 Hz, J = 5.6 Hz, J = 3.2 Hz, 1H), 2.26 (s, 3H); ^{13}C NMR (100 MHz, $CDCl_3$) δ 173.5, 144.0, 141.1, 140.3, 139.8, 138.5, 137.1, 135.9, 135.8, 132.0, 130.1, 130.0, 129.9, 129.0, 128.7, 128.6, 127.9, 127.3, 127.1, 126.8, 126.6, 126.1, 55.4, 54.4, 45.4, 35.7, 21.4; IR (neat): 2925, 1639(s), 1484, 1349, 1170, 1120, 1088, 763, 701, 567; HRESIMS Calcd for $[C_{36}H_{31}NNaO_3S]^+$ (M + Na^+) 580.1917, found 580.1916.

8-chloro-5,6-diphenyl-3-tosyl-2,3,5,6-tetrahydrobenzo[*d*]azocin-4(1*H*)-one (2u)

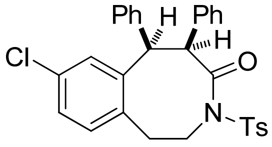

2u

Compound **2u** was prepared in 98% yield (101.1 mg) according to the general procedure (Table 2, entry 21). Pale yellow solid (mp 181-182 °C). ^{1}H NMR (500 MHz, $CDCl_3$) δ

7.50 (d, J = 8.5 Hz, 2H), 7.19 – 7.07 (m, 10H), 7.06 – 6.99 (m, 2H), 6.83 (d, J = 7.5 Hz, 2H), 6.74 (s, 1H), 5.06 (d, J = 6.0 Hz, 1H), 4.83 (d, J = 5.5 Hz, 1H), 4.71 – 4.58 (m, 1H), 3.97 – 3.83 (m, 1H), 3.54 – 3.41 (m, 1H), 3.17 – 3.06 (m, 1H), 2.39 (s, 3H); ^{13}C NMR (125 MHz, $CDCl_3$) δ 173.0, 144.3, 142.5, 137.8, 136.6, 135.6, 135.0, 132.9, 132.6, 131.0, 130.0, 129.7, 129.0, 128.6, 128.0, 127.8, 127.5, 127.1, 126.8, 54.6, 53.9, 45.1, 35.1, 21.5; IR (neat): 2926, 1700(s), 1596, 1496, 1349, 1166, 1087, 736, 700, 536; HRESIMS Calcd for $[C_{30}H_{26}ClNNaO_3S]^+$ (M + Na^+) 538.1214, found 538.1213.

8-bromo-5,6-diphenyl-3-tosyl-2,3,5,6-tetrahydrobenzo[*d*]azocin-4(1*H*)-one (2v)

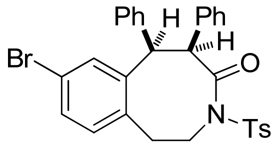

2v

Compound **2v** was prepared in 95% yield (106.5 mg) according to the general procedure (Table 2, entry 22). Pale yellow solid (mp 205-206 °C). ^{1}H NMR (400 MHz, $CDCl_3$) δ 7.51 (d, J = 8.4 Hz, 2H), 7.31 (dd, J = 8.0 Hz, J = 2.0 Hz, 1H), 7.19 – 7.02 (m, 11H), 6.90 (d, J = 1.6 Hz, 1H), 6.83 (d, J = 7.2 Hz, 2H), 5.06 (d, J = 6.0 Hz, 1H), 4.85 (d, J = 6.0 Hz, 1H), 4.71 – 4.59 (m, 1H), 3.92 (ddd, J = 15.6 Hz, J = 6.0 Hz, J = 3.2 Hz, 1H), 3.53 – 3.40 (m, 1H), 3.11 (ddd, J = 14.4 Hz, J = 6.4 Hz, J = 3.2 Hz, 1H), 2.42 (s, 3H); ^{13}C NMR (100 MHz, $CDCl_3$) δ 173.1, 144.3, 142.9, 137.8, 136.6, 135.7, 135.6, 133.9, 132.9, 130.6, 129.9, 129.7, 129.1, 128.7, 128.0, 127.9, 127.2, 126.9, 121.2, 54.8, 53.9, 45.1, 35.3, 21.6; IR (neat): 2922, 1642(s), 1495, 1454, 1347, 1165, 1119, 732, 699, 565; HRESIMS Calcd for $[C_{30}H_{26}BrNNaO_3S]^+$ (M + Na^+) 582.0709, found 582.0709.

9-methyl-5,6-diphenyl-3-tosyl-2,3,5,6-tetrahydrobenzo[*d*]azocin-4(1*H*)-one (2w)

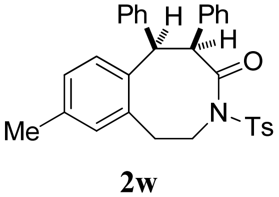

2w

Compound **2w** was prepared in 91% yield (90.2 mg) according to the general procedure (Table 2, entry 23). Pale yellow solid (mp 175-176 °C). ^{1}H NMR (400 MHz, $CDCl_3$) δ

7.49 (d, *J* = 8.4 Hz, 2H), 7.21 – 7.16 (m, 2H), 7.16 – 7.09 (m, 5H), 7.08 – 7.04 (m, 1H), 7.03 – 6.97 (m, 3H), 6.91 – 6.85 (m, 3H), 6.71 (d, *J* = 8.0 Hz, 1H), 5.05 (d, *J* = 5.2 Hz, 1H), 4.90 (d, *J* = 5.2 Hz, 1H), 4.68 – 4.56 (m, 1H), 3.92 (ddd, *J* = 15.6 Hz, *J* = 5.6 Hz, *J* = 4.0 Hz, 1H), 3.50 – 3.39 (m, 1H), 3.09 (ddd, *J* = 14.8 Hz, *J* = 5.6 Hz, *J* = 4.0 Hz, 1H), 2.38 (s, 3H), 2.32 (s, 3H); ^{13}C NMR (100 MHz, $CDCl_3$) δ 173.6, 143.9, 139.1, 137.7, 137.3, 137.2, 136.8, 136.0, 132.2, 131.3, 129.8, 129.7, 129.0, 128.5, 127.9, 127.8, 127.7, 127.0, 126.4, 55.9, 53.7, 45.5, 36.0, 21.5, 20.8; IR (neat): 2923, 1700(s), 1496, 1454, 1347, 1165, 1120, 1087, 701, 552; HRESIMS Calcd for $[C_{31}H_{29}NNaO_3S]^+$ (M + Na^+) 518.1760, found 518.1761.

9-methoxy-5,6-diphenyl-3-tosyl-2,3,5,6-tetrahydrobenzo[*d*]azocin-4(1*H*)-one (2x)

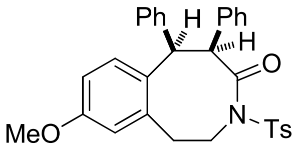

2x

Compound **2x** was prepared in 89% yield (91.0 mg) according to the general procedure (Table 2, entry 24). Pale yellow solid (mp 188-189 °C). ^{1}H NMR (400 MHz, $CDCl_3$) δ 7.54 (d, *J* = 8.4 Hz, 2H), 7.22 – 7.17 (m, 2H), 7.16 – 7.10 (m, 5H), 7.10 – 6.98 (m, 3H), 6.92 (d, *J* = 7.2 Hz, 2H), 6.76 – 6.70 (m, 2H), 6.58 (dd, *J* = 8.4 Hz, *J* = 2.8 Hz, 1H), 5.05 (d, *J* = 5.6 Hz, 1H), 4.90 (d, *J* = 5.2 Hz, 1H), 4.66 – 4.54 (m, 1H), 3.94 (ddd, *J* = 15.6 Hz, *J* = 5.6 Hz, *J* = 3.6 Hz, 1H), 3.78 (s, 3H), 3.50 – 3.39 (m, 1H), 3.08 (ddd, *J* = 14.8 Hz, *J* = 5.6 Hz, *J* = 4.0 Hz, 1H), 2.39 (s, 3H); ^{13}C NMR (100 MHz, $CDCl_3$) δ 173.7, 158.7, 144.0, 139.3, 138.3, 137.3, 136.0, 132.8, 132.4, 129.8, 129.7, 129.0, 128.5, 127.9, 127.0, 126.4, 117.1, 111.6, 56.0, 55.2, 53.1, 45.6, 36.1, 21.5; IR (neat): 2925, 1699(s), 1496, 1455, 1349, 1243, 1166, 1087, 701, 559; HRESIMS Calcd for $[C_{31}H_{29}NNaO_4S]^+$ (M + Na^+) 534.1710, found 534.1708.

9-chloro-5,6-diphenyl-3-tosyl-2,3,5,6-tetrahydrobenzo[*d*]azocin-4(1*H*)-one (2y)

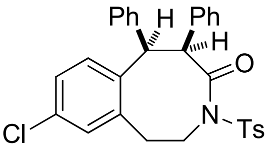

2y

Compound **2y** was prepared in 89% yield (91.8 mg) according to the general procedure (Table 2, entry 25). Pale yellow solid (mp 195-196 °C). ^{1}H NMR (400 MHz, $CDCl_3$) δ 7.57 (d, *J* = 8.4 Hz, 2H), 7.21 – 7.10 (m, 8H), 7.10 – 7.00 (m, 3H), 6.98 (dd, *J* = 8.4 Hz, *J* = 2.4 Hz, 1H), 6.91 (d, *J* = 7.6 Hz, 2H), 6.70 (d, *J* = 8.4 Hz, 1H), 5.06 (d, *J* = 6.0 Hz, 1H), 4.90 (d, *J* = 6.0 Hz, 1H), 4.67 – 4.55 (m, 1H), 3.95 (ddd, *J* = 15.6 Hz, *J* = 6.0 Hz, *J* = 3.6 Hz, 1H), 3.52 – 3.39 (m, 1H), 3.08 (ddd, *J* = 14.8 Hz, *J* = 6.0 Hz, *J* = 3.6 Hz, 1H), 2.39 (s, 3H); ^{13}C NMR (100 MHz, $CDCl_3$) δ 173.2, 144.3, 139.3, 138.5, 138.3, 136.7, 135.6, 133.0, 132.4, 131.0, 129.9, 129.6, 129.1, 128.5, 128.0, 127.9, 127.0, 126.9, 126.7, 55.2, 53.1, 45.3, 35.4, 21.6; IR (neat): 2921, 1638(s), 1452, 1344, 1163, 1111, 1083, 698, 547; HRESIMS Calcd for $[C_{30}H_{26}ClNNaO_3S]^+$ (M + Na^+) 538.1214, found 538.1214.

8,9-dimethyl-5,6-diphenyl-3-tosyl-2,3,5,6-tetrahydrobenzo[*d*]azocin-4(1*H*)-one (2z)

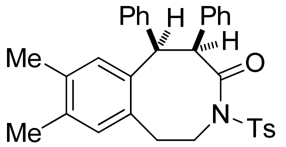

2z

Compound **2z** was prepared in 85% yield (86.7 mg) according to the general procedure (Table 2, entry 26). Pale yellow solid (mp 169-170 °C). ^{1}H NMR (400 MHz, $CDCl_3$) δ 7.52 (d, *J* = 8.4 Hz, 2H), 7.23 – 7.15 (m, 5H), 7.11 (d, *J* = 8.4 Hz, 2H), 7.07 (d, *J* = 7.2 Hz, 1H), 7.03 – 6.95 (m, 3H), 6.89 (d, *J* = 7.6 Hz, 2H), 6.61 (s, 1H), 5.01 (d, *J* = 5.2 Hz, 1H), 4.88 (d, *J* = 4.8 Hz, 1H), 4.64 (ddd, *J* = 15.2 Hz, *J* = 9.6 Hz, *J* = 5.2 Hz, 1H), 3.93 – 3.82 (m, 1H), 3.38 (ddd, *J* = 14.8 Hz, *J* = 9.6 Hz, *J* = 5.2 Hz, 1H), 3.09 – 2.99 (m, 1H), 2.40 (s, 3H), 2.24 (s, 3H), 2.09 (s, 3H); ^{13}C NMR (100 MHz, $CDCl_3$) δ 173.8, 143.9, 139.0, 138.2, 137.8, 136.0, 135.7, 135.1, 134.5, 132.9, 132.7, 129.8, 129.6, 128.9, 128.7, 127.9, 127.8, 127.1, 126.3, 56.3, 53.9, 45.9, 35.8, 21.6, 19.3, 19.1; IR (neat): 2923, 1638(s), 1496, 1453, 1347, 1166, 1114, 1087, 701, 673, 545; HRESIMS Calcd for $[C_{32}H_{31}NNaO_3S]^+$ (M + Na^+) 532.1917, found 532.1916.

9,10-diphenyl-7-tosyl-6,7,9,10-tetrahydro-[1,3]dioxolo[4',5':4,5]benzo[1,2-*d*]azocin-8(5*H*)-one (2aa)

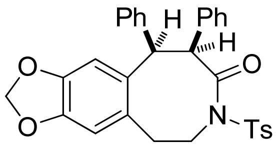

2aa

Compound **2aa** was prepared in 73% yield (76.7 mg) according to the general procedure except by using 10 mol % of $Zn(OTf)_2$ as catalyst and 5 Å MS (60 mg/0.1 mmol) as additive (Table 2, entry 27). Pale yellow solid (mp 201-202 °C). ^{1}H NMR (400 MHz, $CDCl_3$) δ 7.64 (d, J = 8.4 Hz, 2H), 7.23 – 7.12 (m, 7H), 7.10 – 7.03 (m, 3H), 7.01 – 6.96 (m, 2H), 6.62 (s, 1H), 6.29 (s, 1H), 5.91 (d, J = 1.6 Hz, 1H), 5.86 (d, J = 1.6 Hz, 1H), 5.08 (d, J = 6.4 Hz, 1H), 4.93 (d, J = 6.0 Hz, 1H), 4.57 – 4.45 (m, 1H), 3.94 (ddd, J = 15.6 Hz, J = 6.0 Hz, J = 3.6 Hz, 1H), 3.48 – 3.36 (m, 1H), 3.03 (ddd, J = 14.8 Hz, J = 6.0 Hz, J = 3.6 Hz, 1H), 2.40 (s, 3H); ^{13}C NMR (100 MHz, $CDCl_3$) δ 173.9, 146.5, 146.4, 144.1, 139.1, 136.8, 136.0, 134.3, 130.0, 129.9, 129.8, 129.0, 128.6, 128.0, 127.9, 127.0, 126.6, 111.5, 111.1, 101.1, 56.2, 52.9, 46.0, 35.6, 21.6; IR (neat): 2922, 1702(s), 1485, 1455, 1346, 1166, 1112, 1039, 732, 672, 564; HRESIMS Calcd for $[C_{31}H_{27}NNaO_5S]^+$ (M + Na^+) 548.1502, found 548.1501.

5,6-diphenyl-3-tosyl-2,3,5,6-tetrahydronaphtho[2,3-*d*]azocin-4(1*H*)-one (2ab)

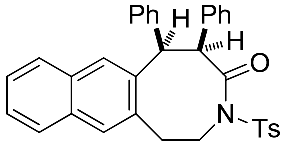

2ab

Compound **2ab** was prepared in 85% yield (90.3 mg) according to the general procedure (Table 2, entry 28). Pale yellow solid (mp 179-180 °C). ^{1}H NMR (400 MHz, $CDCl_3$) δ 7.49 (d, J = 8.4 Hz, 2H), 7.35 – 7.06 (m, 13H), 7.06 – 6.98 (m, 2H), 6.92 – 6.81 (m, 3H), 5.06 (d, J = 5.2 Hz, 1H), 4.94 (d, J = 5.2 Hz, 1H), 4.72 – 4.60 (m, 1H), 3.94 (ddd, J = 15.6 Hz, J = 5.6 Hz, J = 4.4 Hz, 1H), 3.56 – 3.43 (m, 1H), 3.16 (ddd, J = 14.8 Hz, J = 5.6 Hz, J = 4.0 Hz, 1H), 2.40 (s, 3H); ^{13}C NMR (100 MHz, $CDCl_3$) δ 173.6, 144.0, 140.8, 138.9, 137.3, 137.1, 136.1, 131.5, 131.4, 129.9, 129.8, 129.1, 128.6, 127.9(2), 127.9(0), 127.7, 127.2, 127.1, 126.5, 55.8, 54.2, 45.5, 36.2, 21.6; IR (neat): 2924, 2853, 1698(s),

1454, 1346, 1165, 1119, 911, 733, 700, 543; HRESIMS Calcd for $[C_{34}H_{30}NNaO_3S]^{2+}$ (M + H^+ + Na^+) 555.1833, found 555.2675.

6-methyl-5-phenyl-3-tosyl-2,3,5,6-tetrahydrobenzo[*d*]azocin-4(1*H*)-one (2ac)

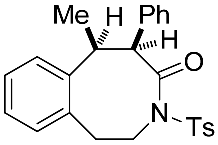

2ac

Compound **2ac** was prepared in 81% yield (67.9 mg) according to the general procedure except at 100 °C for 60 h (Table 2, entry 29). Pale yellow solid (mp 167-168 °C). ^{1}H NMR (400 MHz, $CDCl_3$) δ 7.72 (d, *J* = 8.4 Hz, 2H), 7.31 – 7.25 (m, 3H), 7.22 – 7.08 (m, 6H), 7.08 – 7.01 (m, 2H), 4.57 – 4.43 (m, 1H), 4.39 (d, *J* = 6.8 Hz, 1H), 4.21 – 4.08 (m, 1H), 4.07 – 3.95 (m, 1H), 3.36 (dt, *J* = 15.2 Hz, *J* = 4.8 Hz, 1H), 3.27 – 3.13 (m, 1H), 2.35 (s, 3H), 1.03 (d, *J* = 7.2 Hz, 3H); ^{13}C NMR (100 MHz, $CDCl_3$) δ 173.5, 144.2, 141.2, 136.3, 136.2, 136.1, 131.9, 130.7, 129.2, 128.5, 127.6, 127.5, 127.3, 127.2, 58.8, 46.0, 38.6, 36.2, 21.5, 18.0; IR (neat): 2928, 2853, 1641(s), 1457, 1354, 1170, 1113, 739, 584; HRESIMS Calcd for $[C_{25}H_{25}NNaO_3S]^+$ (M + Na^+) 442.1447, found 442.1449.

6-ethyl-5-phenyl-3-tosyl-2,3,5,6-tetrahydrobenzo[*d*]azocin-4(1*H*)-one (2ad)

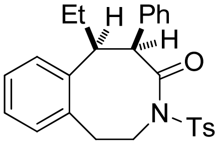

2ad

Compound **2ad** was prepared in 63% yield (54.6 mg) according to the general procedure except at 100 °C for 60 h (Table 2, entry 30). Pale yellow solid (mp 160-161 °C). ^{1}H NMR (400 MHz, $CDCl_3$) δ 7.73 (d, *J* = 6.8 Hz, 2H), 7.30 – 7.25 (m, 3H), 7.23 – 7.16 (m, 3H), 7.15 – 7.04 (m, 4H), 6.99 (d, *J* = 7.6 Hz, 1H), 4.67 – 4.25 (m, 2H), 4.22 – 4.04 (m, 1H), 3.91 – 3.47 (m, 1H), 3.41 – 3.28 (m, 1H), 3.26 – 3.06 (m, 1H), 2.36 (s, 3H), 1.67 – 1.34 (m, 2H), 0.71 (t, *J* = 6.8 Hz, 3H); ^{13}C NMR (100 MHz, $CDCl_3$) δ 173.9, 144.2, 139.2, 137.0, 136.3, 132.0, 130.9, 129.2, 128.5, 127.6, 127.3, 127.2, 127.1, 58.8, 46.3, 36.3, 24.9,

21.5, 12.5; IR (neat): 2967, 2925, 1639(s), 1454, 1347, 1167, 1118, 706, 669, 549; HRESIMS Calcd for $[C_{26}H_{27}NNaO_3S]^+$ (M + Na^+) 456.1604, found 456.1606.

5-methyl-6-phenyl-3-tosyl-2,3,5,6-tetrahydrobenzo[*d*]azocin-4(1*H*)-one (2ae)

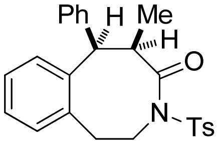

2ae

Compound **2ae** was prepared in 35% yield (29.4 mg) according to the general procedure except at 100 °C for 4 h (Table 2, entry 31). Pale yellow oil. ^{1}H NMR (400 MHz, $CDCl_3$) δ 7.38 (d, J = 8.0 Hz, 2H), 7.25 – 7.17 (m, 5H), 7.14 – 7.05 (m, 3H), 7.04 – 6.95 (m, 2H), 6.72 (d, J = 8.0 Hz, 1H), 4.66 (d, J = 6.4 Hz, 1H), 4.48 (ddd, J = 16.0 Hz, J = 9.6 Hz, J = 6.4 Hz, 1H), 4.00 – 3.91 (m, 1H), 3.85 (ddd, J = 15.2 Hz, J = 6.4 Hz, J = 3.2 Hz, 1H), 3.44 (ddd, J = 16.4 Hz, J = 10.0 Hz, J = 6.4 Hz, 1H), 3.10 (ddd, J = 14.4 Hz, J = 6.8 Hz, J = 3.2 Hz, 1H), 2.37 (s, 3H), 1.04 (d, J = 6.8 Hz, 3H); ^{13}C NMR (100 MHz, $CDCl_3$) δ 176.8, 143.9, 140.3, 139.0, 136.7, 136.1, 131.1, 130.6, 129.7, 129.1, 128.3, 128.1, 127.5, 126.9, 126.6, 51.3, 45.3, 44.3, 35.5, 21.5, 15.2; IR (neat): 2925, 1633(s), 1494, 1454, 1336, 1164, 1124, 1088, 746, 670, 544; HRESIMS Calcd for $[C_{25}H_{25}NNaO_3S]^+$ (M + Na^+) 442.1447, found 442.1448.

5-phenyl-6-(1-phenylvinyl)-3-tosyl-2,3,5,6-tetrahydrobenzo[*d*]azocin-4(1*H*)-one (2af)

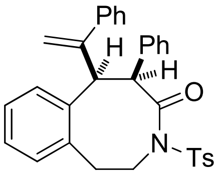

2af

Compound **2af** was prepared in 76% yield (77.2 mg) according to the general procedure (Eq. 1, entry 1). Pale yellow solid (mp 181-182 °C). ^{1}H NMR (400 MHz, $CDCl_3$) δ 7.69 (d, J = 8.4 Hz, 2H), 7.29 – 7.26 (m, 1H), 7.23 – 7.06 (m, 13H), 7.00 (dd, J = 7.6 Hz, J = 1.6 Hz, 2H), 5.32 (s, 1H), 5.25 – 5.20 (m, 2H), 5.07 (d, J = 5.2 Hz, 1H), 4.15 – 4.04 (m, 1H), 4.02 – 3.92 (m, 1H), 3.41 (ddd, J = 14.8 Hz, J = 9.2 Hz, J = 4.8 Hz, 1H), 3.13 – 3.05 (m, 1H), 2.38 (s, 3H); ^{13}C NMR (125 MHz, $CDCl_3$) δ 175.8, 147.4, 144.3, 143.0, 137.9,

137.6, 135.9, 131.3, 131.2, 130.6, 129.4, 128.3, 128.1, 127.7, 127.6, 127.3, 127.2, 126.7, 126.6, 118.2, 58.6, 49.4, 47.0, 36.3, 21.5; IR (neat): 2950, 2844, 1613(s), 1594, 1495, 1366, 1187, 1167, 1024, 833, 572, 531; HRESIMS Calcd for $[C_{32}H_{29}NNaO_3S]^+$ (M + Na^+) 530.1760, found 530.1761.

Methyl-2-(4-oxo-5-phenyl-3-tosyl-1,2,3,4,5,6-hexahydrobenzo[*d*]azocin-6-yl)acrylate (2ag)

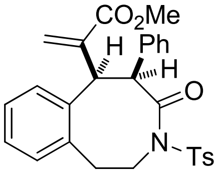

2ag

Compound **2ag** was prepared in 66% yield (64.6 mg) according to the general procedure (Eq. 1, entry 2). Pale yellow solid (mp 179-180 °C). ^{1}H NMR (500 MHz, $CDCl_3$) δ 7.64 (d, J = 8.0 Hz, 2H), 7.30 – 7.20 (m, 6H), 7.18 – 7.10 (m, 5H), 6.11 (s, 1H), 5.58 (s, 1H), 5.28 (d, J = 7.5 Hz, 1H), 4.96 (d, J = 7.5 Hz, 1H), 4.46 – 4.36 (m, 1H), 4.25 – 4.15 (m, 1H), 3.60 (s, 3H), 3.53 – 3.44 (m, 1H), 3.22 – 3.13 (m, 1H), 2.35 (s, 3H); ^{13}C NMR (125 MHz, $CDCl_3$) δ 173.4, 167.8, 144.1, 138.9, 137.1, 136.6, 136.1, 135.6, 132.1, 130.8, 129.5, 129.2, 128.3, 127.8, 127.7, 127.4, 126.9(3), 126.9(0), 56.5, 52.0, 46.0, 43.9, 35.7, 21.5; IR (neat): 2951, 2923, 1713(s), 1448, 1367, 1273, 1151, 1074, 755, 686, 565; HRESIMS Calcd for $[C_{28}H_{27}NNaO_5S]^+$ (M + Na^+) 512.1502, found 512.1502.

5-phenyl-3-tosyl-6-vinyl-2,3,5,6-tetrahydrobenzo[*d*]azocin-4(1*H*)-one (2ah)

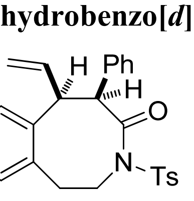

2ah

Compound **2ah** was prepared in 61% yield (52.6 mg) according to the general procedure (Eq. 1, entry 3). Pale yellow oil. ^{1}H NMR (400 MHz, $CDCl_3$) δ 7.79 (d, J = 8.4 Hz, 2H), 7.31 – 7.12 (m, 10H), 7.05 – 7.00 (m, 1H), 5.88 (ddd, J = 17.2 Hz, J = 10.4 Hz, J = 6.8 Hz, 1H), 4.95 (d, J = 10.0 Hz, 1H), 4.91 (d, J = 17.2 Hz, 1H), 4.71 – 4.59 (m, 1H), 4.42 (d, J = 4.8 Hz, 1H), 4.25 (t, J = 5.6 Hz, 1H), 4.06 – 3.96 (m, 1H), 3.30 (t, J = 5.2 Hz, 2H),

2.38 (s, 3H); ^{13}C NMR (100 MHz, $CDCl_3$) δ 172.7, 144.3, 140.4, 138.1, 137.0, 136.2, 136.0, 132.1, 130.1, 129.8, 129.1, 128.8, 128.7, 128.1, 127.5, 127.4, 117.4, 57.5, 53.0, 46.0, 36.7, 21.5; IR (neat): 2920, 1693(s), 1453, 1351, 1166, 1116, 756, 738, 701, 550; HRESIMS Calcd for $[C_{26}H_{25}NNaO_3S]^+$ (M + Na^+) 454.1447, found 454.1447.

1,2-diphenyl-4-tosyl-1,2,4,5,6,7-hexahydro-3*H*-benzo[*e*]azonin-3-one (2ai)

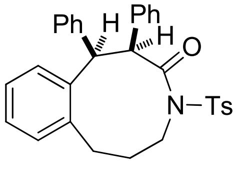

2ai

Compound **2ai** was prepared in 46% yield (45.6 mg) according to the general procedure except by using 10 mol % of $Zn(OTf)_2$ as catalyst and 5 Å MS (60 mg/0.1 mmol) as additive (Eq. 2). Pale yellow solid (mp 174-175 °C). ^{1}H NMR (400 MHz, $CDCl_3$) δ 7.66 (d, J = 8.0 Hz, 2H), 7.40 – 7.26 (m, 2H), 7.23 – 7.15 (m, 3H), 7.15 – 7.00 (m, 9H), 6.95 – 6.82 (m, 1H), 6.80 – 6.56 (m, 1H), 5.90 – 5.26 (m, 1H), 5.24 – 4.92 (m, 1H), 4.50 – 4.05 (m, 1H), 4.03 – 3.65 (m, 1H), 3.35 – 3.06 (m, 1H), 3.05 – 2.68 (m, 1H), 2.39 (s, 3H), 2.38 – 2.17 (m, 2H); ^{13}C NMR (100 MHz, $CDCl_3$) δ 172.9, 144.0, 139.9, 139.1, 136.2, 130.8, 130.5, 129.2, 129.0, 128.4, 128.0, 127.8, 127.5, 126.7, 126.3, 126.0, 52.5, 46.5, 33.0, 30.1, 21.5; IR (neat): 2924, 1698(s), 1495, 1448, 1349, 1167, 1119, 1086, 738, 699, 597; HRESIMS Calcd for $[C_{31}H_{29}NNaO_3S]^+$ (M + Na^+) 518.1760, found 518.1759.

5,6-diphenyl-3,11-ditosyl-1,2,3,5,6,11-hexahydro-4*H*-azocino[4,5-*b*]indol-4-one (2aj)

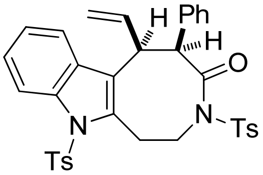

2aj

Compound **2aj** was prepared in 55% yield (68.7 mg) according to the general procedure except by using 20 mol % of $HNTf_2$ as catalyst and 5 Å MS (60 mg/0.1 mmol) as additive (Eq. 3, entry 1). Yellow solid (mp 174-175 °C). ^{1}H NMR (500 MHz, $CDCl_3$) δ 8.13 (d, J = 8.5 Hz, 1H), 7.61 (d, J = 8.0 Hz, 2H), 7.57 (d, J = 8.5 Hz, 2H), 7.47 (d, J =

7.5 Hz, 2H), 7.39 – 7.34 (m, 2H), 7.33 – 7.27 (m, 2H), 7.26 – 7.24 (m, 1H), 7.23 – 7.17 (m, 3H), 7.01 (d, *J* = 8.0 Hz, 2H), 6.11 (ddd, *J* = 17.0 Hz, *J* = 10.5 Hz, *J* = 5.5 Hz, 1H), 4.97 – 4.90 (m, 1H), 4.62 – 4.51 (m, 2H), 4.32 – 4.21 (m, 2H), 3.93 (ddd, *J* = 14.5 Hz, *J* = 10.5 Hz, *J* = 3.5 Hz, 1H), 3.76 – 3.68 (m, 1H), 3.06 (ddd, *J* = 16.5 Hz, *J* = 10.5 Hz, *J* = 4.0 Hz, 1H), 2.35 (s, 3H), 2.27 (s, 3H); ^{13}C NMR (125 MHz, $CDCl_3$) δ 175.0, 145.1, 144.2, 137.3, 136.3, 136.2, 135.6, 134.1, 133.0, 130.0, 129.4, 128.8, 128.6, 127.6, 126.1, 124.7, 123.4, 122.5, 117.4, 117.3, 115.2, 58.8, 45.2, 44.0, 26.6, 21.6, 21.5; IR (neat): 2925, 1639(s), 1494, 1452, 1357, 1222, 1172, 1088, 671, 547; HRESIMS Calcd for $[C_{35}H_{32}N_2NaO_5S_2]^+$ (M + Na^+) 647.1645, found 647.1646.

***tert*-butyl-4-oxo-5,6-diphenyl-3-tosyl-1,2,3,4,5,6-hexahydro-11*H*-azocino[4,5-*b*]indole-11-carboxylate (2ak)**

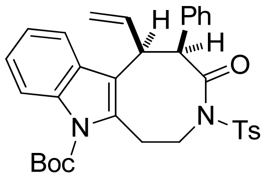

2ak

Compound **2ak** was prepared in 41% yield (46.8 mg) according to the general procedure except by using 20 mol % of $HNTf_2$ as catalyst and 5 Å MS (60 mg/0.1 mmol) as additive (Eq. 3, entry 2). Pale yellow solid (mp 170-171 °C). ^{1}H NMR (500 MHz, $CDCl_3$) δ 7.90 (d, *J* = 8.5 Hz, 1H), 7.58 (d, *J* = 7.5 Hz, 2H), 7.47 (d, *J* = 8.0 Hz, 2H), 7.42 – 7.36 (m, 2H), 7.36 – 7.31 (m, 2H), 7.25 – 7.22 (m, 1H), 7.22 – 7.16 (m, 1H), 6.84 (d, *J* = 8.0 Hz, 2H), 6.22 (ddd, *J* = 17.0 Hz, *J* = 10.5 Hz, *J* = 5.5 Hz, 1H), 5.06 – 4.99 (m, 1H), 4.87 – 4.79 (m, 1H), 4.73 (d, *J* = 4.5 Hz, 1H), 4.41 – 4.34 (m, 1H), 4.29 – 4.21 (m, 1H), 3.92 (ddd, *J* = 15.0 Hz, *J* = 11.0 Hz, *J* = 4.0 Hz, 1H), 3.66 – 3.56 (m, 1H), 3.11 (ddd, *J* = 15.5 Hz, *J* = 11.0 Hz, *J* = 4.5 Hz, 1H), 2.21 (s, 3H), 1.73 (s, 9H); ^{13}C NMR (125 MHz, $CDCl_3$) δ 175.9, 150.2, 143.9, 137.1, 135.6, 135.3, 134.0, 133.2, 129.1, 128.9, 128.8, 128.5, 127.5, 127.2, 124.1, 122.6, 120.5, 117.5, 116.9, 115.4, 84.5, 59.0, 44.9, 44.0, 28.3, 26.8, 21.6; IR (neat): 2925, 1639(s), 1494, 1454, 1349, 1255, 1140, 1035, 671, 591; HRESIMS Calcd for $[C_{33}H_{34}N_2NaO_5S]^+$ (M + Na^+) 593.2081, found 593.2096.

5-phenyl-3-tosyl-6-vinyl-2,3,5,6-tetrahydrobenzo[4,5]thieno[3,2-*d*]azocin-4(1*H*)-one (2al)

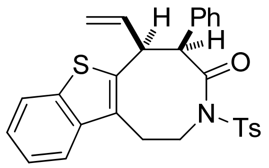

2al

Compound **2al** was prepared in 53% yield (51.7 mg) according to the general procedure except by using 20 mol % of $HNTf_2$ as catalyst and 5 Å MS (60 mg/0.1 mmol) as additive (Eq. 3, entry 3). Pale yellow solid (mp 161-162 °C). ^{1}H NMR (400 MHz, $CDCl_3$) δ 7.75 – 7.64 (m, 2H), 7.49 – 7.38 (m, 5H), 7.37 – 7.27 (m, 4H), 6.90 (d, *J* = 8.4 Hz, 2H), 5.93 (ddd, *J* = 18.0 Hz, *J* = 10.0 Hz, *J* = 8.0 Hz, 1H), 5.07 (d, *J* = 10.4 Hz, 1H), 4.96 (d, *J* = 16.8 Hz, 1H), 4.88 (d, *J* = 5.2 Hz, 1H), 4.73 – 4.61 (m, 1H), 4.29 (dd, *J* = 8.0 Hz, *J* = 5.2 Hz, 1H), 4.08 – 3.99 (m, 1H), 3.53 – 3.36 (m, 2H), 2.29 (s, 3H); ^{13}C NMR (100 MHz, $CDCl_3$) δ 172.8, 144.0, 141.1, 140.6, 138.5, 136.3, 135.5, 134.3, 129.7, 128.9, 128.3, 128.2, 127.6, 127.4, 124.4, 124.2, 122.2, 120.9, 119.2, 56.0, 49.4, 44.7, 26.9, 21.5; IR (neat): 2924, 1634(s), 1455, 1348, 1166, 1110, 1087, 733, 671, 544; HRESIMS Calcd for $[C_{28}H_{25}NNaO_3S_2]^+$ (M + Na^+) 510.1168, found 510.1173.

4,5-diphenyl-7-tosyl-4,7,8,9-tetrahydrothieno[2,3-*d*]azocin-6(5*H*)-one (2am)

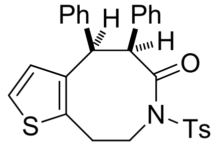

2am

Compound **2am** was prepared in 51% yield (49.8 mg) according to the general procedure except by using 20 mol % of $HNTf_2$ as catalyst and 5 Å MS (60 mg/0.1 mmol) as additive (Eq. 3, entry 4). Pale yellow solid (mp 162-163 °C). ^{1}H NMR (500 MHz, $CDCl_3$) δ 7.58 (d, *J* = 8.5 Hz, 2H), 7.20 (d, *J* = 8.0 Hz, 2H), 7.19 – 7.15 (m, 1H), 7.15 – 7.10 (m, 3H), 7.02 (d, *J* = 7.5 Hz, 2H), 7.00 – 6.95 (m, 3H), 6.42 (d, *J* = 7.5 Hz, 2H), 6.39 (d, *J* = 5.5 Hz, 1H), 5.10 (d, *J* = 5.5 Hz, 1H), 4.66 (d, *J* = 5.0 Hz, 1H), 4.65 – 4.58 (m, 1H), 3.94 (ddd, *J* = 15.5 Hz, *J* = 7.0 Hz, *J* = 4.0 Hz, 1H), 3.74 – 3.62 (m, 1H), 3.42 (ddd, *J* = 16.0 Hz, *J* = 7.0 Hz, *J* = 3.5 Hz, 1H), 2.42 (s, 3H); ^{13}C NMR (125 MHz, $CDCl_3$) δ 173.4, 144.2,

138.7, 138.5, 136.0, 135.8, 133.1, 132.2, 130.5, 130.4, 129.2, 128.9, 127.6, 127.3(9), 127.3(6), 127.0, 121.3, 55.0, 52.4, 44.6, 29.5, 21.6; IR (neat): 2923, 1696(s), 1596, 1453, 1345, 1166, 1120, 1087, 732, 701, 540; HRESIMS Calcd for $[C_{28}H_{25}NNaO_3S_2]^+$ (M + Na^+) 510.1168, found 510.1167.

(*E*)-3-benzylidene-5-phenyl-2-tosyl-2,3,5,6-tetrahydro-1*H*-benzo[*e*][1,3]oxazocine (6ao)

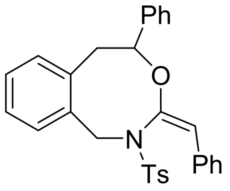

6ao

Compound **6ao** was prepared in 92% yield (88.6 mg) according to the general procedure. Yellow oil. ^{1}H NMR (400 MHz, $CDCl_3$) δ 7.82 (d, *J* = 8.0 Hz, 2H), 7.53 – 7.47 (m, 1H), 7.34 – 7.28 (m, 7H), 7.19 – 7.14 (m, 4H), 7.10 (d, *J* = 7.2 Hz, 1H), 7.06 – 7.00 (m, 3H), 5.94 (s, 1H), 4.84 – 4.73 (m, 2H), 4.35 (d, *J* = 11.2 Hz, 1H), 3.05 (dd, *J* = 14.4 Hz, *J* = 10.0 Hz, 1H), 2.82 (d, *J* = 14.0 Hz, 1H), 2.48 (s, 3H); ^{13}C NMR (100 MHz, $CDCl_3$) δ 145.9, 143.6, 141.1, 138.1, 135.7, 133.8, 133.0, 132.1, 130.4, 129.3, 129.1, 128.5, 128.2, 128.1, 127.8, 127.7, 127.5, 126.9, 125.5, 116.9, 88.1, 49.1, 44.6, 21.6; IR (neat): 2924, 2854, 1495, 1446, 1354, 1161, 1086, 755, 689, 579; HRESIMS Calcd for $[C_{30}H_{27}NNaO_3S]^+$ (M + Na^+) 504.1604, found 504.1609.

(*E*)-2-phenyl-*N*-(5-phenylpent-4-en-1-yl)-*N*-tosylacetamide (2ap′)

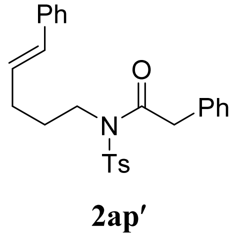

2ap′

Compound **2ap′** was prepared in 90% yield (78.0 mg) according to the general procedure. Pale yellow oil. ^{1}H NMR (400 MHz, $CDCl_3$) δ 7.70 (d, *J* = 8.4 Hz, 2H), 7.34 – 7.17 (m, 10H), 7.09 – 7.03 (m, 2H), 6.38 (d, *J* = 15.6 Hz, 1H), 6.16 (dt, *J* = 15.6 Hz, *J* = 6.8 Hz,

1H), 3.96 (s, 2H), 3.84 – 3.75 (m, 2H), 2.40 (s, 3H), 2.27 – 2.18 (m, 2H), 1.92 – 1.82 (m, 2H); ^{13}C NMR (100 MHz, $CDCl_3$) δ 170.9, 144.8, 137.4, 136.6, 133.4, 130.8, 129.8, 129.2, 128.9, 128.5, 128.4, 127.5, 127.1, 127.0, 125.9, 46.7, 43.0, 30.1, 29.1, 21.5; IR (neat): 3029, 2955, 1693(s), 1495, 1454, 1354, 1164, 1087, 725, 694, 585; HRESIMS Calcd for $[C_{26}H_{27}NNaO_3S]^+$ (M + Na^+) 456.1604, found 456.1604.

N-(2-(methoxy(phenyl)methyl)phenethyl)-2-phenyl-N-tosylacetamide (2aq′)

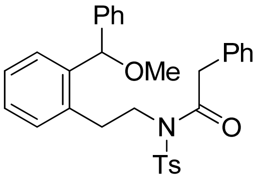

2aq′

Compound **2aq′** was prepared in 78% yield (80.2 mg) according to the general procedure. Yellow oil. ^{1}H NMR (400 MHz, $CDCl_3$) δ 7.67 (d, *J* = 8.4 Hz, 2H), 7.41 (d, *J* = 6.4 Hz, 1H), 7.36 – 7.22 (m, 13H), 7.09 – 7.04 (m, 2H), 5.64 (s, 1H), 3.97 – 3.78 (m, 4H), 3.40 (s, 3H), 3.12 – 2.94 (m, 2H), 2.42 (s, 3H); ^{13}C NMR (100 MHz, $CDCl_3$) δ 171.0, 144.9, 141.2, 139.8, 136.6, 136.1, 133.5, 130.7, 129.9, 129.4, 128.5, 128.4, 127.9, 127.7, 127.6, 127.5, 127.1(2), 127.0(9), 81.9, 57.1, 48.3, 42.8, 33.0, 21.6; IR (neat): 2920, 1693(s), 1494, 1453, 1355, 1160, 1087, 725, 698, 577; HRESIMS Calcd for $[C_{31}H_{31}NNaO_4S]^+$ (M + Na^+) 536.1866, found 536.1866.

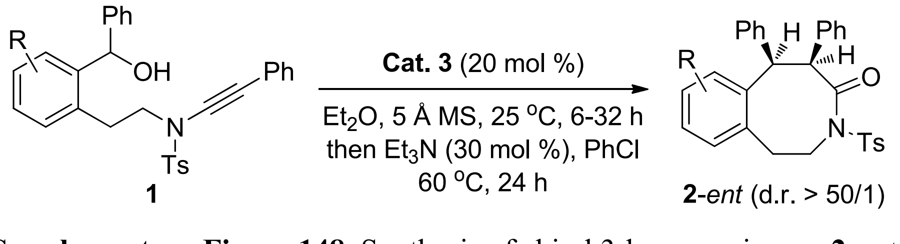

Supplementary Figure 148. Synthesis of chiral 3-benzazocinones **2**-*ent*.

General procedure for the synthesis of chiral 3-benzazocinones 2-*ent*:

To a mixture of the ynamide **1** (0.1 mmol) and 5 Å MS (60 mg) in Et_2O (2 mL) at room temperature, **Cat. 3** (0.02 mmol, 17.6 mg) was added during stiring. Then, the reaction mixture was stirred at 25 °C and the progress of the reaction was monitored by TLC. After the corresponding reaction time (6-32 h), Et_3N (0.03 mmol, 4.2 μL) and PhCl (1 mL) was added to the reaction mixure to quench the **Cat. 3**. The resulting reaction solution was stirred at 60 °C for another 24 h. The mixture was concentrated and the residue was purified by chromatography on silica gel (eluent: hexanes/ethyl acetate) to afford the desired chiral 3-benzazocinone **2**-*ent*.

(5*S*,6*R*)-8-methyl-5,6-diphenyl-3-tosyl-2,3,5,6-tetrahydrobenzo[*d*]azocin-4(1*H*)-one (2p-*ent*)

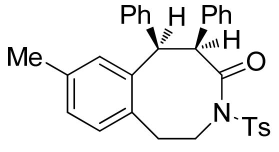

2p-*ent*

Compound **2p**-*ent* was prepared in 42% yield (20.8 mg) according to the general procedure (Table 4, entry 1). $[\alpha]_D^{20}$ = -100.8° (c = 1.0, $CHCl_3$). 95:5 e.r. (determined by HPLC: Chiralcel AS-H Column, 20/80 *i*-PrOH/hexane, 1.0 mL/min, 235 nm, 25 °C; t_R = 13.41 min (major), 19.71 min (minor)).

(5*S*,6*R*)-8-isopropyl-5,6-diphenyl-3-tosyl-2,3,5,6-tetrahydrobenzo[*d*]azocin-4(1*H*)-one (2r-*ent*)

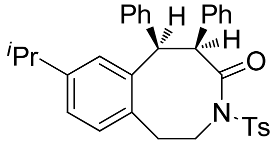

2r-*ent*

Compound **2r**-*ent* was prepared in 40% yield (21.0 mg) according to the general procedure (Table 4, entry 2). $[\alpha]_D^{20}$ = -86.3° (c = 1.0, $CHCl_3$). 93.5:6.5 e.r. (determined by HPLC: Chiralcel AS-H Column, 15/85 *i*-PrOH/hexane, 1.2 mL/min, 235 nm, 25 °C; t_R = 9.12 min (major), 17.33 min (minor)).

(5*S*,6*R*)-5,6,8-triphenyl-3-tosyl-2,3,5,6-tetrahydrobenzo[*d*]azocin-4(1*H*)-one (2t-*ent*)

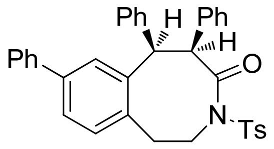

2t-*ent*

Compound **2t**-*ent* was prepared in 50% yield (27.9 mg) according to the general procedure (Table 4, entry 3). $[\alpha]_D^{20}$ = -90.0° (c = 1.0, $CHCl_3$). 92.5:7.5 e.r. (determined by HPLC: Chiralcel AS-H Column, 10/90 *i*-PrOH/hexane, 2.0 mL/min, 235 nm, 25 °C; t_R = 11.79 min (major), 21.63 min (minor)).

(5*S*,6*R*)-9-methyl-5,6-diphenyl-3-tosyl-2,3,5,6-tetrahydrobenzo[*d*]azocin-4(1*H*)-one (2w-*ent*)

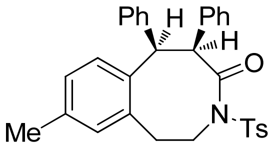

2w-*ent*

Compound **2w**-*ent* was prepared in 51% yield (25.3 mg) according to the general procedure (Table 4, entry 4). $[\alpha]_D^{20}$ = -83.2° (c = 1.0, $CHCl_3$). 90:10 e.r. (determined by HPLC: Chiralcel AS-H Column, 8/92 *i*-PrOH/hexane, 1.0 mL/min, 235 nm, 25 °C; t_R = 20.43 min (major), 29.99 min (minor)).

(5*S*,6*R*)-8,9-dimethyl-5,6-diphenyl-3-tosyl-2,3,5,6-tetrahydrobenzo[*d*]azocin-4(1*H*)-one (2z-*ent*)

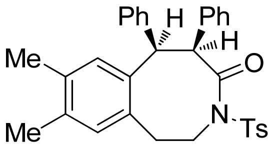

2z-*ent*

Compound **2z**-*ent* was prepared in 46% yield (23.4 mg) according to the general procedure (Table 4, entry 5). $[\alpha]_D^{20}$ = -115.9° (c = 1.0, $CHCl_3$). 93:7 e.r. (determined by HPLC: Chiralcel AS-H Column, 5/95 *i*-PrOH/hexane, 1.3 mL/min, 235 nm, 25 °C; t_R = 22.45 min (major), 32.57 min (minor)).

(5*S*,6*R*)-5,6-diphenyl-3-tosyl-2,3,5,6-tetrahydronaphtho[2,3-*d*]azocin-4(1*H*)-one (2ab-*ent*)

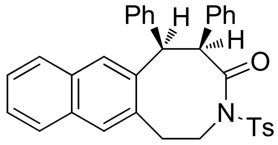

2ab-*ent*

Compound **2ab**-*ent* was prepared in 44% yield (23.4 mg) according to the general procedure (Table 4, entry 6). $[\alpha]_D^{20}$ = -110.6° (c = 1.0, $CHCl_3$). 96:4 e.r. (determined by HPLC: Chiralcel AS-H Column, 5/95 *i*-PrOH/hexane, 2.5 mL/min, 235 nm, 25 °C; t_R = 14.93 min (major), 22.27 min (minor)).

(5*S*,6*R*)-5,6-diphenyl-3-tosyl-2,3,5,6-tetrahydrobenzo[*d*]azocin-4(1*H*)-one (2a-*ent*)

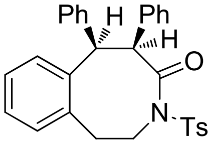

2a-*ent*

Compound **2a**-*ent* was prepared in 41% yield (19.8 mg) according to the general procedure (Table 4, entry 7). $[\alpha]_D^{20}$ = -74.3° (c = 1.0, $CHCl_3$). 89:11 e.r. (determined by HPLC: Chiralcel AS-H Column, 15/85 *i*-PrOH/hexane, 1.0 mL/min, 235 nm, 25 °C; t_R = 20.13 min (major), 28.29 min (minor)).

(5*S*,6*R*)-8-chloro-5,6-diphenyl-3-tosyl-2,3,5,6-tetrahydrobenzo[*d*]azocin-4(1*H*)-one (2u-*ent*)

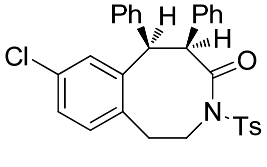

2u-*ent*

Compound **2u**-*ent* was prepared in 42% yield (21.7 mg) according to the general procedure (Table 4, entry 8). $[\alpha]_D^{20}$ = -81.1° (c = 1.0, $CHCl_3$). 89:11 e.r. (determined by

HPLC: Chiralcel AS-H Column, 20/80 *i*-PrOH/hexane, 1.0 mL/min, 235 nm, 25 °C; t_R = 15.95 min (major), 25.04 min (minor)).

5,6-diphenyl-2,3,5,6-tetrahydrobenzo[*d*]azocin-4(1*H*)-one (3a)

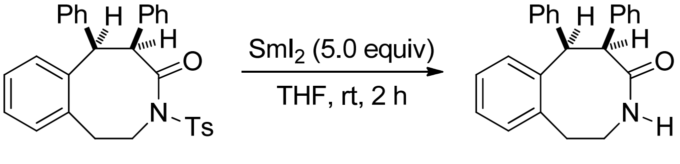

Supplementary Figure 149. Synthesis of compound **3a**.

Compound **3a** was prepared in 87% yield according to the known procedure.[19] Pale yellow solid (mp 199-200 °C). ^{1}H NMR (500 MHz, $CDCl_3$) δ 7.39 (d, *J* = 7.5 Hz, 2H), 7.34 (d, *J* = 7.5 Hz, 2H), 7.22 – 7.06 (m, 9H), 6.90 (d, *J* = 7.5 Hz, 1H), 6.22 (s, 1H), 4.92 (d, *J* = 5.0 Hz, 1H), 4.81 (d, *J* = 5.0 Hz, 1H), 3.67 – 3.54 (m, 1H), 3.35 – 3.14 (m, 2H), 2.99 – 2.87 (m, 1H); ^{13}C NMR (125 MHz, $CDCl_3$) δ 175.8, 142.4, 140.1, 139.2, 138.1, 131.0, 130.9(9), 129.9, 129.2, 127.9(2), 127.9(1), 127.2, 126.9, 126.4, 126.3, 54.1, 53.2, 42.1, 36.4; IR (neat): 3286, 2922, 1660(s), 1496, 1446, 1412, 1288, 1032, 727, 530; HRESIMS Calcd for $[C_{23}H_{21}NNaO]^+$ (M + Na^+) 350.1515, found 350.1514.

3-methyl-5,6-diphenyl-2,3,5,6-tetrahydrobenzo[*d*]azocin-4(1*H*)-one (4a)

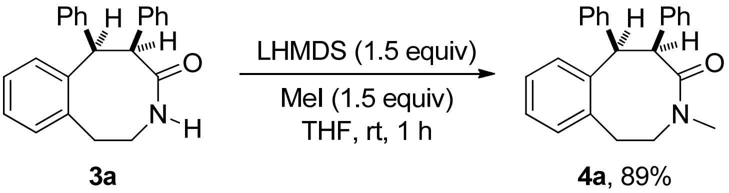

Supplementary Figure 150. Synthesis of compound **4a**.

Compound **4a** was prepared in 89% yield according to the known procedure.[20] Pale yellow oil. ^{1}H NMR (500 MHz, $CDCl_3$) δ 7.42 (d, *J* = 7.5 Hz, 2H), 7.37 (d, *J* = 7.0 Hz, 2H), 7.19 – 7.06 (m, 9H), 6.88 (d, *J* = 8.0 Hz, 1H), 5.04 (d, *J* = 6.5 Hz, 1H), 4.93 (d, *J* = 7.0 Hz, 1H), 4.09 – 3.96 (m, 1H), 3.47 – 3.33 (m, 2H), 3.11 – 3.03 (m, 1H), 2.73 (s, 3H); ^{13}C NMR (125 MHz, $CDCl_3$) δ 172.6, 142.4, 139.9, 139.4, 137.0, 130.7, 130.6, 130.5, 129.4, 127.8, 127.7, 127.1, 127.0, 126.2, 126.1, 54.1, 52.2, 50.4, 36.9, 35.3; IR (neat):

2925, 1642(s), 1492, 1453, 1398, 1185, 1091, 733, 698, 559; HRESIMS Calcd for $[C_{24}H_{23}NNaO]^+$ (M + Na^+) 364.1672, found 364.1673.

(*Z*)-5,6-diphenyl-2,3-dihydrobenzo[*d*]azocin-4(1*H*)-one (5a)

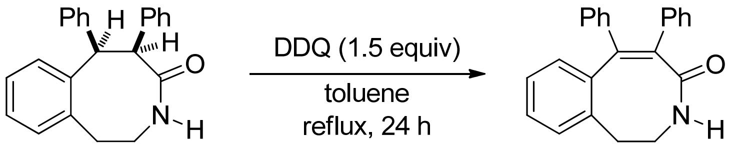

3a **5a**, 72%

Supplementary Figure 151. Synthesis of compound **5a**.

Compound **5a** was prepared in 72% yield according to the known procedure.[21] Pale yellow oil. ^{1}H NMR (400 MHz, $CDCl_3$) δ 7.52 – 7.41 (m, 2H), 7.26 – 7.09 (m, 9H), 7.04 (d, J = 7.2 Hz, 1H), 7.02 – 6.94 (m, 2H), 6.08 – 5.88 (m, 1H), 3.79 – 3.62 (m, 1H), 3.60 – 3.45 (m, 1H), 3.37 – 3.21 (m, 1H), 3.10 – 2.96 (m, 1H); ^{13}C NMR (100 MHz, $CDCl_3$) δ 173.5, 142.0, 139.6, 139.5, 135.9, 135.8, 135.2, 130.4, 130.3, 130.2, 129.3, 128.3, 128.2, 128.0, 127.7, 127.4, 127.1, 40.2, 33.9; IR (neat): 3446, 2923, 1650(s), 1487, 1442, 1404, 1350, 1110, 732, 697, 508; HRESIMS Calcd for $[C_{23}H_{19}NNaO]^+$ (M + Na^+) 348.1359, found 348.1357.

(*E*)-3-benzylidene-1-phenyl-4-tosyl-3,4,5,6-tetrahydro-1*H*-benzo[*f*][1,3]oxazocine (6a)

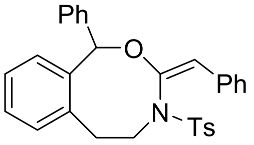

6a

Compound **6a** was prepared in 53% yield (51.0 mg) according to the general procedure (Eq. 4). White solid (mp 181-182 °C). ^{1}H NMR (500 MHz, $CDCl_3$) δ 7.81 (d, J = 8.0 Hz, 2H), 7.36 – 7.29 (m, 3H), 7.24 – 7.18 (m, 6H), 7.17 – 7.09 (m, 3H), 6.99 (d, J = 8.0 Hz, 2H), 6.93 (dd, J = 7.0 Hz, J = 1.5 Hz, 1H), 6.84 (dd, J = 7.0 Hz, J = 1.5 Hz, 1H), 5.51 (s, 1H), 5.38 (s, 1H), 4.41 – 4.30 (m, 1H), 3.10 (t, J = 12.5 Hz, 1H), 2.91 (t, J = 13.5 Hz, 1H), 2.59 (dd, J = 14.5 Hz, J = 2.0 Hz, 1H), 2.26 (s, 3H); ^{13}C NMR (125 MHz, $CDCl_3$) δ 144.2, 143.7, 139.1, 139.0, 138.3, 138.1, 133.1, 129.7, 129.4, 129.1, 129.0, 128.4, 127.8, 127.6, 127.5, 127.4, 127.2, 127.0, 126.6, 113.0, 75.8, 49.4, 34.5, 21.4; IR (neat): 2927,

1488, 1447, 1351, 1220, 1161, 1085, 776, 697, 577; HRESIMS Calcd for $[C_{30}H_{27}NNaO_3S]^+$ (M + Na$^+$) 504.1604, found 504.1606.

(*E*)-3-benzylidene-9-methyl-1-phenyl-4-tosyl-3,4,5,6-tetrahydro-1*H*-benzo[*f*][1,3]oxazocine (6p)

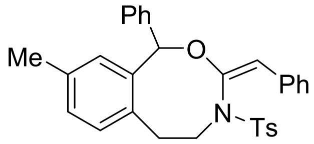

6p

White solid (mp 175-176 °C). ^{1}H NMR (500 MHz, $CDCl_3$) δ 7.79 (d, *J* = 7.5 Hz, 2H), 7.37 – 7.30 (m, 3H), 7.25 – 7.12 (m, 7H), 6.97 (d, *J* = 8.0 Hz, 2H), 6.93 (d, *J* = 7.5 Hz, 1H), 6.81 (d, *J* = 7.5 Hz, 1H), 6.62 (s, 1H), 5.54 (s, 1H), 5.34 (s, 1H), 4.35 (d, *J* = 14.5 Hz, 1H), 3.04 (t, *J* = 13.5 Hz, 1H), 2.89 (t, *J* = 13.5 Hz, 1H), 2.55 (d, *J* = 14.0 Hz, 1H), 2.24 (s, 3H), 2.15 (s, 3H); ^{13}C NMR (125 MHz, $CDCl_3$) δ 144.2, 143.6, 139.2, 138.1, 138.0, 137.0, 136.1, 133.2, 129.8, 129.7, 129.4, 129.3, 128.4, 127.7, 127.6, 127.3, 127.1, 126.9, 126.6, 112.8, 75.8, 49.6, 34.1, 21.4, 21.0; IR (neat): 2922, 1597, 1494, 1445, 1352, 1219, 1161, 1086, 688, 579; HRESIMS Calcd for $[C_{31}H_{29}NNaO_3S]^+$ (M + Na$^+$) 518.1760, found 518.1764.

Methyl-(*E*)-2-(3-benzylidene-4-tosyl-3,4,5,6-tetrahydro-1H-benzo[*f*][1,3]oxazocin-1-yl)acrylate (6ag)

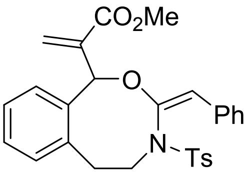

6ag

Compound **6ag** was prepared in 59% yield (57.8 mg) according to the general procedure. Yellow solid (mp 189-190 °C). ^{1}H NMR (400 MHz, $CDCl_3$) δ 7.87 (d, *J* = 8.0 Hz, 2H), 7.30 – 7.07 (m, 10H), 6.92 (d, *J* = 7.2 Hz, 1H), 6.60 (s, 1H), 6.39 (s, 1H), 5.42 (s, 1H), 5.08 (s, 1H), 4.40 – 4.30 (m, 1H), 3.49 (s, 3H), 3.11 – 2.99 (m, 1H), 2.91 – 2.80 (m, 1H), 2.52 (dd, *J* = 14.4 Hz, *J* = 2.8 Hz, 1H), 2.39 (s, 3H); ^{13}C NMR (100 MHz, $CDCl_3$) δ 165.0, 143.8, 143.7, 138.9, 138.3, 137.4, 135.7, 132.9, 129.8, 129.7, 129.2, 128.4, 127.6,

127.4, 127.1, 126.2, 113.1, 72.5, 51.6, 49.4, 34.0, 21.5; IR (neat): 2924, 1720(s), 1597, 1490, 1445, 1352, 1288, 1160, 1085, 757, 670; HRESIMS Calcd for $[C_{28}H_{27}NNaO_5S]^+$ $(M + Na^+)$ 512.1502, found 512.1501.

Of note, the two enantiomers of **1p** could not be separated by chiral HPLC, so we determined the ee of **1p**'s precursor **1p′**.

(*R*)-*N*-(2-(hydroxy(phenyl)methyl)-4-methylphenethyl)-4-methylbenzenesulfonamide (*R*-1p′)

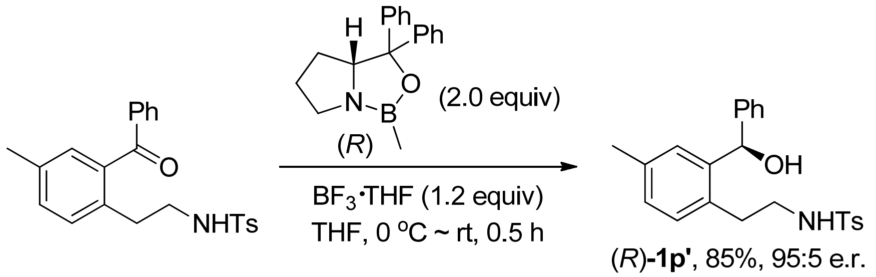

Supplementary Figure 152. Synthesis of compound (*R*)-**1p′**.

Compound (*R*)-**1p′** was prepared in 85% yield according to the known procedure.[22] The absolute configuration of (*R*)-**1p′** was determined by Corey's protocol.[23] $[\alpha]_D^{20}$ = -55.3° (c = 1.0, $CHCl_3$). 95:5 e.r. (determined by HPLC: Chiralcel AD-H Column, 20/80 *i*-PrOH/hexane, 1.0 mL/min, 235 nm, 25 °C; t_R = 18.90 min (minor), 24.01 min (major)). Pale yellow oil. ^{1}H NMR (400 MHz, $CDCl_3$) δ 7.55 (d, *J* = 8.0 Hz, 2H), 7.25 – 7.17 (m, 5H), 7.14 (d, *J* = 8.0 Hz, 2H), 7.05 (s, 1H), 6.94 – 6.85 (m, 2H), 5.88 (d, *J* = 3.6 Hz, 1H), 5.59 (t, *J* = 5.2 Hz, 1H), 3.41 (d, *J* = 4.0 Hz, 1H), 2.99 (dd, *J* = 12.4 Hz, *J* = 6.8 Hz, 2H), 2.77 – 2.60 (m, 2H), 2.35 (s, 3H), 2.22 (s, 3H); ^{13}C NMR (100 MHz, $CDCl_3$) δ 143.2, 142.9, 141.2, 136.6, 136.2, 132.9, 129.9, 129.4, 128.5(8), 128.5(6), 128.2, 127.1, 126.9, 126.5, 72.9, 44.0, 31.6, 21.4, 21.0; IR (neat): 3461 (bs), 2979, 1493, 1450, 1322, 1154, 1093, 1018, 761, 662, 550; HRESIMS Calcd for $[C_{23}H_{25}NNaO_3S]^+$ $(M + Na^+)$ 418.1447, found 418.1443.

(*S*)-*N*-(2-(hydroxy(phenyl)methyl)-4-methylphenethyl)-4-methylbenzenesulfonamide (*S*-1p′)

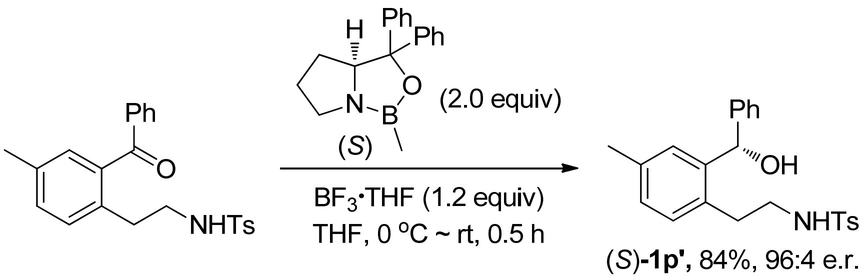

Supplementary Figure 153. Synthesis of compound (*S*)-**1p′**.

Compound (*S*)-**1p′** was prepared in 84% yield according to the known procedure.[22] $[\alpha]_D^{20}$ = +58.1° (c = 1.0, $CHCl_3$). 96:4 e.r. (determined by HPLC: Chiralcel AD-H Column, 20/80 *i*-PrOH/hexane, 1.0 mL/min, 235 nm, 25 °C; t_R = 18.66 min (major), 23.73 min (minor)).

(*R*,*E*)-3-benzylidene-9-methyl-1-phenyl-4-tosyl-3,4,5,6-tetrahydro-1*H*-benzo[*f*][1,3]oxazocine (*R*-6p)

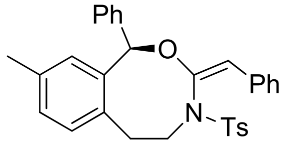

(*R*)-**6p**

$[\alpha]_D^{20}$ = -63.6° (c = 1.0, $CHCl_3$). 88:12 e.r. (determined by HPLC: Chiralcel AS-H Column, 10/90 *i*-PrOH/hexane, 1.0 mL/min, 254 nm, 25 °C; t_R = 21.67 min (minor), 30.68 min (major)).

(5*S*,6*R*)-8-methyl-5,6-diphenyl-3-tosyl-2,3,5,6-tetrahydrobenzo[*d*]azocin-4(1*H*)-one (*R*, *S*-2p)

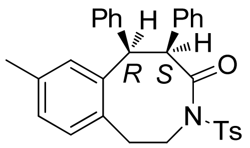

(*R*, *S*)-**2p**

Compound (*R*, *S*)-**2p** was prepared from (*R*)-**1p** (e.r. 95:5) with **Cat. 3** according to the general procedure. $[\alpha]_D^{20}$ = -120.8° (c = 1.0, $CHCl_3$). >99:1 e.r. (determined by HPLC: Chiralcel AS-H Column, 20/80 *i*-PrOH/hexane, 1.0 mL/min, 235 nm, 25 °C; t_R = 13.59 min (major), 18.99 min (minor)).

(5*S*,6*R*)-8-methyl-5,6-diphenyl-3-tosyl-2,3,5,6-tetrahydrobenzo[*d*]azocin-4(1*H*)-one (*R*, *S*-2p)

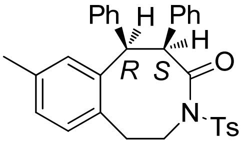

(*R*, *S*)-**2p**

Compound (*R*, *S*)-**2p** was prepared from (*R*)-**1p** (e.r. 95:5) with HOTf according to the general procedure. $[\alpha]_D^{20}$ = -104.1° (c = 1.0, $CHCl_3$). 96:4 e.r. (determined by HPLC: Chiralcel AS-H Column, 20/80 *i*-PrOH/hexane, 1.0 mL/min, 235 nm, 25 °C; t_R = 12.64 min (major), 18.74 min (minor)).

(5*R*,6*S*)-8-methyl-5,6-diphenyl-3-tosyl-2,3,5,6-tetrahydrobenzo[*d*]azocin-4(1*H*)-one (*S*, *R*-2p)

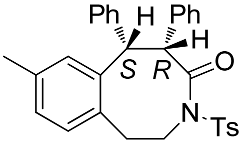

(*S*, *R*)-**2p**

Compound (*S*, *R*)-**2p** was prepared from (*S*)-**1p** (e.r. 96:4) with **Cat. 3** according to the general procedure. $[\alpha]_D^{20}$ = +80.3° (c = 1.0, $CHCl_3$). 89:11 e.r. (determined by HPLC: Chiralcel AS-H Column, 20/80 *i*-PrOH/hexane, 1.0 mL/min, 235 nm, 25 °C; t_R = 13.46 min (minor), 20.23 min (major)).

Supplementary Notes

Computational Methods

All the geometry optimizations and related single point energy amelioration were performed by Gaussian 09[24] at the level of density functional theory, using the dispersion-corrected B3LYP, M062X and ωB97XD functional without any symmetry constraints.[25-27] The 6-31G(d,p) basis set was used for optimizations for all three methods. The single point energies were further estimated using a larger basis set def2TZVPP[28-29] for all atoms with the SMD solvation model.[30] In accordance with the experimental conditions, chlorobenzene was used as solvent in the calculations. All optimized species were verified as either minima or transition structures by the presence of zero or a single imaginary vibrational frequency. Free energies were evaluated at 298.15 K using harmonic vibrational frequencies. Quasi-harmonic Gibbs free energies[31] were evaluated at the reaction temperature (333.15K) using vibrational frequencies: rigid rotor harmonic oscillator (RRHO) vibrational entropies were used above 100 cm^{-1}, while a free rotor description was used below this value, as described by Grimme.[32-33] In testing, this correction was more robust toward choice of cutoff frequency than an alternative quasi-harmonic treatment proposed by Cramer and Truhlar.[34] All the calculated structures were displayed with the CYLview software.[35] The relevant calculation results are summarized in Supplementary Figs. 111-117 and and Supplementary Datasets 1-4.

Supplementary Discussion

More Reaction Scope Study

Attempts to extend the reaction to the terminal ynamide **1an** only gave a complex mixture of products (Supplementary Figs. 118). In addition, the reaction of ynamides **1ao** and **1ap** only led to the formation of the corresponding hydroalkoxylation product **6ao** (even with longer reaction time and higher temperature) and transfer hydration product **2ap′** (presumably via intermediate **6ap**) in 94% and 90% yields, respectively (Supplementary Figs. 118). These results indicate that the formation of stable benzylic carbocation is the key for the subsequent [1,3]-rearrangement. Of note, the reaction of methoxyl-protected ynamide **1aq** only led to the formation of the corresponding hydration product **2aq′** in 78% yield (Supplementary Figs. 119).

Detailed Studies on the Asymmetric Process

1) Control experiments revealed that the chiral induction was achieved via kinetic resolution of racemic ynamide substrate (Supplementary Figs. 121). That is, one enantiomer ((*R*)-**1p)** favored formation of the desired chiral benzo[*d*]azocinone **2p**-*ent* while the other enantiomer ((*S*)-**1p**), which does not match with the **Cat. 3**, favored formation of the corresponding hydration product **2p′** catalyzed by the acid. Notably, the e.r. of **2p′** should depend on the reaction rate of the hydration of the two enantiomers in the presence of **Cat. 3**.

2) The chirality of product was determined in the hydroalkoxylation process and the [1,3]-rearrangement is a stereospecific process, as also confirmed by the control reactions of the ketene aminal **6p** (Supplementary Figs. 122). It is notable that the e.r. of product **2p** is slightly higher than the intermediate **6p**. While the exact reason for the slight chirality amplification remains unclear, we suspect that it may come from the measurement error of HPLC due to the different UV absorption of two enantiomers, or a small amount of the minor enantiomer may undergo other side reactions due to its unique configuration of eight-membered ring.

3) To further confirm the above results, we first synthesized both enantiomers of **1p**. It was found that the reaction of ynamide (*R*)-**1p** led to the desired chiral benzo[*d*]azocinone (*R,S*)-**2p** smoothly while ynamide (*S*)-**1p**, which does not match with the **Cat. 3**, was maily converted into the corresponding hydration product (*S*)-**2p′** in 65% yield catalyzed by the acid (Supplementary Figs. 123). And the residue of (*S*)-**1p** was eventually converted into the corresponding chiral benzo[*d*]azocinone (*S,R*)-**2p** with opposite enantioselectivity in 30% yield with a much longer reaction time (22 h *vs* 12 h). This result also well explained the significantly improved e.r. (>99:1) of the benzo[*d*]azocinone product in comparison with the e.r. of chiral ynamide (*R*)-**1p** (95:5), as most of the other enantiomer (*S*)-**1p** underwent hydration reaction readily in the presence of the unmatched chiral catalyst.

4) Of note, efficient chirality transfer was also observed starting from the chiral ynamide in the presence of HOTf as catalyst, which is quite consistent with the above experimental results (Supplementary Figs. 124).

5) Although the use of 20 mol % of chiral catalyst is not so impressive, chiral catalyst can be readily recovered by column chromatography and reused five times with almost unchanged enantioselectivity and reactivity (Supplementary Figs. 125).

6) As mentioned above, the kinetic resolution in this reaction is actually a parallel kinetic resolution. Therefore, the conversion is almost 100% in every case if based on the starting material. To better calculate or understand the selectivity factor of this kinetic resolution, the conversion in this case is based on the NMR yield of the cyclization product. Thus, the detailed Selectivity factors are summarized in Supplementary Table 9.

Other Mechanistic Studies

Control experiment with $H_2{}^{18}O$ isotopic labeling revealed that no incorporation of ^{18}O into the product **2a** was observed (Supplementary Figs. 126). In addition, attempts to convert the hydration product **2a′** into the corresponding benzo[*d*]azocinone **2a** under the standard conditions failed, and only **2a′** was recoveried (Supplementary Figs. 127). Notably, both HOTf and $Zn(OTf)_2$ did not promote the [1,3] rearrangement process (Supplementary Figs. 128). Finally, the structure of **6ag** was confirmed by X-ray diffraction (Supplementary Figs. 129 and Supplementary Table 7), and the configuration of **6a** was assigned (as *E* configuration of the double bond) by analogy. Of note, only the *cis* diastereoisomer (d.r. > 50:1; determined by crude ^{1}H NMR spectroscopy) of products **2** was observed in all cases, and the *trans* diastereoisomer was not detected even by monitoring the tandem process by ^{1}H NMR. These results indicate that the present [1,3]-rearrangement is highly stereospecific, and the *E* isomer of intermediate **6** leads to the stereospecific formation of *cis* diastereoisomer of final product **2**.

Supplementary References

1. Zhang, Y.; Hsung, R. P.; Tracey, M. R.; Kurtz, K. C. M.; Vera, E. L. Copper sulfate-pentahydrate-1,10-phenanthroline catalyzed amidations of alkynyl bromides. Synthesis of heteroaromatic amine substituted ynamides. *Org. Lett.* **2004**, *6*, 1151.
2. Kurouchi, H.; Kawamoto, K.; Sugimoto, H.; Nakamura, S.; Otani, Y.; Ohwada, T. Activation of electrophilicity of stable Y-delocalized carbamate cations in intramolecular aromatic substitution reaction: evidence for formation of diprotonated carbamates leading to generation of isocyanates. *J. Org. Chem.* **2012**, *77*, 9313.
3. Kong, K.; Moussa, Z.; Lee, C.; Romo, D. Total synthesis of the spirocyclic imine marine toxin (−)-Gymnodimine and an unnatural C4-epimer. *J. Am. Chem. Soc.* **2011**, *133*, 19844.
4. Sall, D. J.; Grunewald, G. L. Inhibition of phenylethanolamine *N*-methyltransferase (PNMT) by aromatic hydroxy-substituted 1,2,3,4-tetrahydroisoquinolines. Further studies on the hydrophilic pocket of the aromatic ring binding region of the active site. *J. Med. Chem.* **1987**, *30*, 2208.
5. Wang, M.; Khan, S.; Miliordos, E.; Chen, M. Enantioselective syntheses of homopropargylic alcohols via asymmetric allenylboration. *Org. Lett.* **2018**, *20*, 3810.
6. Murashige, R.; Ohtsuka, Y.; Sagisawa, K.; Shiraishi, M. Versatile synthesis of 3,4-dihydroisoquinolin-1(2*H*)-one derivatives via intra-molecular Friedel–Crafts reaction with trifluoromethanesulfonic acid. *Tetrahedron Lett.* **2015**, *56*, 3410.
7. In, J.; Hwang, S.; Kim, C.; Seo, J. H.; Kim, S. Synthesis of 3,4-dihydroisoquinolin-1-ones from *N*-Boc-(β-arylethyl)carbamates via isocyanate intermediates. *Eur. J. Org. Chem.* **2013**, 965.
8. Michon, C. et al. Catalytic asymmetric allylic alkylation of 3-arylated piperidin-2-ones. *Eur. J. Org. Chem.* **2013**, 4979.
9. He, Z.; Wibbeling, B.; Studer, A. Oxidative Heck coupling of allylic amines with 2,2,6,6-tetramethylpiperidine-*N*-oxyl (TEMPO) as oxidant for the preparation of tetrasubstituted alkenes. *Adv. Synth. Catal.* **2013**, *355*, 3639.
10. Chen, S.; Feng, B.; Zheng, X.; Yin, J.; Yang, S.; You, J. Iridium-catalyzed direct regioselective C4-amidation of indoles under mild conditions. *Org. Lett.* **2017**, *19*, 2502.

11. Mondal, A.; Hazra, R.; Grover, J.; Raghu, M.; Ramasastry, S. S. V. Organophosphine-catalyzed intramolecular hydroacylation of activated alkynes. *ACS Catal.* **2018**, *8*, 2748.
12. Gigant, N.; Claveau, E.; Bouyssou, P.; Gillaizeau, I. Diversity-oriented synthesis of polycyclic diazinic scaffolds. *Org. Lett.* **2012**, *14*, 844.
13. Hirano, S.; Tanaka, R.; Urabe, H.; Sato, F. Practical preparation of *N*-(1-alkynyl)sulfonamides and their remote diastereoselective addition to aldehydes via titanation. *Org. Lett.* **2004**, *6*, 727.
14. Salamoun, J. M. et al. Photooxygenation of an amino-thienopyridone yields a more potent PTP4A3 inhibitor. *Org. Biomol. Chem.* **2016**, *14*, 6398.
15. Nissen, F.; Richard, V.; Alayrac, C.; Witulski, B. Synthesis of β- and γ-carbolines via ruthenium and rhodium catalysed [2+2+2] cycloadditions of yne-ynamides with methylcyanoformate. *Chem. Commun.* **2011**, *47*, 6656.
16. Feng, J.; Lv, M. F.; Lu, G. P.; Cai, C. Selective formation of C–N and C=N bonds via C(sp^3)–H activation of isochroman in the presence of DTBP. *Org. Chem. Front.* **2015**, *2*, 60.
17. Poloukhtine, A.; Rassadin, V.; Kuzmin, A.; Popik, V. V. Nucleophilic cycloaromatization of ynamide-terminated enediynes. *J. Org. Chem.* **2010**, *75*, 5953.
18. Hong, X. et al. Mechanism and selectivity of *N*-triflylphosphoramide catalyzed (3^++2) cycloaddition between hydrazones and alkenes. *J. Am. Chem. Soc.* **2014**, *136*, 13769.
19. Pace, V.; Rae, J. P.; Procter, D. J. Cu(I)–NHC catalyzed asymmetric silyl transfer to unsaturated lactams and amides. *Org. Lett.* **2014**, *16*, 476.
20. Li, S.; Ji, H.; Cai, L.; Li, G. Pd(II)-catalyzed remote regiodivergent *ortho*- and *meta*-C–H functionalizations of phenylethylamines. *Chem. Sci.* **2015**, *6*, 5595.
21. Magolan, J.; Carson, C. A.; Kerr, M. A. Total synthesis of (±)-Mersicarpine. *Org. Lett.* **2008**, *10*, 1437.
22. Corey, E. J.; Bakshi, R. K.; Shibata, S. Highly enantioselective borane reduction of ketones catalyzed by chiral oxazaborolidines. Mechanism and synthetic implications. *J. Am. Chem. Soc.* **1987**, *109*, 5551.

23. Corey, E. J.; Helal, C. J. Asymmetric synthesis of (*S*)-carbinoxamine. New aspects of oxazaborolidine-catalyzed enantioselective carbonyl reduction. *Tetrahedron Lett.* **1996**, *37*, 5675.
24. Frisch, M. J. et al. *Gaussian 09, Revision D.01*, Gaussian, Inc., Wallingford CT, (2013).
25. Zhao, Y.; Truhlar, D. G. The M06 suite of density functionals for main group thermochemistry, thermochemical kinetics, noncovalent interactions, excited states, and transition elements: two new functionals and systematic testing of four M06-class functionals and 12 other functionals. *Theor. Chem. Accounts.* **2008**, *120*, 215.
26. Chai, J. D.; Head-Gordon, M. Long-range corrected hybrid density functionals with damped atom–atom dispersion corrections. *Phys. Chem. Chem. Phys.* **2008**, *10*, 6615.
27. Stephens, P. J.; Devlin, F. J.; Chabalowski, C. F.; Frisch, M. J. Ab initio calculation of vibrational absorption and circular dichroism spectra using density functional force fields. *J. Phys. Chem.* **1994**, *98*, 11623.
28. Weigend, F.; Ahlrichs, R. Balanced basis sets of split valence, triple zeta valence and quadruple zeta valence quality for H to Rn: design and assessment of accuracy. *Phys. Chem. Chem. Phys*. **2005**, *7*, 3297.
29. Weigend, F. Accurate Coulomb-fitting basis sets for H to Rn. *Phys. Chem. Chem. Phys*. **2006**, *8*, 1057.
30. Marenich, A. V.; Cramer, C. J.; Truhlar, D. G. Universal solvation model based on solute electron density and on a continuum model of the solvent defined by the bulk dielectric constant and atomic surface tensions. *J. Phys. Chem. B* **2009**, *113*, 6378.
31. Funes-Ardoiz, I.; Paton, R. S. GoodVibes fv2.0.1; doi: 0.5281/ zenodo.56091 (accessed 21 June 2016).
32. Grimme, S.; Ehrlich, S.; Georigk, L. Effect of the damping function in dispersion corrected density functional theory. *J. Comput. Chem*. **2011**, *32*, 1456.
33. Grimme, S. Supramolecular binding thermodynamics by dispersion-corrected density functional theory. *Chem. - Eur. J.* **2012**, *18*, 9955.
34. Ribeiro, R. F.; Marenich, A. V.; Cramer, C. J.; Truhlar, D. G. Use of solution-phase vibrational frequencies in continuum models for the free energy of solvation. *J. Phys. Chem. B* **2011**, *115*, 14556 .

35. Legault, C. Y. *CYLview*, *1.0b*; Université de Sherbrooke uébec, Montreal, Canada, 2009 http://www.cylview.org.
