# Supplementary material for: Stereoselective synthesis of medium lactams enabled by metal-free hydroalkoxylation/stereospecific [1,3]-rearrangement
Source: Nat Commun. 2019 Jul 19;10:3234. doi: 10.1038/s41467-019-11245-2 (PMC6642132; doi:10.1038/s41467-019-11245-2)
Supplement: Supplementary file 3 — Description of Additional Supplementary Files [file 41467_2019_11245_MOESM3_ESM.pdf]

Description of Additional Supplementary Files

Supplementary Data 1: Energy and imaginary vibrational frequency of calculated structures using B3LYP-D3 method.

Supplementary Data 2: Energy and imaginary vibrational frequency of calculated structures using M062X method.

Supplementary Data 3: Energy and imaginary vibrational frequency of calculated structures using ωB97XD method.

Supplementary Data 4: B3LYP-D3 (SMD, chlorobenzene) for optimization, Cartesian Coordinates and Energies in Hartree
