# Supplementary material for: Stereoselective synthesis of medium lactams enabled by metal-free hydroalkoxylation/stereospecific [1,3]-rearrangement
Source: Nat Commun. 2019 Jul 19;10:3234. doi: 10.1038/s41467-019-11245-2 (PMC6642132; doi:10.1038/s41467-019-11245-2)
Supplement: Supplementary file 4 — Supplementary Data 1 [file 41467_2019_11245_MOESM4_ESM.pdf]

Energy and imaginary vibrational frequency of calculated structures using B3LYP-D3 method.

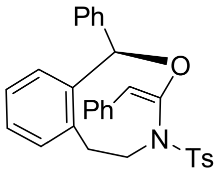

6a

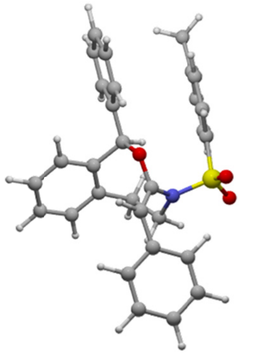

E= -1838.109717 Hartree

E_{SMD}=-1838.720846 Hartree

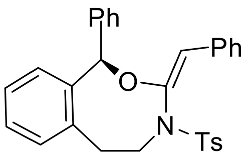

6a_iso

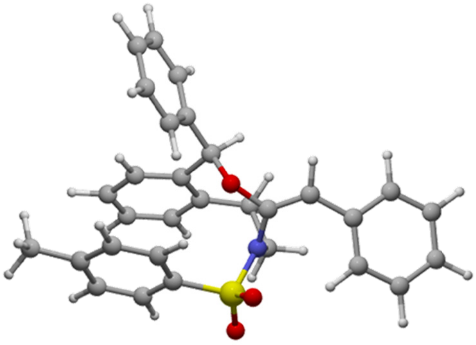

E= -1838.102423 Hartree

E_{SMD}= -1838.71482 Hartree

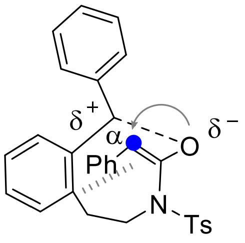

TS_cis

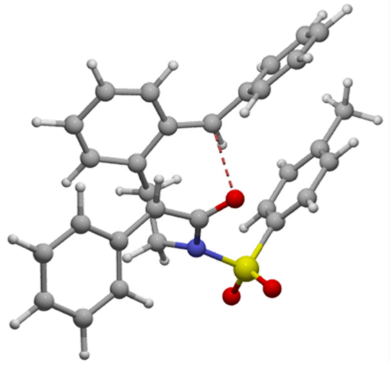

E=-1838.064467 Hartree

E_{SMD}= -1838.677033 Hartree

Frequency= -205.64 cm^{-1}

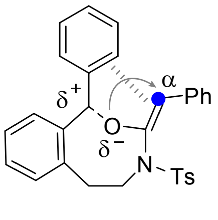

TS_trans

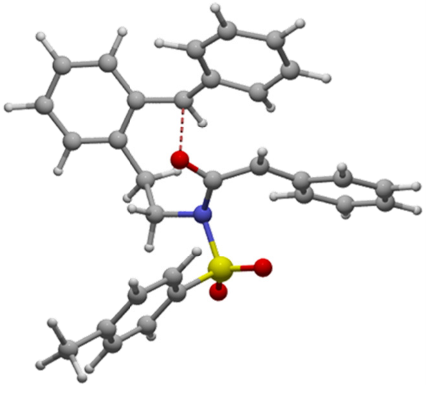

E= -1838.042421 Hartree

E_{SMD}= -1838.660252 Hartree

Frequency= -203.95 cm^{-1}

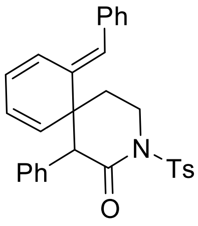

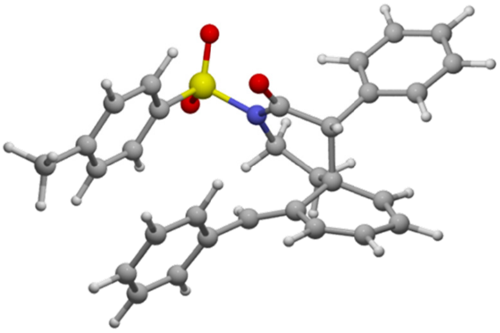

Spiro_Int

E=-1838.090101 Hartree

E_{SMD}= -1838.702537 Hartree

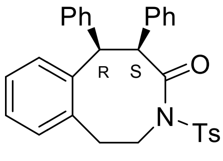

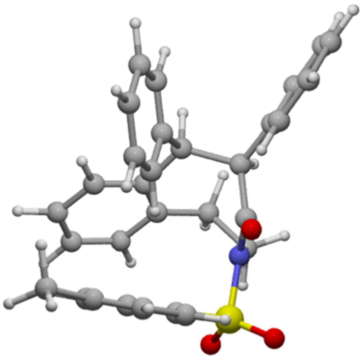

2a_cis

E= -1838.133234 Hartree

E_{SMD}= -1838.742115 Hartree

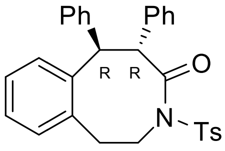

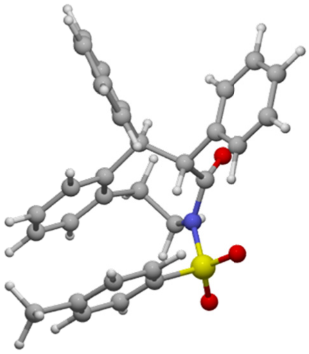

2a_trans

E= -1838.128214 Hartree

E_{SMD}=-1838.742451 Hartree
