# Supplementary material for: Stereoselective synthesis of medium lactams enabled by metal-free hydroalkoxylation/stereospecific [1,3]-rearrangement
Source: Nat Commun. 2019 Jul 19;10:3234. doi: 10.1038/s41467-019-11245-2 (PMC6642132; doi:10.1038/s41467-019-11245-2)
Supplement: Supplementary file 5 — Supplementary Data 2 [file 41467_2019_11245_MOESM5_ESM.pdf]

Energy and imaginary vibrational frequency of calculated structures using M062X method.

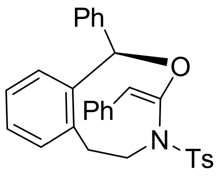

6a

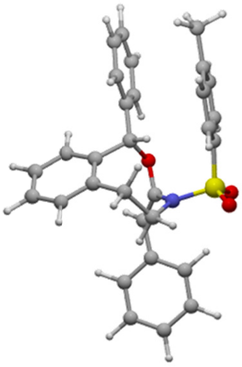

E= -1837.399649 Hartree

E_{SMD}= -1838.027471 Hartree

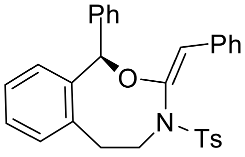

6a_iso

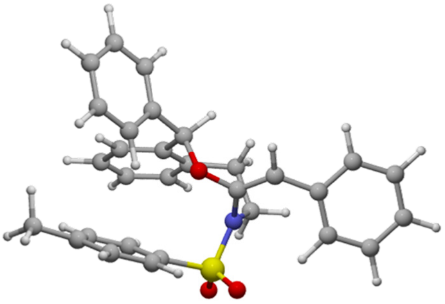

E= -1837.390928 Hartree

E_{SMD}= -1838.01858 Hartree

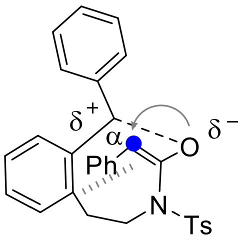

TS_cis

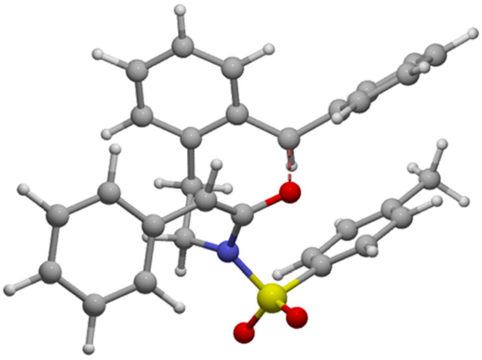

E= -1837.339143 Hartree

E_{SMD}= -1837.968691 Hartree

Frequency= -241.95 cm^{-1}

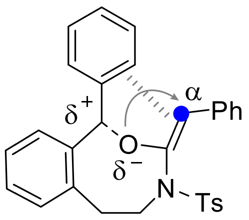

TS_trans

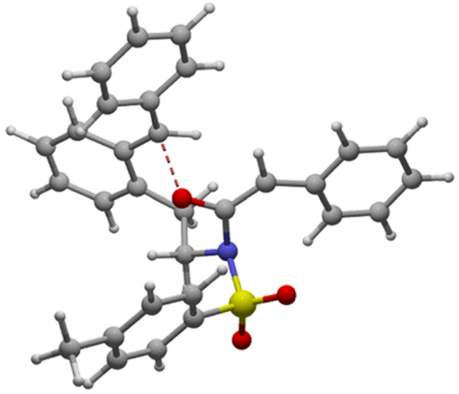

E= -1837.313697 Hartree

E_{SMD}= -1837.948261 Hartree

Frequency= -300.52 cm-1

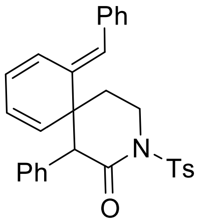

Spiro_Int

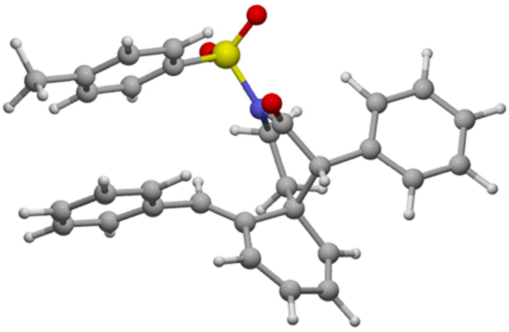

E= -1837.381669 Hartree

E_{SMD}= -1838.009498 Hartree

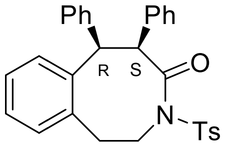

2a_cis

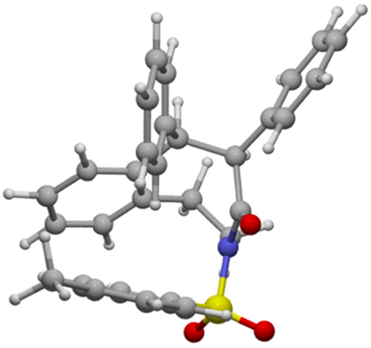

E= -1837.424244 Hartree

E_{SMD}= -1838.050336 Hartree

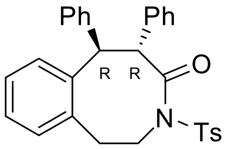

2a_trans

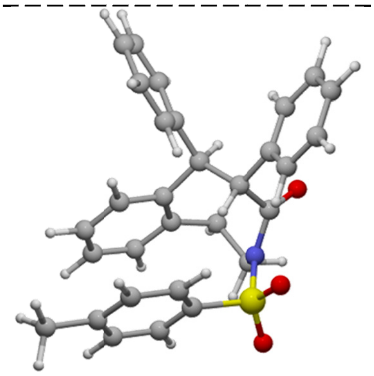

E= -1837.419198 Hartree

E_{SMD}=-1838.050075 Hartree
