# Supplementary material for: Stereoselective synthesis of medium lactams enabled by metal-free hydroalkoxylation/stereospecific [1,3]-rearrangement
Source: Nat Commun. 2019 Jul 19;10:3234. doi: 10.1038/s41467-019-11245-2 (PMC6642132; doi:10.1038/s41467-019-11245-2)
Supplement: Supplementary file 6 — Supplementary Data 3 [file 41467_2019_11245_MOESM6_ESM.pdf]

Energy and imaginary vibrational frequency of calculated structures using ωB97XD method.

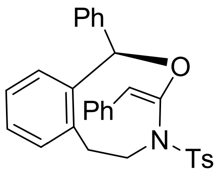

6a

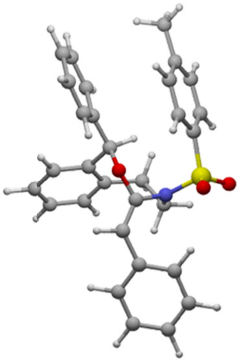

E= -1837.56529 Hartree

E_{SMD}= -1838.150955 Hartree

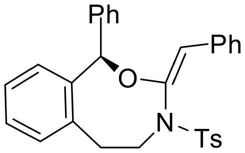

6a_iso

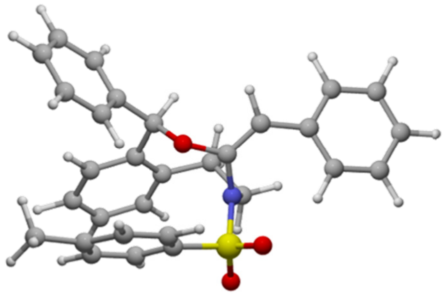

E= -1837.556936 Hartree

E_{SMD}= -1838.142449 Hartree

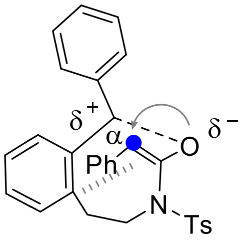

TS_cis

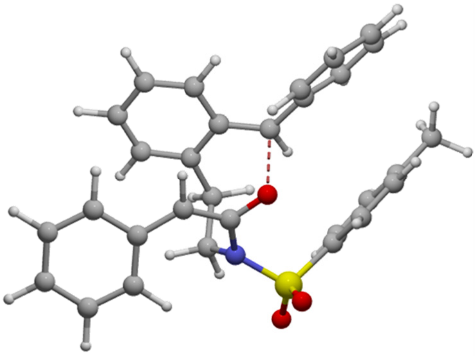

E= -1837.505693 Hartree

E_{SMD}= -1838.093161 Hartree

Frequency= -257.46 cm^{-1}

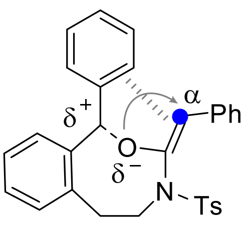

TS_trans

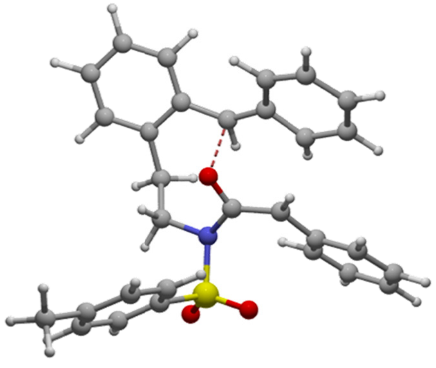

E= -1837.478131 Hartree

E_{SMD}= -1838.071071 Hartree

Frequency= -274.00 cm-1

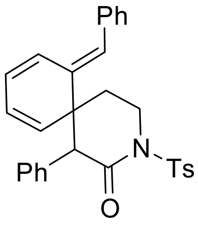

Spiro_Int

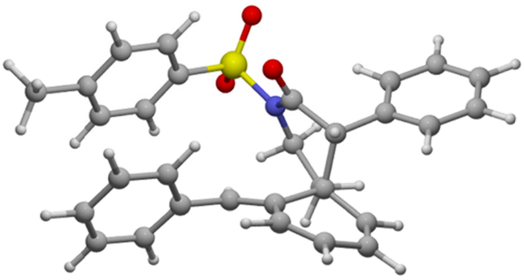

E= -1837.549305 Hartree

E_{SMD}= -1838.135975 Hartree

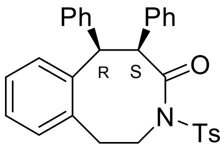

2a_cis

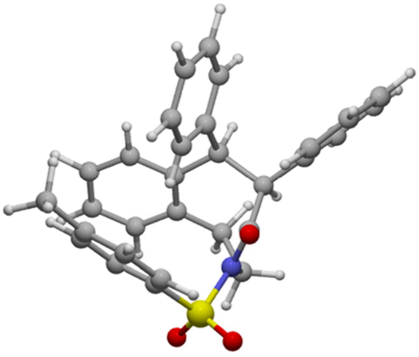

E= -1837.594085 Hartree

E_{SMD}= -1838.178264 Hartree

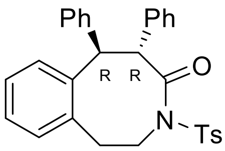

2a_trans

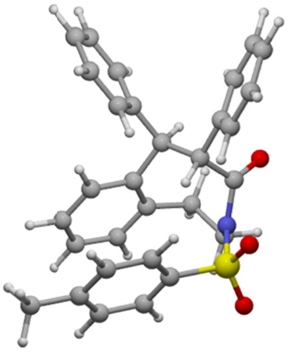

E= -1837.588939 Hartree

E_{SMD}= -1838.178308 Hartree
