# Supplementary material for: Stereoselective synthesis of medium lactams enabled by metal-free hydroalkoxylation/stereospecific [1,3]-rearrangement
Source: Nat Commun. 2019 Jul 19;10:3234. doi: 10.1038/s41467-019-11245-2 (PMC6642132; doi:10.1038/s41467-019-11245-2)
Supplement: Supplementary file 7 — Supplementary Data 4 [file 41467_2019_11245_MOESM7_ESM.pdf]

Molecular Geometries and Energies

B3LYP-D3 (SMD, chlorobenzene) for optimization, Cartesian Coordinates and Energies in Hartree

Cartesian coordinates and imaginary frequency of the calculated structures using B3LYP-D3.

6a

Number of imaginary freguencies: 0

S	0.8080682	-2.7329816	0.1048134
O	0.0802292	0.1612214	-1.1854726
O	1.1458012	-3.1221016	-1.2662316
O	1.2809852	-3.4889256	1.2699694
N	1.3835422	-1.1469946	0.3063054
C	1.3307512	-0.2441256	-0.7866026
C	2.4137742	0.2696324	-1.3887576
H	2.2245472	1.1080024	-2.0533036
C	-0.7697518	0.6885364	-0.1299896
C	3.8124172	-0.1267246	-1.1792026
C	-0.0683558	1.8433394	0.5631424
C	-0.9759278	-2.6349846	0.2136994
C	-1.5841388	-2.6808626	1.4683474
H	-0.9851008	-2.8733876	2.3518264
C	4.2030332	-1.4639616	-0.9830126
H	3.4581502	-2.2487786	-1.0362316
C	0.7341872	1.6240474	1.7024594
C	0.9006282	0.2612044	2.3499764
H	1.2915742	0.4004884	3.3641874
H	-0.0615248	-0.2508996	2.4608744
C	-3.7350798	-2.2532036	0.4116234
C	-2.9620868	-2.4874006	1.5569954
H	-3.4436418	-2.5194536	2.5303174
C	1.8761352	-0.6801476	1.6132264
H	2.0712032	-1.5643266	2.2207534
H	2.8335332	-0.1788036	1.4419764
C	5.5419862	-1.7820046	-0.7653626
H	5.8263792	-2.8201456	-0.6196766
C	-1.7225198	-2.4219296	-0.9462986
H	-1.2228158	-2.3832576	-1.9065626
C	-0.1308828	3.1207644	-0.0062556
H	-0.7502878	3.2717114	-0.8849156
C	4.8050392	0.8696764	-1.1835456
H	4.5161822	1.9047854	-1.3469766
C	0.5795452	4.1876314	0.5367874
H	0.5156482	5.1720204	0.0828594
C	-3.0964478	-2.2368336	-0.8370006
H	-3.6804178	-2.0438856	-1.7320306

C	1.4399542	2.7113014	2.2382434
H	2.0571442	2.5550204	3.1193994
C	6.5161812	-0.7807176	-0.7485496
H	7.5586782	-1.0346816	-0.5800576
C	1.3697872	3.9801924	1.6678644
H	1.9267092	4.8028124	2.1069524
C	6.1429372	0.5473714	-0.9646856
H	6.8938682	1.3323844	-0.9644246
C	-5.2167538	-1.9899786	0.5058084
H	-5.5961418	-2.1626586	1.5163814
H	-5.4307168	-0.9505576	0.2328584
H	-5.7779138	-2.6298236	-0.1832256
H	-0.9490078	-0.1122466	0.5915944
C	-2.1081118	1.0369454	-0.7455486
C	-3.1761988	1.3359934	0.1095684
C	-2.3162228	1.0472584	-2.1265006
C	-4.4353558	1.6341754	-0.4065836
H	-3.0156788	1.3310304	1.1845394
C	-3.5808818	1.3404204	-2.6443486
H	-1.4911678	0.8091604	-2.7869206
C	-4.6433438	1.6321944	-1.7893926
H	-5.2542888	1.8669444	0.2682034
H	-3.7327808	1.3402514	-3.7199396
H	-5.6250528	1.8602654	-2.1939466

Energy (0K) = -1838.109717
Energy (0K) + ZPE = -1837.607638
Enthalpy (298K) = -1837.577256
Free Energy (298K) = -1837.669300

6a_iso

Number of imaginary freguencies: 0

S	-1.2696695	-2.1659772	-0.9656824
O	-0.3401605	0.9335508	-0.7816244
O	-1.7722955	-1.8305582	-2.3015054
O	-1.7187825	-3.3607442	-0.2417584
N	-1.6684845	-0.8504162	0.0220486
C	-1.6034985	0.4577928	-0.5150684
C	-2.6687625	1.2508408	-0.7104584
H	-2.4406255	2.2839048	-0.9622994
C	0.4074685	1.4588428	0.3514136
H	-0.2989075	2.0503908	0.9480806
C	1.0103125	0.3739538	1.2522876
C	2.3733655	0.0669308	1.1480946
H	2.9731415	0.5764438	0.4035926
C	2.9745015	-0.8719212	1.9821246
H	4.0335005	-1.0877082	1.8763116

C	2.2125335	-1.5280562	2.9462066
H	2.6689135	-2.2574782	3.6089846
C	0.8538805	-1.2412992	3.0529856
H	0.2544275	-1.7544642	3.8011096
C	0.2330185	-0.2988792	2.2223016
C	-1.2605075	-0.1027292	2.3602666
H	-1.5543745	0.9319678	2.1569196
H	-1.5733525	-0.3147152	3.3885086
C	-2.0584275	-1.0325592	1.4284186
H	-1.8793085	-2.0774152	1.6849006
H	-3.1331255	-0.8422712	1.5321666
C	-4.0880235	0.8992788	-0.5605754
C	-4.6005455	-0.3640412	-0.9082844
H	-3.9429525	-1.1017252	-1.3513444
C	-5.9523685	-0.6486002	-0.7274054
H	-6.3322845	-1.6275422	-1.0048374
C	-6.8188735	0.3170278	-0.2090274
H	-7.8718745	0.0893678	-0.0714484
C	-6.3258715	1.5821828	0.1160196
H	-6.9933755	2.3444628	0.5075696
C	-4.9748645	1.8714708	-0.0649484
H	-4.5939125	2.8574978	0.1892696
C	1.4413775	2.4154108	-0.2103994
C	1.9000065	2.3123848	-1.5269374
H	1.4798715	1.5528658	-2.1747644
C	2.8814305	3.1858058	-1.9999684
H	3.2281995	3.0987388	-3.0257504
C	3.4129055	4.1684328	-1.1639974
H	4.1744575	4.8482828	-1.5345034
C	2.9557895	4.2763688	0.1515466
H	3.3597715	5.0410388	0.8085996
C	1.9762065	3.4043148	0.6234746
H	1.6287625	3.4832158	1.6506916
C	0.5153415	-2.2447472	-1.0837134
C	1.1753195	-1.4547752	-2.0262274
H	0.6047745	-0.8041112	-2.6772574
C	2.5619695	-1.5248412	-2.1109264
H	3.0832915	-0.9070392	-2.8370314
C	3.2968995	-2.3778162	-1.2748394
C	2.6037945	-3.1749222	-0.3558854
H	3.1568175	-3.8467442	0.2944006
C	1.2165835	-3.1122282	-0.2490424
H	0.6797335	-3.7300602	0.4610736
C	4.8039705	-2.4005392	-1.3303204
H	5.1755965	-2.1517872	-2.3284834
H	5.2032335	-3.3796292	-1.0501754

H	5.2240645	-1.6644282	-0.6328764

Energy (0K) = -1838.102423
Energy (0K) + ZPE = -1837.600672
Enthalpy (298K) = -1837.570232
Free Energy (298K) = -1837.662474

TS_cis

Number of imaginary freguencies: 1

S	0.0864979	-3.2216748	-0.6200053
O	0.1033399	-0.4557568	-1.6586383
O	0.1694179	-3.4238478	-2.0616923
O	0.4269209	-4.2881698	0.3289987
N	1.1780609	-1.9425868	-0.2370753
C	1.1086639	-0.7428408	-1.0054163
C	2.1867149	0.1994482	-0.8122463
H	1.9257189	1.1715542	-1.2149913
C	-1.0276401	0.9630022	0.4410357
C	3.6181519	-0.0749648	-0.7827413
C	0.2074169	1.5118842	0.7114777
C	-1.5566231	-2.6465228	-0.1994753
C	-2.0300611	-2.8512838	1.0970537
H	-1.4215721	-3.3863628	1.8176367
C	4.1808269	-1.3692358	-0.8575873
H	3.5287549	-2.2294098	-0.9636333
C	1.3174759	0.7131542	1.2594117
C	1.0614009	-0.6546258	1.8918257
H	1.4799989	-0.6471888	2.9047037
H	-0.0084631	-0.8407108	2.0062727
C	-4.0984831	-1.7226588	0.4899027
C	-3.2985711	-2.3790918	1.4344277
H	-3.6751591	-2.5338498	2.4418407
C	1.6874419	-1.8388018	1.1436507
H	1.4703949	-2.7792018	1.6497767
H	2.7741479	-1.7362688	1.1150117
C	5.5598389	-1.5477498	-0.8362683
H	5.9680269	-2.5519598	-0.9039443
C	-2.3302391	-1.9956808	-1.1616543
H	-1.9253931	-1.8298888	-2.1509343
C	0.4924699	2.9011392	0.4127867
H	-0.2828241	3.4889752	-0.0643863
C	4.5025969	1.0250222	-0.7264153
H	4.0916019	2.0287262	-0.7158103
C	1.6571399	3.4966002	0.7961507
H	1.8171609	4.5524582	0.5995387
C	-3.5955051	-1.5411948	-0.8075593
H	-4.1957801	-1.0141228	-1.5430473

C	2.4645429	1.4258452	1.7561387
H	3.2216289	0.8595412	2.2906947
C	6.4190889	-0.4481948	-0.7424463
H	7.4949489	-0.5943878	-0.7259583
C	2.6489769	2.7551152	1.5158577
H	3.5416409	3.2571612	1.8761357
C	5.8821039	0.8395162	-0.6957623
H	6.5398649	1.7028082	-0.6485513
C	-5.4804111	-1.2329618	0.8433917
H	-5.6149281	-1.1474868	1.9254237
H	-5.6740891	-0.2553588	0.3923437
H	-6.2455441	-1.9233418	0.4687127
H	-1.2664311	-0.0226798	0.8248797
C	-2.1238851	1.6087112	-0.2918483
C	-3.4089231	1.6697122	0.2744067
C	-1.9330871	2.1186312	-1.5879233
C	-4.4645571	2.2577072	-0.4191433
H	-3.5717501	1.2576422	1.2659037
C	-2.9943361	2.6920432	-2.2861653
H	-0.9596201	2.0007932	-2.0512783
C	-4.2611651	2.7709092	-1.7031083
H	-5.4469131	2.3154822	0.0413377
H	-2.8347471	3.0663982	-3.2931533
H	-5.0862761	3.2200812	-2.2478703

Energy (0K) = -1838.064467
Energy (0K) + ZPE = -1837.565095
Enthalpy (298K) = -1837.534798
Free Energy (298K) = -1837.625178

TS2_trans

Number of imaginary freguencies: 1

S	-1.6775717	-1.8843804	-0.9421685
O	0.1925683	0.6276786	0.9017825
O	-2.1011527	-2.0328854	-2.3368595
O	-1.1055327	-2.9970444	-0.1902725
N	-0.5469427	-0.5611344	-0.9159885
C	0.2853683	-0.4312894	0.2719745
C	1.3012823	-1.4068594	0.6023095
H	1.5093363	-1.3858064	1.6660185
C	2.0680043	1.7061416	-0.6501715
C	1.6904743	-2.6236894	-0.1028805
C	1.3588423	2.9428376	-0.8594295
C	-3.0698217	-1.2589584	0.0032255
C	-2.9230577	-1.0815514	1.3813105
H	-1.9877407	-1.3324934	1.8686795
C	1.6441023	-2.7528224	-1.5074165

H	1.2128433	-1.9539694	-2.1002375
C	0.1767083	2.8963896	-1.6547725
C	-0.3484417	1.6173756	-2.2831755
H	-0.9856197	1.9116266	-3.1230745
H	0.4506153	1.0150546	-2.7246665
C	-5.2119647	-0.2596044	1.4899035
C	-3.9946537	-0.5791874	2.1117795
H	-3.8868367	-0.4319684	3.1828615
C	-1.2101877	0.7002936	-1.3631415
H	-2.0759087	0.3933746	-1.9473275
H	-1.5611267	1.2550496	-0.4912225
C	2.1212523	-3.8978764	-2.1341975
H	2.0632063	-3.9780014	-3.2159485
C	-4.2637967	-0.9473404	-0.6433525
H	-4.3480617	-1.0993314	-1.7138315
C	1.7798993	4.1785326	-0.3260345
H	2.7177293	4.2259166	0.2157565
C	2.2606863	-3.6788734	0.6350845
H	2.3146353	-3.5953474	1.7173305
C	1.0405363	5.3377016	-0.5259395
H	1.3871353	6.2798906	-0.1121565
C	-5.3291777	-0.4520174	0.1075115
H	-6.2648737	-0.2102374	-0.3890905
C	-0.5521177	4.0755166	-1.8307705
H	-1.4625037	4.0473746	-2.4243815
C	2.6712673	-4.9426054	-1.3847145
H	3.0412513	-5.8360774	-1.8786145
C	-0.1386097	5.2859886	-1.2721175
H	-0.7245777	6.1856896	-1.4343705
C	2.7397773	-4.8247444	0.0044205
H	3.1643443	-5.6285454	0.5994385
C	-6.3741617	0.2537786	2.3040785
H	-6.0398407	0.9419016	3.0865215
H	-7.1040977	0.7756556	1.6792675
H	-6.8960367	-0.5734334	2.8003325
H	1.8951353	0.9725576	-1.4317825
C	2.8416393	1.2071186	0.3838985
C	3.2463593	-0.1749754	0.2489925
C	3.1147613	1.8620016	1.6367935
C	4.1099083	-0.7545624	1.2275715
H	3.2381923	-0.6089834	-0.7434365
C	3.8701073	1.2438056	2.5911185
H	2.6878353	2.8387056	1.8277745
C	4.4033843	-0.0688794	2.3748185
H	4.4832703	-1.7600034	1.0626985
H	4.0674233	1.7452836	3.5340065

H	5.0345303	-0.5177624	3.1362845

Energy (0K) = -1838.042421
Energy (0K) + ZPE = -1837.543506
Enthalpy (298K) = -1837.512948
Free Energy (298K) = -1837.605756

spiro_Int

Number of imaginary freguencies: 0

S	-0.1817714	-2.9338648	0.6192544
O	0.6126736	-1.2373818	-1.6201086
O	0.3903216	-3.8933008	-0.3194346
O	-0.3289024	-3.2630368	2.0417744
N	0.8476346	-1.5224018	0.6364474
C	1.1600966	-0.9214598	-0.5766346
C	2.2279426	0.1618052	-0.5258086
H	2.0736976	0.7415482	-1.4383676
C	-0.5888124	1.2349542	0.9718114
C	3.6388696	-0.4171358	-0.5654806
C	0.5483426	1.7375392	0.4285144
C	-1.7654194	-2.3662268	0.0226024
C	-2.7036894	-1.9150918	0.9527204
H	-2.4554394	-1.8938768	2.0075864
C	3.9577996	-1.6907888	-0.0727146
H	3.1819296	-2.3399378	0.3163734
C	1.9366506	1.1315362	0.7046854
C	2.0533986	0.2739352	1.9790184
H	3.1204006	0.0989082	2.1385734
H	1.6938976	0.8484372	2.8379054
C	-4.2875304	-1.5722138	-0.8583616
C	-3.9581584	-1.5163998	0.5022654
H	-4.6873894	-1.1444668	1.2150394
C	1.3492966	-1.1032758	1.9652174
H	0.4934506	-1.1055998	2.6414324
H	2.0326986	-1.8693208	2.3410514
C	5.2704736	-2.1646398	-0.1162686
H	5.4913506	-3.1569758	0.2656664
C	-2.0599214	-2.4312978	-1.3394686
H	-1.3048644	-2.7723518	-2.0352576
C	0.5262336	2.8721172	-0.4923396
H	-0.4212034	3.1707662	-0.9238726
C	4.6672126	0.3520412	-1.1324296
H	4.4319086	1.3290092	-1.5413506
C	1.6265886	3.6342582	-0.6923436
H	1.5696616	4.5183692	-1.3203896
C	-3.3231354	-2.0326798	-1.7661696
H	-3.5635334	-2.0760718	-2.8248446

C	3.0013846	2.2096952	0.7161484
H	3.9248856	1.9929742	1.2438714
C	6.2874966	-1.3787048	-0.6561986
H	7.3076366	-1.7494878	-0.6897996
C	2.8645676	3.3456272	0.0115394
H	3.6712546	4.0727312	-0.0221246
C	5.9784386	-0.1184748	-1.1715236
H	6.7569226	0.4963192	-1.6141786
C	-5.6619204	-1.1703608	-1.3310816
H	-6.0885234	-0.3954708	-0.6892896
H	-5.6360394	-0.7881338	-2.3556736
H	-6.3438574	-2.0294108	-1.3181966
H	-0.5095474	0.5249022	1.7878154
C	-1.9742454	1.5439772	0.5734364
C	-2.9424434	1.8320312	1.5498474
C	-2.3751534	1.4893982	-0.7726006
C	-4.2599784	2.1078472	1.1886964
H	-2.6509884	1.8524342	2.5966664
C	-3.6921524	1.7655032	-1.1329396
H	-1.6525714	1.1872962	-1.5241356
C	-4.6373194	2.0862102	-0.1562276
H	-4.9919754	2.3429672	1.9561004
H	-3.9855844	1.7111322	-2.1771326
H	-5.6630494	2.3035722	-0.4393136

Energy (0K) = -1838.090101
Energy (0K) + ZPE = -1837.588615
Enthalpy (298K) = -1837.557857
Free Energy (298K) = -1837.650523

2a_cis

Number of imaginary freguencies: 0

S	2.2383469	-1.9791085	-1.9081721
O	-0.3179701	-0.6530675	-2.0968401
O	1.9563279	-2.2167125	-3.3207401
O	3.2800049	-2.7474155	-1.2157131
N	0.7956299	-2.2743695	-0.9464671
C	-0.2965031	-1.4361855	-1.1606551
C	-1.3639461	-1.4814705	-0.0716851
H	-1.3848721	-2.4875695	0.3343229
C	-0.9669821	-0.5198625	1.1419949
H	-1.6849531	-0.8099265	1.9210509
C	0.4376499	-0.6899935	1.7740879
C	1.0259889	0.4431255	2.3629149
H	0.5111639	1.3931825	2.3044099
C	2.2541169	0.3961355	3.0182909
H	2.6617709	1.3020695	3.4573849

C	2.9487329	-0.8071275	3.1009439
H	3.9140979	-0.8636195	3.5946179
C	2.3716439	-1.9489685	2.5524549
H	2.8941599	-2.8990035	2.6244589
C	1.1235729	-1.9209595	1.9134579
C	0.5790729	-3.2404035	1.4177779
H	-0.4751101	-3.3377465	1.6911929
H	1.0889149	-4.0562695	1.9394099
C	0.7795479	-3.4821805	-0.1020761
H	1.7508729	-3.9514875	-0.2498421
H	0.0200169	-4.1721785	-0.4848911
C	-2.7855351	-1.2165405	-0.5440651
C	-3.1364121	-0.2165455	-1.4623301
H	-2.3612281	0.3900165	-1.9085551
C	-4.4739621	-0.0068955	-1.7971601
H	-4.7269811	0.7704275	-2.5123491
C	-5.4833101	-0.7768215	-1.2172031
H	-6.5231871	-0.6049595	-1.4798581
C	-5.1461641	-1.7697485	-0.2973001
H	-5.9198951	-2.3791285	0.1607949
C	-3.8076111	-1.9856355	0.0299719
H	-3.5531221	-2.7643635	0.7463629
C	-1.2756971	0.9280325	0.7791879
C	-0.4622721	1.6494515	-0.1074881
H	0.4302149	1.1943895	-0.5120941
C	-0.7886321	2.9538485	-0.4706711
H	-0.1473071	3.4862245	-1.1672821
C	-1.9324041	3.5651405	0.0493819
H	-2.1879301	4.5816375	-0.2356441
C	-2.7431891	2.8604875	0.9387849
H	-3.6351091	3.3239585	1.3500709
C	-2.4140921	1.5521815	1.2985619
H	-3.0618361	1.0020385	1.9757509
C	2.5458089	-0.2416195	-1.6351081
C	2.2106609	0.6791325	-2.6272381
H	1.7648949	0.3321475	-3.5507121
C	2.4269169	2.0325285	-2.3817121
H	2.1620989	2.7602355	-3.1435601
C	2.9561229	2.4719265	-1.1592681
C	3.2985639	1.5167275	-0.1921311
H	3.7065929	1.8371855	0.7614289
C	3.0990529	0.1583485	-0.4190951
H	3.3486169	-0.5728455	0.3400989
C	3.0986339	3.9439355	-0.8633901
H	3.9617099	4.1438485	-0.2216861
H	2.2078899	4.3099375	-0.3374591

H	3.2068049	4.5324265	-1.7787271

Energy (0K) = -1838.133234
Energy (0K) + ZPE = -1837.629862
Enthalpy (298K) = -1837.599901
Free Energy (298K) = -1837.688727

2a_trans

Number of imaginary freguencies: 0

S	1.7628666	-2.8071184	-0.4082512
O	-1.1978124	-2.8117234	1.8283208
O	0.7601856	-3.3028874	-1.3545492
O	2.9021656	-3.6070524	0.0433478
N	0.8896826	-2.4359884	0.9984358
C	-0.5198814	-2.2730944	0.9785618
C	-1.0497154	-1.1507864	0.1041078
H	-0.3711624	-0.9677784	-0.7270902
C	-0.9878834	0.1210746	1.0606678
H	-1.3930934	-0.2125514	2.0211138
C	0.4458026	0.6431416	1.3314408
C	0.8993446	1.7650396	0.6184528
H	0.2624506	2.1999556	-0.1411672
C	2.1269646	2.3681396	0.8767918
H	2.4261446	3.2426896	0.3064698
C	2.9509296	1.8571886	1.8750778
H	3.9044106	2.3239646	2.1042508
C	2.5366246	0.7278366	2.5728258
H	3.1803716	0.3121656	3.3445518
C	1.3063476	0.1007266	2.3217848
C	1.0268376	-1.1711044	3.1004008
H	-0.0336874	-1.2823344	3.3388478
H	1.5557946	-1.1431624	4.0586568
C	1.5024196	-2.4289044	2.3378298
H	2.5849396	-2.4154744	2.2054808
H	1.2329086	-3.3403954	2.8780958
C	-2.4223144	-1.4866284	-0.4408832
C	-2.5565894	-1.7947364	-1.7985702
H	-1.6757284	-1.7921444	-2.4352982
C	-3.8015484	-2.1232154	-2.3358482
H	-3.8881494	-2.3597324	-3.3923972
C	-4.9290974	-2.1516754	-1.5149312
H	-5.9007484	-2.4037454	-1.9296472
C	-4.8005204	-1.8599164	-0.1551822
H	-5.6724154	-1.8876294	0.4919008
C	-3.5557664	-1.5331464	0.3792948
H	-3.4619464	-1.3169964	1.4370298
C	-1.9116724	1.2336846	0.5806828

C	-2.0551054	1.5647616	-0.7736502
H	-1.5186484	0.9972936	-1.5270112
C	-2.8925754	2.6065056	-1.1698972
H	-2.9948064	2.8417966	-2.2252782
C	-3.6043784	3.3372386	-0.2169252
H	-4.2616374	4.1446386	-0.5257272
C	-3.4697224	3.0175466	1.1343378
H	-4.0212844	3.5760106	1.8851018
C	-2.6299614	1.9744026	1.5256908
H	-2.5296444	1.7303256	2.5803198
C	2.4190736	-1.2813414	-1.0778512
C	1.8045116	-0.6976454	-2.1866382
H	0.9471686	-1.1801174	-2.6417242
C	2.3294306	0.4820836	-2.7089912
H	1.8547876	0.9397926	-3.5724272
C	3.4623806	1.0806726	-2.1437642
C	4.0754596	0.4542216	-1.0497492
H	4.9605896	0.9002246	-0.6059532
C	3.5649276	-0.7219744	-0.5122322
H	4.0522966	-1.2070484	0.3250478
C	3.9936426	2.3893816	-2.6713452
H	5.0849826	2.4343006	-2.6099322
H	3.5997166	3.2257386	-2.0802912
H	3.7013346	2.5555046	-3.7117152

Energy (0K) = -1838.128214

Energy (0K) + ZPE = -1837.625559

Enthalpy (298K) = -1837.595255

Free Energy (298K) = -1837.686549

M062X (SMD, chlorobenzene) for optimization, Cartesian Coordinates and Energies in Hartree

Cartesian coordinates and imaginary frequency of the calculated structures using M062X.

6a_1

Number of imaginary freguencies: 0

S	-0.8114095	-2.7001692	0.0617514
O	-0.0637075	0.1484848	-1.2105536
O	-1.1133115	-3.0716902	-1.3096066
O	-1.3074055	-3.4625132	1.1974324
N	-1.3846295	-1.1391892	0.2660944
C	-1.3193585	-0.2306822	-0.8209696
C	-2.3953885	0.2983788	-1.4087506
H	-2.2092985	1.1287888	-2.0841666
C	0.7659095	0.6683898	-0.1554716
C	-3.7909295	-0.1072072	-1.1684926
C	0.0685435	1.8344358	0.5175074

C	0.9560105	-2.6025992	0.2104444
C	1.5290685	-2.6387112	1.4793114
H	0.9081185	-2.8469172	2.3454004
C	-4.1635005	-1.4534672	-1.0603536
H	-3.4120705	-2.2249902	-1.1970406
C	-0.7558405	1.6262508	1.6371174
C	-0.9328875	0.2731778	2.2985244
H	-1.3431335	0.4328548	3.3013104
H	0.0290375	-0.2344372	2.4348644
C	3.6866705	-2.1510782	0.4790224
C	2.8939665	-2.4078162	1.6036004
H	3.3537515	-2.4244742	2.5880814
C	-1.8931985	-0.6737522	1.5618724
H	-2.0893625	-1.5600962	2.1668364
H	-2.8512695	-0.1752182	1.3773324
C	-5.4897685	-1.7952472	-0.8193006
H	-5.7659055	-2.8421022	-0.7422336
C	1.7210315	-2.3694642	-0.9271476
H	1.2413075	-2.3344482	-1.8995396
C	0.1557995	3.1040608	-0.0555336
H	0.7991855	3.2443758	-0.9199686
C	-4.7775825	0.8801848	-1.0553336
H	-4.4946285	1.9255108	-1.1473656
C	-0.5573295	4.1760468	0.4664464
H	-0.4739495	5.1580738	0.0123534
C	3.0860915	-2.1487122	-0.7818006
H	3.6882215	-1.9244672	-1.6582336
C	-1.4653675	2.7165308	2.1502044
H	-2.1011035	2.5668728	3.0192554
C	-6.4618575	-0.8053802	-0.6904796
H	-7.4960215	-1.0769642	-0.5038116
C	-1.3738605	3.9790728	1.5760444
H	-1.9339385	4.8072188	1.9987774
C	-6.1027085	0.5342788	-0.8138006
H	-6.8553715	1.3110458	-0.7216556
C	5.1620165	-1.8883702	0.6246334
H	5.3731165	-1.3297692	1.5403994
H	5.5384175	-1.3106802	-0.2225786
H	5.7209105	-2.8287032	0.6732434
H	0.9287805	-0.1252842	0.5814964
C	2.1141535	0.9945548	-0.7547706
C	3.1873945	1.2240098	0.1091934
C	2.3245575	1.0221528	-2.1312126
C	4.4587235	1.4679498	-0.3953146
H	3.0214915	1.1946508	1.1840404
C	3.6016545	1.2648678	-2.6371616

H	1.4916585	0.8322528	-2.7986766
C	4.6706335	1.4830638	-1.7744176
H	5.2868835	1.6390538	0.2857934
H	3.7593105	1.2795708	-3.7111226
H	5.6636755	1.6684388	-2.1715196

Energy (0K) = -1837.399649
Energy (0K) + ZPE = -1836.892903
Enthalpy (298K) = -1836.862931
Free Energy (298K) = -1836.953723

6a_iso_1

Number of imaginary freguencies: 0

S	-1.3910716	-2.3831359	-0.3725636
O	-0.1136126	0.5989301	-0.6767476
O	-2.1664286	-2.5120399	-1.5991416
O	-1.5701756	-3.3261679	0.7214254
N	-1.6750116	-0.8716819	0.2908424
C	-1.4348986	0.2926701	-0.4781256
C	-2.3825386	1.1216751	-0.9329596
H	-2.0142276	2.0481211	-1.3688876
C	0.5489344	1.3379301	0.3666204
H	-0.1390446	2.1288531	0.6985434
C	0.9330294	0.4767301	1.5769364
C	2.2695844	0.1118321	1.7693884
H	3.0171474	0.4461781	1.0580654
C	2.6626874	-0.6810699	2.8421244
H	3.7086624	-0.9453199	2.9634794
C	1.7123844	-1.1280719	3.7531304
H	2.0016614	-1.7481169	4.5955384
C	0.3836114	-0.7572559	3.5840894
H	-0.3635686	-1.0926109	4.2993764
C	-0.0246606	0.0485011	2.5172134
C	-1.4983766	0.3622161	2.4083604
H	-1.6738496	1.3310941	1.9306774
H	-1.9356216	0.4237221	3.4099744
C	-2.2521506	-0.7222269	1.6316584
H	-2.1569636	-1.6875469	2.1317664
H	-3.3167856	-0.4692939	1.5565724
C	-3.8436996	0.9699081	-0.8373536
C	-4.4922016	-0.2705739	-0.9138546
H	-3.9136526	-1.1699819	-1.0971116
C	-5.8761426	-0.3424259	-0.7961406
H	-6.3655656	-1.3089489	-0.8630386
C	-6.6342076	0.8116481	-0.6119586
H	-7.7140166	0.7472071	-0.5232356
C	-6.0002966	2.0500301	-0.5581726

H	-6.5819136	2.9571631	-0.4277916
C	-4.6174636	2.1269431	-0.6767706
H	-4.1233486	3.0943441	-0.6365776
C	1.7473374	1.9929461	-0.2850126
C	2.3450764	1.4180671	-1.4068486
H	1.9100814	0.5216851	-1.8373586
C	3.4837594	1.9963191	-1.9623856
H	3.9398564	1.5471811	-2.8397466
C	4.0329414	3.1471741	-1.4028546
H	4.9189494	3.5971661	-1.8390456
C	3.4362184	3.7223891	-0.2836226
H	3.8549424	4.6225851	0.1547764
C	2.2982604	3.1460101	0.2727574
H	1.8378424	3.5903991	1.1520384
C	0.3161694	-2.3617729	-0.8538566
C	0.6370494	-2.2146339	-2.1985946
H	-0.1576936	-2.1423799	-2.9329906
C	1.9779254	-2.1605259	-2.5630726
H	2.2446734	-2.0416979	-3.6095956
C	2.9882124	-2.2446539	-1.6005386
C	2.6292744	-2.4146689	-0.2602136
H	3.4045434	-2.4860129	0.4978804
C	1.2961584	-2.4703399	0.1253114
H	1.0191584	-2.5778169	1.1711814
C	4.4363444	-2.1001679	-1.9867226
H	4.5951644	-2.3367439	-3.0410286
H	5.0754854	-2.7514589	-1.3857116
H	4.7660604	-1.0682759	-1.8200946

Energy (0K) = -1837.390928
Energy (0K) + ZPE = -1836.884233
Enthalpy (298K) = -1836.854229
Free Energy (298K) = -1836.945075

TS_cis_1

Number of imaginary freguencies: 1

S	0.4138892	-2.9364738	-0.5850790
O	-0.0668198	-0.0506928	-1.3445250
O	0.5140722	-3.0401388	-2.0281290
O	0.8861322	-3.9920758	0.3004010
N	1.2614942	-1.5638138	-0.1519060
C	1.1453092	-0.4026408	-0.9881960
C	2.2327322	0.3503022	-1.2776100
H	2.0133092	1.3274552	-1.7003220
C	-0.9893548	0.7776762	0.1732210
C	3.6388292	0.0048312	-1.0589450
C	0.0789572	1.5831922	0.7498420

C	-1.2931338	-2.6458708	-0.1702070
C	-1.7208268	-2.9001048	1.1281550
H	-1.0405838	-3.3578338	1.8400720
C	4.1181272	-1.3079508	-1.1904830
H	3.4309872	-2.0932968	-1.4902130
C	1.0382362	0.9933662	1.6057890
C	0.9693522	-0.4423898	2.0808690
H	1.3904592	-0.4864968	3.0912120
H	-0.0621498	-0.7987348	2.1588810
C	-3.9003688	-2.0027298	0.5526670
C	-3.0257318	-2.5664228	1.4848030
H	-3.3710968	-2.7534458	2.4977920
C	1.7805152	-1.4158468	1.2093060
H	1.7923262	-2.4091908	1.6610910
H	2.8188002	-1.0724198	1.1427000
C	5.4581612	-1.5925958	-0.9598050
H	5.8155582	-2.6115768	-1.0698140
C	-2.1474858	-2.1004528	-1.1246410
H	-1.7726358	-1.8982358	-2.1215220
C	0.2474612	2.9195212	0.3465040
H	-0.4935468	3.3669002	-0.3077170
C	4.5428232	1.0200602	-0.7160110
H	4.1771802	2.0398332	-0.6230180
C	1.3301922	3.6677902	0.7772170
H	1.4380182	4.6994552	0.4596690
C	-3.4474658	-1.7861528	-0.7541650
H	-4.1178268	-1.3352138	-1.4813990
C	2.1202732	1.7702462	2.0334870
H	2.8583472	1.3214522	2.6933520
C	6.3449442	-0.5778218	-0.6013500
H	7.3912542	-0.8051598	-0.4238810
C	2.2740112	3.0877322	1.6263630
H	3.1262992	3.6647432	1.9715570
C	5.8836282	0.7304312	-0.4841480
H	6.5694382	1.5278252	-0.2147610
C	-5.3115528	-1.6358868	0.9289760
H	-5.4454138	-1.6183398	2.0128600
H	-5.5711598	-0.6507448	0.5292800
H	-6.0239678	-2.3557978	0.5140460
H	-1.2441818	-0.1376678	0.7042980
C	-2.1531858	1.3848812	-0.5177730
C	-3.3779098	1.4196492	0.1572340
C	-2.0628228	1.9214452	-1.8044470
C	-4.4923888	2.0018962	-0.4375290
H	-3.4547988	0.9845782	1.1510290
C	-3.1841638	2.4891622	-2.4024300

H	-1.1189058	1.8474362	-2.3326490
C	-4.3978518	2.5349692	-1.7215950
H	-5.4353058	2.0353282	0.0998080
H	-3.1091838	2.8920902	-3.4072760
H	-5.2692998	2.9799662	-2.1909380

Energy (0K) = -1837.339143
Energy (0K) + ZPE = -1836.854219
Enthalpy (298K) = -1836.824325
Free Energy (298K) = -1836.914356

TS2_trans_1
Number of imaginary freguencies: 1

S	-2.3473593	2.0164131	-0.5773750
O	0.1914917	0.0143411	0.4411470
O	-2.6751623	2.7219771	-1.8086180
O	-3.3772363	1.5676781	0.3394020
N	-1.4789663	0.6465621	-1.0485140
C	-1.0155743	-0.1827099	0.0654970
C	-1.8233973	-1.1544949	0.6068720
H	-1.3597073	-1.6345279	1.4668250
C	1.4230387	-1.7348409	-0.4767980
C	-3.1376023	-1.6622449	0.2458690
C	2.1891837	-1.1720479	-1.5597540
C	-1.1967903	3.0214401	0.3339080
C	-0.9450243	2.7186321	1.6698970
H	-1.4778563	1.9028741	2.1477610
C	-3.8868453	-1.2825149	-0.8854120
H	-3.5054313	-0.5162439	-1.5473270
C	1.5222177	-0.5893429	-2.6721440
C	0.0307387	-0.3879019	-2.7026060
H	-0.3015943	-0.3723359	-3.7437440
H	-0.5015923	-1.2182929	-2.2263380
C	0.6663207	4.5316971	1.7410460
C	-0.0131023	3.4789731	2.3629730
H	0.1938497	3.2531751	3.4052860
C	-0.3958113	0.9290131	-2.0050990
H	-0.7851273	1.6355771	-2.7378620
H	0.4588447	1.3768251	-1.4860810
C	-5.1068263	-1.8848809	-1.1582870
H	-5.6658093	-1.5749269	-2.0359090
C	-0.5344813	4.0589961	-0.3126320
H	-0.7660943	4.2761151	-1.3503720
C	3.5966377	-1.2772279	-1.5808700
H	4.0997257	-1.7883829	-0.7680120
C	-3.6771623	-2.6613689	1.0782760
H	-3.1141543	-2.9677929	1.9563600

C	4.3341587	-0.7971019	-2.6488600
H	5.4139957	-0.8986389	-2.6521070
C	0.3953697	4.8094931	0.3992840
H	0.9153277	5.6269771	-0.0924180
C	2.2893167	-0.1165399	-3.7348670
H	1.7837687	0.3360801	-4.5833010
C	-5.6253223	-2.8704949	-0.3188180
H	-6.5842463	-3.3292139	-0.5377910
C	3.6777147	-0.2075489	-3.7286640
H	4.2469157	0.1685191	-4.5729270
C	-4.9012733	-3.2552999	0.8063530
H	-5.2905093	-4.0186409	1.4730300
C	1.6494267	5.3699661	2.5160360
H	2.1625117	4.7764581	3.2764690
H	2.4013027	5.8096011	1.8566620
H	1.1368917	6.1906861	3.0285830
H	0.4252467	-2.0957179	-0.7130370
C	1.9235497	-2.1867809	0.7977450
C	1.2749297	-3.2660339	1.4240760
C	2.9669227	-1.5305059	1.4788270
C	1.6965397	-3.7164429	2.6681760
H	0.4476377	-3.7528099	0.9152640
C	3.3730727	-1.9756289	2.7260920
H	3.4087357	-0.6409789	1.0426280
C	2.7502737	-3.0770829	3.3172030
H	1.1998507	-4.5599439	3.1356020
H	4.1679117	-1.4565009	3.2511800
H	3.0766417	-3.4240879	4.2922140

Energy (0K) = -1837.313697
Energy (0K) + ZPE = -1836.813307
Enthalpy (298K) = -1836.783055
Free Energy (298K) = -1836.875291

spiro_Int_1

Number of imaginary freguencies: 0

S	-0.1362226	-2.8324229	0.5831324
O	0.5630704	-1.1287009	-1.6358906
O	0.4026754	-3.7694299	-0.3807176
O	-0.2478376	-3.1894569	1.9900414
N	0.8759504	-1.4508949	0.5961224
C	1.1621814	-0.8437839	-0.6206016
C	2.2468484	0.2154451	-0.5708936
H	2.1227034	0.7936301	-1.4916606
C	-0.5783956	1.1940791	0.9541294
C	3.6428264	-0.3957529	-0.5600466
C	0.5253554	1.7217201	0.3856954

C	-1.7256956	-2.2700319	0.0413284
C	-2.6221586	-1.8051259	0.9981844
H	-2.3236086	-1.7491859	2.0402984
C	3.8765984	-1.7361939	-0.2343726
H	3.0491844	-2.4044849	-0.0155476
C	1.9316114	1.1740761	0.6473684
C	2.0688424	0.3415181	1.9296244
H	3.1382524	0.2685521	2.1468884
H	1.6104744	0.8877801	2.7591694
C	-4.2915946	-1.5628109	-0.7404146
C	-3.9024826	-1.4447939	0.5948554
H	-4.6087746	-1.0636939	1.3263044
C	1.5124574	-1.0989389	1.8783694
H	0.7587394	-1.2483569	2.6518104
H	2.3161964	-1.8129209	2.0818604
C	5.1706284	-2.2528169	-0.2326676
H	5.3259864	-3.2970729	0.0183954
C	-2.0788136	-2.3911989	-1.2996946
H	-1.3475706	-2.7542189	-2.0123046
C	0.4465224	2.8350681	-0.5668716
H	-0.5122116	3.0690651	-1.0144286
C	4.7368004	0.3959631	-0.9323856
H	4.5665494	1.4233501	-1.2377546
C	1.5069524	3.6366601	-0.7776096
H	1.4190714	4.4996691	-1.4296666
C	-3.3655806	-2.0342669	-1.6780966
H	-3.6604786	-2.1233409	-2.7201066
C	2.9428754	2.2990001	0.6546774
H	3.8679564	2.1357381	1.2010014
C	6.2518564	-1.4451049	-0.5648386
H	7.2587834	-1.8496979	-0.5636206
C	2.7544914	3.4181661	-0.0552356
H	3.5201374	4.1877781	-0.0840416
C	6.0281414	-0.1190119	-0.9260886
H	6.8601384	0.5143171	-1.2174476
C	-5.6895996	-1.2026579	-1.1676786
H	-6.1899146	-0.5996009	-0.4068156
H	-5.6805516	-0.6353449	-2.1026986
H	-6.2862686	-2.1053039	-1.3357376
H	-0.4667936	0.4761931	1.7650614
C	-1.9740496	1.4772151	0.5562354
C	-2.9341856	1.8216321	1.5138384
C	-2.3707576	1.3370161	-0.7790756
C	-4.2516216	2.0660751	1.1394324
H	-2.6386096	1.9094421	2.5558634
C	-3.6861236	1.5888441	-1.1534706

H	-1.6427786	0.9930991	-1.5101576
C	-4.6291656	1.9595821	-0.1976306
H	-4.9834436	2.3453671	1.8913154
H	-3.9796176	1.4752311	-2.1930996
H	-5.6559506	2.1553421	-0.4916336

Energy (0K) = -1837.381669
Energy (0K) + ZPE = -1836.875649
Enthalpy (298K) = -1836.846456
Free Energy (298K) = -1836.932646

2a_cis_1

Number of imaginary freguencies: 0

S	2.2054711	-1.8942884	-1.9532599
O	-0.3295299	-0.5878574	-2.1042869
O	1.9156851	-2.0782114	-3.3602349
O	3.2514431	-2.6741324	-1.3074319
N	0.8027591	-2.2238304	-1.0073469
C	-0.2982749	-1.3894404	-1.1945639
C	-1.3526989	-1.4847264	-0.1052879
H	-1.3728179	-2.5117864	0.2484231
C	-0.9459089	-0.5702184	1.1271761
H	-1.6561039	-0.8774764	1.9084861
C	0.4641281	-0.7446334	1.7330771
C	1.0524601	0.3714466	2.3454051
H	0.5358141	1.3231676	2.3159651
C	2.2804581	0.3053396	2.9948261
H	2.6885731	1.1981346	3.4586771
C	2.9738811	-0.8967964	3.0433621
H	3.9391811	-0.9670884	3.5340741
C	2.3970361	-2.0213664	2.4660681
H	2.9163491	-2.9749414	2.5105101
C	1.1502411	-1.9737284	1.8355361
C	0.6002171	-3.2750114	1.3091011
H	-0.4480989	-3.3838724	1.5991781
H	1.1250821	-4.1006324	1.7978141
C	0.7825991	-3.4611224	-0.2149229
H	1.7487181	-3.9323954	-0.3914749
H	0.0115931	-4.1268704	-0.6161999
C	-2.7741739	-1.1932544	-0.5516579
C	-3.1269899	-0.1696024	-1.4371789
H	-2.3534979	0.4429796	-1.8829319
C	-4.4658159	0.0613786	-1.7389879
H	-4.7241169	0.8578786	-2.4296779
C	-5.4702629	-0.7079174	-1.1580559
H	-6.5120319	-0.5169924	-1.3956819
C	-5.1294849	-1.7222104	-0.2693619

H	-5.9010379	-2.3314534	0.1906531
C	-3.7903789	-1.9600884	0.0249231
H	-3.5282479	-2.7574224	0.7180711
C	-1.2603159	0.8798416	0.7948041
C	-0.4358969	1.6266896	-0.0548409
H	0.4828351	1.1948666	-0.4391569
C	-0.7810719	2.9259276	-0.4097129
H	-0.1330599	3.4839806	-1.0801589
C	-1.9506159	3.5027396	0.0836141
H	-2.2199269	4.5163586	-0.1960919
C	-2.7686569	2.7725266	0.9390131
H	-3.6811869	3.2108756	1.3300371
C	-2.4221259	1.4697646	1.2902511
H	-3.0771539	0.8922356	1.9379591
C	2.5005911	-0.1772184	-1.6277489
C	2.1663271	0.7678476	-2.5905029
H	1.7233601	0.4430686	-3.5251149
C	2.3847741	2.1108986	-2.3046459
H	2.1261841	2.8639336	-3.0437729
C	2.9177441	2.5087526	-1.0743669
C	3.2557031	1.5289846	-0.1352629
H	3.6708671	1.8236776	0.8246051
C	3.0531731	0.1810686	-0.4022249
H	3.3072591	-0.5801384	0.3297571
C	3.0903431	3.9674146	-0.7423099
H	4.0466661	4.1492376	-0.2453439
H	2.2971871	4.2969666	-0.0624029
H	3.0447781	4.5887436	-1.6391639

Energy (0K) = -1837.424244
Energy (0K) + ZPE = -1836.916473
Enthalpy (298K) = -1836.886808
Free Energy (298K) = -1836.974938

2a_trans_1

Number of imaginary freguencies: 0

S	1.6764615	-2.7863852	-0.3461979
O	-1.2780335	-2.7352672	1.8478041
O	0.6750685	-3.2440252	-1.2963139
O	2.7815555	-3.6135312	0.1021541
N	0.8145125	-2.4067672	1.0347501
C	-0.5900025	-2.2208512	1.0023171
C	-1.0985575	-1.1064842	0.1093601
H	-0.4102965	-0.9487932	-0.7236199
C	-1.0266175	0.1581588	1.0470121
H	-1.4507765	-0.1523572	2.0099531
C	0.4147415	0.6458708	1.3165251

C	0.8991085	1.7286408	0.5706331
H	0.2736415	2.1562828	-0.2047339
C	2.1403725	2.3024228	0.8156361
H	2.4667235	3.1487748	0.2186121
C	2.9422315	1.8021658	1.8333501
H	3.9057895	2.2511008	2.0542431
C	2.4964165	0.7069598	2.5598441
H	3.1229775	0.2944748	3.3476441
C	1.2538425	0.1083418	2.3194181
C	0.9420775	-1.1391672	3.1184561
H	-0.1238735	-1.2324932	3.3421521
H	1.4621675	-1.0991502	4.0801701
C	1.4092755	-2.4051662	2.3757301
H	2.4945805	-2.4069982	2.2549041
H	1.1178145	-3.3102272	2.9150281
C	-2.4677645	-1.4533662	-0.4313169
C	-2.5658275	-1.9561602	-1.7287599
H	-1.6601755	-2.0844272	-2.3174219
C	-3.8021865	-2.3162152	-2.2569489
H	-3.8649365	-2.7054972	-3.2681309
C	-4.9526305	-2.1811612	-1.4862419
H	-5.9189405	-2.4582012	-1.8955649
C	-4.8587735	-1.6938172	-0.1847349
H	-5.7521825	-1.5934612	0.4233381
C	-3.6229835	-1.3350842	0.3418781
H	-3.5578075	-0.9556202	1.3568611
C	-1.9027275	1.2962568	0.5468901
C	-2.0856555	1.5625648	-0.8122929
H	-1.6258115	0.9168748	-1.5561749
C	-2.8635175	2.6391558	-1.2265479
H	-2.9991985	2.8276108	-2.2867639
C	-3.4741025	3.4655298	-0.2867839
H	-4.0854845	4.3018938	-0.6096429
C	-3.2995525	3.2082298	1.0696001
H	-3.7729495	3.8440968	1.8110431
C	-2.5185335	2.1317358	1.4794821
H	-2.3794685	1.9355248	2.5400021
C	2.3793615	-1.2938872	-1.0105449
C	1.7929795	-0.7059432	-2.1268359
H	0.9147505	-1.1608802	-2.5737909
C	2.3689425	0.4385528	-2.6678149
H	1.9174775	0.9044038	-3.5389529
C	3.5260215	0.9890788	-2.1144849
C	4.1044885	0.3638598	-1.0051329
H	5.0065805	0.7828568	-0.5674649
C	3.5426075	-0.7756682	-0.4477119

H	4.0073105	-1.2709472	0.3984541
C	4.1611005	2.2205538	-2.7040229
H	5.0669385	1.9610468	-3.2609919
H	4.4506235	2.9242588	-1.9184579
H	3.4808245	2.7307248	-3.3888469

Energy (0K) = -1837.419198
Energy (0K) + ZPE = -1836.912260
Enthalpy (298K) = -1836.882292
Free Energy (298K) = -1836.972245

ωB97XD (SMD, chlorobenzene) for optimization, Cartesian Coordinates and Energies in Hartree

Cartesian coordinates and imaginary frequency of the calculated structures using ωB97XD.

6a_2

Number of imaginary freguencies: 0

S	0.8144175	-2.7191396	0.0485937
O	0.0706875	0.1545364	-1.2196933
O	1.1094565	-3.0840706	-1.3259273
O	1.3225255	-3.4863566	1.1749307
N	1.3759975	-1.1523946	0.2571687
C	1.3181725	-0.2399526	-0.8259953
C	2.3953865	0.2852844	-1.4138593
H	2.2093395	1.1167014	-2.0871773
C	-0.7641105	0.6781914	-0.1732003
C	3.7943925	-0.1066926	-1.1759253
C	-0.0654565	1.8338314	0.5159957
C	-0.9539555	-2.6272686	0.2135267
C	-1.5202705	-2.6671256	1.4832407
H	-0.8966905	-2.8675956	2.3480827
C	4.1877095	-1.4461916	-1.0656753
H	3.4499025	-2.2299556	-1.1984533
C	0.7359145	1.6148124	1.6496537
C	0.9012835	0.2534004	2.2956277
H	1.3030545	0.3958974	3.3043277
H	-0.0640185	-0.2497826	2.4193587
C	-3.6907575	-2.2021546	0.5036967
C	-2.8872625	-2.4517696	1.6189617
H	-3.3363705	-2.4769646	2.6074227
C	1.8657655	-0.6906936	1.5591517
H	2.0528755	-1.5786576	2.1635737
H	2.8281145	-0.1957866	1.3946877
C	5.5171855	-1.7685796	-0.8182473
H	5.8061335	-2.8115206	-0.7350163
C	-1.7343895	-2.4023336	-0.9166333
H	-1.2680265	-2.3638306	-1.8937223

C	-0.1345005	3.1074994	-0.0495703
H	-0.7558105	3.2580504	-0.9272703
C	4.7673355	0.8937074	-1.0614813
H	4.4724255	1.9352304	-1.1568643
C	0.5703165	4.1726194	0.4944097
H	0.5011515	5.1572424	0.0436617
C	-3.0969015	-2.1942636	-0.7616483
H	-3.7055415	-1.9824486	-1.6356783
C	1.4372805	2.6988514	2.1858147
H	2.0553095	2.5428304	3.0659887
C	6.4743595	-0.7658756	-0.6880763
H	7.5119305	-1.0217426	-0.4979713
C	1.3604475	3.9652894	1.6203367
H	1.9129905	4.7890124	2.0611057
C	6.0962745	0.5680174	-0.8157993
H	6.8376815	1.3557934	-0.7253853
C	-5.1661115	-1.9388166	0.6450577
H	-5.4603505	-1.8550456	1.6938427
H	-5.4350125	-1.0079596	0.1368517
H	-5.7515585	-2.7457946	0.1929627
H	-0.9432365	-0.1174506	0.5561177
C	-2.1059845	1.0193034	-0.7788643
C	-3.1548015	1.3602164	0.0775287
C	-2.3370535	0.9608024	-2.1500803
C	-4.4198795	1.6289904	-0.4288553
H	-2.9757415	1.4099944	1.1483307
C	-3.6079625	1.2248474	-2.6578083
H	-1.5246595	0.6894344	-2.8134583
C	-4.6523885	1.5553864	-1.8016163
H	-5.2263725	1.8939134	0.2480017
H	-3.7793865	1.1691124	-3.7283003
H	-5.6412575	1.7594154	-2.1997563

Energy (0K) = -1837.56529
Energy (0K) + ZPE = -1837.056472
Enthalpy (298K) = -1837.026540
Free Energy (298K) = -1837.117719

6a_iso_2

Number of imaginary freguencies: 0

S	-1.4057028	-2.3892733	-0.4271886
O	-0.1228368	0.5994257	-0.6852396
O	-2.1575228	-2.4782803	-1.6713696
O	-1.6202248	-3.3536603	0.6405154
N	-1.6842878	-0.8855243	0.2583514
C	-1.4417378	0.2911877	-0.4898496
C	-2.3871038	1.1278567	-0.9319366

H	-2.0189418	2.0593317	-1.3554916
C	0.5437392	1.3311567	0.3587304
H	-0.1467128	2.1138407	0.7020244
C	0.9392442	0.4647607	1.5614704
C	2.2785532	0.1111077	1.7471744
H	3.0197882	0.4538477	1.0350114
C	2.6843622	-0.6794233	2.8148724
H	3.7335562	-0.9323193	2.9307964
C	1.7432832	-1.1395043	3.7271004
H	2.0424282	-1.7604173	4.5654934
C	0.4107562	-0.7829633	3.5639474
H	-0.3284358	-1.1300373	4.2812294
C	-0.0105298	0.0216757	2.5022704
C	-1.4880528	0.3198527	2.3988514
H	-1.6739648	1.2964207	1.9418104
H	-1.9271008	0.3621287	3.4005444
C	-2.2400968	-0.7552203	1.6076974
H	-2.1397318	-1.7262563	2.0941464
H	-3.3063838	-0.5071593	1.5509814
C	-3.8486368	0.9789297	-0.8304596
C	-4.5037528	-0.2492373	-0.9850576
H	-3.9310188	-1.1358723	-1.2337516
C	-5.8854398	-0.3263973	-0.8493436
H	-6.3790338	-1.2849073	-0.9743756
C	-6.6347518	0.8130537	-0.5688206
H	-7.7128458	0.7447087	-0.4634436
C	-5.9946618	2.0418347	-0.4357076
H	-6.5697148	2.9386547	-0.2275396
C	-4.6140108	2.1234647	-0.5720376
H	-4.1170868	3.0843227	-0.4670096
C	1.7307402	2.0048687	-0.2952526
C	2.3424712	1.4387277	-1.4135516
H	1.9293602	0.5321647	-1.8407806
C	3.4706322	2.0344577	-1.9691966
H	3.9375722	1.5880567	-2.8420786
C	3.9963312	3.1982877	-1.4147476
H	4.8756292	3.6620907	-1.8502606
C	3.3846412	3.7679907	-0.3014976
H	3.7851042	4.6777787	0.1343164
C	2.2568052	3.1727837	0.2547694
H	1.7875382	3.6138257	1.1304864
C	0.3133522	-2.3846803	-0.8739526
C	0.6650732	-2.1718453	-2.2020176
H	-0.1102948	-2.0524773	-2.9496836
C	2.0109552	-2.1085693	-2.5390736
H	2.2937052	-1.9341943	-3.5732556

C	3.0035482	-2.2501063	-1.5655836
C	2.6170362	-2.4961173	-0.2460506
H	3.3748492	-2.6132823	0.5226164
C	1.2770992	-2.5602763	0.1105074
H	0.9866812	-2.7215753	1.1435474
C	4.4577672	-2.0814553	-1.9174256
H	4.6474002	-2.3153653	-2.9679436
H	5.0966182	-2.7194863	-1.3013626
H	4.7639962	-1.0427103	-1.7469246

Energy (0K) = -1837.556936
Energy (0K) + ZPE = -1837.047952
Enthalpy (298K) = -1837.018339
Free Energy (298K) = -1837.107346

TS_cis_2
Number of imaginary freguencies: 1

S	0.1289303	-3.0995856	-0.8012533
O	-0.0704917	-0.2290976	-1.4799803
O	0.1550673	-3.1719496	-2.2472283
O	0.5216603	-4.2322006	0.0260107
N	1.1640023	-1.8473906	-0.3565283
C	1.0657353	-0.6031466	-1.0833343
C	2.1808453	0.2161144	-1.1818043
H	1.9319943	1.2121274	-1.5345203
C	-1.0453617	0.9120794	0.3719207
C	3.5905133	-0.0450306	-0.9743023
C	0.1752433	1.4669054	0.7945987
C	-1.5174527	-2.6404956	-0.2871043
C	-1.9218827	-2.9157656	1.0146037
H	-1.2677217	-3.4710846	1.6781857
C	4.1639153	-1.3305696	-0.9325273
H	3.5335533	-2.1982316	-1.0964353
C	1.1449333	0.6713714	1.4842287
C	0.9037003	-0.7615556	1.9024187
H	1.2861813	-0.8836316	2.9218477
H	-0.1592947	-1.0061676	1.9420987
C	-4.0277317	-1.7953206	0.5750037
C	-3.1732817	-2.4807106	1.4416787
H	-3.4915847	-2.6851466	2.4600307
C	1.6345753	-1.7938926	1.0285167
H	1.5204023	-2.7918716	1.4528447
H	2.7055883	-1.5717736	1.0235107
C	5.5236373	-1.4907626	-0.7108283
H	5.9465773	-2.4902846	-0.6880043
C	-2.3547997	-1.9717206	-1.1737003
H	-2.0064617	-1.7528236	-2.1747573

C	0.5203483	2.8131334	0.4774797
H	-0.2054167	3.4239814	-0.0474803
C	4.4440653	1.0626004	-0.8182953
H	4.0213533	2.0609634	-0.8820903
C	1.7180273	3.3601014	0.8670037
H	1.9420923	4.3959454	0.6354247
C	-3.6042397	-1.5584366	-0.7368913
H	-4.2524737	-1.0131236	-1.4162523
C	2.3442063	1.2715474	1.8961947
H	3.0641093	0.6730544	2.4467327
C	6.3505423	-0.3817566	-0.5292913
H	7.4135433	-0.5161306	-0.3569693
C	2.6388853	2.5839214	1.5926537
H	3.5784113	3.0171034	1.9193827
C	5.8046093	0.8963464	-0.5915163
H	6.4413943	1.7675854	-0.4714243
C	-5.3879537	-1.3315266	1.0264057
H	-5.4362017	-1.2187276	2.1126307
H	-5.6429547	-0.3721276	0.5668097
H	-6.1594167	-2.0512486	0.7329907
H	-1.3605857	-0.0294556	0.8095367
C	-2.1012577	1.6115584	-0.3644993
C	-3.3897077	1.6454854	0.1846387
C	-1.8737147	2.2183214	-1.6051233
C	-4.4189877	2.3086464	-0.4706533
H	-3.5764597	1.1539264	1.1350487
C	-2.9105507	2.8656314	-2.2667703
H	-0.8966547	2.1114584	-2.0625163
C	-4.1810617	2.9209154	-1.6993443
H	-5.4080157	2.3449974	-0.0245393
H	-2.7291167	3.3184344	-3.2360733
H	-4.9878107	3.4284884	-2.2183943

Energy (0K) = -1837.505693
Energy (0K) + ZPE = -1837.000231
Enthalpy (298K) = -1836.970468
Free Energy (298K) = -1837.059350

TS2_trans_2

Number of imaginary freguencies: 1

S	-1.4461653	-2.1024544	-0.9952154
O	0.1329417	0.7218936	0.6512086
O	-1.9140763	-2.3247254	-2.3532234
O	-0.7129183	-3.1205004	-0.2740154
N	-0.5015653	-0.6734794	-1.0411244
C	0.3274257	-0.3862674	0.1166906
C	1.4207247	-1.1968444	0.5218336

H	1.6530267	-1.0406184	1.5693616
C	1.6979257	1.9331576	-0.6044894
C	2.0084007	-2.3823394	-0.1020444
C	0.8302847	3.0707536	-0.8147984
C	-2.8494713	-1.6422624	0.0054786
C	-2.6673613	-1.4595244	1.3746476
H	-1.6937943	-1.6236764	1.8236016
C	2.0227167	-2.5873824	-1.4914824
H	1.4879087	-1.8982824	-2.1374524
C	-0.2960743	2.8667776	-1.6500064
C	-0.5937863	1.5481536	-2.3399064
H	-1.2423103	1.7809316	-3.1896474
H	0.3017077	1.1017256	-2.7815444
C	-5.0110323	-0.8662474	1.5808136
C	-3.7489633	-1.0684324	2.1499846
H	-3.6125103	-0.9173124	3.2167366
C	-1.3293113	0.4731106	-1.5000944
H	-2.0934783	0.0405576	-2.1445114
H	-1.8190193	0.9414516	-0.6427854
C	2.6893277	-3.6710944	-2.0432634
H	2.6746477	-3.8164344	-3.1190284
C	-4.0903543	-1.4469864	-0.5867504
H	-4.2057923	-1.6028884	-1.6537404
C	1.0556337	4.3345126	-0.2522674
H	1.9575577	4.5072826	0.3240166
C	2.7113507	-3.2884064	0.7032886
H	2.7234427	-3.1375314	1.7794136
C	0.1648217	5.3771546	-0.4615044
H	0.3549967	6.3496346	-0.0196704
C	-5.1653023	-1.0629344	0.2075036
H	-6.1390513	-0.9124734	-0.2494314
C	-1.1791123	3.9281506	-1.8376364
H	-2.0529673	3.7823806	-2.4668354
C	3.3727377	-4.5715364	-1.2276434
H	3.8903877	-5.4202344	-1.6629334
C	-0.9649273	5.1692916	-1.2458884
H	-1.6713093	5.9760526	-1.4130594
C	3.3816317	-4.3730204	0.1489956
H	3.9114387	-5.0655944	0.7959966
C	-6.1817913	-0.4706194	2.4425136
H	-5.8811053	0.2402476	3.2167286
H	-6.9782063	-0.0139354	1.8500116
H	-6.6033093	-1.3472554	2.9458846
H	1.6471837	1.2116646	-1.4138044
C	2.6307297	1.5684706	0.3556146
C	3.2457507	0.2959566	0.1420036

C	2.8378017	2.2102316	1.6209026
C	4.2002217	-0.1955444	1.0727796
H	3.2362677	-0.1284734	-0.8547594
C	3.7017257	1.6772266	2.5299626
H	2.2744137	3.1023526	1.8658196
C	4.4123927	0.4736256	2.2444606
H	4.7159667	-1.1243684	0.8550246
H	3.8495267	2.1655006	3.4876996
H	5.1220487	0.0914326	2.9715826

Energy (0K) = -1837.478131
Energy (0K) + ZPE = -1836.972473
Enthalpy (298K) = -1836.942599
Free Energy (298K) = -1837.032761

spiro_Int_2

Number of imaginary freguencies: 0

S	-0.1402223	-2.9268116	0.5561159
O	0.6255867	-1.2093636	-1.6527381
O	0.3978907	-3.8579456	-0.4125531
O	-0.2541483	-3.2967396	1.9588179
N	0.8739777	-1.5385836	0.5875809
C	1.1780437	-0.9127026	-0.6127611
C	2.2329067	0.1769634	-0.5397701
H	2.0850647	0.7594984	-1.4524041
C	-0.6051633	1.1723064	0.9359629
C	3.6488837	-0.3854486	-0.5648071
C	0.5195247	1.6985154	0.4133859
C	-1.7288453	-2.3466526	0.0217779
C	-2.6294513	-1.9042316	0.9862669
H	-2.3427843	-1.8889086	2.0318219
C	3.9611707	-1.6799076	-0.1404631
H	3.1791647	-2.3562686	0.1870039
C	1.9123077	1.1184644	0.6875719
C	2.0259687	0.2619924	1.9569489
H	3.0932697	0.1438394	2.1607639
H	1.6012647	0.8112904	2.8017539
C	-4.2823843	-1.5670096	-0.7515131
C	-3.8999523	-1.5112156	0.5902299
H	-4.6034073	-1.1492606	1.3332959
C	1.4082367	-1.1508296	1.9048859
H	0.5862857	-1.2364946	2.6160609
H	2.1550287	-1.8867806	2.2142859
C	5.2739147	-2.1429166	-0.1737021
H	5.4895037	-3.1545536	0.1546839
C	-2.0768833	-2.4048396	-1.3237451
H	-1.3514803	-2.7442606	-2.0518421

C	0.4787097	2.8465814	-0.4988561
H	-0.4658653	3.1082244	-0.9600911
C	4.6834907	0.4168834	-1.0587421
H	4.4536227	1.4119244	-1.4252101
C	1.5521987	3.6395414	-0.6622711
H	1.4876997	4.5257934	-1.2852751
C	-3.3551023	-2.0140216	-1.6971791
H	-3.6377603	-2.0573986	-2.7449941
C	2.9445477	2.2245244	0.7340039
H	3.8654337	2.0254414	1.2738189
C	6.2969287	-1.3242396	-0.6366071
H	7.3191277	-1.6878716	-0.6637111
C	2.7853957	3.3713044	0.0639359
H	3.5682057	4.1234134	0.0678909
C	5.9946857	-0.0423306	-1.0871181
H	6.7798297	0.6002344	-1.4731011
C	-5.6767213	-1.1796476	-1.1657951
H	-6.1087103	-0.4620756	-0.4644941
H	-5.6845833	-0.7293406	-2.1618911
H	-6.3285973	-2.0596516	-1.1945731
H	-0.5265763	0.4230354	1.7184119
C	-1.9879163	1.4974114	0.5273449
C	-2.9459143	1.8587914	1.4796149
C	-2.3765133	1.3901364	-0.8125781
C	-4.2514703	2.1474554	1.0974759
H	-2.6595643	1.9266074	2.5254139
C	-3.6798123	1.6846614	-1.1950981
H	-1.6540693	1.0407714	-1.5442021
C	-4.6193083	2.0712354	-0.2436551
H	-4.9811603	2.4394904	1.8461859
H	-3.9660723	1.5924874	-2.2382021
H	-5.6374323	2.2994814	-0.5433651

Energy (0K) = -1837.549305
Energy (0K) + ZPE = -1837.040942
Enthalpy (298K) = -1837.010864
Free Energy (298K) = -1837.100978

2a_cis_2

Number of imaginary freguencies: 0

S	2.2516417	-1.9449795	-1.8957265
O	-0.2927143	-0.6513375	-2.1140565
O	1.9854277	-2.1788105	-3.2996935
O	3.2890517	-2.7021805	-1.2108355
N	0.8305067	-2.2495535	-0.9579555
C	-0.2684143	-1.4249495	-1.1777445
C	-1.3384683	-1.4886495	-0.1001575

H	-1.3584723	-2.5037785	0.2847315
C	-0.9654703	-0.5456475	1.1215185
H	-1.6829843	-0.8532955	1.8940795
C	0.4323597	-0.6968605	1.7613795
C	1.0002297	0.4354175	2.3606575
H	0.4787507	1.3821885	2.3029975
C	2.2174637	0.3951335	3.0288265
H	2.6116807	1.3011565	3.4782845
C	2.9198687	-0.7986535	3.1125925
H	3.8783167	-0.8498215	3.6185535
C	2.3625767	-1.9392605	2.5510235
H	2.8913037	-2.8853235	2.6250995
C	1.1265617	-1.9171035	1.8988935
C	0.5998577	-3.2342445	1.3878025
H	-0.4534353	-3.3480745	1.6569865
H	1.1173567	-4.0471645	1.9052305
C	0.8137377	-3.4586245	-0.1265555
H	1.7899047	-3.9192385	-0.2714185
H	0.0627707	-4.1512905	-0.5199685
C	-2.7579233	-1.2238755	-0.5717555
C	-3.1081953	-0.2443995	-1.5049365
H	-2.3323593	0.3445285	-1.9751495
C	-4.4442263	-0.0279305	-1.8245885
H	-4.6989983	0.7349215	-2.5533485
C	-5.4506813	-0.7705115	-1.2147235
H	-6.4917263	-0.5918225	-1.4647025
C	-5.1130093	-1.7439165	-0.2806595
H	-5.8865563	-2.3331235	0.2017555
C	-3.7762293	-1.9668595	0.0313195
H	-3.5193783	-2.7309215	0.7623055
C	-1.2953863	0.8977475	0.7729605
C	-0.4845983	1.6456505	-0.0865455
H	0.4262247	1.2153995	-0.4839625
C	-0.8321003	2.9439675	-0.4366375
H	-0.1900273	3.5024405	-1.1113805
C	-1.9954023	3.5202725	0.0690745
H	-2.2664233	4.5351345	-0.2046665
C	-2.8050263	2.7872955	0.9292645
H	-3.7141043	3.2247735	1.3293335
C	-2.4539053	1.4858925	1.2770545
H	-3.1021983	0.9104045	1.9320355
C	2.5426337	-0.2175855	-1.6169345
C	2.2188867	0.7021475	-2.6068975
H	1.7863357	0.3570525	-3.5375925
C	2.4290837	2.0525645	-2.3582275
H	2.1714247	2.7802745	-3.1218235

C	2.9458517	2.4889985	-1.1353405
C	3.2785027	1.5360285	-0.1679675
H	3.6819897	1.8557805	0.7879065
C	3.0830737	0.1822775	-0.3989905
H	3.3322437	-0.5507265	0.3600425
C	3.1041767	3.9571025	-0.8407785
H	4.0520717	4.1606595	-0.3351755
H	2.2992157	4.2993785	-0.1809385
H	3.0673297	4.5559285	-1.7538755

Energy (0K) = -1837.594085
Energy (0K) + ZPE = -1837.083842
Enthalpy (298K) = -1837.054466
Free Energy (298K) = -1837.141507

2a_trans_2

Number of imaginary freguencies: 0

S	1.7476191	-2.7979195	-0.4207110
O	-1.1804989	-2.8341115	1.8064970
O	0.7499621	-3.2802045	-1.3606260
O	2.8794251	-3.5998305	0.0056660
N	0.8948671	-2.4411535	0.9760760
C	-0.5121689	-2.2796755	0.9674630
C	-1.0500369	-1.1530465	0.1085950
H	-0.3794929	-0.9712495	-0.7313020
C	-0.9836469	0.1095535	1.0563340
H	-1.3884219	-0.2160005	2.0209730
C	0.4469661	0.6322345	1.3197870
C	0.8900321	1.7541565	0.6096380
H	0.2464931	2.1898815	-0.1444970
C	2.1122961	2.3603135	0.8643730
H	2.4029241	3.2399075	0.2983450
C	2.9412511	1.8500485	1.8530350
H	3.8927251	2.3207365	2.0793310
C	2.5380251	0.7175245	2.5445370
H	3.1876561	0.3008235	3.3104830
C	1.3124051	0.0896865	2.2974610
C	1.0396151	-1.1831535	3.0708990
H	-0.0199979	-1.2994325	3.3120870
H	1.5709251	-1.1549105	4.0267790
C	1.5158891	-2.4300175	2.3033350
H	2.5973211	-2.4049395	2.1607860
H	1.2634151	-3.3446985	2.8454410
C	-2.4257569	-1.4864475	-0.4253200
C	-2.5633729	-1.8190825	-1.7721140
H	-1.6816039	-1.8462665	-2.4070150
C	-3.8105819	-2.1300495	-2.3042300

H	-3.9015139	-2.3826505	-3.3558910
C	-4.9365089	-2.1165265	-1.4879200
H	-5.9122119	-2.3522805	-1.9007100
C	-4.8043669	-1.8019405	-0.1380300
H	-5.6771899	-1.7967355	0.5071870
C	-3.5574439	-1.4924525	0.3910390
H	-3.4643569	-1.2534215	1.4445210
C	-1.9010729	1.2239875	0.5770360
C	-2.0562809	1.5408075	-0.7745640
H	-1.5298429	0.9643855	-1.5292290
C	-2.8890389	2.5806755	-1.1709050
H	-3.0043599	2.8035235	-2.2268500
C	-3.5819539	3.3243035	-0.2199590
H	-4.2384999	4.1310195	-0.5295620
C	-3.4322519	3.0206915	1.1288840
H	-3.9695419	3.5917585	1.8794700
C	-2.5984049	1.9779085	1.5201590
H	-2.4842999	1.7464735	2.5759930
C	2.3976531	-1.2743745	-1.0702500
C	1.7793591	-0.6749805	-2.1617540
H	0.9146961	-1.1453085	-2.6165910
C	2.3046011	0.5053835	-2.6746700
H	1.8251761	0.9754565	-3.5280960
C	3.4443141	1.0852355	-2.1173800
C	4.0600961	0.4470315	-1.0364920
H	4.9513781	0.8833475	-0.5956100
C	3.5469141	-0.7266455	-0.5081610
H	4.0384941	-1.2204555	0.3219350
C	4.0003381	2.3802205	-2.6467740
H	5.0746231	2.3000895	-2.8361300
H	3.8564561	3.1839595	-1.9165250
H	3.5108101	2.6788375	-3.5762800

Energy (0K) = -1837.588939

Energy (0K) + ZPE = -1837.079721

Enthalpy (298K) = -1837.050005

Free Energy (298K) = -1837.138962
